# Proteomic Profiling and Protein Identification by MALDI-TOF Mass Spectrometry in Unsequenced Parasitic Nematodes

**DOI:** 10.1371/journal.pone.0033590

**Published:** 2012-03-29

**Authors:** Paul Millares, E. James LaCourse, Samirah Perally, Deborah A. Ward, Mark C. Prescott, Jane E. Hodgkinson, Peter M. Brophy, Huw H. Rees

**Affiliations:** 1 Institute of Integrative Biology, University of Liverpool, Liverpool, United Kingdom; 2 Liverpool School of Tropical Medicine, Liverpool, United Kingdom; 3 Institute of Infection and Global Health, University of Liverpool, Liverpool, United Kingdom; 4 Institute of Biological, Environmental and Rural Sciences (IBERS), Aberystwyth University, Aberystwyth, United Kingdom; Stanford University, United States of America

## Abstract

Lack of genomic sequence data and the relatively high cost of tandem mass spectrometry have hampered proteomic investigations into helminths, such as resolving the mechanism underpinning globally reported anthelmintic resistance. Whilst detailed mechanisms of resistance remain unknown for the majority of drug-parasite interactions, gene mutations and changes in gene and protein expression are proposed key aspects of resistance. Comparative proteomic analysis of drug-resistant and -susceptible nematodes may reveal protein profiles reflecting drug-related phenotypes. Using the gastro-intestinal nematode, *Haemonchus contortus* as case study, we report the application of freely available expressed sequence tag (EST) datasets to support proteomic studies in unsequenced nematodes. EST datasets were translated to theoretical protein sequences to generate a searchable database. In conjunction with matrix-assisted laser desorption ionisation time-of-flight mass spectrometry (MALDI-TOF-MS), Peptide Mass Fingerprint (PMF) searching of databases enabled a cost-effective protein identification strategy. The effectiveness of this approach was verified in comparison with MS/MS *de novo* sequencing with searching of the same EST protein database and subsequent searches of the NCBInr protein database using the Basic Local Alignment Search Tool (BLAST) to provide protein annotation. Of 100 proteins from 2-DE gel spots, 62 were identified by MALDI-TOF-MS and PMF searching of the EST database. Twenty randomly selected spots were analysed by electrospray MS/MS and MASCOT Ion Searches of the *same* database. The resulting sequences were subjected to BLAST searches of the NCBI protein database to provide annotation of the proteins and confirm concordance in protein identity from both approaches. Further confirmation of protein identifications from the MS/MS data were obtained by *de novo* sequencing of peptides, followed by FASTS algorithm searches of the EST putative protein database. This study demonstrates the cost-effective use of available EST databases and inexpensive, accessible MALDI-TOF MS in conjunction with PMF for reliable protein identification in unsequenced organisms.

## Introduction

The identification of proteins in proteomic studies requires either (i) Tandem Mass Spectrometry (MS/MS) [1], which is relatively expensive, followed by either ion searches of databases or limited *de novo* sequencing of peptides [2] before database searching; or (ii) Matrix-Assisted Laser Desorption Ionization Time-Of-Flight mass spectrometry (MALDI-TOF MS) [3] (significantly less expensive than MS/MS), followed by Peptide Mass Fingerprint (PMF) searching of databases [4]. The costly MS/MS strategy is typically used where little or no genome sequence is available for the organism of interest, whilst the significantly cheaper strategy of MALDI-TOF MS/PMF can be employed where genome sequence of the particular organism under study is available. Unfortunately, parasite proteomics is currently limited by lack of genomic sequence availability and inadequate representation in annotated, non-redundant (nr) protein databases. Expensive and time-consuming methods of *de novo* peptide MS/MS sequencing and identity alignment methods such as Basic Local Alignment Search Tool (BLAST) are typically necessary to infer protein homology for non-sequenced parasites. Thus, proteomic profiles of the majority of economically important parasites are poorly represented and remain to be resolved.

Gastro-intestinal nematode infections involving parasites such as *Haemonchus contortus*, *Teladorsagia circumcincta*, *Trichostrongylus* spp. or *Nematodirus* spp. pose a great health and economic burden to the sheep industry worldwide [5]–[7]. In the absence of viable vaccines, control of these infections has relied heavily on the use of broad-spectrum anthelmintics for 50 years in intensively farmed livestock [8]. However, frequent anthelmintic drug usage has led to development of widespread nematode drug resistance [9]. In some areas, sheep farming is already economically challenged [10]. *H. contortus* is a highly pathogenic parasite of small ruminants, posing a significant risk to animal health and productivity worldwide and as such is a major focus of anthelmintic resistance studies [11]–[12].

A full understanding of the metabolism and mechanisms of many anthelmintics is either unknown or yet to be entirely established. Without a better understanding of the genes and proteins involved in the mediation of drug resistance, effective early monitoring of anthelmintic resistance, vital in ensuring efficient drug usage and preventing economic crisis within the livestock market, cannot be achieved. Despite the availability of certain tests (*in vivo* and *in vitro*) designed to detect drug resistance, there still remains drawbacks in terms of their cost, applicability or reproducibility [9].

On a molecular level, resistance may arise through gene mutation and changes in gene expression [6] and protein synthesis. Drug targets, drug efflux, sequestration, protein/substrate pathways, and a variety of complex protein interaction equilibria may thus be affected, altering overall dynamic status of the phenotype-inducing proteome. Comparative proteomic analysis of drug-resistant and -susceptible nematodes may, therefore, reveal protein profiles reflecting drug-related phenotype, with subsequent applications in biomarker and novel drug-target discovery, elucidation of drug action and metabolism as well as resistance monitoring. Proteomics offers sensitive approaches to resolve, quantify and unravel the protein mechanisms that may underpin the development and establishment of drug resistance in parasitic helminths [13].

However, only one proteomic study of *H. contortus* currently exists and this is focused upon the relatively small excretory/secretory sub-proteome [14]. A much more in-depth exploration of the potentially larger group of drug metabolising proteins in the cytosolic and membrane sub-proteomes is urgently needed if anthelmintic resistance (AR) mechanisms are to be understood.

In order to assist, amongst other aims, AR research, the economically significant livestock parasitic nematode *H. contortus* at least is currently the subject of a full-genome sequencing project at the Wellcome Trust Sanger Institute. This work is ongoing and the assembled and annotated protein sequences are not available (http://www.sanger.ac.uk/resources/downloads/helminths/haemonchus-contortus.html).

In the year 2000, a number of Expressed Sequence Tag (EST) sequencing projects were initiated [15] in order to provide researchers with sequence data. These sequences, coming from different nematode species, are grouped in the NEMBASE dataset, which already consisted in 2006 of more than 340,000 sequences available online (http://www.nematode.org), 34% of which were from parasites of veterinary importance [16]. In the particular case of *H. contortus*, these sequences have already been used as a database to assist in the proteomic identification process of its excretory/secretory sub-proteome by Yatsuda *et al.* [14]. During this study, 1,876 available, clustered nucleotide ESTs were translated in the six different Open Reading Frames (ORFs) in order to produce a putative EST protein database, in which all sequences shorter than 60 amino acids were deleted. This method of protein translation, although useful, suffers in terms of the quality of protein sequence output due to the fact that ESTs can be of relatively poor quality, subject to frameshifts and likely to contain sequencing errors. All of these limitations will reduce the size and usefulness of a protein dataset derived from basic six-frame translation with inclusion of false positive and truncated translations due to frameshift error. Nowadays, translation and annotation of ESTs is often performed using specialised algorithms, based on similarity and prediction of ORFs, with the inclusion of frameshift error accounted for [17]. These methods produce more reliable protein sequences which can be used in protein identification studies with greater confidence.

The objectives of this investigation were to: (i) create an annotated two-dimensional electrophoresis (2-DE) gel reference profile of *H. contortus* cytosolic proteins as a prerequisite to initiate proteomic biomarker investigations of resistant and susceptible worm isolates; (ii) overcome the issue of poor sequence representation for proteomic identification by theoretical translation of publicly available EST data (around 6,400 nucleotide sequences) using specialised algorithms to generate a searchable database suitable for use with PMF software; (iii) demonstrate the feasibility of identification of a high percentage of the proteins by MALDI-TOF MS in conjunction with PMF searching of the foregoing database; (iv) verify the effectiveness of MALDI-TOF MS in conjunction with a PMF database searching approach utilising the theoretically translated EST dataset of *H. contortus* using MS/MS ion searches.

In the case of proteins identified by both MALDI-TOF MS and MS/MS, *H. contortus* proteins were subjected to searches of the NCBI non-redundant database using BLAST to infer protein identity and function. An initial set of a hundred abundant 2-DE protein spots were selected for identification by MALDI-TOF MS with PMF database searching. Twenty duplicate spots from this batch were randomly chosen for MS/MS followed by ion searches of databases. Further verification of the proteins identified was provided through *de novo* sequencing of peptides and searching of the resulting sequence to assess concordance with both PMF and MS/MS ions searches. Proteomic studies where confidence in protein identification is difficult to assess by the non-proteomic specialist would benefit from a ‘simple-to-understand’ value or measure of the confidence in identification. For this reason, at all stages in identification of proteins in this study, the use of the ‘universally understood’ measure of statistical significance of protein identification used in the MASCOT search engine [18] was included.

## Methods

### Preparation of *Haemonchus contortus* cytosolic protein extracts

Anthelmintic-susceptible MHco3 *H. contortus* were obtained from Dr Philip Skuce (Moredun Research Institute, Edinburgh, UK). They were stored frozen at −80°C and sampled into 1 mL cubes for the purposes of the experiment.

Samples were thawed, mixed in approximately, equal volumes (v/v/v) with a buffer containing 20 mM KHPO_4_, 50 mM NaCl, 0.1% Triton X-100, a protease inhibitor cocktail (mini-complete, Boehringer-Mannheim, IN, USA) and 1.0 mm Zirconia Beads, in a ‘Mini-Bead Beater’ (Biospec Products) for 1 min followed by 1 min on ice, repeated three times. To assure thorough homogenization, the resulting fraction, separated from the beads by pipetting, was manually crushed in a glass grinder cooled on ice until homogenisation was complete. Samples were centrifuged at 100 000× *g* for 1 h at 4°C. The resulting supernatant, was precipitated overnight at −20°C with an equal volume of 10% trichloroacetic acid-acetone, washed twice with acetone at 4°C, and resuspended in isoelectric focusing buffer (6 M urea, 1.5 M Thiourea, 3% (w/v) 3-[(3-cholamidopropyl) dimethylammonio]-1-propanesulfonate (CHAPS), 66 mM dithiothreitol (DTT), 0.5% v/v ampholytes pH 3–10 (Pharmalytes, Amersham BioSciences, UK.). Protein concentration was estimated using the Biorad Protein Assay Reagent (Cat. No. 500-0006) according to the manufacturer's instructions, based upon the adapted method of Bradford [19].

### Two-dimensional electrophoresis (2-DE)

2-DE was performed according to the method of Görg *et al.* [20]. 250 µg or 500 µg of MHco3 *H. contortus* samples were actively in-gel rehydrated at 50 V for 12 h into 17 cm, pH 3–10 non-linear immobilised pH gradient strips (BioRad). These strips were covered with mineral oil in order to prevent dehydration. They were focused at 250 V for 15 min (linear ramp), 10 000 V for 3 h, 10 000 V to 50 000 Vh (linear ramp) using a Protean IEF Cell (BioRad). Immobilised pH gradient strips were equilibrated in two stages (reducing and alkylating). Reduction was undertaken for 15 min in equilibration buffer [50 mM Tris-HCl pH 8.8, 6 M urea, 30% glycerol, 2% sodium dodecyl sulphate (SDS)] containing 1% (w/v) DTT, followed by 15 min of alkylation in the same equilibration buffer with 2.5% (w/v) iodoacetamide replacing DTT. Strips were then loaded on the top of vertical, 20 cm×20 cm×1 mm, 12.5% polyacrylamide gels and sealed with dyed agarose. Polyacrylamide gels were run at 15 mA/gel until the dye front reached the resolving gel, then at 30 mA/gel until the end of dye migration. Electrophoresis was performed according to the discontinuous (Laemmli) system in Tris/Glycine/SDS buffer (25 mM Tris, 192 mM glycine, 0.1% (w/v) SDS), using the Protean II xi 2-D Cell (BioRad) system.

2-DE gels were stained with colloidal Coomassie Blue and scanned using a GS170 Calibrated Imaging Densitometer and PDQuest software (BioRad). The analysis of the gel images was performed with Progenesis Software PG200 version 2006. Spot detection was manual, background subtraction was performed using the ‘average on boundaries’ method and normalisation was performed using the total spot volume multiplied by total area.

### Protein identification

2-DE protein spots from both 250 µg and 500 µg protein gels were excised and tryptically digested in modified trypsin (sequencing grade, Roche, UK) as previously described by Chemale *et al.* [21]. Gel plugs were destained by washings in 50% v/v acetonitrile, 50% v/v 50 mM ammonium bicarbonate at 37°C. Cleared gel plugs were dehydrated in a centrifugal vacuum concentrator at 60°C. Following addition of 10 µL of trypsin solution (20 ng/µL of trypsin in 50 mM ammonium bicarbonate), digestion was undertaken for 16 h at 37°C.

Peptides were eluted according to the method of Shevchenko *et al.* [22]. In this, supernatant of successive 20 min washes with 20 µL of 50 mM ammonium bicarbonate∶acetonitrile 1∶1 (v/v; once), then with 20 µL of 5% formic acid∶acetonitrile 1∶1 (v/v; twice), were recovered and dried by centrifugal evaporation at 60°C. Samples were resuspended in 10 µL 0.1% (v/v) formic acid for Quadrupole Time-of-Flight (Q-TOF) MS/MS or resuspended in 5 µL 0.1% (v/v) trifluoroacetic acid for MALDI-TOF MS.

For MALDI-TOF MS, a 3 µL first layer of saturated α-cyano-4-hydroxycinnamic acid in methanol was dropped onto the metal target plate as described by Vorm *et al.* [23] in order to increase the homogeneity of the matrix and analysed peptides. 6 µL of each sample mixed with an equal volume of saturated matrix were dropped onto the metal target plate in three step to allow it to air dry correctly.

Samples were analysed using a M@LDI mass spectrometer (Waters, Manchester, UK). Resulting spectra were analysed directly using Masslynx version 4.0 MS software (Waters). For each sample, spectra were combined and peak lists were generated using the ProteinLynx tool with the following settings: background subtraction (polynomial order 3, 10 or 20, between 90 and 40% below curve, tolerance 0.01), smoothing using the Savitzky Golay method twice and centring using the centroïd top method at 80%. When automatic peak collection was problematic due to background noise, the peak list was collected manually.

For Q-TOF MS/MS, samples were analysed using an UltiMate nano-liquid chromatograph (LC Packings) connected to a Q-TOF Micro electrospray tandem mass spectrometer (Waters). Chromatography was carried out on a μ-Precolumn C18 cartridge (LC Packings) connected to a PepMap C18 column (3 µm 100 Å packing; 15 cm×75 µm internal diameter), using a linear gradient of 5% v/v solvent B (0.1% v/v formic acid in 80% v/v acetonitrile in water) in solvent A (0.1% v/v formic acid in 2% v/v acetonitrile in water) to 100% solvent B over 60 min at a flow rate of 200 nL/min. The spectrometer was operated in Data Directed Analysis mode, where a survey scan was acquired over mass-to-charge ratio (m/z) 400–1500, with switching to MS/MS on multiply charged ions. Resulting spectra were processed as previously with the settings: background subtraction (polynomial order 1, below curve 40%, tolerance 0.01), smoothing using Savitzky Golay method once and centring using the centroïd top method at 80%. Raw MS data (Accession numbers: 21573–21592) have been deposited in the PRoteomics IDEntification database, PRIDE (www.ebi.ac.uk/pride/) [24].

### *H. contortus* putative protein database production from ESTs and bioinformatic analysis of mass spectrometry data

Clustered EST sequences from *H. contortus* were downloaded from the University of Edinburgh website (http://www.nematodes.org) and theoretically translated to a putative protein dataset via a Linux-based pipeline prot4EST [17]. 6,387 nucleotide EST sequences (918,038 residues) were processed through the pipeline. The resulting database file, containing each sequence translated in the appropriate ORF, was then added to the databases of a locally installed (The University of Liverpool) MASCOT search engine (Matrix Science; http://www.maxtrixscience.com; [18]).

For each MALDI-TOF spectrum, a MASCOT PMF search of the National Center for Biotechnology Information (NCBI) nr protein database was first undertaken (NCBI nr release 20091112, containing 10 032 801 sequences, 3 422 028 181 residues; http://www.ncbi.nlm.nih.gov/). Searches were performed allowing up to 1 trypsin miscleavage and without restriction on protein mass or isoelectric point (p*I*). Variable modifications included carbamidomethylation of cysteine, modifications of glutamic acid into pyro-glutamic acid and oxidation of methionine. Peptide mass tolerance was set to ±1.2 Da. A MASCOT PMF search of all data was made against the *H. contortus* translated protein dataset. Significance was established according to expectancy (e) value transformed into a MOWSE score (with significance at p-value<0.05 at scores over 51), percentage coverage and theoretical p*I* and molecular weight (Mw) compared to the approximate experimental values observed on 2-DE gels.

For each sample analysed by Q-TOF MS/MS, peptide spectra were collected as Sequest-compatible .dta files using MassLynx software version 4.0 and assembled into merged peak list files using the ProteomeCommons Peak list Conversion Utility (http://www.proteomecommons.org; [25]). These merged files were used to search the *H. contortus* dataset using the local MASCOT MS/MS Ion Search engine. The parameters previously described for MALDI-TOF analysis were set in a similar way. Peptide mass tolerance was set again to ±1.2 Da and MS/MS fragment ion tolerance set to ±0.6 Da. The identified *H. contortus* sequences were then submitted to the NCBI nr protein database (previously described) using Protein BLAST (http://www.ncbi.nlm.nih.gov/blast; [26]) in order to search for orthologous sequences and infer protein identity and function.

In order to confirm our previous annotation results, Q-TOF MS/MS data from each protein spot were also analysed using MassLynx Peptide Sequencing software Pepseq version 3.3, with manual intervention, in order to produce limited peptide sequences. Peptide spectra selected for sequencing were combined, smoothed using the Savitzky Golay method once and centred using the centroïd top method at 80%. Sequencing was then performed manually to produce at least two unique peptides for each protein spot. Resulting sequences were then searched against the NCBI nr protein database (previously described) using the FASTS algorithm (fasta.bioch.virginia.edu/fasta_www2; [27]) to provide a statistical measure of identification confidence, infer protein identity and function, in addition to assessing similarity to PMF and MS/MS ion search identifications.

## Results

### Two-dimensional electrophoresis reference map profile of *Haemonchus contortus* cytosolic proteins

**Figure 1:**
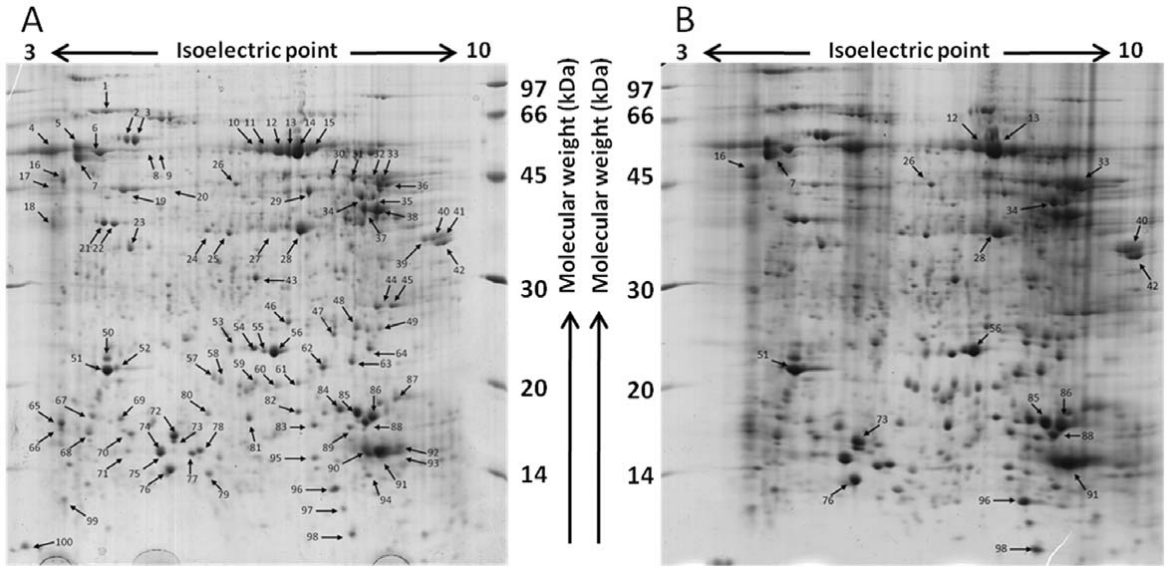
Colloidal Coomassie blue-stained 2-DE gel images of *H. contortus* cytosolic proteome. 250 µg (A) or 500 µg (B) *H. contortus* cytosolic proteins were fractionated on pH 3–10 non-linear immobilised pH gradient strips and then run on a 12.5% polyacrylamide gel for the second dimension. (A) 100 spots showing the highest normalised volumes (area×intensity) were analysed using MALDI-TOF MS with PMF searching of databases. (B) 20 spots, matched to the previous gel image (A), were analysed using Q-TOF MS/MS with Ion Searches of the putative EST *H. contortus* protein database and the identified sequences used for BLASTp searches of the NCBI protein database.

2-DE gels loaded with two different quantities of protein were produced to resolve and display the high and low abundance cytosolic proteins of *H. contortus* (Figure 1). Analyses by Progenesis PG200 v2006 software (Non-Linear Dynamics) allowed all the spots derived from the differently loaded gels (two gels at each loading level) to be matched. 602 spots were common to all differently loaded gels. 100 abundant spots were selected according to their normalised volumes, and were processed for further identification by PMF using MALDI-TOF MS (Figure 1).

### Identifications of cytosolic proteins from *Haemonchus contortus*

The MALDI-TOF mass spectra resulting from the 100 tryptically digested spots were processed using ProteinLynx software (within the Waters Masslynx software). The m/z peak lists were automatically produced, using ProteinLynx software except in the cases of spots 10, 24, 28 and 92 where the parameter settings failed to produce a peak list (peak lists were collected manually in these cases). The lists were then entered into MASCOT PMF searches of the complete NCBI nr protein database using the MASCOT search engine. Only three spots were identified using this method. Spots 12, 22 and 45 were identified, with a statistically significant score (respectively 120, 91 and 96; significant score is 82, p value<0.05, which is relative to the size of the database) and matched to glutamate dehydrogenase (Accession number: ACT34056.1), disorganised muscle protein 1 (Accession number: ADZ24723.1) and Triosephosphate isomerase (Accession number: ADR66027.1) from *H. contortus*.

Local MASCOT PMF searches of the putative EST protein database were then carried out with the MALDI MS spectral data from the 100 protein spots (Table 1). The detailed outputs for all the MASCOT PMF searches of the MALDI MS data are given in Figure S1. Protein BLAST searches of the most significantly scoring hits against the NCBI nr protein database was undertaken to provide scored matches of *H. contortus* proteins or orthologous proteins in other organisms from which putative function and homology could be inferred (Table 1).

**Table 1:** Successful protein spot identification by MALDI-TOF MS in conjunction with PMF and/or Q-TOF MS/MS with BLAST database searching.

MASCOT PMF searches (or Ion Searches in brackets) of putative EST protein database			BLASTp search against entire NCBI nr protein database				
Protein spot	Mascot MOWSE Score	EST Accession Number	BLAST score	Accession Number	Protein Identified	Species	Identification Method
2	57	03377 1	697	XP_001664603	CBG11701	*C. briggsae AF16*	MALDI-TOF/PMF^1^
4	75	00006 1	1077	ACT34056	Putative Glutamate deHydrogenase	*H. contortus*	MALDI-TOF/PMF
6	54	00273 1	420	ABC86956	Protein Disulfide Isomerase	*T. circumcincta*	MALDI-TOF/PMF
7	35 (77)	00592 1	540	CAL30086	Calreticulin precursor	*H. polygyrus*	“MALDI-TOF PMF similarity”+MS/MS/BLAST^2^
8	66	00006 1	1077	ACT34056	Putative Glutamate dehydrogenase	*H. contortus*	MALDI-TOF/PMF
9	81	00006 1	1077	ACT34056	Putative Glutamate deHydrogenase	*H. contortus*	MALDI-TOF/PMF
10	59	08936 1	248	NP_506413	F53C11.3	*C. elegans*	MALDI-TOF/PMF
11	92	00006 1	1077	ACT34056	Putative Glutamate deHydrogenase	*H. contortus*	MALDI-TOF/PMF
12	105 (659)	00006 1	1077	ACT34056	Putative Glutamate deHydrogenase	*H. contortus*	MALDI-TOF/PMF+MS/MS/BLAST^3^
13	65 (707)	00006 1	1077	ACT34056	Putative Glutamate deHydrogenase	*H. contortus*	MALDI-TOF/PMF+MS/MS/BLAST
14	58	00006 1	1077	ACT34056	Putative Glutamate deHydrogenase	*H. contortus*	MALDI-TOF/PMF
15	74	00006 1	1077	ACT34056	Putative Glutamate deHydrogenase	*H. contortus*	MALDI-TOF/PMF
16	(342)	00296 2	388	NP_001024806	Calumenin	*C. elegans*	MS/MS/BLAST^4^
17	54	00006 1	1077	ACT34056	Putative Glutamate deHydrogenase	*H. contortus*	MALDI-TOF/PMF
18	58	06327 1	298	XP_001897798	Ubiquitin conjugating enzyme E2 H	*B. malayi*	MALDI-TOF/PMF
19	55	00199 6	683	ABX82966	Actin variant 1	*D. vivparus*	MALDI-TOF/PMF
20	34	00199 8	515	ABX82966	Actin variant 1	*D. vivparus*	“MALDI-TOF PMF similarity”^5^
21	55	00195 1	398	XP_001899521	Disorganized muscle protein 1	*B. malayi*	MALDI-TOF/PMF
22	58	00195 1	398	XP_001899521	Disorganized muscle protein 1	*B. malayi*	MALDI-TOF/PMF
24	43	01204 1	351	NP_001023074	Inorganic Pyrophosphatase	*C. elegans*	“MALDI-TOF PMF similarity”
25	55	01204 1	351	NP_001023074	Inorganic Pyrophosphatase	*C. elegans*	MALDI-TOF/PMF
26	(52)	00280 1	344	XP_001679131	CBG03214	*C. briggsae AF16*	MS/MS/BLAST
27	56	00006 1	1077	ACT34056	Putative Glutamate deHydrogenase	*H. contortus*	MALDI-TOF/PMF
28	54 (404)	00006 1	1077	ACT34056	Putative Glutamate deHydrogenase	*H. contortus*	MALDI-TOF/PMF+MS/MS/BLAST
29	53	00006 1	1077	ACT34056	Putative Glutamate deHydrogenase	*H. contortus*	MALDI-TOF/PMF
30	39	00280 1	344	XP_001679131	CBG03214	*C. briggsae AF16*	“MALDI-TOF PMF similarity”
32	39	00537 1	317	NP_491955	K02F2.2	*C. elegans*	“MALDI-TOF PMF similarity”
33	(238)	00183 1	446	XP_001666207	CBG09180	*C. briggsae AF16*	MS/MS/BLAST
34	56 (56)	00183 1	446	XP_001666207	CBG09180	*C. briggsae AF16*	MALDI-TOF/PMF+MS/MS/BLAST
35	58	00183 1	446	XP_001666207	CBG09180	*C. briggsae AF16*	MALDI-TOF/PMF
37	39	01607 2	605	NP_001021240	F01F1.12	*C. elegans*	“MALDI-TOF PMF similarity”
40	60 (232)	11007 1	336	XP_001666501	CBG15213	*C. briggsae AF16*	MALDI-TOF/PMF+MS/MS/BLAST
41	73	11007 1	336	XP_001666501	CBG15213	*C. briggsae AF16*	MALDI-TOF/PMF
42	37 (333)	11007 1	336	XP_001666501	CBG15213	*C. briggsae AF16*	“MALDI-TOF PMF similarity”+MS/MS/BLAST
43	60	00006 1	1077	ACT34056	Putative Glutamate deHydrogenase	*H. contortus*	MALDI-TOF/PMF
44	77	06393 1	400	XP_001680248	CBG21017	*C. briggsae AF16*	MALDI-TOF/PMF
45	79	06393 1	400	XP_001680248	CBG21017	*C. briggsae AF16*	MALDI-TOF/PMF
46	82	02208 1	252	1TW9A	Glutathione Transferase-2, Apo Form	*H. polygyrus*	MALDI-TOF/PMF
47	42	00515 1	171	XP_001671373	CBG17729	*C. briggsae AF16*	“MALDI-TOF PMF similarity”
51	(2727)	00182 4	362	CAJ09947	NIM-1 protein	*H. contortus*	MS/MS/BLAST
53	55	01409 1	249	NP_499900	K02D7.1	*C. elegans*	MALDI-TOF/PMF
54	63	07180 1	226	XP_001669439	CBG19736	*C. briggsae AF16*	MALDI-TOF/PMF
56	(1839)	00006 1	1077	ACT34056	Putative Glutamate deHydrogenase	*H. contortus*	MS/MS/BLAST
57	38	06327 1	298	XP_001897798	Ubiquitin conjugating enzyme E2 H	*B. malayi*	“MALDI-TOF PMF similarity”
59	59	06327 1	298	XP_001897798	Ubiquitin conjugating enzyme E2 H	*B. malayi*	MALDI-TOF/PMF
60	45	00814 1	328	AAN05752	heat shock protein 20	*H. contortus*	“MALDI-TOF PMF similarity”
61	60	02260 2	267	CAG25499	heat shock protein 20	*O. ostertagi*	MALDI-TOF/PMF
62	53	03240 3	297	NP_001023903	F40A3.3	*C. elegans*	MALDI-TOF/PMF
63	51	03240 1	297	NP_001023903	F40A3.3	*C. elegans*	MALDI-TOF/PMF
70	63	02240 2	167	NP_001033512	Lipid Binding Protein	*C. elegans*	MALDI-TOF/PMF
72	66	02740 1	167	NP_001033512	Lipid Binding Protein	*C. elegans*	MALDI-TOF/PMF
73	81 (145)	02740 1	167	NP_001033512	Lipid Binding Protein	*C. elegans*	MALDI-TOF/PMF+MS/MS/BLAST
76	(158)	00822 1	303	XP 001675459	CBG18577	*C. briggsae AF16*	MS/MS/BLAST
77	79	07574 1	69,3	NP_495503	E04F6.9	*C. elegans*	MALDI-TOF/PMF
78	35	00413 1	170	NP_001123180	T08A9.11	*C. elegans*	“MALDI-TOF PMF similarity”
80	47	11248 1	154	AAN05752	heat shock protein 20	*H. contortus*	“MALDI-TOF PMF similarity”
81	61	00942 2	165	AAN05752	heat shock protein 20	*H. contortus*	MALDI-TOF/PMF
82	46	00047 3	333	Q27666	Superoxide dismutase	*H. contortus*	“MALDI-TOF PMF similarity”
85	97 (1595)	00208 1	197	P27613	Globin-like host protective antigen	*T. colubriformis*	MALDI-TOF/PMF+MS/MS/BLAST
86	37 (384)	02230 2	260	ABJ97284	major sperm protein	*D. viviparus*	“MALDI-TOF PMF similarity”+MS/MS/BLAST
88	56 (67)	00202 5	216	P27613	Globin-like host protective antigen	*T. colubriformis*	MALDI-TOF/PMF+MS/MS/BLAST
89	106	01375 1	205	NP_508557	Lipid Binding Protein	*C. elegans*	MALDI-TOF/PMF
90	66	00907 1	64	NP_001024064	R02C2.7	*C. elegans*	MALDI-TOF/PMF
91	72 (196)	00372 1	210	CAP20913	CBG24261	*C. briggsae*	MALDI-TOF/PMF+MS/MS/BLAST
92	52	00372 1	210	CAP20913	CBG24261	*C. briggsae*	MALDI-TOF/PMF
93	40	03264 2	157	CAP20913	CBG24261	*C. briggsae*	“MALDI-TOF PMF similarity”
94	41	04833 1	188	2OS5_A	Macrophage Migration Inhibitory Factor	*A. ceylanicum*	“MALDI-TOF PMF similarity”
96	44 (114)	00229 1	159	XP_001664602	CBG11702	*C. briggsae AF16*	“MALDI-TOF PMF similarity”+MS/MS/BLAST
98	(122)	01027 1	290	XP 0024231426	ubiquitin, putative	*P. humanus corporis*	MS/MS/BLAST

1 Statistically significant protein spot identification after MALDI-TOF MS analysis followed by PMF search of the *H. contortus* putative EST protein database.

2 Non-statistically significant protein spot identification after MALDI-TOF MS analysis followed by PMF search of the *H. contortus* putative EST protein database, but verified by statistically significant protein spot identification after Q-TOF MS/MS analysis.

3 Statistically significant protein spot identification after MALDI-TOF MS analysis followed by PMF search of the *H. contortus* putative EST protein database. These results were confirmed by Q-TOF MS/MS analysis.

4 Statistically significant protein spot identification after Q-TOF MS/MS followed by BLASTp search of the *H. contortus* putative EST protein database.

5 Non-statistically significant protein spot identification after MALDI-TOF MS analysis followed by PMF search of the *H. contortus* putative EST protein database. However, the observed Mw of the spots, calculated on the gel image, were correlated with the theoretical Mw of the intact protein in the best BLASTp match. These data are detailed in Tables S3 & S4.

Each protein spot was excised from the 250 µg protein-loaded gel and analysed by MALDI-TOF MS. A local MASCOT PMF search of the *H. contortus* putative EST protein database was performed and the highest scoring EST sequence match, along with its MOWSE-based score (significance threshold score >51, p-value<0.05) is reported. 20 spots were also selected for Q-TOF MS/MS, excised from the 500 µg protein-loaded gel and analysed by Q-TOF MS/MS. A local MASCOT Ion Search of the *H. contortus* putative EST protein database was performed and the MOWSE-based score of the highest scoring EST sequence match (significance threshold score >25, p-value<0.05) are given in brackets. Following either method, each EST sequence hit was submitted to a BLASTp search against the entire NCBI nr protein database. For each search, the highest scoring hit score (significance threshold >44, p-value<0.01), its accession number and protein name are reported. For information, the species corresponding to the highest scoring hit and the method of identification used are also given.

39 spots out of 100 (Table S1) were significantly matched to an entry in the *H. contortus* EST dataset according to the significance score threshold for MASCOT searches (score >51, p-value<0.05). 23 spots among the non-significant identifications were matched by Protein BLAST to proteins having similar computed p*I* and Mw to the approximate experimental values (Table S3), leaving 38 spots without such correlation (data not shown). These 62 sequences were then matched by Protein BLAST to an entry from the NCBI nr database with a significant score (score >45, p-value<0.01; Tables S2 & S4).

Spots 28 and 56 were both matched to a putative glutamate dehydrogenase (PGDH) of 60 kDa from the *H. contortus* putative EST protein database. Although this result is inconsistent with both observed molecular weights (approximately 41 and 22 kDa, respectively), spot 28 was identified with a statistically significant score but spot 56 was not. Their analyses by MS/MS confirmed the PMF results (Tables S5 & S6).

### Peptide sequencing

To confirm the validity of the PMF approach, a selection of 20 spots from the 500 µg protein-loaded gel (Figure 1) were also analysed by Q-TOF MS/MS. These 20 protein spots were matched to 20 spots on the 250 µg gel and corresponded to spots belonging to either significant or non-significant identifications, or unidentified protein spots from the previous MALDI-TOF MS analyses. Spectra were analysed using ProteinLynx and Sequest-compatible (.dta) output files were collected and merged before being subjected to a MASCOT MS/MS ion search of the putative EST protein database (Table S5). All the 20 proteins were statistically identified (score >25, p-value<0.05) and subsequently annotated using Protein BLAST (score >45, p-value under 0.01; Table S6).

**Figure 2:**
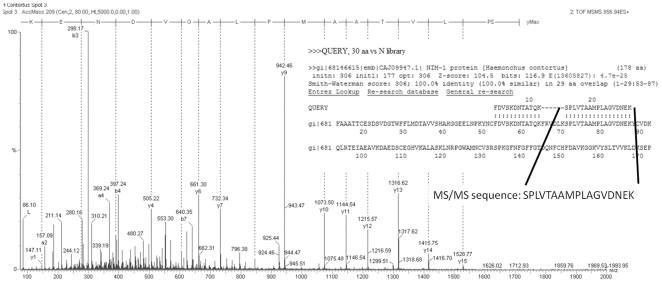
MS/MS spectrum of peptide SPLVTAAMPLAGVDNEK from spot 51. Example of MS/MS spectrum *de novo* limited peptide sequencing from spot 51 and its inclusion into a FASTS search against the NCBI nr protein database (example of FASTS output format is inset).

This mini-selection of 20 spots was also subjected to manual *de novo* peptide sequencing in order to confirm the validity of the PMF approaches. Peptide spectra were analysed using Pepseq software (see example in Figure 2) and resulting sequences were searched against the NCBI nr protein database using the FASTS algorithm (Table S7 and Figure S2). With the exception of spot 34, all protein spots were statistically matched (score >44, p-value under 0.01) to an entry in NCBI nr protein that correlates closely to the other approaches for identification. The effectiveness of MALDI-TOF and MS/MS proteomic techniques in conjunction with searching of the NCBI nr or *H. contortus* putative EST protein databases for protein identification are compared in Table 2.

**Table 2:** Comparison of effectiveness of two proteomic techniques in conjunction with searching of different databases for protein identification.

Mass Spectrometry	Bioinformatic analysis	Database	No of spots analysed	Successful Identification
MALDI-TOF	PMF	NCBI nr^1^	100	3
MALDI-TOF	PMF	EST Hc^2^	100	39 Statistically Significant+(23 non-statistically significant)^3^
nano-LC Q-TOF MS/MS	MS/MS Ion search	EST Hc^2^	20	20
nano-LC Q-TOF MS/MS	*de novo* sequencing	NCBI nr^1^	20	19

1 NCBI non-redundant database.

2 *H. contortus* putative EST protein database.

3 Consist of entries in Table 1 identified by MALDI-TOF MS.

## Discussion

The main issue for proteomic studies on parasitic nematodes remains the lack of availability of genomics data to allow searching of MS analysis data. In order to possess sequences to search with proteomics results, the perfect solution is the full sequencing, assembly and annotation of the genome of the organism in question. Being an expensive task, most parasitic nematodes have not been the subject of such studies, but a number of EST sequencing projects have been launched and assembled to provide researchers with some transcriptomic sequence data. However, the recent advent of high-throughput second-generation sequencing technologies in conjunction with more bioinformatic tools has dramatically reduced sequencing costs and will in the near future provide much greater availability of partial sequence datasets to many more research groups.

In the case of *H. contortus*, a full genome sequencing project (*H. contortus* Sequencing Project; http://www.sanger.ac.uk/Projects/H_contortus/) has been completed and supercontigs were assembled recently (26/08/2009), but completion of the assembly and gene annotations remain in progress. This solution would be ideal for protein studies, as the full genome sequencing covers all potential proteins present in the organism. However, the lack of assembly and annotation of the genes renders the current output limited in supporting urgently required protein level studies on anthelmintic resistance and vaccine discovery.

By definition, ESTs are short, one-shot sequences with no overlapping sequencing and contain errors. This resource is of course not as reliable as costly full genome sequencing. However, many research groups are able to accommodate the costs involved in generating their own EST datasets for their organism of interest. Inherent errors in EST datasets make crude 6-frame translations much less useful for protein datasets. However, an advantage of using more sophisticated methods of translation, such as prot4EST software, in translating ESTs to yield putative protein sequences, is that the algorithm attempts to avoid problems resulting from inaccuracy in the nucleotide EST sequences. Furthermore, where no matches are found for the nucleotide sequence in available public databases, prot4EST uses the ‘ESTScan’ algorithm to perform a prediction considering the statistical Hidden Markov Model [28].

In our study, 6,387 EST nucleotide sequences, available at the time of our study from the NEMBASE project, were processed by prot4EST using default settings and searching the NCBI nr proteins as a reference dataset. The resulting translation suggests that a vast majority of the nucleotide sequences were of good quality. 5,332 sequences (83% of the total) were either found to have a good match in the NCBI nr protein database or were found to have a predicted correct ORF by ‘ESTScan’ algorithm. Out of these 5,332 sequences, 3,087 (58%) were found to have significant identity and inferred homology to a nuclear or mitochondrial gene entry in the NCBI nr protein database. These results are a little lower than the proportion of *H. contortus* ESTs found to have significant identity and inferred homology to *C. elegans* proteins within the NCBI nr protein database (around 70%) performed by Parkinson *et al.* [29].

EST sequences have been utilised in nematode proteome studies to help protein identifications or infer functions of transcript sequences. Hewitson *et al.* and Robinson *et al.* used available EST databases to assist MALDI-TOF MS and electrospray MS/MS identifications of secreted proteomes of *Brugia malayi*, *Trichinella spiralis* and *Trichinella pseudospiralis* [30]–[31]. Smith *et al.* used the newly created NEMBASE putative EST protein database (which was also built with prot4EST) to help identify proteins of the secretome of *Teladorsagia circumcincta* [32]. Recently, Chemale *et al.* used available translated *Fasciola hepatica* EST sequences from the Sanger Institute to support their proteomic identifications of soluble *F. hepatica* proteins [33]. The annotation of the EST sequences identified in this study was performed using Protein BLAST searches against the NCBI nr protein database.

Optimisation of 2-DE profiling of *H. contortus*, a parasitic nematode of global welfare and economic significance to the livestock industry, is a pre-requisite to further proteomic biomarker investigations, novel drug-target discovery, elucidation of drug action and resistance monitoring of drug-susceptible and -resistant worm isolates. Therefore, 2-DE reference maps of cytosolic proteins were produced and gel images were processed in order to select 100 highly expressed proteins to be subject to MALDI-TOF MS analysis (Figure 1).

From these 100 major spots, 62 protein identifications by MALDI-TOF MS were obtained using the species-specific EST database (Table 1). The remaining proteins could have been identified readily by MS/MS, but our focus was on demonstrating the validity of MALDI-TOF-MS in conjunction with an EST database. This approach would represent a significant cost saving to laboratories and importantly allow the focus of identification to be upon a dataset compiled of the very parasite species sequences under investigation with less reliance on cross-species public datasets of related organisms and the subsequent problems in confidence arising from inferred relationship and identification. Furthermore, the inclusion of the MASCOT scoring system that assigns measures of significance at 5%, 1% or 0.1% for example, to given scores, provides the opportunity for the non-proteomics specialist to gain a measure of confidence in the protein identification. It must be appreciated however, that this measure offers only a guide and in the case of incomplete datasets, potential errors within EST sets, and truncated sequences for example, caution must always be employed to avoid including or excluding identifications based purely upon significance scores alone. Further information (e.g. molecular weight, pI) may be available to support ‘weak’ identifications.

Slight differences in the calculated and observed Mw and p*I* for some proteins can be observed in this profile, but they are generally within limits of known differences, such as due to post-translational modifications. In the case of partial EST protein sequences, the NCBI nr protein sequence database could give an approximate full length Mw and p*I*, as in the case of spots 20, 24, 30 and 32. Following this approach, 23 protein spots were considered as conclusive and robust identifications even when the initial MASCOT score was not significant (see Tables 1 and 2).

In order to verify this new approach to protein identification, Q-TOF MS/MS was performed on 20 randomly selected spots, including both statistically (9 spots) and non-statistically (3 spots) identified spots (Table 1) . Q-TOF MS/MS analysis of the statistically identified spots 12, 13, 28, 40, 73, 85, 86, 88 and 91 supported the validity of our technique by confirming the previous identifications. Tentative conclusions concerning the non-statistically identified spots (7, 42 and 96) were also validated, as all three spots were, this time, statistically matched with a similar *H. contortus* EST sequence. Q-TOF MS/MS was also performed on seven spots without inferred identifications previously (16, 26, 33, 51, 56, 76 and 98). For these spots, only Q-TOF MS/MS, followed by MASCOT Ion Searches of the putative EST protein database was able to produce statistically significant identifications in all cases. It is also to be noted that limited *de novo* sequencing of peptides derived from the Q-TOF MS/MS analysis and BLASTp searching of the NCBI nr protein database also provided the same results as for MS/MS Ion Searches against the *H. contortus* putative EST protein database (see Table 1). This result confirmed that the significant expense and time-consuming effort involved in MS/MS limited *de novo* sequencing of protein spots in the case of non-sequenced genome organisms could at least in part be replaced or reduced by the use of more easily accessible MALDI-TOF technology and the appropriate use of bioinformatic tools and available resources. In the case of failure of MALDI-TOF in conjunction with PMF searching of a species-specific database, the use of MS/MS Ion Searches against a species-specific EST database, which requires less labour intensive work than *de novo* sequencing, has also been shown as a suitable alternative.

The rate of protein identification was considerably increased by using this new EST library produced using ‘prot4EST’ software (3% of identifications using NCBI nr proteins, while 62% were identified using the *H. contortus* putative EST protein database). Our results with the cytosolic proteome of *H. contortus* can be placed into perspective by comparison to the earlier work by Yatsuda *et al.* [14] on the *H. contortus* excretory/secretory proteome (ESP). Using MALDI-TOF MS supported by MASCOT PMF searches against the GenBank nr database and 1,876 *H. contortus* EST sequences available at the time, Yatsuda *et al.* [14] identified 55 proteins from 130 2D-PAGE spots (42% success), 43 of which were statistically significant identifications (33% success), but an additional 12 were considered identified by “fingerprint similarity”. The use of EMOWSE scores assigned to protein identifications within the *H. contortus* database in the Yatsuda *et al.* [14] study may be relatively more difficult to understand by the non-specialist, as scores do not easily translate to universally accepted parameters of significance such as those presented by MASCOT [18]. Whilst the Yatsuda *et al.* [14] study made good use of supporting information to qualify and support protein identification, it was necessary to use relatively expensive MS/MS sequencing followed by Protein BLAST searches against the GenBank nr database, combined with the available *H. contortus* EST sequences, to achieve a high level of protein profiling (107 proteins identified out of a selection of 130).

**Figure 3:**
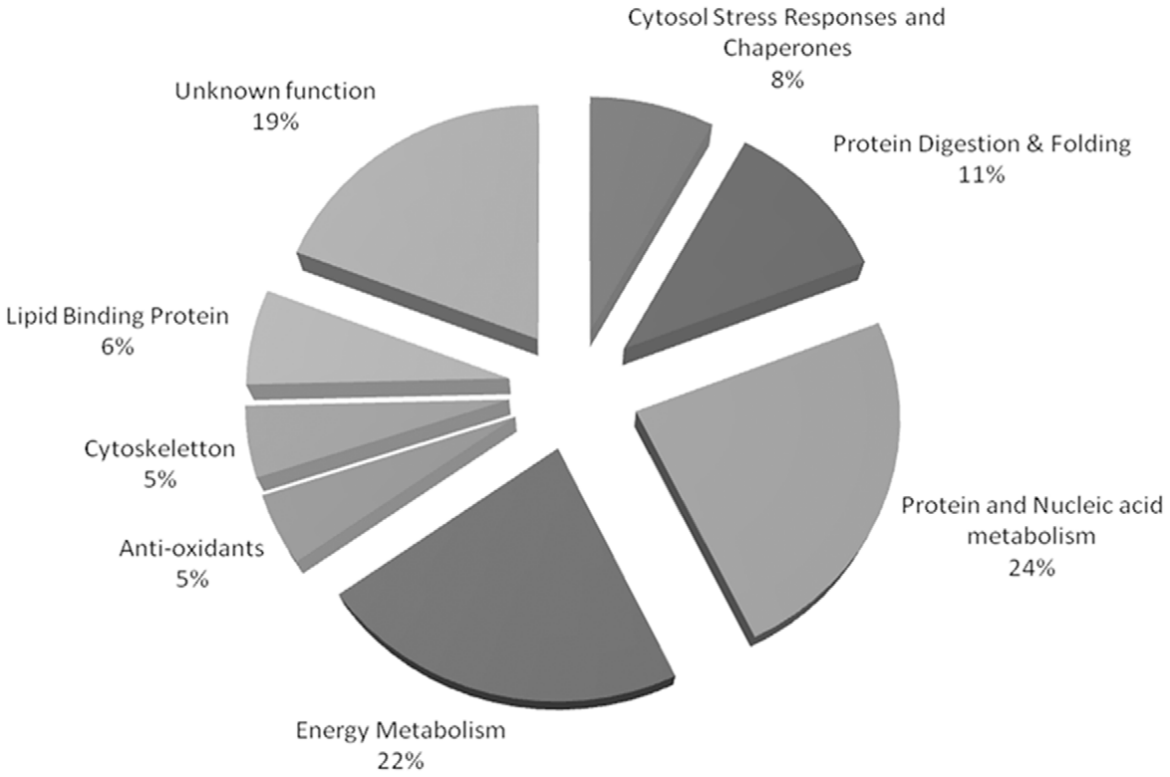
Schematic representation of functional distribution of 62 identified cytosolic proteins from *H. contortus*.

Our proteome profile of the cytosol of *H. contortus* also provided evidence of the presence of an interesting protein complement for future discovery biology (Figure 3). The major components of the cytosolic proteome are, as expected, involved in physiological functions of the cytosol, such as metabolism (over 50% of the identified proteins), and maintenance of cytoskeleton structure (actin, major sperm protein). The cytosol proteome also contained a significant number of stress response elements [such as Heat Shock Protein 20 (spots 60, 61, 80 and 81)], anti-oxidant agents [like the superoxide dismutase (spot 82)] or lipid-binding proteins (spots 70, 72, 73 and 79), all of which can be the subject of up- or down-regulation in the case of drug stress in the organism. Protein spot 46 was matched to a glutathione transferase from *H. polygyrus bakeri* [34].The glutathione transferase enzyme family is known to be involved in xenobiotic detoxification [35] and is already believed to play a role in ivermectin resistance in *C. elegans* [36].

The over-representation of PGDH is a major feature among the 100 highly expressed proteins (14 spots). This enzyme, also reported in a whole organism study by Yan *et al.* [37] and in excretory/secretory products of *H. contortus* from Yatsuda *et al.* [14], is a mitochondrial enzyme involved in the oxidative deamination of glutamate using nicotinamide coenzymes. Skuce *et al.* [38] localised PGDH in sections of *H. contortus* by immunostaining and showed that it was intensely represented in cytoplasm of gut cells. Analysis of sequences of PGDH also confirmed the lack of mitochondrial signal peptide, which is found in other organisms, but not in the nematode *C. elegans*. PGDH was also proven to be only expressed in L4 and adult stages of the *H. contortus* life cycle which is consistent with our study [38].

It can be concluded that the strategy to assist MS protein identification by developing a species-specific putative EST protein database bioinformatics resource in this study, provides a realistic, economic means of correctly identifying 2-DE protein spots from a non-genome sequenced parasite. Furthermore, with increasing availability of cost-effective commercial MALDI-TOF MS services, this approach immediately opens up proteomic opportunities to researchers wishing to utilise existing EST datasets to investigate non-genome sequenced organisms.

## Supporting Information

Figure S1 **(S1.01–S1.64) Protein spot identification by MALDI-TOF MS in conjunction with PMF database searching.** Results output using MASCOT searches for each PMF search for the 2D gel spots summarised in Table 1 and Tables S1, S2, S3 and S4 are given.
(PDF)

Figure S2 **(S2.01–S2.47)** ***De novo*** **limited peptide sequencing from MS/MS spectra for each 2D gel spot, together with the output of FASTS searches of the sequences against the NCBI nr protein database.** The results of these searches for each spot number is summarised in Tables S5, S6 and S7.
(PDF)

Table S1 **Statistically significant MALDI-TOF MS protein spot identifications by PMF searching of the** ***H. contortus*** **putative EST protein database.** Each protein spot was excised from the 250 µg protein-loaded gel and analysed by MALDI-TOF MS. A local MASCOT PMF search of the *H. contortus* putative EST protein database was performed and the highest scoring EST sequence match, along with its MOWSE-based score (significance threshold score >51, p-value<0.05), sequence coverage and the number of matched peptides is reported. For each search, the highest scoring hit EST sequence accession number and its theoretical Mw/p*I* are also detailed.
(DOC)

Table S2 **Annotation of the statistically significant EST sequence hits using BLASTp searches against the entire NCBI nr protein database.** Each EST sequence hit was submitted to a BLASTp search against the entire NCBI nr protein database. For each search, the highest scoring hit score (significance threshold >44, p-value<0.01), its accession number and protein name are reported. For information, the species corresponding to the highest scoring hit, its molecular function according to the NCBI nr protein database and Wormbase when available^1^ and the theoretical Mw/p*I* of the full length sequence are also described.
(DOC)

Table S3 **Non-statistically significant MALDI-TOF MS protein spot identifications using the** ***H. contortus*** **putative EST protein database.** Each protein spot was excised from the 250 µg protein-loaded gel and analysed by MALDI-TOF MS. A local MASCOT PMF search of the *H. contortus* putative EST protein database was performed and the highest scoring EST sequence match along with its MOWSE-based score (significance threshold score >51, p-value<0.05), sequence coverage and the number of matched peptides is reported. For each search, the highest scoring hit EST sequence accession number and its theoretical Mw/pI are also detailed. The observed Mw of the spots, calculated on the gel image, were correlated with the theoretical Mw of the intact protein in the best BLASTp match, given in Table S4.
(DOC)

Table S4 **Annotation of the non-statistically significant hit EST sequences using BLASTp against the entire NCBI nr protein database.** Each EST sequence hit was submitted to a BLASTp search against the entire NCBI nr protein database. For each search, the highest scoring hit score (significance threshold >44, p-value<0.01), its accession number and protein name are reported. For information, the species corresponding to the highest scoring hit, its molecular function according to the NCBI nr database and Wormbase when available^1^ and the theoretical Mw/p*I* of its full length sequence are also described.
(DOC)

Table S5 **Q-TOF MS/MS protein spot identifications using the** ***H. contortus*** **putative EST protein database.** Each protein spot was excised from the 500 µg protein-loaded gel and analysed by Q-TOF MS/MS. A local MASCOT Ion search of the *H. contortus* putative EST protein database was performed and the highest scoring EST sequence match along with its MOWSE-based score (significance threshold score >25, p-value<0.05), sequence coverage and the number of matched peptides is reported. For each search, the highest scoring hit EST sequence accession number and its theoretical Mw/p*I* are also detailed.
(DOC)

Table S6 **Annotation of the hit EST sequences from Table S5 using BLASTp searches against the entire NCBI nr protein database.** Each EST sequence hit was submitted to a BLASTp search against the entire NCBI nr protein database. For each search, the highest scoring hit score (significance threshold >44, p-value<0.01), its accession number and protein name are reported. For information, the species for the highest scoring hit, its molecular function according to Wormbase^1^ and the theoretical Mw/pI of its sequence are also described.
(DOC)

Table S7 **Q-TOF MS/MS protein spot identifications using FASTS against the NCBI nr protein database.** Each protein spot was excised from the 500 µg protein-loaded gel and analysed by Q-TOF MS/MS. Selected peptides were then subjected to manual *de novo* sequencing and reported in this table. These peptide sequences were finally submitted to a FASTS search against the entire NCBI nr protein database. For each search, the highest scoring hit score (significance threshold >44, p-value<0.01) and corresponding e-value, accession number, protein name and species are also reported. Identical amino-acids between query sequence and FASTS search highest scoring hit are shown as underlined text.
(DOC)
