# Supplementary material for: Proteomic Profiling and Protein Identification by MALDI-TOF Mass Spectrometry in Unsequenced Parasitic Nematodes
Source: PLoS One. 2012 Mar 29;7(3):e33590. doi: 10.1371/journal.pone.0033590 (PMC3315570; doi:10.1371/journal.pone.0033590)
Supplement: Figure S1 — (S1.01–S1.64) Protein spot identification by MALDI-TOF MS in conjunction with PMF database searching. Results output using MASCOT searches for each PMF search for the 2D gel spots summarised in Table 1 and Tables S1, S2, S3 and S4 are given. (PDF) [file pone.0033590.s001.pdf]

Figure S1 (S1.01 – S1.64). Protein spot identification by MALDI-TOF MS in conjunction with PMF database searching.

Results output using MASCOT searches for each PMF search for the 2D gel spots summarized in Table 1 and Supplementary Multi-Media Tables S1, S2, S3 and S4 are given.

Figure S1.01

Mascot Search Results Spot 2

User : Paul Millares
Email : paul.millares@gmail.com
Search title : Spot 2
Database : Haemonchus 210108 (6387 sequences; 918038 residues)
Timestamp : 1 Aug 2011 at 10:11:04 GMT
Top Score : 34 for HCP03377_1, putative nuclear encoded protein Method: similarity and extension

Mascot Score Histogram

Protein score is -10*Log(P), where P is the probability that the observed match is a random event.

Protein scores greater than 51 are significant (p<0.05).

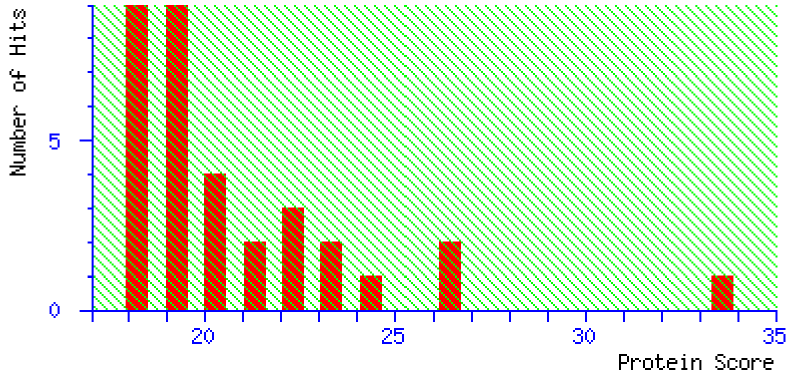

Concise Protein Summary Report

1. HCP03377_1 **Mass:** 45357 **Score:** 34 **Expect:** 2.8 **Matches:** 7
putative nuclear encoded protein Method: similarity and extension
HCP09476_1 **Mass:** 6889 **Score:** 21 **Expect:** 56 **Matches:** 2
putative nuclear encoded protein Method: Longest ORF
HCP03443_2 **Mass:** 21481 **Score:** 19 **Expect:** 80 **Matches:** 3
putative nuclear encoded protein Method: ESTScan

HCP03443_1 **Mass:** 21481 **Score:** 19 **Expect:** 80 **Matches:** 3
putative nuclear encoded protein Method: ESTScan
HCP08004_1 **Mass:** 18216 **Score:** 19 **Expect:** 88 **Matches:** 3
putative nuclear encoded protein Method: similarity and extension
HCP08004_2 **Mass:** 18544 **Score:** 18 **Expect:** 94 **Matches:** 3
putative nuclear encoded protein Method: similarity and extension

---

Search Parameters

Type of search : Peptide Mass Fingerprint
Enzyme : Trypsin
Mass values : Monoisotopic
Protein Mass : Unrestricted
Peptide Mass Tolerance : ± 1.2 Da
Peptide Charge State : 1+
Max Missed Cleavages : 1
Number of queries : 17

Protein View

Match to: **HCP03377_1** Score: **34** Expect: **2.8**
putative nuclear encoded protein Method: similarity and extension

Nominal mass (M_r): **45357**; Calculated pI value: **5.93**
NCBI BLAST search of HCP03377_1 against nr
Unformatted sequence string for pasting into other applications

Cleavage by Trypsin: cuts C-term side of KR unless next residue is P
Number of mass values searched: **17**
Number of mass values matched: **7**
Sequence Coverage: **19%**

Matched peptides shown in **Bold Red**

```
  1 RLAGANIARS FVRSYAKDIK FGAEGRKAML VGVDLLADAV SVTMGPKGRN
 51 VILEQSWGSP KITKDGVTVA KAIDLKDKYH NMGAKLIQDV ANKTNEEAGD
101 GTTCATILAR SITKEGFDNI SKGANAVEIR RGVMAAVELI VKDLKQQSKQ
151 VTTPEEIAQV ATISANGDSN IGNLISEAMK KVGRKGVITV KDGKTLNDEL
201 ELIEGMKFDR GYISPYFINT AKGAKVEYEK ALVLLSEKKI NNVQDIVPAL
```

```
251 ELANKVRKPL VVIAEDVDGE ALTTLVLNRL KVGLQVVAVK APGFGDNRKN
301 TLRDMAIATG GTVFGDDTNL VKLEDIQLSD FGEVEEVTIT KDDTLLLRGK
351 GESAEIEKRI EQIADEIEQS TSDYEKEKLN ERLAKLSKGV AVLKVGGASE
401 VEVSEKKDRV TDALCATRAA V
```

Start - End	Observed	Mr(expt)	Mr(calc)	Delta	Miss	Sequence
50 - 61	1358.13	1357.12	1356.70	0.42	0	R.NVILEQSWGSPK.I
86 - 93	900.70	899.70	899.51	0.19	0	K.LIQDVANK.T
211 - 222	1374.11	1373.10	1372.70	0.40	0	R.GYISPYFINTAK.G
223 - 230	922.69	921.68	922.48	-0.80	1	K.GAKVEYEK.A
258 - 279	2365.74	2364.74	2364.33	0.41	0	R.KPLVVIAEDVDGEALTTLVLNR.L
282 - 290	912.77	911.76	911.58	0.18	0	K.VGLQVVAVK.A
291 - 299	961.67	960.66	960.48	0.19	1	K.APGFGDNRK.N

No match to: 907.70, 934.75, 951.45, 1006.69, 1146.09, 1302.18, 1538.24, 1599.18, 1608.31, 1939.35

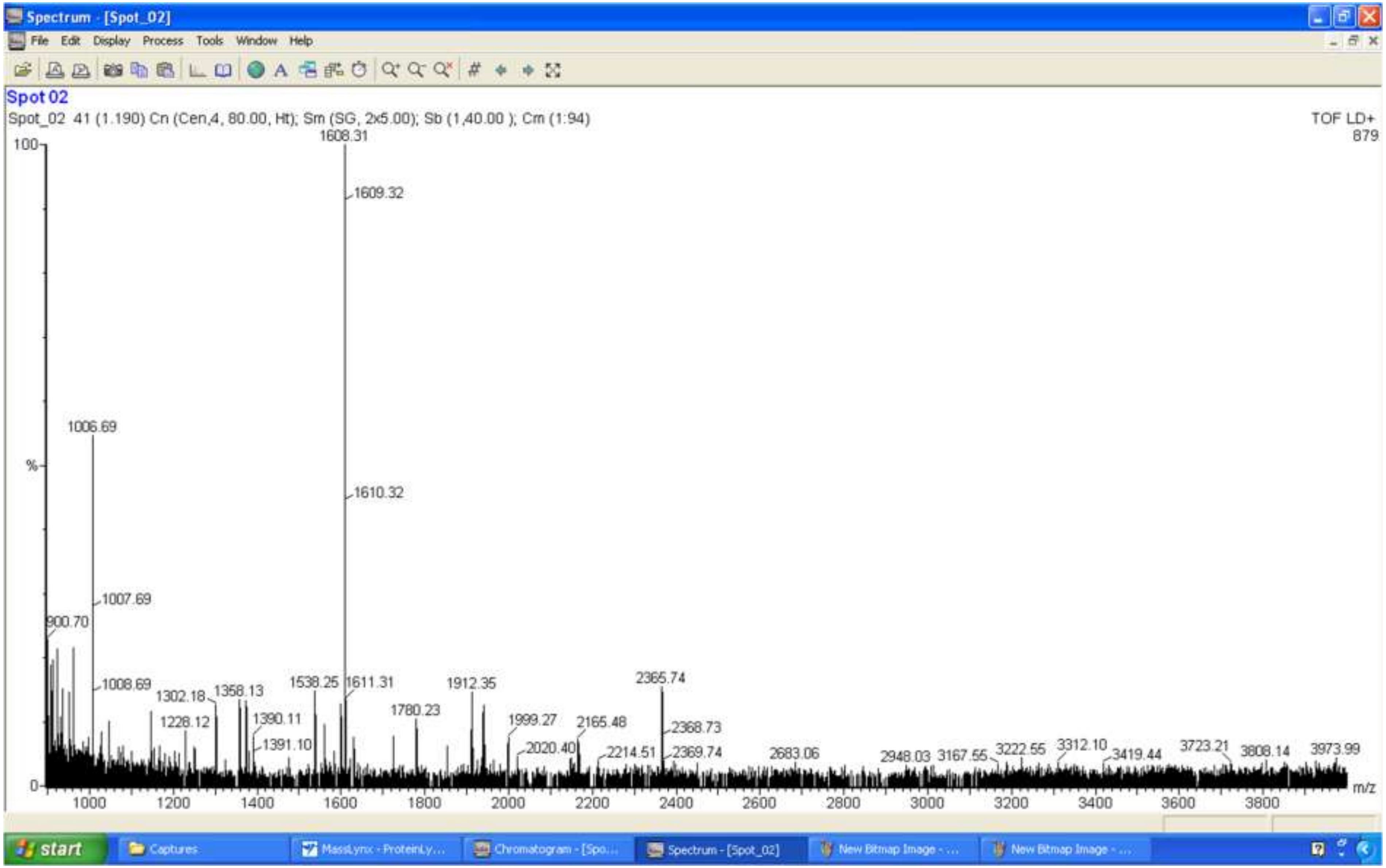
Spectrum - [Spot_02]
File Edit Display Process Tools Window Help
Spot 02
Spot_02 41 (1.190) Cn (Cen,4, 80.00, Ht); Sm (SG, 2x5.00); Sb (1,40.00); Cm (1:94)
TOF LD+
879
1608.31
1609.32
1610.32
1006.69
1007.69
900.70
1008.69
1302.18
1358.13
1228.12
1390.11
1391.10
1538.25
1611.31
1780.23
1912.35
1999.27
2020.40
2165.48
2214.51
2365.74
2368.73
2369.74
2683.06
2948.03
3167.55
3222.55
3312.10
3419.44
3723.21
3808.14
3973.99
100
%
0
m/z
start
Captures
MassLynx - ProteinLy...
Chromatogram - [Spo...
Spectrum - [Spot_02]
New Bitmap Image - ...
New Bitmap Image - ...

Figure S1.02

Mascot Search Results Spot 4

User : **Paul Millares**
Email : **paul.millares@gmail.com**
Search title : **Spot 4**
Database : **Haemonchus 210108 (6387 sequences; 918038 residues)**
Timestamp : **1 Aug 2011 at 10:16:19 GMT**
Top Score : **65 for HCP00006_1, putative nuclear encoded protein Method: similarity and extension**

Mascot Score Histogram

Protein score is -10*Log(P), where P is the probability that the observed match is a random event.

Protein scores greater than 51 are significant (p<0.05).

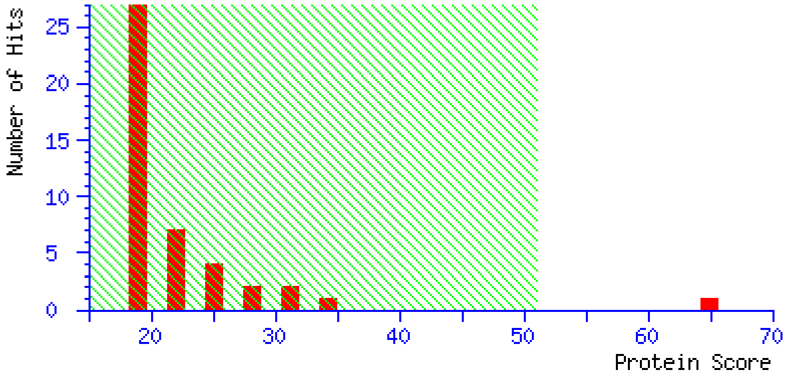

Concise Protein Summary Report

1. HCP00006_1 **Mass:** 59610 **Score:** **65** **Expect:** 0.0021 **Matches:** 20
putative nuclear encoded protein Method: similarity and extension
HCP00006_2 **Mass:** 59610 **Score:** **65** **Expect:** 0.0021 **Matches:** 20
putative nuclear encoded protein Method: similarity and extension

HCP00006_3 **Mass:** 59543 **Score:** 29 **Expect:** 9 **Matches:** 13
putative nuclear encoded protein Method: similarity and extension
HCP05669_1 **Mass:** 5537 **Score:** 20 **Expect:** 70 **Matches:** 3
putative nuclear encoded protein Method: Longest ORF

---

2. HCP00091_1 **Mass:** 32524 **Score:** 33 **Expect:** 2.9 **Matches:** 10
putative nuclear encoded protein Method: similarity and extension

---

Search Parameters

Type of search : **Peptide Mass Fingerprint**
Enzyme : **Trypsin**
Variable modifications : **Carbamidomethyl (C),Glu->pyro-Glu (N-term E),Oxidation (M)**
Mass values : **Monoisotopic**
Protein Mass : **Unrestricted**
Peptide Mass Tolerance : **± 1.2 Da**
Peptide Charge State : **1+**
Max Missed Cleavages : **1**
Number of queries : **34**

Protein View

Match to: **HCP00006_1** Score: **65** Expect: **0.0021**
putative nuclear encoded protein Method: similarity and extension

Nominal mass (M_r): **59610**; Calculated pI value: **6.67**
NCBI BLAST search of HCP00006_1 against nr
Unformatted sequence string for pasting into other applications

Variable modifications: Carbamidomethyl (C),Glu->pyro-Glu (N-term E),Oxidation (M)
Cleavage by Trypsin: cuts C-term side of KR unless next residue is P
Number of mass values searched: **34**
Number of mass values matched: **20**
Sequence Coverage: **30%**

Matched peptides shown in **Bold Red**

1 MLSNLARTSG RMAFIRGISS AQMDAHAQVI DDQKPMEEQS NPSFFKMVDY
51 YFDKGASVIE PKLVEEMKSN VMSTKDKKNL VSGILKAIKP VNK**VLYITFP**

101 **IRR**DNGEFEV IEAWRAQHSE HRTPTK**GGIR YSMDVCEDEV KALSALMTYK**
151 CAAVDVPFGG AKGGVKIDPK QYTDYEIEKI TR**RIAIEFAK KGFLGPGVDV**
201 **PAPDMGTGER** EMGWIADTYA QTIGHLDR**DA SACITGKPIV AGGIHGR**VSA
251 TGRGVWKGLE VFTK**EPEYMN KVGLSLGLEG K**TIIIQGFGN VGLHTMRYLH
301 RAGAKVIGVQ EWDCAVFNPD GIHPK**ELEDW RDENGTIK**NF PKAKNFEPFA
351 ELMYEPCDIF VPAACEKAIH KENANRIQAK IIAEAANGPT TPAADKILLE
401 RGNCLIIPDM FINSGGVTVS YFEWLK**NLNH VSYGR**LSFK**Y EEDSNR**MLLQ
451 SVQDSLEKAL NK**EAPVHPND EFTAR**IAGAS EK**DIVHSGLE YTMTR**SGEAI
501 IRTAR**KYNLG LDIR**TAAYAN SIEKVYNTYR TAGFTFT

Start - End	Observed	Mr(expt)	Mr(calc)	Delta	Miss	Sequence
94 - 102	1122.07	1121.06	1120.66	0.40	0	K.VLYITFPIR.R
94 - 103	1278.23	1277.23	1276.77	0.46	1	K.VLYITFPIRR.D
127 - 141	1773.41	1772.40	1772.77	-0.37	1	K.GGIRYSMDVCEDEVK.A Carbamidomethyl (C); Oxidation (M)
142 - 150	997.50	996.50	996.53	-0.03	0	K.ALSALMTYK.C
142 - 150	1013.76	1012.75	1012.53	0.22	0	K.ALSALMTYK.C Oxidation (M)
183 - 190	947.78	946.77	946.56	0.21	1	R.RIAIEFAK.K
184 - 191	919.49	918.49	918.55	-0.07	1	R.IAIEFAKK.G
191 - 210	2016.47	2015.46	2014.98	0.48	1	K.KGFLGPGVDVPAPDMGTGER.E Oxidation (M)
229 - 247	1880.46	1879.45	1878.97	0.48	0	R.DASACITGKPIVAGGIHGR.V Carbamidomethyl (C)
265 - 271	907.49	906.49	907.37	-0.89	0	K.EPEYMNK.V Glu->pyro-Glu (N-term E); Oxidation (M)
265 - 271	926.55	925.54	925.39	0.16	0	K.EPEYMNK.V Oxidation (M)
272 - 281	972.78	971.77	971.57	0.21	0	K.VGLSLGLEGK.T
326 - 338	1605.21	1604.20	1603.75	0.45	1	K.ELEDWRDENGTIK.N
427 - 435	1059.75	1058.75	1058.53	0.22	0	K.NLNHVSYGR.L
440 - 446	912.57	911.56	911.36	0.20	0	K.YEEDSNR.M
463 - 475	1465.14	1464.13	1463.68	0.45	0	K.EAPVHPNDEFTAR.I Glu->pyro-Glu (N-term E)
463 - 475	1483.16	1482.15	1481.69	0.46	0	K.EAPVHPNDEFTAR.I
483 - 495	1538.19	1537.19	1536.72	0.46	0	K.DIVHSGLEYTMTR.S Oxidation (M)
506 - 514	1091.87	1090.87	1090.61	0.25	1	R.KYNLGLDIR.T
507 - 514	963.74	962.73	962.52	0.22	0	K.YNLGLDIR.T

No match to: 900.48, 938.65, 951.49, 969.74, 976.78, 1045.78, 1077.43, 1113.96, 1144.12, 1448.11, 1480.11, 1497.13, 1889.37, 2125.45

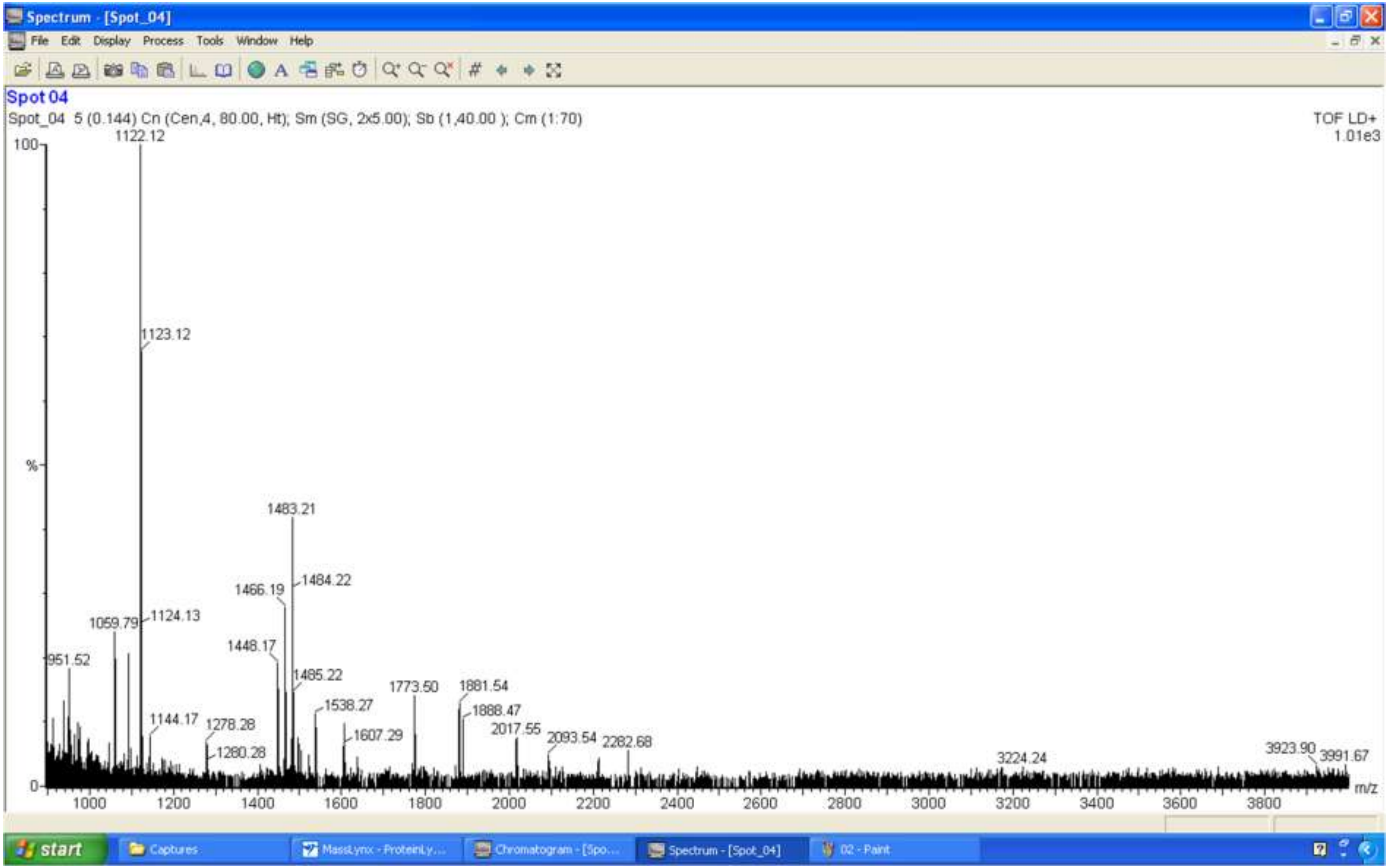
Spectrum - [Spot_04]
File Edit Display Process Tools Window Help
Spot 04
Spot_04 5 (0.144) Cn (Cen,4, 80.00, Ht); Sm (SG, 2x5.00); Sb (1,40.00); Cm (1:70)
TOF LD+
1.01e3
100
%
0
1122.12
1123.12
1124.13
1059.79
951.52
1144.17
1278.28
1280.28
1448.17
1466.19
1483.21
1484.22
1485.22
1538.27
1607.29
1773.50
1881.54
1888.47
2017.55
2093.54
2282.68
3224.24
3923.90
3991.67
1000
1200
1400
1600
1800
2000
2200
2400
2600
2800
3000
3200
3400
3600
3800
m/z
start
Captures
MassLynx - ProteinLy...
Chromatogram - [Spo...
Spectrum - [Spot_04]
02 - Paint

Figure S1.03

Mascot Search Results Spot 6

User : Paul Millares
Email : paul.millares@gmail.com
Search title : Spot 6
Database : Haemonchus 210108 (6387 sequences; 918038 residues)
Timestamp : 1 Aug 2011 at 10:17:24 GMT
Top Score : 47 for HCP00273_1, putative nuclear encoded protein Method: similarity and extension

Mascot Score Histogram

Protein score is -10*Log(P), where P is the probability that the observed match is a random event.

Protein scores greater than 51 are significant (p<0.05).

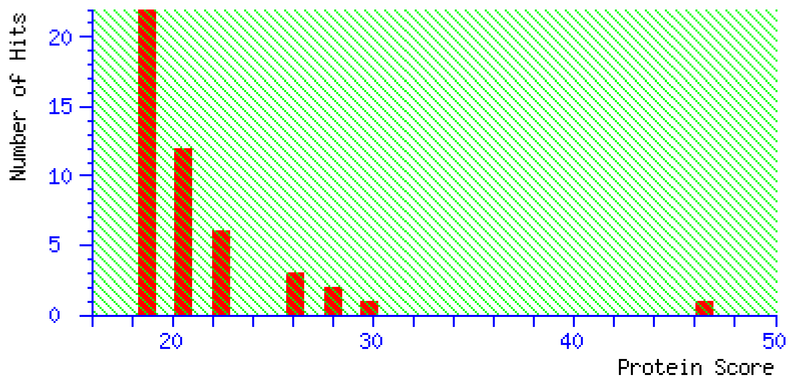

Concise Protein Summary Report

1. HCP00273_1 **Mass:** 26452 **Score:** 47 **Expect:** 0.14 **Matches:** 11

putative nuclear encoded protein Method: similarity and extension

Search Parameters

Type of search : Peptide Mass Fingerprint
Enzyme : Trypsin
Variable modifications : Carbamidomethyl (C),Glu->pyro-Glu (N-term E),Oxidation (M)
Mass values : Monoisotopic
Protein Mass : Unrestricted
Peptide Mass Tolerance : ± 1.2 Da
Peptide Charge State : 1+
Max Missed Cleavages : 1
Number of queries : 36

Protein View

Match to: **HCP00273_1** Score: **47** Expect: **0.14**
putative nuclear encoded protein Method: similarity and extension

Nominal mass (M_r): **26452**; Calculated pI value: **6.04**
NCBI BLAST search of HCP00273_1 against nr
Unformatted sequence string for pasting into other applications

Variable modifications: Carbamidomethyl (C),Glu->pyro-Glu (N-term E),Oxidation (M)
Cleavage by Trypsin: cuts C-term side of KR unless next residue is P
Number of mass values searched: **36**
Number of mass values matched: **11**
Sequence Coverage: **49%**

Matched peptides shown in **Bold Red**

```
  1 PRAAGIRHEF KLTCVFLFAL SAFAATVEEE KNVIVLTKDN FDEVINSHEF
 51 VLAEFYAPWC GHCKALAPEY EKAATQLKEE GSAIKLAKLD ATVHGDVASK
101 FEVRGYPTLK LFRNGKHSEY TGGRDAASIV AWLKKKTGPV AKTLKTADDV
151 KALQEEADVV VVGYFKNVDG EKAKVFLEVA SGIDDIPFGI TTESAAKKHL
201 ELKDEGIVLL KKFDEGRDVF EEKHTADAIK AWIQANRLAL
```

Start - End	Observed	Mr(expt)	Mr(calc)	Delta	Miss	Sequence
65 - 72	919.77	918.76	919.47	-0.70	0	K.ALAPEYEK.A
79 - 88	1044.83	1043.82	1044.58	-0.76	1	K.EEGSAIKLAK.L
89 - 100	1213.16	1212.16	1211.61	0.54	0	K.LDATVHGDVASK.F
117 - 124	905.65	904.64	905.40	-0.76	0	K.HSEYTGGR.D
117 - 124	906.64	905.63	905.40	0.24	0	K.HSEYTGGR.D
125 - 134	1073.83	1072.83	1072.59	0.24	0	R.DAASIVAWLK.K
137 - 145	913.75	912.75	913.56	-0.81	1	K.TGPVAKTLK.T

152 - 166	1667.39	1666.38	1665.86	0.52	0	K.ALQEEADVVVVGYFK.N
175 - 197	2380.82	2379.81	2379.22	0.59	0	K.VFLEVASGIDDIPFGITTESAAK.K
199 - 211	1507.38	1506.37	1505.88	0.49	1	K.HLELKDEGIVLLK.K
213 - 223	1371.13	1370.12	1369.62	0.51	1	K.FDEGRDVFEEK.H

No match to: 900.51, 928.60, 938.71, 951.52, 960.69, 970.55, 984.75, 997.55, 1000.77, 1016.78, 1059.76, 1066.82, 1105.94, 1122.09, 1195.14, 1235.15, 1287.19, 1309.18, 1483.20, 1499.23, 1689.37, 1765.44, 1768.43, 1787.42, 2211.66

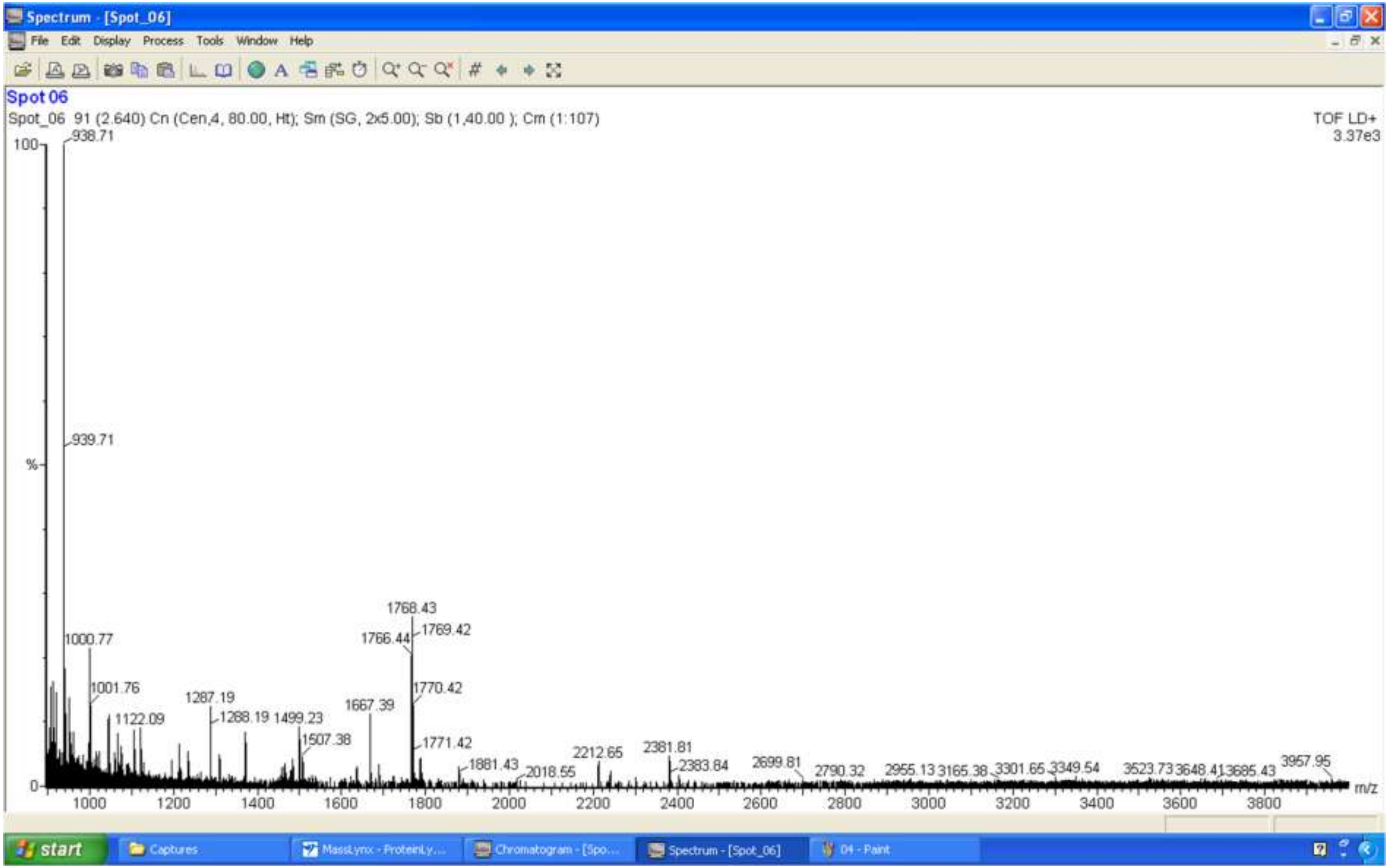
Spectrum - [Spot_06]
File Edit Display Process Tools Window Help
Spot 06
Spot_06 91 (2.640) Cn (Cen,4, 80.00, Ht); Sm (SG, 2x5.00); Sb (1,40.00); Cm (1:107)
TOF LD+
3.37e3
100
%
0
938.71
939.71
1000.77
1001.76
1122.09
1287.19
1288.19
1499.23
1507.38
1667.39
1766.44
1768.43
1769.42
1770.42
1771.42
1881.43
2018.55
2212.65
2381.81
2383.84
2699.81
2790.32
2955.13
3165.38
3301.65
3349.54
3523.73
3648.41
3685.43
3957.95
1000
1200
1400
1600
1800
2000
2200
2400
2600
2800
3000
3200
3400
3600
3800
m/z
start
Captures
MassLynx - ProteinLy...
Chromatogram - [Spo...
Spectrum - [Spot_06]
04 - Paint

Figure S1.04

Mascot Search Results Spot 7

```
User            : Paul Millares
Email           : paul.millares@gmail.com
Search title    : Spot 7
Database        : Haemonchus 210108 (6387 sequences; 918038 residues)
Timestamp       : 1 Aug 2011 at 10:18:02 GMT
Top Score       : 41 for HCP00592_1, putative nuclear encoded protein  Method: similarity and extension
```

Mascot Score Histogram

Protein score is -10*Log(P), where P is the probability that the observed match is a random event.

Protein scores greater than 51 are significant (p<0.05).

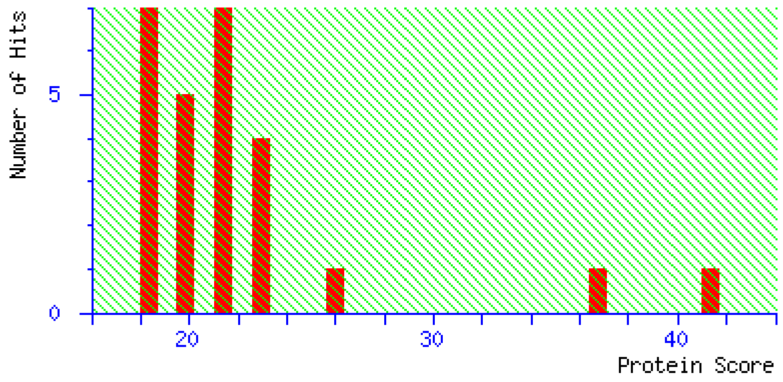

Concise Protein Summary Report

1. HCP00592_1 **Mass:** 46935 **Score:** 41 **Expect:** 0.47 **Matches:** 8
   putative nuclear encoded protein Method: similarity and extension
   HCP00592_2 **Mass:** 28964 **Score:** 29 **Expect:** 7.7 **Matches:** 6
   putative nuclear encoded protein Method: similarity and extension

HCP05171_1 **Mass:** 23268 **Score:** 23 **Expect:** 30 **Matches:** 4
putative nuclear encoded protein Method: similarity and extension

HCP02527_1 **Mass:** 10836 **Score:** 20 **Expect:** 60 **Matches:** 3
putative nuclear encoded protein Method: ESTScan

HCP05114_3 **Mass:** 3696 **Score:** 20 **Expect:** 70 **Matches:** 2
putative nuclear encoded protein Method: ESTScan

HCP05153_1 **Mass:** 4619 **Score:** 19 **Expect:** 73 **Matches:** 2
putative nuclear encoded protein Method: Longest ORF

HCP02046_1 **Mass:** 14122 **Score:** 19 **Expect:** 75 **Matches:** 3
putative nuclear encoded protein Method: ESTScan

HCP12613_1 **Mass:** 15007 **Score:** 19 **Expect:** 90 **Matches:** 3
putative nuclear encoded protein Method: similarity and extension

---

Search Parameters

Type of search : Peptide Mass Fingerprint
Enzyme : Trypsin
Variable modifications : Carbamidomethyl (C),Glu->pyro-Glu (N-term E),Oxidation (M)
Mass values : Monoisotopic
Protein Mass : Unrestricted
Peptide Mass Tolerance : ± 1.2 Da
Peptide Charge State : 1+
Max Missed Cleavages : 1
Number of queries : 14

Protein View

Match to: **HCP00592_1** Score: **41** Expect: **0.47**
putative nuclear encoded protein Method: similarity and extension

Nominal mass (M_r): **46935**; Calculated pI value: **4.77**
NCBI BLAST search of HCP00592_1 against nr
Unformatted sequence string for pasting into other applications

Variable modifications: Carbamidomethyl (C),Glu->pyro-Glu (N-term E),Oxidation (M)
Cleavage by Trypsin: cuts C-term side of KR unless next residue is P
Number of mass values searched: **14**

Number of mass values matched: **8**
Sequence Coverage: **18%**

Matched peptides shown in **Bold Red**

```
  1 MRSLALLLPL LGIVAAEIFF KEEFLDDSWE KRWVQSKHKD DYGAFKLSAG
 51 KYYDDAKRDQ GLKTSQDAKF YSLAAKFPKK FTNKGKTVVI QYTVKHEQGI
101 DCGGGYVKVM SSDVDLKDFH GETPYNVMFG PDICGPTKKV HVIFSYKGKN
151 HLIKKDIRGK DDELTHLYTL ILNPDNTYEV KIDGEKVESG ELEADWDMLP
201 PKKIKDPDAK KPEDWDEREY IDDADDKKPE DWDKPEHIPD PDAKKPDDWD
251 DEMDGEWEPP MIDNPEYKGE WKPKQIKNPD YKGKWIHPEI DNPEYTPDDE
301 LYLYKDWGAI GFDLWQVKSG TIFDNILVTD SVDDAKAHAA ETFEKLKAVE
351 KEKKDKADEE ERKKIEEEAK KREEEDKKKK EAKEKEEKED EDEDKEEEAH
401 DEL
```

Start - End	Observed	Mr(expt)	Mr(calc)	Delta	Miss	Sequence
38 - 46	1080.75	1079.75	1079.50	0.24	1	K.HKDDYGAFK.L
52 - 58	930.67	929.66	929.42	0.23	1	K.YYDDAKR.D
87 - 95	1050.86	1049.85	1049.61	0.24	0	K.TVVIQYTVK.H
96 - 108	1420.12	1419.11	1418.62	0.49	0	K.HEQGIDCGGGYVK.V Carbamidomethyl (C)
139 - 147	1121.06	1120.05	1119.64	0.41	1	K.KVHVIFSYK.G
140 - 147	992.79	991.79	991.55	0.24	0	K.VHVIFSYK.G
319 - 336	1910.44	1909.43	1908.93	0.50	0	K.SGTIFDNILVTDSVDDAK.A
337 - 345	1003.73	1002.72	1002.48	0.25	0	K.AHAAETFEK.L

No match to: 951.50, 1014.77, 1025.70, 1045.81, 1072.84, 1102.82

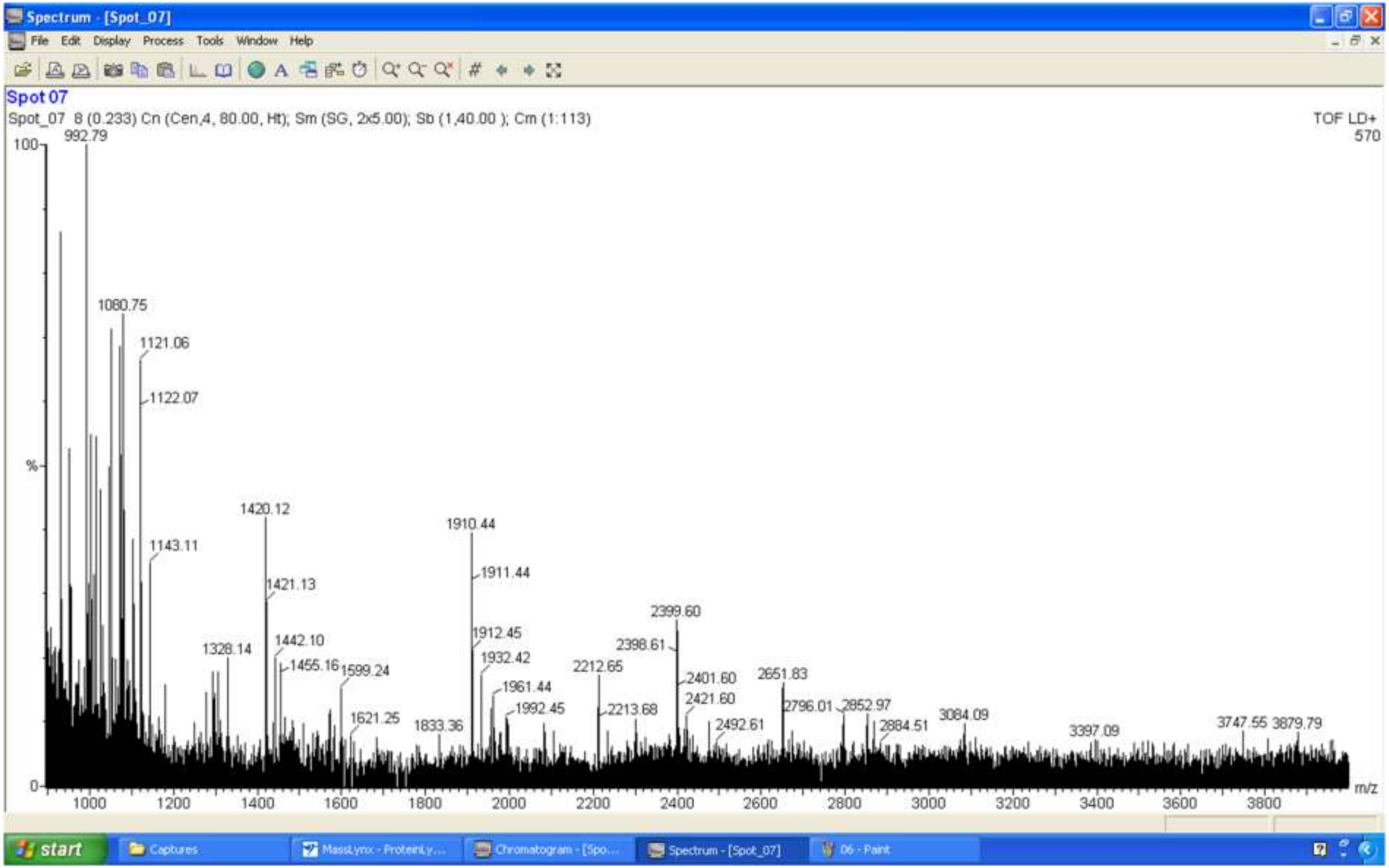

Spectrum - [Spot_07]
File Edit Display Process Tools Window Help
Spot 07
Spot_07 8 (0.233) Cn (Cen,4, 80.00, Ht); Sm (SG, 2x5.00); Sb (1,40.00); Cm (1:113)
TOF LD+
570
100
%
0
992.79
1080.75
1121.06
1122.07
1143.11
1328.14
1420.12
1421.13
1442.10
1455.16
1599.24
1621.25
1833.36
1910.44
1911.44
1912.45
1932.42
1961.44
1992.45
2212.65
2213.68
2398.61
2399.60
2401.60
2421.60
2492.61
2651.83
2796.01
2852.97
2884.51
3084.09
3397.09
3747.55
3879.79
1000 1200 1400 1600 1800 2000 2200 2400 2600 2800 3000 3200 3400 3600 3800
m/z
start
Captures
MassLynx - ProteinLy...
Chromatogram - [Spo...
Spectrum - [Spot_07]
06 - Paint

Figure S1.05

Mascot Search Results Spot 8

```
User             : Paul Millares
Email            : paul.millares@gmail.com
Search title     : Spot 8
Database         : Haemonchus 210108 (6387 sequences; 918038 residues)
Timestamp        : 1 Aug 2011 at 10:23:22 GMT
Top Score        : 58 for HCP00006_1, putative nuclear encoded protein  Method: similarity and extension
```

Mascot Score Histogram

Protein score is -10*Log(P), where P is the probability that the observed match is a random event.

Protein scores greater than 51 are significant (p<0.05).

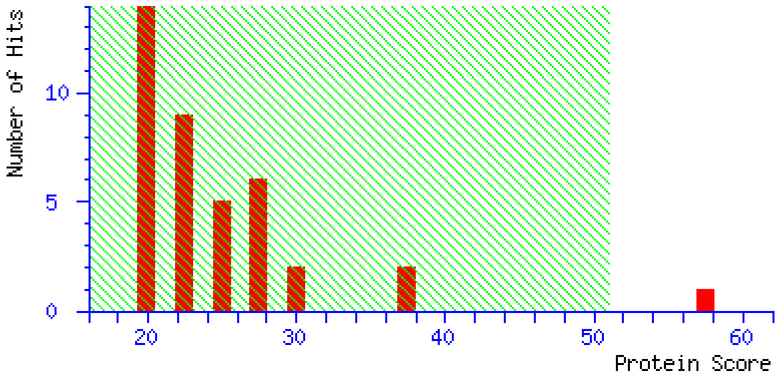

Concise Protein Summary Report

1. HCP00006_1 **Mass:** 59610 **Score:** 58 **Expect:** 0.011 **Matches:** 14

   putative nuclear encoded protein Method: similarity and extension

   HCP00006_2 **Mass:** 59610 **Score:** 58 **Expect:** 0.011 **Matches:** 14

putative nuclear encoded protein Method: similarity and extension

HCP00006_3 **Mass:** 59543 **Score:** 31 **Expect:** 5.4 **Matches:** 10

putative nuclear encoded protein Method: similarity and extension

HCP06413_2 **Mass:** 21950 **Score:** 24 **Expect:** 23 **Matches:** 5

putative nuclear encoded protein Method: similarity and extension

HCP09394_1 **Mass:** 5341 **Score:** 24 **Expect:** 26 **Matches:** 5

putative nuclear encoded protein Method: similarity and extension

HCP01303_2 **Mass:** 28007 **Score:** 21 **Expect:** 54 **Matches:** 5

putative nuclear encoded protein Method: similarity and extension

HCP00304_1 **Mass:** 9136 **Score:** 20 **Expect:** 65 **Matches:** 4

putative nuclear encoded protein Method: similarity and extension

HCP10570_1 **Mass:** 9759 **Score:** 20 **Expect:** 65 **Matches:** 3

putative nuclear encoded protein Method: ESTScan

---

2. HCP07935_1 **Mass:** 6052 **Score:** 37 **Expect:** 1.3 **Matches:** 4

putative nuclear encoded protein Method: ESTScan

Search Parameters

Type of search : Peptide Mass Fingerprint
Enzyme : Trypsin
Variable modifications : Carbamidomethyl (C),Glu->pyro-Glu (N-term E),Oxidation (M)
Mass values : Monoisotopic
Protein Mass : Unrestricted
Peptide Mass Tolerance : ± 1.2 Da
Peptide Charge State : 1+
Max Missed Cleavages : 1
Number of queries : 21

Protein View

Match to: **HCP00006_1** Score: **58** Expect: **0.011**
putative nuclear encoded protein Method: similarity and extension

Nominal mass (M_r): **59610**; Calculated pI value: **6.67**

NCBI BLAST search of HCP00006 1 against nr
Unformatted sequence string for pasting into other applications

Variable modifications: Carbamidomethyl (C),Glu->pyro-Glu (N-term E),Oxidation (M)
Cleavage by Trypsin: cuts C-term side of KR unless next residue is P
Number of mass values searched: **21**
Number of mass values matched: **14**
Sequence Coverage: **24%**

Matched peptides shown in **Bold Red**

1 MLSNLARTSG RMAFIRGISS AQMDAHAQVI DDQKPMEEQS NPSFFKMVDY
51 YFDKGASVIE PKLVEEMKSN VMSTKDKKNL VSGILKAIKP VNK**VLYITFP**
101 **IRR**DNGEFEV IEAWRAQHSE HRTPTKGGIR YSMDVCEDEV KALSALMTYK
151 CAAVDVPFGG AKGGVKIDPK QYTDYEIEKI TRRIAIEFAK K**GFLGPGVDV**
201 **PAPDMGTGER** EMGWIADTYA QTIGHLDR**DA** **SACITGKPIV** **AGGIHGR**VSA
251 TGRGVWKGLE VFTK**EPEYMN** **KVGLSLGLEG** **KTIIIQGFGN** **VGLHTMR**YLH
301 RAGAKVIGVQ EWDCAVFNPD GIHPKELEDW RDENGTIKNF PKAKNFEPFA
351 ELMYEPCDIF VPAACEKAIH KENANRIQAK IIAEAANGPT TPAADKILLE
401 RGNCLIIPDM FINSGGVTVS YFEWLK**NLNH** **VSYGR**LSFK**Y** **EEDSNR**MLLQ
451 SVQDSLEKAL NK**EAPVHPND** **EFTAR**IAGAS EK**DIVHSGLE** **YTMTR**SGEAI
501 IRTAR**KYNLG** **LDIR**TAAYAN SIEKVYNTYR TAGFTFT

**Start - End**	**Observed**	**Mr(expt)**	**Mr(calc)**	**Delta**	**Miss**	**Sequence**
**94 - 102**	**1121.89**	**1120.88**	**1120.66**	**0.22**	**0**	**K.VLYITFPIR.R**
**94 - 103**	**1278.04**	**1277.03**	**1276.77**	**0.26**	**1**	**K.VLYITFPIRR.D**
**192 - 210**	**1889.05**	**1888.05**	**1886.88**	**1.16**	**0**	**K.GFLGPGVDVPAPDMGTGER.E** Oxidation (M)
**229 - 247**	**1880.22**	**1879.22**	**1878.97**	**0.24**	**0**	**R.DASACITGKPIVAGGIHGR.V** Carbamidomethyl (C)
**265 - 271**	**907.36**	**906.36**	**907.37**	**-1.02**	**0**	**K.EPEYMNK.V** Glu->pyro-Glu (N-term E); Oxidation (M)
**265 - 271**	**925.37**	**924.36**	**925.39**	**-1.02**	**0**	**K.EPEYMNK.V** Oxidation (M)
**272 - 281**	**973.60**	**972.59**	**971.57**	**1.02**	**0**	**K.VGLSLGLEGK.T**
**282 - 297**	**1773.17**	**1772.16**	**1771.94**	**0.22**	**0**	**K.TIIIQGFGNVGLHTMR.Y** Oxidation (M)
**427 - 435**	**1059.58**	**1058.57**	**1058.53**	**0.05**	**0**	**K.NLNHVSYGR.L**
**440 - 446**	**913.40**	**912.39**	**911.36**	**1.03**	**0**	**K.YEEDSNR.M**
**463 - 475**	**1464.92**	**1463.91**	**1463.68**	**0.23**	**0**	**K.EAPVHPNDEFTAR.I** Glu->pyro-Glu (N-term E)
**463 - 475**	**1482.92**	**1481.92**	**1481.69**	**0.23**	**0**	**K.EAPVHPNDEFTAR.I**
**483 - 495**	**1537.97**	**1536.96**	**1536.72**	**0.23**	**0**	**K.DIVHSGLEYTMTR.S** Oxidation (M)
**506 - 514**	**1091.70**	**1090.69**	**1090.61**	**0.08**	**1**	**R.KYNLGLDIR.T**

No match to: 951.34, 976.62, 992.35, 1033.51, 1118.68, 1447.88, 1476.97

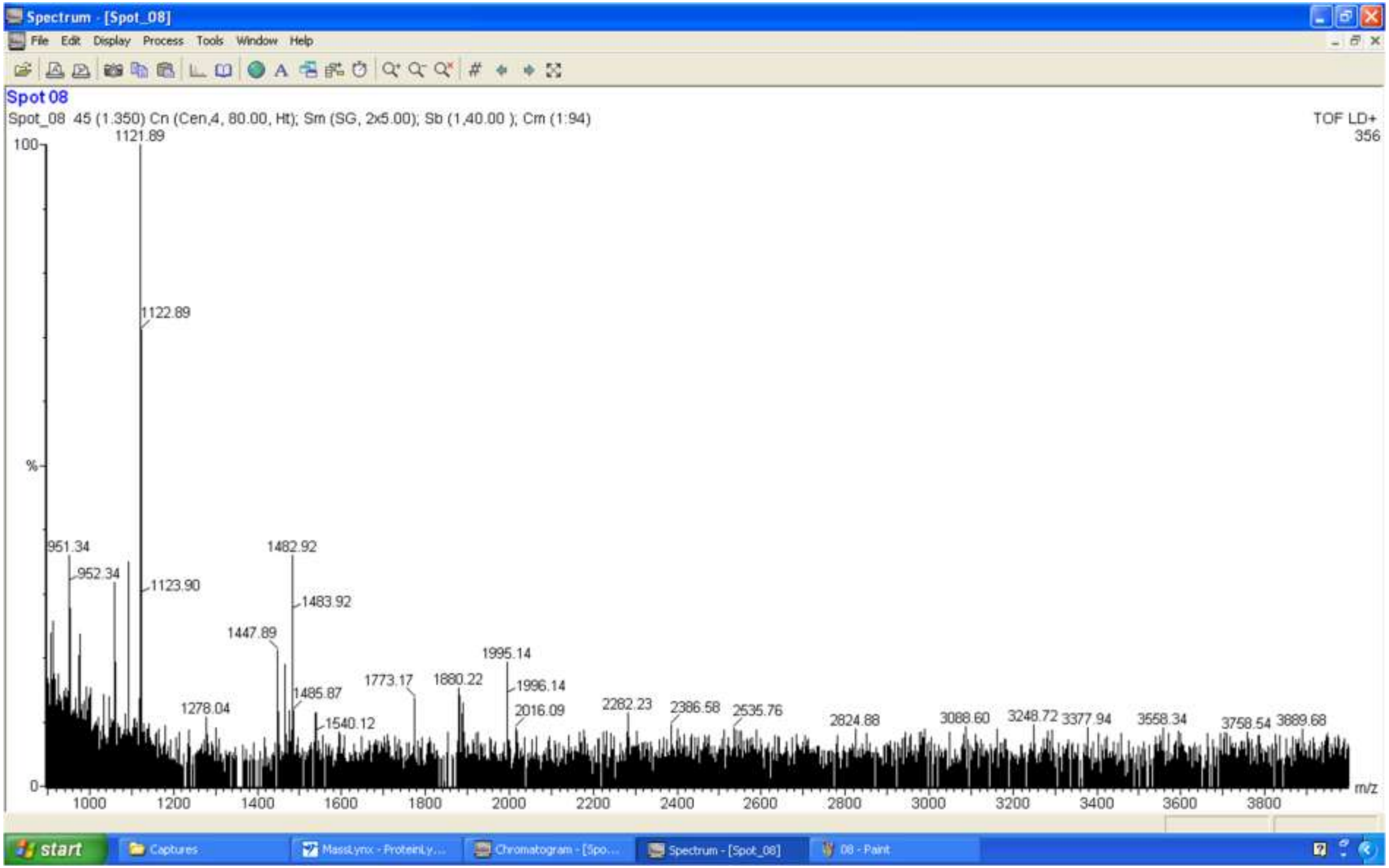
Spectrum - [Spot_08]
File Edit Display Process Tools Window Help
Spot 08
Spot_08 45 (1.350) Cn (Cen,4, 80.00, Ht); Sm (SG, 2x5.00); Sb (1,40.00); Cm (1:94)
TOF LD+
356
100
%
0
1121.89
1122.89
951.34
952.34
1123.90
1482.92
1483.92
1447.89
1485.87
1278.04
1540.12
1773.17
1880.22
1995.14
1996.14
2016.09
2282.23
2386.58
2535.76
2824.88
3088.60
3248.72
3377.94
3558.34
3758.54
3889.68
1000
1200
1400
1600
1800
2000
2200
2400
2600
2800
3000
3200
3400
3600
3800
m/z
start
Captures
MassLynx - ProteinLy...
Chromatogram - [Spo...
Spectrum - [Spot_08]
08 - Paint

Figure S1.06

Mascot Search Results Spot 9

User : Paul Millares
Email : paul.millares@gmail.com
Search title : Spot 9
Database : Haemonchus 210108 (6387 sequences; 918038 residues)
Timestamp : 1 Aug 2011 at 10:23:50 GMT
Top Score : 68 for HCP00006_1, putative nuclear encoded protein Method: similarity and extension

Mascot Score Histogram

Protein score is -10*Log(P), where P is the probability that the observed match is a random event.

Protein scores greater than 51 are significant (p<0.05).

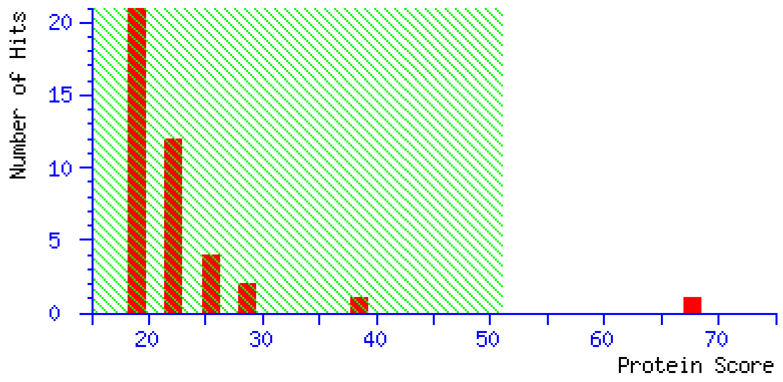

Concise Protein Summary Report

1. HCP00006_1 **Mass:** 59610 **Score:** **68** **Expect:** 0.0011 **Matches:** 18
putative nuclear encoded protein Method: similarity and extension
HCP00006_2 **Mass:** 59610 **Score:** **68** **Expect:** 0.0011 **Matches:** 18
putative nuclear encoded protein Method: similarity and extension
HCP06454_2 **Mass:** 8348 **Score:** 31 **Expect:** 5.1 **Matches:** 6
putative nuclear encoded protein Method: ESTScan

HCP06454_1 **Mass:** 8220 **Score:** 31 **Expect:** 5.3 **Matches:** 6
putative nuclear encoded protein Method: ESTScan
HCP00006_3 **Mass:** 59543 **Score:** 27 **Expect:** 13 **Matches:** 12
putative nuclear encoded protein Method: similarity and extension
HCP00304_1 **Mass:** 9136 **Score:** 24 **Expect:** 28 **Matches:** 5
putative nuclear encoded protein Method: similarity and extension
HCP13194_1 **Mass:** 5695 **Score:** 20 **Expect:** 67 **Matches:** 3
putative nuclear encoded protein Method: similarity and extension

---

2. HCP00091_1 **Mass:** 32524 **Score:** 38 **Expect:** 1.1 **Matches:** 10
putative nuclear encoded protein Method: similarity and extension

---

Search Parameters

Type of search : Peptide Mass Fingerprint
Enzyme : Trypsin
Variable modifications : Carbamidomethyl (C),Glu->pyro-Glu (N-term E),Oxidation (M)
Mass values : Monoisotopic
Protein Mass : Unrestricted
Peptide Mass Tolerance : ± 1.2 Da
Peptide Charge State : 1+
Max Missed Cleavages : 1
Number of queries : 31

Protein View

Match to: **HCP00006_1** Score: **68** Expect: **0.0011**
putative nuclear encoded protein Method: similarity and extension

Nominal mass (M_r): **59610**; Calculated pI value: **6.67**
NCBI BLAST search of HCP00006_1 against nr
Unformatted sequence string for pasting into other applications

Variable modifications: Carbamidomethyl (C),Glu->pyro-Glu (N-term E),Oxidation (M)
Cleavage by Trypsin: cuts C-term side of KR unless next residue is P
Number of mass values searched: **31**
Number of mass values matched: **18**

Sequence Coverage: **29%**

Matched peptides shown in **Bold Red**

```
  1 MLSNLARTSG RMAFIRGISS AQMDAHAQVI DDQKPMEEQS NPSFFKMVDY
 51 YFDKGASVIE PKLVEEMKSN VMSTKDKKNL VSGILKAIKP VNKVLYITFP
101 IRRDNGEFEV IEAWRAQHSE HRTPTKGGIR YSMDVCEDEV KALSALMTYK
151 CAAVDVPFGG AKGGVKIDPK QYTDYEIEKI TRRIAIEFAK KGFLGPGVDV
201 PAPDMGTGER EMGWIADTYA QTIGHLDRDA SACITGKPIV AGGIHGRVSA
251 TGRGVWKGLE VFTKEPEYMN KVGLSLGLEG KTIIIQGFGN VGLHTMRYLH
301 RAGAKVIGVQ EWDCAVFNPD GIHPKELEDW RDENGTIKNF PKAKNFEPFA
351 ELMYEPCDIF VPAACEKAIH KENANRIQAK IIAEAANGPT TPAADKILLE
401 RGNCLIIPDM FINSGGVTVS YFEWLKNLNH VSYGRLSFKY EEDSNRMLLQ
451 SVQDSLEKAL NKEAPVHPND EFTARIAGAS EKDIVHSGLE YTMTRSGEAI
501 IRTARKYNLG LDIRTAAYAN SIEKVYNTYR TAGFTFT
```

Start - End	Observed	Mr(expt)	Mr(calc)	Delta	Miss	Sequence
94 - 102	1122.05	1121.04	1120.66	0.37	0	K.VLYITFPIR.R
127 - 141	1773.40	1772.39	1772.77	-0.38	1	K.GGIRYSMDVCEDEVK.A Carbamidomethyl (C); Oxidation (M)
142 - 150	1013.74	1012.74	1012.53	0.21	0	K.ALSALMTYK.C Oxidation (M)
171 - 179	1189.04	1188.03	1187.53	0.50	0	K.QYTDYEIEK.I
183 - 190	947.76	946.76	946.56	0.20	1	R.RIAIEFAK.K
184 - 191	919.71	918.70	918.55	0.15	1	R.IAIEFAKK.G
229 - 247	1880.42	1879.41	1878.97	0.44	0	R.DASACITGKPIVAGGIHGR.V Carbamidomethyl (C)
265 - 271	926.59	925.58	925.39	0.20	0	K.EPEYMNK.V Oxidation (M)
272 - 281	972.79	971.78	971.57	0.22	0	K.VGLSLGLEGK.T
427 - 435	1059.74	1058.73	1058.53	0.20	0	K.NLNHVSYGR.L
440 - 446	912.57	911.56	911.36	0.20	0	K.YEEDSNR.M
447 - 458	1407.16	1406.15	1405.71	0.44	0	R.MLLQSVQDSLEK.A Oxidation (M)
463 - 475	1465.12	1464.12	1463.68	0.44	0	K.EAPVHPNDEFTAR.I Glu->pyro-Glu (N-term E)
463 - 475	1483.14	1482.13	1481.69	0.44	0	K.EAPVHPNDEFTAR.I
483 - 495	1538.18	1537.17	1536.72	0.45	0	K.DIVHSGLEYTMTR.S Oxidation (M)
506 - 514	1091.85	1090.85	1090.61	0.23	1	R.KYNLGLDIR.T
507 - 514	963.73	962.72	962.52	0.20	0	K.YNLGLDIR.T
515 - 524	1067.75	1066.74	1066.53	0.21	0	R.TAAYANSIEK.V

No match to: 906.62, 938.67, 954.49, 957.88, 976.77, 994.75, 1045.78, 1081.72, 1089.75, 1106.82, 1139.93, 1248.13, 1350.13

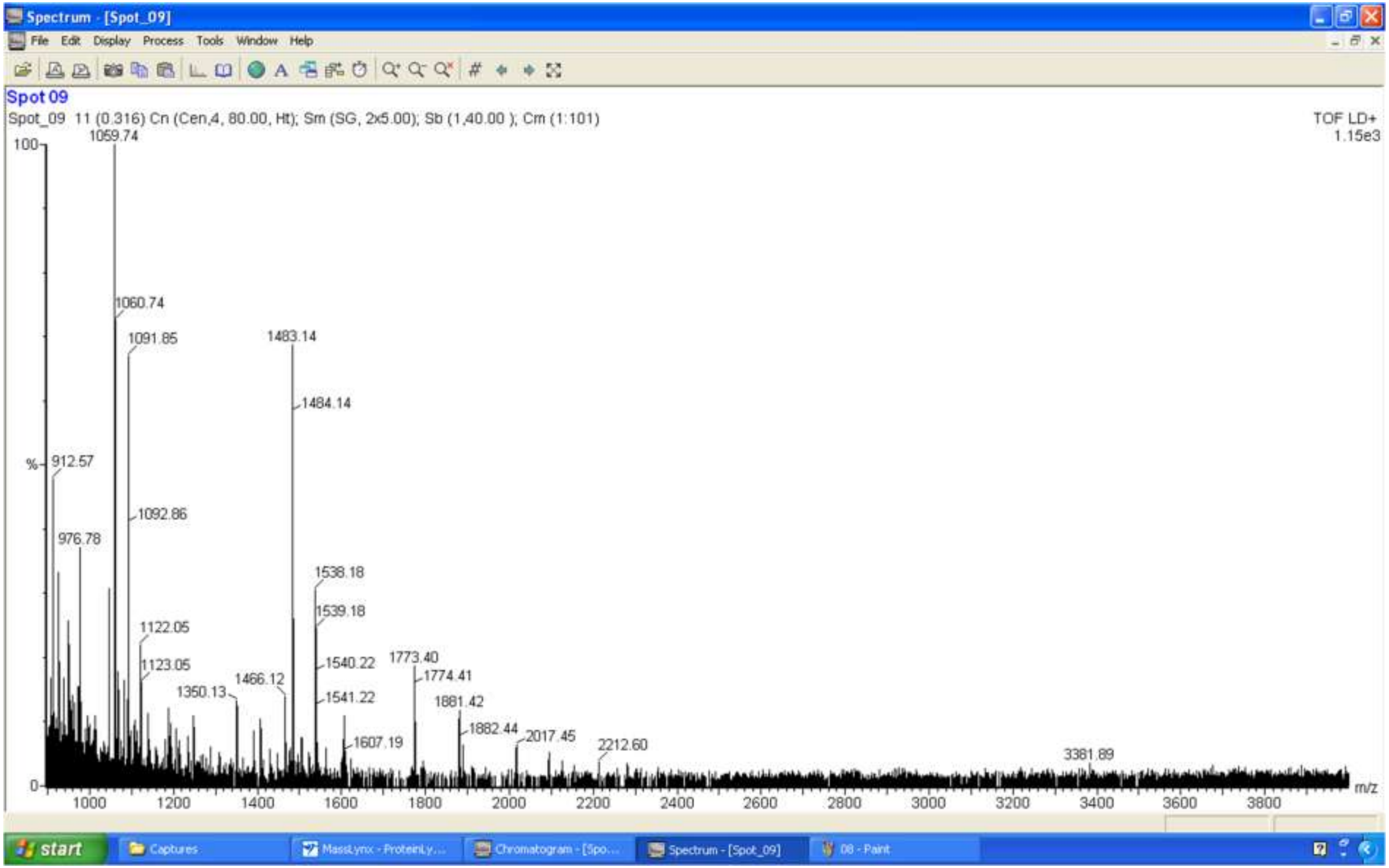
Spectrum - [Spot_09]
File Edit Display Process Tools Window Help
Spot 09
Spot_09 11 (0.316) Cn (Cen,4, 80.00, Ht); Sm (SG, 2x5.00); Sb (1,40.00); Cm (1:101)
TOF LD+
1.15e3
100
%
0
1059.74
1060.74
1091.85
1483.14
1484.14
912.57
1092.86
976.78
1538.18
1539.18
1122.05
1123.05
1540.22
1773.40
1774.41
1466.12
1350.13
1541.22
1881.42
1882.44
2017.45
2212.60
1607.19
3381.89
1000 1200 1400 1600 1800 2000 2200 2400 2600 2800 3000 3200 3400 3600 3800
m/z
start
Captures
MassLynx - ProteinLy...
Chromatogram - [Spo...
Spectrum - [Spot_09]
08 - Paint

Figure S1.07

Mascot Search Results Spot 10

```
User             : Paul Millares
Email            : paul.millares@gmail.com
Search title     : Spot 10
Database         : Haemonchus 210108 (6387 sequences; 918038 residues)
Timestamp        : 1 Aug 2011 at 10:33:21 GMT
Top Score        : 64 for HCP00006_1, putative nuclear encoded protein  Method: similarity and extension
```

Mascot Score Histogram

Protein score is -10*Log(P), where P is the probability that the observed match is a random event.

Protein scores greater than 51 are significant (p<0.05).

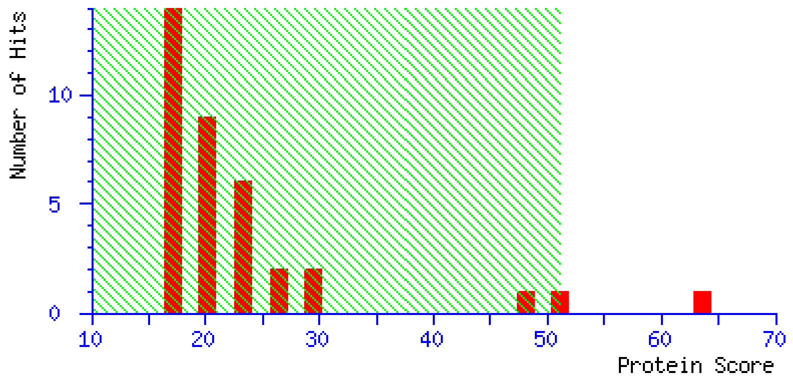

Concise Protein Summary Report

1. HCP00006_1 **Mass:** 59610 **Score:** **64** **Expect:** 0.0029 **Matches:** 14
   putative nuclear encoded protein Method: similarity and extension
   HCP00006_2 **Mass:** 59610 **Score:** **64** **Expect:** 0.0029 **Matches:** 14
   putative nuclear encoded protein Method: similarity and extension
   HCP10211_1 **Mass:** 18016 **Score:** 23 **Expect:** 29 **Matches:** 4
   putative nuclear encoded protein Method: ESTScan

HCP10651_1 **Mass:** 7078 **Score:** 21 **Expect:** 52 **Matches:** 3
putative nuclear encoded protein Method: Longest ORF
HCP09613_1 **Mass:** 26598 **Score:** 19 **Expect:** 80 **Matches:** 6
putative nuclear encoded protein Method: similarity and extension

---

2. HCP08936_1 **Mass:** 22506 **Score:** 52 **Expect:** 0.037 **Matches:** 9
putative nuclear encoded protein Method: similarity and extension
HCP02052_1 **Mass:** 5211 **Score:** 19 **Expect:** 90 **Matches:** 4
putative nuclear encoded protein Method: ESTScan
HCP04919_1 **Mass:** 7576 **Score:** 18 **Expect:** 99 **Matches:** 3
putative nuclear encoded protein Method: Longest ORF

---

3. HCP00006_3 **Mass:** 59543 **Score:** 48 **Expect:** 0.092 **Matches:** 12
putative nuclear encoded protein Method: similarity and extension

---

Search Parameters

Type of search : Peptide Mass Fingerprint
Enzyme : Trypsin
Variable modifications : Carbamidomethyl (C),Glu->pyro-Glu (N-term E),Oxidation (M)
Mass values : Monoisotopic
Protein Mass : Unrestricted
Peptide Mass Tolerance : ± 1.2 Da
Peptide Charge State : 1+
Max Missed Cleavages : 1
Number of queries : 23

Protein View

Match to: **HCP00006_1** Score: **64** Expect: **0.0029**
putative nuclear encoded protein Method: similarity and extension

Nominal mass (M_r): **59610**; Calculated pI value: **6.67**
NCBI BLAST search of HCP00006_1 against nr
Unformatted sequence string for pasting into other applications

Variable modifications: Carbamidomethyl (C),Glu->pyro-Glu (N-term E),Oxidation (M)
Cleavage by Trypsin: cuts C-term side of KR unless next residue is P
Number of mass values searched: **23**
Number of mass values matched: **14**
Sequence Coverage: **29%**

Matched peptides shown in **Bold Red**

1 MLSNLARTSG RMAFIRGISS AQMDAHAQVI DDQKPMEEQS NPSFFKMVDY
51 YFDKGASVIE PK**LVEEMKSN** **VMSTK**DKKNL VSGILKAIKP VNK**VLYITFP**
101 **IR**R**DNGEFEV** **IEAWR**AQHSE HRTPTK**GGIR** **YSMDVCEDEV** **KALSALMTYK**
151 CAAVDVPFGG AKGGVKIDPK QYTDYEIEKI TRRIAIEFAK **KGFLGPGVDV**
201 **PAPDMGTGER** EMGWIADTYA QTIGHLDR**DA** **SACITGKPIV** **AGGIHGR**VSA
251 TGRGVWKGLE VFTKEPEYMN KVGLSLGLEG K**TIIIQGFGN** **VGLHTMR**YLH
301 RAGAK**VIGVQ** **EWDCAVFNPD** **GIHPK**ELEDW RDENGTIKNF PKAKNFEPFA
351 ELMYEPCDIF VPAACEKAIH KENANRIQAK IIAEAANGPT TPAADKILLE
401 RGNCLIIPDM FINSGGVTVS YFEWLKNLNH VSYGRLSFKY EEDSNRMLLQ
451 SVQDSLEKAL NK**EAPVHPND** **EFTAR**IAGAS EK**DIVHSGLE** **YTMTR**SGEAI
501 IRTARKYNLG LDIRTAAYAN SIEKVYNTYR TAGFTFT

Start - End	Observed	Mr(expt)	Mr(calc)	Delta	Miss	Sequence
63 - 75	1496.06	1495.05	1494.74	0.31	1	K.LVEEMKSNVMSTK.D
94 - 102	1121.03	1120.02	1120.66	-0.64	0	K.VLYITFPIR.R
104 - 115	1464.02	1463.01	1463.67	-0.66	0	R.DNGEFEVIEAWR.A
127 - 141	1757.45	1756.44	1756.78	-0.33	1	K.GGIRYSMDVCEDEVK.A Carbamidomethyl (C)
131 - 141	1334.29	1333.28	1332.52	0.76	0	R.YSMDVCEDEVK.A Oxidation (M)
131 - 150	2296.45	2295.44	2295.05	0.40	1	R.YSMDVCEDEVKALSALMTYK.C
131 - 150	2312.50	2311.49	2311.04	0.45	1	R.YSMDVCEDEVKALSALMTYK.C Oxidation (M)
191 - 210	2016.37	2015.36	2014.98	0.38	1	K.KGFLGPGVDVPAPDMGTGER.E Oxidation (M)
192 - 210	1887.32	1886.31	1886.88	-0.57	0	K.GFLGPGVDVPAPDMGTGER.E Oxidation (M)
229 - 247	1880.37	1879.36	1878.97	0.39	0	R.DASACITGKPIVAGGIHGR.V Carbamidomethyl (C)
282 - 297	1772.34	1771.33	1771.94	-0.61	0	K.TIIIQGFGNVGLHTMR.Y Oxidation (M)
306 - 325	2280.44	2279.43	2280.10	-0.67	0	K.VIGVQEWDCAVFNPDGIHPK.E Carbamidomethyl (C)
463 - 475	1482.05	1481.04	1481.69	-0.65	0	K.EAPVHPNDEFTAR.I
483 - 495	1537.06	1536.05	1536.72	-0.67	0	K.DIVHSGLEYTMTR.S Oxidation (M)

No match to: 951.45, 994.33, 1029.49, 1208.81, 1224.56, 1435.58, 1448.06, 1732.50, 2282.49

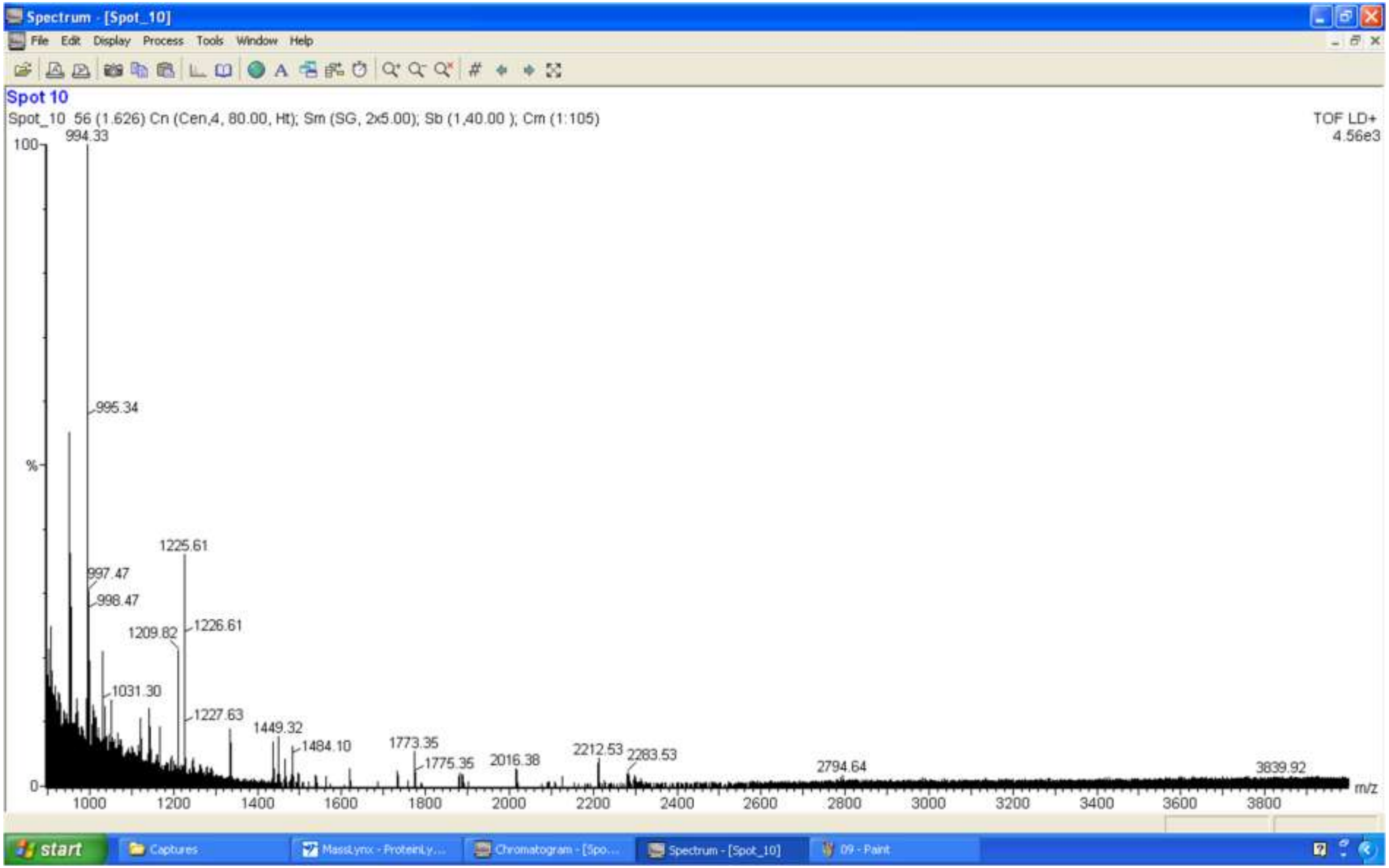
Spectrum - [Spot_10]
File Edit Display Process Tools Window Help
Spot 10
Spot_10 56 (1.626) Cn (Cen,4, 80.00, Ht); Sm (SG, 2x5.00); Sb (1,40.00); Cm (1:105)
TOF LD+
4.56e3
100
%
0
994.33
995.34
997.47
998.47
1031.30
1209.82
1225.61
1226.61
1227.63
1449.32
1484.10
1773.35
1775.35
2016.38
2212.53
2283.53
2794.64
3839.92
1000
1200
1400
1600
1800
2000
2200
2400
2600
2800
3000
3200
3400
3600
3800
m/z
start
Captures
Spectrum - [Spot_10]
09 - Paint

Figure S1.08

Mascot Search Results Spot 11

```
User            : Paul Millares
Email           : paul.millares@gmail.com
Search title    : Spot 11
Database        : Haemonchus 210108 (6387 sequences; 918038 residues)
Timestamp       : 1 Aug 2011 at 10:33:48 GMT
Top Score       : 78 for HCP00006_1, putative nuclear encoded protein  Method: similarity and extension
```

Mascot Score Histogram

Protein score is -10*Log(P), where P is the probability that the observed match is a random event.

Protein scores greater than 51 are significant (p<0.05).

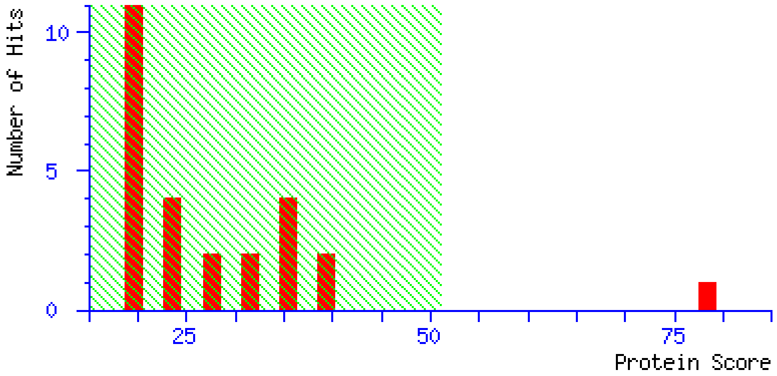

Concise Protein Summary Report

1. HCP00006_1 **Mass:** 59610 **Score:** 78 **Expect:** 9.2e-005 **Matches:** 15

putative nuclear encoded protein Method: similarity and extension

HCP00006_2 **Mass:** 59610 **Score:** 78 **Expect:** 9.2e-005 **Matches:** 15

putative nuclear encoded protein Method: similarity and extension

HCP00006_3 **Mass:** 59543 **Score:** 40 **Expect:** 0.6 **Matches:** 11

putative nuclear encoded protein Method: similarity and extension

HCP01433_1 **Mass:** 18575 **Score:** 29 **Expect:** 7.3 **Matches:** 5
putative nuclear encoded protein Method: similarity and extension
HCP07252_1 **Mass:** 8658 **Score:** 23 **Expect:** 32 **Matches:** 4
putative nuclear encoded protein Method: ESTScan
HCP02433_2 **Mass:** 8440 **Score:** 23 **Expect:** 33 **Matches:** 3
putative nuclear encoded protein Method: ESTScan
HCP12930_1 **Mass:** 9977 **Score:** 21 **Expect:** 52 **Matches:** 3
putative nuclear encoded protein Method: similarity and extension
HCP00304_1 **Mass:** 9136 **Score:** 21 **Expect:** 57 **Matches:** 4
putative nuclear encoded protein Method: similarity and extension
HCP07860_1 **Mass:** 19788 **Score:** 20 **Expect:** 67 **Matches:** 4
putative nuclear encoded protein Method: ESTScan
HCP05064_1 **Mass:** 19948 **Score:** 20 **Expect:** 67 **Matches:** 4
putative nuclear encoded protein Method: ESTScan
HCP02533_1 **Mass:** 10316 **Score:** 20 **Expect:** 70 **Matches:** 3
putative nuclear encoded protein Method: Longest ORF

---

2. HCP00560_1 **Mass:** 24138 **Score:** 40 **Expect:** 0.6 **Matches:** 8
putative nuclear encoded protein Method: similarity and extension

---

Search Parameters

Type of search : Peptide Mass Fingerprint
Enzyme : Trypsin
Variable modifications : Carbamidomethyl (C),Glu->pyro-Glu (N-term E),Oxidation (M)
Mass values : Monoisotopic
Protein Mass : Unrestricted
Peptide Mass Tolerance : ± 1.2 Da
Peptide Charge State : 1+
Max Missed Cleavages : 1
Number of queries : 20

Protein View

Match to: **HCP00006_1** Score: **78** Expect: **9.2e-005**
putative nuclear encoded protein Method: similarity and extension

Nominal mass (M_r): **59610**; Calculated pI value: **6.67**
NCBI BLAST search of HCP00006_1 against nr
Unformatted sequence string for pasting into other applications

Variable modifications: Carbamidomethyl (C),Glu->pyro-Glu (N-term E),Oxidation (M)
Cleavage by Trypsin: cuts C-term side of KR unless next residue is P
Number of mass values searched: **20**
Number of mass values matched: **15**
Sequence Coverage: **29%**

Matched peptides shown in **Bold Red**

1 MLSNLARTSG RMAFIRGISS AQMDAHAQVI DDQKPMEEQS NPSFFKMVDY
51 YFDKGASVIE PKLVEEMKSN VMSTKDKKNL VSGILKAIKP VNK**VLYITFP**
101 **IR**RDNGEFEV IEAWRAQHSE HRTPTKGGIR YSMDVCEDEV K**ALSALMTYK**
151 CAAVDVPFGG AKGGVKIDPK QYTDYEIEKI TR**RIAIEFAK** K**GFLGPGVDV**
201 **PAPDMGTGER** EMGWIADTYA QTIGHLDR**DA** **SACITGKPIV** **AGGIHGR**VSA
251 TGRGVWKGLE VFTK**EPEYMN** **KVGLSLGLEG** **KTIIIQGFGN** **VGLHTMR**YLH
301 RAGAKVIGVQ EWDCAVFNPD GIHPKELEDW R**DENGTIKNF** **PK**AKNFEPFA
351 ELMYEPCDIF VPAACEKAIH KENANRIQAK IIAEAANGPT TPAADKILLE
401 RGNCLIIPDM FINSGGVTVS YFEWLK**NLNH** **VSYGR**LSFK**Y** **EEDSNR**MLLQ
451 SVQDSLEKAL NK**EAPVHPND** **EFTAR**IAGAS EK**DIVHSGLE** **YTMTR**SGEAI
501 IRTAR**KYNLG** **LDIR**TAAYAN SIEKVYNTYR TAGFTFT

Start - End	Observed	Mr(expt)	Mr(calc)	Delta	Miss	Sequence	
94 - 102	1121.96	1120.96	1120.66	0.29	0	K.VLYITFPIR.R	
142 - 150	1013.62	1012.61	1012.53	0.09	0	K.ALSALMTYK.C	Oxidation (M)
183 - 190	947.71	946.70	946.56	0.14	1	R.RIAIEFAK.K	
192 - 210	1888.19	1887.18	1886.88	0.30	0	K.GFLGPGVDVPAPDMGTGER.E	Oxidation (M)
229 - 247	1880.29	1879.28	1878.97	0.30	0	R.DASACITGKPIVAGGIHGR.V	Carbamidomethyl (C)
265 - 271	926.54	925.53	925.39	0.15	0	K.EPEYMNK.V	Oxidation (M)
272 - 281	972.69	971.68	971.57	0.11	0	K.VGLSLGLEGK.T	
282 - 297	1773.29	1772.28	1771.94	0.34	0	K.TIIIQGFGNVGLHTMR.Y	Oxidation (M)
332 - 342	1263.01	1262.00	1261.63	0.37	1	R.DENGTIKNFPK.A	
427 - 435	1059.65	1058.65	1058.53	0.12	0	K.NLNHVSYGR.L	
440 - 446	912.49	911.48	911.36	0.12	0	K.YEEDSNR.M	
463 - 475	1465.01	1464.00	1463.68	0.32	0	K.EAPVHPNDEFTAR.I	Glu->pyro-Glu (N-term E)
463 - 475	1483.02	1482.01	1481.69	0.32	0	K.EAPVHPNDEFTAR.I	
483 - 495	1538.07	1537.06	1536.72	0.34	0	K.DIVHSGLEYTMTR.S	Oxidation (M)

506 - 514 1091.76 1090.76 1090.61 0.14 1 R.KYNLGLDIR.T

No match to: 938.42, 976.71, 1119.76, 1135.93, 1447.98

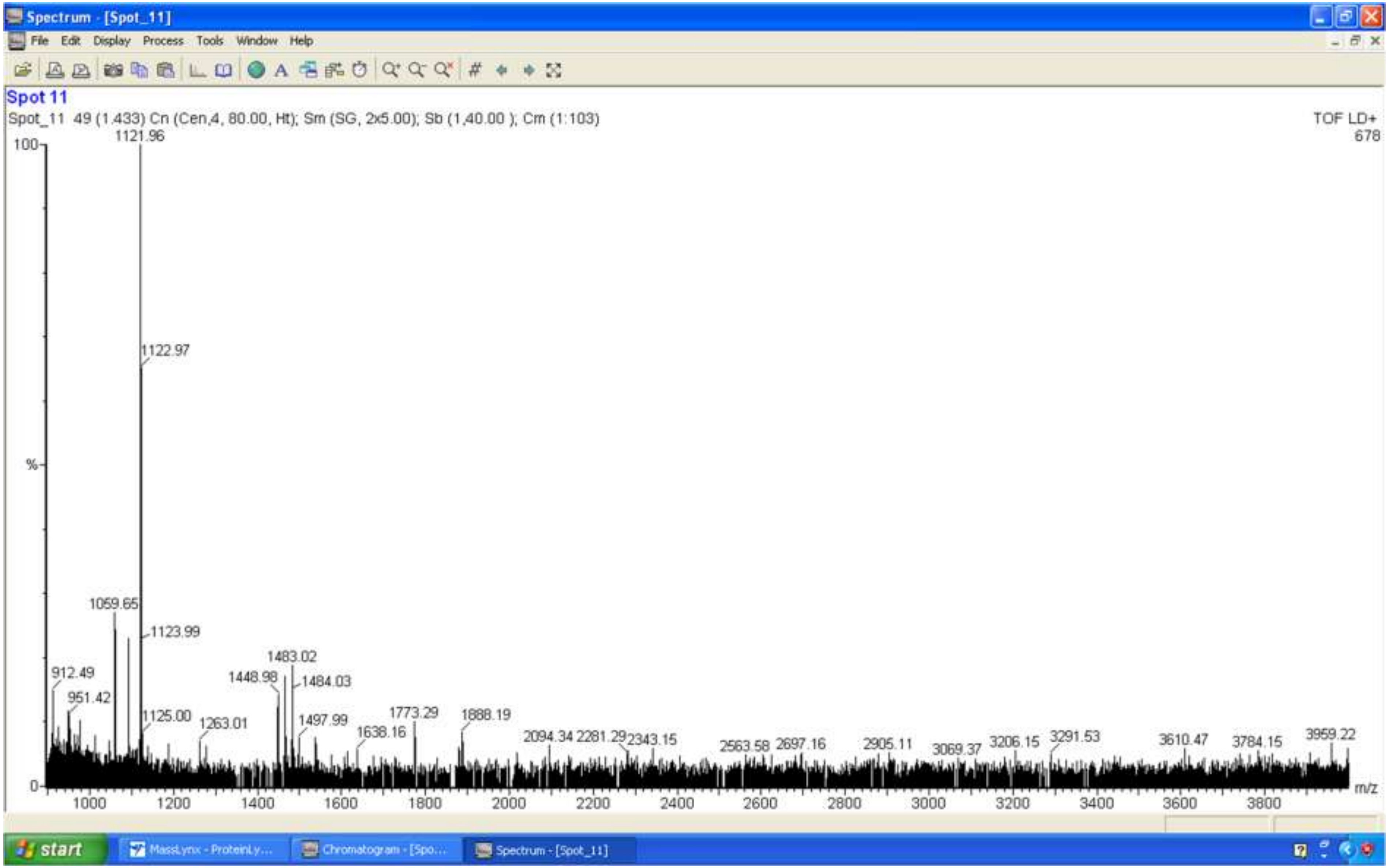

Figure S1.09

Mascot Search Results Spot 12

```
User            : Paul Millares
Email           : paul.millares@gmail.com
Search title    : Spot 12
Database        : Haemonchus 210108 (6387 sequences; 918038 residues)
Timestamp       : 1 Aug 2011 at 10:34:29 GMT
Top Score       : 108 for HCP00006_1, putative nuclear encoded protein  Method: similarity and extension
```

Mascot Score Histogram

Protein score is -10*Log(P), where P is the probability that the observed match is a random event.

Protein scores greater than 51 are significant (p<0.05).

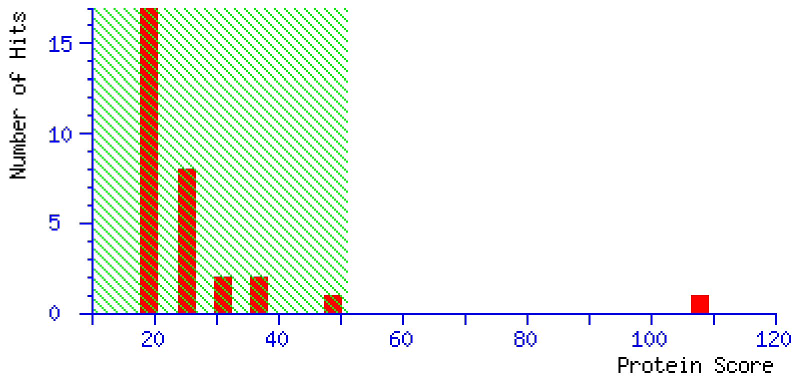

Concise Protein Summary Report

1. HCP00006_1 **Mass:** 59610 **Score:** **108** **Expect:** 1e-007 **Matches:** 21
   putative nuclear encoded protein Method: similarity and extension
   HCP00006_2 **Mass:** 59610 **Score:** **108** **Expect:** 1e-007 **Matches:** 21
   putative nuclear encoded protein Method: similarity and extension
   HCP00006_3 **Mass:** 59543 **Score:** 49 **Expect:** 0.088 **Matches:** 15
   putative nuclear encoded protein Method: similarity and extension

HCP12588_1 **Mass:** 13370 **Score:** 34 **Expect:** 2.8 **Matches:** 6
putative nuclear encoded protein Method: Longest ORF
HCP09394_1 **Mass:** 5341 **Score:** 27 **Expect:** 12 **Matches:** 6
putative nuclear encoded protein Method: similarity and extension
HCP00304_1 **Mass:** 9136 **Score:** 26 **Expect:** 15 **Matches:** 6
putative nuclear encoded protein Method: similarity and extension
HCP04919_2 **Mass:** 8297 **Score:** 23 **Expect:** 34 **Matches:** 4
putative nuclear encoded protein Method: Longest ORF
HCP13194_1 **Mass:** 5695 **Score:** 21 **Expect:** 46 **Matches:** 5
putative nuclear encoded protein Method: similarity and extension
HCP04735_2 **Mass:** 15549 **Score:** 21 **Expect:** 53 **Matches:** 6
putative nuclear encoded protein Method: similarity and extension
HCP10912_1 **Mass:** 21000 **Score:** 21 **Expect:** 56 **Matches:** 6
putative nuclear encoded protein Method: similarity and extension
HCP02811_2 **Mass:** 7716 **Score:** 21 **Expect:** 57 **Matches:** 5
putative nuclear encoded protein Method: similarity and extension
HCP00670_1 **Mass:** 7717 **Score:** 21 **Expect:** 57 **Matches:** 5
putative nuclear encoded protein Method: similarity and extension
HCP06413_2 **Mass:** 21950 **Score:** 20 **Expect:** 65 **Matches:** 5
putative nuclear encoded protein Method: similarity and extension
HCP06730_1 **Mass:** 6331 **Score:** 20 **Expect:** 67 **Matches:** 3
putative nuclear encoded protein Method: Longest ORF
HCP00193_3 **Mass:** 15017 **Score:** 20 **Expect:** 72 **Matches:** 4
putative nuclear encoded protein Method: ESTScan

---

2. HCP00406_1 **Mass:** 32394 **Score:** 49 **Expect:** 0.086 **Matches:** 11
putative nuclear encoded protein Method: similarity and extension
HCP00406_2 **Mass:** 18965 **Score:** 22 **Expect:** 40 **Matches:** 5
putative nuclear encoded protein Method: similarity and extension

---

Search Parameters

Type of search : Peptide Mass Fingerprint
Enzyme : Trypsin
Variable modifications : Carbamidomethyl (C),Glu->pyro-Glu (N-term E),Oxidation (M)
Mass values : Monoisotopic
Protein Mass : Unrestricted
Peptide Mass Tolerance : ± 1.2 Da
Peptide Charge State : 1+
Max Missed Cleavages : 1
Number of queries : 27

Protein View

Match to: **HCP00006_1** Score: **108** Expect: **1e-007**
putative nuclear encoded protein Method: similarity and extension

Nominal mass (M_r): **59610**; Calculated pI value: **6.67**
NCBI BLAST search of HCP00006_1 against nr
Unformatted sequence string for pasting into other applications

Variable modifications: Carbamidomethyl (C),Glu->pyro-Glu (N-term E),Oxidation (M)
Cleavage by Trypsin: cuts C-term side of KR unless next residue is P
Number of mass values searched: **27**
Number of mass values matched: **21**
Sequence Coverage: **38%**

Matched peptides shown in **Bold Red**

```
  1 MLSNLARTSG RMAFIRGISS AQMDAHAQVI DDQKPMEEQS NPSFFKMVDY
 51 YFDKGASVIE PKLVEEMKSN VMSTKDKKNL VSGILKAIKP VNKVLYITFP
101 IRRDNGEFEV IEAWRAQHSE HRTPTKGGIR YSMDVCEDEV KALSALMTYK
151 CAAVDVPFGG AKGGVKIDPK QYTDYEIEKI TRRIAIEFAK KGFLGPGVDV
201 PAPDMGTGER EMGWIADTYA QTIGHLDRDA SACITGKPIV AGGIHGRVSA
251 TGRGVWKGLE VFTKEPEYMN KVGLSLGLEG KTIIIQGFGN VGLHTMRYLH
301 RAGAKVIGVQ EWDCAVFNPD GIHPKELEDW RDENGTIKNF PKAKNFEPFA
351 ELMYEPCDIF VPAACEKAIH KENANRIQAK IIAEAANGPT TPAADKILLE
401 RGNCLIIPDM FINSGGVTVS YFEWLKNLNH VSYGRLSFKY EEDSNRMLLQ
451 SVQDSLEKAL NKEAPVHPND EFTARIAGAS EKDIVHSGLE YTMTRSGEAI
501 IRTARKYNLG LDIRTAAYAN SIEKVYNTYR TAGFTFT
```

Start - End	Observed	Mr(expt)	Mr(calc)	Delta	Miss	Sequence
63 - 75	1496.94	1495.93	1494.74	1.19	1	K.LVEEMKSNVMSTK.D

94 - 102	1121.93	1120.93	1120.66	0.26	0	K.VLYITFPIR.R
104 - 115	1463.92	1462.92	1463.67	-0.75	0	R.DNGEFEVIEAWR.A
142 - 150	1013.61	1012.60	1012.53	0.07	0	K.ALSALMTYK.C Oxidation (M)
151 - 162	1191.91	1190.90	1190.58	0.33	0	K.CAAVDVPFGGAK.G Carbamidomethyl (C)
183 - 190	947.65	946.65	946.56	0.09	1	R.RIAIEFAK.K
191 - 210	2016.22	2015.21	2014.98	0.23	1	K.KGFLGPGVDVPAPDMGTGER.E Oxidation (M)
229 - 247	1880.22	1879.21	1878.97	0.24	0	R.DASACITGKPIVAGGIHGR.V Carbamidomethyl (C)
265 - 271	926.45	925.44	925.39	0.05	0	K.EPEYMNK.V Oxidation (M)
272 - 281	972.65	971.64	971.57	0.08	0	K.VGLSLGLEGK.T
282 - 297	1773.21	1772.20	1771.94	0.26	0	K.TIIIQGFGNVGLHTMR.Y Oxidation (M)
427 - 435	1059.61	1058.60	1058.53	0.07	0	K.NLNHVSYGR.L
440 - 446	912.44	911.44	911.36	0.07	0	K.YEEDSNR.M
447 - 458	1406.98	1405.97	1405.71	0.26	0	R.MLLQSVQDSLEK.A Oxidation (M)
463 - 475	1464.96	1463.95	1463.68	0.27	0	K.EAPVHPNDEFTAR.I Glu->pyro-Glu (N-term E)
463 - 475	1482.97	1481.96	1481.69	0.27	0	K.EAPVHPNDEFTAR.I
483 - 495	1522.04	1521.03	1520.73	0.30	0	K.DIVHSGLEYTMTR.S
483 - 495	1537.99	1536.98	1536.72	0.26	0	K.DIVHSGLEYTMTR.S Oxidation (M)
506 - 514	1091.72	1090.72	1090.61	0.10	1	R.KYNLGLDIR.T
507 - 514	963.61	962.60	962.52	0.08	0	K.YNLGLDIR.T
515 - 524	1068.61	1067.61	1066.53	1.08	0	R.TAAYANSIEK.V

No match to: 976.66, 1045.63, 1174.89, 1447.93, 1479.92, 1889.13

Figure S1.10

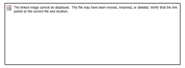

Mascot Search Results Spot 12

User : Paul Millares
Email : paul.millares@gmail.com
Search title : Spot 12
MS data file : Spot 12.txt
Database : NCBInr 20110729 (14821581 sequences; 5074018658 residues)
Timestamp : 3 Aug 2011 at 21:29:41 GMT
Top Score : 120 for gi|253721985, glutamate dehydrogenase [Haemonchus contortus]

Mascot Score Histogram

Protein score is -10*Log(P), where P is the probability that the observed match is a random event.

Protein scores greater than 84 are significant (p<0.05).

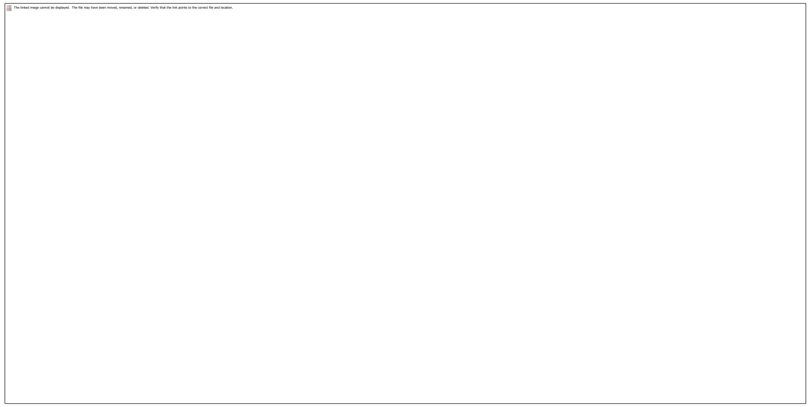

Concise Protein Summary Report

1. gi|253721985 **Mass:** 59100 **Score:** **120** **Expect:** 1.5e-05 **Matches:** 22
glutamate dehydrogenase [Haemonchus contortus]
gi|253721983 **Mass:** 59045 **Score:** **108** **Expect:** 0.00023 **Matches:** 21
glutamate dehydrogenase [Haemonchus contortus]
gi|160933224 **Mass:** 10020 **Score:** 54 **Expect:** 63 **Matches:** 8

hypothetical protein CLOLEP_02070 [Clostridium leptum DSM 753]

---

2. gi|66768323 **Mass:** 72543 **Score:** 67 **Expect:** 2.8 **Matches:** 18
phage-related integrase [Xanthomonas campestris pv. campestris str. 8004]
gi|21231557 **Mass:** 72561 **Score:** 66 **Expect:** 3.4 **Matches:** 18
phage-related integrase [Xanthomonas campestris pv. campestris str. ATCC 33913]
gi|323474786 **Mass:** 9152 **Score:** 55 **Expect:** 43 **Matches:** 7
integrase [Sulfolobus islandicus REY15A]
gi|323477513 **Mass:** 9516 **Score:** 55 **Expect:** 48 **Matches:** 7
integrase [Sulfolobus islandicus HVE10/4]
gi|188991462 **Mass:** 72513 **Score:** 51 **Expect:** 1.3e+02 **Matches:** 16
phage-related integrase [Xanthomonas campestris pv. campestris str. B100]

Search Parameters

Type of search : Peptide Mass Fingerprint
Enzyme : Trypsin
Variable modifications : Carbamidomethyl (C),Glu->pyro-Glu (N-term E),Oxidation (M)
Mass values : Monoisotopic
Protein Mass : Unrestricted
Peptide Mass Tolerance : ± 1.2 Da
Peptide Charge State : 1+
Max Missed Cleavages : 1
Number of queries : 27

Protein View

Match to: **gi|253721985** Score: **120** Expect: **1.5e-05**
glutamate dehydrogenase [Haemonchus contortus]
Found in search of Spot 12.txt

Nominal mass (M_r): **59100**; Calculated pI value: **6.67**
NCBI BLAST search of gi|253721985 against nr
Unformatted sequence string for pasting into other applications

Taxonomy: Haemonchus contortus

Variable modifications: Carbamidomethyl (C),Glu->pyro-Glu (N-term E),Oxidation (M)
Cleavage by Trypsin: cuts C-term side of KR unless next residue is P
Number of mass values searched: **27**
Number of mass values matched: **22**
Sequence Coverage: **40%**

Matched peptides shown in **Bold Red**

```
  1 MVSNLARTSG RMAFIRGISS AQMDAHAQVI DDQKPMEEQS NPSFFKMVDY
 51 YFDKGASVIE PKLVEEMKSN VMSTKDKKNL VSGILKAIKP VNKVLYITFP
101 IRRDNGEFEV IEAWRAQHSE HRTPTKGGIR YSMDVCEDEV KALSALMTYK
151 CAAVDVPFGG AKGGVKIDPK QYTDYEIEKI TRRIAIEFAK KGFLGPGVDV
201 PAPDMGTGER EMGWIADTYA QTIGHLDRDA SACITGKPIV AGGIHGRVSA
251 TGRGVWKGLE VFTKEPEYMN KVGLSLGLEG KTIIIQGFGN VGLHTMRYLH
301 RAGAKVIGVQ EWDCAVFNPD GIHPKELEDW RDENGTIKNF PKAKNFEPFA
351 ELMYEPCDIF VPAACEKAIH KENANRIQAK IIAEAANGPT TPAADKILLE
401 RGNCLIIPDM FINSGGVTVS YFEWLKNLNH VSYGRLSFKY EEDSNRMLLQ
451 SVQDSLEKAL NKEAPVHPND EFTARIAGAS EKDIVHSGLE YTMTRSGEAI
501 IRTARKYNLG LDIRTAAYAN SIEKVYNTYR TAG
```

Start - End	Observed	Mr(expt)	Mr(calc)	Delta	Miss	Sequence
1 - 11	1191.9103	1190.9030	1190.6190	0.2841	1	-.MVSNLARTSGR.M
63 - 75	1496.9417	1495.9344	1494.7422	1.1923	1	K.LVEEMKSNVMSTK.D
94 - 102	1121.9324	1120.9251	1120.6645	0.2607	0	K.VLYITFPIR.R
104 - 115	1463.9226	1462.9153	1463.6681	-0.7528	0	R.DNGEFEVIEAWR.A
142 - 150	1013.6068	1012.5995	1012.5263	0.0732	0	K.ALSALMTYK.C Oxidation (M)
183 - 190	947.6532	946.6459	946.5600	0.0860	1	R.RIAIEFAK.K
191 - 210	2016.2173	2015.2100	2014.9783	0.2318	1	K.KGFLGPGVDVPAPDMGTGER.E Oxidation (M)
229 - 247	1880.2167	1879.2094	1878.9734	0.2360	0	R.DASACITGKPIVAGGIHGR.V Carbamidomethyl (C)
265 - 271	926.4462	925.4389	925.3851	0.0538	0	K.EPEYMNK.V Oxidation (M)
272 - 281	972.6520	971.6447	971.5651	0.0796	0	K.VGLSLGLEGK.T
282 - 297	1773.2059	1772.1986	1771.9403	0.2583	0	K.TIIIQGFGNVGLHTMR.Y Oxidation (M)
427 - 435	1059.6073	1058.6000	1058.5257	0.0743	0	K.NLNHVSYGR.L
440 - 446	912.4442	911.4369	911.3621	0.0749	0	K.YEEDSNR.M
447 - 458	1406.9818	1405.9745	1405.7123	0.2623	0	R.MLLQSVQDSLEK.A Oxidation (M)
463 - 475	1464.9581	1463.9508	1463.6793	0.2715	0	K.EAPVHPNDEFTAR.I Glu->pyro-Glu (N-term E)
463 - 475	1482.9707	1481.9634	1481.6899	0.2735	0	K.EAPVHPNDEFTAR.I
483 - 495	1522.0380	1521.0307	1520.7293	0.3014	0	K.DIVHSGLEYTMTR.S
483 - 495	1537.9899	1536.9826	1536.7242	0.2584	0	K.DIVHSGLEYTMTR.S Oxidation (M)
506 - 514	1091.7228	1090.7155	1090.6135	0.1021	1	R.KYNLGLDIR.T
507 - 514	963.6067	962.5994	962.5185	0.0809	0	K.YNLGLDIR.T
515 - 524	1068.6140	1067.6067	1066.5294	1.0773	0	R.TAAYANSIEK.V

525 - 533 1045.6277 1044.6204 1043.5036 1.1168 1 K.VYNTYRTAG.-

No match to: 976.6599, 1174.8909, 1447.9296, 1479.9229, 1889.1295

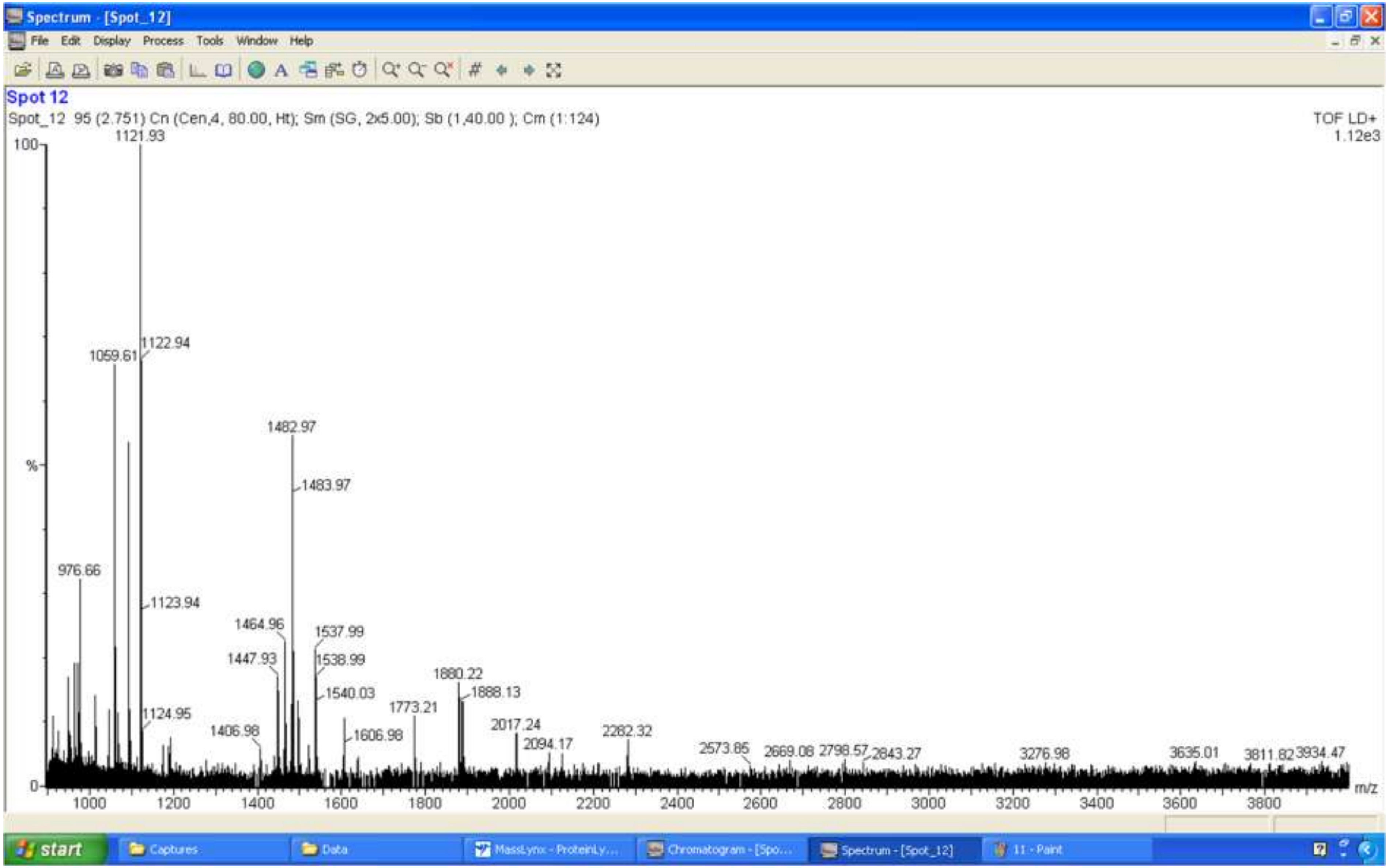

Figure S1.11

Mascot Search Results Spot 13

```
User            : Paul Millares
Email           : paul.millares@gmail.com
Search title    : Spot 13
Database        : Haemonchus 210108 (6387 sequences; 918038 residues)
Timestamp       : 1 Aug 2011 at 10:35:01 GMT
Top Score       : 71 for HCP00006_1, putative nuclear encoded protein  Method: similarity and extension
```

Mascot Score Histogram

Protein score is -10*Log(P), where P is the probability that the observed match is a random event.

Protein scores greater than 51 are significant (p<0.05).

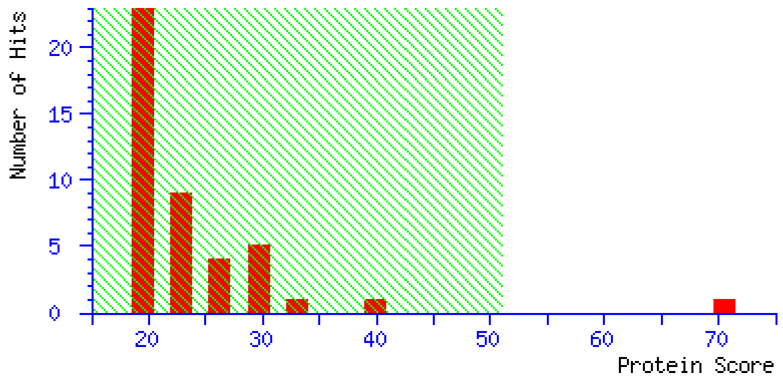

Concise Protein Summary Report

1. HCP00006_1 **Mass:** 59610 **Score:** 71 **Expect:** 0.00057 **Matches:** 36

   putative nuclear encoded protein Method: similarity and extension

   HCP00006_2 **Mass:** 59610 **Score:** 71 **Expect:** 0.00057 **Matches:** 36

   putative nuclear encoded protein Method: similarity and extension

---

2. HCP00006_3 **Mass:** 59543 **Score:** 40 **Expect:** 0.68 **Matches:** 27

putative nuclear encoded protein Method: similarity and extension

Search Parameters

Type of search : Peptide Mass Fingerprint
Enzyme : Trypsin
Variable modifications : Carbamidomethyl (C),Glu->pyro-Glu (N-term E),Oxidation (M)
Mass values : Monoisotopic
Protein Mass : Unrestricted
Peptide Mass Tolerance : ± 1.2 Da
Peptide Charge State : 1+
Max Missed Cleavages : 1
Number of queries : 82

Protein View

Match to: **HCP00006_1** Score: **71** Expect: **0.00057**
putative nuclear encoded protein Method: similarity and extension

Nominal mass (M_r): **59610**; Calculated pI value: **6.67**
NCBI BLAST search of HCP00006_1 against nr
Unformatted sequence string for pasting into other applications

Variable modifications: Carbamidomethyl (C),Glu->pyro-Glu (N-term E),Oxidation (M)
Cleavage by Trypsin: cuts C-term side of KR unless next residue is P
Number of mass values searched: **82**
Number of mass values matched: **36**
Sequence Coverage: **60%**

Matched peptides shown in **Bold Red**

```
  1 MLSNLARTSG RMAFIRGISS AQMDAHAQVI DDQKPMEEQS NPSFFKMVDY
 51 YFDKGASVIE PKLVEEMKSN VMSTKDKKNL VSGILKAIKP VNKVLYITFP
101 IRRDNGEFEV IEAWRAQHSE HRTPTKGGIR YSMDVCEDEV KALSALMTYK
151 CAAVDVPFGG AKGGVKIDPK QYTDYEIEKI TRRIAIEFAK KGFLGPGVDV
201 PAPDMGTGER EMGWIADTYA QTIGHLDRDA SACITGKPIV AGGIHGRVSA
251 TGRGVWKGLE VFTKEPEYMN KVGLSLGLEG KTIIIQGFGN VGLHTMRYLH
301 RAGAKVIGVQ EWDCAVFNPD GIHPKELEDW RDENGTIKNF PKAKNFEPFA
351 ELMYEPCDIF VPAACEKAIH KENANRIQAK IIAEAANGPT TPAADKILLE
401 RGNCLIIPDM FINSGGVTVS YFEWLKNLNH VSYGRLSFKY EEDSNRMLLQ
451 SVQDSLEKAL NKEAPVHPND EFTARIAGAS EKDIVHSGLE YTMTRSGEAI
```

501 IRTAR**KYNLG LDIRTAAYAN SIEK**VYNTYR TAGFTFT

Start - End	Observed	Mr(expt)	Mr(calc)	Delta	Miss	Sequence
47 - 54	1081.60	1080.59	1079.46	1.13	0	K.MVDYYFDK.G
63 - 75	1495.00	1493.99	1494.74	-0.75	1	K.LVEEMKSNVMSTK.D
87 - 102	1872.08	1871.07	1871.14	-0.07	1	K.AIKPVNKVLYITFPIR.R
94 - 102	1120.57	1119.57	1120.66	-1.10	0	K.VLYITFPIR.R
94 - 102	1121.93	1120.92	1120.66	0.26	0	K.VLYITFPIR.R
94 - 103	1278.12	1277.11	1276.77	0.34	1	K.VLYITFPIRR.D
104 - 115	1463.96	1462.95	1463.67	-0.72	0	R.DNGEFEVIEAWR.A
131 - 150	2296.33	2295.32	2295.05	0.27	1	R.YSMDVCEDEVKALSALMTYK.C
142 - 150	1012.46	1011.45	1012.53	-1.07	0	K.ALSALMTYK.C Oxidation (M)
142 - 150	1013.59	1012.58	1012.53	0.05	0	K.ALSALMTYK.C Oxidation (M)
151 - 166	1476.10	1475.09	1474.76	0.33	1	K.CAAVDVPFGGAKGGVK.I
171 - 179	1188.91	1187.90	1187.53	0.36	0	K.QYTDYEIEK.I
183 - 190	947.65	946.65	946.56	0.09	1	R.RIAIEFAK.K
191 - 210	2016.26	2015.25	2014.98	0.27	1	K.KGFLGPGVDVPAPDMGTGER.E Oxidation (M)
211 - 228	2093.25	2092.24	2091.97	0.27	0	R.EMGWIADTYAQTIGHLDR.D Oxidation (M)
229 - 247	1880.23	1879.22	1878.97	0.25	0	R.DASACITGKPIVAGGIHGR.V Carbamidomethyl (C)
265 - 271	909.34	908.33	907.37	0.96	0	K.EPEYMNK.V Glu->pyro-Glu (N-term E); Oxidation (M)
265 - 271	926.37	925.37	925.39	-0.02	0	K.EPEYMNK.V Oxidation (M)
272 - 281	972.64	971.64	971.57	0.07	0	K.VGLSLGLEGK.T
282 - 297	1773.25	1772.25	1771.94	0.31	0	K.TIIIQGFGNVGLHTMR.Y Oxidation (M)
282 - 301	2326.20	2325.19	2325.25	-0.06	1	K.TIIIQGFGNVGLHTMRYLHR.A
306 - 325	2281.31	2280.30	2280.10	0.20	0	K.VIGVQEWDCAVFNPDGIHPK.E Carbamidomethyl (C)
326 - 338	1585.97	1584.97	1585.74	-0.77	1	K.ELEDWRDENGTIK.N Glu->pyro-Glu (N-term E)
326 - 338	1605.04	1604.04	1603.75	0.29	1	K.ELEDWRDENGTIK.N
345 - 367	2736.42	2735.41	2735.20	0.21	0	K.NFEPFAELMYEPCDIFVPAACEK.A Carbamidomethyl (C); Oxidation (M)
427 - 435	1059.61	1058.61	1058.53	0.08	0	K.NLNHVSYGR.L
440 - 446	912.44	911.43	911.36	0.07	0	K.YEEDSNR.M
447 - 458	1406.99	1405.98	1405.71	0.27	0	R.MLLQSVQDSLEK.A Oxidation (M)
463 - 475	1464.97	1463.96	1463.68	0.28	0	K.EAPVHPNDEFTAR.I Glu->pyro-Glu (N-term E)
463 - 475	1482.98	1481.97	1481.69	0.28	0	K.EAPVHPNDEFTAR.I
483 - 495	1521.98	1520.97	1520.73	0.24	0	K.DIVHSGLEYTMTR.S
483 - 495	1538.01	1537.00	1536.72	0.28	0	K.DIVHSGLEYTMTR.S Oxidation (M)
506 - 514	1090.63	1089.62	1090.61	-1.00	1	R.KYNLGLDIR.T
506 - 514	1091.72	1090.71	1090.61	0.10	1	R.KYNLGLDIR.T
507 - 514	963.61	962.61	962.52	0.09	0	K.YNLGLDIR.T
515 - 524	1067.58	1066.57	1066.53	0.04	0	R.TAAYANSIEK.V

No match to: 897.47, 902.40, 921.41, 933.54, 935.46, 942.45, 951.37, 958.38, 976.66, 983.43, 984.43, 990.58, 992.41, 994.65, 1001.40, 1005.53, 1033.60, 1047.49, 1050.40, 1105.51, 1107.72, 1113.81, 1142.74, 1143.95, 1165.95, 1266.99, 1363.77, 1447.94,

1479.95, 1496.96, 1563.05, 1708.96, 1714.03, 1732.66, 1821.10, 1849.82, 1889.16, 1926.57, 1966.06, 2036.36, 2064.15, 2125.21, 2313.29, 2505.29, 3229.09, 3912.70

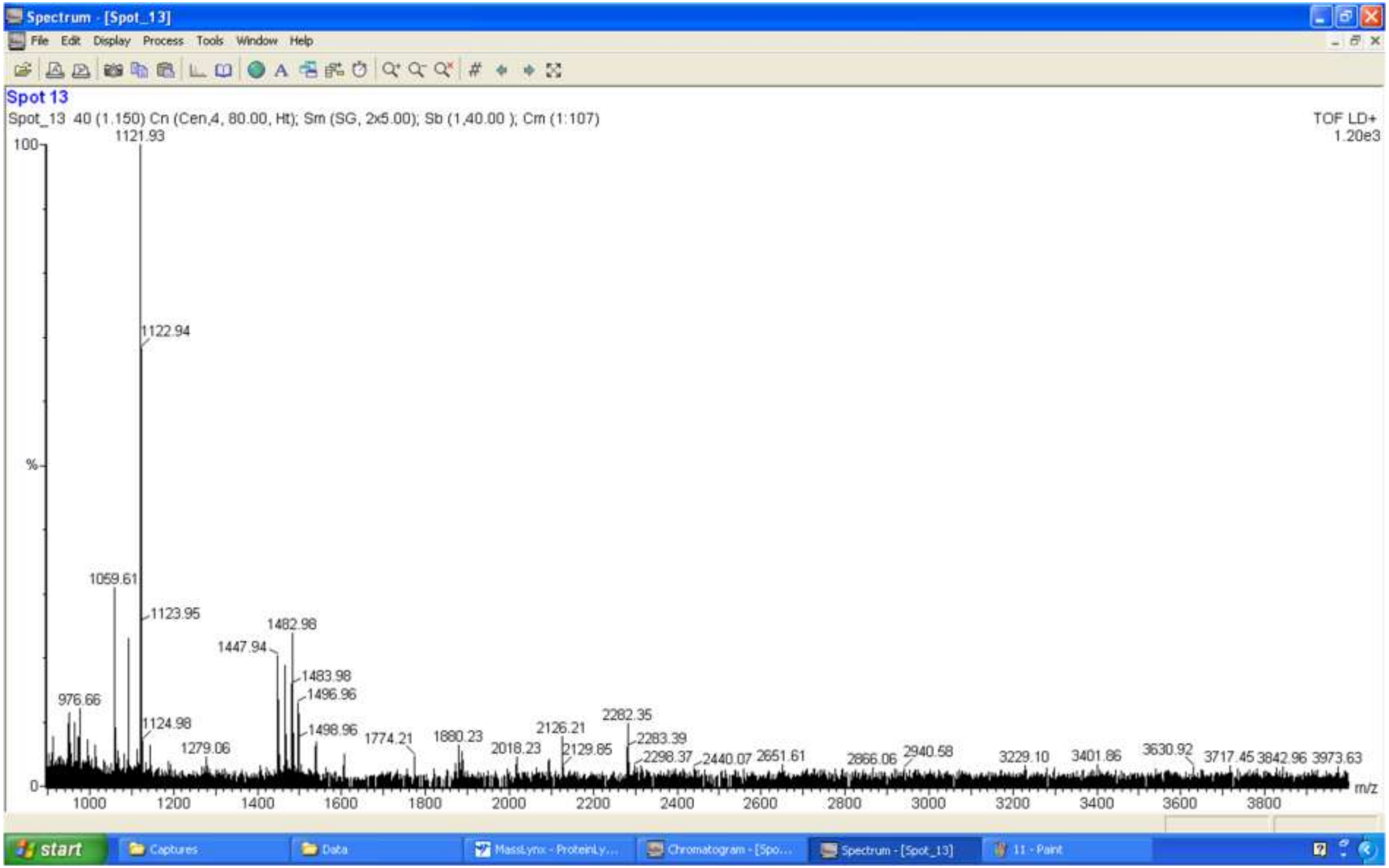

Figure S1.12

Mascot Search Results Spot 14

```
User            : Paul Millares
Email           : paul.millares@gmail.com
Search title    : Spot 14
Database        : Haemonchus 210108 (6387 sequences; 918038 residues)
Timestamp       : 1 Aug 2011 at 10:35:27 GMT
Top Score       : 56 for HCP00006_1, putative nuclear encoded protein  Method: similarity and extension
```

Mascot Score Histogram

Protein score is -10*Log(P), where P is the probability that the observed match is a random event.

Protein scores greater than 51 are significant (p<0.05).

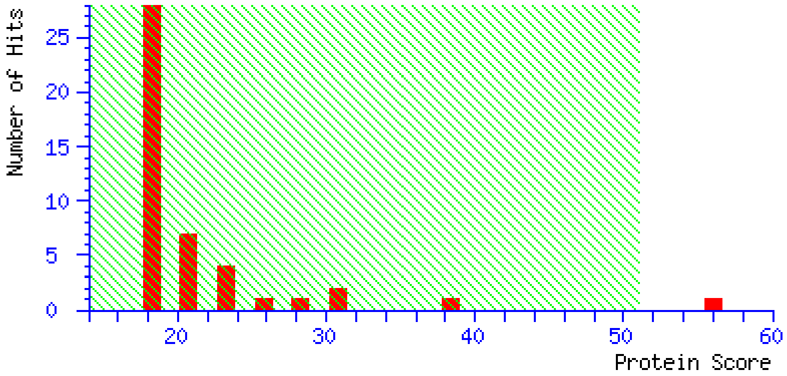

Concise Protein Summary Report

1. HCP00006_1 **Mass:** 59610 **Score:** **56** **Expect:** 0.016 **Matches:** 17
   putative nuclear encoded protein Method: similarity and extension
   HCP00006_2 **Mass:** 59610 **Score:** **56** **Expect:** 0.016 **Matches:** 17
   putative nuclear encoded protein Method: similarity and extension
   HCP11302_1 **Mass:** 14460 **Score:** 19 **Expect:** 73 **Matches:** 4
   putative nuclear encoded protein Method: ESTScan

2. HCP00006_3 **Mass:** 59543 **Score:** 39 **Expect:** 0.8 **Matches:** 14
putative nuclear encoded protein Method: similarity and extension

Search Parameters

Type of search : Peptide Mass Fingerprint
Enzyme : Trypsin
Variable modifications : Carbamidomethyl (C),Glu->pyro-Glu (N-term E),Oxidation (M)
Mass values : Monoisotopic
Protein Mass : Unrestricted
Peptide Mass Tolerance : ± 1.2 Da
Peptide Charge State : 1+
Max Missed Cleavages : 1
Number of queries : 33

Protein View

Match to: **HCP00006_1** Score: **56** Expect: **0.016**
putative nuclear encoded protein Method: similarity and extension

Nominal mass (M_r): **59610;** Calculated pI value: **6.67**
NCBI BLAST search of HCP00006_1 against nr
Unformatted sequence string for pasting into other applications

Variable modifications: Carbamidomethyl (C),Glu->pyro-Glu (N-term E),Oxidation (M)
Cleavage by Trypsin: cuts C-term side of KR unless next residue is P
Number of mass values searched: **33**
Number of mass values matched: **17**
Sequence Coverage: **33%**

Matched peptides shown in **Bold Red**

```
  1 MLSNLARTSG RMAFIRGISS AQMDAHAQVI DDQKPMEEQS NPSFFKMVDY
 51 YFDKGASVIE PKLVEEMKSN VMSTKDKKNL VSGILKAIKP VNKVLYITFP
101 IRRDNGEFEV IEAWRAQHSE HRTPTKGGIR YSMDVCEDEV KALSALMTYK
151 CAAVDVPFGG AKGGVKIDPK QYTDYEIEKI TRRIAIEFAK KGFLGPGVDV
201 PAPDMGTGER EMGWIADTYA QTIGHLDRDA SACITGKPIV AGGIHGRVSA
251 TGRGVWKGLE VFTKEPEYMN KVGLSLGLEG KTIIIQGFGN VGLHTMRYLH
301 RAGAKVIGVQ EWDCAVFNPD GIHPKELEDW RDENGTIKNF PKAKNFEPFA
```

```
351 ELMYEPCDIF VPAACEKAIH KENANRIQAK IIAEAANGPT TPAADKILLE
401 RGNCLIIPDM FINSGGVTVS YFEWLKNLNH VSYGRLSFKY EEDSNRMLLQ
451 SVQDSLEKAL NKEAPVHPND EFTARIAGAS EKDIVHSGLE YTMTRSGEAI
501 IRTARKYNLG LDIRTAAYAN SIEKVYNTYR TAGFTFT
```

Start - End	Observed	Mr(expt)	Mr(calc)	Delta	Miss	Sequence
94 - 102	1121.92	1120.91	1120.66	0.25	0	K.VLYITFPIR.R
183 - 190	948.66	947.65	946.56	1.09	1	R.RIAIEFAK.K
191 - 210	1999.79	1998.79	1998.98	-0.20	1	K.KGFLGPGVDVPAPDMGTGER.E
192 - 210	1889.08	1888.08	1886.88	1.19	0	K.GFLGPGVDVPAPDMGTGER.E Oxidation (M)
211 - 228	2094.14	2093.14	2091.97	1.17	0	R.EMGWIADTYAQTIGHLDR.D Oxidation (M)
229 - 247	1880.23	1879.22	1878.97	0.25	0	R.DASACITGKPIVAGGIHGR.V Carbamidomethyl (C)
248 - 257	1060.13	1059.12	1059.58	-0.46	1	R.VSATGRGVWK.G
265 - 271	926.44	925.43	925.39	0.05	0	K.EPEYMNK.V Oxidation (M)
272 - 281	973.03	972.02	971.57	0.46	0	K.VGLSLGLEGK.T
282 - 297	1773.12	1772.11	1771.94	0.17	0	K.TIIIQGFGNVGLHTMR.Y Oxidation (M)
381 - 396	1539.01	1538.00	1538.79	-0.80	0	K.IIAEAANGPTTPAADK.I
402 - 426	2875.98	2874.97	2875.39	-0.42	0	R.GNCLIIPDMFINSGGVTVSYFEWLK.N Carbamidomethyl (C); Oxidation (M)
463 - 475	1464.96	1463.95	1463.68	0.27	0	K.EAPVHPNDEFTAR.I Glu->pyro-Glu (N-term E)
463 - 475	1481.84	1480.84	1481.69	-0.85	0	K.EAPVHPNDEFTAR.I
463 - 475	1482.96	1481.95	1481.69	0.26	0	K.EAPVHPNDEFTAR.I
506 - 514	1091.73	1090.72	1090.61	0.11	1	R.KYNLGLDIR.T
507 - 514	962.99	961.98	962.52	-0.54	0	K.YNLGLDIR.T

No match to: 897.11, 906.94, 917.05, 924.46, 924.98, 934.99, 954.32, 976.64, 977.65, 1082.19, 1447.93, 1553.44, 1860.56, 2116.10, 2814.02, 3550.83

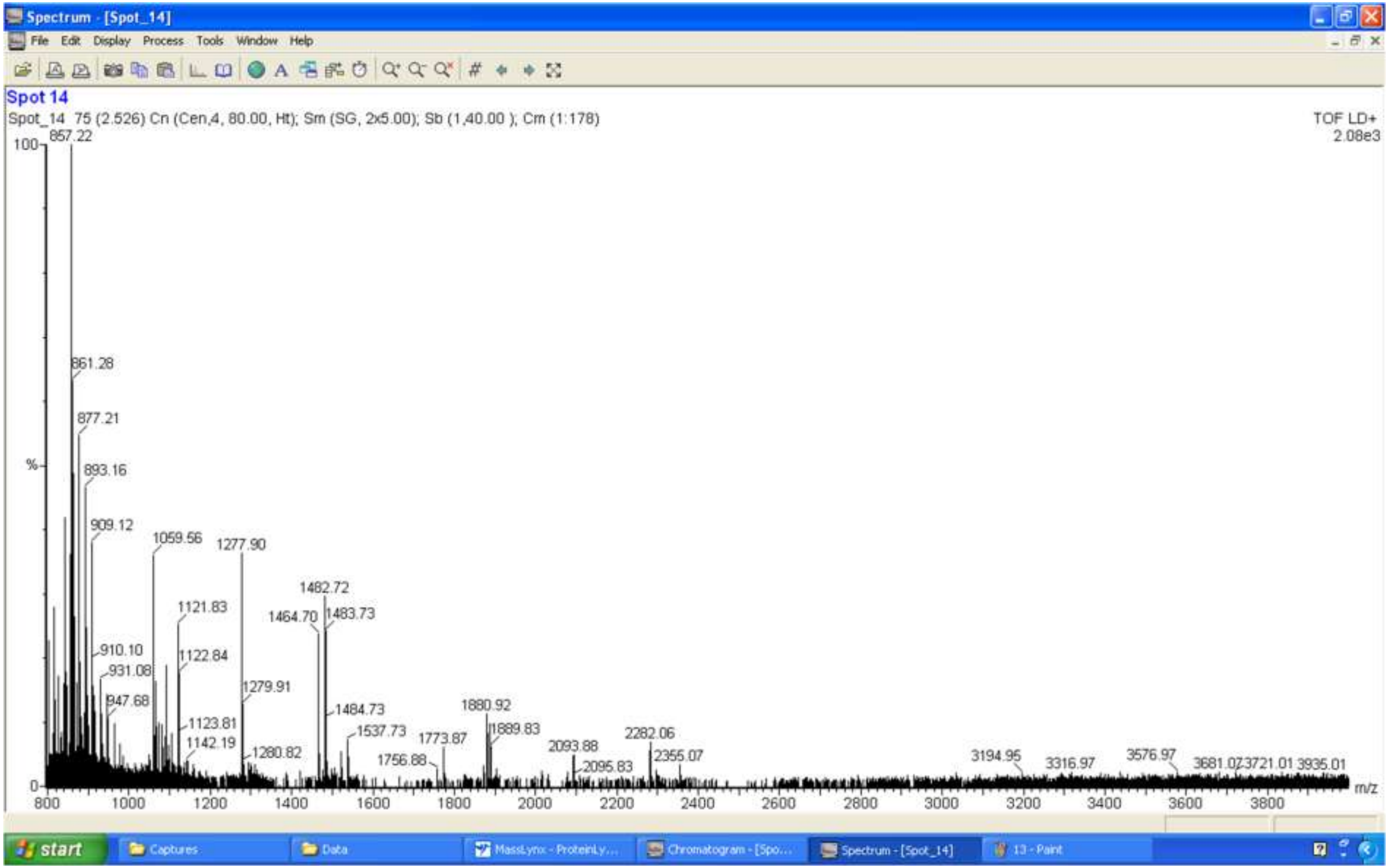
Spectrum - [Spot_14]
File Edit Display Process Tools Window Help
Spot 14
Spot_14 75 (2.526) Cn (Cen,4, 80.00, Ht); Sm (SG, 2x5.00); Sb (1,40.00); Cm (1:178)
TOF LD+
2.08e3
857.22
861.28
877.21
893.16
909.12
910.10
931.08
947.68
1059.56
1121.83
1122.84
1123.81
1142.19
1277.90
1279.91
1280.82
1464.70
1482.72
1483.73
1484.73
1537.73
1756.88
1773.87
1880.92
1889.83
2093.88
2095.83
2282.06
2355.07
3194.95
3316.97
3576.97
3681.07
3721.01
3935.01
m/z
%
start
Captures
Data
MassLynx - ProteinLy...
Chromatogram - [Spo...
Spectrum - [Spot_14]
13 - Paint

Figure S1.13

Mascot Search Results Spot 15

```
User            : Paul Millares
Email           : paul.millares@gmail.com
Search title    : Spot 15
Database        : Haemonchus 210108 (6387 sequences; 918038 residues)
Timestamp       : 1 Aug 2011 at 10:35:52 GMT
Top Score       : 74 for HCP00006_1, putative nuclear encoded protein  Method: similarity and extension
```

Mascot Score Histogram

Protein score is -10*Log(P), where P is the probability that the observed match is a random event.

Protein scores greater than 51 are significant (p<0.05).

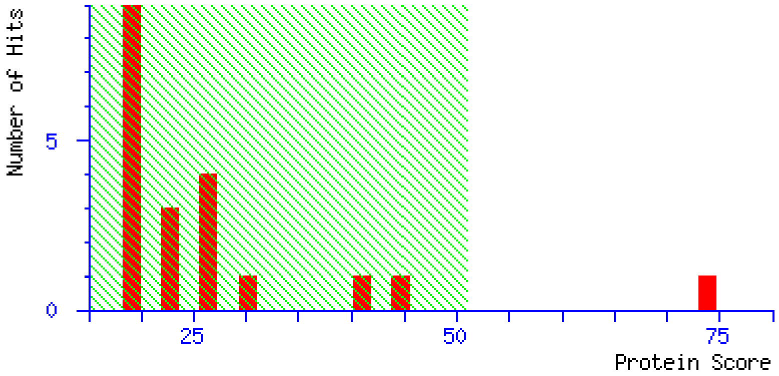

Concise Protein Summary Report

1. HCP00006_1 **Mass:** 59610 **Score:** 74 **Expect:** 0.00026 **Matches:** 13
   putative nuclear encoded protein Method: similarity and extension
   HCP00006_2 **Mass:** 59610 **Score:** 74 **Expect:** 0.00026 **Matches:** 13
   putative nuclear encoded protein Method: similarity and extension
   HCP04735_2 **Mass:** 15549 **Score:** 34 **Expect:** 2.3 **Matches:** 6
   putative nuclear encoded protein Method: similarity and extension

HCP04076_1 **Mass:** 12094 **Score:** 31 **Expect:** 5.3 **Matches:** 4
putative nuclear encoded protein Method: similarity and extension
HCP12588_1 **Mass:** 13370 **Score:** 27 **Expect:** 14 **Matches:** 4
putative nuclear encoded protein Method: Longest ORF
HCP07252_1 **Mass:** 8658 **Score:** 27 **Expect:** 14 **Matches:** 4
putative nuclear encoded protein Method: ESTScan
HCP01303_2 **Mass:** 28007 **Score:** 25 **Expect:** 18 **Matches:** 5
putative nuclear encoded protein Method: similarity and extension
HCP04735_1 **Mass:** 14659 **Score:** 24 **Expect:** 26 **Matches:** 5
putative nuclear encoded protein Method: similarity and extension
HCP00006_3 **Mass:** 59543 **Score:** 24 **Expect:** 26 **Matches:** 7
putative nuclear encoded protein Method: similarity and extension
HCP09103_1 **Mass:** 20368 **Score:** 23 **Expect:** 30 **Matches:** 6
putative nuclear encoded protein Method: ESTScan
HCP00560_1 **Mass:** 24138 **Score:** 23 **Expect:** 34 **Matches:** 5
putative nuclear encoded protein Method: similarity and extension
HCP00304_1 **Mass:** 9136 **Score:** 23 **Expect:** 35 **Matches:** 3
putative nuclear encoded protein Method: similarity and extension
HCP02815_1 **Mass:** 19621 **Score:** 23 **Expect:** 36 **Matches:** 4
putative nuclear encoded protein Method: similarity and extension
HCP06454_2 **Mass:** 8348 **Score:** 22 **Expect:** 38 **Matches:** 4
putative nuclear encoded protein Method: ESTScan
HCP06454_1 **Mass:** 8220 **Score:** 22 **Expect:** 38 **Matches:** 4
putative nuclear encoded protein Method: ESTScan
HCP03413_1 **Mass:** 16318 **Score:** 21 **Expect:** 47 **Matches:** 5
putative nuclear encoded protein Method: similarity and extension
HCP02193_2 **Mass:** 18532 **Score:** 20 **Expect:** 61 **Matches:** 4
putative nuclear encoded protein Method: similarity and extension
HCP07634_1 **Mass:** 18373 **Score:** 20 **Expect:** 64 **Matches:** 4
putative nuclear encoded protein Method: similarity and extension

HCP04691_3 **Mass:** 13606 **Score:** 19 **Expect:** 80 **Matches:** 3
putative nuclear encoded protein Method: ESTScan
HCP00414_2 **Mass:** 26641 **Score:** 19 **Expect:** 80 **Matches:** 5
putative nuclear encoded protein Method: similarity and extension
HCP00817_2 **Mass:** 26203 **Score:** 19 **Expect:** 84 **Matches:** 4
putative nuclear encoded protein Method: similarity and extension

---

2. HCP04665_1 **Mass:** 23510 **Score:** 46 **Expect:** 0.17 **Matches:** 8
putative nuclear encoded protein Method: ESTScan

Search Parameters

Type of search : Peptide Mass Fingerprint
Enzyme : Trypsin
Variable modifications : Carbamidomethyl (C),Glu->pyro-Glu (N-term E),Oxidation (M)
Mass values : Monoisotopic
Protein Mass : Unrestricted
Peptide Mass Tolerance : ± 1.2 Da
Peptide Charge State : 1+
Max Missed Cleavages : 1
Number of queries : 16

Protein View

Match to: **HCP00006_1** Score: **74** Expect: **0.00026**
putative nuclear encoded protein Method: similarity and extension

Nominal mass (M_r): **59610**; Calculated pI value: **6.67**
NCBI BLAST search of HCP00006_1 against nr
Unformatted sequence string for pasting into other applications

Variable modifications: Carbamidomethyl (C),Glu->pyro-Glu (N-term E),Oxidation (M)
Cleavage by Trypsin: cuts C-term side of KR unless next residue is P
Number of mass values searched: **16**
Number of mass values matched: **13**
Sequence Coverage: **21%**

Matched peptides shown in **Bold Red**

1 MLSNLARTSG RMAFIRGISS AQMDAHAQVI DDQKPMEEQS NPSFFKMVDY
51 YFDKGASVIE PKLVEEMKSN VMSTKDKKNL VSGILKAIKP VNK**VLYITFP**
101 **IR**RDNGEFEV IEAWRAQHSE HRTPTK**GGIR YSMDVCEDEV KALSALMTYK**
151 CAAVDVPFGG AKGGVKIDPK QYTDYEIEKI TR**RIAIEFAK** KGFLGPGVDV
201 PAPDMGTGER EMGWIADTYA QTIGHLDR**DA SACITGKPIV AGGIHGR**VSA
251 TGRGVWKGLE VFTK**EPEYMN K**VGLSLGLEG KTIIIQGFGN VGLHTMRYLH
301 RAGAKVIGVQ EWDCAVFNPD GIHPKELEDW RDENGTIKNF PKAKNFEPFA
351 ELMYEPCDIF VPAACEKAIH KENANRIQAK IIAEAANGPT TPAADKILLE
401 RGNCLIIPDM FINSGGVTVS YFEWLK**NLNH VSYGR**LSFK**Y EEDSNR**MLLQ
451 SVQDSLEKAL NK**EAPVHPND EFTAR**IAGAS EK**DIVHSGLE YTMTR**SGEAI
501 IRTAR**KYNLG LDIR**TAAYAN SIEKVYNTYR TAGFTFT

Start - End	Observed	Mr(expt)	Mr(calc)	Delta	Miss	Sequence	
94 - 102	1122.07	1121.06	1120.66	0.39	0	K.VLYITFPIR.R	
127 - 141	1773.44	1772.43	1772.77	-0.34	1	K.GGIRYSMDVCEDEVK.A	Carbamidomethyl (C); Oxidation (M)
142 - 150	996.53	995.53	996.53	-1.00	0	K.ALSALMTYK.C	
183 - 190	947.77	946.76	946.56	0.20	1	R.RIAIEFAK.K	
229 - 247	1880.43	1879.42	1878.97	0.44	0	R.DASACITGKPIVAGGIHGR.V	Carbamidomethyl (C)
265 - 271	907.50	906.50	907.37	-0.88	0	K.EPEYMNK.V	Glu->pyro-Glu (N-term E); Oxidation (M)
265 - 271	926.54	925.53	925.39	0.14	0	K.EPEYMNK.V	Oxidation (M)
427 - 435	1059.72	1058.71	1058.53	0.19	0	K.NLNHVSYGR.L	
440 - 446	912.58	911.57	911.36	0.21	0	K.YEEDSNR.M	
463 - 475	1483.13	1482.12	1481.69	0.43	0	K.EAPVHPNDEFTAR.I	
483 - 495	1538.16	1537.15	1536.72	0.43	0	K.DIVHSGLEYTMTR.S	Oxidation (M)
506 - 514	1091.85	1090.84	1090.61	0.23	1	R.KYNLGLDIR.T	
507 - 514	963.11	962.10	962.52	-0.42	0	K.YNLGLDIR.T	

No match to: 935.07, 951.51, 976.78

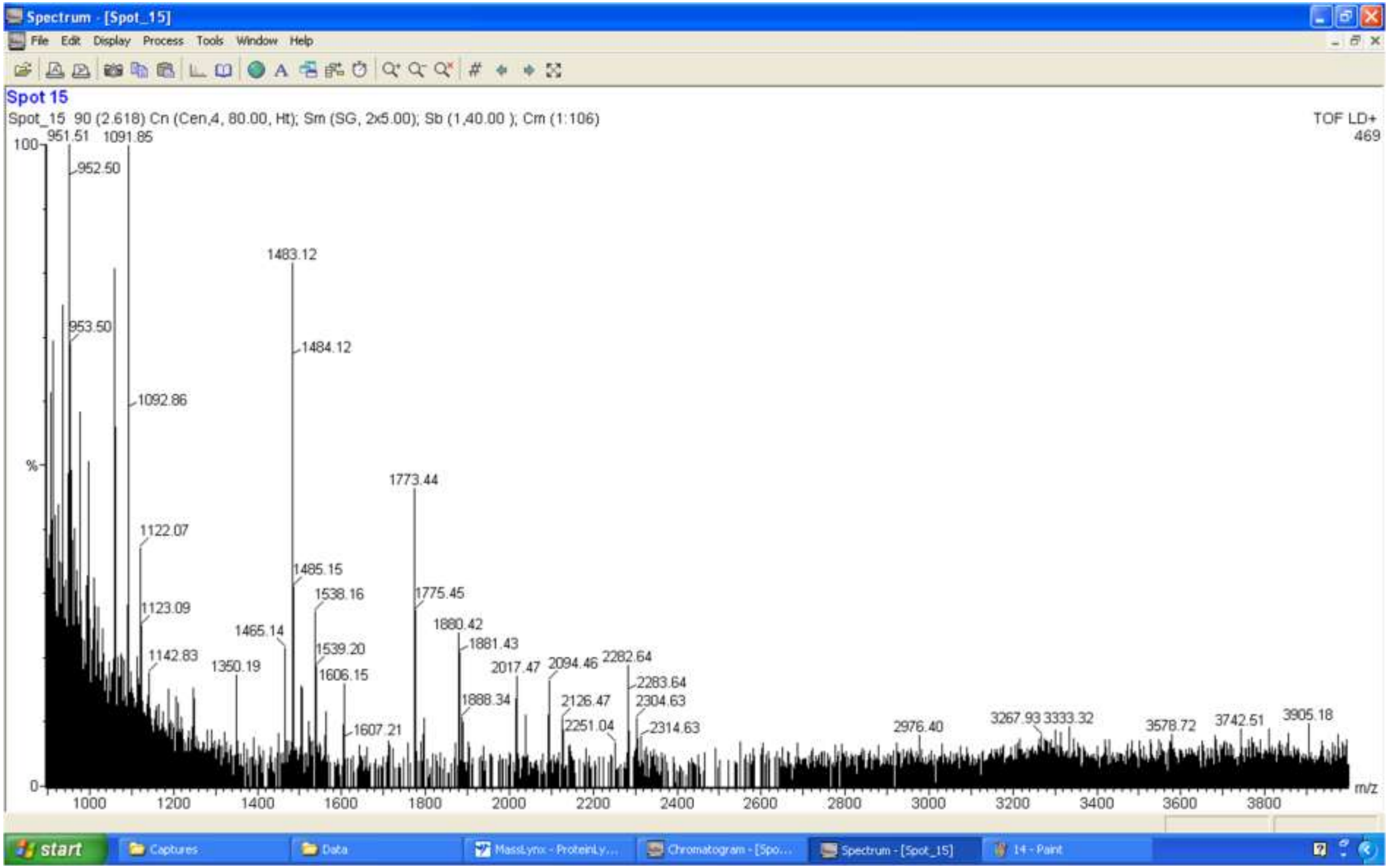
Spectrum - [Spot_15]
File Edit Display Process Tools Window Help
Spot 15
Spot_15 90 (2.618) Cn (Cen,4, 80.00, Ht); Sm (SG, 2x5.00); Sb (1,40.00); Cm (1:106)
TOF LD+
469
951.51
1091.85
952.50
953.50
1092.86
1122.07
1123.09
1142.83
1350.19
1465.14
1483.12
1484.12
1485.15
1538.16
1539.20
1606.15
1607.21
1773.44
1775.45
1880.42
1881.43
1888.34
2017.47
2094.46
2126.47
2251.04
2282.64
2283.64
2304.63
2314.63
2976.40
3267.93
3333.32
3578.72
3742.51
3905.18
100
%
0
1000
1200
1400
1600
1800
2000
2200
2400
2600
2800
3000
3200
3400
3600
3800
m/z
start
Captures
Data
MassLynx - ProteinLy...
Chromatogram - [Spo...
Spectrum - [Spot_15]
14 - Paint

Figure S1.14

Mascot Search Results Spot 17

```
User          : Paul Millares
Email         : paul.millares@gmail.com
Search title  : Spot 17
Database      : Haemonchus 210108 (6387 sequences; 918038 residues)
Timestamp     : 1 Aug 2011 at 10:36:16 GMT
Top Score     : 51 for HCP00006_1, putative nuclear encoded protein  Method: similarity and extension
```

Mascot Score Histogram

Protein score is -10*Log(P), where P is the probability that the observed match is a random event.

Protein scores greater than 51 are significant (p<0.05).

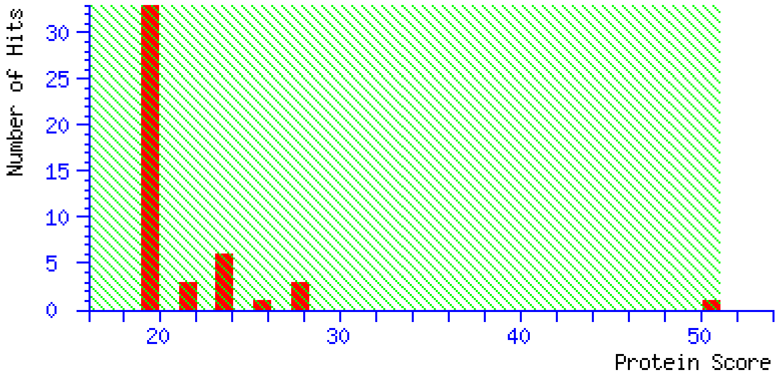

Concise Protein Summary Report

1. HCP00006_1 **Mass:** 59610 **Score:** 51 **Expect:** 0.056 **Matches:** 44

   putative nuclear encoded protein Method: similarity and extension

   HCP00006_2 **Mass:** 59610 **Score:** 51 **Expect:** 0.056 **Matches:** 44

   putative nuclear encoded protein Method: similarity and extension

Search Parameters

Type of search : Peptide Mass Fingerprint
Enzyme : Trypsin
Variable modifications : Carbamidomethyl (C),Glu->pyro-Glu (N-term E),Oxidation (M)
Mass values : Monoisotopic
Protein Mass : Unrestricted
Peptide Mass Tolerance : ± 1.2 Da
Peptide Charge State : 1+
Max Missed Cleavages : 1
Number of queries : 129

Protein View

Match to: **HCP00006_1** Score: **51** Expect: **0.056**
putative nuclear encoded protein Method: similarity and extension

Nominal mass (M_r): **59610**; Calculated pI value: **6.67**
NCBI BLAST search of HCP00006_1 against nr
Unformatted sequence string for pasting into other applications

Variable modifications: Carbamidomethyl (C),Glu->pyro-Glu (N-term E),Oxidation (M)
Cleavage by Trypsin: cuts C-term side of KR unless next residue is P
Number of mass values searched: **129**
Number of mass values matched: **44**
Sequence Coverage: **54%**

Matched peptides shown in **Bold Red**

```
  1 MLSNLARTSG RMAFIRGISS AQMDAHAQVI DDQKPMEEQS NPSFFKMVDY
 51 YFDKGASVIE PKLVEEMKSN VMSTKDKKNL VSGILKAIKP VNKVLYITFP
101 IRRDNGEFEV IEAWRAQHSE HRTPTKGGIR YSMDVCEDEV KALSALMTYK
151 CAAVDVPFGG AKGGVKIDPK QYTDYEIEKI TRRIAIEFAK KGFLGPGVDV
201 PAPDMGTGER EMGWIADTYA QTIGHLDRDA SACITGKPIV AGGIHGRVSA
251 TGRGVWKGLE VFTKEPEYMN KVGLSLGLEG KTIIIQGFGN VGLHTMRYLH
301 RAGAKVIGVQ EWDCAVFNPD GIHPKELEDW RDENGTIKNF PKAKNFEPFA
351 ELMYEPCDIF VPAACEKAIH KENANRIQAK IIAEAANGPT TPAADKILLE
401 RGNCLIIPDM FINSGGVTVS YFEWLKNLNH VSYGRLSFKY EEDSNRMLLQ
451 SVQDSLEKAL NKEAPVHPND EFTARIAGAS EKDIVHSGLE YTMTRSGEAI
501 IRTARKYNLG LDIRTAAYAN SIEKVYNTYR TAGFTFT
```

Start - End	Observed	Mr(expt)	Mr(calc)	Delta	Miss	Sequence
2 - 11	1074.59	1073.58	1073.59	-0.02	1	M.LSNLARTSGR.M

47 - 54	1081.56	1080.55	1079.46	1.08	0	K.MVDYYFDK.G	
69 - 77	1010.36	1009.36	1008.49	0.86	1	K.SNVMSTKDK.K	
94 - 102	1121.86	1120.85	1120.66	0.19	0	K.VLYITFPIR.R	
94 - 103	1277.98	1276.97	1276.77	0.21	1	K.VLYITFPIRR.D	
116 - 126	1291.94	1290.94	1290.64	0.29	1	R.AQHSEHRTPTK.G	
127 - 141	1700.06	1699.05	1699.75	-0.70	1	K.GGIRYSMDVCEDEVK.A	
127 - 141	1717.05	1716.04	1715.75	0.29	1	K.GGIRYSMDVCEDEVK.A	Oxidation (M)
131 - 141	1317.89	1316.88	1316.53	0.36	0	R.YSMDVCEDEVK.A	
131 - 141	1333.89	1332.89	1332.52	0.37	0	R.YSMDVCEDEVK.A	Oxidation (M)
131 - 150	2384.07	2383.06	2384.06	-1.00	1	R.YSMDVCEDEVKALSALMTYK.C	Carbamidomethyl (C); 2 Oxidation (M)
142 - 150	996.35	995.34	996.53	-1.19	0	K.ALSALMTYK.C	
142 - 150	1013.49	1012.48	1012.53	-0.05	0	K.ALSALMTYK.C	Oxidation (M)
151 - 162	1191.91	1190.90	1190.58	0.32	0	K.CAAVDVPFGGAK.G	Carbamidomethyl (C)
151 - 166	1475.99	1474.98	1474.76	0.22	1	K.CAAVDVPFGGAKGGVK.I	
171 - 179	1188.87	1187.86	1187.53	0.33	0	K.QYTDYEIEK.I	
183 - 190	948.57	947.57	946.56	1.01	1	R.RIAIEFAK.K	
191 - 210	2016.08	2015.08	2014.98	0.10	1	K.KGFLGPGVDVPAPDMGTGER.E	Oxidation (M)
192 - 210	1889.08	1888.07	1886.88	1.19	0	K.GFLGPGVDVPAPDMGTGER.E	Oxidation (M)
229 - 247	1880.14	1879.13	1878.97	0.16	0	R.DASACITGKPIVAGGIHGR.V	Carbamidomethyl (C)
265 - 271	907.35	906.35	907.37	-1.03	0	K.EPEYMNK.V	Glu->pyro-Glu (N-term E); Oxidation (M)
265 - 271	925.35	924.35	925.39	-1.04	0	K.EPEYMNK.V	Oxidation (M)
272 - 281	973.60	972.59	971.57	1.03	0	K.VGLSLGLEGK.T	
306 - 325	2225.23	2224.22	2223.08	1.14	0	K.VIGVQEWDCAVFNPDGIHPK.E	
306 - 331	3053.62	3052.61	3051.45	1.16	1	K.VIGVQEWDCAVFNPDGIHPKELEDWR.D	
326 - 338	1604.97	1603.97	1603.75	0.22	1	K.ELEDWRDENGTIK.N	
332 - 342	1261.94	1260.93	1261.63	-0.70	1	R.DENGTIKNFPK.A	
368 - 376	1051.62	1050.61	1051.55	-0.94	1	K.AIHKENANR.I	
368 - 376	1052.43	1051.42	1051.55	-0.13	1	K.AIHKENANR.I	
372 - 380	1026.54	1025.53	1024.54	0.99	1	K.ENANRIQAK.I	Glu->pyro-Glu (N-term E)
372 - 380	1043.37	1042.36	1042.55	-0.19	1	K.ENANRIQAK.I	
427 - 435	1058.55	1057.55	1058.53	-0.98	0	K.NLNHVSYGR.L	
427 - 435	1059.58	1058.57	1058.53	0.05	0	K.NLNHVSYGR.L	
440 - 446	913.55	912.54	911.36	1.18	0	K.YEEDSNR.M	
440 - 458	2300.28	2299.27	2299.06	0.21	1	K.YEEDSNRMLLQSVQDSLEK.A	Oxidation (M)
447 - 458	1405.87	1404.87	1405.71	-0.85	0	R.MLLQSVQDSLEK.A	Oxidation (M)
463 - 475	1464.96	1463.96	1463.68	0.28	0	K.EAPVHPNDEFTAR.I	Glu->pyro-Glu (N-term E)
463 - 475	1482.92	1481.91	1481.69	0.22	0	K.EAPVHPNDEFTAR.I	
483 - 495	1521.99	1520.99	1520.73	0.26	0	K.DIVHSGLEYTMTR.S	
483 - 495	1537.95	1536.94	1536.72	0.22	0	K.DIVHSGLEYTMTR.S	Oxidation (M)
496 - 505	1073.60	1072.59	1072.60	-0.01	1	R.SGEAIIRTAR.K	
506 - 514	1091.67	1090.66	1090.61	0.05	1	R.KYNLGLDIR.T	
507 - 514	964.54	963.54	962.52	1.02	0	K.YNLGLDIR.T	

515 - 524 1067.60 1066.60 1066.53 0.07 0 R.TAAYANSIEK.V

No match to: 896.58, 897.37, 900.35, 906.35, 917.36, 923.54, 924.36, 928.35, 932.55, 938.54, 940.36, 942.55, 951.35, 957.55, 958.36, 966.38, 969.36, 979.55, 985.39, 991.61, 992.36, 994.34, 1002.39, 1004.36, 1007.36, 1020.36, 1027.36, 1033.58, 1036.54, 1045.62, 1065.57, 1089.40, 1098.41, 1103.47, 1106.66, 1113.52, 1117.54, 1128.82, 1129.82, 1135.84, 1137.61, 1141.62, 1147.90, 1158.89, 1163.91, 1169.92, 1179.92, 1197.90, 1212.91, 1231.96, 1240.94, 1250.91, 1258.90, 1265.94, 1274.92, 1282.98, 1299.95, 1307.93, 1308.92, 1315.00, 1341.96, 1349.88, 1358.95, 1383.92, 1418.94, 1428.09, 1442.96, 1497.96, 1506.00, 1605.97, 1708.98, 1765.07, 1791.94, 1838.12, 1941.11, 1994.13, 2034.19, 2083.15, 2105.11, 2131.20, 2211.23, 2233.21, 2242.23, 2256.20, 2682.41

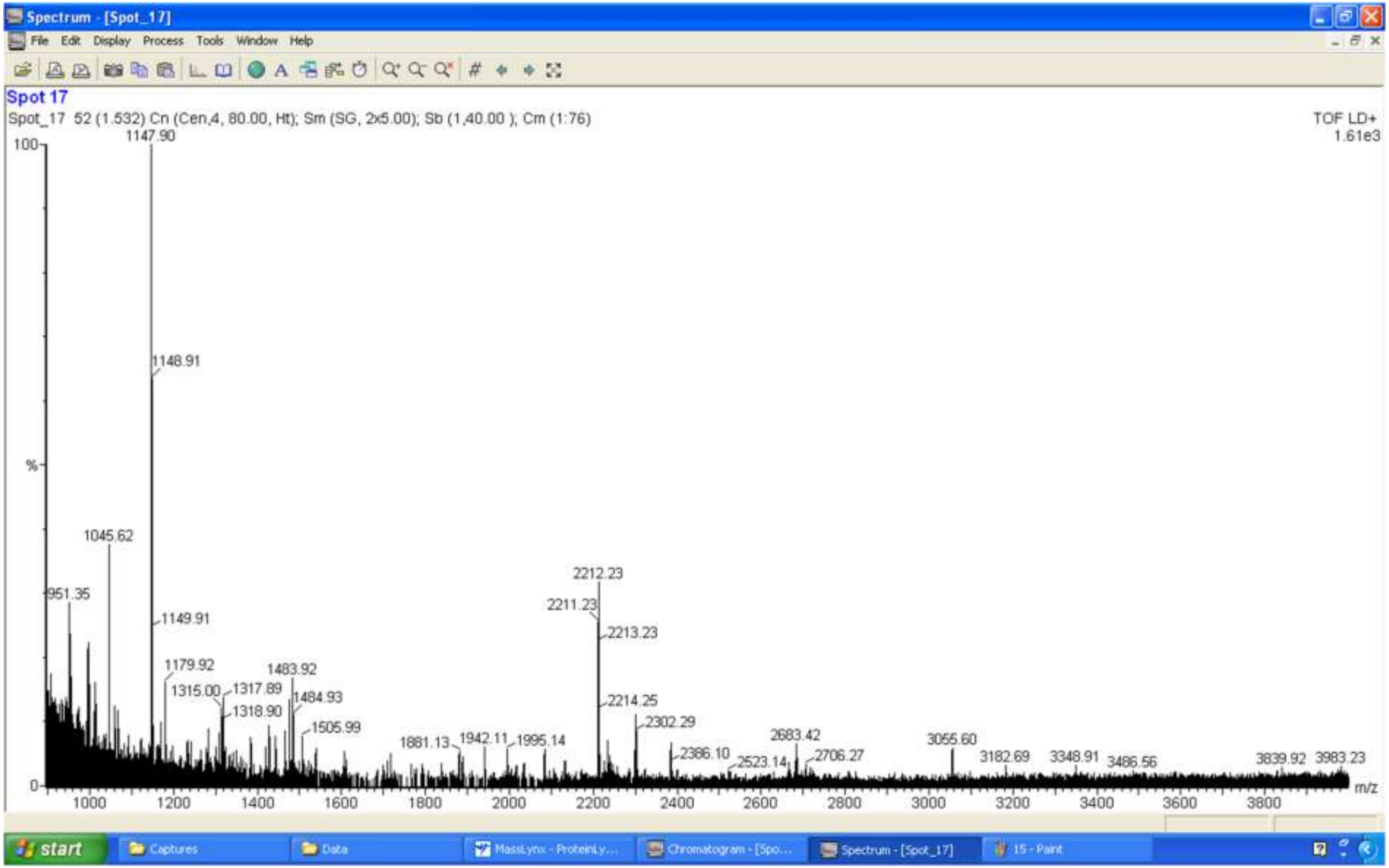

Spectrum - [Spot_17]
File Edit Display Process Tools Window Help
Spot 17
Spot_17 52 (1.532) Cn (Cen,4, 80.00, Ht); Sm (SG, 2x5.00); Sb (1,40.00); Cm (1:76)
TOF LD+
1.61e3
100
%
0
1147.90
1148.91
1045.62
951.35
1149.91
1179.92
1315.00
1317.89
1318.90
1483.92
1484.93
1505.99
1881.13
1942.11
1995.14
2212.23
2211.23
2213.23
2214.25
2302.29
2386.10
2523.14
2683.42
2706.27
3055.60
3182.69
3348.91
3486.56
3839.92
3983.23
m/z
1000 1200 1400 1600 1800 2000 2200 2400 2600 2800 3000 3200 3400 3600 3800
start
Captures
Data
MassLynx - ProteinLy...
Chromatogram - [Spo...
Spectrum - [Spot_17]
15 - Paint

Figure S1.15

Mascot Search Results Sport 18

User : **Paul Millares**
Email : **paul.millares@gmail.com**
Search title : **Spot 18**
Database : **Haemonchus 210108 (6387 sequences; 918038 residues)**
Timestamp : **1 Aug 2011 at 10:36:53 GMT**
Top Score : **54 for HCP06327_1, putative nuclear encoded protein Method: similarity and extension**

Mascot Score Histogram

Protein score is -10*Log(P), where P is the probability that the observed match is a random event.

Protein scores greater than 51 are significant (p<0.05).

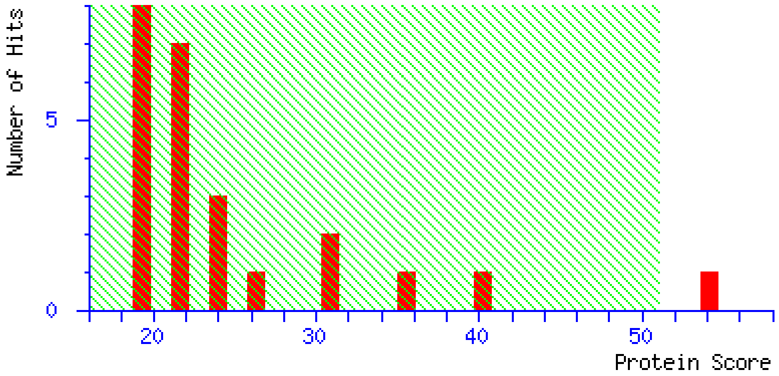

Concise Protein Summary Report

1. HCP06327_1 **Mass:** 18739 **Score:** **54** **Expect:** 0.025 **Matches:** 9
putative nuclear encoded protein Method: similarity and extension
HCP00857_1 **Mass:** 13007 **Score:** 24 **Expect:** 26 **Matches:** 4
putative nuclear encoded protein Method: ESTScan
HCP02099_1 **Mass:** 19901 **Score:** 22 **Expect:** 40 **Matches:** 5
putative nuclear encoded protein Method: ESTScan

HCP00674_4 **Mass:** 14920 **Score:** 19 **Expect:** 75 **Matches:** 4
putative nuclear encoded protein Method: similarity and extension
HCP00674_1 **Mass:** 14920 **Score:** 19 **Expect:** 75 **Matches:** 4
putative nuclear encoded protein Method: similarity and extension
HCP06208_1 **Mass:** 14920 **Score:** 19 **Expect:** 75 **Matches:** 4
putative nuclear encoded protein Method: similarity and extension
HCP08864_1 **Mass:** 16368 **Score:** 19 **Expect:** 82 **Matches:** 4
putative nuclear encoded protein Method: ESTScan

---

2. HCP02271_2 **Mass:** 24413 **Score:** 39 **Expect:** 0.73 **Matches:** 8
putative nuclear encoded protein Method: similarity and extension
HCP02271_1 **Mass:** 22097 **Score:** 22 **Expect:** 38 **Matches:** 5
putative nuclear encoded protein Method: similarity and extension
HCP07821_1 **Mass:** 16197 **Score:** 21 **Expect:** 54 **Matches:** 4
putative nuclear encoded protein Method: ESTScan

Search Parameters

Type of search : Peptide Mass Fingerprint
Enzyme : Trypsin
Variable modifications : Carbamidomethyl (C),Glu->pyro-Glu (N-term E),Oxidation (M)
Mass values : Monoisotopic
Protein Mass : Unrestricted
Peptide Mass Tolerance : ± 1.2 Da
Peptide Charge State : 1+
Max Missed Cleavages : 1
Number of queries : 14

Protein View

Match to: **HCP06327_1** Score: **54** Expect: **0.025**
putative nuclear encoded protein Method: similarity and extension

Nominal mass (M_r): **18739**; Calculated pI value: **5.96**
NCBI BLAST search of HCP06327_1 against nr
Unformatted sequence string for pasting into other applications

Variable modifications: Carbamidomethyl (C),Glu->pyro-Glu (N-term E),Oxidation (M)
Cleavage by Trypsin: cuts C-term side of KR unless next residue is P
Number of mass values searched: **14**
Number of mass values matched: **9**
Sequence Coverage: **25%**

Matched peptides shown in **Bold Red**

1 **IGMSSPSSGK** **RRMDTDVIK**L IESKHEVNIT GGLNEFNVKF YGPSGTAYEG
51 GVWRVRVELP EKYPFK**SPSI** **GFMNK**IFHPN IDEASGSVCL DVINQAWTAL
101 YDLANIFESF LPQLLTYPNP TDPLNGDAAA LYLHKPEEFK KK**CKDYVERF**
151 **ASEDALR**RFF NESSCK

Start - End	Observed	Mr(expt)	Mr(calc)	Delta	Miss	Sequence
1 - 10	950.48	949.47	949.45	0.02	0	-.IGMSSPSSGK.R
1 - 10	951.48	950.47	949.45	1.02	0	-.IGMSSPSSGK.R
1 - 10	967.51	966.50	965.45	1.05	0	-.IGMSSPSSGK.R Oxidation (M)
1 - 11	1122.06	1121.05	1121.55	-0.50	1	-.IGMSSPSSGKR.R Oxidation (M)
12 - 19	992.50	991.49	992.50	-1.00	1	R.RMDTDVIK.L Oxidation (M)
67 - 75	995.50	994.49	995.47	-0.99	0	K.SPSIGFMNK.I Oxidation (M)
67 - 75	997.50	996.49	995.47	1.02	0	K.SPSIGFMNK.I Oxidation (M)
143 - 149	969.50	968.49	968.44	0.05	1	K.CKDYVER.F Carbamidomethyl (C)
150 - 157	907.49	906.48	907.44	-0.96	0	R.FASEDALR.R

No match to: 900.47, 926.48, 936.49, 1010.52, 1142.79

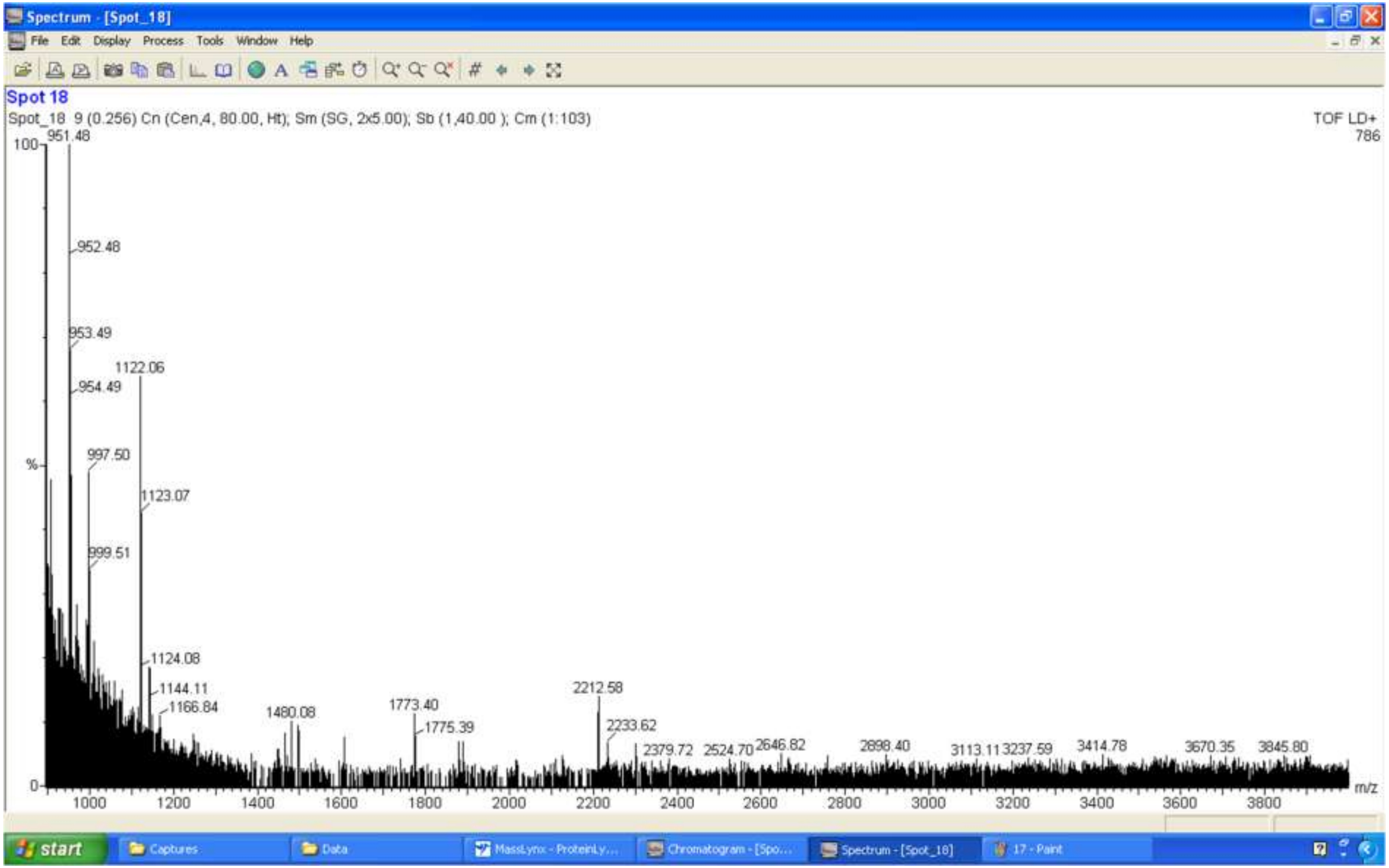
Spectrum - [Spot_18]
File Edit Display Process Tools Window Help
Spot 18
Spot_18 9 (0.256) Cn (Cen,4, 80.00, Ht); Sm (SG, 2x5.00); Sb (1,40.00); Cm (1:103)
TOF LD+
786
951.48
952.48
953.49
954.49
1122.06
997.50
1123.07
999.51
1124.08
1144.11
1166.84
1480.08
1773.40
1775.39
2212.58
2233.62
2379.72
2524.70
2646.82
2898.40
3113.11
3237.59
3414.78
3670.35
3845.80
100
%
0
m/z
1000
1200
1400
1600
1800
2000
2200
2400
2600
2800
3000
3200
3400
3600
3800
start
Captures
Data
MassLynx - ProteinLy...
Chromatogram - [Spo...
Spectrum - [Spot_18]
17 - Paint

Figure S1.16

Mascot Search Results Spot 19

```
User          : Paul Millares
Email         : paul.millares@gmail.com
Search title  : Spot 19
Database      : Haemonchus 210108 (6387 sequences; 918038 residues)
Timestamp     : 1 Aug 2011 at 10:37:18 GMT
Top Score     : 50 for HCP00199_6, putative nuclear encoded protein  Method: similarity and extension
```

Mascot Score Histogram

Protein score is -10*Log(P), where P is the probability that the observed match is a random event.

Protein scores greater than 51 are significant (p<0.05).

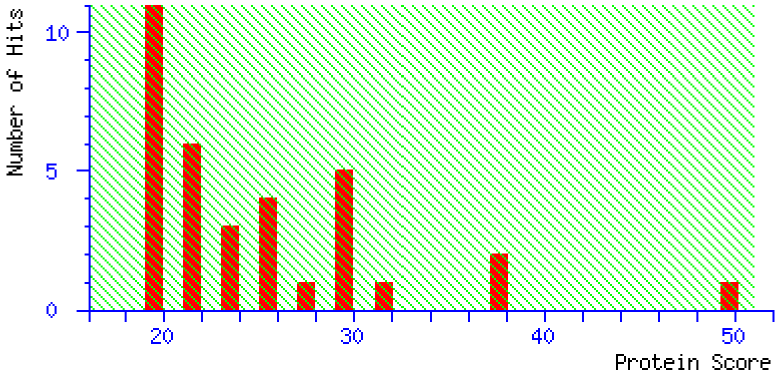

Concise Protein Summary Report

1. HCP00199_6 **Mass:** 37676 **Score:** 50 **Expect:** 0.068 **Matches:** 9

   putative nuclear encoded protein Method: similarity and extension

   HCP00199_8 **Mass:** 28973 **Score:** 38 **Expect:** 1.1 **Matches:** 7

   putative nuclear encoded protein Method: similarity and extension

   HCP00199_5 **Mass:** 26092 **Score:** 31 **Expect:** 5.2 **Matches:** 6

   putative nuclear encoded protein Method: similarity and extension

HCP00199_4 **Mass:** 26109 **Score:** 31 **Expect:** 5.3 **Matches:** 6
putative nuclear encoded protein Method: similarity and extension
HCP00199_3 **Mass:** 26937 **Score:** 30 **Expect:** 6 **Matches:** 6
putative nuclear encoded protein Method: similarity and extension
HCP02782_1 **Mass:** 3624 **Score:** 24 **Expect:** 25 **Matches:** 3
putative nuclear encoded protein Method: ESTScan
HCP12158_1 **Mass:** 15581 **Score:** 24 **Expect:** 28 **Matches:** 4
putative nuclear encoded protein Method: similarity and extension
HCP02508_1 **Mass:** 5805 **Score:** 22 **Expect:** 38 **Matches:** 3
putative nuclear encoded protein Method: ESTScan
HCP00199_2 **Mass:** 20826 **Score:** 20 **Expect:** 70 **Matches:** 4
putative nuclear encoded protein Method: similarity and extension

---

Search Parameters

Type of search : Peptide Mass Fingerprint
Enzyme : Trypsin
Variable modifications : Carbamidomethyl (C),Glu->pyro-Glu (N-term E),Oxidation (M)
Mass values : Monoisotopic
Protein Mass : Unrestricted
Peptide Mass Tolerance : ± 1.2 Da
Peptide Charge State : 1+
Max Missed Cleavages : 1
Number of queries : 20

Protein View

Match to: **HCP00199_6** Score: **50** Expect: **0.068**
putative nuclear encoded protein Method: similarity and extension

Nominal mass (M_r): **37676**; Calculated pI value: **5.20**
NCBI BLAST search of HCP00199_6 against nr
Unformatted sequence string for pasting into other applications

Variable modifications: Carbamidomethyl (C),Glu->pyro-Glu (N-term E),Oxidation (M)
Cleavage by Trypsin: cuts C-term side of KR unless next residue is P
Number of mass values searched: **20**

Number of mass values matched: **9**
Sequence Coverage: **33%**

Matched peptides shown in **Bold Red**

```
  1 MCDDEVAALV VDNGSGMCKA GFAGDDAPRA VFPSIVGRPR HQGVMVGMGQ
 51 KDSYVGDEAQ SKRGILTLKY PIEHGIVTNW DDMEKIWHHT FYNELRVAPE
101 EHPVLLTEAP LNPKANREKM TQIMFETFNT PAMYVAIQAV LSLYASGRTT
151 GVVLDSGDGV THTVPIYEGY ALPHAILRLD LAGRDLTDYL MKILTERGYS
201 FTTTAEREIV RDIKEKLCYV ALDFEQEMAT AASSSSLEKS YELPDGQVIT
251 VGNERFRCPE AMFQPSFIGM ESAGIHENSY NSIMKCDIDI RKDLYANTVL
301 SGGSTMYPGI ADRMQKEMTA LAPSTMKIKI IAPPERKYSV
```

Start - End	Observed	Mr(expt)	Mr(calc)	Delta	Miss	Sequence	
20 - 29	976.58	975.57	975.44	0.13	0	K.AGFAGDDAPR.A	
30 - 40	1199.12	1198.11	1197.70	0.41	0	R.AVFPSIVGRPR.H	
86 - 96	1516.11	1515.10	1514.74	0.36	0	K.IWHHTFYNELR.V	
97 - 114	1954.42	1953.41	1953.06	0.36	0	R.VAPEEHPVLLTEAPLNPK.A	
185 - 192	997.45	996.44	997.48	-1.04	0	R.DLTDYLMK.I	
198 - 207	1132.87	1131.87	1131.52	0.35	0	R.GYSFTTTAER.E	
240 - 255	1777.23	1776.22	1775.87	0.35	0	K.SYELPDGQVITVGNER.F	
293 - 313	2217.42	2216.41	2216.04	0.37	0	K.DLYANTVLSGGSTMYPGIADR.M	Oxidation (M)
328 - 336	1036.58	1035.57	1035.64	-0.07	1	K.IKIIAPPER.K	

No match to: 900.42, 907.43, 945.69, 952.42, 967.67, 1130.89, 1500.04, 1532.10, 1548.09, 1791.24, 1977.31

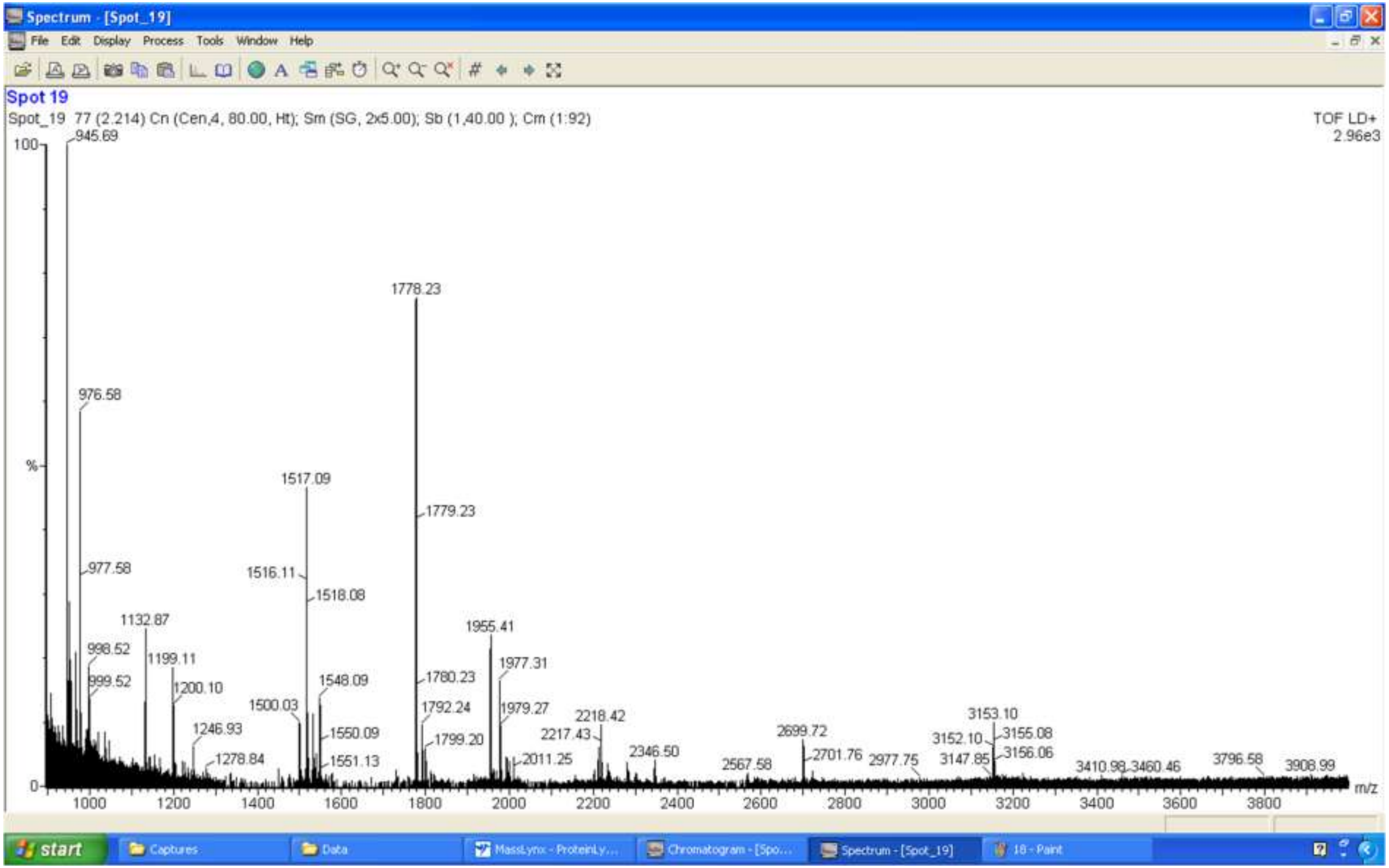

Spectrum - [Spot_19]
Spot 19
Spot_19 77 (2.214) Cn (Cen,4, 80.00, Ht); Sm (SG, 2x5.00); Sb (1,40.00); Cm (1:92)
TOF LD+
2.96e3
100
%
0
945.69
976.58
977.58
998.52
999.52
1132.87
1199.11
1200.10
1246.93
1278.84
1500.03
1516.11
1517.09
1518.08
1548.09
1550.09
1551.13
1778.23
1779.23
1780.23
1792.24
1799.20
1955.41
1977.31
1979.27
2011.25
2217.43
2218.42
2346.50
2567.58
2699.72
2701.76
2977.75
3147.85
3152.10
3153.10
3155.08
3156.06
3410.98
3460.46
3796.58
3908.99
1000
1200
1400
1600
1800
2000
2200
2400
2600
2800
3000
3200
3400
3600
3800
m/z

Figure S1.17

Mascot Search Results Spot 20

```
User            : Paul Millares
Email           : paul.millares@gmail.com
Search title    : Spot 20
Database        : Haemonchus 210108 (6387 sequences; 918038 residues)
Timestamp       : 1 Aug 2011 at 10:37:42 GMT
Top Score       : 31 for HCP00199_8, putative nuclear encoded protein  Method: similarity and extension
```

Mascot Score Histogram

Protein score is -10*Log(P), where P is the probability that the observed match is a random event.

Protein scores greater than 51 are significant (p<0.05).

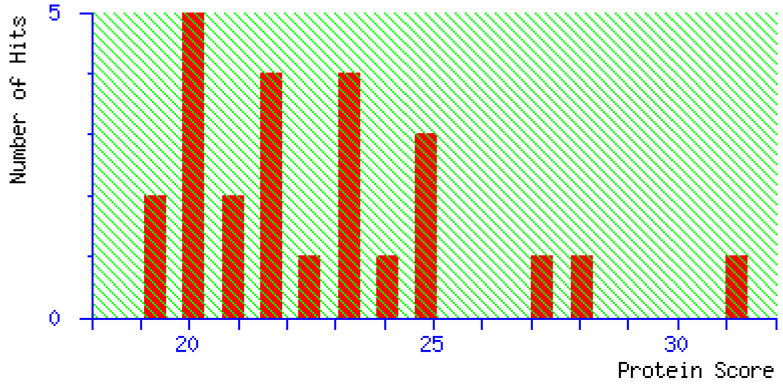

Concise Protein Summary Report

1. HCP00199_8 **Mass:** 28973 **Score:** 31 **Expect:** 4.8 **Matches:** 5
   putative nuclear encoded protein Method: similarity and extension
   HCP00199_6 **Mass:** 37676 **Score:** 26 **Expect:** 16 **Matches:** 5
   putative nuclear encoded protein Method: similarity and extension
   HCP00199_2 **Mass:** 20826 **Score:** 26 **Expect:** 15 **Matches:** 4
   putative nuclear encoded protein Method: similarity and extension

HCP00199_5 **Mass:** 26092 **Score:** 23 **Expect:** 31 **Matches:** 4
putative nuclear encoded protein Method: similarity and extension
HCP00199_4 **Mass:** 26109 **Score:** 23 **Expect:** 32 **Matches:** 4
putative nuclear encoded protein Method: similarity and extension
HCP00199_3 **Mass:** 26937 **Score:** 23 **Expect:** 34 **Matches:** 4
putative nuclear encoded protein Method: similarity and extension
HCP12158_1 **Mass:** 15581 **Score:** 21 **Expect:** 54 **Matches:** 3
putative nuclear encoded protein Method: similarity and extension

---

Search Parameters

Type of search : Peptide Mass Fingerprint
Enzyme : Trypsin
Variable modifications : Carbamidomethyl (C),Glu->pyro-Glu (N-term E),Oxidation (M)
Mass values : Monoisotopic
Protein Mass : Unrestricted
Peptide Mass Tolerance : ± 1.2 Da
Peptide Charge State : 1+
Max Missed Cleavages : 1
Number of queries : 13

Protein View

Match to: **HCP00199_8** Score: **31** Expect: **4.8**
putative nuclear encoded protein Method: similarity and extension

Nominal mass (M_r): **28973**; Calculated pI value: **5.30**
NCBI BLAST search of HCP00199_8 against nr
Unformatted sequence string for pasting into other applications

Variable modifications: Carbamidomethyl (C),Glu->pyro-Glu (N-term E),Oxidation (M)
Cleavage by Trypsin: cuts C-term side of KR unless next residue is P
Number of mass values searched: **13**
Number of mass values matched: **5**
Sequence Coverage: **25%**

Matched peptides shown in **Bold Red**

```
  1 AAGIRHEVVD NGSGMCKAGF AGDDAPRAVF PSIVGRPRHQ GVMVGMGQKD
 51 SYVGDEAQSK RGILTLKYPI EHGIVTNWDD MEKIWHHTFY NELRVAPEEH
101 PVLLTEAPLN PKANREKMTQ IMFETFNTPA MYVAIQAVLS LYASGRTTGV
151 VLDSGDGVTH TVPIYEGYAL PHAILRLDLA GRDLTDYLMK ILTERGYSFT
201 TTAEREIVRD IKEKLCYVAL DFEQEMATAA SSSSLEKSYE LPDGQVITVG
251 NERFRCPEAM F
```

Start - End	Observed	Mr(expt)	Mr(calc)	Delta	Miss	Sequence
18 - 27	976.59	975.58	975.44	0.14	0	K.AGFAGDDAPR.A
28 - 38	1199.11	1198.10	1197.70	0.40	0	R.AVFPSIVGRPR.H
84 - 94	1516.10	1515.09	1514.74	0.35	0	K.IWHHTFYNELR.V
95 - 112	1954.40	1953.39	1953.06	0.33	0	R.VAPEEHPVLLTEAPLNPK.A
238 - 253	1777.21	1776.21	1775.87	0.34	0	K.SYELPDGQVITVGNER.F

No match to: 945.70, 952.41, 1045.70, 1130.90, 1500.02, 1792.22, 1977.29, 2211.43

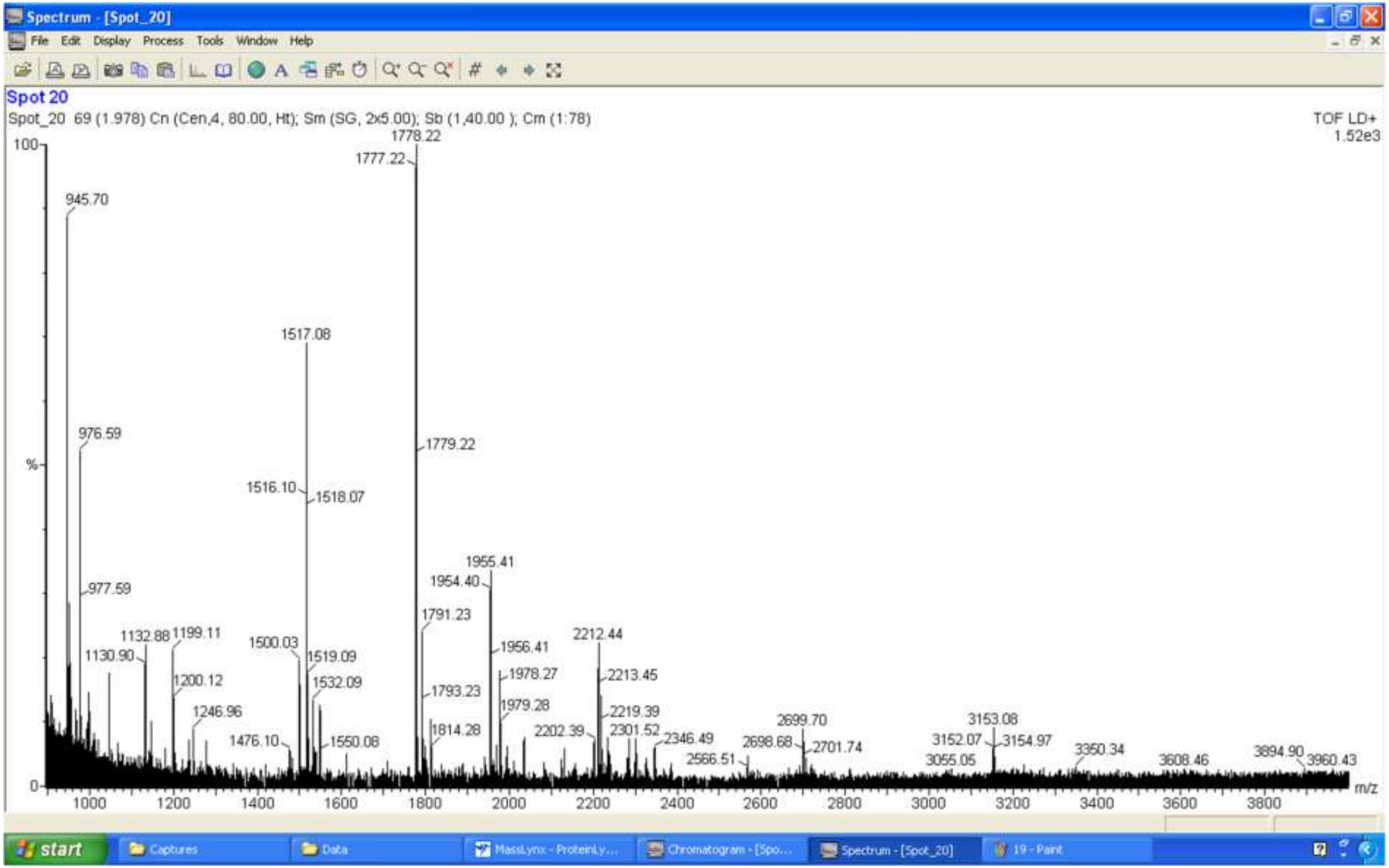
Spectrum - [Spot_20]
File Edit Display Process Tools Window Help
Spot 20
Spot_20 69 (1.978) Cn (Cen,4, 80.00, Ht); Sm (SG, 2x5.00); Sb (1,40.00); Cm (1:78)
TOF LD+
1.52e3
100
%
0
1778.22
1777.22
945.70
1517.08
976.59
1779.22
1516.10
1518.07
1955.41
1954.40
977.59
1791.23
1132.88
1199.11
1130.90
1500.03
1956.41
2212.44
1519.09
1200.12
1532.09
1978.27
2213.45
1793.23
1246.96
1979.28
2219.39
1814.28
2202.39
2301.52
2346.49
2699.70
3153.08
1476.10
1550.08
2698.68
2701.74
3152.07
3154.97
2566.51
3055.05
3350.34
3608.46
3894.90
3960.43
1000
1200
1400
1600
1800
2000
2200
2400
2600
2800
3000
3200
3400
3600
3800
m/z
start
Captures
Data
MassLynx - ProteinLy...
Chromatogram - [Spo...
Spectrum - [Spot_20]
19 - Paint

Figure S1.18

Mascot Search Results Spot 21

```
User            : Paul Millares
Email           : paul.millares@gmail.com
Search title    : Spot 21
Database        : Haemonchus 210108 (6387 sequences; 918038 residues)
Timestamp       : 1 Aug 2011 at 10:38:04 GMT
Top Score       : 79 for HCP00195_1, putative nuclear encoded protein  Method: similarity and extension
```

Mascot Score Histogram

Protein score is -10*Log(P), where P is the probability that the observed match is a random event.

Protein scores greater than 51 are significant (p<0.05).

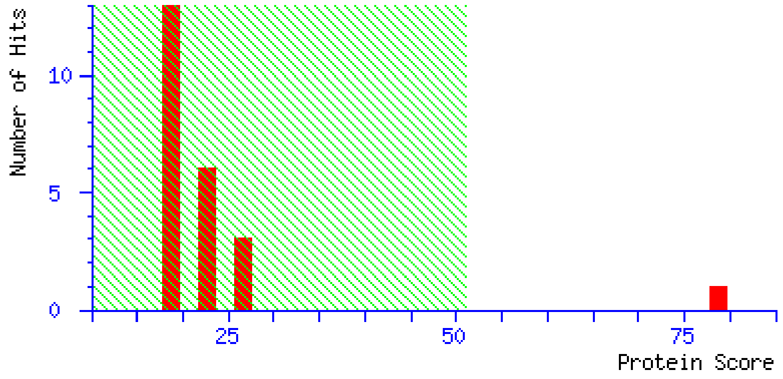

Concise Protein Summary Report

1. HCP00195_1 **Mass:** 24527 **Score:** 79 **Expect:** 8.6e-005 **Matches:** 8

   putative nuclear encoded protein Method: similarity and extension

   HCP09808_1 **Mass:** 24527 **Score:** 79 **Expect:** 8.6e-005 **Matches:** 8

   putative nuclear encoded protein Method: similarity and extension

   HCP10281_1 **Mass:** 9642 **Score:** 26 **Expect:** 15 **Matches:** 3

   putative nuclear encoded protein Method: Longest ORF

HCP00116_1 **Mass:** 10842 **Score:** 22 **Expect:** 39 **Matches:** 3
putative nuclear encoded protein Method: Longest ORF

HCP00081_1 **Mass:** 3719 **Score:** 21 **Expect:** 53 **Matches:** 2
putative nuclear encoded protein Method: Longest ORF

HCP02446_1 **Mass:** 13180 **Score:** 20 **Expect:** 64 **Matches:** 3
putative nuclear encoded protein Method: similarity and extension

HCP09424_1 **Mass:** 5469 **Score:** 20 **Expect:** 70 **Matches:** 2
putative nuclear encoded protein Method: Longest ORF

HCP11146_1 **Mass:** 13235 **Score:** 20 **Expect:** 70 **Matches:** 3
putative nuclear encoded protein Method: similarity and extension

HCP02375_1 **Mass:** 17935 **Score:** 19 **Expect:** 73 **Matches:** 3
putative nuclear encoded protein Method: ESTScan

HCP01900_1 **Mass:** 17456 **Score:** 19 **Expect:** 73 **Matches:** 3
putative nuclear encoded protein Method: similarity and extension

HCP02415_1 **Mass:** 36399 **Score:** 19 **Expect:** 77 **Matches:** 4
putative nuclear encoded protein Method: similarity and extension

HCP02271_2 **Mass:** 24413 **Score:** 19 **Expect:** 88 **Matches:** 3
putative nuclear encoded protein Method: similarity and extension

HCP09510_2 **Mass:** 20584 **Score:** 19 **Expect:** 88 **Matches:** 3
putative nuclear encoded protein Method: similarity and extension

HCP02375_2 **Mass:** 17612 **Score:** 19 **Expect:** 90 **Matches:** 3
putative nuclear encoded protein Method: ESTScan

HCP02572_1 **Mass:** 22145 **Score:** 19 **Expect:** 90 **Matches:** 3
putative nuclear encoded protein Method: similarity and extension

HCP05631_2 **Mass:** 23359 **Score:** 18 **Expect:** 92 **Matches:** 3
putative nuclear encoded protein Method: ESTScan

HCP02153_1 **Mass:** 7845 **Score:** 18 **Expect:** 92 **Matches:** 2
putative nuclear encoded protein Method: similarity and extension

---

2. HCP05082_1 **Mass:** 11013 **Score:** 26 **Expect:** 16 **Matches:** 3

putative nuclear encoded protein Method: ESTScan

HCP07265_1 **Mass:** 6012 **Score:** 19 **Expect:** 84 **Matches:** 2

putative nuclear encoded protein Method: Longest ORF

Search Parameters

Type of search : Peptide Mass Fingerprint
Enzyme : Trypsin
Variable modifications : Carbamidomethyl (C),Glu->pyro-Glu (N-term E),Oxidation (M)
Mass values : Monoisotopic
Protein Mass : Unrestricted
Peptide Mass Tolerance : ± 1.2 Da
Peptide Charge State : 1+
Max Missed Cleavages : 1
Number of queries : 11

Protein View

Match to: **HCP00195_1** Score: **79** Expect: **8.6e-005**
putative nuclear encoded protein Method: similarity and extension

Nominal mass (M_r): **24527**; Calculated pI value: **5.87**
NCBI BLAST search of HCP00195_1 against nr
Unformatted sequence string for pasting into other applications

Variable modifications: Carbamidomethyl (C),Glu->pyro-Glu (N-term E),Oxidation (M)
Cleavage by Trypsin: cuts C-term side of KR unless next residue is P
Number of mass values searched: **11**
Number of mass values matched: **8**
Sequence Coverage: **42%**

Matched peptides shown in **Bold Red**

```
  1 PAENRHEPHF PQQPVARQND DGSLELECFL DASPQPDIKW FYDNKEVKQD
 51 SRFQFKLDSK GNDSYSAILQ IKDLADSDAG GYRCAIVNPH GKGNANFNLK
101 LTGFSSPTFV EKPQISSRDD GQVMVMEFRA KSILKPTFVW HKGDEIVAQS
151 DRVNIVLREE ANQIYYAALE IKEPTKEKDA GQFICTAKND SGKLTATFTV
201 KFEVPQGAPT FTRKPQIL
```

Start - End Observed Mr(expt) Mr(calc) Delta Miss Sequence

61 - 72	1309.09	1308.08	1307.67	0.41	0	K.GNDSYSAILQIK.D	
73 - 83	1139.91	1138.90	1138.49	0.41	0	K.DLADSDAGGYR.C	
84 - 92	995.69	994.68	994.50	0.18	0	R.CAIVNPHGK.G	Carbamidomethyl (C)
101 - 118	1981.42	1980.42	1980.03	0.38	0	K.LTGFSSPTFVEKPQISSR.D	
119 - 129	1358.97	1357.97	1357.56	0.40	0	R.DDGQVMVMEFR.A	2 Oxidation (M)
143 - 152	1089.71	1088.70	1088.51	0.19	0	K.GDEIVAQSDR.V	
179 - 188	1110.81	1109.81	1109.52	0.29	0	K.DAGQFICTAK.N	Carbamidomethyl (C)
202 - 213	1350.09	1349.08	1348.68	0.40	0	K.FEVPQGAPTFTR.K	

No match to: 899.60, 1248.09, 1270.06

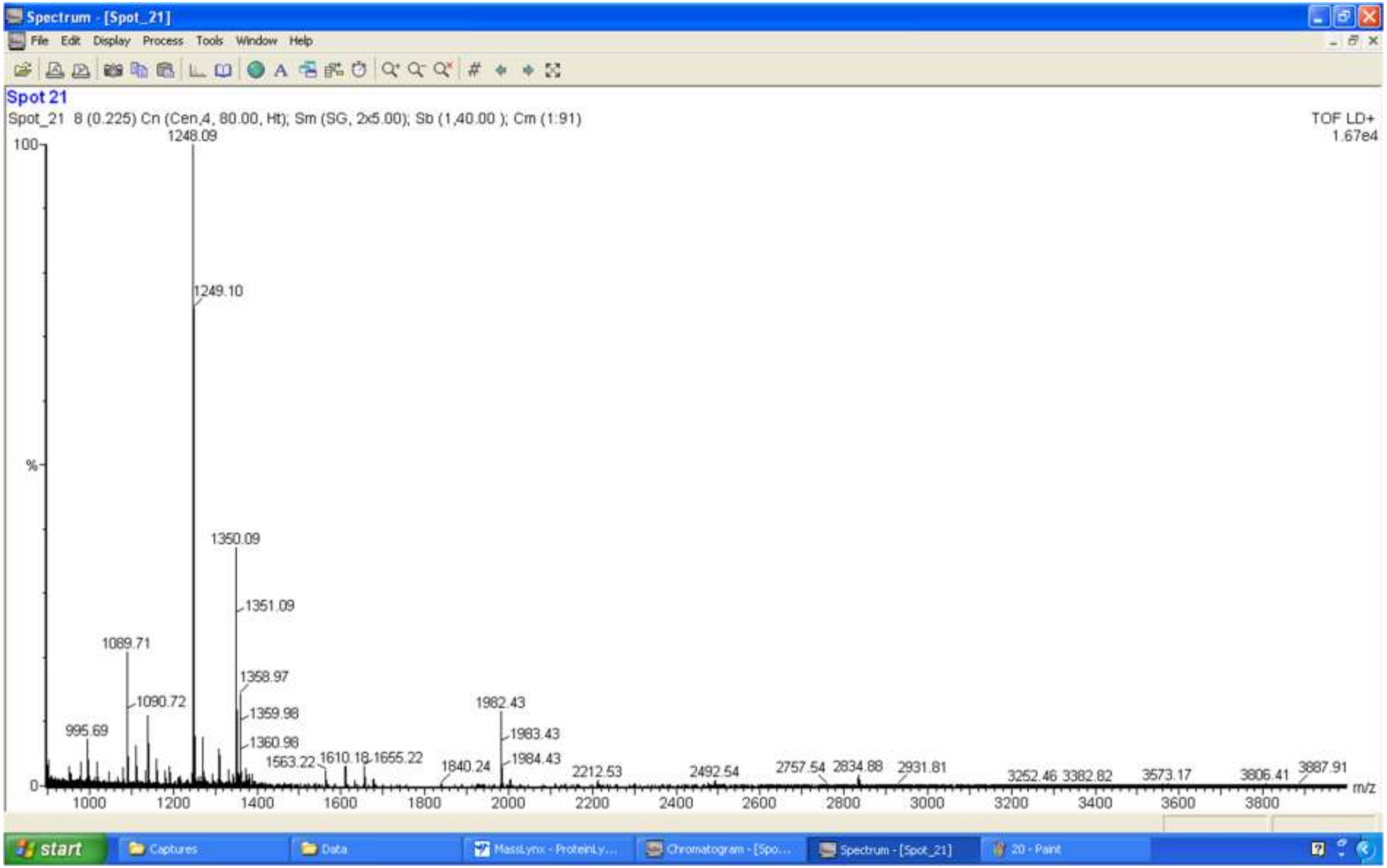
Spectrum - [Spot_21]
File Edit Display Process Tools Window Help
Spot 21
Spot_21 8 (0.225) Cn (Cen,4, 80.00, Ht); Sm (SG, 2x5.00); Sb (1,40.00); Cm (1:91)
TOF LD+
1.67e4
100
%
0
1248.09
1249.10
1350.09
1351.09
1089.71
1090.72
1358.97
1359.98
1360.98
995.69
1563.22
1610.18
1655.22
1840.24
1982.43
1983.43
1984.43
2212.53
2492.54
2757.54
2834.88
2931.81
3252.46
3382.82
3573.17
3806.41
3887.91
1000
1200
1400
1600
1800
2000
2200
2400
2600
2800
3000
3200
3400
3600
3800
m/z
start
Captures
Data
MassLynx - ProteinLy...
Chromatogram - [Spo...
Spectrum - [Spot_21]
20 - Paint

Figure S1.19

Mascot Search Results Spot 22

User : **Paul Millares**
Email : **paul.millares@gmail.com**
Search title : **Spot 22**
Database : **Haemonchus 210108 (6387 sequences; 918038 residues)**
Timestamp : **1 Aug 2011 at 10:38:28 GMT**
Top Score : **77 for HCP00195_1, putative nuclear encoded protein Method: similarity and extension**

Mascot Score Histogram

Protein score is -10*Log(P), where P is the probability that the observed match is a random event.

Protein scores greater than 51 are significant (p<0.05).

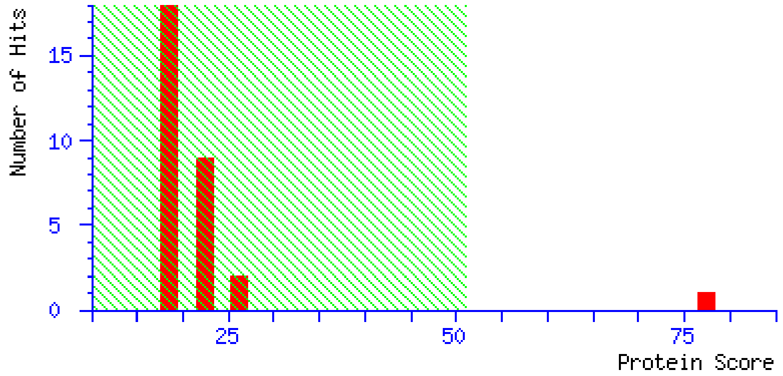

Concise Protein Summary Report

1. HCP00195_1 **Mass:** 24527 **Score:** 77 **Expect:** 0.00012 **Matches:** 9
putative nuclear encoded protein Method: similarity and extension
HCP09808_1 **Mass:** 24527 **Score:** 77 **Expect:** 0.00012 **Matches:** 9
putative nuclear encoded protein Method: similarity and extension
HCP10281_1 **Mass:** 9642 **Score:** 22 **Expect:** 37 **Matches:** 3
putative nuclear encoded protein Method: Longest ORF

HCP02415_1 **Mass:** 36399 **Score:** 22 **Expect:** 41 **Matches:** 5
putative nuclear encoded protein Method: similarity and extension
HCP11146_1 **Mass:** 13235 **Score:** 20 **Expect:** 67 **Matches:** 3
putative nuclear encoded protein Method: similarity and extension
HCP01172_1 **Mass:** 13283 **Score:** 19 **Expect:** 77 **Matches:** 3
putative nuclear encoded protein Method: Longest ORF
HCP06885_1 **Mass:** 14903 **Score:** 19 **Expect:** 79 **Matches:** 3
putative nuclear encoded protein Method: ESTScan
HCP06602_1 **Mass:** 7947 **Score:** 19 **Expect:** 80 **Matches:** 3
putative nuclear encoded protein Method: similarity and extension
HCP00161_1 **Mass:** 17285 **Score:** 19 **Expect:** 88 **Matches:** 3
putative nuclear encoded protein Method: similarity and extension
HCP00116_1 **Mass:** 10842 **Score:** 18 **Expect:** 92 **Matches:** 3
putative nuclear encoded protein Method: Longest ORF

---

2. HCP07265_1 **Mass:** 6012 **Score:** 26 **Expect:** 15 **Matches:** 3
putative nuclear encoded protein Method: Longest ORF

Search Parameters

Type of search : Peptide Mass Fingerprint
Enzyme : Trypsin
Variable modifications : Carbamidomethyl (C),Glu->pyro-Glu (N-term E),Oxidation (M)
Mass values : Monoisotopic
Protein Mass : Unrestricted
Peptide Mass Tolerance : ± 1.2 Da
Peptide Charge State : 1+
Max Missed Cleavages : 1
Number of queries : 15

Protein View

Match to: **HCP00195_1** Score: **77** Expect: **0.00012**
putative nuclear encoded protein Method: similarity and extension

Nominal mass (M_r): **24527**; Calculated pI value: **5.87**
NCBI BLAST search of HCP00195_1 against nr
Unformatted sequence string for pasting into other applications

Variable modifications: Carbamidomethyl (C),Glu->pyro-Glu (N-term E),Oxidation (M)
Cleavage by Trypsin: cuts C-term side of KR unless next residue is P
Number of mass values searched: **15**
Number of mass values matched: **9**
Sequence Coverage: **49%**

Matched peptides shown in **Bold Red**

1 PAENRHEPHF PQQPVARQND DGSLELECFL DASPQPDIKW FYDNKEVKQD
51 SRFQFKLDSK **GNDSYSAILQ IKDLADSDAG GYRCAIVNPH GK**GNANFNLK
101 **LTGFSSPTFV EKPQISSRDD GQVMVMEFR**A KSILKPTFVW HK**GDEIVAQS**
151 **DR**VNIVLR**EE ANQIYYAALE IK**EPTKEK**DA GQFICTAK**ND SGKLTATFTV
201 K**FEVPQGAPT FTR**KPQIL

Start - End	Observed	Mr(expt)	Mr(calc)	Delta	Miss	Sequence
61 - 72	1309.06	1308.06	1307.67	0.38	0	K.GNDSYSAILQIK.D
73 - 83	1139.88	1138.87	1138.49	0.38	0	K.DLADSDAGGYR.C
84 - 92	995.66	994.65	994.50	0.15	0	R.CAIVNPHGK.G Carbamidomethyl (C)
101 - 118	1981.39	1980.39	1980.03	0.36	0	K.LTGFSSPTFVEKPQISSR.D
119 - 129	1358.95	1357.94	1357.56	0.38	0	R.DDGQVMVMEFR.A 2 Oxidation (M)
143 - 152	1089.69	1088.68	1088.51	0.17	0	K.GDEIVAQSDR.V
159 - 172	1655.19	1654.19	1653.82	0.36	0	R.EEANQIYYAALEIK.E
179 - 188	1110.79	1109.79	1109.52	0.27	0	K.DAGQFICTAK.N Carbamidomethyl (C)
202 - 213	1350.07	1349.06	1348.68	0.38	0	K.FEVPQGAPTFTR.K

No match to: 951.42, 978.64, 1159.93, 1244.03, 1248.07, 1610.15

Figure S1.20

Mascot Search Results Spot 22

```
User            : Paul Millares
Email           : paul.millares@gmail.com
Search title    : Spot 22
MS data file    : Spot 22.txt
Database        : NCBInr 20110729 (14821581 sequences; 5074018658 residues)
Timestamp       : 3 Aug 2011 at 21:32:08 GMT
Top Score       : 91 for gi|325516326, disorganized muscle protein 1 [Haemonchus contortus]
```

Mascot Score Histogram

Protein score is -10*Log(P), where P is the probability that the observed match is a random event.

Protein scores greater than 84 are significant (p<0.05).

Concise Protein Summary Report

1. gi|325516326 **Mass:** 35597 **Score:** **91** **Expect:** 0.012 **Matches:** 11
   disorganized muscle protein 1 [Haemonchus contortus]
   gi|297726473 **Mass:** 56258 **Score:** 50 **Expect:** 1.4e+02 **Matches:** 10
   Os08g0442350 [Oryza sativa Japonica Group]

2. gi|317484595 **Mass:** 24644 **Score:** 56 **Expect:** 36 **Matches:** 9
cyclic nucleotide-binding domain-containing protein [Bilophila wadsworthia 3_1_6]

Search Parameters

Type of search : Peptide Mass Fingerprint
Enzyme : Trypsin
Variable modifications : Carbamidomethyl (C),Glu->pyro-Glu (N-term E),Oxidation (M)
Mass values : Monoisotopic
Protein Mass : Unrestricted
Peptide Mass Tolerance : ± 1.2 Da
Peptide Charge State : 1+
Max Missed Cleavages : 1
Number of queries : 15

Protein View

Match to: **gi|325516326** Score: **91** Expect: **0.012**
disorganized muscle protein 1 [Haemonchus contortus]
Found in search of Spot 22.txt

Nominal mass (M_r): **35597**; Calculated pI value: **5.16**
NCBI BLAST search of gi|325516326 against nr
Unformatted sequence string for pasting into other applications

Taxonomy: Haemonchus contortus

Variable modifications: Carbamidomethyl (C),Glu->pyro-Glu (N-term E),Oxidation (M)
Cleavage by Trypsin: cuts C-term side of KR unless next residue is P
Number of mass values searched: **15**
Number of mass values matched: **11**
Sequence Coverage: **41%**

Matched peptides shown in **Bold Red**

1 MPEGK**APHFP QQPVAR**QNDD GSLELECFLD ASPQPDIKWF YDNKEVKQDS
51 RFQFKLDSK**G NDSYSAILQI KDLADSDAGG YRCAIVNPHG K**GNANFNLK**L**
101 **TGFSSPTFVE KPQISSRDDG QVMVMEFR**AK SILKPTFVWH K**GDEIVAQSD**
151 **R**VNIVLR**EEA NQIYYAALEI K**EPTKEK**DAG QFICTAK**NDS GKLTATFTVK
201 **FEVPQGAPTF TR**KPQILQVT SDSGDPAIVF DIGYQADRNP EVTWINPKGK
251 KMKESTRIR**F LTSPDGPNNT FTAK**LELKNY KAKDSGTYTC NIKNEAGEAN

301 VELTLNIEGP MDDGGDDGSE A

Start - End	Observed	Mr(expt)	Mr(calc)	Delta	Miss	Sequence
6 - 16	1248.0714	1247.0641	1246.6571	0.4070	0	K.APHFPQQPVAR.Q
60 - 71	1309.0642	1308.0569	1307.6721	0.3848	0	K.GNDSYSAILQIK.D
72 - 82	1139.8812	1138.8739	1138.4891	0.3848	0	K.DLADSDAGGYR.C
83 - 91	995.6602	994.6529	994.5018	0.1511	0	R.CAIVNPHGK.G Carbamidomethyl (C)
100 - 117	1981.3940	1980.3867	1980.0316	0.3551	0	K.LTGFSSPTFVEKPQISSR.D
118 - 128	1358.9493	1357.9420	1357.5643	0.3778	0	R.DDGQVMVMEFR.A 2 Oxidation (M)
142 - 151	1089.6886	1088.6813	1088.5098	0.1715	0	K.GDEIVAQSDR.V
158 - 171	1655.1941	1654.1868	1653.8249	0.3619	0	R.EEANQIYYAALEIK.E
178 - 187	1110.7930	1109.7857	1109.5175	0.2682	0	K.DAGQFICTAK.N Carbamidomethyl (C)
201 - 212	1350.0665	1349.0592	1348.6776	0.3817	0	K.FEVPQGAPTFTR.K
260 - 274	1610.1490	1609.1417	1608.7784	0.3633	0	R.FLTSPDGPNNTFTAK.L

No match to: 951.4172, 978.6419, 1159.9283, 1244.0259

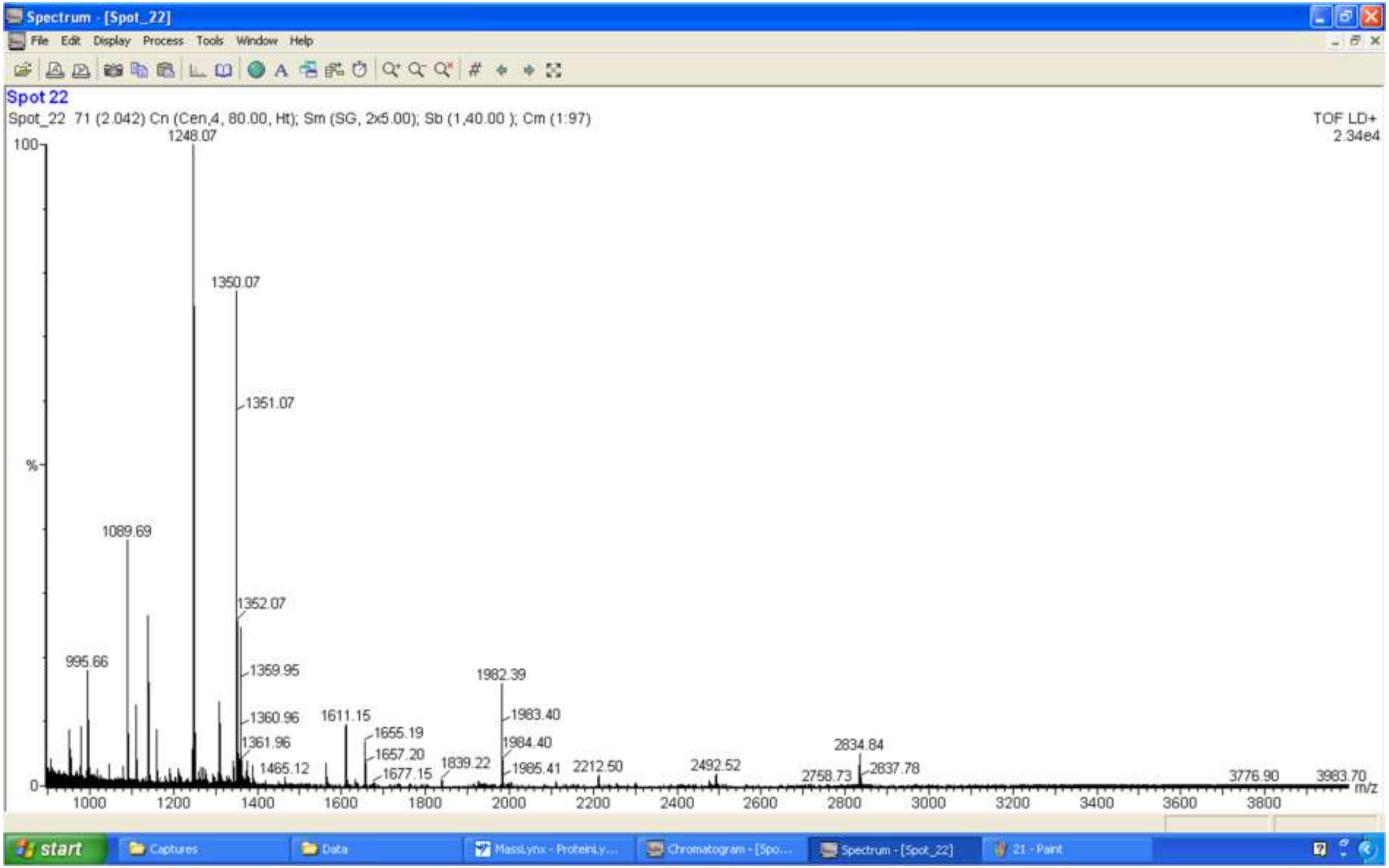
Spectrum - [Spot_22]
File Edit Display Process Tools Window Help
Spot 22
Spot_22 71 (2.042) Cn (Cen,4, 80.00, Ht); Sm (SG, 2x5.00); Sb (1,40.00); Cm (1:97)
TOF LD+
2.34e4
100
%
0
1248.07
1350.07
1351.07
1089.69
1352.07
995.66
1359.95
1982.39
1360.96
1611.15
1983.40
1655.19
1361.96
1984.40
2834.84
1465.12
1657.20
1839.22
1985.41
2212.50
2492.52
2837.78
1677.15
2758.73
3776.90
3983.70
m/z
1000
1200
1400
1600
1800
2000
2200
2400
2600
2800
3000
3200
3400
3600
3800
start
Captures
Data
MassLynx - ProteinLy...
Chromatogram - [Spo...
Spectrum - [Spot_22]
21 - Paint

Figure S1.21

Mascot Search Results Spot 24

User : **Paul Millares**
Email : **paul.millares@gmail.com**
Search title : **Spot 24**
Database : **Haemonchus 210108 (6387 sequences; 918038 residues)**
Timestamp : **1 Aug 2011 at 10:39:15 GMT**
Top Score : **40 for HCP01204_1, putative nuclear encoded protein Method: similarity and extension**

Mascot Score Histogram

Protein score is -10*Log(P), where P is the probability that the observed match is a random event.

Protein scores greater than 51 are significant (p<0.05).

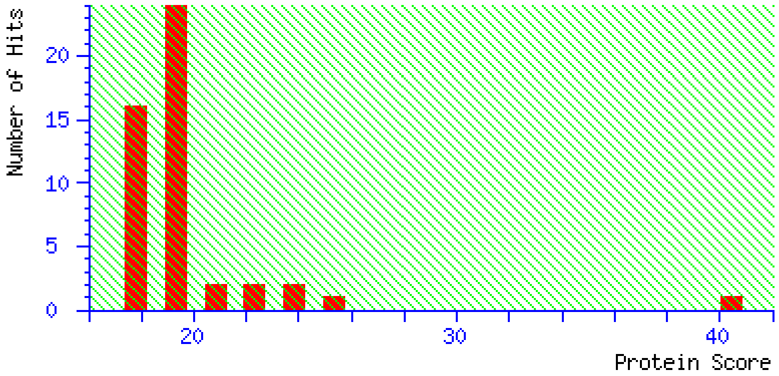

Concise Protein Summary Report

1. HCP01204_1 **Mass:** 25988 **Score:** 40 **Expect:** 0.58 **Matches:** 14

putative nuclear encoded protein Method: similarity and extension

---

Search Parameters

Type of search : **Peptide Mass Fingerprint**
Enzyme : **Trypsin**

Variable modifications : Carbamidomethyl (C),Glu->pyro-Glu (N-term E),Oxidation (M)
Mass values : Monoisotopic
Protein Mass : Unrestricted
Peptide Mass Tolerance : ± 1.2 Da
Peptide Charge State : 1+
Max Missed Cleavages : 1
Number of queries : 61

Protein View

Match to: **HCP01204_1** Score: **40** Expect: **0.58**
putative nuclear encoded protein Method: similarity and extension

Nominal mass (M_r): **25988**; Calculated pI value: **6.10**
NCBI BLAST search of HCP01204_1 against nr
Unformatted sequence string for pasting into other applications

Variable modifications: Carbamidomethyl (C),Glu->pyro-Glu (N-term E),Oxidation (M)
Cleavage by Trypsin: cuts C-term side of KR unless next residue is P
Number of mass values searched: **61**
Number of mass values matched: **14**
Sequence Coverage: **53%**

Matched peptides shown in **Bold Red**

```
  1 GVPLSAQAID TERIEPKRKM AGGDAVKYET IERGSLYSMD YRIFIKGPDG
 51 VISPWHDIPL YADESKKIYN MIVEIPRWTN AKMEMATAEP MNPIKQDLKK
101 GLPRFVHNIF PHKGYIWNYG ALPQTWEDPN HTVPDTGAKG DNDPIDVIEI
151 GSKVQGRGAV VKVKIVGTLA LIDEGETDWK LVAIDIEDPV AAQINNIGDV
201 EKHFPGLLKA THEWFRIYKI PNWKAGKQIC F
```

Start - End	Observed	Mr(expt)	Mr(calc)	Delta	Miss	Sequence
34 - 42	1106.75	1105.74	1106.47	-0.73	0	R.GSLYSMDYR.I Oxidation (M)
47 - 66	2195.47	2194.46	2195.05	-0.59	0	K.GPDGVISPWHDIPLYADESK.K
47 - 67	2323.42	2322.41	2323.15	-0.74	1	K.GPDGVISPWHDIPLYADESKK.I
67 - 77	1391.16	1390.15	1390.76	-0.61	1	K.KIYNMIVEIPR.W Oxidation (M)
68 - 77	1247.10	1246.09	1246.67	-0.58	0	K.IYNMIVEIPR.W
68 - 77	1263.02	1262.01	1262.67	-0.66	0	K.IYNMIVEIPR.W Oxidation (M)
83 - 95	1494.11	1493.10	1493.66	-0.55	0	K.MEMATAEPMNPIK.Q 2 Oxidation (M)
83 - 95	1510.19	1509.18	1509.65	-0.47	0	K.MEMATAEPMNPIK.Q 3 Oxidation (M)
105 - 113	1138.01	1137.00	1137.61	-0.61	0	R.FVHNIFPHK.G
140 - 153	1471.15	1470.14	1470.72	-0.58	0	K.GDNDPIDVIEIGSK.V

165 - 180	1759.21	1758.20	1758.90	-0.70	0	K.IVGTLALIDEGETDWK.L
181 - 202	2336.58	2335.57	2335.23	0.35	0	K.LVAIDIEDPVAAQINNIGDVEK.H
210 - 216	946.63	945.62	945.45	0.18	0	K.ATHEWFR.I
210 - 219	1350.09	1349.08	1349.69	-0.61	1	K.ATHEWFRIYK.I

No match to: 918.67, 936.58, 951.44, 960.62, 977.69, 983.72, 993.69, 1004.70, 1016.73, 1044.73, 1084.77, 1160.04, 1179.06, 1183.03, 1213.01, 1235.07, 1278.07, 1294.06, 1300.01, 1308.07, 1310.04, 1323.03, 1341.03, 1365.09, 1372.10, 1433.13, 1449.27, 1532.20, 1548.12, 1569.11, 1612.09, 1639.25, 1661.28, 1684.21, 1716.20, 1763.23, 1791.26, 1801.18, 1907.37, 2018.30, 2039.27, 2064.43, 2087.42, 2211.50, 2300.56, 2358.53, 2847.78

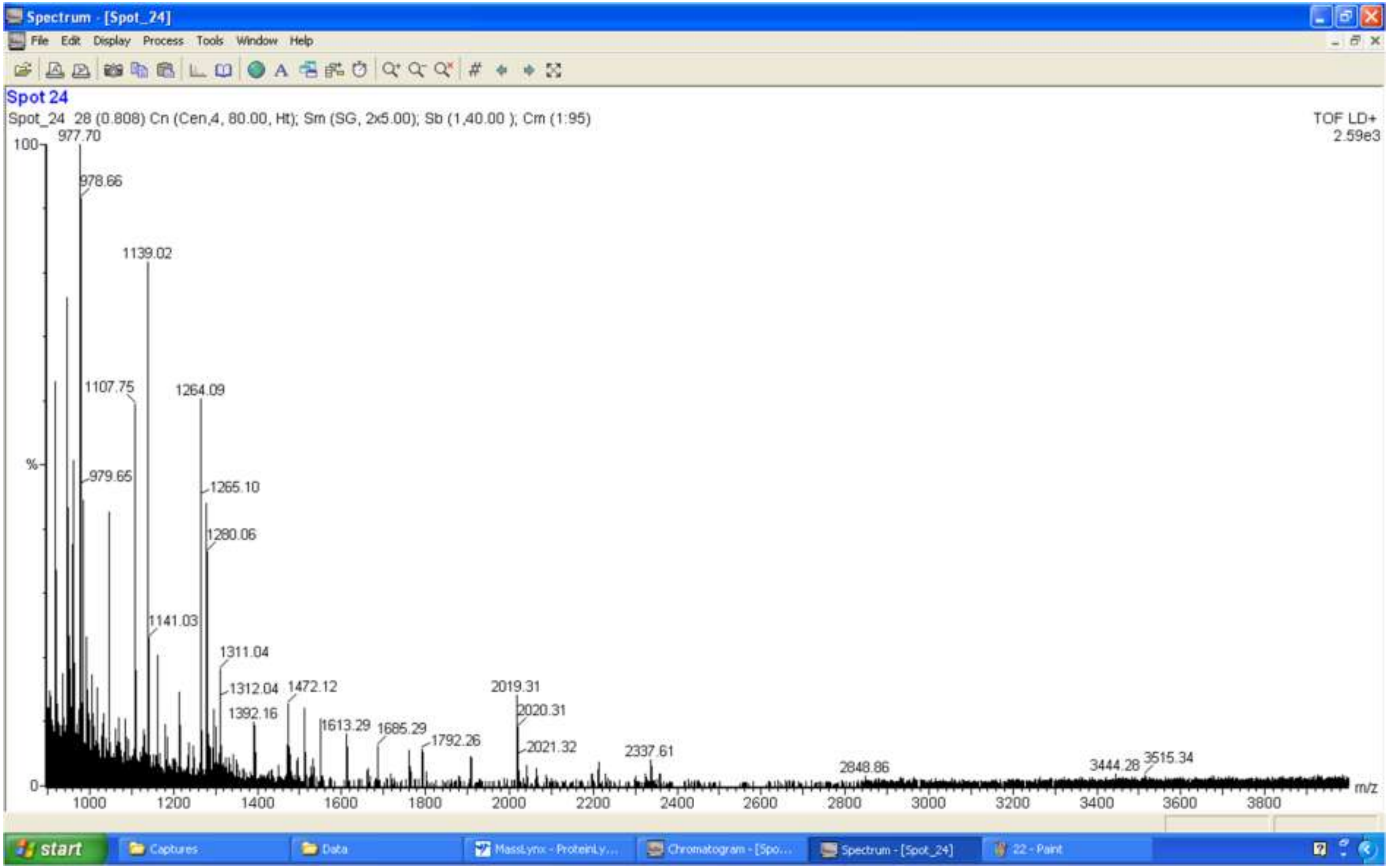

Spectrum - [Spot_24]
File Edit Display Process Tools Window Help
Spot 24
Spot_24 28 (0.808) Cn (Cen,4, 80.00, Ht); Sm (SG, 2x5.00); Sb (1,40.00); Cm (1:95)
TOF LD+
2.59e3
100
%
0
977.70
978.66
1139.02
1107.75
1264.09
979.65
1265.10
1280.06
1141.03
1311.04
1312.04
1472.12
1392.16
1613.29
1685.29
1792.26
2019.31
2020.31
2021.32
2337.61
2848.86
3444.28
3515.34
1000 1200 1400 1600 1800 2000 2200 2400 2600 2800 3000 3200 3400 3600 3800
m/z
start
Captures
Data
MassLynx - ProteinLy...
Chromatogram - [Spo...
Spectrum - [Spot_24]
22 - Paint

Figure S1.22

Mascot Search Results Spot 25

```
User            : Paul Millares
Email           : paul.millares@gmail.com
Search title    : Spot 25
Database        : Haemonchus 210108 (6387 sequences; 918038 residues)
Timestamp       : 1 Aug 2011 at 10:39:37 GMT
Top Score       : 53 for HCP01204_1, putative nuclear encoded protein  Method: similarity and extension
```

Mascot Score Histogram

Protein score is -10*Log(P), where P is the probability that the observed match is a random event.

Protein scores greater than 51 are significant (p<0.05).

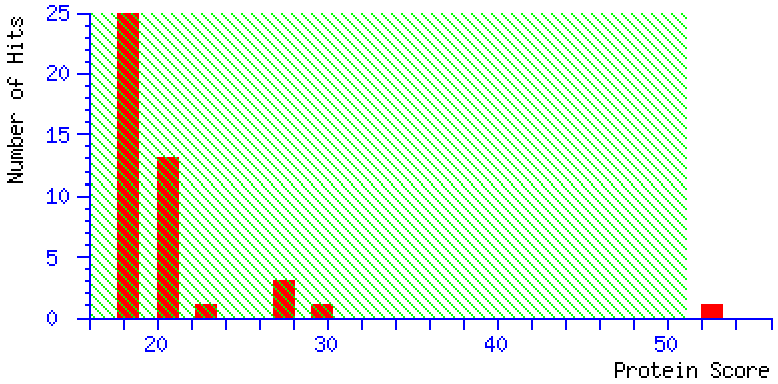

Concise Protein Summary Report

1. HCP01204_1 **Mass:** 25988 **Score:** 53 **Expect:** 0.036 **Matches:** 13

   putative nuclear encoded protein Method: similarity and extension

---

2. HCP06690_1 **Mass:** 14593 **Score:** 31 **Expect:** 5.7 **Matches:** 7

   putative nuclear encoded protein Method: similarity and extension

   HCP06690_2 **Mass:** 18676 **Score:** 25 **Expect:** 18 **Matches:** 7

putative nuclear encoded protein Method: similarity and extension

---

Search Parameters

Type of search : Peptide Mass Fingerprint
Enzyme : Trypsin
Variable modifications : Carbamidomethyl (C),Glu->pyro-Glu (N-term E),Oxidation (M)
Mass values : Monoisotopic
Protein Mass : Unrestricted
Peptide Mass Tolerance : ± 1.2 Da
Peptide Charge State : 1+
Max Missed Cleavages : 1
Number of queries : 41

Protein View

Match to: **HCP01204_1** Score: **53** Expect: **0.036**
putative nuclear encoded protein Method: similarity and extension

Nominal mass (M_r): **25988**; Calculated pI value: **6.10**
NCBI BLAST search of HCP01204_1 against nr
Unformatted sequence string for pasting into other applications

Variable modifications: Carbamidomethyl (C),Glu->pyro-Glu (N-term E),Oxidation (M)
Cleavage by Trypsin: cuts C-term side of KR unless next residue is P
Number of mass values searched: **41**
Number of mass values matched: **13**
Sequence Coverage: **50%**

Matched peptides shown in **Bold Red**

1 GVPLSAQAID TERIEPKRKM AGGDAVKYET IER**GSLYSMD YR**IFIK**GPDG**
51 **VISPWHDIPL YADESKKIYN MIVEIPR**WTN AKMEMATAEP MNPIKQDLKK
101 GLPR**FVHNIF PHK**GYIWNYG ALPQTWEDPN HTVPDTGAK**G DNDPIDVIEI**
151 **GSKVQGRGAV VK**VK**IVGTLA LIDEGETDWK LVAIDIEDPV AAQINNIGDV**
201 **EK**HFPGLLK**A THEWFR**IYKI PNWKAGKQIC F

Start - End	Observed	Mr(expt)	Mr(calc)	Delta	Miss	Sequence
34 - 42	1091.68	1090.68	1090.48	0.20	0	R.GSLYSMDYR.I
34 - 42	1107.76	1106.75	1106.47	0.28	0	R.GSLYSMDYR.I Oxidation (M)
47 - 66	2196.41	2195.40	2195.05	0.35	0	K.GPDGVISPWHDIPLYADESK.K

47 - 67	2324.52	2323.51	2323.15	0.36	1	K.GPDGVISPWHDIPLYADESKK.I
67 - 77	1392.16	1391.16	1390.76	0.39	1	K.KIYNMIVEIPR.W Oxidation (M)
68 - 77	1248.10	1247.09	1246.67	0.42	0	K.IYNMIVEIPR.W
68 - 77	1264.10	1263.09	1262.67	0.43	0	K.IYNMIVEIPR.W Oxidation (M)
105 - 113	1139.02	1138.01	1137.61	0.40	0	R.FVHNIFPHK.G
140 - 153	1472.11	1471.10	1470.72	0.38	0	K.GDNDPIDVIEIGSK.V
154 - 162	914.45	913.44	912.55	0.89	1	K.VQGRGAVVK.V
165 - 180	1760.28	1759.28	1758.90	0.37	0	K.IVGTLALIDEGETDWK.L
181 - 202	2336.61	2335.60	2335.23	0.37	0	K.LVAIDIEDPVAAQINNIGDVEK.H
210 - 216	946.63	945.62	945.45	0.17	0	K.ATHEWFR.I

No match to: 900.44, 907.45, 917.45, 925.45, 939.45, 953.45, 957.46, 960.63, 969.49, 978.62, 984.69, 993.46, 995.47, 997.46, 1045.74, 1068.82, 1161.04, 1167.08, 1177.02, 1232.01, 1279.06, 1295.05, 1311.04, 1335.28, 1449.30, 2018.28, 2211.47, 2846.84

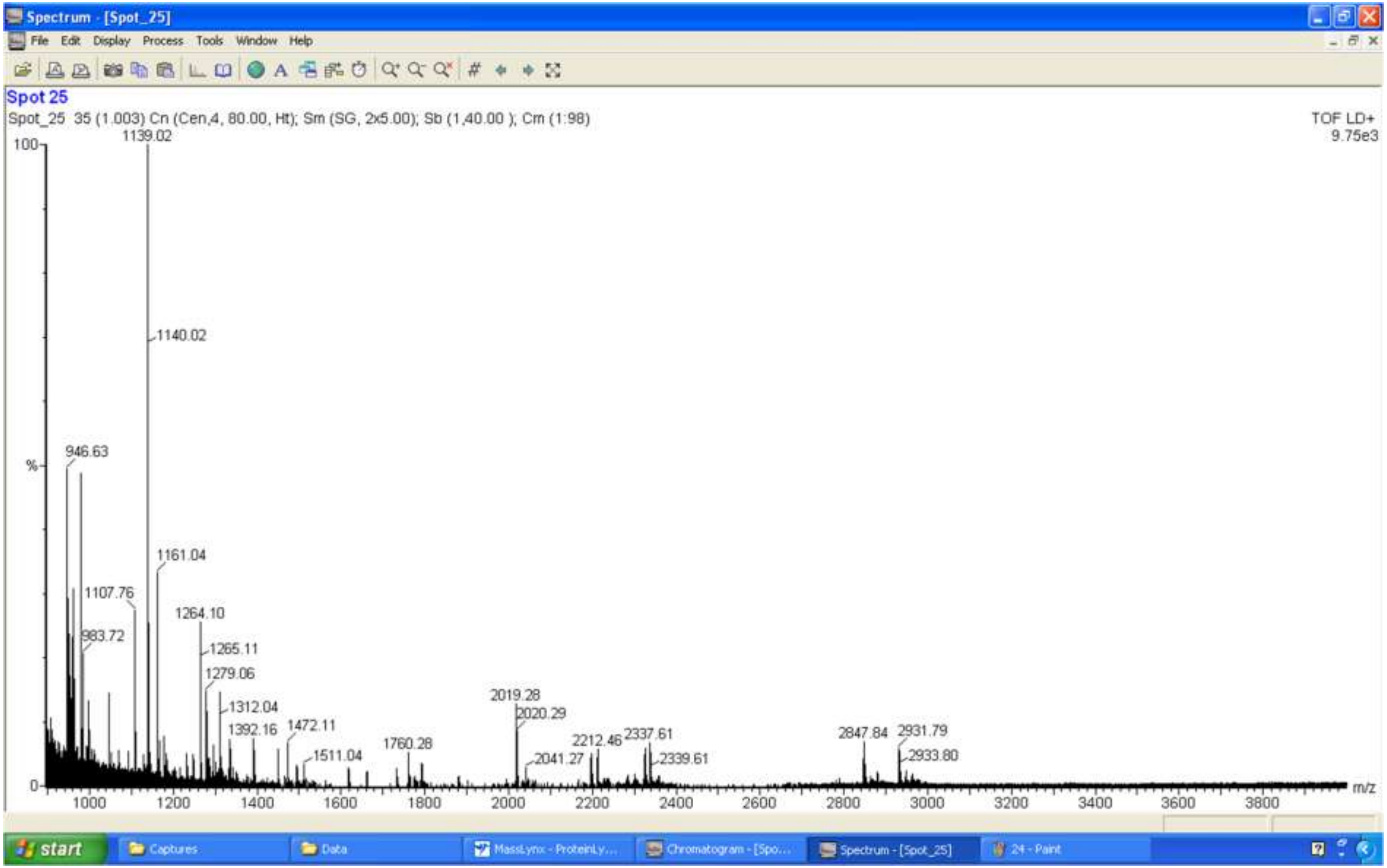
Spectrum - [Spot_25]
File Edit Display Process Tools Window Help
Spot 25
Spot_25 35 (1.003) Cn (Cen,4, 80.00, Ht); Sm (SG, 2x5.00); Sb (1,40.00); Cm (1:98)
TOF LD+
9.75e3
100
%
0
1139.02
1140.02
946.63
1161.04
1107.76
1264.10
983.72
1265.11
1279.06
1312.04
1392.16
1472.11
1511.04
1760.28
2019.28
2020.29
2041.27
2212.46
2337.61
2339.61
2847.84
2931.79
2933.80
1000
1200
1400
1600
1800
2000
2200
2400
2600
2800
3000
3200
3400
3600
3800
m/z
start
Captures
Data
MassLynx - ProteinLy...
Chromatogram - [Spo...
Spectrum - [Spot_25]
24 - Paint

Figure S1.23

Mascot Search Results Spot 27

```
User            : Paul Millares
Email           : paul.millares@gmail.com
Search title    : Spot 27
Database        : Haemonchus 210108 (6387 sequences; 918038 residues)
Timestamp       : 1 Aug 2011 at 10:40:02 GMT
Top Score       : 62 for HCP00006_1, putative nuclear encoded protein  Method: similarity and extension
```

Mascot Score Histogram

Protein score is -10*Log(P), where P is the probability that the observed match is a random event.

Protein scores greater than 51 are significant (p<0.05).

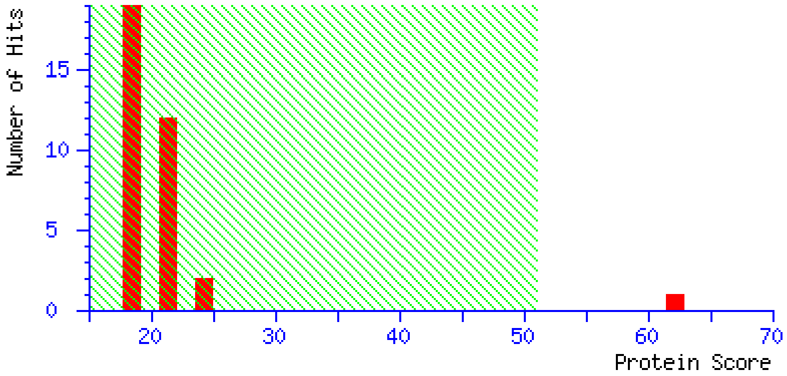

Concise Protein Summary Report

1. HCP00006_1 **Mass:** 59610 **Score:** 62 **Expect:** 0.0039 **Matches:** 13

   putative nuclear encoded protein Method: similarity and extension

   HCP00006_2 **Mass:** 59610 **Score:** 62 **Expect:** 0.0039 **Matches:** 13

   putative nuclear encoded protein Method: similarity and extension

   HCP00006_3 **Mass:** 59543 **Score:** 42 **Expect:** 0.39 **Matches:** 10

   putative nuclear encoded protein Method: similarity and extension

HCP06579_2 **Mass:** 4464 **Score:** 27 **Expect:** 12 **Matches:** 3
putative nuclear encoded protein Method: Longest ORF
HCP07804_1 **Mass:** 8558 **Score:** 20 **Expect:** 67 **Matches:** 3
putative nuclear encoded protein Method: ESTScan
HCP02052_1 **Mass:** 5211 **Score:** 19 **Expect:** 86 **Matches:** 3
putative nuclear encoded protein Method: ESTScan
HCP05631_2 **Mass:** 23359 **Score:** 19 **Expect:** 90 **Matches:** 5
putative nuclear encoded protein Method: ESTScan
HCP02648_1 **Mass:** 8149 **Score:** 18 **Expect:** 94 **Matches:** 3
putative nuclear encoded protein Method: ESTScan

---

2. HCP00998_1 **Mass:** 67689 **Score:** 25 **Expect:** 23 **Matches:** 10
putative nuclear encoded protein Method: similarity and extension
HCP07908_1 **Mass:** 7882 **Score:** 21 **Expect:** 53 **Matches:** 3
putative nuclear encoded protein Method: ESTScan
HCP04076_1 **Mass:** 12094 **Score:** 19 **Expect:** 82 **Matches:** 4
putative nuclear encoded protein Method: similarity and extension

---

Search Parameters

Type of search : Peptide Mass Fingerprint
Enzyme : Trypsin
Variable modifications : Carbamidomethyl (C),Glu->pyro-Glu (N-term E),Oxidation (M)
Mass values : Monoisotopic
Protein Mass : Unrestricted
Peptide Mass Tolerance : ± 1.2 Da
Peptide Charge State : 1+
Max Missed Cleavages : 1
Number of queries : 20

Protein View

Match to: **HCP00006_1** Score: **62** Expect: **0.0039**
putative nuclear encoded protein Method: similarity and extension

Nominal mass (M_r): **59610**; Calculated pI value: **6.67**
NCBI BLAST search of HCP00006_1 against nr
Unformatted sequence string for pasting into other applications

Variable modifications: Carbamidomethyl (C),Glu->pyro-Glu (N-term E),Oxidation (M)
Cleavage by Trypsin: cuts C-term side of KR unless next residue is P
Number of mass values searched: **20**
Number of mass values matched: **13**
Sequence Coverage: **28%**

Matched peptides shown in **Bold Red**

```
  1 MLSNLARTSG RMAFIRGISS AQMDAHAQVI DDQKPMEEQS NPSFFKMVDY
 51 YFDKGASVIE PKLVEEMKSN VMSTKDKKNL VSGILKAIKP VNKVLYITFP
101 IRRDNGEFEV IEAWRAQHSE HRTPTKGGIR YSMDVCEDEV KALSALMTYK
151 CAAVDVPFGG AKGGVKIDPK QYTDYEIEKI TRRIAIEFAK KGFLGPGVDV
201 PAPDMGTGER EMGWIADTYA QTIGHLDRDA SACITGKPIV AGGIHGRVSA
251 TGRGVWKGLE VFTKEPEYMN KVGLSLGLEG KTIIIQGFGN VGLHTMRYLH
301 RAGAKVIGVQ EWDCAVFNPD GIHPKELEDW RDENGTIKNF PKAKNFEPFA
351 ELMYEPCDIF VPAACEKAIH KENANRIQAK IIAEAANGPT TPAADKILLE
401 RGNCLIIPDM FINSGGVTVS YFEWLKNLNH VSYGRLSFKY EEDSNRMLLQ
451 SVQDSLEKAL NKEAPVHPND EFTARIAGAS EKDIVHSGLE YTMTRSGEAI
501 IRTARKYNLG LDIRTAAYAN SIEKVYNTYR TAGFTFT
```

Start - End	Observed	Mr(expt)	Mr(calc)	Delta	Miss	Sequence	
94 - 102	1122.09	1121.08	1120.66	0.42	0	K.VLYITFPIR.R	
127 - 141	1773.46	1772.46	1772.77	-0.31	1	K.GGIRYSMDVCEDEVK.A	Carbamidomethyl (C); Oxidation (M)
142 - 150	996.55	995.54	996.53	-0.99	0	K.ALSALMTYK.C	
142 - 150	997.55	996.54	996.53	0.01	0	K.ALSALMTYK.C	
183 - 190	947.79	946.79	946.56	0.23	1	R.RIAIEFAK.K	
191 - 210	2016.52	2015.52	2014.98	0.54	1	K.KGFLGPGVDVPAPDMGTGER.E	Oxidation (M)
192 - 210	1888.41	1887.40	1886.88	0.52	0	K.GFLGPGVDVPAPDMGTGER.E	Oxidation (M)
211 - 228	2093.50	2092.49	2091.97	0.52	0	R.EMGWIADTYAQTIGHLDR.D	Oxidation (M)
229 - 247	1880.49	1879.48	1878.97	0.51	0	R.DASACITGKPIVAGGIHGR.V	Carbamidomethyl (C)
272 - 281	972.81	971.80	971.57	0.24	0	K.VGLSLGLEGK.T	
306 - 325	2281.65	2280.65	2280.10	0.55	0	K.VIGVQEWDCAVFNPDGIHPK.E	Carbamidomethyl (C)
326 - 338	1605.27	1604.26	1603.75	0.51	1	K.ELEDWRDENGTIK.N	
463 - 475	1465.16	1464.16	1463.68	0.48	0	K.EAPVHPNDEFTAR.I	Glu->pyro-Glu (N-term E)

No match to: 951.52, 976.81, 988.69, 1144.16, 1448.15, 1480.15, 1497.15

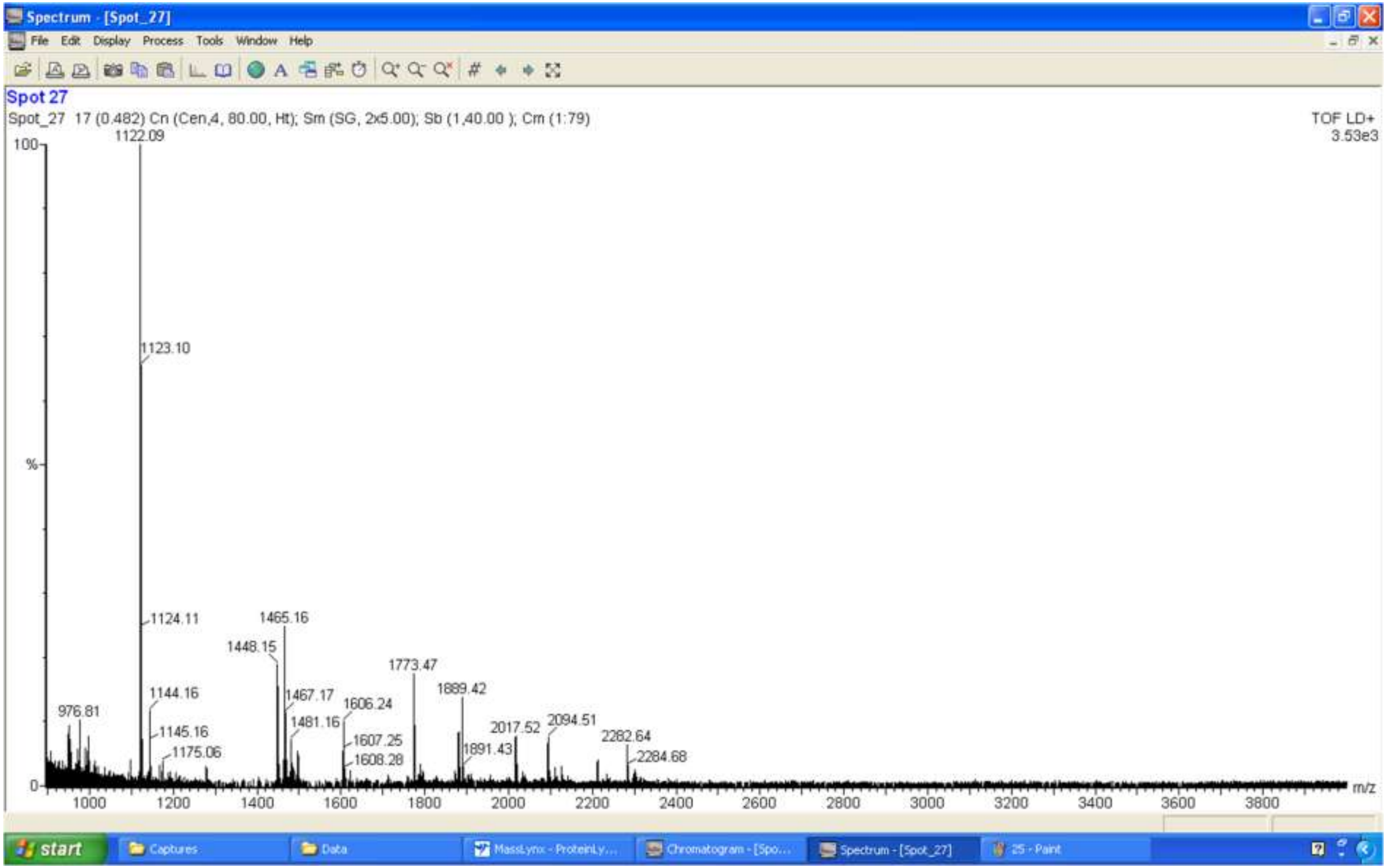
Spectrum - [Spot_27]
File Edit Display Process Tools Window Help
Spot 27
Spot_27 17 (0.482) Cn (Cen,4, 80.00, Ht); Sm (SG, 2x5.00); Sb (1,40.00); Cm (1:79)
TOF LD+
3.53e3
100
%
0
1122.09
1123.10
1124.11
1144.16
1145.16
1175.06
976.81
1448.15
1465.16
1467.17
1481.16
1606.24
1607.25
1608.28
1773.47
1889.42
1891.43
2017.52
2094.51
2282.64
2284.68
1000 1200 1400 1600 1800 2000 2200 2400 2600 2800 3000 3200 3400 3600 3800
m/z
start
Captures
Data
MassLynx - ProteinLy...
Chromatogram - [Spo...
Spectrum - [Spot_27]
25 - Paint

Figure S1.24

Mascot Search Results Spot 28

```
User            : Paul Millares
Email           : paul.millares@gmail.com
Search title    : Spot 28
Database        : Haemonchus 210108 (6387 sequences; 918038 residues)
Timestamp       : 1 Aug 2011 at 10:40:28 GMT
Top Score       : 59 for HCP00006_1, putative nuclear encoded protein  Method: similarity and extension
```

Mascot Score Histogram

Protein score is -10*Log(P), where P is the probability that the observed match is a random event.

Protein scores greater than 51 are significant (p<0.05).

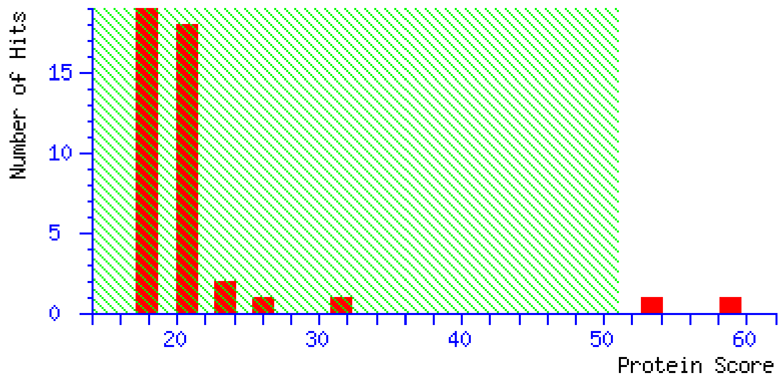

Concise Protein Summary Report

1. HCP00006_1 **Mass:** 59610 **Score:** 59 **Expect:** 0.0084 **Matches:** 26
   putative nuclear encoded protein Method: similarity and extension
   HCP00006_2 **Mass:** 59610 **Score:** 59 **Expect:** 0.0084 **Matches:** 26
   putative nuclear encoded protein Method: similarity and extension

---

2. HCP00006_3 **Mass:** 59543 **Score:** 52 **Expect:** 0.04 **Matches:** 25

putative nuclear encoded protein Method: similarity and extension

---

3. HCP06579_2 **Mass:** 4464 **Score:** 31 **Expect:** 5.2 **Matches:** 6

putative nuclear encoded protein Method: Longest ORF

Search Parameters

Type of search : Peptide Mass Fingerprint
Enzyme : Trypsin
Variable modifications : Carbamidomethyl (C),Glu->pyro-Glu (N-term E),Oxidation (M)
Mass values : Monoisotopic
Protein Mass : Unrestricted
Peptide Mass Tolerance : ± 1.2 Da
Peptide Charge State : 1+
Max Missed Cleavages : 1
Number of queries : 60

Protein View

Match to: **HCP00006_1** Score: **59** Expect: **0.0084**
putative nuclear encoded protein Method: similarity and extension

Nominal mass (M_r): **59610**; Calculated pI value: **6.67**
NCBI BLAST search of HCP00006_1 against nr
Unformatted sequence string for pasting into other applications

Variable modifications: Carbamidomethyl (C),Glu->pyro-Glu (N-term E),Oxidation (M)
Cleavage by Trypsin: cuts C-term side of KR unless next residue is P
Number of mass values searched: **60**
Number of mass values matched: **26**
Sequence Coverage: **45%**

Matched peptides shown in **Bold Red**

```
  1 MLSNLARTSG RMAFIRGISS AQMDAHAQVI DDQKPMEEQS NPSFFKMVDY
 51 YFDKGASVIE PKLVEEMKSN VMSTKDKKNL VSGILKAIKP VNKVLYITFP
101 IRRDNGEFEV IEAWRAQHSE HRTPTKGGIR YSMDVCEDEV KALSALMTYK
151 CAAVDVPFGG AKGGVKIDPK QYTDYEIEKI TRRIAIEFAK KGFLGPGVDV
201 PAPDMGTGER EMGWIADTYA QTIGHLDRDA SACITGKPIV AGGIHGRVSA
251 TGRGVWKGLE VFTKEPEYMN KVGLSLGLEG KTIIIQGFGN VGLHTMRYLH
```

```
301 RAGAKVIGVQ EWDCAVFNPD GIHPKELEDW RDENGTIKNF PKAKNFEPFA
351 ELMYEPCDIF VPAACEKAIH KENANRIQAK IIAEAANGPT TPAADKILLE
401 RGNCLIIPDM FINSGGVTVS YFEWLKNLNH VSYGRLSFKY EEDSNRMLLQ
451 SVQDSLEKAL NKEAPVHPND EFTARIAGAS EKDIVHSGLE YTMTRSGEAI
501 IRTARKYNLG LDIRTAAYAN SIEKVYNTYR TAGFTFT
```

Start - End	Observed	Mr(expt)	Mr(calc)	Delta	Miss	Sequence
47 - 54	1095.72	1094.71	1095.46	-0.75	0	K.MVDYYFDK.G Oxidation (M)
63 - 75	1496.12	1495.11	1494.74	0.37	1	K.LVEEMKSNVMSTK.D
69 - 77	1009.23	1008.22	1008.49	-0.27	1	K.SNVMSTKDK.K
87 - 102	1872.36	1871.35	1871.14	0.21	1	K.AIKPVNKVLYITFPIR.R
94 - 102	1121.05	1120.04	1120.66	-0.62	0	K.VLYITFPIR.R
94 - 103	1277.22	1276.21	1276.77	-0.55	1	K.VLYITFPIRR.D
103 - 115	1621.14	1620.13	1619.77	0.36	1	R.RDNGEFEVIEAWR.A
104 - 115	1464.10	1463.09	1463.67	-0.58	0	R.DNGEFEVIEAWR.A
127 - 141	1757.41	1756.40	1756.78	-0.37	1	K.GGIRYSMDVCEDEVK.A Carbamidomethyl (C)
131 - 150	2312.63	2311.62	2311.04	0.58	1	R.YSMDVCEDEVKALSALMTYK.C Oxidation (M)
183 - 190	947.77	946.76	946.56	0.20	1	R.RIAIEFAK.K
191 - 210	1999.50	1998.49	1998.98	-0.49	1	K.KGFLGPGVDVPAPDMGTGER.E
191 - 210	2016.45	2015.44	2014.98	0.46	1	K.KGFLGPGVDVPAPDMGTGER.E Oxidation (M)
192 - 210	1888.34	1887.33	1886.88	0.45	0	K.GFLGPGVDVPAPDMGTGER.E Oxidation (M)
211 - 228	2077.46	2076.45	2075.97	0.48	0	R.EMGWIADTYAQTIGHLDR.D
211 - 228	2093.45	2092.44	2091.97	0.47	0	R.EMGWIADTYAQTIGHLDR.D Oxidation (M)
229 - 247	1880.43	1879.42	1878.97	0.45	0	R.DASACITGKPIVAGGIHGR.V Carbamidomethyl (C)
272 - 281	972.76	971.75	971.57	0.19	0	K.VGLSLGLEGK.T
282 - 297	1772.40	1771.39	1771.94	-0.55	0	K.TIIIQGFGNVGLHTMR.Y Oxidation (M)
282 - 301	2325.47	2324.46	2325.25	-0.79	1	K.TIIIQGFGNVGLHTMRYLHR.A
306 - 325	2281.59	2280.58	2280.10	0.48	0	K.VIGVQEWDCAVFNPDGIHPK.E Carbamidomethyl (C)
306 - 325	2281.59	2280.58	2280.10	0.48	0	K.VIGVQEWDCAVFNPDGIHPK.E Carbamidomethyl (C)
326 - 338	1587.15	1586.14	1585.74	0.41	1	K.ELEDWRDENGTIK.N Glu->pyro-Glu (N-term E)
326 - 338	1605.21	1604.20	1603.75	0.45	1	K.ELEDWRDENGTIK.N
345 - 371	3112.08	3111.07	3111.45	-0.38	1	K.NFEPFAELMYEPCDIFVPAACEKAIHK.E
447 - 462	1816.38	1815.37	1815.98	-0.60	1	R.MLLQSVQDSLEKALNK.E

No match to: 976.77, 988.65, 994.75, 1015.73, 1019.75, 1034.72, 1057.69, 1077.39, 1143.11, 1165.13, 1420.10, 1447.10, 1469.10, 1480.10, 1486.10, 1637.20, 1711.53, 1789.39, 1795.39, 1811.30, 1826.32, 1903.39, 1910.36, 1931.32, 1951.47, 1955.30, 2031.52, 2109.44, 2115.44, 2124.40, 2125.45, 2140.46, 2303.60, 3311.27

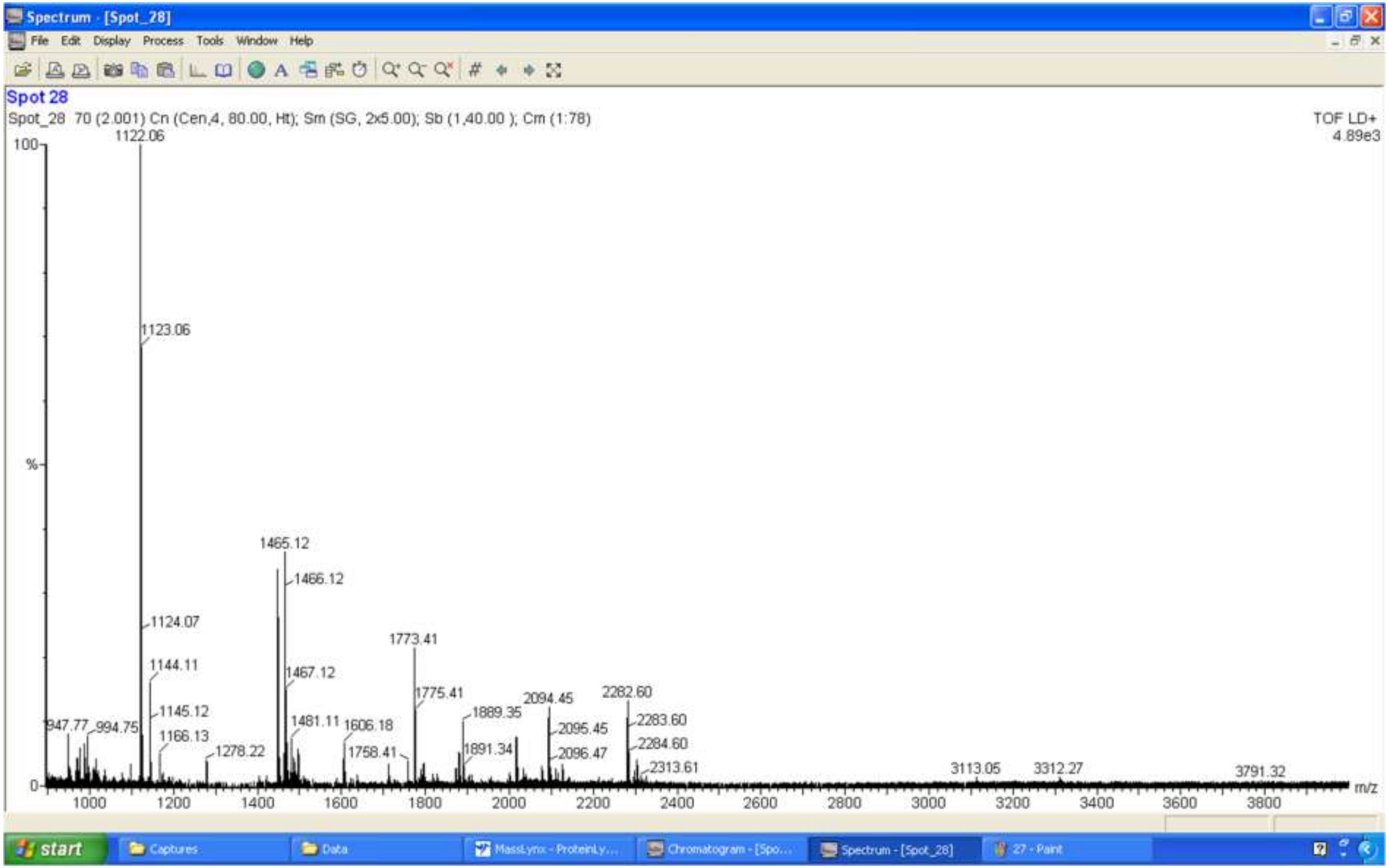
Spectrum - [Spot_28]
File Edit Display Process Tools Window Help
Spot 28
Spot_28 70 (2.001) Cn (Cen,4, 80.00, Ht); Sm (SG, 2x5.00); Sb (1,40.00); Cm (1:78)
TOF LD+
4.89e3
100
%
0
1122.06
1123.06
1124.07
1144.11
1145.12
1166.13
1278.22
947.77
994.75
1465.12
1466.12
1467.12
1481.11
1606.18
1758.41
1773.41
1775.41
1889.35
1891.34
2094.45
2095.45
2096.47
2282.60
2283.60
2284.60
2313.61
3113.05
3312.27
3791.32
1000 1200 1400 1600 1800 2000 2200 2400 2600 2800 3000 3200 3400 3600 3800
m/z
start
Captures
Data
MassLynx - ProteinLy...
Chromatogram - [Spo...
Spectrum - [Spot_28]
27 - Paint

Figure S1.25

Mascot Search Results Spot 29

```
User            : Paul Millares
Email           : paul.millares@gmail.com
Search title    : Spot 29
Database        : Haemonchus 210108 (6387 sequences; 918038 residues)
Timestamp       : 1 Aug 2011 at 10:41:31 GMT
Top Score       : 59 for HCP00006_1, putative nuclear encoded protein  Method: similarity and extension
```

Mascot Score Histogram

Protein score is -10*Log(P), where P is the probability that the observed match is a random event.

Protein scores greater than 51 are significant (p<0.05).

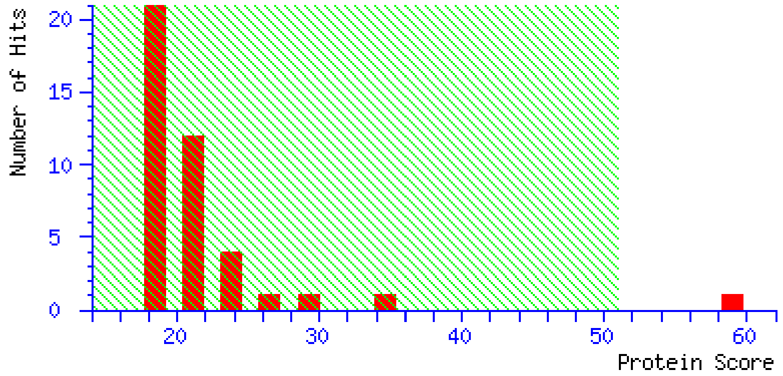

Concise Protein Summary Report

1. HCP00006_1 **Mass:** 59610 **Score:** 59 **Expect:** 0.008 **Matches:** 25
putative nuclear encoded protein Method: similarity and extension
HCP00006_2 **Mass:** 59610 **Score:** 59 **Expect:** 0.008 **Matches:** 25
putative nuclear encoded protein Method: similarity and extension
HCP00006_3 **Mass:** 59543 **Score:** 34 **Expect:** 2.7 **Matches:** 20
putative nuclear encoded protein Method: similarity and extension

2. HCP03024_2 **Mass:** 18329 **Score:** 34 **Expect:** 2.3 **Matches:** 10
putative nuclear encoded protein Method: similarity and extension

Search Parameters

Type of search : Peptide Mass Fingerprint
Enzyme : Trypsin
Variable modifications : Carbamidomethyl (C),Glu->pyro-Glu (N-term E),Oxidation (M)
Mass values : Monoisotopic
Protein Mass : Unrestricted
Peptide Mass Tolerance : ± 1.2 Da
Peptide Charge State : 1+
Max Missed Cleavages : 1
Number of queries : 52

Protein View

Match to: **HCP00006_1** Score: **59** Expect: **0.008**
putative nuclear encoded protein Method: similarity and extension

Nominal mass (M_r): **59610;** Calculated pI value: **6.67**
NCBI BLAST search of HCP00006_1 against nr
Unformatted sequence string for pasting into other applications

Variable modifications: Carbamidomethyl (C),Glu->pyro-Glu (N-term E),Oxidation (M)
Cleavage by Trypsin: cuts C-term side of KR unless next residue is P
Number of mass values searched: **52**
Number of mass values matched: **25**
Sequence Coverage: **40%**

Matched peptides shown in **Bold Red**

```
  1 MLSNLARTSG RMAFIRGISS AQMDAHAQVI DDQKPMEEQS NPSFFKMVDY
 51 YFDKGASVIE PKLVEEMKSN VMSTKDKKNL VSGILKAIKP VNKVLYITFP
101 IRRDNGEFEV IEAWRAQHSE HRTPTKGGIR YSMDVCEDEV KALSALMTYK
151 CAAVDVPFGG AKGGVKIDPK QYTDYEIEKI TRRIAIEFAK KGFLGPGVDV
201 PAPDMGTGER EMGWIADTYA QTIGHLDRDA SACITGKPIV AGGIHGRVSA
251 TGRGVWKGLE VFTKEPEYMN KVGLSLGLEG KTIIIQGFGN VGLHTMRYLH
301 RAGAKVIGVQ EWDCAVFNPD GIHPKELEDW RDENGTIKNF PKAKNFEPFA
```

```
351 ELMYEPCDIF VPAACEKAIH KENANRIQAK IIAEAANGPT TPAADKILLE
401 RGNCLIIPDM FINSGGVTVS YFEWLKNLNH VSYGRLSFKY EEDSNRMLLQ
451 SVQDSLEKAL NKEAPVHPND EFTARIAGAS EKDIVHSGLE YTMTRSGEAI
501 IRTARKYNLG LDIRTAAYAN SIEKVYNTYR TAGFTFT
```

Start - End	Observed	Mr(expt)	Mr(calc)	Delta	Miss	Sequence
94 - 102	1122.16	1121.15	1120.66	0.49	0	K.VLYITFPIR.R
94 - 103	1278.29	1277.29	1276.77	0.52	1	K.VLYITFPIRR.D
127 - 141	1700.49	1699.49	1699.75	-0.27	1	K.GGIRYSMDVCEDEVK.A
127 - 141	1717.46	1716.45	1715.75	0.70	1	K.GGIRYSMDVCEDEVK.A Oxidation (M)
127 - 141	1758.57	1757.56	1756.78	0.79	1	K.GGIRYSMDVCEDEVK.A Carbamidomethyl (C)
127 - 141	1773.61	1772.60	1772.77	-0.17	1	K.GGIRYSMDVCEDEVK.A Carbamidomethyl (C); Oxidation (M)
131 - 150	2384.71	2383.71	2384.06	-0.35	1	R.YSMDVCEDEVKALSALMTYK.C Carbamidomethyl (C); 2 Oxidation (M)
151 - 166	1476.36	1475.35	1474.76	0.59	1	K.CAAVDVPFGGAKGGVK.I
171 - 179	1189.18	1188.18	1187.53	0.64	0	K.QYTDYEIEK.I
183 - 190	947.86	946.85	946.56	0.29	1	R.RIAIEFAK.K
184 - 191	919.72	918.71	918.55	0.16	1	R.IAIEFAKK.G
191 - 210	2016.68	2015.67	2014.98	0.69	1	K.KGFLGPGVDVPAPDMGTGER.E Oxidation (M)
211 - 228	2093.70	2092.69	2091.97	0.72	0	R.EMGWIADTYAQTIGHLDR.D Oxidation (M)
229 - 247	1880.62	1879.61	1878.97	0.64	0	R.DASACITGKPIVAGGIHGR.V Carbamidomethyl (C)
272 - 281	972.82	971.81	971.57	0.25	0	K.VGLSLGLEGK.T
306 - 325	2281.85	2280.84	2280.10	0.74	0	K.VIGVQEWDCAVFNPDGIHPK.E Carbamidomethyl (C)
427 - 435	1059.84	1058.83	1058.53	0.31	0	K.NLNHVSYGR.L
440 - 446	912.66	911.66	911.36	0.29	0	K.YEEDSNR.M
447 - 458	1406.27	1405.26	1405.71	-0.45	0	R.MLLQSVQDSLEK.A Oxidation (M)
463 - 475	1465.29	1464.28	1463.68	0.60	0	K.EAPVHPNDEFTAR.I Glu->pyro-Glu (N-term E)
483 - 495	1522.34	1521.33	1520.73	0.60	0	K.DIVHSGLEYTMTR.S
483 - 495	1538.34	1537.34	1536.72	0.61	0	K.DIVHSGLEYTMTR.S Oxidation (M)
506 - 514	1090.86	1089.85	1090.61	-0.77	1	R.KYNLGLDIR.T
506 - 514	1091.95	1090.94	1090.61	0.33	1	R.KYNLGLDIR.T
507 - 514	963.81	962.80	962.52	0.29	0	K.YNLGLDIR.T

No match to: 951.63, 973.84, 989.80, 995.81, 1029.91, 1036.84, 1107.97, 1166.19, 1174.13, 1180.21, 1202.23, 1204.22, 1260.25, 1301.14, 1308.26, 1380.31, 1408.29, 1484.30, 1498.35, 1508.36, 1606.36, 1708.40, 1889.57, 1994.68, 2064.84, 2151.81, 2717.96

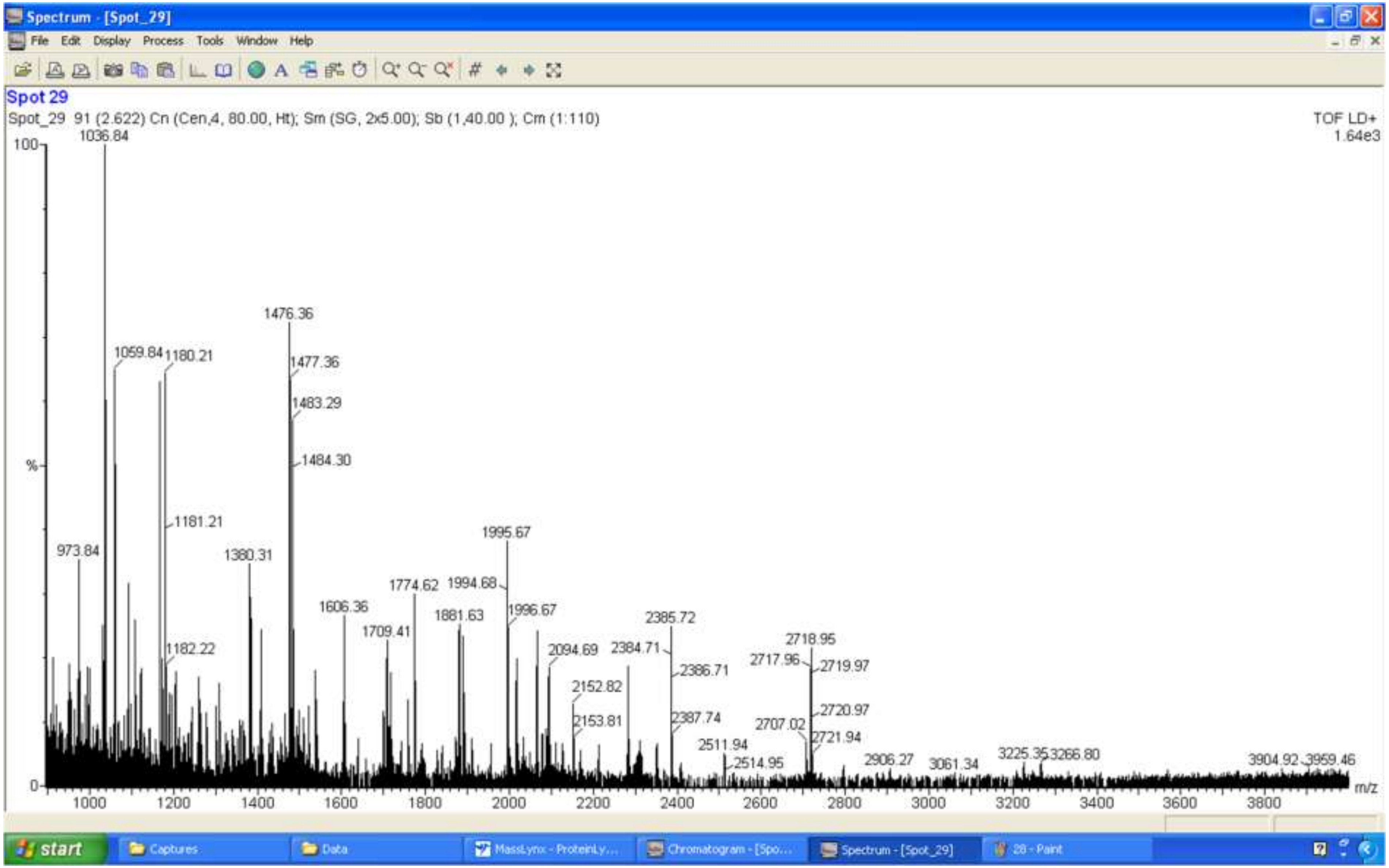

Spectrum - [Spot_29]
File Edit Display Process Tools Window Help
Spot 29
Spot_29 91 (2.622) Cn (Cen,4, 80.00, Ht); Sm (SG, 2x5.00); Sb (1,40.00); Cm (1:110)
TOF LD+
1.64e3
100
%
0
1036.84
1476.36
1059.84
1180.21
1477.36
1483.29
1484.30
1181.21
973.84
1380.31
1995.67
1774.62
1994.68
1606.36
1881.63
1996.67
1709.41
2385.72
2094.69
2384.71
2718.95
1182.22
2717.96
2719.97
2386.71
2152.82
2720.97
2153.81
2387.74
2707.02
2721.94
2511.94
2514.95
2906.27
3061.34
3225.35
3266.80
3904.92
3959.46
m/z
1000 1200 1400 1600 1800 2000 2200 2400 2600 2800 3000 3200 3400 3600 3800
start
Captures
Data
MassLynx - ProteinLy...
Chromatogram - [Spo...
Spectrum - [Spot_29]
28 - Paint

Figure S1.26

Mascot Search Results Spot 30

```
User            : Paul Millares
Email           : paul.millares@gmail.com
Search title    : Spot 30
Database        : Haemonchus 210108 (6387 sequences; 918038 residues)
Timestamp       : 1 Aug 2011 at 10:41:56 GMT
Top Score       : 42 for HCP00280_1, putative nuclear encoded protein  Method: similarity and extension
```

Mascot Score Histogram

Protein score is -10*Log(P), where P is the probability that the observed match is a random event.

Protein scores greater than 51 are significant (p<0.05).

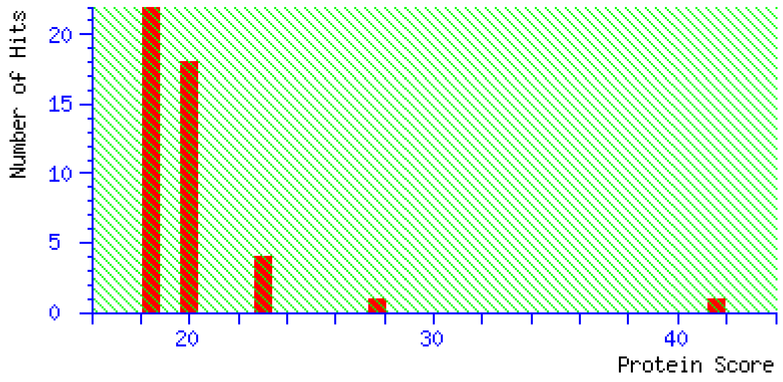

Concise Protein Summary Report

1. HCP00280_1 **Mass:** 20552 **Score:** 42 **Expect:** 0.44 **Matches:** 10

putative nuclear encoded protein Method: similarity and extension

Search Parameters

Type of search : Peptide Mass Fingerprint
Enzyme : Trypsin
Variable modifications : Carbamidomethyl (C),Glu->pyro-Glu (N-term E),Oxidation (M)
Mass values : Monoisotopic
Protein Mass : Unrestricted
Peptide Mass Tolerance : ± 1.2 Da
Peptide Charge State : 1+
Max Missed Cleavages : 1
Number of queries : 50

Protein View

Match to: **HCP00280_1** Score: **42** Expect: **0.44**
putative nuclear encoded protein Method: similarity and extension

Nominal mass (M_r): **20552**; Calculated pI value: **9.14**
NCBI BLAST search of HCP00280_1 against nr
Unformatted sequence string for pasting into other applications

Variable modifications: Carbamidomethyl (C),Glu->pyro-Glu (N-term E),Oxidation (M)
Cleavage by Trypsin: cuts C-term side of KR unless next residue is P
Number of mass values searched: **50**
Number of mass values matched: **10**
Sequence Coverage: **72%**

Matched peptides shown in **Bold Red**

```
  1 ITKIHARQIY DSRGNPTVEV DLYTEKGVFR AAVPSGASTG VHEALELRDQ
 51 DKKVHHGKGV LKAVANINDK IAPALIAKNF CVTQQRDIDQ FMLALDGTEN
101 KSNLGANAIL GVSLAVAKAG AVHKGMPLYK YIAELAGVSK VILPVPAFNV
151 INGGSHAGNK LAMQEFMILP VGATSFHEAM RMGSEVYHHL KA
```

Start - End	Observed	Mr(expt)	Mr(calc)	Delta	Miss	Sequence
1 - 7	839.30	838.29	837.52	0.77	1	-.ITKIHAR.Q
14 - 26	1465.15	1464.14	1463.71	0.43	0	R.GNPTVEVDLYTEK.G
31 - 48	1765.35	1764.34	1763.92	0.43	0	R.AAVPSGASTGVHEALELR.D
79 - 86	1052.71	1051.70	1051.49	0.21	0	K.NFCVTQQR.D Carbamidomethyl (C)
87 - 101	1726.23	1725.22	1724.79	0.43	0	R.DIDQFMLALDGTENK.S Oxidation (M)
102 - 118	1598.36	1597.35	1596.92	0.43	0	K.SNLGANAILGVSLAVAK.A
131 - 140	1050.31	1049.30	1049.58	-0.27	0	K.YIAELAGVSK.V
141 - 160	2004.55	2003.54	2003.10	0.44	0	K.VILPVPAFNVINGGSHAGNK.L

**161 - 181**	**2427.63**	**2426.62**	**2426.14**	**0.48**	**0**	**K.LAMQEFMILPVGATSFHEAMR.M**	3 Oxidation (M)
**182 - 191**	**1217.06**	**1216.06**	**1215.57**	**0.49**	**0**	**R.MGSEVYHHLK.A**	Oxidation (M)

No match to: 801.70, 807.65, 815.67, 817.67, 829.50, 845.73, 855.26, 861.28, 871.24, 877.25, 885.25, 889.76, 893.23, 901.22, 907.49, 909.20, 917.19, 933.78, 949.76, 951.48, 977.80, 997.51, 1066.28, 1072.30, 1082.25, 1089.29, 1104.33, 1140.99, 1246.05, 1262.03, 1277.54, 1293.52, 1547.19, 1713.40, 2026.53, 2211.55, 2252.50, 2264.63, 2283.63, 2443.62

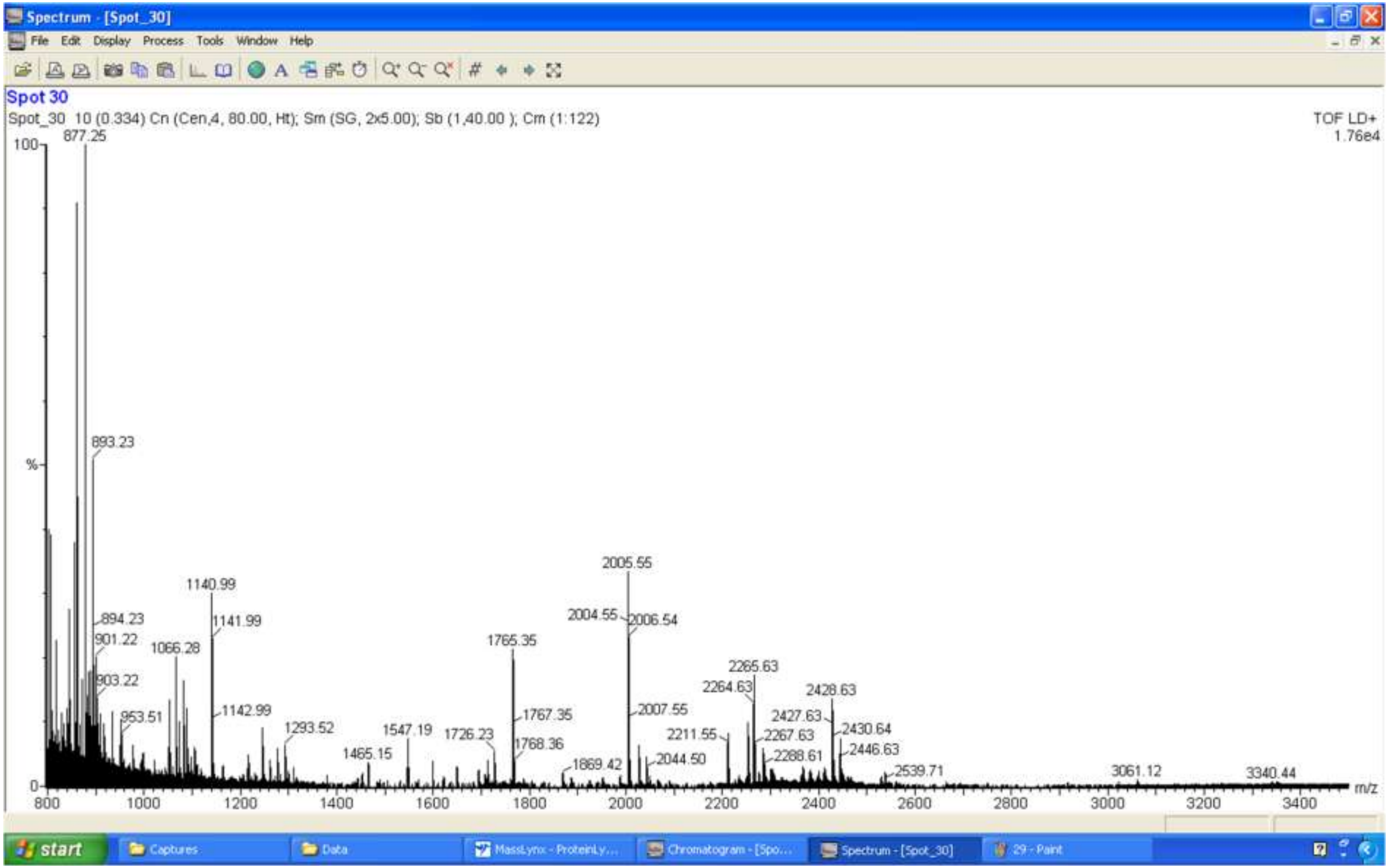
Spectrum - [Spot_30]
File Edit Display Process Tools Window Help
Spot 30
Spot_30 10 (0.334) Cn (Cen,4, 80.00, Ht); Sm (SG, 2x5.00); Sb (1,40.00); Cm (1:122)
TOF LD+
1.76e4
100
%
0
877.25
893.23
894.23
901.22
903.22
953.51
1066.28
1140.99
1141.99
1142.99
1293.52
1465.15
1547.19
1726.23
1765.35
1767.35
1768.36
1869.42
2004.55
2005.55
2006.54
2007.55
2044.50
2211.55
2264.63
2265.63
2267.63
2288.61
2427.63
2428.63
2430.64
2446.63
2539.71
3061.12
3340.44
800 1000 1200 1400 1600 1800 2000 2200 2400 2600 2800 3000 3200 3400
m/z
start
Captures
Data
MassLynx - ProteinLy...
Chromatogram - [Spo...
Spectrum - [Spot_30]
29 - Paint

Figure S1.27

Mascot Search Results Spot 32

User : Paul Millares
Email : paul.millares@gmail.com
Search title : Spot 32
Database : Haemonchus 210108 (6387 sequences; 918038 residues)
Timestamp : 1 Aug 2011 at 10:42:25 GMT
Top Score : 43 for HCP00537_1, putative nuclear encoded protein Method: similarity and extension

Mascot Score Histogram

Protein score is -10*Log(P), where P is the probability that the observed match is a random event.

Protein scores greater than 51 are significant (p<0.05).

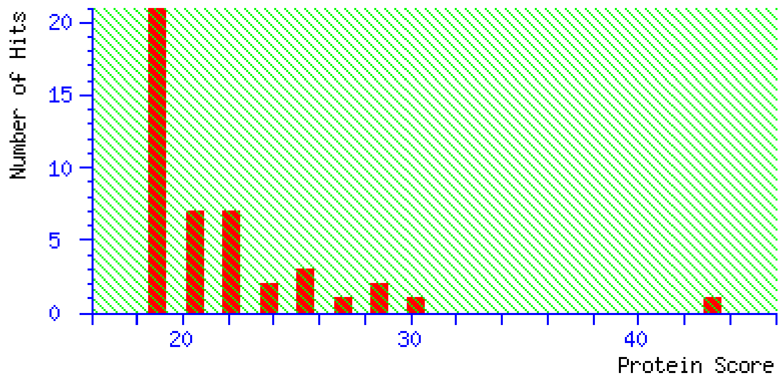

Concise Protein Summary Report

1. HCP00537_1 **Mass:** 18426 **Score:** 43 **Expect:** 0.31 **Matches:** 8

putative nuclear encoded protein Method: similarity and extension

---

Search Parameters

Type of search : Peptide Mass Fingerprint
Enzyme : Trypsin

Variable modifications : Carbamidomethyl (C),Glu->pyro-Glu (N-term E),Oxidation (M)
Mass values : **Monoisotopic**
Protein Mass : **Unrestricted**
Peptide Mass Tolerance : **± 1.2 Da**
Peptide Charge State : **1+**
Max Missed Cleavages : **1**
Number of queries : **31**

Protein View

Match to: **HCP00537_1** Score: **43** Expect: **0.31**
putative nuclear encoded protein Method: similarity and extension

Nominal mass (M_r): **18426**; Calculated pI value: **5.81**
NCBI BLAST search of HCP00537_1 against nr
Unformatted sequence string for pasting into other applications

Variable modifications: Carbamidomethyl (C),Glu->pyro-Glu (N-term E),Oxidation (M)
Cleavage by Trypsin: cuts C-term side of KR unless next residue is P
Number of mass values searched: **31**
Number of mass values matched: **8**
Sequence Coverage: **44%**

Matched peptides shown in **Bold Red**

```
  1 VNDSVTKSKF DNLYGIRESL PDGIKRATDV MLAGKVSVVC GYGDVGKGSA
 51 ASLRAFGSHV IVTEIDPINA LQAAMEGYEV TTLDEAAPRA NIVVTTTGCK
101 DIVLGRHMEL LPNDAIVCNV GHFDCEIDVK WLNENAAKKE VVKPQVDRYL
151 MKNGRHIILL AEGRLVNLGC A
```

Start - End	Observed	Mr(expt)	Mr(calc)	Delta	Miss	Sequence
10 - 17	997.37	996.37	996.50	-0.14	0	K.FDNLYGIR.E
18 - 26	1014.53	1013.52	1013.55	-0.03	1	R.ESLPDGIKR.A
26 - 35	1060.59	1059.58	1060.57	-0.99	1	K.RATDVMLAGK.V
90 - 100	1163.91	1162.90	1162.60	0.30	0	R.ANIVVTTTGCK.D Carbamidomethyl (C)
90 - 106	1760.06	1759.06	1758.97	0.09	1	R.ANIVVTTTGCKDIVLGR.H
131 - 138	946.56	945.55	944.47	1.08	0	K.WLNENAAK.K
140 - 152	1621.99	1620.98	1619.87	1.11	1	K.EVVKPQVDRYLMK.N Oxidation (M)
153 - 164	1348.87	1347.86	1347.77	0.09	1	K.NGRHIILLAEGR.L

No match to: 911.65, 929.51, 953.38, 968.52, 969.57, 989.55, 1026.58, 1045.60, 1140.83, 1143.87, 1146.83, 1149.93, 1161.95, 1179.90, 1277.95, 1332.01, 1442.97, 1496.03, 1649.02, 1694.13, 1706.05, 1713.17, 1764.10

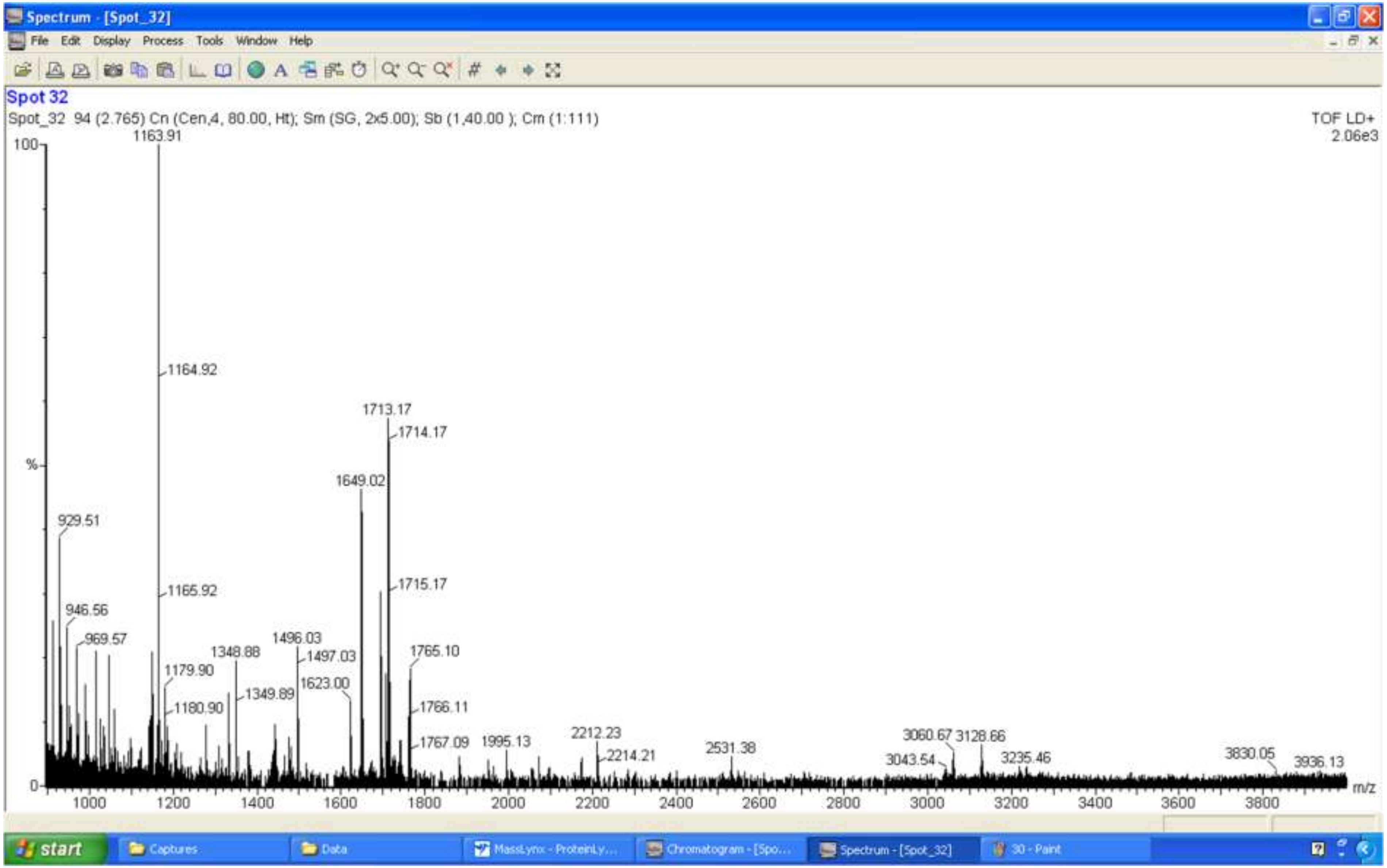

Spectrum - [Spot_32]
File Edit Display Process Tools Window Help
Spot 32
Spot_32 94 (2.765) Cn (Cen,4, 80.00, Ht); Sm (SG, 2x5.00); Sb (1,40.00); Cm (1:111)
TOF LD+
2.06e3
100
%
0
1163.91
1164.92
1165.92
1713.17
1714.17
1715.17
1649.02
929.51
946.56
969.57
1179.90
1180.90
1348.88
1349.89
1496.03
1497.03
1623.00
1765.10
1766.11
1767.09
1995.13
2212.23
2214.21
2531.38
3043.54
3060.67
3128.66
3235.46
3830.05
3936.13
1000 1200 1400 1600 1800 2000 2200 2400 2600 2800 3000 3200 3400 3600 3800
m/z
start
Captures
Data
MassLynx - ProteinLy...
Chromatogram - [Spo...
Spectrum - [Spot_32]
30 - Paint

Figure S1.28

Mascot Search Results Spot 34

```
User            : Paul Millares
Email           : paul.millares@gmail.com
Search title    : Spot 34
Database        : Haemonchus 210108 (6387 sequences; 918038 residues)
Timestamp       : 1 Aug 2011 at 10:42:52 GMT
Top Score       : 50 for HCP00183_1, putative nuclear encoded protein  Method: similarity and extension
```

Mascot Score Histogram

Protein score is -10*Log(P), where P is the probability that the observed match is a random event.

Protein scores greater than 51 are significant (p<0.05).

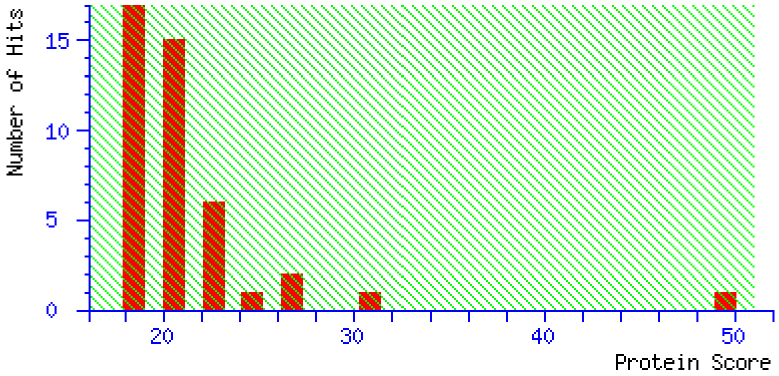

Concise Protein Summary Report

1. HCP00183_1 **Mass:** 29610 **Score:** 50 **Expect:** 0.072 **Matches:** 12
   putative nuclear encoded protein Method: similarity and extension
   HCP00183_2 **Mass:** 23326 **Score:** 41 **Expect:** 0.46 **Matches:** 10
   putative nuclear encoded protein Method: similarity and extension

Search Parameters

Type of search : Peptide Mass Fingerprint
Enzyme : Trypsin
Variable modifications : Carbamidomethyl (C),Glu->pyro-Glu (N-term E),Oxidation (M)
Mass values : Monoisotopic
Protein Mass : Unrestricted
Peptide Mass Tolerance : ± 1.2 Da
Peptide Charge State : 1+
Max Missed Cleavages : 1
Number of queries : 36

Protein View

Match to: **HCP00183_1** Score: **50** Expect: **0.072**
putative nuclear encoded protein Method: similarity and extension

Nominal mass (M_r): **29610**; Calculated pI value: **7.11**
NCBI BLAST search of HCP00183_1 against nr
Unformatted sequence string for pasting into other applications

Variable modifications: Carbamidomethyl (C),Glu->pyro-Glu (N-term E),Oxidation (M)
Cleavage by Trypsin: cuts C-term side of KR unless next residue is P
Number of mass values searched: **36**
Number of mass values matched: **12**
Sequence Coverage: **56%**

Matched peptides shown in **Bold Red**

```
  1 MAVRIERDTF GELEVPADRY YGAQTARSQM NFRIGGPEER MPLPVVHAFG
 51 ILKKAAAMVN TEYGLDQKIA DAICKAADEV TEGKLDGHFP LVTWQTGSGT
101 QSNMNVNEVI SNRAIEMLGG QLGSKKPVHP NDHVNMSQSS NDTFPTAMHI
151 AVAREINSRL LPALKQLHGS LKKKADEFKD IIKIGRTHTQ DAVPLTLGQE
201 FSGYVQQVEN GIARVEAALP RLYELAAGGT AVGTGLNTRK GFAEKVAKTV
251 ADLTGLPFKT APKQVETLAA HDAL
```

Start - End	Observed	Mr(expt)	Mr(calc)	Delta	Miss	Sequence
8 - 19	1349.06	1348.06	1347.63	0.42	0	R.DTFGELEVPADR.Y
20 - 27	929.63	928.63	928.44	0.19	0	R.YYGAQTAR.S
41 - 53	1422.23	1421.22	1420.83	0.40	0	R.MPLPVVHAFGILK.K
41 - 53	1438.25	1437.24	1436.82	0.42	0	R.MPLPVVHAFGILK.K Oxidation (M)
55 - 68	1527.13	1526.12	1525.71	0.41	0	K.AAAMVNTEYGLDQK.I Oxidation (M)
85 - 113	3218.16	3217.16	3216.53	0.63	0	K.LDGHFPLVTWQTGSGTQSNMNVNEVISNR.A Oxidation (M)

**114 - 125**	**1204.12**	**1203.11**	**1202.63**	**0.48**	**0**	**R.AIEMLGGQLGSK.K**
**114 - 125**	**1220.08**	**1219.07**	**1218.63**	**0.44**	**0**	**R.AIEMLGGQLGSK.K** Oxidation (M)
**126 - 154**	**3234.06**	**3233.06**	**3232.51**	**0.54**	**0**	**K.KPVHPNDHVNMSQSSNDTFPTAMHIAVAR.E** 2 Oxidation (M)
**175 - 183**	**1078.32**	**1077.31**	**1077.57**	**-0.26**	**1**	**K.ADEFKDIIK.I**
**222 - 239**	**1764.36**	**1763.35**	**1762.92**	**0.43**	**0**	**R.LYELAAGGTAVGTGLNTR.K**
**249 - 259**	**1162.11**	**1161.10**	**1160.64**	**0.46**	**0**	**K.TVADLTGLPFK.T**

No match to: 900.70, 907.46, 951.55, 973.59, 996.51, 1009.22, 1010.74, 1026.73, 1048.72, 1128.99, 1146.98, 1150.11, 1172.11, 1184.10, 1195.10, 1371.02, 1461.24, 1482.19, 1786.32, 1804.36, 2097.46, 2188.49, 2211.52, 3060.10

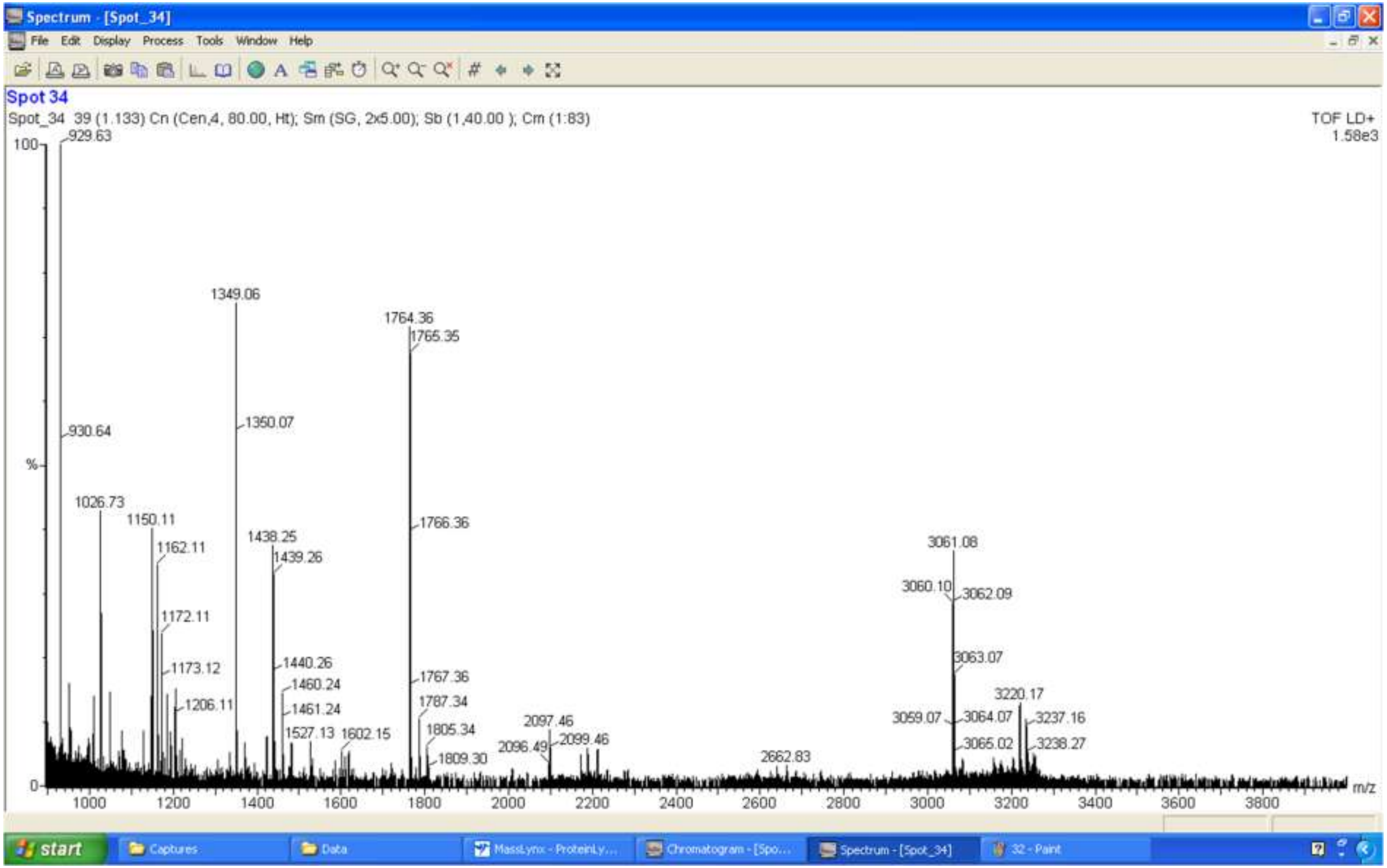

Spectrum - [Spot_34]
File Edit Display Process Tools Window Help
Spot 34
Spot_34 39 (1.133) Cn (Cen,4, 80.00, Ht); Sm (SG, 2x5.00); Sb (1,40.00); Cm (1:83)
TOF LD+
1.58e3
100
%
0
929.63
930.64
1026.73
1150.11
1162.11
1172.11
1173.12
1206.11
1349.06
1350.07
1438.25
1439.26
1440.26
1460.24
1461.24
1527.13
1602.15
1764.36
1765.35
1766.36
1767.36
1787.34
1805.34
1809.30
2096.49
2097.46
2099.46
2662.83
3059.07
3060.10
3061.08
3062.09
3063.07
3064.07
3065.02
3220.17
3237.16
3238.27
1000 1200 1400 1600 1800 2000 2200 2400 2600 2800 3000 3200 3400 3600 3800
m/z
start
Captures
Data
MassLynx - ProteinLy...
Chromatogram - [Spo...
Spectrum - [Spot_34]
32 - Paint

Figure S1.29

Mascot Search Results Spot 35

```
User               : Paul Millares
Email              : paul.millares@gmail.com
Search title       : Spot 35
Database           : Haemonchus 210108 (6387 sequences; 918038 residues)
Timestamp          : 1 Aug 2011 at 10:43:33 GMT
Top Score          : 48 for HCP00183_1, putative nuclear encoded protein  Method: similarity and extension
```

Mascot Score Histogram

Protein score is -10*Log(P), where P is the probability that the observed match is a random event.

Protein scores greater than 51 are significant (p<0.05).

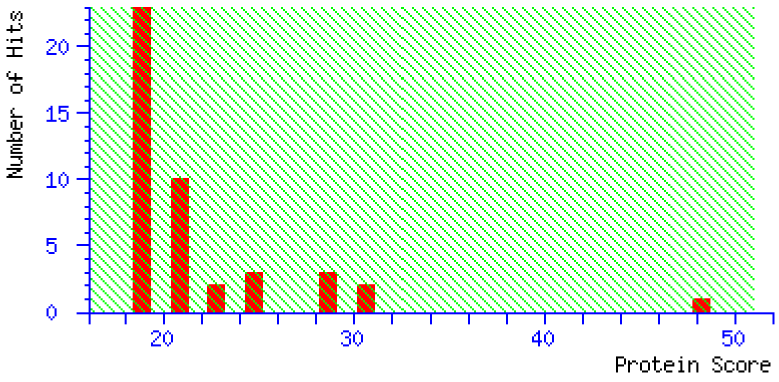

Concise Protein Summary Report

1. HCP00183_1 **Mass:** 29610 **Score:** 48 **Expect:** 0.094 **Matches:** 15

   putative nuclear encoded protein Method: similarity and extension

   HCP00183_2 **Mass:** 23326 **Score:** 37 **Expect:** 1.2 **Matches:** 12

   putative nuclear encoded protein Method: similarity and extension

Search Parameters

Type of search : Peptide Mass Fingerprint
Enzyme : Trypsin
Variable modifications : Carbamidomethyl (C),Glu->pyro-Glu (N-term E),Oxidation (M)
Mass values : Monoisotopic
Protein Mass : Unrestricted
Peptide Mass Tolerance : ± 1.2 Da
Peptide Charge State : 1+
Max Missed Cleavages : 1
Number of queries : 71

Protein View

Match to: **HCP00183_1** Score: **48** Expect: **0.094**
putative nuclear encoded protein Method: similarity and extension

Nominal mass (M_r): **29610**; Calculated pI value: **7.11**
NCBI BLAST search of HCP00183_1 against nr
Unformatted sequence string for pasting into other applications

Variable modifications: Carbamidomethyl (C),Glu->pyro-Glu (N-term E),Oxidation (M)
Cleavage by Trypsin: cuts C-term side of KR unless next residue is P
Number of mass values searched: **71**
Number of mass values matched: **15**
Sequence Coverage: **68%**

Matched peptides shown in **Bold Red**

```
  1 MAVRIERDTF GELEVPADRY YGAQTARSQM NFRIGGPEER MPLPVVHAFG
 51 ILKKAAAMVN TEYGLDQKIA DAICKAADEV TEGKLDGHFP LVTWQTGSGT
101 QSNMNVNEVI SNRAIEMLGG QLGSKKPVHP NDHVNMSQSS NDTFPTAMHI
151 AVAREINSRL LPALKQLHGS LKKKADEFKD IIKIGRTHTQ DAVPLTLGQE
201 FSGYVQQVEN GIARVEAALP RLYELAAGGT AVGTGLNTRK GFAEKVAKTV
251 ADLTGLPFKT APKQVETLAA HDAL
```

Start - End	Observed	Mr(expt)	Mr(calc)	Delta	Miss	Sequence
1 - 7	889.76	888.76	889.48	-0.72	1	-.MAVRIER.D Oxidation (M)
5 - 19	1746.36	1745.35	1745.86	-0.51	1	R.IERDTFGELEVPADR.Y
8 - 19	1349.09	1348.08	1347.63	0.45	0	R.DTFGELEVPADR.Y

20 - 27	929.66	928.66	928.44	0.22	0	R.YYGAQTAR.S
41 - 53	1438.26	1437.25	1436.82	0.43	0	R.MPLPVVHAFGILK.K Oxidation (M)
41 - 54	1566.36	1565.35	1564.92	0.43	1	R.MPLPVVHAFGILKK.A Oxidation (M)
55 - 68	1527.17	1526.17	1525.71	0.46	0	K.AAAMVNTEYGLDQK.I Oxidation (M)
85 - 113	3218.22	3217.21	3216.53	0.69	0	K.LDGHFPLVTWQTGSGTQSNMNVNEVISNR.A Oxidation (M)
114 - 125	1204.14	1203.14	1202.63	0.50	0	R.AIEMLGGQLGSK.K
114 - 125	1220.11	1219.11	1218.63	0.48	0	R.AIEMLGGQLGSK.K Oxidation (M)
126 - 154	3234.23	3233.22	3232.51	0.71	0	K.KPVHPNDHVNMSQSSNDTFPTAMHIAVAR.E 2 Oxidation (M)
180 - 186	815.48	814.47	813.51	0.96	1	K.DIIKIGR.T
187 - 214	3059.15	3058.14	3057.52	0.63	0	R.THTQDAVPLTLGQEFSGYVQQVENGIAR.V
222 - 239	1764.38	1763.37	1762.92	0.45	0	R.LYELAAGGTAVGTGLNTR.K
249 - 259	1162.13	1161.13	1160.64	0.48	0	K.TVADLTGLPFK.T

No match to: 801.71, 808.47, 809.47, 817.67, 825.33, 831.53, 833.30, 839.31, 841.30, 847.46, 853.51, 855.28, 861.68, 862.30, 868.48, 870.75, 871.25, 877.27, 883.26, 893.25, 899.49, 901.24, 908.50, 914.50, 915.51, 925.52, 935.51, 951.50, 967.53, 994.42, 996.51, 1030.33, 1044.31, 1050.33, 1060.29, 1066.30, 1072.31, 1082.28, 1089.30, 1098.31, 1106.35, 1146.99, 1150.11, 1271.55, 1277.57, 1293.54, 1309.51, 1454.25, 1482.55, 1581.38, 1930.41, 2099.48, 2211.56, 2283.64, 2464.88, 3254.31

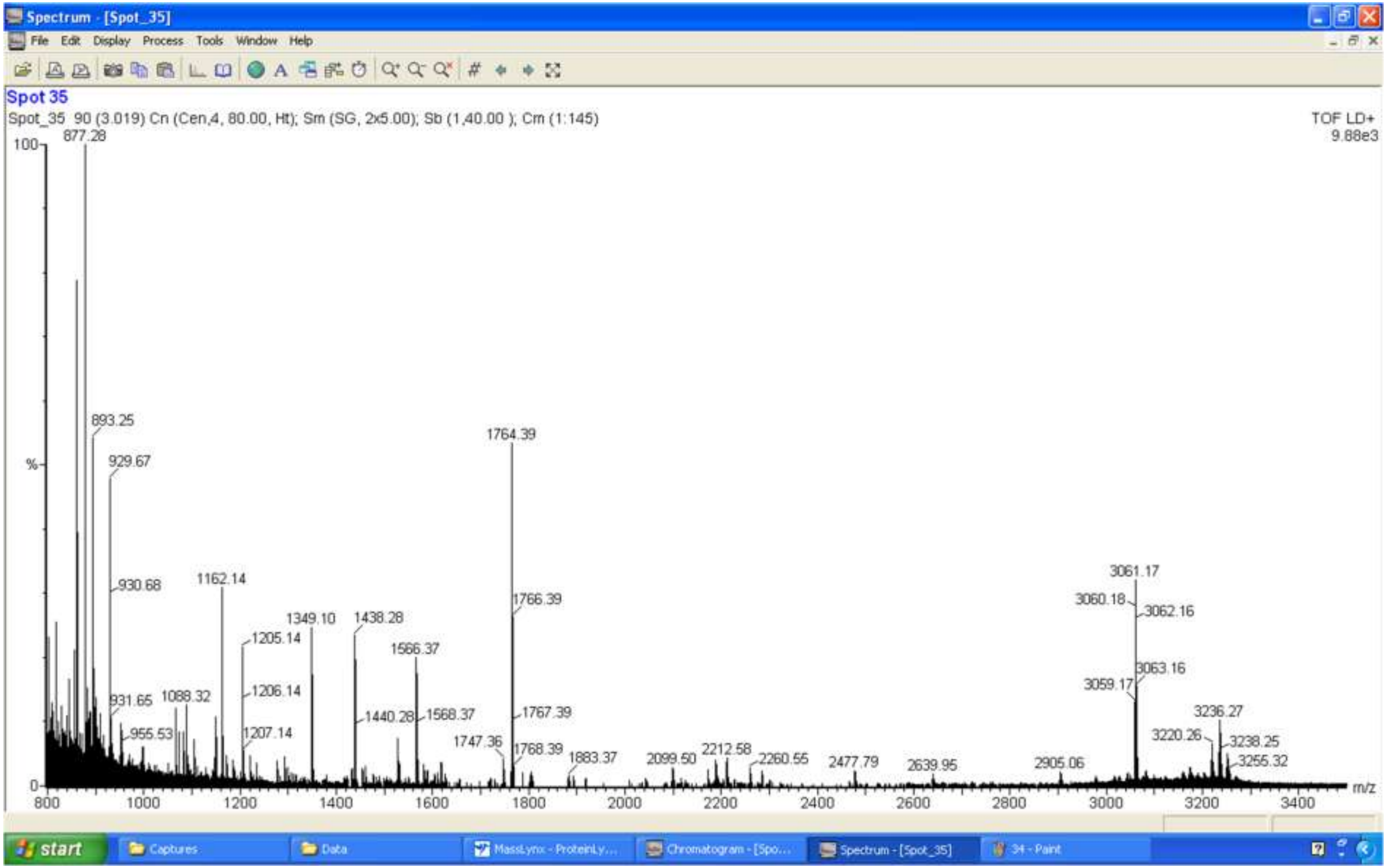
Spectrum - [Spot_35]
File Edit Display Process Tools Window Help
Spot 35
Spot_35 90 (3.019) Cn (Cen,4, 80.00, Ht); Sm (SG, 2x5.00); Sb (1,40.00); Cm (1:145)
TOF LD+
9.88e3
100
%
0
877.28
893.25
929.67
930.68
931.65
955.53
1088.32
1162.14
1205.14
1206.14
1207.14
1349.10
1438.28
1440.28
1566.37
1568.37
1747.36
1764.39
1766.39
1767.39
1768.39
1883.37
2099.50
2212.58
2260.55
2477.79
2639.95
2905.06
3059.17
3060.18
3061.17
3062.16
3063.16
3220.26
3236.27
3238.25
3255.32
800
1000
1200
1400
1600
1800
2000
2200
2400
2600
2800
3000
3200
3400
m/z
start
Captures
Data
MassLynx - ProteinLy...
Chromatogram - [Spo...
Spectrum - [Spot_35]
34 - Paint

Figure S1.30

Mascot Search Results Spot 37

```
User                : Paul Millares
Email               : paul.millares@gmail.com
Search title        : Spot 37
Database            : Haemonchus 210108 (6387 sequences; 918038 residues)
Timestamp           : 1 Aug 2011 at 10:44:05 GMT
Top Score           : 37 for HCP01607_2, putative nuclear encoded protein  Method: similarity and extension
```

Mascot Score Histogram

Protein score is -10*Log(P), where P is the probability that the observed match is a random event.

Protein scores greater than 51 are significant (p<0.05).

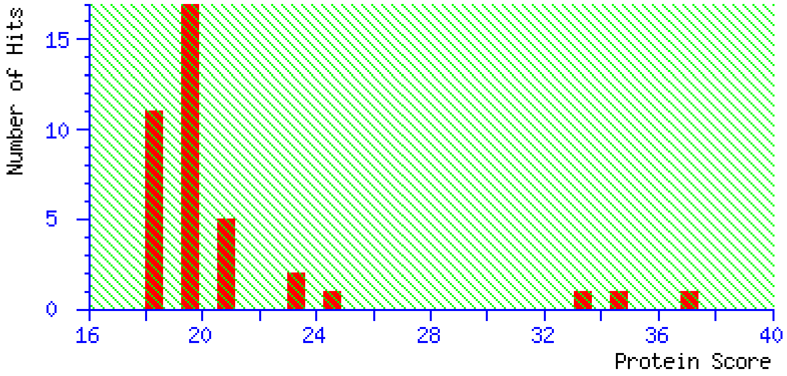

Concise Protein Summary Report

1. HCP01607_2 **Mass:** 39138 **Score:** 37 **Expect:** 1.2 **Matches:** 9

   putative nuclear encoded protein Method: similarity and extension

   HCP01607_3 **Mass:** 25355 **Score:** 32 **Expect:** 4 **Matches:** 7

   putative nuclear encoded protein Method: similarity and extension

HCP01607_1 **Mass:** 20882 **Score:** 19 **Expect:** 75 **Matches:** 4

putative nuclear encoded protein Method: similarity and extension

---

Search Parameters

Type of search : Peptide Mass Fingerprint
Enzyme : Trypsin
Variable modifications : Carbamidomethyl (C),Glu->pyro-Glu (N-term E),Oxidation (M)
Mass values : Monoisotopic
Protein Mass : Unrestricted
Peptide Mass Tolerance : ± 1.2 Da
Peptide Charge State : 1+
Max Missed Cleavages : 1
Number of queries : 31

Protein View

Match to: **HCP01607_2** Score: **37** Expect: **1.2**
putative nuclear encoded protein Method: similarity and extension

Nominal mass (M_r): **39138**; Calculated pI value: **7.83**
NCBI BLAST search of HCP01607_2 against nr
Unformatted sequence string for pasting into other applications

Variable modifications: Carbamidomethyl (C),Glu->pyro-Glu (N-term E),Oxidation (M)
Cleavage by Trypsin: cuts C-term side of KR unless next residue is P
Number of mass values searched: **31**
Number of mass values matched: **9**
Sequence Coverage: **33%**

Matched peptides shown in **Bold Red**

1 AEVVGSFKDS LTQAQKDELS AIAQKIVADG KGILAADEST GTIGKR**LSAI**
51 **NLENNETNR**Q KYRQLLFTTP NLGEHISGVI LFEETFHQST DKGEKFVDLL
101 KKQGVIPGIK **VDLGVVPLAG TIGETTTQGL DNLAQR**AAVF K**KGGCGFAK**W
151 RCVLNIGPHT PSHLAMLENA NVLARYASIC QANGLVPIVE PEVLCDGDHD
201 IHRAQK**VTEQ VLAYTYK**ALA DHHVYLEGTL LKPNMVTPGQ SCPHK**VSHEE**
251 **IGLATVTALR RTVPAAVPGI TFLSGGQSEL DATANLHAIN NAK**LLKPWKL
301 SFSYGR**ALQA SVLK**AWQGK**D ENVQAAQR**VF LHRAKANGTA AMGKYEGEDA
351 AGAAAESLFV AKHAY

Start - End	Observed	Mr(expt)	Mr(calc)	Delta	Miss	Sequence
47 - 59	1488.21	1487.20	1486.74	0.47	0	R.LSAINLENNETNR.Q
111 - 136	2638.97	2637.96	2637.40	0.56	0	K.VDLGVVPLAGTIGETTTQGLDNLAQR.A
142 - 149	825.33	824.33	823.40	0.93	1	K.KGGCGFAK.W Carbamidomethyl (C)
207 - 217	1315.17	1314.16	1313.69	0.47	0	K.VTEQVLAYTYK.A
246 - 260	1596.35	1595.34	1594.87	0.48	0	K.VSHEEIGLATVTALR.R
246 - 261	1752.45	1751.45	1750.97	0.48	1	K.VSHEEIGLATVTALRR.T
262 - 293	3178.37	3177.36	3176.65	0.71	0	R.TVPAAVPGITFLSGGQSELDATANLHAINNAK.L
307 - 314	829.65	828.64	828.51	0.13	0	R.ALQASVLK.A
320 - 328	1030.72	1029.72	1029.48	0.23	0	K.DENVQAAQR.V

No match to: 801.72, 839.33, 841.31, 855.29, 861.31, 868.32, 877.28, 885.28, 894.25, 901.25, 951.51, 1050.34, 1066.31, 1072.33, 1088.32, 1131.07, 1277.59, 1326.17, 1342.17, 1380.14, 1536.33, 1611.37

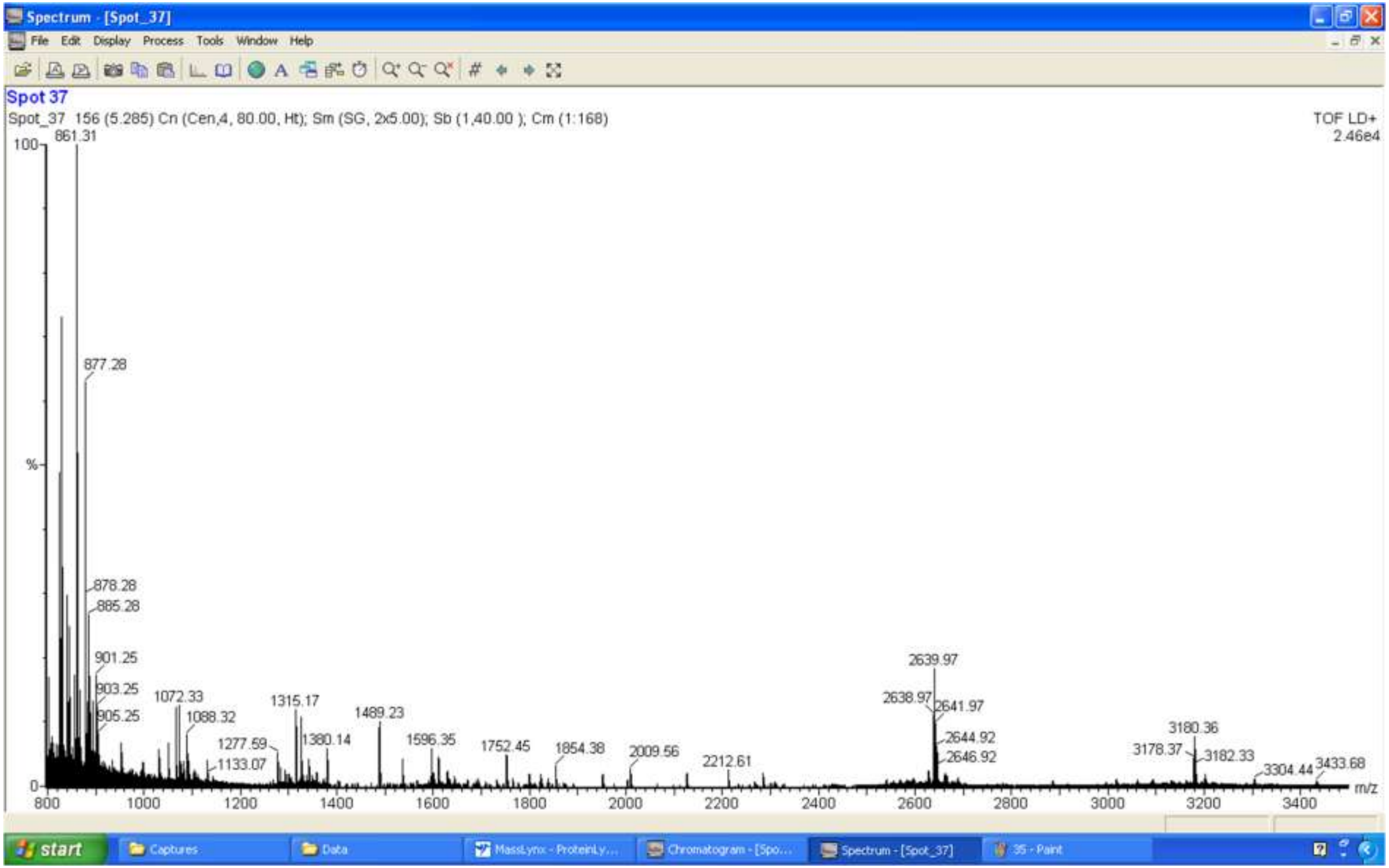
Spectrum - [Spot_37]
File Edit Display Process Tools Window Help
Spot 37
Spot_37 156 (5.285) Cn (Cen,4, 80.00, Ht); Sm (SG, 2x5.00); Sb (1,40.00); Cm (1:168)
TOF LD+
2.46e4
100
%
0
861.31
877.28
878.28
885.28
901.25
903.25
905.25
1072.33
1088.32
1133.07
1277.59
1315.17
1380.14
1489.23
1596.35
1752.45
1854.38
2009.56
2212.61
2638.97
2639.97
2641.97
2644.92
2646.92
3178.37
3180.36
3182.33
3304.44
3433.68
m/z
800 1000 1200 1400 1600 1800 2000 2200 2400 2600 2800 3000 3200 3400
start
Captures
Data
MassLynx - ProteinLy...
Chromatogram - [Spo...
Spectrum - [Spot_37]
35 - Paint

Figure S1.31

Mascot Search Results Spot 40

```
User            : Paul Millares
Email           : paul.millares@gmail.com
Search title    : Spot 40
Database        : Haemonchus 210108 (6387 sequences; 918038 residues)
Timestamp       : 1 Aug 2011 at 10:44:36 GMT
Top Score       : 51 for HCP11007_1, putative nuclear encoded protein  Method: similarity and extension
```

Mascot Score Histogram

Protein score is -10*Log(P), where P is the probability that the observed match is a random event.

Protein scores greater than 51 are significant (p<0.05).

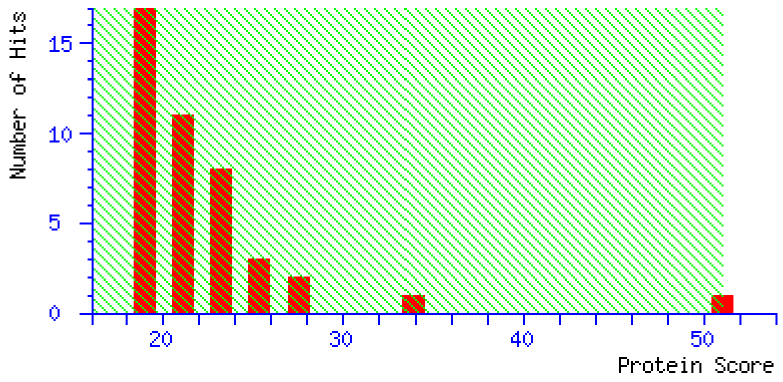

Concise Protein Summary Report

1. HCP11007_1 **Mass:** 23857 **Score:** 51 **Expect:** 0.05 **Matches:** 10
putative nuclear encoded protein Method: similarity and extension

---

2. HCP01876_1 **Mass:** 18540 **Score:** 33 **Expect:** 3.1 **Matches:** 8
putative nuclear encoded protein Method: similarity and extension

---

Search Parameters

Type of search : Peptide Mass Fingerprint
Enzyme : Trypsin
Variable modifications : Carbamidomethyl (C),Glu->pyro-Glu (N-term E),Oxidation (M)
Mass values : Monoisotopic
Protein Mass : Unrestricted
Peptide Mass Tolerance : ± 1.2 Da
Peptide Charge State : 1+
Max Missed Cleavages : 1
Number of queries : 37

Protein View

Match to: **HCP11007_1** Score: **51** Expect: **0.05**
putative nuclear encoded protein Method: similarity and extension

Nominal mass (M_r): **23857**; Calculated pI value: **8.12**
NCBI BLAST search of HCP11007_1 against nr
Unformatted sequence string for pasting into other applications

Variable modifications: Carbamidomethyl (C),Glu->pyro-Glu (N-term E),Oxidation (M)
Cleavage by Trypsin: cuts C-term side of KR unless next residue is P
Number of mass values searched: **37**
Number of mass values matched: **10**
Sequence Coverage: **62%**

Matched peptides shown in **Bold Red**

```
  1 VAAFSNGLRS LPHSSSQPKV TLIGASGGIG QPLGLLLKQD NLVKHLALYD
 51 VVGTAGVAAD LSHIDTNAKV TAHTGPKELA AAVANADVIV IPAGVPRKPG
101 MTRDDLFNTN AGIVRDIVDV IAVEAPKAMI AIITNPVNST VPIASEVMKK
151 HGVYDKRRIF GVTTLDVVRA QAFVAELKGL DATKTVIPVV GGHAGTTIIP
201 LLSQVTPKVN FTEDEIMKLT PKIQDAGTE
```

Start - End	Observed	Mr(expt)	Mr(calc)	Delta	Miss	Sequence
20 - 38	1807.35	1806.34	1806.10	0.24	0	K.VTLIGASGGIGQPLGLLLK.Q
45 - 69	2551.54	2550.53	2550.31	0.22	0	K.HLALYDVVGTAGVAADLSHIDTNAK.V
78 - 97	1945.16	1944.16	1945.10	-0.94	0	K.ELAAAVANADVIVIPAGVPR.K
104 - 115	1334.97	1333.96	1333.66	0.30	0	R.DDLFNTNAGIVR.D
116 - 127	1269.03	1268.02	1267.70	0.32	0	R.DIVDVIAVEAPK.A
158 - 169	1376.09	1375.08	1374.80	0.28	1	R.RIFGVTTLDVVR.A
159 - 169	1220.05	1219.04	1218.70	0.34	0	R.IFGVTTLDVVR.A

**170 - 178**	**976.65**	**975.64**	**975.54**	**0.10**	**0**	**R.AQAFVAELK.G**
**185 - 208**	**2398.62**	**2397.62**	**2397.40**	**0.22**	**0**	**K.TVIPVVGGHAGTTIIPLLSQVTPK.V**
**209 - 218**	**1242.01**	**1241.01**	**1240.56**	**0.44**	**0**	**K.VNFTEDEIMK.L** Oxidation (M)

No match to: 900.39, 907.38, 916.66, 922.63, 951.37, 972.69, 998.62, 1020.70, 1025.69, 1081.63, 1291.00, 1357.94, 1378.96, 1488.06, 1542.06, 1563.15, 1722.11, 1744.10, 1830.32, 1846.33, 1923.18, 1931.31, 1947.33, 1969.31, 1991.26, 2421.60, 2577.56

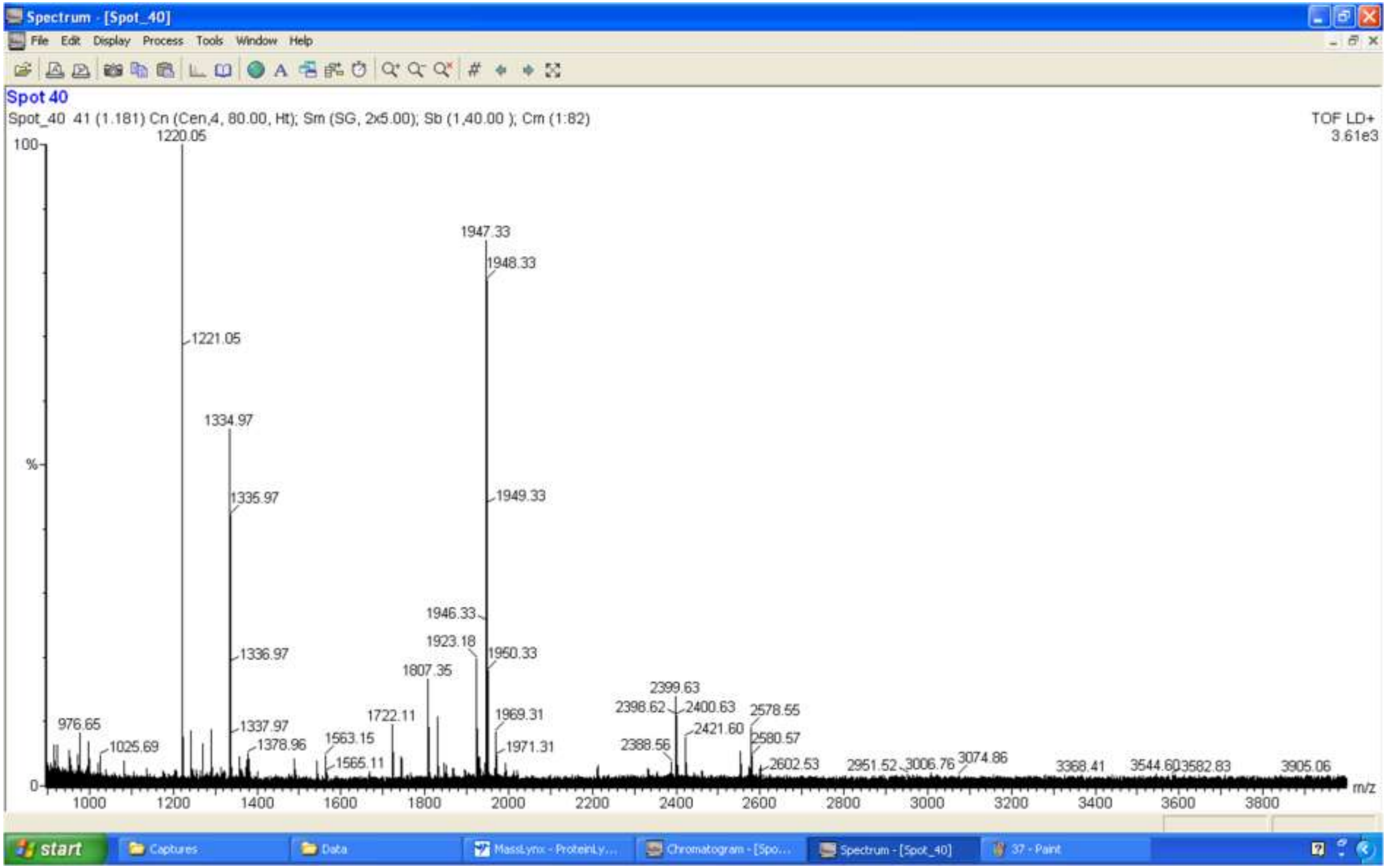

Spectrum - [Spot_40]
Spot 40
Spot_40 41 (1.181) Cn (Cen,4, 80.00, Ht); Sm (SG, 2x5.00); Sb (1,40.00); Cm (1:82)
TOF LD+
3.61e3
1220.05
1221.05
1334.97
1335.97
1336.97
1337.97
1378.96
976.65
1025.69
1563.15
1565.11
1722.11
1807.35
1923.18
1946.33
1947.33
1948.33
1949.33
1950.33
1969.31
1971.31
2388.56
2398.62
2399.63
2400.63
2421.60
2578.55
2580.57
2602.53
2951.52
3006.76
3074.86
3368.41
3544.60
3582.83
3905.06
m/z
%

Figure S1.32

Mascot Search Results Spot 41

```
User          : Paul Millares
Email         : paul.millares@gmail.com
Search title  : Spot 41
Database      : Haemonchus 210108 (6387 sequences; 918038 residues)
Timestamp     : 1 Aug 2011 at 10:45:15 GMT
Top Score     : 64 for HCP11007_1, putative nuclear encoded protein  Method: similarity and extension
```

Mascot Score Histogram

Protein score is -10*Log(P), where P is the probability that the observed match is a random event.

Protein scores greater than 51 are significant (p<0.05).

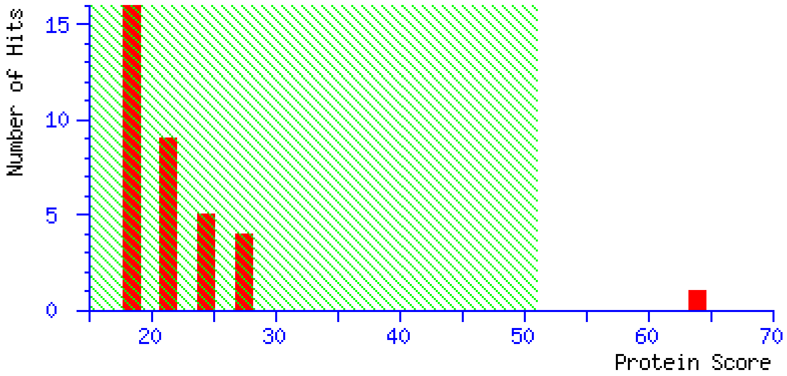

Concise Protein Summary Report

1. HCP11007_1 **Mass:** 23857 **Score:** **64** **Expect:** 0.0025 **Matches:** 9
   putative nuclear encoded protein Method: similarity and extension
   HCP08807_1 **Mass:** 10383 **Score:** 20 **Expect:** 72 **Matches:** 3
   putative nuclear encoded protein Method: ESTScan
   HCP07849_1 **Mass:** 13755 **Score:** 18 **Expect:** 92 **Matches:** 3
   putative nuclear encoded protein Method: similarity and extension

HCP01229_1 **Mass:** 13782 **Score:** 18 **Expect:** 92 **Matches:** 3

putative nuclear encoded protein Method: similarity and extension

---

2. HCP06437_2 **Mass:** 18225 **Score:** 29 **Expect:** 8.6 **Matches:** 5

putative nuclear encoded protein Method: similarity and extension

---

Search Parameters

Type of search : Peptide Mass Fingerprint
Enzyme : Trypsin
Variable modifications : Carbamidomethyl (C),Glu->pyro-Glu (N-term E),Oxidation (M)
Mass values : Monoisotopic
Protein Mass : Unrestricted
Peptide Mass Tolerance : ± 1.2 Da
Peptide Charge State : 1+
Max Missed Cleavages : 1
Number of queries : 22

Protein View

Match to: **HCP11007_1** Score: **64** Expect: **0.0025**
putative nuclear encoded protein Method: similarity and extension

Nominal mass (M_r): **23857**; Calculated pI value: **8.12**
NCBI BLAST search of HCP11007_1 against nr
Unformatted sequence string for pasting into other applications

Variable modifications: Carbamidomethyl (C),Glu->pyro-Glu (N-term E),Oxidation (M)
Cleavage by Trypsin: cuts C-term side of KR unless next residue is P
Number of mass values searched: **22**
Number of mass values matched: **9**
Sequence Coverage: **62%**

Matched peptides shown in **Bold Red**

1 VAAFSNGLRS LPHSSSQPK**V TLIGASGGIG QPLGLLLK**QD NLVK**HLALYD**
51 VVGTAGVAAD LSHIDTNAKV TAHTGPK**ELA AAVANADVIV IPAGVPR**KPG
101 MTR**DDLFNTN AGIVRDIVDV IAVEAPK**AMI AIITNPVNST VPIASEVMKK
151 HGVYDKRR**IF GVTTLDVVRA QAFVAELK**GL DATK**TVIPVV GGHAGTTIIP**
201 LLSQVTPKVN FTEDEIMKLT PKIQDAGTE

Start - End	Observed	Mr(expt)	Mr(calc)	Delta	Miss	Sequence
20 - 38	1807.51	1806.50	1806.10	0.41	0	K.VTLIGASGGIGQPLGLLLK.Q
45 - 69	2551.76	2550.75	2550.31	0.45	0	K.HLALYDVVGTAGVAADLSHIDTNAK.V
78 - 97	1946.50	1945.49	1945.10	0.39	0	K.ELAAAVANADVIVIPAGVPR.K
104 - 115	1335.09	1334.08	1333.66	0.42	0	R.DDLFNTNAGIVR.D
116 - 127	1269.15	1268.14	1267.70	0.44	0	R.DIVDVIAVEAPK.A
159 - 169	1220.16	1219.16	1218.70	0.46	0	R.IFGVTTLDVVR.A
170 - 178	976.73	975.73	975.54	0.19	0	R.AQAFVAELK.G
185 - 208	2398.83	2397.82	2397.40	0.42	0	K.TVIPVVGGHAGTTIIPLLSQVTPK.V
209 - 218	1242.12	1241.11	1240.56	0.55	0	K.VNFTEDEIMK.L Oxidation (M)

No match to: 900.73, 916.74, 922.71, 951.44, 972.77, 997.47, 1025.80, 1291.11, 1542.19, 1563.31, 1722.27, 1829.47, 1923.36

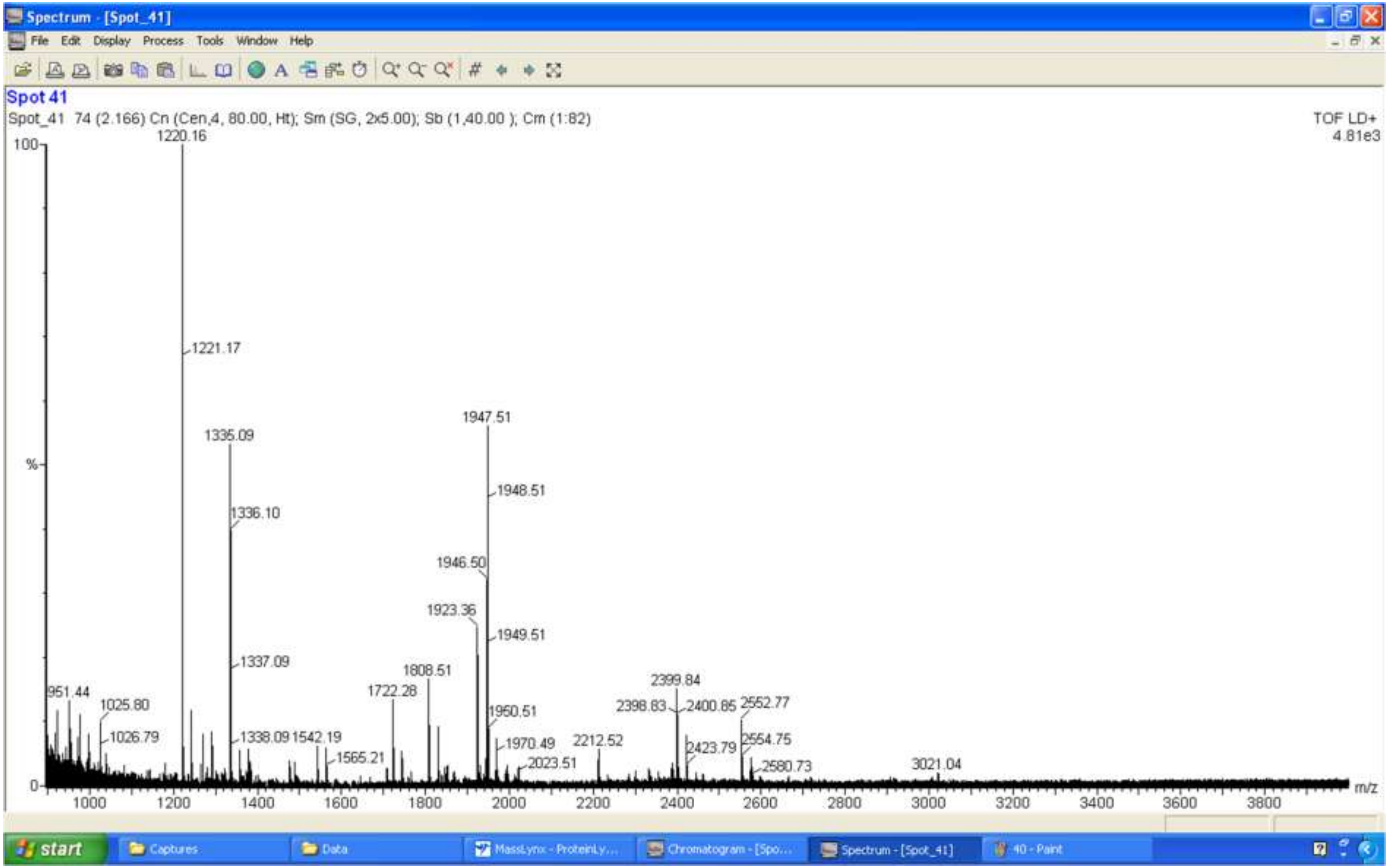

Spectrum - [Spot_41]
File Edit Display Process Tools Window Help
Spot 41
Spot_41 74 (2.166) Cn (Cen,4, 80.00, Ht); Sm (SG, 2x5.00); Sb (1,40.00); Cm (1:82)
TOF LD+
4.81e3
100
%
0
1220.16
1221.17
1335.09
1336.10
1337.09
1338.09
1542.19
1565.21
951.44
1025.80
1026.79
1722.28
1808.51
1923.36
1946.50
1947.51
1948.51
1949.51
1950.51
1970.49
2023.51
2212.52
2398.83
2399.84
2400.85
2423.79
2552.77
2554.75
2580.73
3021.04
1000 1200 1400 1600 1800 2000 2200 2400 2600 2800 3000 3200 3400 3600 3800
m/z
start
Captures
Data
MassLynx - ProteinLy...
Chromatogram - [Spo...
Spectrum - [Spot_41]
40 - Paint

Figure S1.33

Mascot Search Results Spot 42

```
User            : Paul Millares
Email           : paul.millares@gmail.com
Search title    : Spot 42
Database        : Haemonchus 210108 (6387 sequences; 918038 residues)
Timestamp       : 1 Aug 2011 at 10:45:36 GMT
Top Score       : 34 for HCP11007_1, putative nuclear encoded protein  Method: similarity and extension
```

Mascot Score Histogram

Protein score is -10*Log(P), where P is the probability that the observed match is a random event.

Protein scores greater than 51 are significant (p<0.05).

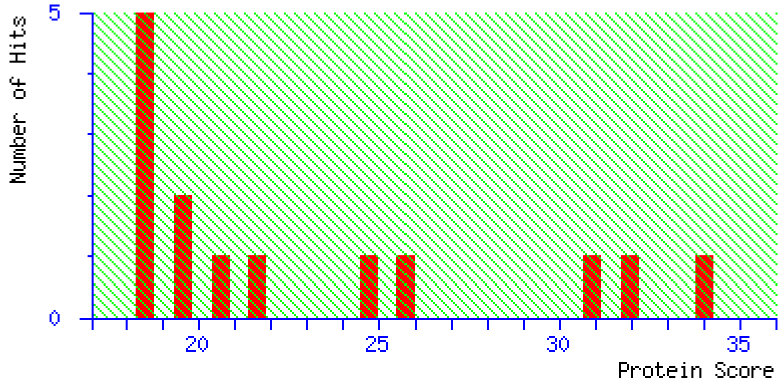

Concise Protein Summary Report

1. HCP11007_1 **Mass:** 23857 **Score:** 34 **Expect:** 2.5 **Matches:** 4
   putative nuclear encoded protein Method: similarity and extension
   HCP00857_1 **Mass:** 13007 **Score:** 26 **Expect:** 16 **Matches:** 3
   putative nuclear encoded protein Method: ESTScan
   HCP10466_1 **Mass:** 5335 **Score:** 24 **Expect:** 27 **Matches:** 2
   putative nuclear encoded protein Method: ESTScan

HCP02005_2 **Mass:** 8953 **Score:** 20 **Expect:** 67 **Matches:** 2
putative nuclear encoded protein Method: ESTScan
HCP00863_1 **Mass:** 9495 **Score:** 20 **Expect:** 68 **Matches:** 2
putative nuclear encoded protein Method: Longest ORF
HCP10624_1 **Mass:** 10178 **Score:** 20 **Expect:** 72 **Matches:** 2
putative nuclear encoded protein Method: ESTScan
HCP03034_2 **Mass:** 10296 **Score:** 19 **Expect:** 84 **Matches:** 2
putative nuclear encoded protein Method: ESTScan

Search Parameters

Type of search : Peptide Mass Fingerprint
Enzyme : Trypsin
Variable modifications : Carbamidomethyl (C),Glu->pyro-Glu (N-term E),Oxidation (M)
Mass values : Monoisotopic
Protein Mass : Unrestricted
Peptide Mass Tolerance : ± 1.2 Da
Peptide Charge State : 1+
Max Missed Cleavages : 1
Number of queries : 7

Protein View

Match to: **HCP11007_1** Score: **34** Expect: **2.5**
putative nuclear encoded protein Method: similarity and extension

Nominal mass (M_r): **23857**; Calculated pI value: **8.12**
NCBI BLAST search of HCP11007_1 against nr
Unformatted sequence string for pasting into other applications

Variable modifications: Carbamidomethyl (C),Glu->pyro-Glu (N-term E),Oxidation (M)
Cleavage by Trypsin: cuts C-term side of KR unless next residue is P
Number of mass values searched: **7**
Number of mass values matched: **4**
Sequence Coverage: **18%**

Matched peptides shown in **Bold Red**

```
  1 VAAFSNGLRS LPHSSSQPKV TLIGASGGIG QPLGLLLKQD NLVKHLALYD
 51 VVGTAGVAAD LSHIDTNAKV TAHTGPKELA AAVANADVIV IPAGVPRKPG
101 MTRDDLFNTN AGIVRDIVDV IAVEAPKAMI AIITNPVNST VPIASEVMKK
151 HGVYDKRRIF GVTTLDVVRA QAFVAELKGL DATKTVIPVV GGHAGTTIIP
201 LLSQVTPKVN FTEDEIMKLT PKIQDAGTE
```

Start - End	Observed	Mr(expt)	Mr(calc)	Delta	Miss	Sequence
104 - 115	1335.04	1334.04	1333.66	0.37	0	R.DDLFNTNAGIVR.D
159 - 169	1220.14	1219.13	1218.70	0.43	0	R.IFGVTTLDVVR.A
170 - 178	976.69	975.68	975.54	0.14	0	R.AQAFVAELK.G
209 - 218	1241.95	1240.94	1240.56	0.38	0	K.VNFTEDEIMK.L Oxidation (M)

No match to: 900.69, 1059.69, 1379.03

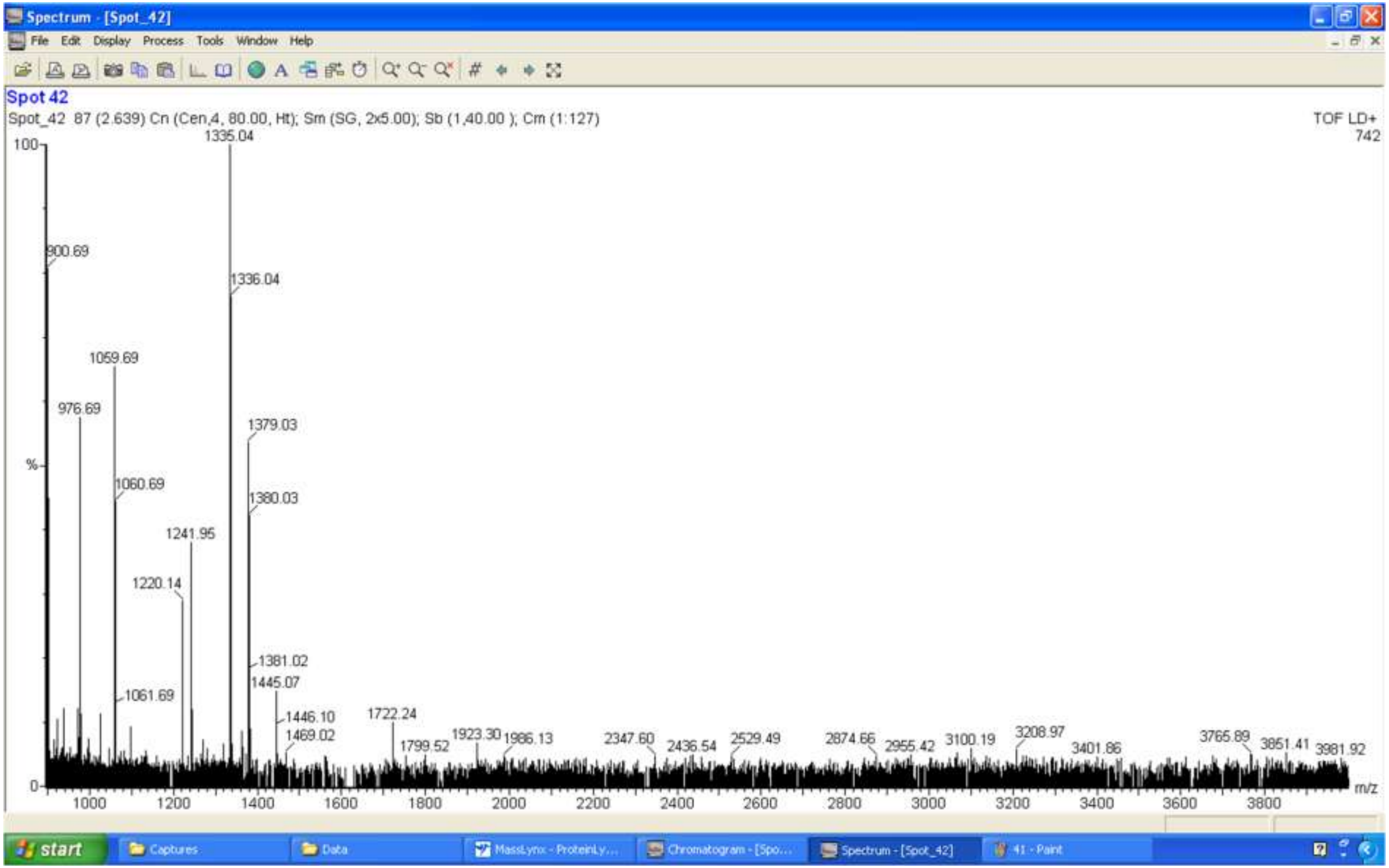
Spectrum - [Spot_42]
File Edit Display Process Tools Window Help
Spot 42
Spot_42 87 (2.639) Cn (Cen,4, 80.00, Ht); Sm (SG, 2x5.00); Sb (1,40.00); Cm (1:127)
TOF LD+
742
100
%
0
1335.04
900.69
1336.04
1059.69
976.69
1379.03
1060.69
1380.03
1241.95
1220.14
1381.02
1445.07
1061.69
1446.10
1722.24
1469.02
1799.52
1923.30
1986.13
2347.60
2436.54
2529.49
2874.66
2955.42
3100.19
3208.97
3401.86
3765.89
3851.41
3981.92
1000 1200 1400 1600 1800 2000 2200 2400 2600 2800 3000 3200 3400 3600 3800
m/z
start
Captures
Data
MassLynx - ProteinLy...
Chromatogram - [Spo...
Spectrum - [Spot_42]
41 - Paint

Figure S1.34

Mascot Search Results Spot 43

```
User            : Paul Millares
Email           : paul.millares@gmail.com
Search title    : Spot 43
Database        : Haemonchus 210108 (6387 sequences; 918038 residues)
Timestamp       : 1 Aug 2011 at 10:46:03 GMT
Top Score       : 68 for HCP00006_1, putative nuclear encoded protein  Method: similarity and extension
```

Mascot Score Histogram

Protein score is -10*Log(P), where P is the probability that the observed match is a random event.

Protein scores greater than 51 are significant (p<0.05).

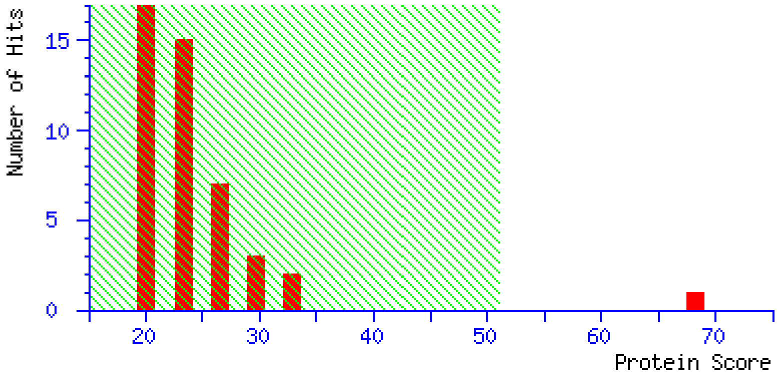

Concise Protein Summary Report

1. HCP00006_1 **Mass:** 59610 **Score:** 68 **Expect:** 0.00097 **Matches:** 35
putative nuclear encoded protein Method: similarity and extension
HCP00006_2 **Mass:** 59610 **Score:** 68 **Expect:** 0.00097 **Matches:** 35
putative nuclear encoded protein Method: similarity and extension
HCP00006_3 **Mass:** 59543 **Score:** 37 **Expect:** 1.4 **Matches:** 27
putative nuclear encoded protein Method: similarity and extension

2. HCP11556_1 **Mass:** 21247 **Score:** 32 **Expect:** 3.9 **Matches:** 14

putative nuclear encoded protein Method: ESTScan

Search Parameters

Type of search : Peptide Mass Fingerprint
Enzyme : Trypsin
Variable modifications : Carbamidomethyl (C),Glu->pyro-Glu (N-term E),Oxidation (M)
Mass values : Monoisotopic
Protein Mass : Unrestricted
Peptide Mass Tolerance : ± 1.2 Da
Peptide Charge State : 1+
Max Missed Cleavages : 1
Number of queries : 83

Protein View

Match to: **HCP00006_1** Score: **68** Expect: **0.00097**
putative nuclear encoded protein Method: similarity and extension

Nominal mass (M_r): **59610**; Calculated pI value: **6.67**
NCBI BLAST search of HCP00006_1 against nr
Unformatted sequence string for pasting into other applications

Variable modifications: Carbamidomethyl (C),Glu->pyro-Glu (N-term E),Oxidation (M)
Cleavage by Trypsin: cuts C-term side of KR unless next residue is P
Number of mass values searched: **83**
Number of mass values matched: **35**
Sequence Coverage: **55%**

Matched peptides shown in **Bold Red**

```
  1 MLSNLARTSG RMAFIRGISS AQMDAHAQVI DDQKPMEEQS NPSFFKMVDY
 51 YFDKGASVIE PKLVEEMKSN VMSTKDKKNL VSGILKAIKP VNKVLYITFP
101 IRRDNGEFEV IEAWRAQHSE HRTPTKGGIR YSMDVCEDEV KALSALMTYK
151 CAAVDVPFGG AKGGVKIDPK QYTDYEIEKI TRRIAIEFAK KGFLGPGVDV
201 PAPDMGTGER EMGWIADTYA QTIGHLDRDA SACITGKPIV AGGIHGRVSA
251 TGRGVWKGLE VFTKEPEYMN KVGLSLGLEG KTIIIQGFGN VGLHTMRYLH
301 RAGAKVIGVQ EWDCAVFNPD GIHPKELEDW RDENGTIKNF PKAKNFEPFA
```

```
351 ELMYEPCDIF VPAACEKAIH KENANRIQAK IIAEAANGPT TPAADKILLE
401 RGNCLIIPDM FINSGGVTVS YFEWLKNLNH VSYGRLSFKY EEDSNRMLLQ
451 SVQDSLEKAL NKEAPVHPND EFTARIAGAS EKDIVHSGLE YTMTRSGEAI
501 IRTARKYNLG LDIRTAAYAN SIEKVYNTYR TAGFTFT
```

Start - End	Observed	Mr(expt)	Mr(calc)	Delta	Miss	Sequence
1 - 11	1204.84	1203.83	1204.63	-0.80	1	-.MLSNLARTSGR.M
2 - 11	1074.37	1073.36	1073.59	-0.23	1	M.LSNLARTSGR.M
8 - 16	1038.57	1037.56	1037.54	0.02	1	R.TSGRMAFIR.G
47 - 54	1081.51	1080.50	1079.46	1.03	0	K.MVDYYFDK.G
63 - 75	1496.85	1495.84	1494.74	1.10	1	K.LVEEMKSNVMSTK.D
69 - 77	1010.32	1009.31	1008.49	0.82	1	K.SNVMSTKDK.K
94 - 102	1121.82	1120.81	1120.66	0.15	0	K.VLYITFPIR.R
94 - 103	1277.93	1276.92	1276.77	0.16	1	K.VLYITFPIRR.D
103 - 115	1619.84	1618.84	1619.77	-0.93	1	R.RDNGEFEVIEAWR.A
127 - 141	1758.05	1757.04	1756.78	0.26	1	K.GGIRYSMDVCEDEVK.A Carbamidomethyl (C)
131 - 150	2313.14	2312.13	2311.04	1.09	1	R.YSMDVCEDEVKALSALMTYK.C Oxidation (M)
151 - 166	1475.90	1474.89	1474.76	0.13	1	K.CAAVDVPFGGAKGGVK.I
171 - 182	1559.92	1558.91	1557.77	1.14	1	K.QYTDYEIEKITR.R
183 - 190	947.34	946.33	946.56	-0.23	1	R.RIAIEFAK.K
184 - 191	919.30	918.29	918.55	-0.26	1	R.IAIEFAKK.G
265 - 271	907.32	906.31	907.37	-1.06	0	K.EPEYMNK.V Glu->pyro-Glu (N-term E); Oxidation (M)
265 - 271	925.30	924.30	925.39	-1.09	0	K.EPEYMNK.V Oxidation (M)
272 - 281	972.56	971.56	971.57	-0.01	0	K.VGLSLGLEGK.T
282 - 297	1773.05	1772.04	1771.94	0.10	0	K.TIIIQGFGNVGLHTMR.Y Oxidation (M)
306 - 325	2281.12	2280.11	2280.10	0.01	0	K.VIGVQEWDCAVFNPDGIHPK.E Carbamidomethyl (C)
326 - 338	1587.85	1586.84	1585.74	1.10	1	K.ELEDWRDENGTIK.N Glu->pyro-Glu (N-term E)
326 - 338	1604.89	1603.88	1603.75	0.13	1	K.ELEDWRDENGTIK.N
345 - 367	2793.16	2792.15	2792.22	-0.07	0	K.NFEPFAELMYEPCDIFVPAACEK.A 2 Carbamidomethyl (C); Oxidation (M)
372 - 380	1043.36	1042.35	1042.55	-0.20	1	K.ENANRIQAK.I
427 - 435	1059.53	1058.53	1058.53	0.00	0	K.NLNHVSYGR.L
436 - 446	1387.82	1386.81	1386.64	0.17	1	R.LSFKYEEDSNR.M
440 - 446	912.37	911.36	911.36	-0.00	0	K.YEEDSNR.M
447 - 458	1406.88	1405.87	1405.71	0.16	0	R.MLLQSVQDSLEK.A Oxidation (M)
463 - 475	1464.84	1463.83	1463.68	0.15	0	K.EAPVHPNDEFTAR.I Glu->pyro-Glu (N-term E)
463 - 475	1482.85	1481.85	1481.69	0.16	0	K.EAPVHPNDEFTAR.I
483 - 495	1521.88	1520.88	1520.73	0.15	0	K.DIVHSGLEYTMTR.S
483 - 495	1537.87	1536.87	1536.72	0.14	0	K.DIVHSGLEYTMTR.S Oxidation (M)
506 - 514	1091.64	1090.64	1090.61	0.02	1	R.KYNLGLDIR.T
507 - 514	963.53	962.53	962.52	0.01	0	K.YNLGLDIR.T
515 - 524	1067.53	1066.52	1066.53	-0.01	0	R.TAAYANSIEK.V

No match to: 897.32, 900.27, 928.32, 935.30, 941.30, 944.34, 951.28, 958.35, 969.56, 970.28, 979.35, 982.35, 985.51, 992.32, 994.54, 1000.48, 1006.47, 1007.31, 1016.53, 1023.33, 1031.30, 1049.33, 1050.33, 1056.30, 1103.44, 1113.72, 1135.79, 1142.54, 1150.85, 1157.80, 1179.87, 1213.87, 1320.77, 1442.89, 1472.86, 1504.84, 1636.88, 1707.82, 1712.03, 1751.93, 1784.90, 1789.01, 1795.02, 1839.04, 2211.11, 2233.02, 2298.15, 2303.13

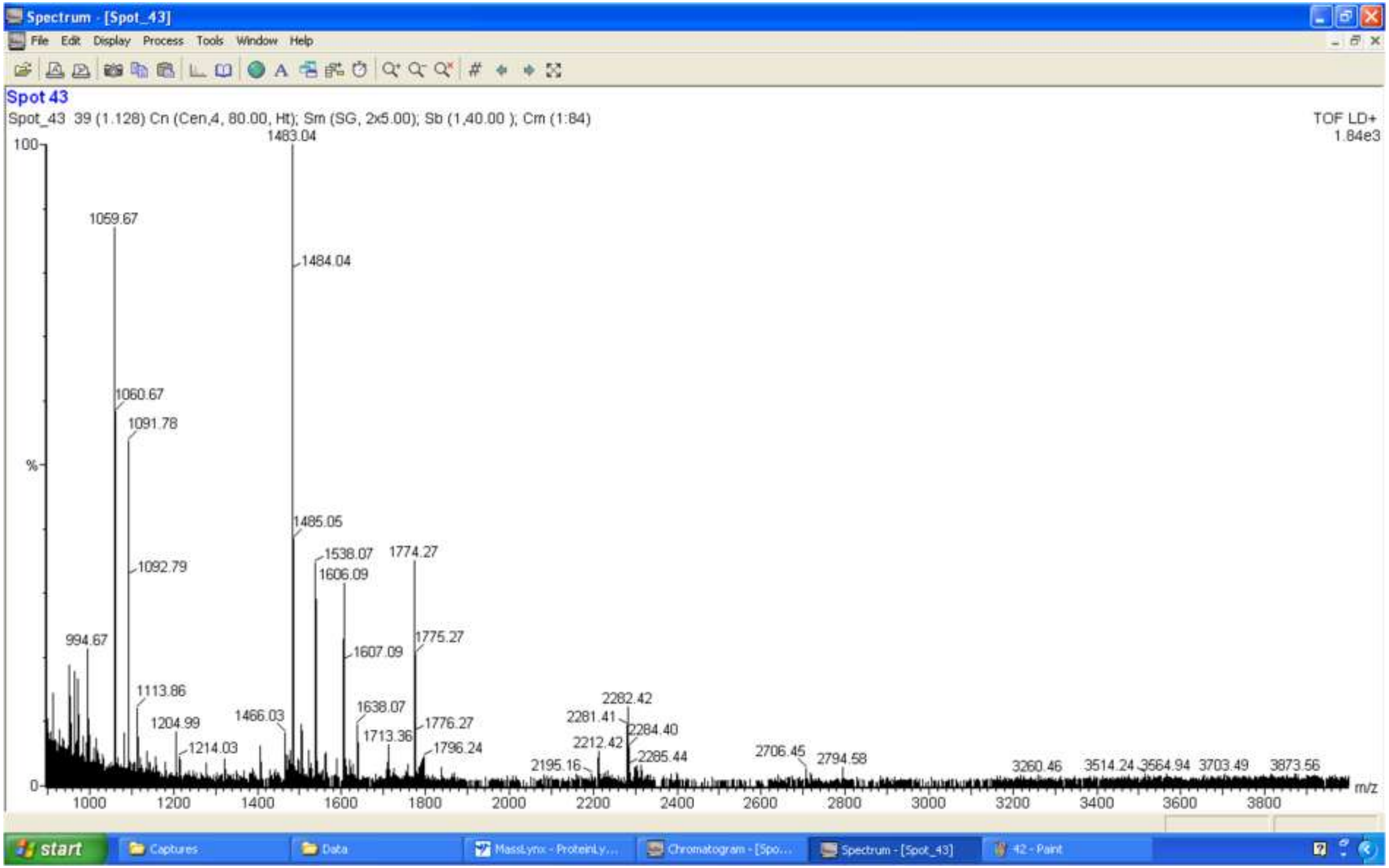
Spectrum - [Spot_43]
File Edit Display Process Tools Window Help
Spot 43
Spot_43 39 (1.128) Cn (Cen,4, 80.00, Ht); Sm (SG, 2x5.00); Sb (1,40.00); Cm (1:84)
TOF LD+
1.84e3
100
%
0
1483.04
1484.04
1059.67
1060.67
1091.78
1485.05
1538.07
1774.27
1606.09
1092.79
994.67
1775.27
1607.09
1113.86
1638.07
2282.42
1204.99
1466.03
1776.27
2281.41
2284.40
1713.36
1214.03
1796.24
2212.42
2285.44
2706.45
2794.58
2195.16
3260.46
3514.24
3564.94
3703.49
3873.56
1000 1200 1400 1600 1800 2000 2200 2400 2600 2800 3000 3200 3400 3600 3800
m/z
start
Captures
Data
MassLynx - ProteinLy...
Chromatogram - [Spo...
Spectrum - [Spot_43]
42 - Paint

Figure S1.35

Mascot Search Results Spot 44

```
User            : Paul Millares
Email           : paul.millares@gmail.com
Search title    : Spot 44
Database        : Haemonchus 210108 (6387 sequences; 918038 residues)
Timestamp       : 1 Aug 2011 at 10:46:25 GMT
Top Score       : 83 for HCP06393_1, putative nuclear encoded protein  Method: similarity and extension
```

Mascot Score Histogram

Protein score is -10*Log(P), where P is the probability that the observed match is a random event.

Protein scores greater than 51 are significant (p<0.05).

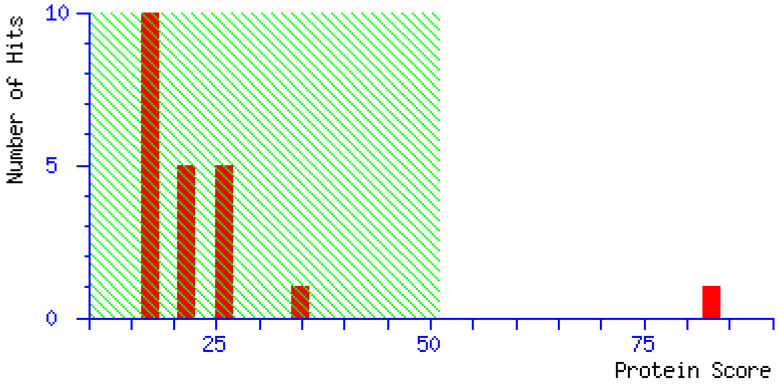

Concise Protein Summary Report

1. HCP06393_1 **Mass:** 27457 **Score:** 83 **Expect:** 3.3e-005 **Matches:** 9
   putative nuclear encoded protein Method: similarity and extension
   HCP06393_2 **Mass:** 27457 **Score:** 83 **Expect:** 3.3e-005 **Matches:** 9
   putative nuclear encoded protein Method: similarity and extension
   HCP03031_1 **Mass:** 18795 **Score:** 25 **Expect:** 23 **Matches:** 5
   putative nuclear encoded protein Method: ESTScan

HCP01606_1 **Mass:** 17651 **Score:** 20 **Expect:** 64 **Matches:** 3
putative nuclear encoded protein Method: ESTScan
HCP00598_1 **Mass:** 18052 **Score:** 20 **Expect:** 70 **Matches:** 3
putative nuclear encoded protein Method: ESTScan
HCP10342_1 **Mass:** 5786 **Score:** 19 **Expect:** 73 **Matches:** 2
putative nuclear encoded protein Method: Longest ORF
HCP03214_1 **Mass:** 5392 **Score:** 19 **Expect:** 82 **Matches:** 2
putative nuclear encoded protein Method: ESTScan
HCP06142_1 **Mass:** 6321 **Score:** 19 **Expect:** 84 **Matches:** 2
putative nuclear encoded protein Method: Longest ORF
HCP08535_2 **Mass:** 5423 **Score:** 19 **Expect:** 86 **Matches:** 2
putative nuclear encoded protein Method: ESTScan
HCP10464_1 **Mass:** 20463 **Score:** 18 **Expect:** 92 **Matches:** 3
putative nuclear encoded protein Method: ESTScan
HCP01373_1 **Mass:** 24104 **Score:** 18 **Expect:** 97 **Matches:** 4
putative nuclear encoded protein Method: similarity and extension
HCP07158_1 **Mass:** 20254 **Score:** 18 **Expect:** 1e+002 **Matches:** 3
putative nuclear encoded protein Method: Longest ORF
HCP05120_1 **Mass:** 6614 **Score:** 18 **Expect:** 1.1e+002 **Matches:** 2
putative nuclear encoded protein Method: ESTScan
HCP07777_1 **Mass:** 8359 **Score:** 17 **Expect:** 1.2e+002 **Matches:** 2
putative nuclear encoded protein Method: ESTScan
HCP02813_1 **Mass:** 20342 **Score:** 17 **Expect:** 1.2e+002 **Matches:** 3
putative nuclear encoded protein Method: ESTScan

---

2. HCP09633_1 **Mass:** 4434 **Score:** 36 **Expect:** 1.5 **Matches:** 4
putative nuclear encoded protein Method: ESTScan
HCP00097_1 **Mass:** 6994 **Score:** 18 **Expect:** 94 **Matches:** 2
putative nuclear encoded protein Method: Longest ORF
HCP01362_1 **Mass:** 7015 **Score:** 18 **Expect:** 1.1e+002 **Matches:** 2

putative nuclear encoded protein Method: ESTScan

HCP01437_1 **Mass:** 7604 **Score:** 17 **Expect:** 1.2e+002 **Matches:** 2

putative nuclear encoded protein Method: ESTScan

Search Parameters

Type of search : Peptide Mass Fingerprint
Enzyme : Trypsin
Variable modifications : Carbamidomethyl (C),Glu->pyro-Glu (N-term E),Oxidation (M)
Mass values : Monoisotopic
Protein Mass : Unrestricted
Peptide Mass Tolerance : ± 1.2 Da
Peptide Charge State : 1+
Max Missed Cleavages : 1
Number of queries : 13

Protein View

Match to: **HCP06393_1** Score: **83** Expect: **3.3e-005**
putative nuclear encoded protein Method: similarity and extension

Nominal mass (M_r): **27457**; Calculated pI value: **8.18**
NCBI BLAST search of HCP06393_1 against nr
Unformatted sequence string for pasting into other applications

Variable modifications: Carbamidomethyl (C),Glu->pyro-Glu (N-term E),Oxidation (M)
Cleavage by Trypsin: cuts C-term side of KR unless next residue is P
Number of mass values searched: **13**
Number of mass values matched: **9**
Sequence Coverage: **36%**

Matched peptides shown in **Bold Red**

```
  1 MTRKFFVGGN WKMNGDKKSI DGICAFLNQS GGVADVEVIV APPALYLTYV
 51 KDQIKNSVKV SAQNCYKVPK GAFTGEVSPA MLKDLGIEWV ILGHSERRHI
101 FGESDQLIAE KVVHCLENHI NVIFCIGEKL EEREAGKTKE VNFRQMQALV
151 DKKVDWTNIV IAYEPVWAIG TGKTATPEQA QEVHLWIREF LKEKVSPDVA
201 EKTRIIYGGS VTAENCRDLG KKPDIDGFLV GGASLKPDFV KIINARK
```

Start - End Observed Mr(expt) Mr(calc) Delta Miss Sequence

5 - 12	954.61	953.61	953.48	0.13	0	K.FFVGGNWK.M
84 - 97	1624.17	1623.16	1622.84	0.32	0	K.DLGIEWVILGHSER.R
98 - 111	1643.18	1642.17	1641.85	0.32	1	R.RHIFGESDQLIAEK.V
99 - 111	1487.09	1486.08	1485.75	0.34	0	R.HIFGESDQLIAEK.V
174 - 188	1779.23	1778.22	1777.91	0.31	0	K.TATPEQAQEVHLWIR.E
203 - 217	1640.16	1639.16	1638.81	0.34	1	K.TRIIYGGSVTAENCR.D
205 - 217	1440.04	1439.03	1438.69	0.35	0	R.IIYGGSVTAENCR.D Carbamidomethyl (C)
205 - 221	1795.22	1794.22	1794.89	-0.68	1	R.IIYGGSVTAENCRDLGK.K
222 - 241	2103.45	2102.44	2102.14	0.30	0	K.KPDIDGFLVGGASLKPDFVK.I

No match to: 1067.59, 1395.08, 1656.15, 1811.22

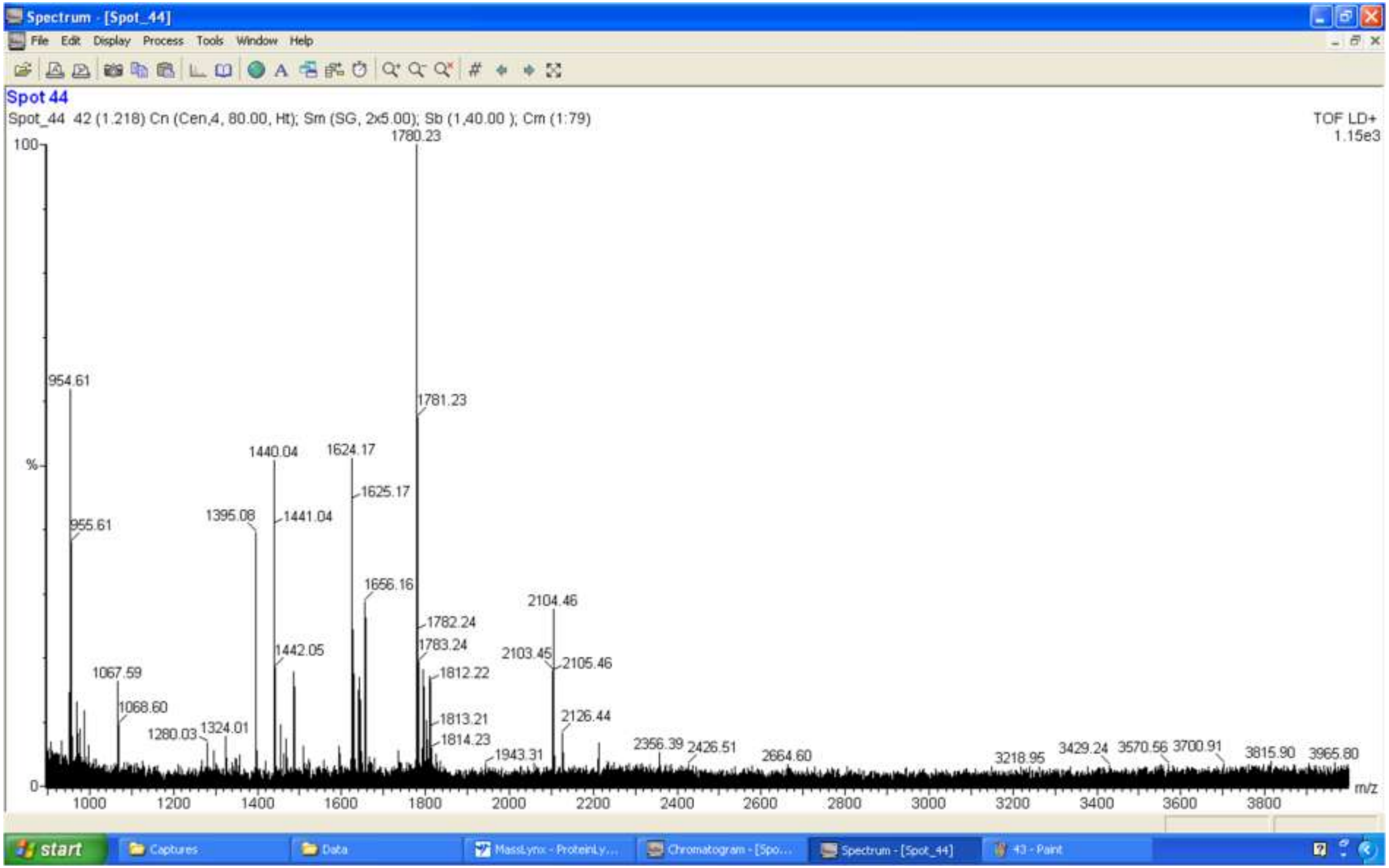
Spectrum - [Spot_44]
File Edit Display Process Tools Window Help
Spot 44
Spot_44 42 (1.218) Cn (Cen,4, 80.00, Ht); Sm (SG, 2x5.00); Sb (1,40.00); Cm (1:79)
TOF LD+
1.15e3
100
%
0
1780.23
954.61
1781.23
1440.04
1624.17
1625.17
955.61
1395.08
1441.04
1656.16
2104.46
1782.24
1783.24
1442.05
2103.45
2105.46
1067.59
1812.22
1068.60
1813.21
2126.44
1280.03
1324.01
1814.23
1943.31
2356.39
2426.51
2664.60
3218.95
3429.24
3570.56
3700.91
3815.90
3965.80
1000 1200 1400 1600 1800 2000 2200 2400 2600 2800 3000 3200 3400 3600 3800
m/z
start
Captures
Data
MassLynx - ProteinLy...
Chromatogram - [Spo...
Spectrum - [Spot_44]
43 - Paint

Figure S1.36

Mascot Search Results Spot 45

```
User            : Paul Millares
Email           : paul.millares@gmail.com
Search title    : Spot 45
Database        : Haemonchus 210108 (6387 sequences; 918038 residues)
Timestamp       : 1 Aug 2011 at 10:46:47 GMT
Top Score       : 96 for HCP06393_1, putative nuclear encoded protein  Method: similarity and extension
```

Mascot Score Histogram

Protein score is -10*Log(P), where P is the probability that the observed match is a random event.

Protein scores greater than 51 are significant (p<0.05).

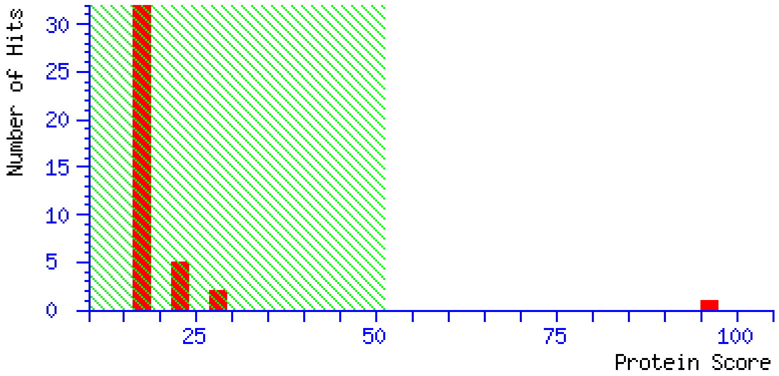

Concise Protein Summary Report

1. HCP06393_1 **Mass:** 27457 **Score:** 96 **Expect:** 1.5e-006 **Matches:** 13
   putative nuclear encoded protein Method: similarity and extension
   HCP06393_2 **Mass:** 27457 **Score:** 96 **Expect:** 1.5e-006 **Matches:** 13
   putative nuclear encoded protein Method: similarity and extension
   HCP01606_1 **Mass:** 17651 **Score:** 22 **Expect:** 40 **Matches:** 4
   putative nuclear encoded protein Method: ESTScan

HCP01373_1 **Mass:** 24104 **Score:** 19 **Expect:** 84 **Matches:** 5
putative nuclear encoded protein Method: similarity and extension
HCP05661_1 **Mass:** 10447 **Score:** 18 **Expect:** 94 **Matches:** 3
putative nuclear encoded protein Method: ESTScan

---

2. HCP01156_1 **Mass:** 17450 **Score:** 29 **Expect:** 7.9 **Matches:** 6
putative nuclear encoded protein Method: similarity and extension
HCP08165_1 **Mass:** 21507 **Score:** 18 **Expect:** 1e+002 **Matches:** 4
putative nuclear encoded protein Method: ESTScan

Search Parameters

Type of search : Peptide Mass Fingerprint
Enzyme : Trypsin
Variable modifications : Carbamidomethyl (C),Glu->pyro-Glu (N-term E),Oxidation (M)
Mass values : Monoisotopic
Protein Mass : Unrestricted
Peptide Mass Tolerance : ± 1.2 Da
Peptide Charge State : 1+
Max Missed Cleavages : 1
Number of queries : 21

Protein View

Match to: **HCP06393_1** Score: **96** Expect: **1.5e-006**
putative nuclear encoded protein Method: similarity and extension

Nominal mass (M_r): **27457**; Calculated pI value: **8.18**
NCBI BLAST search of HCP06393_1 against nr
Unformatted sequence string for pasting into other applications

Variable modifications: Carbamidomethyl (C),Glu->pyro-Glu (N-term E),Oxidation (M)
Cleavage by Trypsin: cuts C-term side of KR unless next residue is P
Number of mass values searched: **21**
Number of mass values matched: **13**
Sequence Coverage: **48%**

Matched peptides shown in **Bold Red**

```
  1 MTRKFFVGGN WKMNGDKKSI DGICAFLNQS GGVADVEVIV APPALYLTYV
 51 KDQIKNSVKV SAQNCYKVPK GAFTGEVSPA MLKDLGIEWV ILGHSERRHI
101 FGESDQLIAE KVVHCLENHI NVIFCIGEKL EEREAGKTKE VNFRQMQALV
151 DKKVDWTNIV IAYEPVWAIG TGKTATPEQA QEVHLWIREF LKEKVSPDVA
201 EKTRIIYGGS VTAENCRDLG KKPDIDGFLV GGASLKPDFV KIINARK
```

Start - End	Observed	Mr(expt)	Mr(calc)	Delta	Miss	Sequence
5 - 12	954.59	953.59	953.48	0.11	0	K.FFVGGNWK.M
60 - 67	969.56	968.56	968.44	0.12	0	K.VSAQNCYK.V Carbamidomethyl (C)
60 - 67	970.58	969.58	968.44	1.14	0	K.VSAQNCYK.V Carbamidomethyl (C)
71 - 83	1323.98	1322.97	1322.65	0.32	0	K.GAFTGEVSPAMLK.D Oxidation (M)
84 - 97	1624.14	1623.14	1622.84	0.30	0	K.DLGIEWVILGHSER.R
98 - 111	1643.14	1642.14	1641.85	0.29	1	R.RHIFGESDQLIAEK.V
99 - 111	1487.05	1486.04	1485.75	0.30	0	R.HIFGESDQLIAEK.V
145 - 152	932.56	931.55	931.48	0.07	0	R.QMQALVDK.K
174 - 188	1779.21	1778.20	1777.91	0.29	0	K.TATPEQAQEVHLWIR.E
203 - 217	1640.14	1639.13	1638.81	0.31	1	K.TRIIYGGSVTAENCR.D
205 - 217	1440.00	1439.00	1438.69	0.31	0	R.IIYGGSVTAENCR.D Carbamidomethyl (C)
205 - 221	1795.20	1794.19	1794.89	-0.70	1	R.IIYGGSVTAENCRDLGK.K
222 - 241	2103.41	2102.40	2102.14	0.26	0	K.KPDIDGFLVGGASLKPDFVK.I

No match to: 977.57, 986.58, 999.55, 1128.90, 1280.02, 1454.02, 1656.13, 1811.19

Figure S1.37

Mascot Search Results Spot 45

```
User            : Paul Millares
Email           : paul.millares@gmail.com
Search title    : Spot 45
MS data file    : Spot 45.txt
Database        : NCBInr 20110729 (14821581 sequences; 5074018658 residues)
Timestamp       : 3 Aug 2011 at 21:33:31 GMT
Top Score       : 96 for gi|313509547, triosephosphate isomerase [Haemonchus contortus]
```

Mascot Score Histogram

Protein score is -10*Log(P), where P is the probability that the observed match is a random event.

Protein scores greater than 84 are significant (p<0.05).

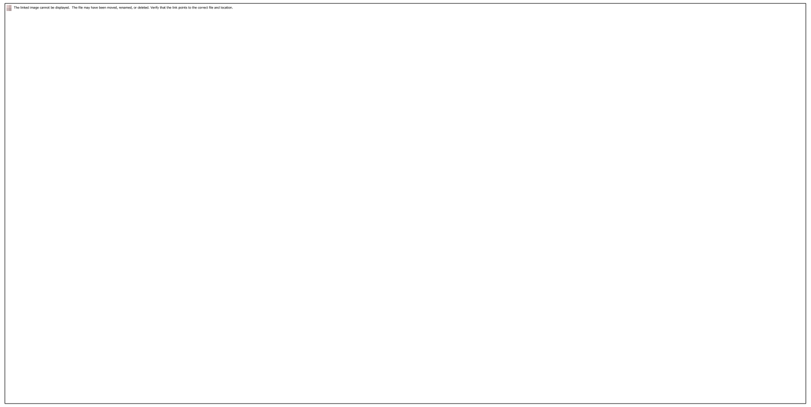

Concise Protein Summary Report

1. gi|313509547 **Mass:** 27457 **Score:** **96** **Expect:** 0.0036 **Matches:** 13
   triosephosphate isomerase [Haemonchus contortus]
   gi|303242830 **Mass:** 7243 **Score:** 49 **Expect:** 2e+02 **Matches:** 6
   hypothetical protein AceceDRAFT_4645 [Acetivibrio cellulolyticus CD2]

2. gi|86749249 **Mass:** 16814 **Score:** 64 **Expect:** 5.9 **Matches:** 8
GCN5-related N-acetyltransferase [Rhodopseudomonas palustris HaA2]

Search Parameters

Type of search : Peptide Mass Fingerprint
Enzyme : Trypsin
Variable modifications : Carbamidomethyl (C),Glu->pyro-Glu (N-term E),Oxidation (M)
Mass values : Monoisotopic
Protein Mass : Unrestricted
Peptide Mass Tolerance : ± 1.2 Da
Peptide Charge State : 1+
Max Missed Cleavages : 1
Number of queries : 21

Protein View

Match to: **gi|313509547** Score: **96** Expect: **0.0036**
triosephosphate isomerase [Haemonchus contortus]
Found in search of Spot 45.txt

Nominal mass (M_r): **27457**; Calculated pI value: **8.18**
NCBI BLAST search of gi|313509547 against nr
Unformatted sequence string for pasting into other applications

Taxonomy: Haemonchus contortus

Variable modifications: Carbamidomethyl (C),Glu->pyro-Glu (N-term E),Oxidation (M)
Cleavage by Trypsin: cuts C-term side of KR unless next residue is P
Number of mass values searched: **21**
Number of mass values matched: **13**
Sequence Coverage: **48%**

Matched peptides shown in **Bold Red**

1 MTRK**FFVGGN WK**MNGDKKSI DGICAFLNQS GGVADVEVIV APPALYLTYV
51 KDQIKNSVK**V SAQNCYK**VPK **GAFTGEVSPA MLKDLGIEWV ILGHSERRHI**
101 **FGESDQLIAE K**VVHCLENHI NVIFCIGEKL EEREAGKTKE VNFR**QMQALV**
151 **DK**KVDWTNIV IAYEPVWAIG TGK**TATPEQA QEVHLWIR**EF LKEKVSPDVA
201 EK**TRIIYGGS VTAENCRDLG KKPDIDGFLV GGASLKPDFV K**IINARK

Start - End	Observed	Mr(expt)	Mr(calc)	Delta	Miss	Sequence	
5 - 12	954.5941	953.5868	953.4760	0.1109	0	K.FFVGGNWK.M	
60 - 67	969.5643	968.5570	968.4386	0.1185	0	K.VSAQNCYK.V	Carbamidomethyl (C)
60 - 67	970.5823	969.5750	968.4386	1.1365	0	K.VSAQNCYK.V	Carbamidomethyl (C)
71 - 83	1323.9795	1322.9722	1322.6540	0.3182	0	K.GAFTGEVSPAMLK.D	Oxidation (M)
84 - 97	1624.1448	1623.1375	1622.8416	0.2959	0	K.DLGIEWVILGHSER.R	
98 - 111	1643.1448	1642.1375	1641.8474	0.2901	1	R.RHIFGESDQLIAEK.V	
99 - 111	1487.0494	1486.0421	1485.7463	0.2958	0	R.HIFGESDQLIAEK.V	
145 - 152	932.5569	931.5496	931.4797	0.0699	0	R.QMQALVDK.K	
174 - 188	1779.2051	1778.1978	1777.9111	0.2867	0	K.TATPEQAQEVHLWIR.E	
203 - 217	1640.1367	1639.1294	1638.8148	0.3146	1	K.TRIIYGGSVTAENCR.D	
205 - 217	1440.0034	1438.9961	1438.6875	0.3087	0	R.IIYGGSVTAENCR.D	Carbamidomethyl (C)
205 - 221	1795.1957	1794.1884	1794.8934	-0.7050	1	R.IIYGGSVTAENCRDLGK.K	
222 - 241	2103.4050	2102.3977	2102.1412	0.2565	0	K.KPDIDGFLVGGASLKPDFVK.I	

No match to: 977.5720, 986.5824, 999.5510, 1128.9027, 1280.0199, 1454.0184, 1656.1294, 1811.1886

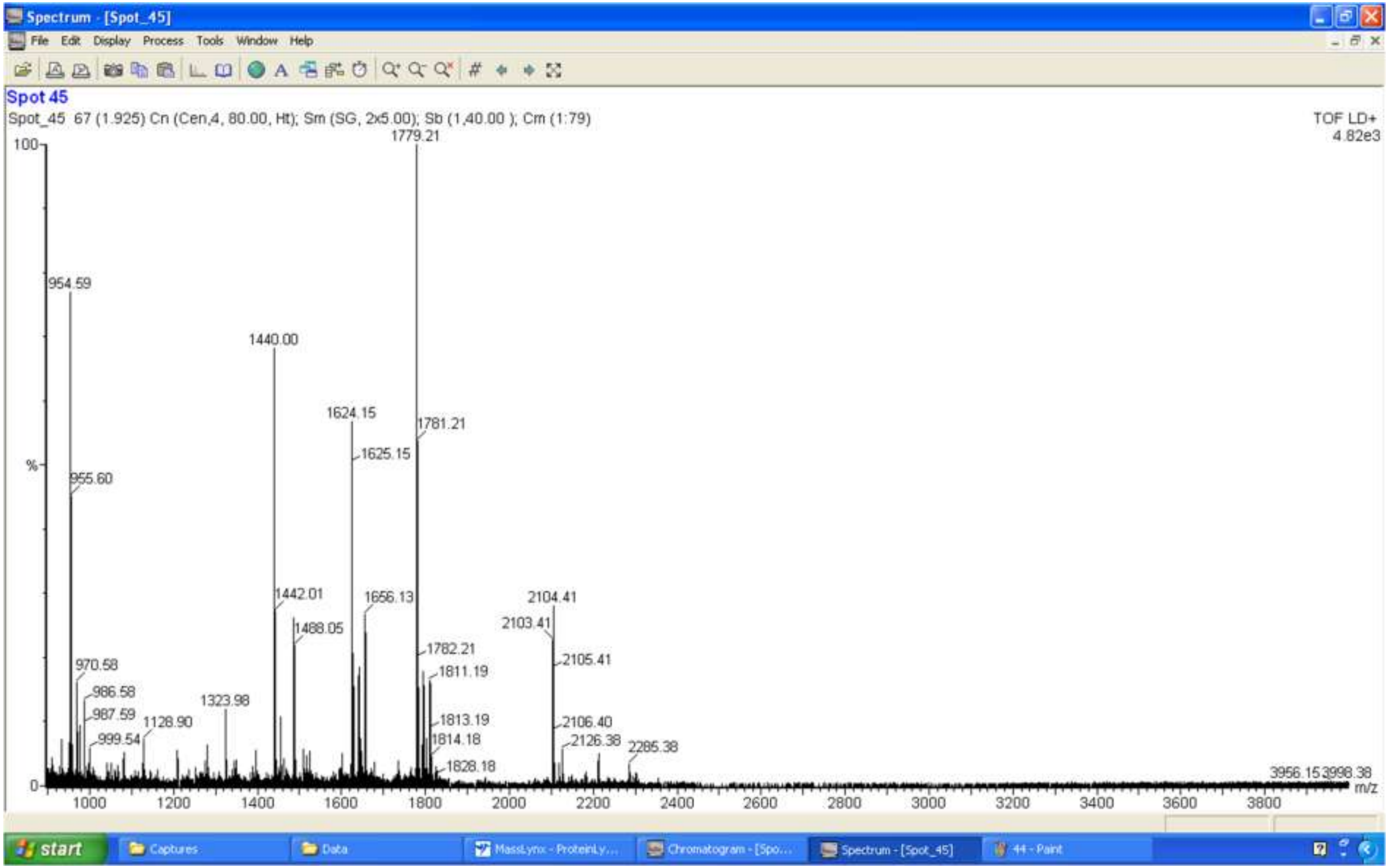

Spectrum - [Spot_45]
File Edit Display Process Tools Window Help
Spot 45
Spot_45 67 (1.925) Cn (Cen,4, 80.00, Ht); Sm (SG, 2x5.00); Sb (1,40.00); Cm (1:79)
TOF LD+
4.82e3
100
%
0
1779.21
954.59
1440.00
1624.15
1781.21
1625.15
955.60
1442.01
1656.13
2104.41
1488.05
2103.41
1782.21
2105.41
970.58
1811.19
986.58
1323.98
987.59
1128.90
1813.19
2106.40
999.54
1814.18
2126.38
2285.38
1828.18
3956.15
3998.38
m/z
1000
1200
1400
1600
1800
2000
2200
2400
2600
2800
3000
3200
3400
3600
3800
start
Captures
Data
MassLynx - ProteinLy...
Chromatogram - [Spo...
Spectrum - [Spot_45]
44 - Paint

Figure S1.38

Mascot Search Results Spot 46

User : Paul Millares
Email : paul.millares@gmail.com
Search title : Spot 46
Database : Haemonchus 210108 (6387 sequences; 918038 residues)
Timestamp : 1 Aug 2011 at 10:47:09 GMT
Top Score : 78 for HCP02208_1, putative nuclear encoded protein Method: similarity and extension

Mascot Score Histogram

Protein score is -10*Log(P), where P is the probability that the observed match is a random event.

Protein scores greater than 51 are significant (p<0.05).

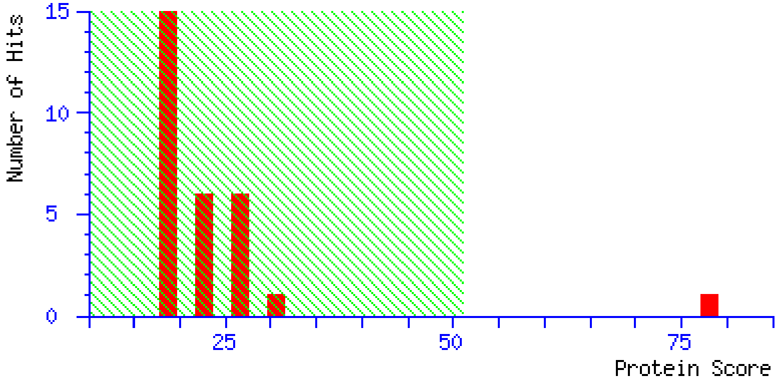

Concise Protein Summary Report

1. HCP02208_1 **Mass:** 19500 **Score:** 78 **Expect:** 0.0001 **Matches:** 8
putative nuclear encoded protein Method: similarity and extension
HCP04093_1 **Mass:** 20465 **Score:** 26 **Expect:** 16 **Matches:** 4
putative nuclear encoded protein Method: similarity and extension
HCP05289_2 **Mass:** 11639 **Score:** 22 **Expect:** 38 **Matches:** 3
putative nuclear encoded protein Method: similarity and extension

HCP01264_1 **Mass:** 14734 **Score:** 21 **Expect:** 52 **Matches:** 3
putative nuclear encoded protein Method: similarity and extension
HCP06527_1 **Mass:** 6804 **Score:** 20 **Expect:** 70 **Matches:** 2
putative nuclear encoded protein Method: ESTScan
HCP06226_1 **Mass:** 15634 **Score:** 20 **Expect:** 70 **Matches:** 3
putative nuclear encoded protein Method: ESTScan
HCP05289_1 **Mass:** 18962 **Score:** 19 **Expect:** 75 **Matches:** 3
putative nuclear encoded protein Method: similarity and extension
HCP04348_1 **Mass:** 20384 **Score:** 19 **Expect:** 79 **Matches:** 3
putative nuclear encoded protein Method: ESTScan
HCP06549_1 **Mass:** 4876 **Score:** 19 **Expect:** 80 **Matches:** 2
putative nuclear encoded protein Method: Longest ORF

---

2. HCP06226_2 **Mass:** 14375 **Score:** 29 **Expect:** 8.4 **Matches:** 4
putative nuclear encoded protein Method: ESTScan

Search Parameters

Type of search : Peptide Mass Fingerprint
Enzyme : Trypsin
Variable modifications : Carbamidomethyl (C),Glu->pyro-Glu (N-term E),Oxidation (M)
Mass values : Monoisotopic
Protein Mass : Unrestricted
Peptide Mass Tolerance : ± 1.2 Da
Peptide Charge State : 1+
Max Missed Cleavages : 1
Number of queries : 14

Protein View

Match to: **HCP02208_1** Score: **78** Expect: **0.0001**
putative nuclear encoded protein Method: similarity and extension

Nominal mass (M_r): **19500**; Calculated pI value: **5.63**
NCBI BLAST search of HCP02208_1 against nr
Unformatted sequence string for pasting into other applications

Variable modifications: Carbamidomethyl (C),Glu->pyro-Glu (N-term E),Oxidation (M)
Cleavage by Trypsin: cuts C-term side of KR unless next residue is P
Number of mass values searched: **14**
Number of mass values matched: **8**
Sequence Coverage: **60%**

Matched peptides shown in **Bold Red**

1 MVNYKLTYFD GRGVAETAR**Q IFALADQK**FE DNRLTR**EAFA EVKPTLPFGQ**
51 **VPVLEVDGKQ LAQSLAINR**Y LAKTFGFAGK **DDFEAALIDS LGDQYSDYR**A
101 EMK**TYYYAAH GFMPGDPEKL KTEVLFPAR**D KFLNFITKFL KNNASHGYLI
151 GDK**ISWVDVL IAEHMADMSR** TV

Start - End	Observed	Mr(expt)	Mr(calc)	Delta	Miss	Sequence
20 - 28	1033.67	1032.67	1032.56	0.11	0	R.QIFALADQK.F
37 - 59	2470.54	2469.53	2469.32	0.21	0	R.EAFAEVKPTLPFGQVPVLEVDGK.Q
60 - 69	1113.86	1112.86	1112.63	0.23	0	K.QLAQSLAINR.Y
81 - 99	2193.18	2192.17	2191.95	0.22	0	K.DDFEAALIDSLGDQYSDYR.A
104 - 119	1863.06	1862.05	1861.80	0.25	0	K.TYYYAAHGFMPGDPEK.L Oxidation (M)
120 - 129	1174.07	1173.06	1172.69	0.37	1	K.LKTEVLFPAR.D
122 - 129	932.62	931.62	931.51	0.10	0	K.TEVLFPAR.D
154 - 170	2005.18	2004.17	2003.94	0.23	0	K.ISWVDVLIAEHMADMSR.T 2 Oxidation (M)

No match to: 899.63, 954.60, 1016.65, 1096.75, 1477.06, 1499.02

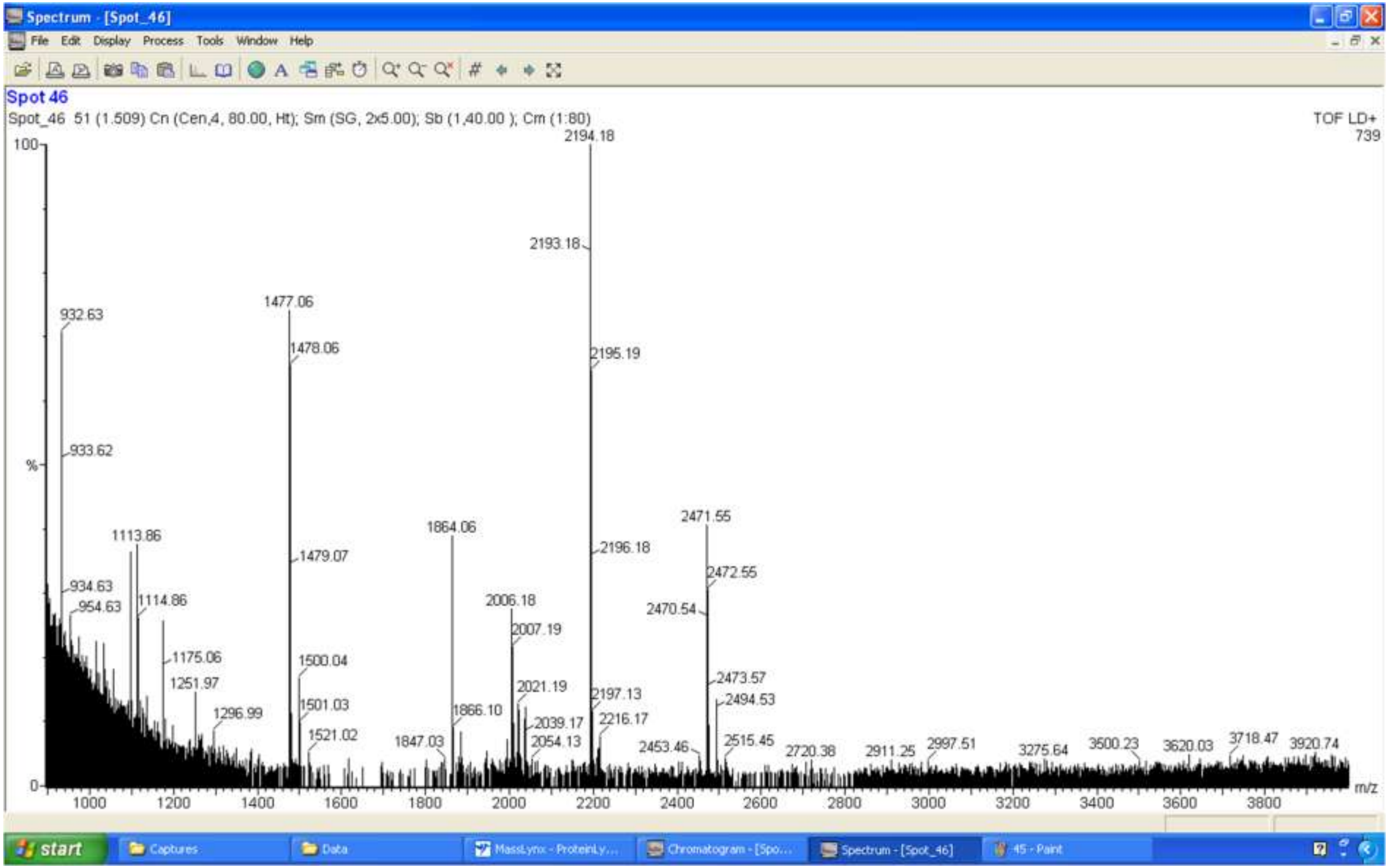
Spectrum - [Spot_46]
File Edit Display Process Tools Window Help
Spot 46
Spot_46 51 (1.509) Cn (Cen,4, 80.00, Ht); Sm (SG, 2x5.00); Sb (1,40.00); Cm (1:80)
TOF LD+
739
100
%
0
2194.18
2193.18
932.63
1477.06
1478.06
2195.19
933.62
1864.06
2471.55
1113.86
1479.07
2196.18
934.63
2472.55
954.63
1114.86
2006.18
2470.54
2007.19
1175.06
1500.04
1251.97
2021.19
2473.57
2197.13
1296.99
1501.03
1866.10
2494.53
2039.17
2216.17
1521.02
1847.03
2054.13
2453.46
2515.45
2720.38
2911.25
2997.51
3275.64
3500.23
3620.03
3718.47
3920.74
m/z
1000
1200
1400
1600
1800
2000
2200
2400
2600
2800
3000
3200
3400
3600
3800
start
Captures
Data
MassLynx - ProteinLy...
Chromatogram - [Spo...
Spectrum - [Spot_46]
45 - Paint

Figure S1.39

Mascot Search Results Spot 47

User : Paul Millares
Email : paul.millares@gmail.com
Search title : Spot 47
Database : Haemonchus 210108 (6387 sequences; 918038 residues)
Timestamp : 1 Aug 2011 at 10:47:37 GMT
Top Score : 58 for HCP00515_1, putative nuclear encoded protein Method: similarity and extension

Mascot Score Histogram

Protein score is -10*Log(P), where P is the probability that the observed match is a random event.

Protein scores greater than 51 are significant (p<0.05).

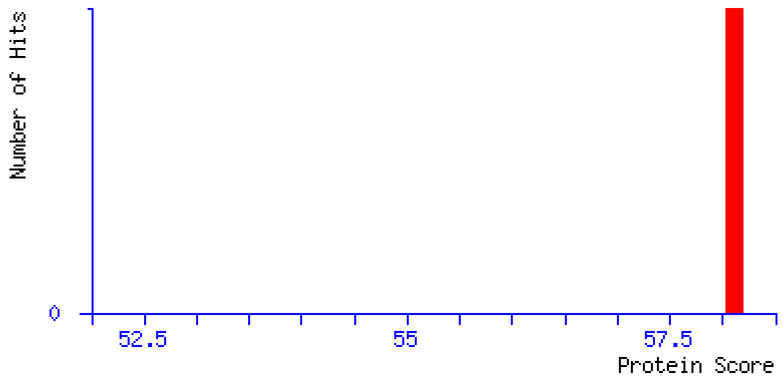

Concise Protein Summary Report

1. HCP00515_1 **Mass:** 22998 **Score:** 58 **Expect:** 0.0099 **Matches:** 5
putative nuclear encoded protein Method: similarity and extension
HCP03173_1 **Mass:** 20643 **Score:** 30 **Expect:** 6.5 **Matches:** 3
putative nuclear encoded protein Method: similarity and extension
HCP03208_1 **Mass:** 20709 **Score:** 26 **Expect:** 17 **Matches:** 3
putative nuclear encoded protein Method: ESTScan

HCP01524_1 **Mass:** 20779 **Score:** 26 **Expect:** 17 **Matches:** 3
putative nuclear encoded protein Method: ESTScan
HCP13312_1 **Mass:** 4311 **Score:** 24 **Expect:** 26 **Matches:** 2
putative nuclear encoded protein Method: ESTScan
HCP04778_1 **Mass:** 7820 **Score:** 24 **Expect:** 29 **Matches:** 2
putative nuclear encoded protein Method: ESTScan
HCP02612_1 **Mass:** 8535 **Score:** 22 **Expect:** 42 **Matches:** 2
putative nuclear encoded protein Method: ESTScan
HCP13050_1 **Mass:** 7127 **Score:** 22 **Expect:** 42 **Matches:** 2
putative nuclear encoded protein Method: ESTScan
HCP01117_1 **Mass:** 10450 **Score:** 22 **Expect:** 43 **Matches:** 2
putative nuclear encoded protein Method: Longest ORF
HCP11290_1 **Mass:** 8091 **Score:** 21 **Expect:** 53 **Matches:** 2
putative nuclear encoded protein Method: ESTScan
HCP00039_2 **Mass:** 47081 **Score:** 20 **Expect:** 62 **Matches:** 3
putative nuclear encoded protein Method: similarity and extension
HCP00039_3 **Mass:** 46979 **Score:** 20 **Expect:** 62 **Matches:** 3
putative nuclear encoded protein Method: similarity and extension
HCP02170_1 **Mass:** 9372 **Score:** 20 **Expect:** 70 **Matches:** 2
putative nuclear encoded protein Method: Longest ORF
HCP10884_1 **Mass:** 14988 **Score:** 20 **Expect:** 70 **Matches:** 2
putative nuclear encoded protein Method: similarity and extension
HCP03495_1 **Mass:** 15075 **Score:** 20 **Expect:** 72 **Matches:** 2
putative nuclear encoded protein Method: similarity and extension
HCP02768_1 **Mass:** 14070 **Score:** 19 **Expect:** 73 **Matches:** 2
putative nuclear encoded protein Method: similarity and extension
HCP06722_1 **Mass:** 13905 **Score:** 19 **Expect:** 73 **Matches:** 2
putative nuclear encoded protein Method: similarity and extension
HCP11061_1 **Mass:** 13595 **Score:** 19 **Expect:** 73 **Matches:** 2
putative nuclear encoded protein Method: ESTScan

HCP01665_1 **Mass:** 15028 **Score:** 19 **Expect:** 73 **Matches:** 2
putative nuclear encoded protein Method: similarity and extension
HCP08791_1 **Mass:** 15284 **Score:** 19 **Expect:** 75 **Matches:** 2
putative nuclear encoded protein Method: similarity and extension
HCP02385_2 **Mass:** 14310 **Score:** 19 **Expect:** 75 **Matches:** 2
putative nuclear encoded protein Method: Longest ORF
HCP01608_1 **Mass:** 15964 **Score:** 19 **Expect:** 80 **Matches:** 2
putative nuclear encoded protein Method: ESTScan
HCP03413_1 **Mass:** 16318 **Score:** 19 **Expect:** 80 **Matches:** 2
putative nuclear encoded protein Method: similarity and extension
HCP10391_1 **Mass:** 14409 **Score:** 19 **Expect:** 80 **Matches:** 2
putative nuclear encoded protein Method: similarity and extension
HCP09642_1 **Mass:** 7623 **Score:** 19 **Expect:** 82 **Matches:** 2
putative nuclear encoded protein Method: similarity and extension
HCP06145_1 **Mass:** 16233 **Score:** 19 **Expect:** 88 **Matches:** 2
putative nuclear encoded protein Method: similarity and extension
HCP01123_1 **Mass:** 57815 **Score:** 18 **Expect:** 92 **Matches:** 3
putative nuclear encoded protein Method: similarity and extension
HCP13129_1 **Mass:** 16277 **Score:** 18 **Expect:** 92 **Matches:** 2
putative nuclear encoded protein Method: similarity and extension
HCP00768_2 **Mass:** 16354 **Score:** 18 **Expect:** 97 **Matches:** 2
putative nuclear encoded protein Method: similarity and extension
HCP00768_1 **Mass:** 16354 **Score:** 18 **Expect:** 97 **Matches:** 2
putative nuclear encoded protein Method: similarity and extension
HCP05193_1 **Mass:** 17315 **Score:** 18 **Expect:** 97 **Matches:** 2
putative nuclear encoded protein Method: similarity and extension
HCP01417_1 **Mass:** 17965 **Score:** 18 **Expect:** 1e+002 **Matches:** 2
putative nuclear encoded protein Method: similarity and extension
HCP08366_1 **Mass:** 19017 **Score:** 18 **Expect:** 1.1e+002 **Matches:** 2
putative nuclear encoded protein Method: similarity and extension

HCP04876_1 **Mass:** 18086 **Score:** 18 **Expect:** 1.1e+002 **Matches:** 2
putative nuclear encoded protein Method: similarity and extension
HCP10662_1 **Mass:** 11096 **Score:** 18 **Expect:** 1.1e+002 **Matches:** 2
putative nuclear encoded protein Method: ESTScan
HCP12381_1 **Mass:** 20021 **Score:** 17 **Expect:** 1.2e+002 **Matches:** 2
putative nuclear encoded protein Method: similarity and extension
HCP04830_1 **Mass:** 18351 **Score:** 17 **Expect:** 1.2e+002 **Matches:** 2
putative nuclear encoded protein Method: ESTScan
HCP01218_1 **Mass:** 12475 **Score:** 17 **Expect:** 1.2e+002 **Matches:** 2
putative nuclear encoded protein Method: ESTScan
HCP07132_1 **Mass:** 21056 **Score:** 17 **Expect:** 1.2e+002 **Matches:** 2
putative nuclear encoded protein Method: similarity and extension
HCP01504_1 **Mass:** 18519 **Score:** 17 **Expect:** 1.2e+002 **Matches:** 2
putative nuclear encoded protein Method: ESTScan
HCP00537_1 **Mass:** 18426 **Score:** 17 **Expect:** 1.2e+002 **Matches:** 2
putative nuclear encoded protein Method: similarity and extension
HCP00465_2 **Mass:** 19784 **Score:** 17 **Expect:** 1.3e+002 **Matches:** 2
putative nuclear encoded protein Method: ESTScan
HCP11423_1 **Mass:** 19328 **Score:** 17 **Expect:** 1.3e+002 **Matches:** 2
putative nuclear encoded protein Method: similarity and extension
HCP11542_1 **Mass:** 19282 **Score:** 17 **Expect:** 1.3e+002 **Matches:** 2
putative nuclear encoded protein Method: similarity and extension
HCP10268_1 **Mass:** 20019 **Score:** 17 **Expect:** 1.3e+002 **Matches:** 2
putative nuclear encoded protein Method: ESTScan
HCP01027_1 **Mass:** 21286 **Score:** 17 **Expect:** 1.3e+002 **Matches:** 2
putative nuclear encoded protein Method: similarity and extension
HCP12564_1 **Mass:** 21028 **Score:** 17 **Expect:** 1.3e+002 **Matches:** 2
putative nuclear encoded protein Method: ESTScan
HCP01533_1 **Mass:** 22169 **Score:** 17 **Expect:** 1.3e+002 **Matches:** 2
putative nuclear encoded protein Method: similarity and extension

HCP01533_2 **Mass:** 22167 **Score:** 17 **Expect:** 1.3e+002 **Matches:** 2
putative nuclear encoded protein Method: similarity and extension
HCP00465_1 **Mass:** 20964 **Score:** 17 **Expect:** 1.4e+002 **Matches:** 2
putative nuclear encoded protein Method: ESTScan

Search Parameters

Type of search : Peptide Mass Fingerprint
Enzyme : Trypsin
Variable modifications : Carbamidomethyl (C),Glu->pyro-Glu (N-term E),Oxidation (M)
Mass values : Monoisotopic
Protein Mass : Unrestricted
Peptide Mass Tolerance : ± 1.2 Da
Peptide Charge State : 1+
Max Missed Cleavages : 1
Number of queries : 5

Protein View

Match to: **HCP00515_1** Score: **58** Expect: **0.0099**
putative nuclear encoded protein Method: similarity and extension

Nominal mass (M_r): **22998**; Calculated pI value: **6.69**
NCBI BLAST search of HCP00515_1 against nr
Unformatted sequence string for pasting into other applications

Variable modifications: Carbamidomethyl (C),Glu->pyro-Glu (N-term E),Oxidation (M)
Cleavage by Trypsin: cuts C-term side of KR unless next residue is P
Number of mass values searched: **5**
Number of mass values matched: **5**
Sequence Coverage: **25%**

Matched peptides shown in **Bold Red**

1 TRGTLVLVAI SVAAVSAAGL FAHHPPPECG LPPFVNDLPA DDQAKLKDIW
51 KNWKEGDK**CY HEQGLTRDLV ETLPTEIR**RK ISK**DALLPPP VR**K**APEEVQE**
101 **QFRK**IINDKT IPVEEKHKKM NELAQKVLTG DNLK**EYNEFT AHIEDR**HKAV
151 ADKAATLSPE AKAAYDKIAK LEKEKHDIIA SLNEQAQEEL FQVFKLRHSK
201 SAKD

Start - End	Observed	Mr(expt)	Mr(calc)	Delta	Miss	Sequence
59 - 67	1163.90	1162.89	1162.52	0.37	0	K.CYHEQGLTR.D Carbamidomethyl (C)
68 - 78	1286.04	1285.03	1284.69	0.34	0	R.DLVETLPTEIR.R
84 - 92	977.69	976.68	976.57	0.11	0	K.DALLPPPVR.K
94 - 104	1361.01	1360.00	1359.68	0.32	1	K.APEEVQEQFRK.I
135 - 146	1523.99	1522.98	1522.67	0.31	0	K.EYNEFTAHIEDR.H

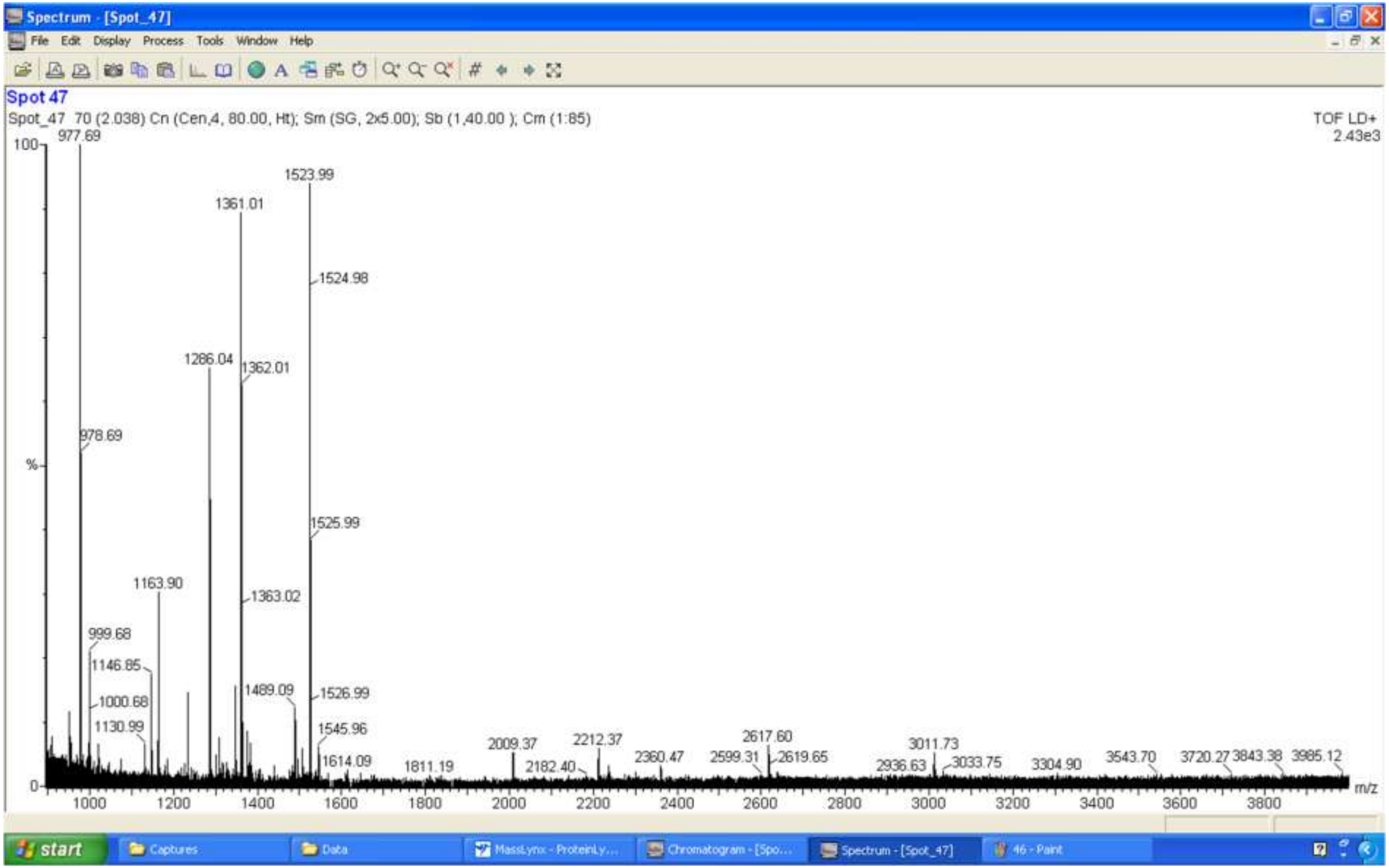
Spectrum - [Spot_47]
File Edit Display Process Tools Window Help
Spot 47
Spot_47 70 (2.038) Cn (Cen,4, 80.00, Ht); Sm (SG, 2x5.00); Sb (1,40.00); Cm (1:85)
TOF LD+
2.43e3
100
%
0
977.69
978.69
999.68
1000.68
1130.99
1146.85
1163.90
1286.04
1361.01
1362.01
1363.02
1489.09
1523.99
1524.98
1525.99
1526.99
1545.96
1614.09
1811.19
2009.37
2182.40
2212.37
2360.47
2599.31
2617.60
2619.65
2936.63
3011.73
3033.75
3304.90
3543.70
3720.27
3843.38
3985.12
1000
1200
1400
1600
1800
2000
2200
2400
2600
2800
3000
3200
3400
3600
3800
m/z
start
Captures
Data
MassLynx - ProteinLy...
Chromatogram - [Spo...
Spectrum - [Spot_47]
46 - Paint

Figure S1.40

Mascot Search Results Spot 53

```
User           : Paul Millares
Email          : paul.millares@gmail.com
Search title   : Spot 53
Database       : Haemonchus 210108 (6387 sequences; 918038 residues)
Timestamp      : 1 Aug 2011 at 10:48:07 GMT
Top Score      : 52 for HCP01409_1, putative nuclear encoded protein  Method: similarity and extension
```

Mascot Score Histogram

Protein score is -10*Log(P), where P is the probability that the observed match is a random event.

Protein scores greater than 51 are significant (p<0.05).

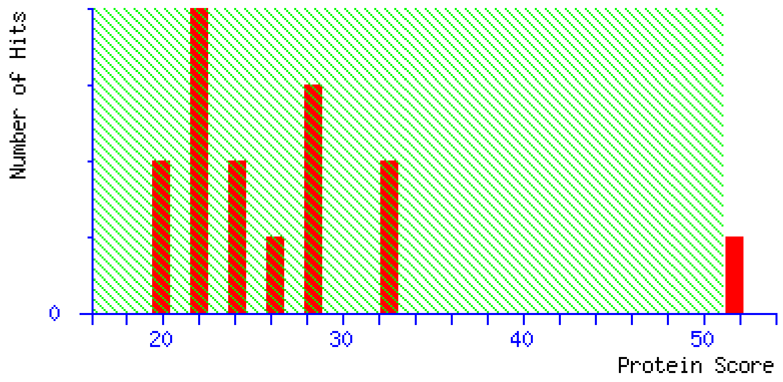

Concise Protein Summary Report

1. HCP01409_1 **Mass:** 21934 **Score:** 52 **Expect:** 0.043 **Matches:** 6
putative nuclear encoded protein Method: similarity and extension
HCP01409_2 **Mass:** 22392 **Score:** 51 **Expect:** 0.051 **Matches:** 6
putative nuclear encoded protein Method: similarity and extension
HCP03369_1 **Mass:** 20767 **Score:** 28 **Expect:** 11 **Matches:** 4
putative nuclear encoded protein Method: ESTScan

HCP00409_1 **Mass:** 21016 **Score:** 27 **Expect:** 14 **Matches:** 4
putative nuclear encoded protein Method: similarity and extension
HCP00409_2 **Mass:** 21016 **Score:** 27 **Expect:** 14 **Matches:** 4
putative nuclear encoded protein Method: similarity and extension
HCP01303_2 **Mass:** 28007 **Score:** 26 **Expect:** 17 **Matches:** 4
putative nuclear encoded protein Method: similarity and extension
HCP01974_2 **Mass:** 11899 **Score:** 24 **Expect:** 27 **Matches:** 3
putative nuclear encoded protein Method: ESTScan
HCP07935_1 **Mass:** 6052 **Score:** 23 **Expect:** 31 **Matches:** 2
putative nuclear encoded protein Method: ESTScan
HCP02271_1 **Mass:** 22097 **Score:** 22 **Expect:** 43 **Matches:** 4
putative nuclear encoded protein Method: similarity and extension
HCP06327_2 **Mass:** 21952 **Score:** 21 **Expect:** 46 **Matches:** 4
putative nuclear encoded protein Method: similarity and extension
HCP10885_1 **Mass:** 16764 **Score:** 21 **Expect:** 50 **Matches:** 3
putative nuclear encoded protein Method: ESTScan
HCP00366_2 **Mass:** 15656 **Score:** 21 **Expect:** 51 **Matches:** 3
putative nuclear encoded protein Method: similarity and extension
HCP13263_1 **Mass:** 6094 **Score:** 21 **Expect:** 54 **Matches:** 2
putative nuclear encoded protein Method: ESTScan
HCP06270_1 **Mass:** 11250 **Score:** 21 **Expect:** 56 **Matches:** 3
putative nuclear encoded protein Method: Longest ORF
HCP02271_2 **Mass:** 24413 **Score:** 21 **Expect:** 57 **Matches:** 4
putative nuclear encoded protein Method: similarity and extension
HCP10350_1 **Mass:** 6655 **Score:** 20 **Expect:** 70 **Matches:** 2
putative nuclear encoded protein Method: Longest ORF

---

2. HCP00006_1 **Mass:** 59610 **Score:** 34 **Expect:** 2.9 **Matches:** 6
putative nuclear encoded protein Method: similarity and extension
HCP00006_2 **Mass:** 59610 **Score:** 34 **Expect:** 2.9 **Matches:** 6

putative nuclear encoded protein Method: similarity and extension

HCP00304_1 **Mass:** 9136 **Score:** 30 **Expect:** 6.2 **Matches:** 3

putative nuclear encoded protein Method: similarity and extension

HCP04735_2 **Mass:** 15549 **Score:** 30 **Expect:** 7 **Matches:** 4

putative nuclear encoded protein Method: similarity and extension

HCP06454_2 **Mass:** 8348 **Score:** 29 **Expect:** 8.2 **Matches:** 3

putative nuclear encoded protein Method: ESTScan

HCP06454_1 **Mass:** 8220 **Score:** 29 **Expect:** 8.4 **Matches:** 3

putative nuclear encoded protein Method: ESTScan

HCP12588_1 **Mass:** 13370 **Score:** 25 **Expect:** 19 **Matches:** 3

putative nuclear encoded protein Method: Longest ORF

HCP09394_1 **Mass:** 5341 **Score:** 22 **Expect:** 42 **Matches:** 3

putative nuclear encoded protein Method: similarity and extension

HCP06413_2 **Mass:** 21950 **Score:** 21 **Expect:** 53 **Matches:** 3

putative nuclear encoded protein Method: similarity and extension

HCP10550_1 **Mass:** 6166 **Score:** 20 **Expect:** 67 **Matches:** 2

putative nuclear encoded protein Method: Longest ORF

HCP02722_1 **Mass:** 24368 **Score:** 20 **Expect:** 70 **Matches:** 3

putative nuclear encoded protein Method: similarity and extension

HCP02811_2 **Mass:** 7716 **Score:** 19 **Expect:** 73 **Matches:** 2

putative nuclear encoded protein Method: similarity and extension

---

Search Parameters

Type of search : Peptide Mass Fingerprint
Enzyme : Trypsin
Variable modifications : Carbamidomethyl (C),Glu->pyro-Glu (N-term E),Oxidation (M)
Mass values : Monoisotopic
Protein Mass : Unrestricted
Peptide Mass Tolerance : ± 1.2 Da
Peptide Charge State : 1+
Max Missed Cleavages : 1
Number of queries : 9

Protein View

Match to: **HCP01409_1** Score: **52** Expect: **0.043**
putative nuclear encoded protein Method: similarity and extension

Nominal mass (M_r): **21934**; Calculated pI value: **8.76**
NCBI BLAST search of HCP01409_1 against nr
Unformatted sequence string for pasting into other applications

Variable modifications: Carbamidomethyl (C),Glu->pyro-Glu (N-term E),Oxidation (M)
Cleavage by Trypsin: cuts C-term side of KR unless next residue is P
Number of mass values searched: **9**
Number of mass values matched: **6**
Sequence Coverage: **23%**

Matched peptides shown in **Bold Red**

```
  1 MSSGAKQYMP VNRLAMGIIN NNHILSKISK SDPDVPQSQD PVMRTIDPRS
 51 YDDLLMVADS IRSRVGEKAN VEVGIICGSG LGPIGDQVED AFVLPYEKIP
101 GFPSVKVVGH KGNLIFGRIG GKKVVCMQGR FHPYEHEMDL ALCAFPVRVM
151 HQLGVKRLVV SNAAGGVNPN FKYGDLMLIK DHIFMPGLAG FSPLVGLSDG
201 RF
```

Start - End	Observed	Mr(expt)	Mr(calc)	Delta	Miss	Sequence
7 - 13	907.37	906.36	906.44	-0.08	0	K.QYMPVNR.L
14 - 27	1538.01	1537.00	1536.84	0.15	0	R.LAMGIINNNHILSK.I
14 - 30	1881.27	1880.27	1881.05	-0.78	1	R.LAMGIINNNHILSKISK.S Oxidation (M)
123 - 130	935.34	934.34	935.47	-1.13	1	K.KVVCMQGR.F Oxidation (M)
149 - 156	912.43	911.42	910.51	0.92	0	R.VMHQLGVK.R
173 - 180	952.35	951.35	951.51	-0.16	0	K.YGDLMLIK.D

No match to: 1059.60, 1092.74, 1482.97

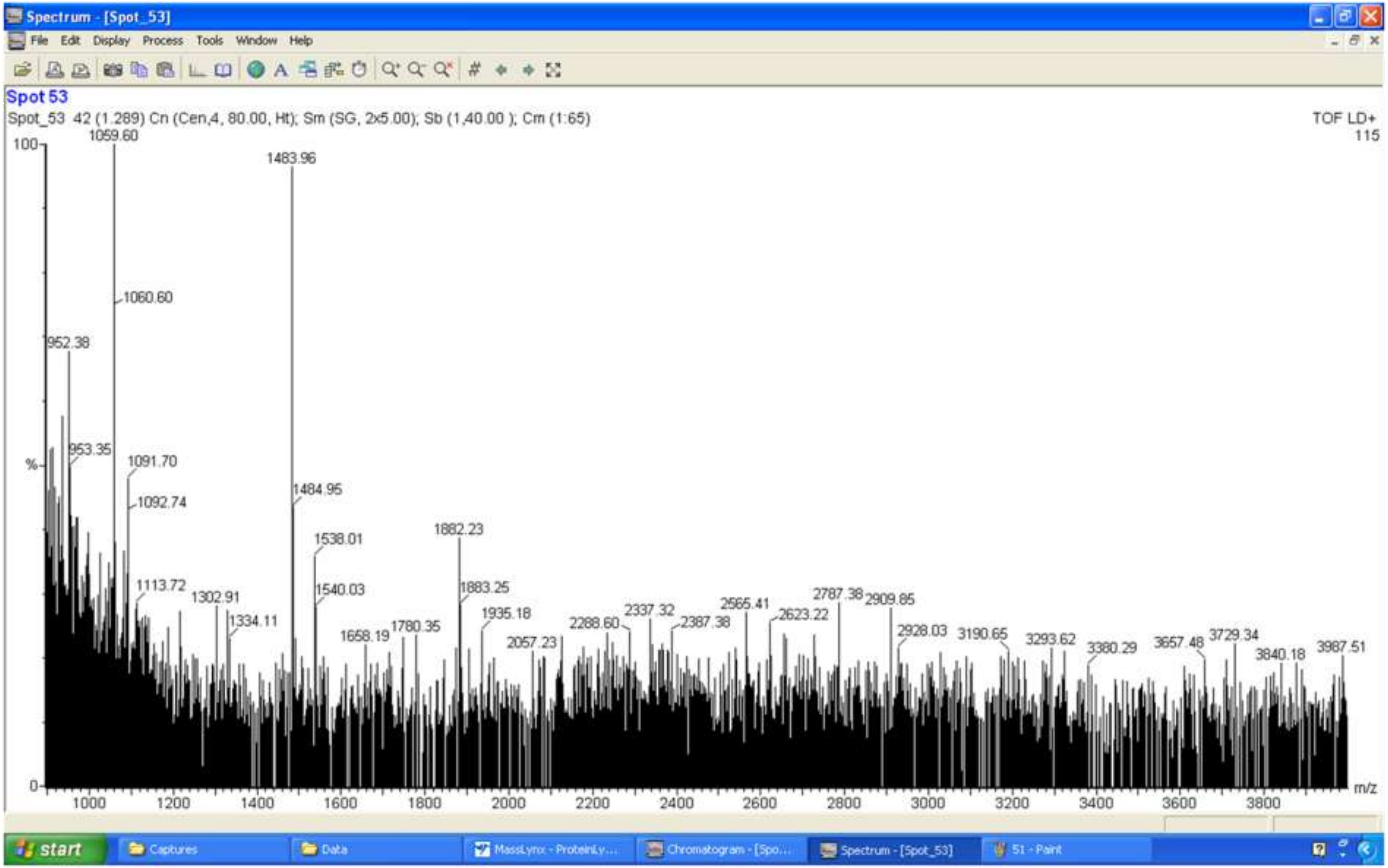

Spectrum - [Spot_53]
File Edit Display Process Tools Window Help
Spot 53
Spot_53 42 (1.289) Cn (Cen,4, 80.00, Ht); Sm (SG, 2x5.00); Sb (1,40.00); Cm (1:65)
TOF LD+
115
100
%
0
1059.60
1483.96
1060.60
952.38
953.35
1091.70
1092.74
1484.95
1882.23
1538.01
1540.03
1883.25
1113.72
1302.91
1334.11
1658.19
1780.35
1935.18
2057.23
2288.60
2337.32
2387.38
2565.41
2623.22
2787.38
2909.85
2928.03
3190.65
3293.62
3380.29
3657.48
3729.34
3840.18
3987.51
1000
1200
1400
1600
1800
2000
2200
2400
2600
2800
3000
3200
3400
3600
3800
m/z
start
Captures
Data
MassLynx - ProteinLy...
Chromatogram - [Spo...
Spectrum - [Spot_53]
51 - Paint

Figure S1.41

Mascot Search Results Spot 54

```
User          : Paul Millares
Email         : paul.millares@gmail.com
Search title  : Spot 54
Database      : Haemonchus 210108 (6387 sequences; 918038 residues)
Timestamp     : 1 Aug 2011 at 10:48:33 GMT
Top Score     : 57 for HCP07180_1, putative nuclear encoded protein  Method: similarity and extension
```

Mascot Score Histogram

Protein score is -10*Log(P), where P is the probability that the observed match is a random event.

Protein scores greater than 51 are significant (p<0.05).

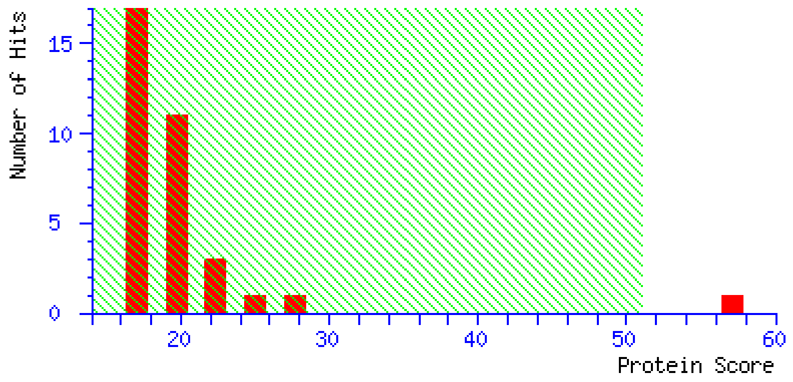

Concise Protein Summary Report

1. HCP07180_1 **Mass:** 19653 **Score:** 57 **Expect:** 0.012 **Matches:** 9

   putative nuclear encoded protein Method: similarity and extension

   HCP07180_2 **Mass:** 21678 **Score:** 54 **Expect:** 0.024 **Matches:** 9

   putative nuclear encoded protein Method: similarity and extension

   HCP12395_1 **Mass:** 17881 **Score:** 24 **Expect:** 24 **Matches:** 5

   putative nuclear encoded protein Method: similarity and extension

HCP07939_1 **Mass:** 12097 **Score:** 19 **Expect:** 73 **Matches:** 3
putative nuclear encoded protein Method: ESTScan
HCP01904_2 **Mass:** 13637 **Score:** 19 **Expect:** 80 **Matches:** 3
putative nuclear encoded protein Method: similarity and extension
HCP10514_1 **Mass:** 12431 **Score:** 19 **Expect:** 86 **Matches:** 3
putative nuclear encoded protein Method: similarity and extension
HCP01904_1 **Mass:** 14927 **Score:** 18 **Expect:** 1e+002 **Matches:** 3
putative nuclear encoded protein Method: similarity and extension
HCP08070_1 **Mass:** 4471 **Score:** 18 **Expect:** 1.1e+002 **Matches:** 2
putative nuclear encoded protein Method: ESTScan

---

2. HCP04783_1 **Mass:** 25162 **Score:** 29 **Expect:** 8.4 **Matches:** 5
putative nuclear encoded protein Method: similarity and extension

---

Search Parameters

Type of search : Peptide Mass Fingerprint
Enzyme : Trypsin
Variable modifications : Carbamidomethyl (C),Glu->pyro-Glu (N-term E),Oxidation (M)
Mass values : Monoisotopic
Protein Mass : Unrestricted
Peptide Mass Tolerance : ± 1.2 Da
Peptide Charge State : 1+
Max Missed Cleavages : 1
Number of queries : 17

Protein View

Match to: **HCP07180_1** Score: **57** Expect: **0.012**
putative nuclear encoded protein Method: similarity and extension

Nominal mass (M_r): **19653**; Calculated pI value: **7.88**
NCBI BLAST search of HCP07180_1 against nr
Unformatted sequence string for pasting into other applications

Variable modifications: Carbamidomethyl (C),Glu->pyro-Glu (N-term E),Oxidation (M)

Cleavage by Trypsin: cuts C-term side of KR unless next residue is P
Number of mass values searched: **17**
Number of mass values matched: **9**
Sequence Coverage: **42%**

Matched peptides shown in **Bold Red**

```
  1 MLALAVLGLI GLVSWSHAAD TVTVTKQVYF DISIGGKPAG RIVIGLFGNA
 51 VPKTAENFLQ LATGSKGYGY KGSKFHRVIK DFMIQGGDFT RGDGTGGKSI
101 YGERFADENF DLHHYGPGWL SMANAGPDTN GSQFFICTVK TPWLDGRHVV
151 FGKVLEGMSV VRKIESTETH PGDRPKEDVV IT
```

Start - End	Observed	Mr(expt)	Mr(calc)	Delta	Miss	Sequence
27 - 41	1608.19	1607.18	1606.85	0.33	0	K.QVYFDISIGGKPAGR.I
42 - 53	1227.04	1226.03	1226.74	-0.71	0	R.IVIGLFGNAVPK.T
42 - 53	1228.13	1227.12	1226.74	0.38	0	R.IVIGLFGNAVPK.T
54 - 66	1380.07	1379.07	1378.71	0.36	0	K.TAENFLQLATGSK.G
78 - 91	1643.15	1642.14	1641.82	0.32	1	R.VIKDFMIQGGDFTR.G Oxidation (M)
81 - 91	1286.95	1285.94	1285.58	0.37	0	K.DFMIQGGDFTR.G
81 - 91	1302.94	1301.94	1301.57	0.36	0	K.DFMIQGGDFTR.G Oxidation (M)
154 - 162	1005.68	1004.67	1004.53	0.14	0	K.VLEGMSVVR.K Oxidation (M)
163 - 176	1595.16	1594.15	1593.81	0.34	1	R.KIESTETHPGDRPK.E

No match to: 1027.66, 1249.01, 1250.10, 1272.07, 1324.93, 1483.04, 1591.17, 2234.42

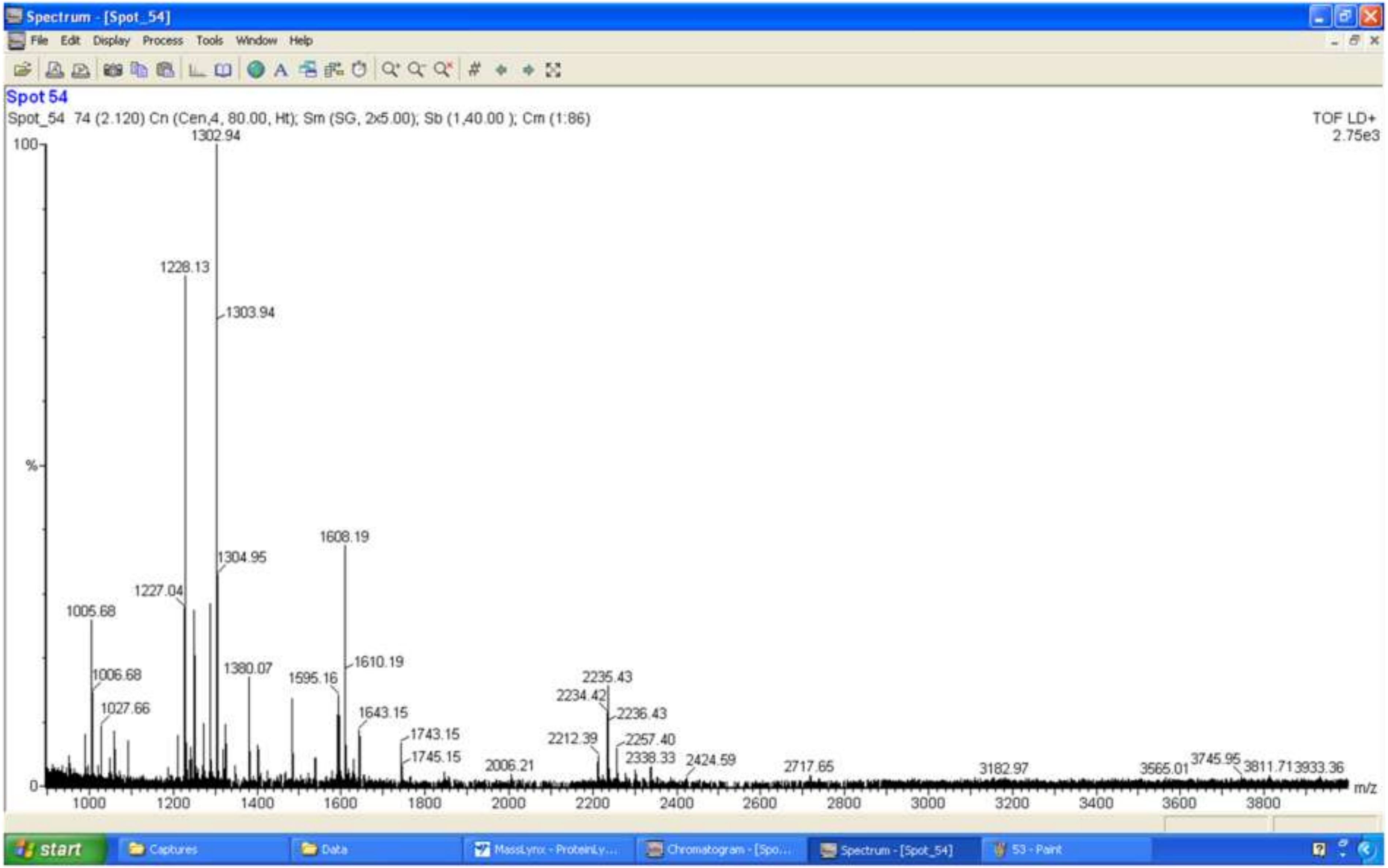
Spectrum - [Spot_54]
File Edit Display Process Tools Window Help
Spot 54
Spot_54 74 (2.120) Cn (Cen,4, 80.00, Ht); Sm (SG, 2x5.00); Sb (1,40.00); Cm (1:86)
TOF LD+
2.75e3
100
%
0
1302.94
1228.13
1303.94
1608.19
1304.95
1227.04
1005.68
1610.19
1380.07
1595.16
1006.68
1027.66
1643.15
1743.15
1745.15
2006.21
2235.43
2234.42
2236.43
2212.39
2257.40
2338.33
2424.59
2717.65
3182.97
3565.01
3745.95
3811.71
3933.36
1000 1200 1400 1600 1800 2000 2200 2400 2600 2800 3000 3200 3400 3600 3800
m/z
start
Captures
Data
MassLynx - ProteinLy...
Chromatogram - [Spo...
Spectrum - [Spot_54]
53 - Paint

Figure S1.42

Mascot Search Results Spot 57

```
User            : Paul Millares
Email           : paul.millares@gmail.com
Search title    : Spot 57
Database        : Haemonchus 210108 (6387 sequences; 918038 residues)
Timestamp       : 1 Aug 2011 at 10:49:32 GMT
Top Score       : 35 for HCP06327_1, putative nuclear encoded protein  Method: similarity and extension
```

Mascot Score Histogram

Protein score is -10*Log(P), where P is the probability that the observed match is a random event.

Protein scores greater than 51 are significant (p<0.05).

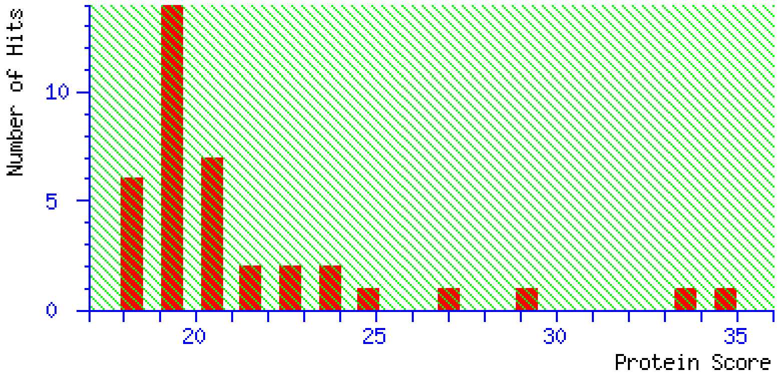

Concise Protein Summary Report

1. HCP06327_1 **Mass:** 18739 **Score:** 35 **Expect:** 2.2 **Matches:** 7
   putative nuclear encoded protein Method: similarity and extension
   HCP06327_2 **Mass:** 21952 **Score:** 30 **Expect:** 6 **Matches:** 6
   putative nuclear encoded protein Method: similarity and extension

Search Parameters

**Type of search**	:	**Peptide Mass Fingerprint**
**Enzyme**	:	**Trypsin**
**Variable modifications**	:	Carbamidomethyl (C),Glu->pyro-Glu (N-term E),Oxidation (M)
**Mass values**	:	**Monoisotopic**
**Protein Mass**	:	**Unrestricted**
**Peptide Mass Tolerance**	:	**± 1.2 Da**
**Peptide Charge State**	:	**1+**
**Max Missed Cleavages**	:	**1**
**Number of queries**	:	**23**

Protein View

Match to: **HCP06327_1** Score: **35** Expect: **2.2**
putative nuclear encoded protein Method: similarity and extension

Nominal mass (M_r): **18739**; Calculated pI value: **5.96**
NCBI BLAST search of HCP06327_1 against nr
Unformatted sequence string for pasting into other applications

Variable modifications: Carbamidomethyl (C),Glu->pyro-Glu (N-term E),Oxidation (M)
Cleavage by Trypsin: cuts C-term side of KR unless next residue is P
Number of mass values searched: **23**
Number of mass values matched: **7**
Sequence Coverage: **34%**

Matched peptides shown in **Bold Red**

1 **IGMSSPSSGK** R**RMDTDVIK**L IESK**HEVNIT** **GGLNEFNVK**F YGPSGTAYEG
51 GVWRVRVELP EKYPFK**SPSI** **GFMNK**IFHPN IDEASGSVCL DVINQAWTAL
101 YDLANIFESF LPQLLTYPNP TDPLNGDAAA LYLHKPEEFK KK**CKDYVERF**
151 **ASEDALR**RFF NESSCK

Start - End	Observed	Mr(expt)	Mr(calc)	Delta	Miss	Sequence
1 - 10	951.60	950.60	949.45	1.14	0	-.IGMSSPSSGK.R
1 - 10	965.82	964.81	965.45	-0.64	0	-.IGMSSPSSGK.R Oxidation (M)
12 - 19	994.64	993.63	992.50	1.14	1	R.RMDTDVIK.L Oxidation (M)
25 - 39	1671.44	1670.43	1669.84	0.59	0	K.HEVNITGGLNEFNVK.F
67 - 75	997.63	996.62	995.47	1.15	0	K.SPSIGFMNK.I Oxidation (M)
143 - 149	968.61	967.60	968.44	-0.84	1	K.CKDYVER.F Carbamidomethyl (C)
150 - 157	907.62	906.62	907.44	-0.82	0	R.FASEDALR.R

No match to: 900.58, 915.59, 925.62, 927.62, 984.75, 1059.81, 1115.93, 1132.01, 1148.04, 1164.09, 1180.16, 1227.15, 1244.11, 1350.30, 2211.79, 2263.87

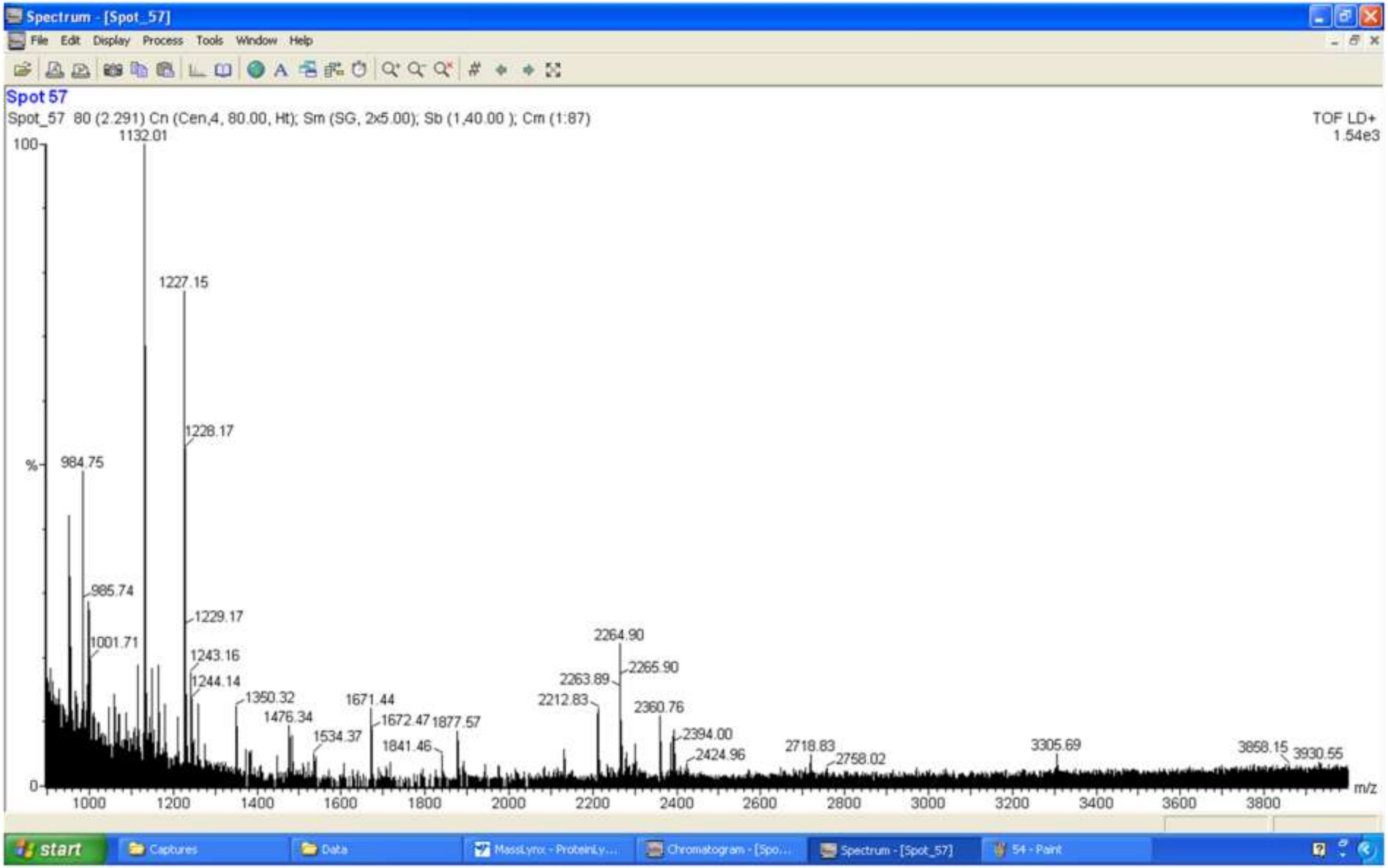

Figure S1.43

Mascot Search Results Spot 59

```
User           : Paul Millares
Email          : paul.millares@gmail.com
Search title   : Spot 59
Database       : Haemonchus 210108 (6387 sequences; 918038 residues)
Timestamp      : 1 Aug 2011 at 10:49:57 GMT
Top Score      : 53 for HCP06327_1, putative nuclear encoded protein  Method: similarity and extension
```

Mascot Score Histogram

Protein score is -10*Log(P), where P is the probability that the observed match is a random event.

Protein scores greater than 51 are significant (p<0.05).

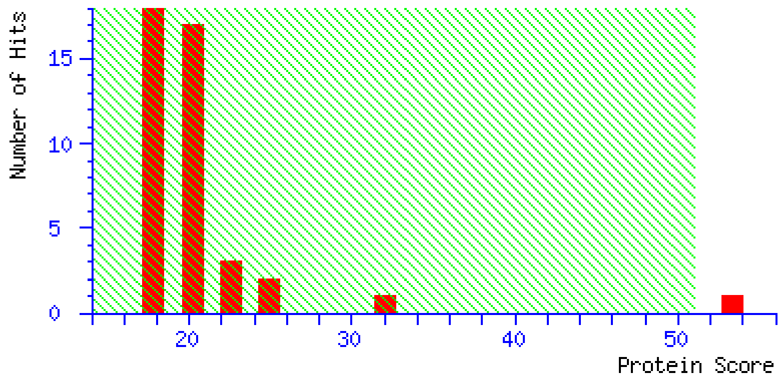

Concise Protein Summary Report

1. HCP06327_1 **Mass:** 18739 **Score:** 53 **Expect:** 0.03 **Matches:** 9
   putative nuclear encoded protein Method: similarity and extension

---

2. HCP06327_2 **Mass:** 21952 **Score:** 33 **Expect:** 3.6 **Matches:** 8
   putative nuclear encoded protein Method: similarity and extension

---

Search Parameters

Type of search : Peptide Mass Fingerprint
Enzyme : Trypsin
Variable modifications : Carbamidomethyl (C),Glu->pyro-Glu (N-term E),Oxidation (M)
Mass values : Monoisotopic
Protein Mass : Unrestricted
Peptide Mass Tolerance : ± 1.2 Da
Peptide Charge State : 1+
Max Missed Cleavages : 1
Number of queries : 30

Protein View

Match to: **HCP06327_1** Score: **53** Expect: **0.03**
putative nuclear encoded protein Method: similarity and extension

Nominal mass (M_r): **18739**; Calculated pI value: **5.96**
NCBI BLAST search of HCP06327_1 against nr
Unformatted sequence string for pasting into other applications

Variable modifications: Carbamidomethyl (C),Glu->pyro-Glu (N-term E),Oxidation (M)
Cleavage by Trypsin: cuts C-term side of KR unless next residue is P
Number of mass values searched: **30**
Number of mass values matched: **9**
Sequence Coverage: **43%**

Matched peptides shown in **Bold Red**

```
  1 IGMSSPSSGK RRMDTDVIKL IESKHEVNIT GGLNEFNVKF YGPSGTAYEG
 51 GVWRVRVELP EKYPFKSPSI GFMNKIFHPN IDEASGSVCL DVINQAWTAL
101 YDLANIFESF LPQLLTYPNP TDPLNGDAAA LYLHKPEEFK KKCKDYVERF
151 ASEDALRRFF NESSCK
```

Start - End	Observed	Mr(expt)	Mr(calc)	Delta	Miss	Sequence
1 - 10	950.92	949.91	949.45	0.46	0	-.IGMSSPSSGK.R
1 - 11	1122.10	1121.09	1121.55	-0.46	1	-.IGMSSPSSGKR.R Oxidation (M)
12 - 19	993.93	992.92	992.50	0.43	1	R.RMDTDVIK.L Oxidation (M)
13 - 24	1407.26	1406.26	1406.73	-0.48	1	R.MDTDVIKLIESK.H Oxidation (M)
25 - 39	1671.34	1670.34	1669.84	0.50	0	K.HEVNITGGLNEFNVK.F
67 - 75	980.96	979.95	979.48	0.47	0	K.SPSIGFMNK.I
143 - 149	968.92	967.91	968.44	-0.53	1	K.CKDYVER.F Carbamidomethyl (C)
150 - 157	908.86	907.85	907.44	0.41	0	R.FASEDALR.R

158 - 166   1116.96 1115.95 1116.50   -0.55   1 R.RFFNESSCK.-

No match to: 898.83, 915.88, 922.90, 923.60, 936.92, 952.94, 964.92, 1008.98, 1031.82, 1051.92, 1053.78, 1061.22, 1066.26, 1150.13, 1279.17, 1522.19, 1824.32, 1841.32, 1892.42, 1909.39, 2130.47

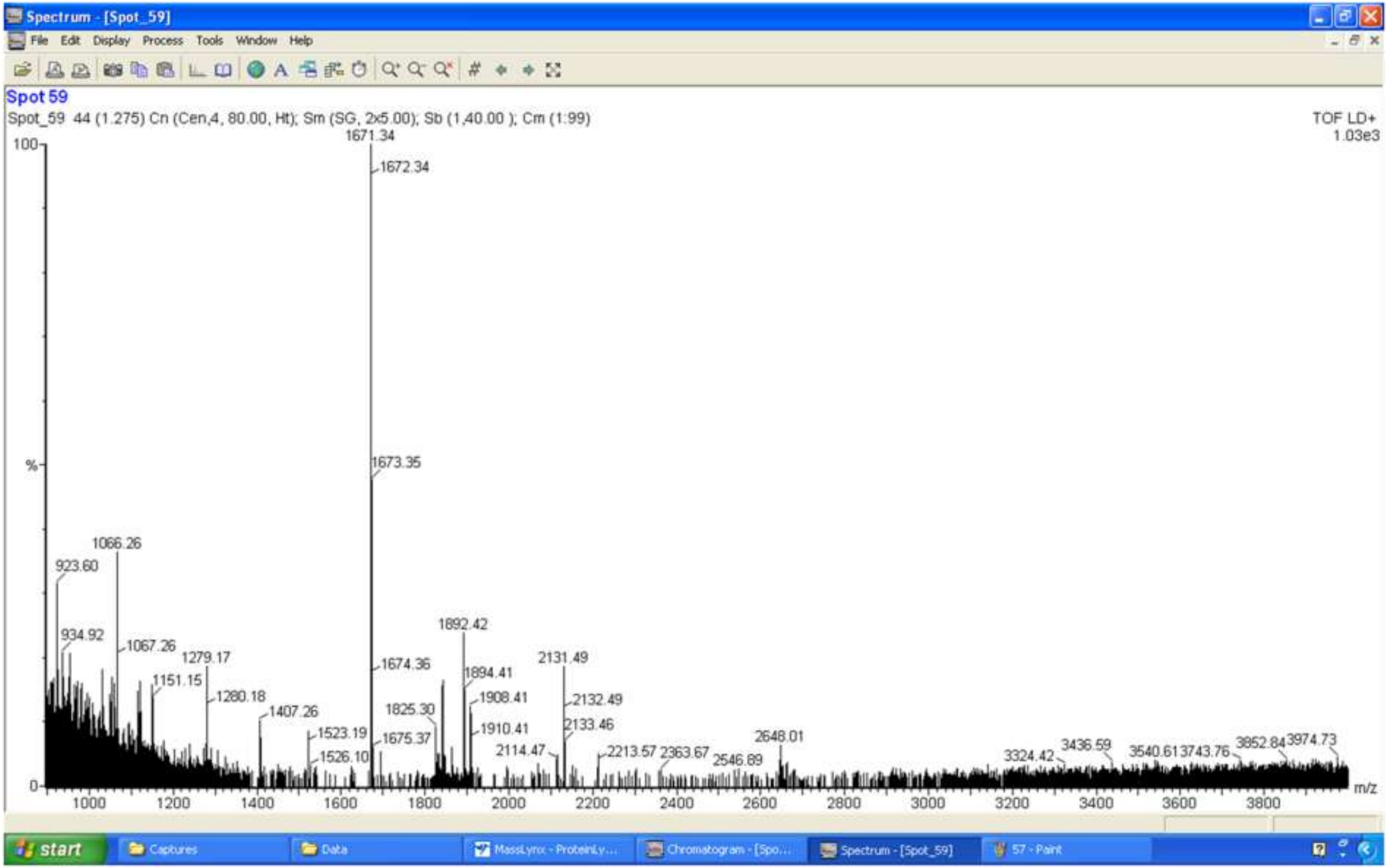

Spectrum - [Spot_59]
Spot 59
Spot_59 44 (1.275) Cn (Cen,4, 80.00, Ht); Sm (SG, 2x5.00); Sb (1,40.00); Cm (1:99)
TOF LD+
1.03e3
100
%
0
1671.34
1672.34
1673.35
1674.36
1675.37
1066.26
923.60
934.92
1067.26
1279.17
1151.15
1280.18
1407.26
1523.19
1526.10
1825.30
1892.42
1894.41
1908.41
1910.41
2131.49
2132.49
2133.46
2114.47
2213.57
2363.67
2546.89
2648.01
3324.42
3436.59
3540.61
3743.76
3852.84
3974.73
m/z
1000
1200
1400
1600
1800
2000
2200
2400
2600
2800
3000
3200
3400
3600
3800

Figure S1.44

Mascot Search Results Spot 60

```
User            : Paul Millares
Email           : paul.millares@gmail.com
Search title    : Spot 60
Database        : Haemonchus 210108 (6387 sequences; 918038 residues)
Timestamp       : 1 Aug 2011 at 10:50:27 GMT
Top Score       : 43 for HCP00814_1, putative nuclear encoded protein  Method: similarity and extension
```

Mascot Score Histogram

Protein score is -10*Log(P), where P is the probability that the observed match is a random event.

Protein scores greater than 51 are significant (p<0.05).

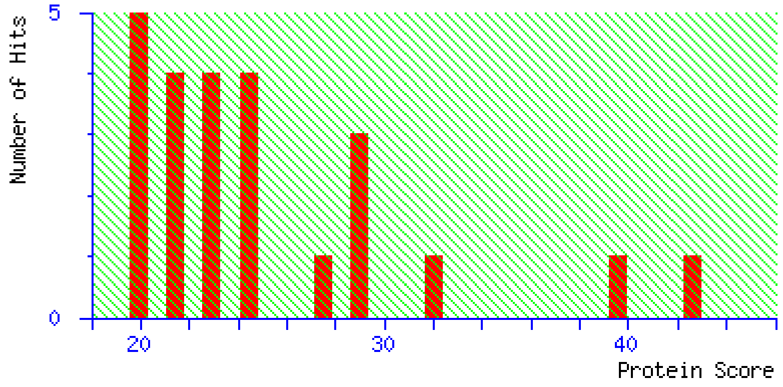

Concise Protein Summary Report

1. HCP00814_1 **Mass:** 18339 **Score:** 43 **Expect:** 0.35 **Matches:** 6
   putative nuclear encoded protein Method: similarity and extension
   HCP12626_1 **Mass:** 18042 **Score:** 42 **Expect:** 0.43 **Matches:** 6
   putative nuclear encoded protein Method: similarity and extension
   HCP00814_2 **Mass:** 18335 **Score:** 41 **Expect:** 0.54 **Matches:** 6
   putative nuclear encoded protein Method: similarity and extension

HCP00814_3 **Mass:** 18335 **Score:** 41 **Expect:** 0.54 **Matches:** 6
putative nuclear encoded protein Method: similarity and extension
HCP00814_4 **Mass:** 18335 **Score:** 41 **Expect:** 0.54 **Matches:** 6
putative nuclear encoded protein Method: similarity and extension
HCP11063_1 **Mass:** 12053 **Score:** 32 **Expect:** 3.8 **Matches:** 4
putative nuclear encoded protein Method: similarity and extension
HCP02260_1 **Mass:** 18509 **Score:** 31 **Expect:** 4.8 **Matches:** 5
putative nuclear encoded protein Method: similarity and extension
HCP02260_2 **Mass:** 18469 **Score:** 31 **Expect:** 4.8 **Matches:** 5
putative nuclear encoded protein Method: similarity and extension
HCP02260_3 **Mass:** 18503 **Score:** 24 **Expect:** 28 **Matches:** 4
putative nuclear encoded protein Method: similarity and extension
HCP10392_1 **Mass:** 3982 **Score:** 20 **Expect:** 64 **Matches:** 2
putative nuclear encoded protein Method: ESTScan

Search Parameters

Type of search : Peptide Mass Fingerprint
Enzyme : Trypsin
Variable modifications : Carbamidomethyl (C),Glu->pyro-Glu (N-term E),Oxidation (M)
Mass values : Monoisotopic
Protein Mass : Unrestricted
Peptide Mass Tolerance : ± 1.2 Da
Peptide Charge State : 1+
Max Missed Cleavages : 1
Number of queries : 12

Protein View

Match to: **HCP00814_1** Score: **43** Expect: **0.35**
putative nuclear encoded protein Method: similarity and extension

Nominal mass (M_r): **18339**; Calculated pI value: **6.23**
NCBI BLAST search of HCP00814_1 against nr
Unformatted sequence string for pasting into other applications

Variable modifications: Carbamidomethyl (C),Glu->pyro-Glu (N-term E),Oxidation (M)
Cleavage by Trypsin: cuts C-term side of KR unless next residue is P
Number of mass values searched: **12**
Number of mass values matched: **6**
Sequence Coverage: **32%**

Matched peptides shown in **Bold Red**

```
  1 MALWPIDRVE RMMEEPFRRM GRYCPIRDMD WMAQDMMPYW RDADHSMLHV
 51 GNQTNFMIND DKKFSVALDV SHFKPEELKV QLDGRDLTIE GHQEVKTEHG
101 YMKKNFVHRW ALPEDCDLDA VHTQLDNNGQ LSVEAPKTGQ HTKTRNIPIM
151 AAPKKK
```

Start - End	Observed	Mr(expt)	Mr(calc)	Delta	Miss	Sequence
12 - 18	971.60	970.59	970.39	0.20	0	R.MMEEPFR.R 2 Oxidation (M)
12 - 19	1127.90	1126.90	1126.49	0.41	1	R.MMEEPFRR.M 2 Oxidation (M)
20 - 27	996.52	995.51	994.48	1.03	1	R.MGRYCPIR.D
64 - 79	1846.45	1845.44	1844.97	0.47	0	K.FSVALDVSHFKPEELK.V
86 - 96	1269.11	1268.10	1267.64	0.46	0	R.DLTIEGHQEVK.T
138 - 145	928.69	927.68	927.49	0.20	1	K.TGQHTKTR.N

No match to: 900.48, 907.49, 912.70, 925.67, 944.69, 951.49

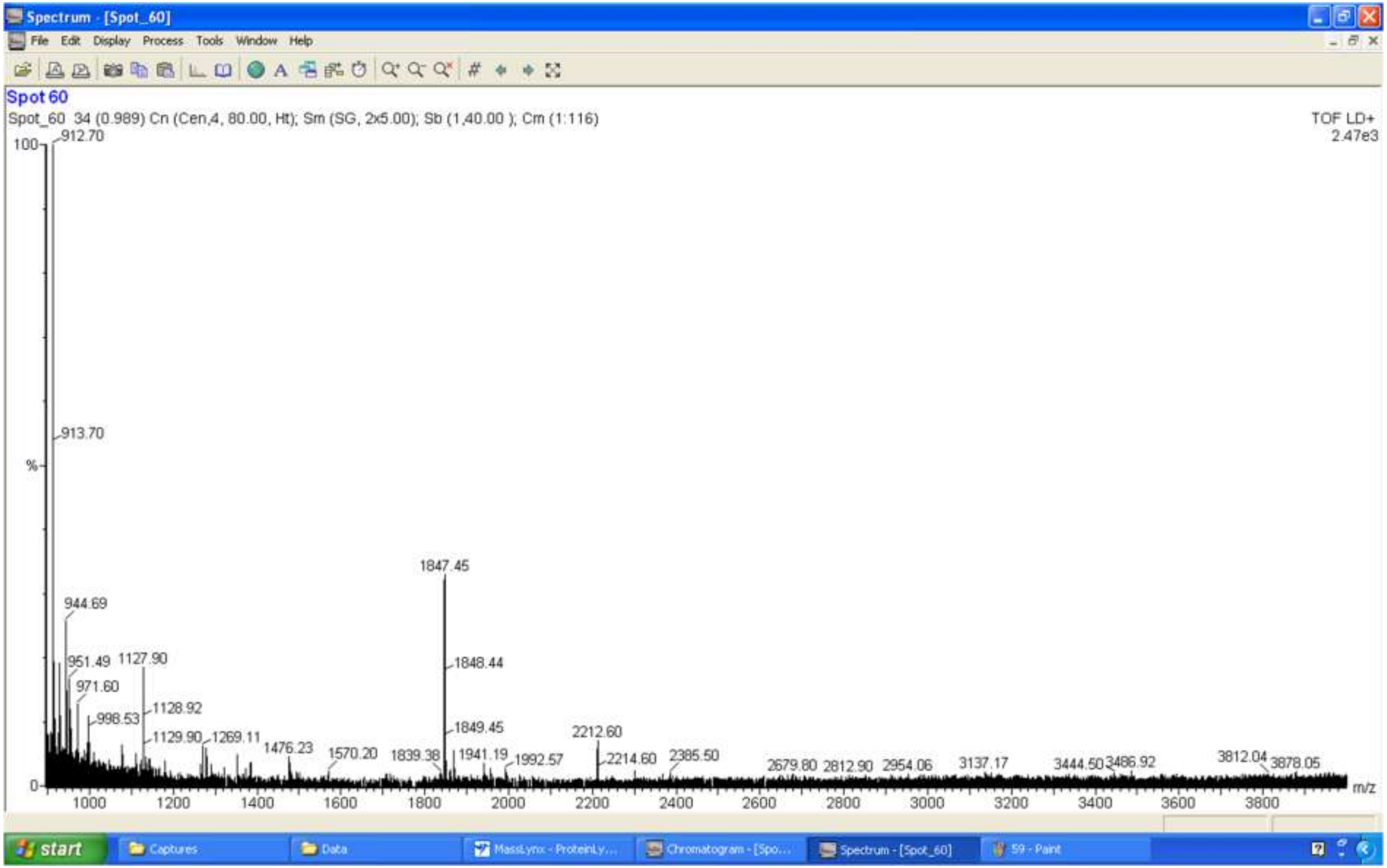
Spectrum - [Spot_60]
File Edit Display Process Tools Window Help
Spot 60
Spot_60 34 (0.989) Cn (Cen,4, 80.00, Ht); Sm (SG, 2x5.00); Sb (1,40.00); Cm (1:116)
TOF LD+
2.47e3
100
%
0
912.70
913.70
944.69
951.49
971.60
998.53
1127.90
1128.92
1129.90
1269.11
1476.23
1570.20
1839.38
1847.45
1848.44
1849.45
1941.19
1992.57
2212.60
2214.60
2385.50
2679.80
2812.90
2954.06
3137.17
3444.50
3486.92
3812.04
3878.05
1000 1200 1400 1600 1800 2000 2200 2400 2600 2800 3000 3200 3400 3600 3800
m/z
start
Captures
Data
MassLynx - ProteinLy...
Chromatogram - [Spo...
Spectrum - [Spot_60]
59 - Paint

Figure S1.45

Mascot Search Results Spot 61

```
User          : Paul Millares
Email         : paul.millares@gmail.com
Search title  : Spot 61
Database      : Haemonchus 210108 (6387 sequences; 918038 residues)
Timestamp     : 1 Aug 2011 at 10:52:15 GMT
Top Score     : 59 for HCP02260_2, putative nuclear encoded protein  Method: similarity and extension
```

Mascot Score Histogram

Protein score is -10*Log(P), where P is the probability that the observed match is a random event.

Protein scores greater than 51 are significant (p<0.05).

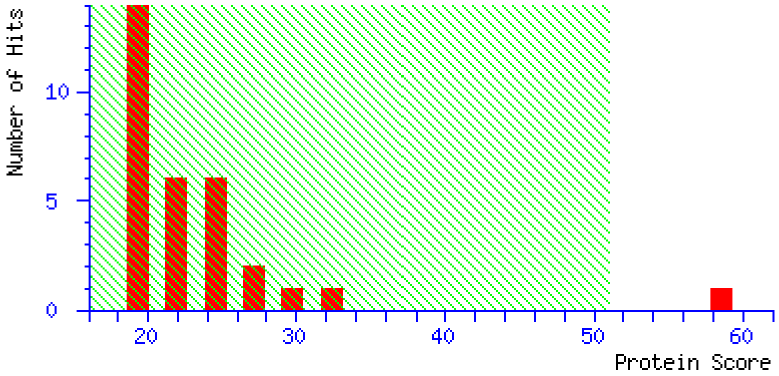

Concise Protein Summary Report

1. HCP02260_2 **Mass:** 18469 **Score:** 59 **Expect:** 0.009 **Matches:** 9

   putative nuclear encoded protein Method: similarity and extension

   HCP02260_1 **Mass:** 18509 **Score:** 46 **Expect:** 0.18 **Matches:** 8

   putative nuclear encoded protein Method: similarity and extension

   HCP02260_3 **Mass:** 18503 **Score:** 41 **Expect:** 0.46 **Matches:** 8

   putative nuclear encoded protein Method: similarity and extension

HCP11063_1 **Mass:** 12053 **Score:** 35 **Expect:** 2.1 **Matches:** 5
putative nuclear encoded protein Method: similarity and extension
HCP02926_3 **Mass:** 4932 **Score:** 34 **Expect:** 2.8 **Matches:** 4
putative nuclear encoded protein Method: Longest ORF
HCP12626_1 **Mass:** 18042 **Score:** 33 **Expect:** 3.6 **Matches:** 6
putative nuclear encoded protein Method: similarity and extension
HCP00814_2 **Mass:** 18335 **Score:** 32 **Expect:** 4.4 **Matches:** 6
putative nuclear encoded protein Method: similarity and extension
HCP00814_3 **Mass:** 18335 **Score:** 32 **Expect:** 4.4 **Matches:** 6
putative nuclear encoded protein Method: similarity and extension
HCP00814_4 **Mass:** 18335 **Score:** 32 **Expect:** 4.4 **Matches:** 6
putative nuclear encoded protein Method: similarity and extension
HCP00814_1 **Mass:** 18339 **Score:** 26 **Expect:** 15 **Matches:** 6
putative nuclear encoded protein Method: similarity and extension
HCP02379_1 **Mass:** 11420 **Score:** 20 **Expect:** 65 **Matches:** 3
putative nuclear encoded protein Method: ESTScan
HCP02583_1 **Mass:** 13887 **Score:** 20 **Expect:** 67 **Matches:** 4
putative nuclear encoded protein Method: similarity and extension
HCP01640_1 **Mass:** 9672 **Score:** 20 **Expect:** 70 **Matches:** 3
putative nuclear encoded protein Method: similarity and extension

---

2. HCP06327_1 **Mass:** 18739 **Score:** 32 **Expect:** 3.9 **Matches:** 5
putative nuclear encoded protein Method: similarity and extension
HCP06327_2 **Mass:** 21952 **Score:** 29 **Expect:** 7.7 **Matches:** 5
putative nuclear encoded protein Method: similarity and extension

---

Search Parameters

Type of search : Peptide Mass Fingerprint
Enzyme : Trypsin
Variable modifications : Carbamidomethyl (C),Glu->pyro-Glu (N-term E),Oxidation (M)

Mass values : Monoisotopic
Protein Mass : Unrestricted
Peptide Mass Tolerance : ± 1.2 Da
Peptide Charge State : 1+
Max Missed Cleavages : 1
Number of queries : 16

Protein View

Match to: **HCP02260_2** Score: **59** Expect: **0.009**
putative nuclear encoded protein Method: similarity and extension

Nominal mass (M_r): **18469**; Calculated pI value: **6.30**
NCBI BLAST search of HCP02260_2 against nr
Unformatted sequence string for pasting into other applications

Variable modifications: Carbamidomethyl (C),Glu->pyro-Glu (N-term E),Oxidation (M)
Cleavage by Trypsin: cuts C-term side of KR unless next residue is P
Number of mass values searched: **16**
Number of mass values matched: **9**
Sequence Coverage: **42%**

Matched peptides shown in **Bold Red**

1 MALWPVDRLE R**MMEEPFRR**M ERFNPMR**DMD** **WMSRQIMPYW** **R**NADHSMLHV
51 GNVTKELVND DKK**FAVALDV** **SHFRPEELK**V QLEGR**DLTIE** **GHQEVK**TEHG
101 YIEKNFVHRW ALPEDCDLDA VHTQIDNNGH LSVEAPK**TGQ** **HTKTRNIPIM**
151 **PAPK**RK

Start - End	Observed	Mr(expt)	Mr(calc)	Delta	Miss	Sequence
12 - 18	971.57	970.56	970.39	0.17	0	R.MMEEPFR.R 2 Oxidation (M)
12 - 19	1127.87	1126.86	1126.49	0.37	1	R.MMEEPFRR.M 2 Oxidation (M)
28 - 34	957.50	956.49	955.35	1.14	0	R.DMDWMSR.Q Oxidation (M)
35 - 41	992.63	991.62	992.49	-0.87	0	R.QIMPYWR.N
35 - 41	1009.67	1008.66	1008.49	0.18	0	R.QIMPYWR.N Oxidation (M)
64 - 79	1858.39	1857.38	1856.98	0.41	0	K.FAVALDVSHFRPEELK.V
86 - 96	1269.07	1268.06	1267.64	0.42	0	R.DLTIEGHQEVK.T
138 - 145	928.50	927.49	927.49	0.00	1	K.TGQHTKTR.N
146 - 154	996.73	995.72	995.55	0.17	0	R.NIPIMPAPK.R Oxidation (M)

No match to: 898.66, 907.48, 911.62, 914.66, 930.65, 951.45, 1357.10

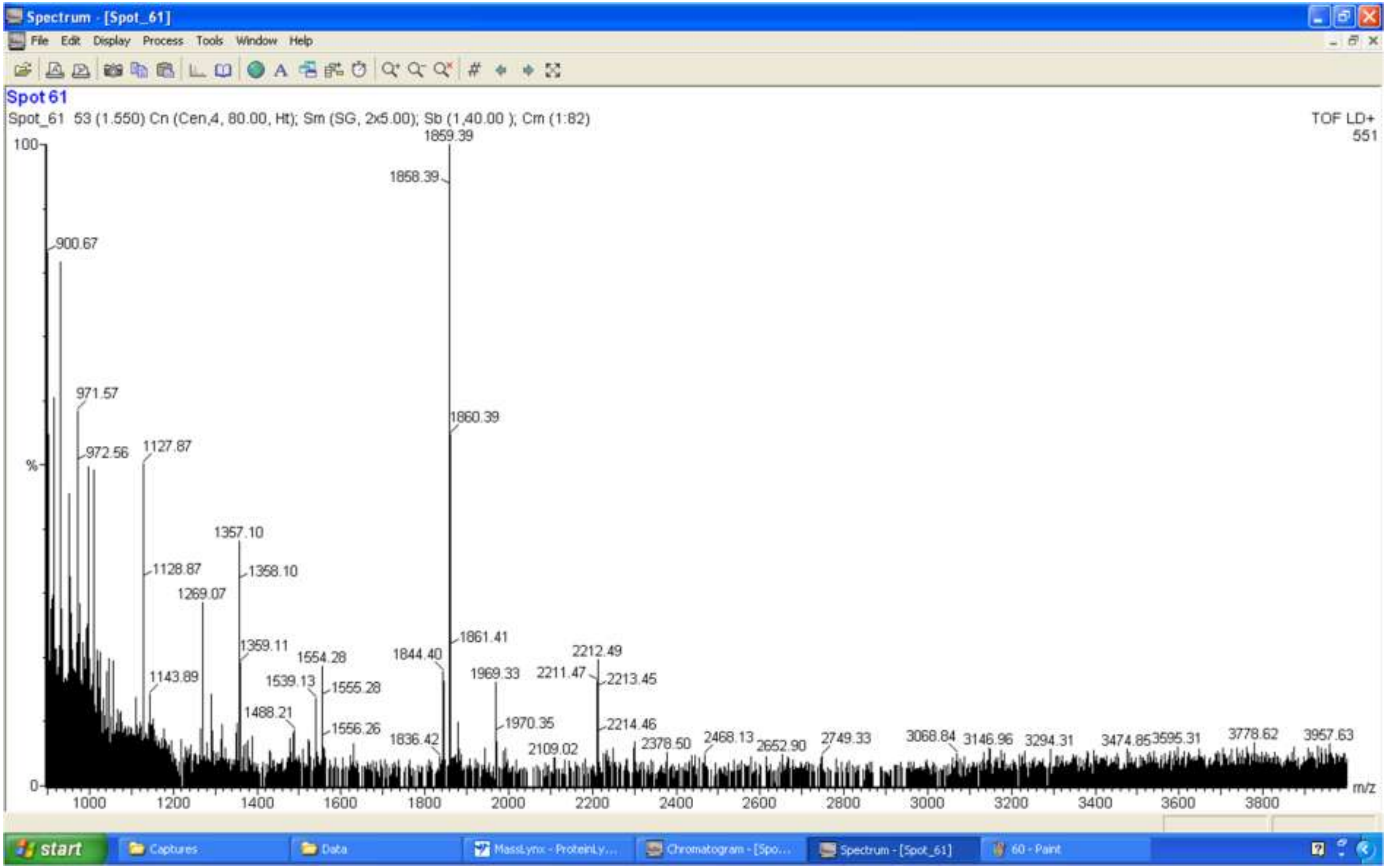

Spectrum - [Spot_61]
File Edit Display Process Tools Window Help
Spot 61
Spot_61 53 (1.550) Cn (Cen,4, 80.00, Ht); Sm (SG, 2x5.00); Sb (1,40.00); Cm (1:82)
TOF LD+
551
1859.39
1858.39
900.67
971.57
972.56
1127.87
1860.39
1357.10
1128.87
1358.10
1269.07
1359.11
1554.28
1844.40
1861.41
2212.49
1143.89
1539.13
1555.28
1969.33
2211.47
2213.45
1488.21
1556.26
1970.35
2214.46
1836.42
2109.02
2378.50
2468.13
2652.90
2749.33
3068.84
3146.96
3294.31
3474.85
3595.31
3778.62
3957.63
100
%
0
1000 1200 1400 1600 1800 2000 2200 2400 2600 2800 3000 3200 3400 3600 3800
m/z
start
Captures
Data
MassLynx - ProteinLy...
Chromatogram - [Spo...
Spectrum - [Spot_61]
60 - Paint

Figure S1.46

Mascot Search Results Spot 62

```
User            : Paul Millares
Email           : paul.millares@gmail.com
Search title    : Spot 62
Database        : Haemonchus 210108 (6387 sequences; 918038 residues)
Timestamp       : 1 Aug 2011 at 10:52:51 GMT
Top Score       : 48 for HCP03240_3, putative nuclear encoded protein  Method: similarity and extension
```

Mascot Score Histogram

Protein score is -10*Log(P), where P is the probability that the observed match is a random event.

Protein scores greater than 51 are significant (p<0.05).

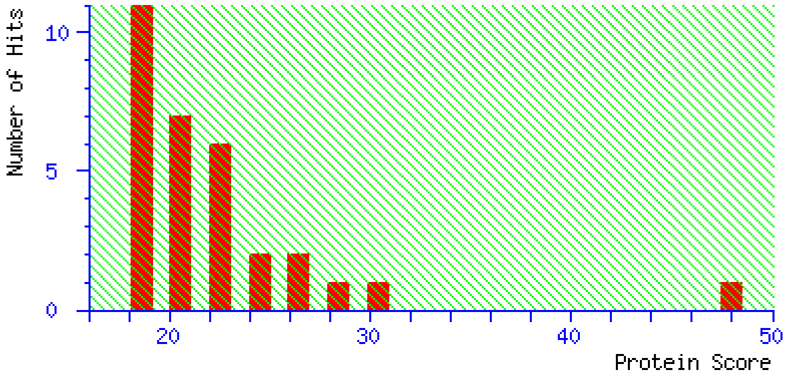

Concise Protein Summary Report

1. HCP03240_3 **Mass:** 23007 **Score:** 48 **Expect:** 0.1 **Matches:** 6
   putative nuclear encoded protein Method: similarity and extension
   HCP03240_1 **Mass:** 23453 **Score:** 48 **Expect:** 0.11 **Matches:** 6
   putative nuclear encoded protein Method: similarity and extension
   HCP03240_2 **Mass:** 23741 **Score:** 25 **Expect:** 19 **Matches:** 4
   putative nuclear encoded protein Method: similarity and extension

[HCP01259_1](#) **Mass:** 19231 **Score:** 21 **Expect:** 48 **Matches:** 3
putative nuclear encoded protein Method: similarity and extension
[HCP02686_1](#) **Mass:** 14532 **Score:** 21 **Expect:** 56 **Matches:** 3
putative nuclear encoded protein Method: similarity and extension

---

Search Parameters

Type of search : Peptide Mass Fingerprint
Enzyme : Trypsin
Variable modifications : Carbamidomethyl (C),Glu->pyro-Glu (N-term E),Oxidation (M)
Mass values : Monoisotopic
Protein Mass : Unrestricted
Peptide Mass Tolerance : ± 1.2 Da
Peptide Charge State : 1+
Max Missed Cleavages : 1
Number of queries : 13

Protein View

Match to: **HCP03240_3** Score: **48** Expect: **0.1**
putative nuclear encoded protein Method: similarity and extension

Nominal mass (M_r): **23007**; Calculated pI value: **8.69**
NCBI BLAST search of HCP03240_3 against nr
Unformatted sequence string for pasting into other applications

Variable modifications: Carbamidomethyl (C),Glu->pyro-Glu (N-term E),Oxidation (M)
Cleavage by Trypsin: cuts C-term side of KR unless next residue is P
Number of mass values searched: **13**
Number of mass values matched: **6**
Sequence Coverage: **40%**

Matched peptides shown in **Bold Red**

```
  1 FGTGLTRGLV RPLAALPTVQ RSFASMAAEA FKKHEVVPDV LATAPTKVVK
 51 AHYDSGAEVN LGNVLTPTQV KNPPKLTWDA EPGALYTVIF TDPDAPSRKE
101 ATFREWHHWL VVNVPGNDIN KGDVLAEYIG SGPPKDTGLH RYVFLVYKQP
151 SGRITDSEHG HLTNRSGDGR GGFKTEKFVA KHKLGTPIAG NFYQAEWDDY
201 VPILYKQLGA
```

Start - End	Observed	Mr(expt)	Mr(calc)	Delta	Miss	Sequence
34 - 47	1477.26	1476.25	1475.80	0.45	0	K.HEVVPDVLATAPTK.V
51 - 71	2213.57	2212.57	2212.11	0.45	0	K.AHYDSGAEVNLGNVLTPTQVK.N
105 - 121	2057.47	2056.46	2056.03	0.43	0	R.EWHHWLVVNVPGNDINK.G
122 - 135	1403.15	1402.14	1401.71	0.43	0	K.GDVLAEYIGSGPPK.D
142 - 148	931.73	930.72	930.52	0.20	0	R.YVFLVYK.Q
154 - 165	1380.11	1379.10	1378.66	0.44	0	R.ITDSEHGHLTNR.S

No match to: 907.47, 951.48, 996.48, 1122.05, 1448.09, 1465.11, 1666.30

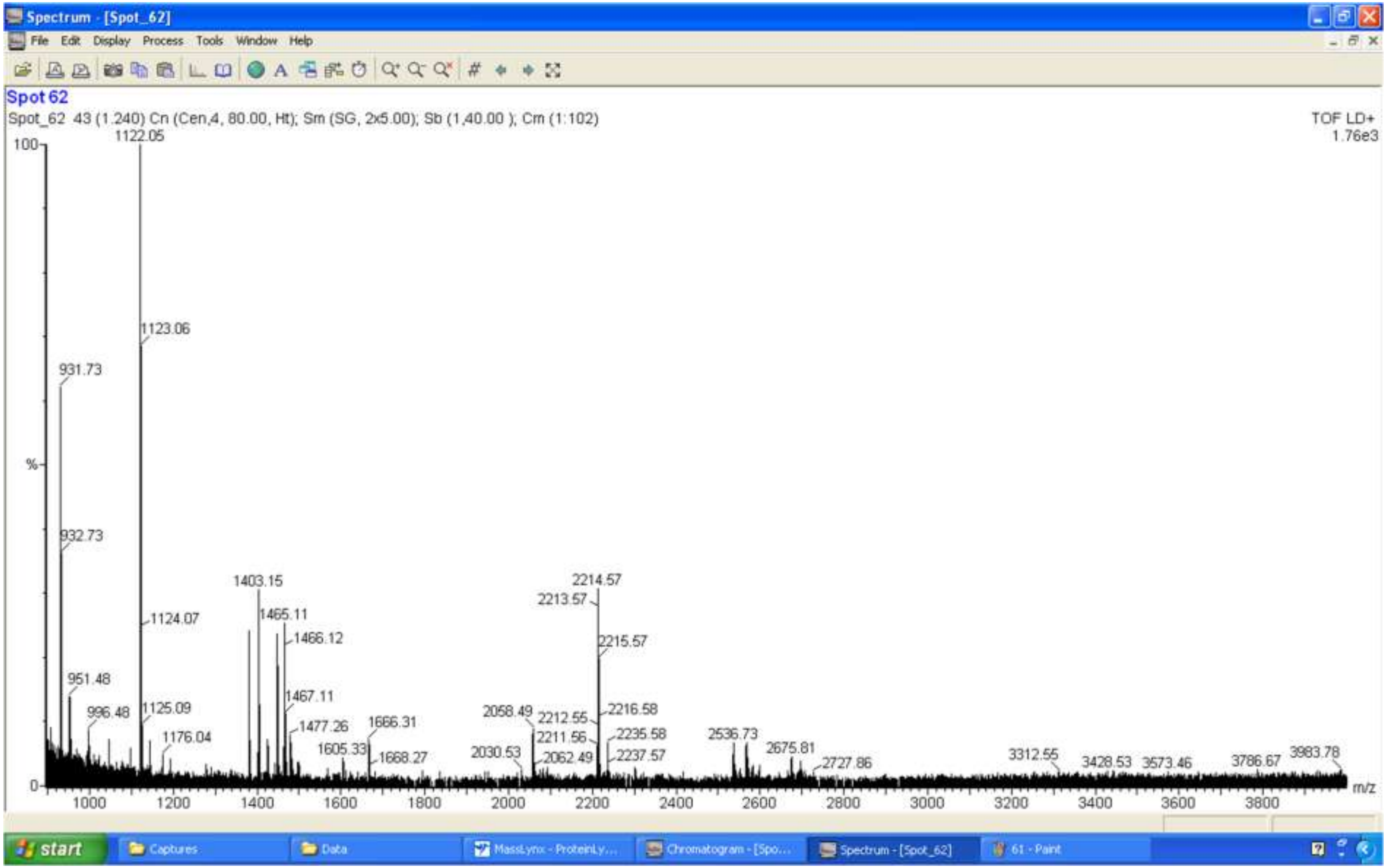

Spectrum - [Spot_62]
File Edit Display Process Tools Window Help
Spot 62
Spot_62 43 (1.240) Cn (Cen,4, 80.00, Ht); Sm (SG, 2x5.00); Sb (1,40.00); Cm (1:102)
TOF LD+
1.76e3
1122.05
1123.06
931.73
932.73
1403.15
1465.11
1466.12
1124.07
2214.57
2213.57
2215.57
951.48
996.48
1125.09
1176.04
1467.11
1477.26
1605.33
1666.31
1668.27
2058.49
2030.53
2212.55
2211.56
2062.49
2216.58
2235.58
2237.57
2536.73
2675.81
2727.86
3312.55
3428.53
3573.46
3786.67
3983.78
100
%
0
m/z
1000 1200 1400 1600 1800 2000 2200 2400 2600 2800 3000 3200 3400 3600 3800
start
Captures
Data
MassLynx - ProteinLy...
Chromatogram - [Spo...
Spectrum - [Spot_62]
61 - Paint

Figure S1.047

Mascot Search Results Spot 63

```
User            : Paul Millares
Email           : paul.millares@gmail.com
Search title    : Spot 63
Database        : Haemonchus 210108 (6387 sequences; 918038 residues)
Timestamp       : 1 Aug 2011 at 10:53:36 GMT
Top Score       : 39 for HCP03240_1, putative nuclear encoded protein  Method: similarity and extension
```

Mascot Score Histogram

Protein score is -10*Log(P), where P is the probability that the observed match is a random event.

Protein scores greater than 51 are significant (p<0.05).

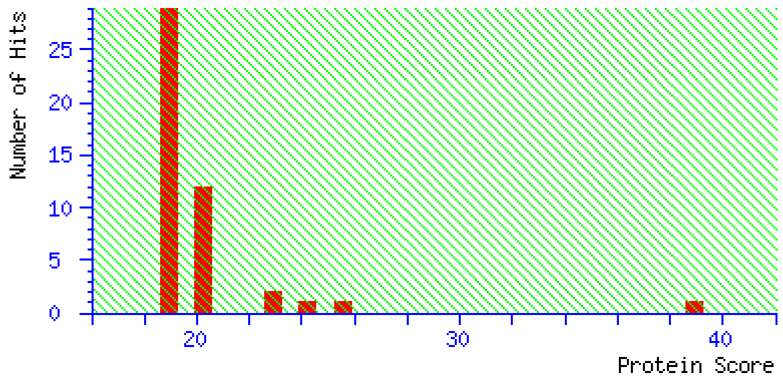

Concise Protein Summary Report

1. HCP03240_1 **Mass:** 23453 **Score:** 39 **Expect:** 0.82 **Matches:** 15

   putative nuclear encoded protein Method: similarity and extension

   HCP03240_3 **Mass:** 23007 **Score:** 31 **Expect:** 5.1 **Matches:** 13

   putative nuclear encoded protein Method: similarity and extension

Search Parameters

Type of search : Peptide Mass Fingerprint
Enzyme : Trypsin
Variable modifications : Carbamidomethyl (C),Glu->pyro-Glu (N-term E),Oxidation (M)
Mass values : Monoisotopic
Protein Mass : Unrestricted
Peptide Mass Tolerance : ± 1.2 Da
Peptide Charge State : 1+
Max Missed Cleavages : 1
Number of queries : 119

Protein View

Match to: **HCP03240_1** Score: **39** Expect: **0.82**
putative nuclear encoded protein Method: similarity and extension

Nominal mass (M_r): **23453**; Calculated pI value: **8.99**
NCBI BLAST search of HCP03240_1 against nr
Unformatted sequence string for pasting into other applications

Variable modifications: Carbamidomethyl (C),Glu->pyro-Glu (N-term E),Oxidation (M)
Cleavage by Trypsin: cuts C-term side of KR unless next residue is P
Number of mass values searched: **119**
Number of mass values matched: **15**
Sequence Coverage: **78%**

Matched peptides shown in **Bold Red**

```
  1 LAVRLAQGLT RGLVRPLAAL PTVQRSFASM AAEAFKKHEV VPDVLATAPT
 51 KVVKAHYDSG AEVNLGNVLT PTQVKNPPKL TWDAEPGALY TVIFTDPDAP
101 SRKEATFREW HHWLVVNVPG NDINKGDVLA EYIGSGPPKD TGLHRYVFLV
151 YKQPSGRITD SEHGHLTNRS GDGRGGFKTE KFVAKHKLGT PIAGNFYQAE
201 WDDYVPILYK QLGA
```

Start - End	Observed	Mr(expt)	Mr(calc)	Delta	Miss	Sequence
1 - 11	1196.80	1195.79	1196.74	-0.94	1	-.LAVRLAQGLTR.G
5 - 25	2229.49	2228.49	2229.34	-0.86	1	R.LAQGLTRGLVRPLAALPTVQR.S
26 - 36	1159.77	1158.77	1158.54	0.23	0	R.SFASMAAEAFK.K
37 - 51	1605.30	1604.29	1603.89	0.40	1	K.KHEVVPDVLATAPTK.V
38 - 51	1477.21	1476.20	1475.80	0.40	0	K.HEVVPDVLATAPTK.V
55 - 75	2213.53	2212.52	2212.11	0.41	0	K.AHYDSGAEVNLGNVLTPTQVK.N
80 - 102	2535.69	2534.68	2534.23	0.45	0	K.LTWDAEPGALYTVIFTDPDAPSR.K
80 - 103	2663.71	2662.70	2662.33	0.37	1	K.LTWDAEPGALYTVIFTDPDAPSRK.E

109 - 125	2057.43	2056.42	2056.03	0.39	0	R.EWHHWLVVNVPGNDINK.G
109 - 139	3441.23	3440.22	3439.73	0.49	1	R.EWHHWLVVNVPGNDINKGDVLAEYIGSGPPK.D
126 - 139	1402.04	1401.03	1401.71	-0.68	0	K.GDVLAEYIGSGPPK.D
126 - 139	1403.12	1402.11	1401.71	0.40	0	K.GDVLAEYIGSGPPK.D
146 - 152	931.71	930.70	930.52	0.18	0	R.YVFLVYK.Q
158 - 169	1380.06	1379.05	1378.66	0.39	0	R.ITDSEHGHLTNR.S
188 - 210	2673.74	2672.73	2672.32	0.42	0	K.LGTPIAGNFYQAEWDDYVPILYK.Q

No match to: 897.45, 900.45, 906.68, 907.44, 913.47, 920.45, 922.46, 925.48, 927.48, 938.46, 941.58, 948.49, 951.45, 958.47, 963.55, 970.50, 977.46, 984.47, 985.50, 992.48, 994.49, 997.48, 1004.48, 1006.46, 1010.50, 1018.51, 1022.49, 1029.47, 1030.51, 1036.48, 1037.49, 1043.50, 1045.66, 1051.89, 1052.48, 1059.48, 1066.50, 1069.71, 1075.52, 1078.34, 1084.51, 1091.52, 1098.56, 1101.59, 1107.85, 1114.63, 1120.72, 1123.66, 1132.72, 1135.69, 1140.76, 1142.76, 1149.81, 1155.76, 1166.18, 1179.01, 1200.81, 1218.03, 1245.79, 1254.84, 1259.81, 1262.81, 1271.90, 1278.82, 1289.56, 1315.04, 1326.07, 1337.10, 1339.87, 1346.07, 1363.81, 1394.13, 1424.09, 1425.11, 1441.11, 1455.11, 1499.17, 1591.26, 1595.19, 1618.86, 1627.30, 1666.27, 1687.13, 1840.25, 2008.47, 2030.41, 2067.40, 2073.43, 2083.39, 2089.43, 2121.46, 2153.10, 2211.53, 2235.51, 2252.91, 2565.68, 2581.72, 2597.62, 2691.74, 2694.78, 2705.73, 2725.72, 3004.41, 3072.62

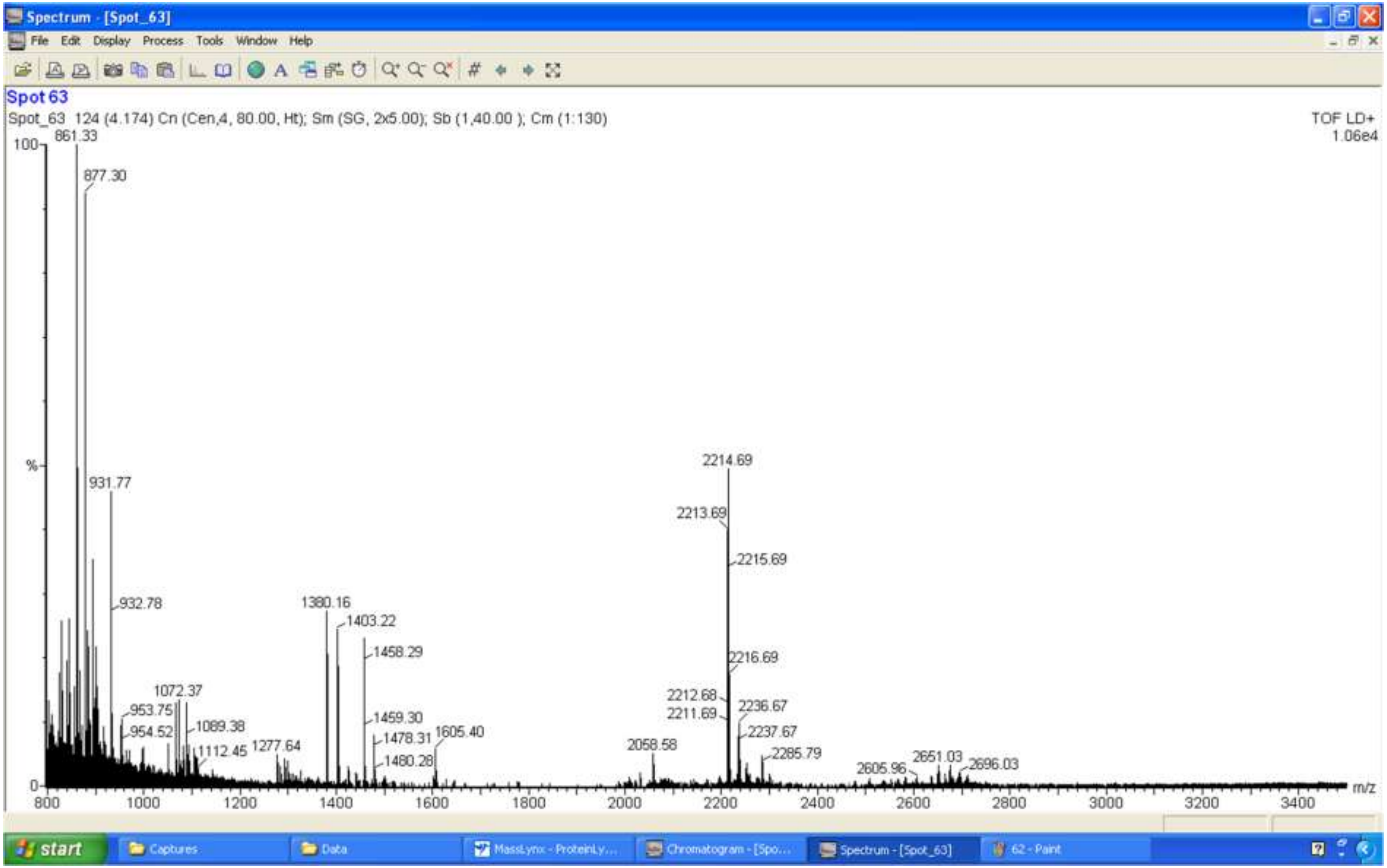
Spectrum - [Spot_63]
File Edit Display Process Tools Window Help
Spot 63
Spot_63 124 (4.174) Cn (Cen,4, 80.00, Ht); Sm (SG, 2x5.00); Sb (1,40.00); Cm (1:130)
TOF LD+
1.06e4
100
%
0
861.33
877.30
931.77
932.78
953.75
954.52
1072.37
1089.38
1112.45
1277.64
1380.16
1403.22
1458.29
1459.30
1478.31
1480.28
1605.40
2058.58
2211.69
2212.68
2213.69
2214.69
2215.69
2216.69
2236.67
2237.67
2285.79
2605.96
2651.03
2696.03
800
1000
1200
1400
1600
1800
2000
2200
2400
2600
2800
3000
3200
3400
m/z
start
Captures
Data
MassLynx - ProteinLy...
Chromatogram - [Spo...
Spectrum - [Spot_63]
62 - Paint

Figure S1.48

Mascot Search Results Spot 70

```
User            : Paul Millares
Email           : paul.millares@gmail.com
Search title    : Spot 70
Database        : Haemonchus 210108 (6387 sequences; 918038 residues)
Timestamp       : 1 Aug 2011 at 10:54:18 GMT
Top Score       : 66 for HCP02240_2, putative nuclear encoded protein  Method: similarity and extension
```

Mascot Score Histogram

Protein score is -10*Log(P), where P is the probability that the observed match is a random event.

Protein scores greater than 51 are significant (p<0.05).

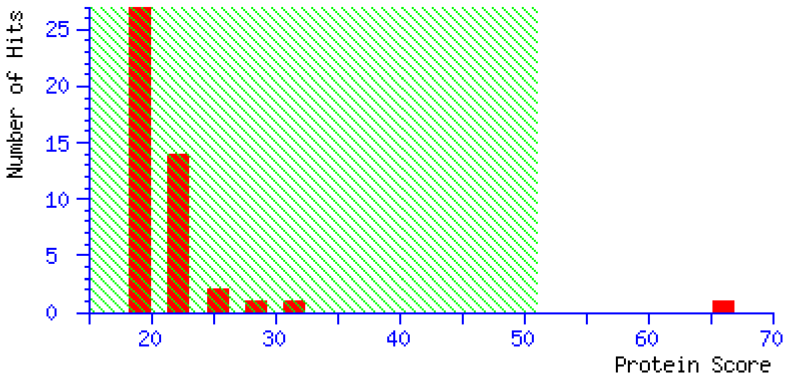

Concise Protein Summary Report

1. HCP02240_2 **Mass:** 14822 **Score:** **66** **Expect:** 0.0016 **Matches:** 9
putative nuclear encoded protein Method: similarity and extension
HCP02240_1 **Mass:** 14822 **Score:** **66** **Expect:** 0.0016 **Matches:** 9
putative nuclear encoded protein Method: similarity and extension

HCP02240_3 **Mass:** 15682 **Score:** 60 **Expect:** 0.0061 **Matches:** 9

putative nuclear encoded protein Method: similarity and extension

---

2. HCP00222_1 **Mass:** 26744 **Score:** 31 **Expect:** 5.7 **Matches:** 10

putative nuclear encoded protein Method: ESTScan

Search Parameters

Type of search : Peptide Mass Fingerprint
Enzyme : Trypsin
Variable modifications : Carbamidomethyl (C),Glu->pyro-Glu (N-term E),Oxidation (M)
Mass values : Monoisotopic
Protein Mass : Unrestricted
Peptide Mass Tolerance : ± 1.2 Da
Peptide Charge State : 1+
Max Missed Cleavages : 1
Number of queries : 30

Protein View

Match to: **HCP02240_2** Score: **66** Expect: **0.0016**
putative nuclear encoded protein Method: similarity and extension

Nominal mass (M_r): **14822**; Calculated pI value: **5.40**
NCBI BLAST search of HCP02240_2 against nr
Unformatted sequence string for pasting into other applications

Variable modifications: Carbamidomethyl (C),Glu->pyro-Glu (N-term E),Oxidation (M)
Cleavage by Trypsin: cuts C-term side of KR unless next residue is P
Number of mass values searched: **30**
Number of mass values matched: **9**
Sequence Coverage: **72%**

Matched peptides shown in **Bold Red**

1 MKYLILIALF GCCLASR**LQS VAITGK**LLCG SKPAAGVKVK **LWEEDSGPDP**

51 DDLLDEGKTS SAGTFTLKGS DNEITTIDPV FKIYHDCDDG IKPGQRKVKF
101 RIPSSYISTG STPKRTFDIG VLNLETIFPG EERDFI

Start - End	Observed	Mr(expt)	Mr(calc)	Delta	Miss	Sequence
18 - 26	915.43	914.42	915.54	-1.12	0	R.LQSVAITGK.L
41 - 58	2030.13	2029.13	2028.88	0.25	0	K.LWEEDSGPDPDDLLDEGK.T
59 - 68	1012.65	1011.64	1011.52	0.12	0	K.TSSAGTFTLK.G
69 - 82	1536.05	1535.04	1534.75	0.29	0	K.GSDNEITTIDPVFK.I
83 - 96	1674.05	1673.05	1672.76	0.28	0	K.IYHDCDDGIKPGQR.K Carbamidomethyl (C)
100 - 114	1641.15	1640.15	1639.86	0.29	1	K.FRIPSSYISTGSTPK.R
102 - 114	1338.01	1337.01	1336.69	0.32	0	R.IPSSYISTGSTPK.R
115 - 133	2206.38	2205.37	2205.14	0.23	1	K.RTFDIGVLNLETIFPGEER.D
116 - 133	2050.31	2049.30	2049.04	0.26	0	R.TFDIGVLNLETIFPGEER.D

No match to: 901.39, 908.42, 921.63, 929.40, 938.64, 949.59, 951.41, 960.56, 994.44, 996.41, 998.66, 1034.63, 1045.67, 1067.65, 1179.97, 1278.05, 1304.98, 1359.99, 1430.14, 2103.30, 2211.34

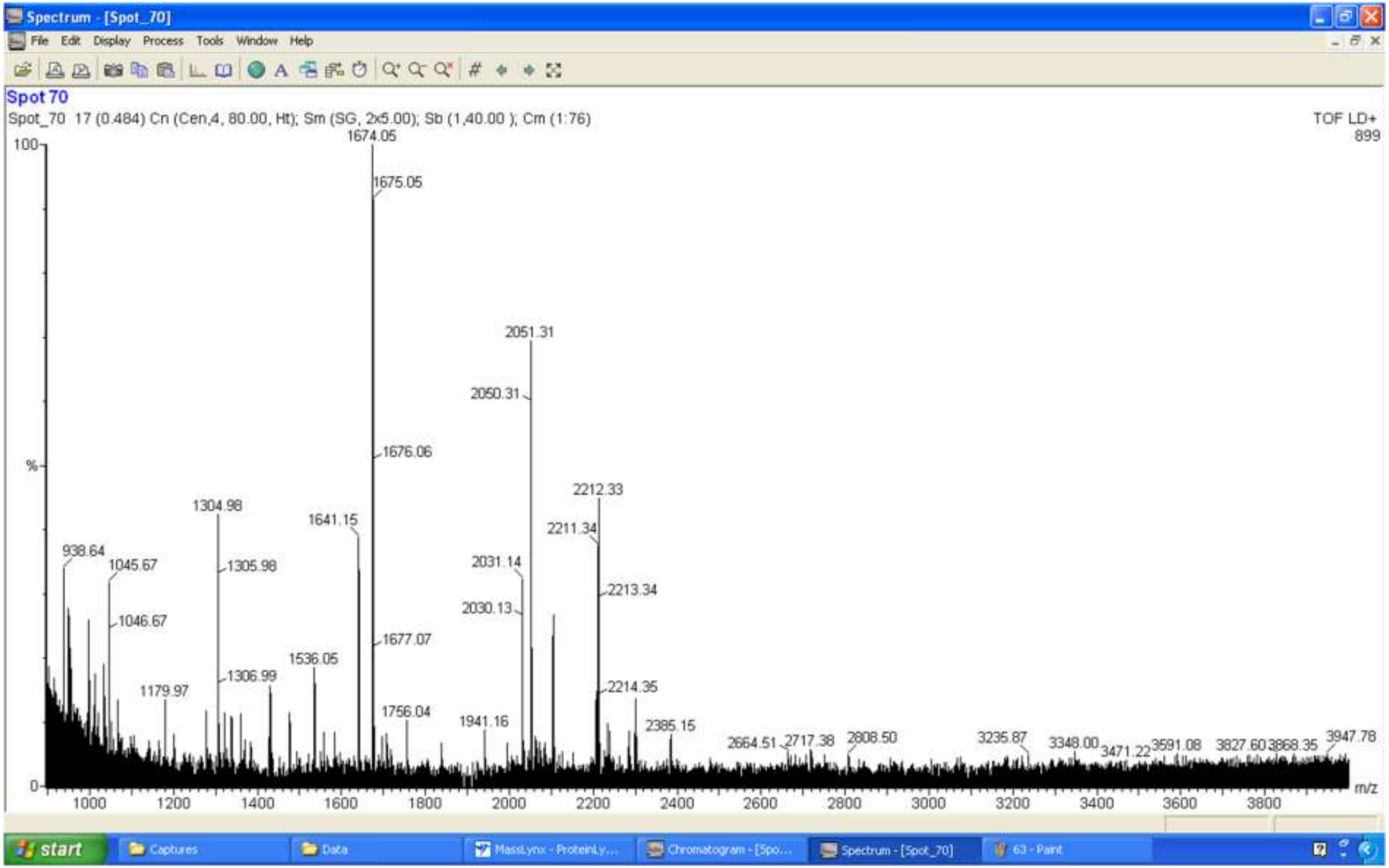

Spectrum - [Spot_70]
File Edit Display Process Tools Window Help
Spot 70
Spot_70 17 (0.484) Cn (Cen,4, 80.00, Ht); Sm (SG, 2x5.00); Sb (1,40.00); Cm (1:76)
TOF LD+
899
100
%
0
1674.05
1675.05
1676.06
1677.07
2051.31
2050.31
2212.33
2211.34
2213.34
2214.35
1304.98
1305.98
1306.99
1641.15
938.64
1045.67
1046.67
2031.14
2030.13
1536.05
1179.97
1756.04
1941.16
2385.15
2664.51
2717.38
2808.50
3235.87
3348.00
3471.22
3591.08
3827.60
3868.35
3947.78
m/z
1000
1200
1400
1600
1800
2000
2200
2400
2600
2800
3000
3200
3400
3600
3800
start
Captures
Data
MassLynx - ProteinLy...
Chromatogram - [Spo...
Spectrum - [Spot_70]
63 - Paint

Figure S1.49

Mascot Search Results Spot 73

```
User          : Paul Millares
Email         : paul.millares@gmail.com
Search title  : Spot 73
Database      : Haemonchus 210108 (6387 sequences; 918038 residues)
Timestamp     : 1 Aug 2011 at 10:56:09 GMT
Top Score     : 85 for HCP02740_1, putative nuclear encoded protein  Method: similarity and extension
```

Mascot Score Histogram

Protein score is -10*Log(P), where P is the probability that the observed match is a random event.

Protein scores greater than 51 are significant (p<0.05).

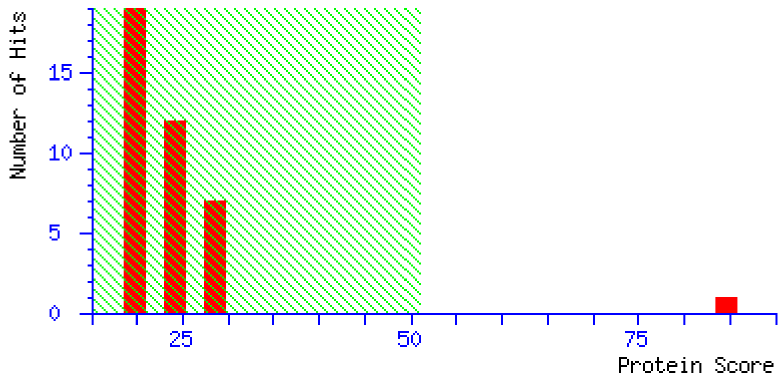

Concise Protein Summary Report

1. HCP02740_1 **Mass:** 15137 **Score:** 85 **Expect:** 2.2e-005 **Matches:** 11
   putative nuclear encoded protein Method: similarity and extension
   HCP02740_2 **Mass:** 15137 **Score:** 85 **Expect:** 2.2e-005 **Matches:** 11
   putative nuclear encoded protein Method: similarity and extension
   HCP00729_1 **Mass:** 11098 **Score:** 41 **Expect:** 0.51 **Matches:** 6
   putative nuclear encoded protein Method: similarity and extension

HCP02013_1 **Mass:** 9929 **Score:** 34 **Expect:** 2.5 **Matches:** 5

putative nuclear encoded protein Method: similarity and extension

---

2. HCP04090_2 **Mass:** 18988 **Score:** 31 **Expect:** 5.6 **Matches:** 6

putative nuclear encoded protein Method: similarity and extension

---

Search Parameters

Type of search : Peptide Mass Fingerprint
Enzyme : Trypsin
Variable modifications : Carbamidomethyl (C),Glu->pyro-Glu (N-term E),Oxidation (M)
Mass values : Monoisotopic
Protein Mass : Unrestricted
Peptide Mass Tolerance : ± 1.2 Da
Peptide Charge State : 1+
Max Missed Cleavages : 1
Number of queries : 27

Protein View

Match to: **HCP02740_1** Score: **85** Expect: **2.2e-005**
putative nuclear encoded protein Method: similarity and extension

Nominal mass (M_r): **15137**; Calculated pI value: **6.15**
NCBI BLAST search of HCP02740_1 against nr
Unformatted sequence string for pasting into other applications

Variable modifications: Carbamidomethyl (C),Glu->pyro-Glu (N-term E),Oxidation (M)
Cleavage by Trypsin: cuts C-term side of KR unless next residue is P
Number of mass values searched: **27**
Number of mass values matched: **11**
Sequence Coverage: **77%**

Matched peptides shown in **Bold Red**

```
  1 TGILGKWTFV SSENFEAYMK EAGVGLMTRK VAANLKPTLE FVKEGDHIQM
 51 TSVSTFKTYV TKFKIGETFD EKTGDGRDVS QTYTVENDHL VLIEKSKSGG
101 PDSRIERYVE GDKLYIICEC NGVKSTRIYE RVKE
```

Start - End Observed Mr(expt) Mr(calc) Delta Miss Sequence

7 - 20	1754.97	1753.96	1753.77	0.19	0	K.WTFVSSENFEAYMK.E Oxidation (M)
21 - 29	949.53	948.53	948.47	0.06	0	K.EAGVGLMTR.K Oxidation (M)
21 - 30	1042.56	1041.55	1042.56	-1.00	1	K.EAGVGLMTRK.V Glu->pyro-Glu (N-term E)
30 - 43	1558.14	1557.13	1556.93	0.20	1	R.KVAANLKPTLEFVK.E
31 - 43	1430.06	1429.05	1428.83	0.22	0	K.VAANLKPTLEFVK.E
44 - 57	1595.94	1594.93	1594.73	0.20	0	K.EGDHIQMTSVSTFK.T Oxidation (M)
65 - 72	938.50	937.49	937.44	0.05	0	K.IGETFDEK.T
78 - 95	2103.18	2102.18	2102.05	0.12	0	R.DVSQTYTVENDHLVLIEK.S
98 - 107	1074.68	1073.67	1072.53	1.15	1	K.SGGPDSRIER.Y
108 - 124	1945.06	1944.05	1944.93	-0.88	1	R.YVEGDKLYIICECNGVK.S
114 - 124	1253.87	1252.86	1253.61	-0.76	0	K.LYIICECNGVK.S

No match to: 900.33, 907.34, 960.48, 971.51, 982.44, 993.47, 998.60, 1010.60, 1020.59, 1116.67, 1138.74, 1252.88, 1275.84, 1452.05, 1733.02, 2125.16

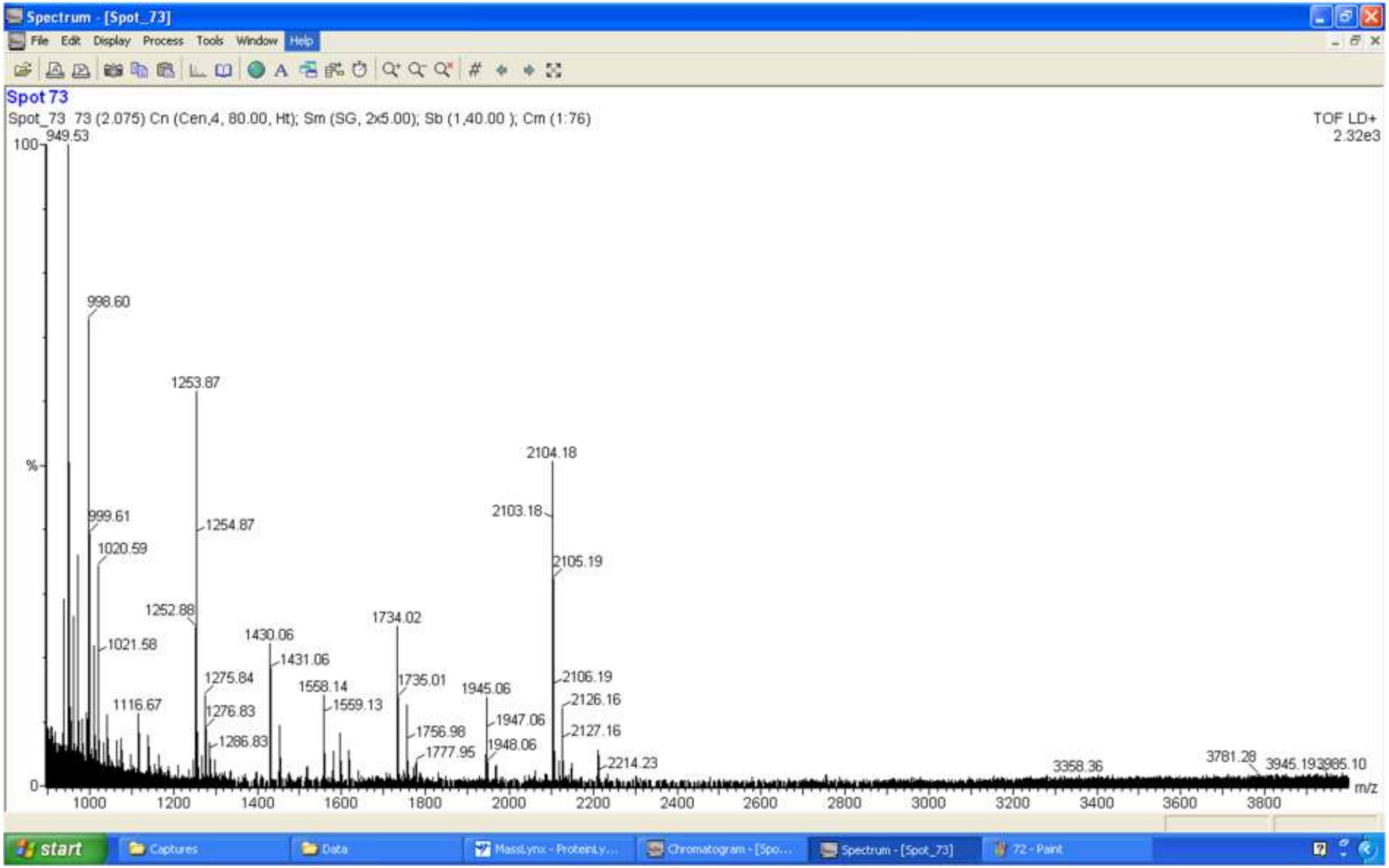
Spectrum - [Spot_73]
File Edit Display Process Tools Window Help
Spot 73
Spot_73 73 (2.075) Cn (Cen,4, 80.00, Ht); Sm (SG, 2x5.00); Sb (1,40.00); Cm (1:76)
TOF LD+
2.32e3
100
%
0
949.53
998.60
999.61
1020.59
1021.58
1116.67
1253.87
1254.87
1252.88
1275.84
1276.83
1286.83
1430.06
1431.06
1558.14
1559.13
1734.02
1735.01
1756.98
1777.95
1945.06
1947.06
1948.06
2104.18
2103.18
2105.19
2106.19
2126.16
2127.16
2214.23
3358.36
3781.28
3945.193985.10
m/z
1000 1200 1400 1600 1800 2000 2200 2400 2600 2800 3000 3200 3400 3600 3800
start
Captures
Data
MassLynx - ProteinLy...
Chromatogram - [Spo...
Spectrum - [Spot_73]
72 - Paint

Figure S1.50

Mascot Search Results Sport 77

```
User            : Paul Millares
Email           : paul.millares@gmail.com
Search title    : Spot 77
Database        : Haemonchus 210108 (6387 sequences; 918038 residues)
Timestamp       : 1 Aug 2011 at 10:58:10 GMT
Top Score       : 71 for HCP07574_1, putative nuclear encoded protein  Method: ESTScan
```

Mascot Score Histogram

Protein score is -10*Log(P), where P is the probability that the observed match is a random event.

Protein scores greater than 51 are significant (p<0.05).

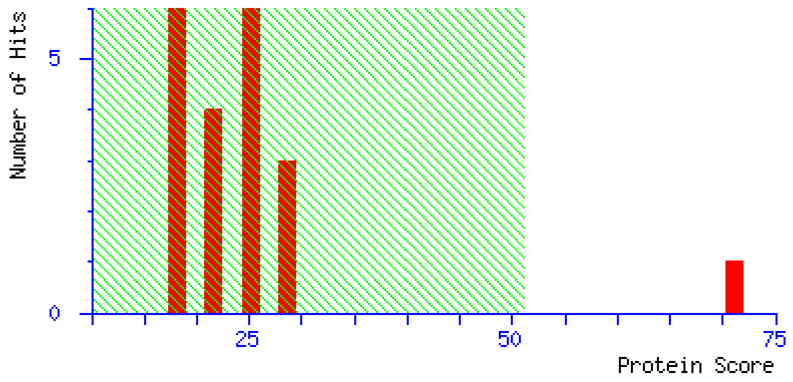

Concise Protein Summary Report

1. HCP07574_1 **Mass:** 14204 **Score:** 71 **Expect:** 0.00051 **Matches:** 6
   putative nuclear encoded protein Method: ESTScan
   HCP05510_1 **Mass:** 9520 **Score:** 31 **Expect:** 5.7 **Matches:** 3
   putative nuclear encoded protein Method: Longest ORF
   HCP00552_1 **Mass:** 8160 **Score:** 30 **Expect:** 6 **Matches:** 3
   putative nuclear encoded protein Method: Longest ORF

HCP00251_2 **Mass:** 16861 **Score:** 24 **Expect:** 24 **Matches:** 3
putative nuclear encoded protein Method: similarity and extension
HCP02831_1 **Mass:** 15391 **Score:** 24 **Expect:** 25 **Matches:** 3
putative nuclear encoded protein Method: similarity and extension
HCP03187_1 **Mass:** 7639 **Score:** 23 **Expect:** 31 **Matches:** 2
putative nuclear encoded protein Method: ESTScan
HCP03187_2 **Mass:** 7639 **Score:** 23 **Expect:** 31 **Matches:** 2
putative nuclear encoded protein Method: ESTScan
HCP00076_1 **Mass:** 5973 **Score:** 22 **Expect:** 42 **Matches:** 2
putative nuclear encoded protein Method: Longest ORF
HCP00778_1 **Mass:** 6518 **Score:** 21 **Expect:** 46 **Matches:** 2
putative nuclear encoded protein Method: Longest ORF
HCP00251_1 **Mass:** 24230 **Score:** 20 **Expect:** 68 **Matches:** 3
putative nuclear encoded protein Method: similarity and extension
HCP04661_1 **Mass:** 6367 **Score:** 20 **Expect:** 70 **Matches:** 2
putative nuclear encoded protein Method: similarity and extension
HCP02127_1 **Mass:** 6808 **Score:** 19 **Expect:** 73 **Matches:** 2
putative nuclear encoded protein Method: similarity and extension
HCP04192_1 **Mass:** 8925 **Score:** 19 **Expect:** 82 **Matches:** 2
putative nuclear encoded protein Method: Longest ORF
HCP00885_1 **Mass:** 9550 **Score:** 19 **Expect:** 84 **Matches:** 2
putative nuclear encoded protein Method: ESTScan

---

2. HCP12390_1 **Mass:** 23266 **Score:** 30 **Expect:** 6.7 **Matches:** 4
putative nuclear encoded protein Method: similarity and extension
HCP06691_1 **Mass:** 7534 **Score:** 19 **Expect:** 77 **Matches:** 2
putative nuclear encoded protein Method: Longest ORF
HCP06691_2 **Mass:** 7534 **Score:** 19 **Expect:** 77 **Matches:** 2
putative nuclear encoded protein Method: Longest ORF
HCP09722_1 **Mass:** 21896 **Score:** 18 **Expect:** 92 **Matches:** 3

putative nuclear encoded protein Method: ESTScan

Search Parameters

Type of search : Peptide Mass Fingerprint
Enzyme : Trypsin
Variable modifications : Carbamidomethyl (C),Glu->pyro-Glu (N-term E),Oxidation (M)
Mass values : Monoisotopic
Protein Mass : Unrestricted
Peptide Mass Tolerance : ± 1.2 Da
Peptide Charge State : 1+
Max Missed Cleavages : 1
Number of queries : 11

Protein View

Match to: **HCP07574_1** Score: **71** Expect: **0.00051**
putative nuclear encoded protein Method: ESTScan

Nominal mass (M_r): **14204**; Calculated pI value: **6.13**
NCBI BLAST search of HCP07574_1 against nr
Unformatted sequence string for pasting into other applications

Variable modifications: Carbamidomethyl (C),Glu->pyro-Glu (N-term E),Oxidation (M)
Cleavage by Trypsin: cuts C-term side of KR unless next residue is P
Number of mass values searched: **11**
Number of mass values matched: **6**
Sequence Coverage: **58%**

Matched peptides shown in **Bold Red**

```
  1 WTQAILLIFV VGSAFGSIAI QQAKLGERVE LNLGSGVVTW KRVTSNGDEY
 51 IKYCGPTEKG PRCAQFVKED NTPVKPQSSA HVEPNGVLVI DSFKKSDEGL
101 YSSPDLKPRE TNHPDGSVSA VAAPTIQLML AN
```

Start - End	Observed	Mr(expt)	Mr(calc)	Delta	Miss	Sequence
29 - 41	1402.04	1401.03	1400.77	0.26	0	R.VELNLGSGVVTWK.R
69 - 94	2808.58	2807.58	2806.41	1.16	0	K.EDNTPVKPQSSAHVEPNGVLVIDSFK.K
69 - 95	2936.69	2935.68	2934.51	1.17	1	K.EDNTPVKPQSSAHVEPNGVLVIDSFKK.S
95 - 109	1692.09	1691.08	1690.85	0.23	1	K.KSDEGLYSSPDLKPR.E
96 - 109	1564.01	1563.01	1562.76	0.25	0	K.SDEGLYSSPDLKPR.E
110 - 132	2352.32	2351.31	2351.14	0.17	0	R.ETNHPDGSVSAVAAPTIQLMLAN.- Oxidation (M)

No match to: 951.37, 997.37, 1417.03, 1434.02, 1585.99

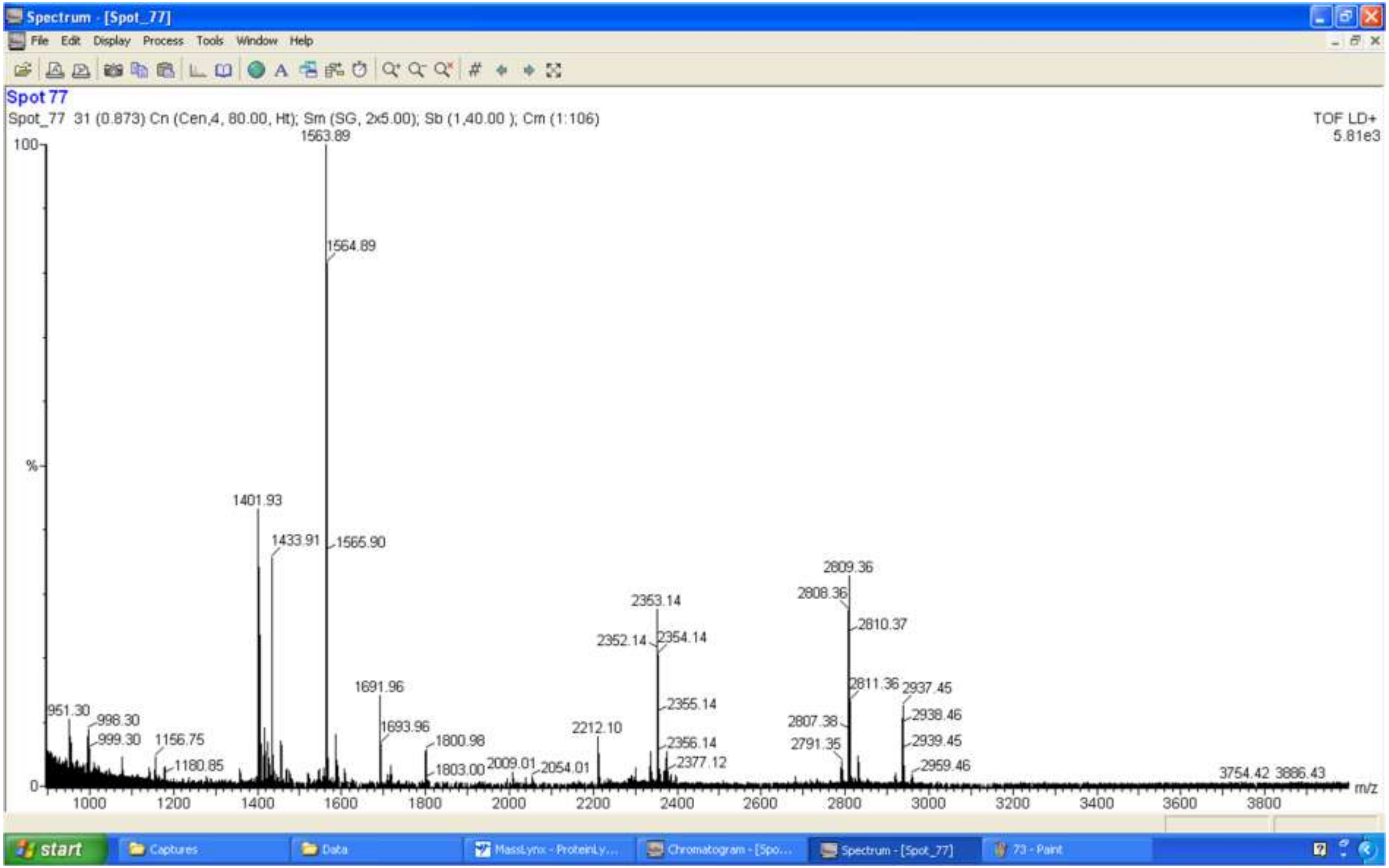

Figure S1.51

Mascot Search Results Spot 78

```
User            : Paul Millares
Email           : paul.millares@gmail.com
Search title    : Spot 78
Database        : Haemonchus 210108 (6387 sequences; 918038 residues)
Timestamp       : 1 Aug 2011 at 10:59:20 GMT
Top Score       : 49 for HCP00413_1, putative nuclear encoded protein  Method: similarity and extension
```

Mascot Score Histogram

Protein score is -10*Log(P), where P is the probability that the observed match is a random event.

Protein scores greater than 51 are significant (p<0.05).

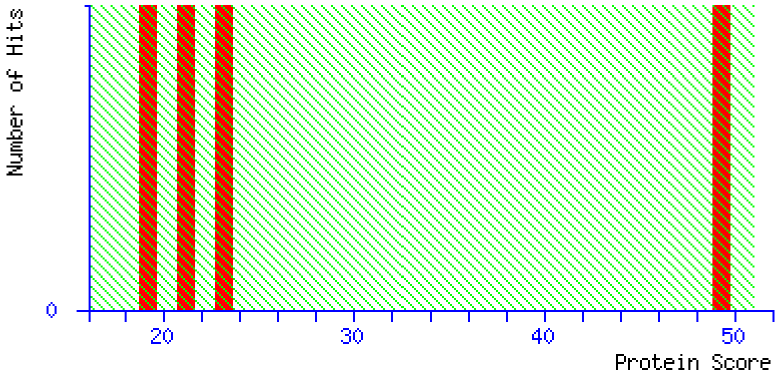

Concise Protein Summary Report

1. HCP00413_1 **Mass:** 16125 **Score:** 49 **Expect:** 0.075 **Matches:** 4
   putative nuclear encoded protein Method: similarity and extension
   HCP02707_1 **Mass:** 24382 **Score:** 38 **Expect:** 0.94 **Matches:** 4
   putative nuclear encoded protein Method: similarity and extension
   HCP01113_1 **Mass:** 20400 **Score:** 29 **Expect:** 8.8 **Matches:** 3
   putative nuclear encoded protein Method: Longest ORF

HCP02707_2 **Mass:** 23772 **Score:** 25 **Expect:** 20 **Matches:** 3
putative nuclear encoded protein Method: similarity and extension
HCP02477_1 **Mass:** 13254 **Score:** 21 **Expect:** 52 **Matches:** 2
putative nuclear encoded protein Method: similarity and extension
HCP00641_2 **Mass:** 12440 **Score:** 21 **Expect:** 57 **Matches:** 2
putative nuclear encoded protein Method: similarity and extension
HCP00641_1 **Mass:** 12470 **Score:** 21 **Expect:** 57 **Matches:** 2
putative nuclear encoded protein Method: similarity and extension
HCP00641_3 **Mass:** 12440 **Score:** 21 **Expect:** 57 **Matches:** 2
putative nuclear encoded protein Method: similarity and extension
HCP02814_1 **Mass:** 12389 **Score:** 20 **Expect:** 61 **Matches:** 2
putative nuclear encoded protein Method: similarity and extension
HCP09515_2 **Mass:** 14649 **Score:** 20 **Expect:** 67 **Matches:** 2
putative nuclear encoded protein Method: similarity and extension
HCP01267_2 **Mass:** 14472 **Score:** 20 **Expect:** 67 **Matches:** 2
putative nuclear encoded protein Method: ESTScan
HCP05975_1 **Mass:** 14129 **Score:** 20 **Expect:** 67 **Matches:** 2
putative nuclear encoded protein Method: similarity and extension
HCP10832_1 **Mass:** 14856 **Score:** 20 **Expect:** 68 **Matches:** 2
putative nuclear encoded protein Method: similarity and extension
HCP09515_1 **Mass:** 14636 **Score:** 20 **Expect:** 68 **Matches:** 2
putative nuclear encoded protein Method: similarity and extension
HCP00690_1 **Mass:** 14918 **Score:** 20 **Expect:** 70 **Matches:** 2
putative nuclear encoded protein Method: similarity and extension
HCP00690_3 **Mass:** 15276 **Score:** 19 **Expect:** 73 **Matches:** 2
putative nuclear encoded protein Method: similarity and extension
HCP01834_4 **Mass:** 15244 **Score:** 19 **Expect:** 79 **Matches:** 2
putative nuclear encoded protein Method: similarity and extension
HCP03479_1 **Mass:** 13734 **Score:** 19 **Expect:** 84 **Matches:** 2
putative nuclear encoded protein Method: similarity and extension

HCP03084_1 **Mass:** 16241 **Score:** 19 **Expect:** 84 **Matches:** 2
putative nuclear encoded protein Method: similarity and extension
HCP13296_1 **Mass:** 16325 **Score:** 18 **Expect:** 92 **Matches:** 2
putative nuclear encoded protein Method: Longest ORF
HCP06536_1 **Mass:** 13515 **Score:** 18 **Expect:** 92 **Matches:** 2
putative nuclear encoded protein Method: ESTScan
HCP06525_2 **Mass:** 17689 **Score:** 18 **Expect:** 97 **Matches:** 2
putative nuclear encoded protein Method: ESTScan
HCP03389_1 **Mass:** 18395 **Score:** 18 **Expect:** 97 **Matches:** 2
putative nuclear encoded protein Method: similarity and extension
HCP00439_1 **Mass:** 16866 **Score:** 18 **Expect:** 1e+002 **Matches:** 2
putative nuclear encoded protein Method: similarity and extension
HCP06525_1 **Mass:** 18330 **Score:** 18 **Expect:** 1e+002 **Matches:** 2
putative nuclear encoded protein Method: ESTScan
HCP03084_3 **Mass:** 18398 **Score:** 18 **Expect:** 1e+002 **Matches:** 2
putative nuclear encoded protein Method: similarity and extension
HCP03572_1 **Mass:** 18522 **Score:** 18 **Expect:** 1.1e+002 **Matches:** 2
putative nuclear encoded protein Method: similarity and extension
HCP02193_2 **Mass:** 18532 **Score:** 18 **Expect:** 1.1e+002 **Matches:** 2
putative nuclear encoded protein Method: similarity and extension
HCP06833_1 **Mass:** 20514 **Score:** 17 **Expect:** 1.2e+002 **Matches:** 2
putative nuclear encoded protein Method: ESTScan
HCP02040_1 **Mass:** 19341 **Score:** 17 **Expect:** 1.2e+002 **Matches:** 2
putative nuclear encoded protein Method: ESTScan
HCP02143_1 **Mass:** 7324 **Score:** 17 **Expect:** 1.3e+002 **Matches:** 2
putative nuclear encoded protein Method: similarity and extension
HCP05837_1 **Mass:** 19609 **Score:** 17 **Expect:** 1.3e+002 **Matches:** 2
putative nuclear encoded protein Method: similarity and extension
HCP00555_1 **Mass:** 18999 **Score:** 17 **Expect:** 1.3e+002 **Matches:** 2
putative nuclear encoded protein Method: similarity and extension

HCP07084_1 **Mass:** 21277 **Score:** 17 **Expect:** 1.3e+002 **Matches:** 2

putative nuclear encoded protein Method: similarity and extension

HCP00998_2 **Mass:** 67189 **Score:** 17 **Expect:** 1.3e+002 **Matches:** 3

putative nuclear encoded protein Method: similarity and extension

---

Search Parameters

Type of search : Peptide Mass Fingerprint
Enzyme : Trypsin
Variable modifications : Carbamidomethyl (C),Glu->pyro-Glu (N-term E),Oxidation (M)
Mass values : Monoisotopic
Protein Mass : Unrestricted
Peptide Mass Tolerance : ± 1.2 Da
Peptide Charge State : 1+
Max Missed Cleavages : 1
Number of queries : 5

Protein View

Match to: **HCP00413_1** Score: **49** Expect: **0.075**
putative nuclear encoded protein Method: similarity and extension

Nominal mass (M_r): **16125**; Calculated pI value: **5.17**
NCBI BLAST search of HCP00413_1 against nr
Unformatted sequence string for pasting into other applications

Variable modifications: Carbamidomethyl (C),Glu->pyro-Glu (N-term E),Oxidation (M)
Cleavage by Trypsin: cuts C-term side of KR unless next residue is P
Number of mass values searched: **5**
Number of mass values matched: **4**
Sequence Coverage: **30%**

Matched peptides shown in **Bold Red**

1 PGAESAENWV SYWVVLVSSC SALLGIIGTE QSVEVIGK**LI CNGKPADDIK**
51 VK**LYDKEMIL DSK**LGEAHTN KEGFFKVSGH KR**ELSTLDPK** VNIYHRCNHD
101 GICDRKFSIS IPENFITDGE NPTK**TFDIGT INLADQFK**GE TTDCIN

Start - End	Observed	Mr(expt)	Mr(calc)	Delta	Miss	Sequence	
39 - 50	1343.99	1342.99	1342.69	0.29	0	K.LICNGKPADDIK.V	Carbamidomethyl (C)

53 - 63	1370.99	1369.99	1369.68	0.31	1	K.LYDKEMILDSK.L	Oxidation (M)
83 - 90	902.59	901.58	901.48	0.10	0	R.ELSTLDPK.V	
125 - 138	1583.06	1582.05	1581.80	0.25	0	K.TFDIGTINLADQFK.G	

No match to: 1345.00

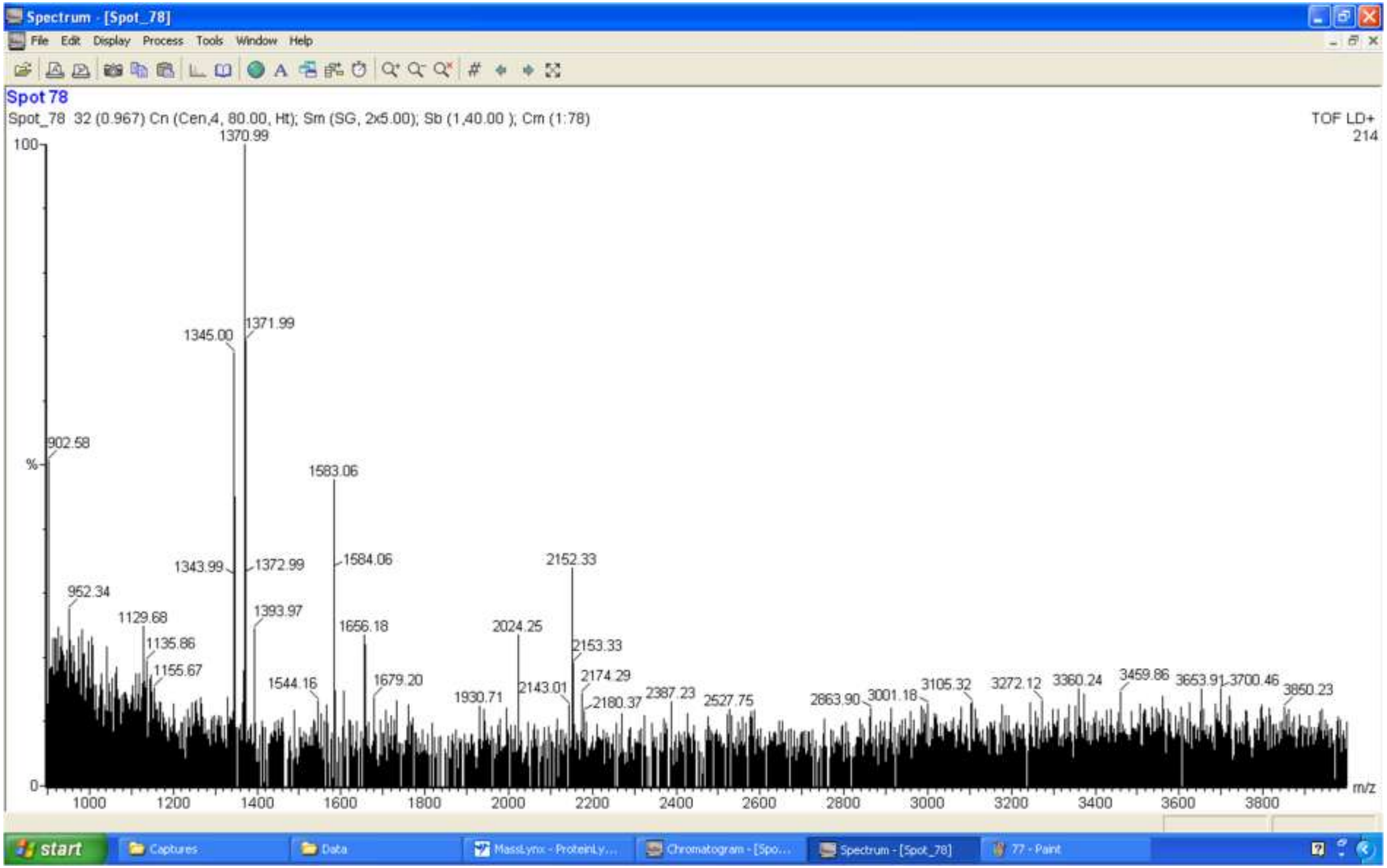

Spectrum - [Spot_78]
File Edit Display Process Tools Window Help
Spot 78
Spot_78 32 (0.967) Cn (Cen,4, 80.00, Ht); Sm (SG, 2x5.00); Sb (1,40.00); Cm (1:78)
TOF LD+
214
100
%
0
1370.99
1371.99
1345.00
902.58
1583.06
1343.99
1372.99
1584.06
2152.33
952.34
1393.97
1129.68
1656.18
2024.25
1135.86
2153.33
1155.67
2174.29
1544.16
1679.20
2143.01
3459.86
1930.71
2180.37
2387.23
2527.75
3105.32
3272.12
3360.24
3653.91
3700.46
3850.23
2863.90
3001.18
1000
1200
1400
1600
1800
2000
2200
2400
2600
2800
3000
3200
3400
3600
3800
m/z
start
Captures
Data
MassLynx - ProteinLy...
Chromatogram - [Spo...
Spectrum - [Spot_78]
77 - Paint

Figure S1.52

Mascot Search Results Spot 80

User : Paul Millares
Email : paul.millares@gmail.com
Search title : Spot 80
Database : Haemonchus 210108 (6387 sequences; 918038 residues)
Timestamp : 1 Aug 2011 at 10:59:44 GMT
Top Score : 45 for HCP11248_1, putative nuclear encoded protein Method: similarity and extension

Mascot Score Histogram

Protein score is -10*Log(P), where P is the probability that the observed match is a random event.

Protein scores greater than 51 are significant (p<0.05).

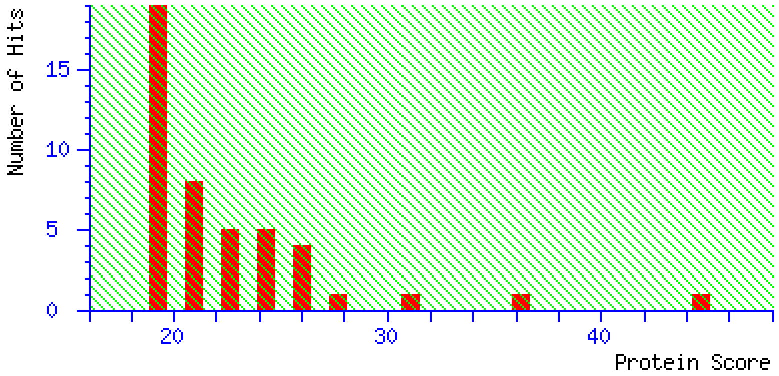

Concise Protein Summary Report

1. HCP11248_1 **Mass:** 14897 **Score:** 45 **Expect:** 0.22 **Matches:** 8

putative nuclear encoded protein Method: similarity and extension

---

Search Parameters

Type of search : Peptide Mass Fingerprint
Enzyme : Trypsin

Variable modifications : Carbamidomethyl (C),Glu->pyro-Glu (N-term E),Oxidation (M)
Mass values : Monoisotopic
Protein Mass : Unrestricted
Peptide Mass Tolerance : ± 1.2 Da
Peptide Charge State : 1+
Max Missed Cleavages : 1
Number of queries : 39

Protein View

Match to: **HCP11248_1** Score: **45** Expect: **0.22**
putative nuclear encoded protein Method: similarity and extension

Nominal mass (M_r): **14897**; Calculated pI value: **5.67**
NCBI BLAST search of HCP11248_1 against nr
Unformatted sequence string for pasting into other applications

Variable modifications: Carbamidomethyl (C),Glu->pyro-Glu (N-term E),Oxidation (M)
Cleavage by Trypsin: cuts C-term side of KR unless next residue is P
Number of mass values searched: **39**
Number of mass values matched: **8**
Sequence Coverage: **65%**

Matched peptides shown in **Bold Red**

```
  1 FDRLEKSLYP YWRDADHSVL HVANETQKVV DDDQKFAVAV DVSQFHPEEL
 51 KVHLEGRELT IEGKQQHKSD NSFMERSFIR KWTLPENVDL EAVRTQLNDS
101 GHLSVEAPKL TEGGTQRRTL PIERAPAKQ
```

Start - End	Observed	Mr(expt)	Mr(calc)	Delta	Miss	Sequence
7 - 13	984.61	983.60	983.49	0.12	0	K.SLYPYWR.D
14 - 28	1664.10	1663.09	1662.80	0.30	0	R.DADHSVLHVANETQK.V
36 - 51	1816.20	1815.19	1814.92	0.27	0	K.FAVAVDVSQFHPEELK.V
69 - 76	1000.59	999.59	1000.39	-0.80	0	K.SDNSFMER.S Oxidation (M)
81 - 94	1670.17	1669.16	1668.88	0.28	1	R.KWTLPENVDLEAVR.T
82 - 94	1542.10	1541.09	1540.79	0.30	0	K.WTLPENVDLEAVR.T
95 - 109	1596.10	1595.09	1594.80	0.29	0	R.TQLNDSGHLSVEAPK.L
110 - 118	1016.59	1015.58	1016.54	-0.95	1	K.LTEGGTQRR.T

No match to: 897.50, 900.39, 907.52, 914.42, 923.48, 929.50, 938.61, 940.62, 951.41, 982.58, 996.41, 1007.59, 1015.48, 1031.49, 1045.67, 1082.71, 1179.99, 1308.01, 1476.07, 1556.11, 1639.16, 1684.20, 1700.16, 1716.20, 1792.01, 1838.20, 1853.22, 2211.35, 2384.20, 2705.45, 2717.35

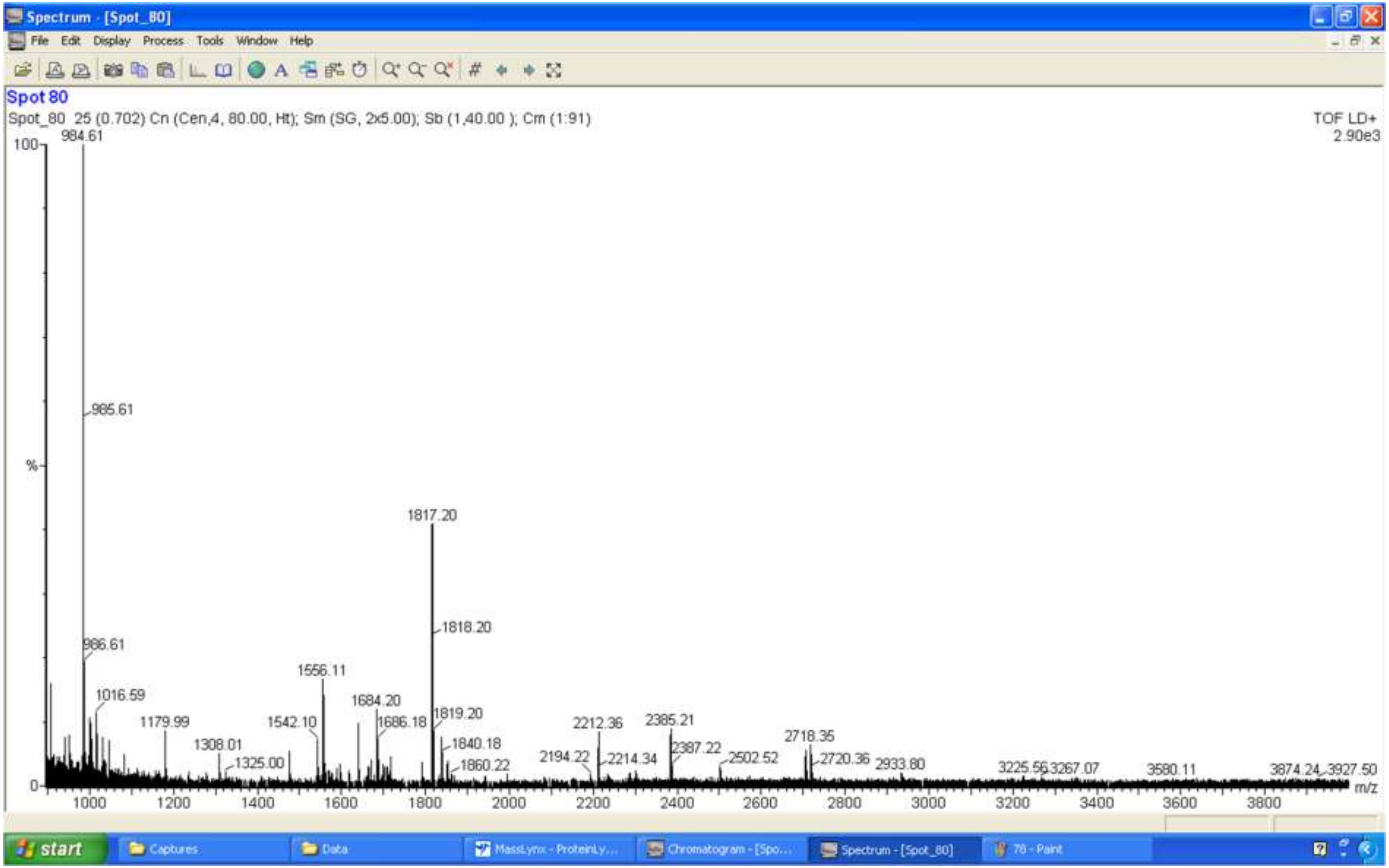
Spectrum - [Spot_80]
File Edit Display Process Tools Window Help
Spot 80
Spot_80 25 (0.702) Cn (Cen,4, 80.00, Ht); Sm (SG, 2x5.00); Sb (1,40.00); Cm (1:91)
TOF LD+
2.90e3
100
%
0
984.61
985.61
986.61
1016.59
1179.99
1308.01
1325.00
1542.10
1556.11
1684.20
1686.18
1817.20
1818.20
1819.20
1840.18
1860.22
2194.22
2212.36
2214.34
2385.21
2387.22
2502.52
2718.35
2720.36
2933.80
3225.56
3267.07
3580.11
3874.24
3927.50
m/z
1000 1200 1400 1600 1800 2000 2200 2400 2600 2800 3000 3200 3400 3600 3800
start
Captures
Data
MassLynx - ProteinLy...
Chromatogram - [Spo...
Spectrum - [Spot_80]
78 - Paint

Figure S1.53

Mascot Search Results Spot 81

User : **Paul Millares**
Email : **paul.millares@gmail.com**
Search title : **Spot 81**
Database : **Haemonchus 210108 (6387 sequences; 918038 residues)**
Timestamp : **1 Aug 2011 at 11:00:11 GMT**
Top Score : **61 for HCP00942_2, putative nuclear encoded protein Method: similarity and extension**

Mascot Score Histogram

Protein score is -10*Log(P), where P is the probability that the observed match is a random event.

Protein scores greater than 51 are significant (p<0.05).

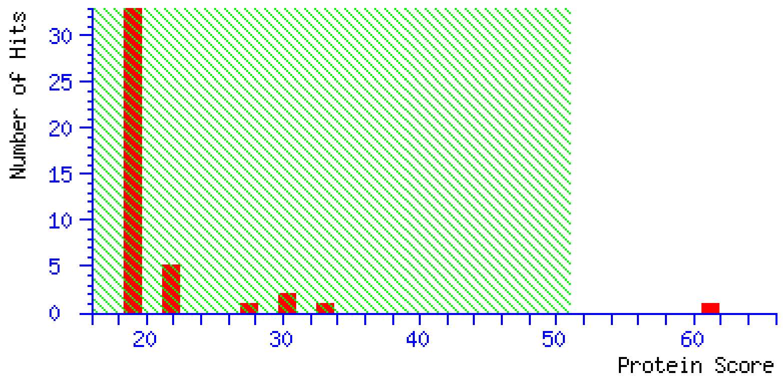

Concise Protein Summary Report

1. HCP00942_2 **Mass:** 17440 **Score:** 61 **Expect:** 0.0048 **Matches:** 19
   putative nuclear encoded protein Method: similarity and extension
   HCP00942_3 **Mass:** 16696 **Score:** 40 **Expect:** 0.62 **Matches:** 16
   putative nuclear encoded protein Method: similarity and extension

---

2. HCP06730_1 **Mass:** 6331 **Score:** 33 **Expect:** 3.4 **Matches:** 7

putative nuclear encoded protein Method: Longest ORF

Search Parameters

Type of search : Peptide Mass Fingerprint
Enzyme : Trypsin
Variable modifications : Carbamidomethyl (C),Glu->pyro-Glu (N-term E),Oxidation (M)
Mass values : Monoisotopic
Protein Mass : Unrestricted
Peptide Mass Tolerance : ± 1.2 Da
Peptide Charge State : 1+
Max Missed Cleavages : 1
Number of queries : 73

Protein View

Match to: **HCP00942_2** Score: **61** Expect: **0.0048**
putative nuclear encoded protein Method: similarity and extension

Nominal mass (M_r): **17440**; Calculated pI value: **6.31**
NCBI BLAST search of HCP00942_2 against nr
Unformatted sequence string for pasting into other applications

Variable modifications: Carbamidomethyl (C),Glu->pyro-Glu (N-term E),Oxidation (M)
Cleavage by Trypsin: cuts C-term side of KR unless next residue is P
Number of mass values searched: **73**
Number of mass values matched: **19**
Sequence Coverage: **83%**

Matched peptides shown in **Bold Red**

```
  1 MDFRPMPRLM NRIMYDTMRD MERFERSIFP YWREADHSVL HVANETQKLV
 51 DDDKKFAVAL DVSQFRPEEL NVHLEGRELT IEGKQEHKTE NSAMHRSFTR
101 KWILPENVNL EAIRTQLDDK GHLSVEAPKN VEGQPQKRNI PIMAAPKP
```

Start - End	Observed	Mr(expt)	Mr(calc)	Delta	Miss	Sequence
1 - 12	1579.20	1578.19	1578.76	-0.57	1	-.MDFRPMPRLMNR.I Oxidation (M)
2 - 8	935.43	934.42	933.45	0.97	0	M.DFRPMPR.L Oxidation (M)
9 - 19	1476.11	1475.10	1474.67	0.42	1	R.LMNRIMYDTMR.D 2 Oxidation (M)
13 - 19	944.45	943.45	944.41	-0.96	0	R.IMYDTMR.D Oxidation (M)
20 - 26	981.63	980.62	981.43	-0.81	1	R.DMERFER.S

20 - 26	998.44	997.44	997.43	0.01	1	R.DMERFER.S Oxidation (M)
27 - 33	967.62	966.61	967.49	-0.88	0	R.SIFPYWR.E
27 - 33	968.64	967.63	967.49	0.14	0	R.SIFPYWR.E
34 - 48	1678.15	1677.15	1676.81	0.34	0	R.EADHSVLHVANETQK.L
56 - 77	2526.61	2525.60	2525.30	0.30	0	K.FAVALDVSQFRPEELNVHLEGR.E
89 - 96	961.56	960.55	960.41	0.14	0	K.TENSAMHR.S Oxidation (M)
89 - 100	1436.12	1435.11	1435.66	-0.55	1	K.TENSAMHRSFTR.K
101 - 114	1695.29	1694.28	1693.95	0.33	1	R.KWILPENVNLEAIR.T
102 - 114	1567.20	1566.19	1565.86	0.34	0	K.WILPENVNLEAIR.T
121 - 129	937.65	936.65	936.50	0.14	0	K.GHLSVEAPK.N
130 - 137	899.60	898.59	898.45	0.14	0	K.NVEGQPQK.R
130 - 138	1055.68	1054.67	1054.55	0.12	1	K.NVEGQPQKR.N
139 - 148	1066.70	1065.69	1066.58	-0.89	0	R.NIPIMAAPKP.- Oxidation (M)
139 - 148	1067.72	1066.71	1066.58	0.13	0	R.NIPIMAAPKP.- Oxidation (M)

No match to: 907.42, 914.42, 921.58, 924.65, 951.42, 959.64, 974.63, 984.63, 991.62, 1000.63, 1006.61, 1011.48, 1018.48, 1022.61, 1036.66, 1043.49, 1045.70, 1073.63, 1075.62, 1077.66, 1087.65, 1089.72, 1103.72, 1111.80, 1113.82, 1119.79, 1126.83, 1132.83, 1135.84, 1142.84, 1149.88, 1151.88, 1158.89, 1165.98, 1167.90, 1253.04, 1264.06, 1278.07, 1380.06, 1479.11, 1550.18, 1574.19, 1581.19, 1589.17, 1597.17, 1599.19, 1707.20, 1711.28, 1721.26, 1724.24, 1727.27, 1835.26, 2025.32, 2211.40

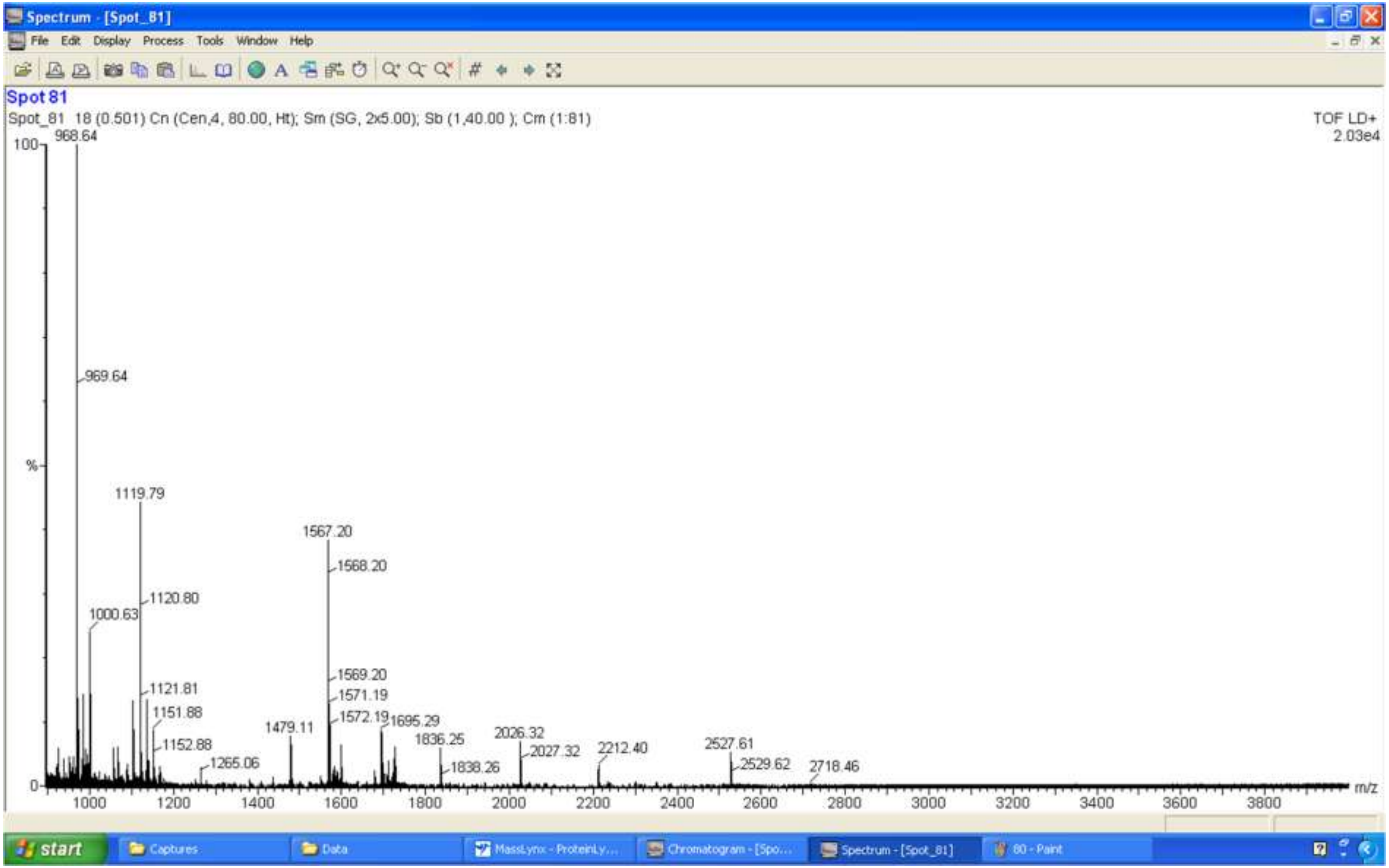

Spectrum - [Spot_81]
File Edit Display Process Tools Window Help
Spot 81
Spot_81 18 (0.501) Cn (Cen,4, 80.00, Ht); Sm (SG, 2x5.00); Sb (1,40.00); Cm (1:81)
TOF LD+
2.03e4
100
%
0
968.64
969.64
1119.79
1120.80
1000.63
1121.81
1151.88
1152.88
1265.06
1479.11
1567.20
1568.20
1569.20
1571.19
1572.19
1695.29
1836.25
1838.26
2026.32
2027.32
2212.40
2527.61
2529.62
2718.46
1000
1200
1400
1600
1800
2000
2200
2400
2600
2800
3000
3200
3400
3600
3800
m/z
start
Captures
Data
MassLynx - ProteinLy...
Chromatogram - [Spo...
Spectrum - [Spot_81]
80 - Paint

Figure S1.54

Mascot Search Results Spot 82

```
User            : Paul Millares
Email           : paul.millares@gmail.com
Search title    : Spot 82
Database        : Haemonchus 210108 (6387 sequences; 918038 residues)
Timestamp       : 1 Aug 2011 at 11:00:43 GMT
Top Score       : 41 for HCP00047_3, putative nuclear encoded protein  Method: similarity and extension
```

Mascot Score Histogram

Protein score is -10*Log(P), where P is the probability that the observed match is a random event.

Protein scores greater than 51 are significant (p<0.05).

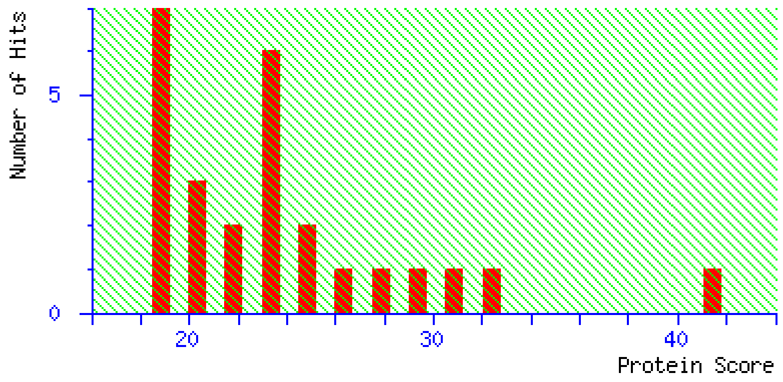

Concise Protein Summary Report

1. HCP00047_3 **Mass:** 16507 **Score:** 41 **Expect:** 0.46 **Matches:** 5
   putative nuclear encoded protein Method: similarity and extension
   HCP00047_2 **Mass:** 16229 **Score:** 30 **Expect:** 6 **Matches:** 4
   putative nuclear encoded protein Method: similarity and extension
   HCP00047_1 **Mass:** 17238 **Score:** 29 **Expect:** 7.7 **Matches:** 4
   putative nuclear encoded protein Method: similarity and extension

Search Parameters

Type of search : Peptide Mass Fingerprint
Enzyme : Trypsin
Variable modifications : Carbamidomethyl (C),Glu->pyro-Glu (N-term E),Oxidation (M)
Mass values : Monoisotopic
Protein Mass : Unrestricted
Peptide Mass Tolerance : ± 1.2 Da
Peptide Charge State : 1+
Max Missed Cleavages : 1
Number of queries : 13

Protein View

Match to: **HCP00047_3** Score: **41** Expect: **0.46**
putative nuclear encoded protein Method: similarity and extension

Nominal mass (M_r): **16507**; Calculated pI value: **6.39**
NCBI BLAST search of HCP00047_3 against nr
Unformatted sequence string for pasting into other applications

Variable modifications: Carbamidomethyl (C),Glu->pyro-Glu (N-term E),Oxidation (M)
Cleavage by Trypsin: cuts C-term side of KR unless next residue is P
Number of mass values searched: **13**
Number of mass values matched: **5**
Sequence Coverage: **43%**

Matched peptides shown in **Bold Red**

```
  1 MSNRAVAVLR GDPGVTGTVW FSQDKESDPC VIKGEIKGLT PGLHGFHVHQ
 51 YGDSTNGCTS AGPHFNPFNK THGGPKDDVR HVGDLGNVEA GADGVAHFEI
101 KDHLVKIHGE HTVVGRSLVV HAGTDDLGKG VGEKKEESLK TGNAGARVAC
151 GVIATAAPQ
```

Start - End	Observed	Mr(expt)	Mr(calc)	Delta	Miss	Sequence
11 - 25	1594.05	1593.04	1592.75	0.30	0	R.GDPGVTGTVWFSQDK.E
71 - 80	1081.66	1080.65	1080.53	0.12	1	K.THGGPKDDVR.H
81 - 101	2135.34	2134.33	2134.04	0.29	0	R.HVGDLGNVEAGADGVAHFEIK.D
107 - 116	1104.80	1103.79	1103.58	0.21	0	K.IHGEHTVVGR.S
117 - 129	1312.02	1311.02	1310.68	0.33	0	R.SLVVHAGTDDLGK.G

No match to: 900.39, 907.40, 951.39, 996.42, 1045.67, 1126.88, 1179.97, 1335.02

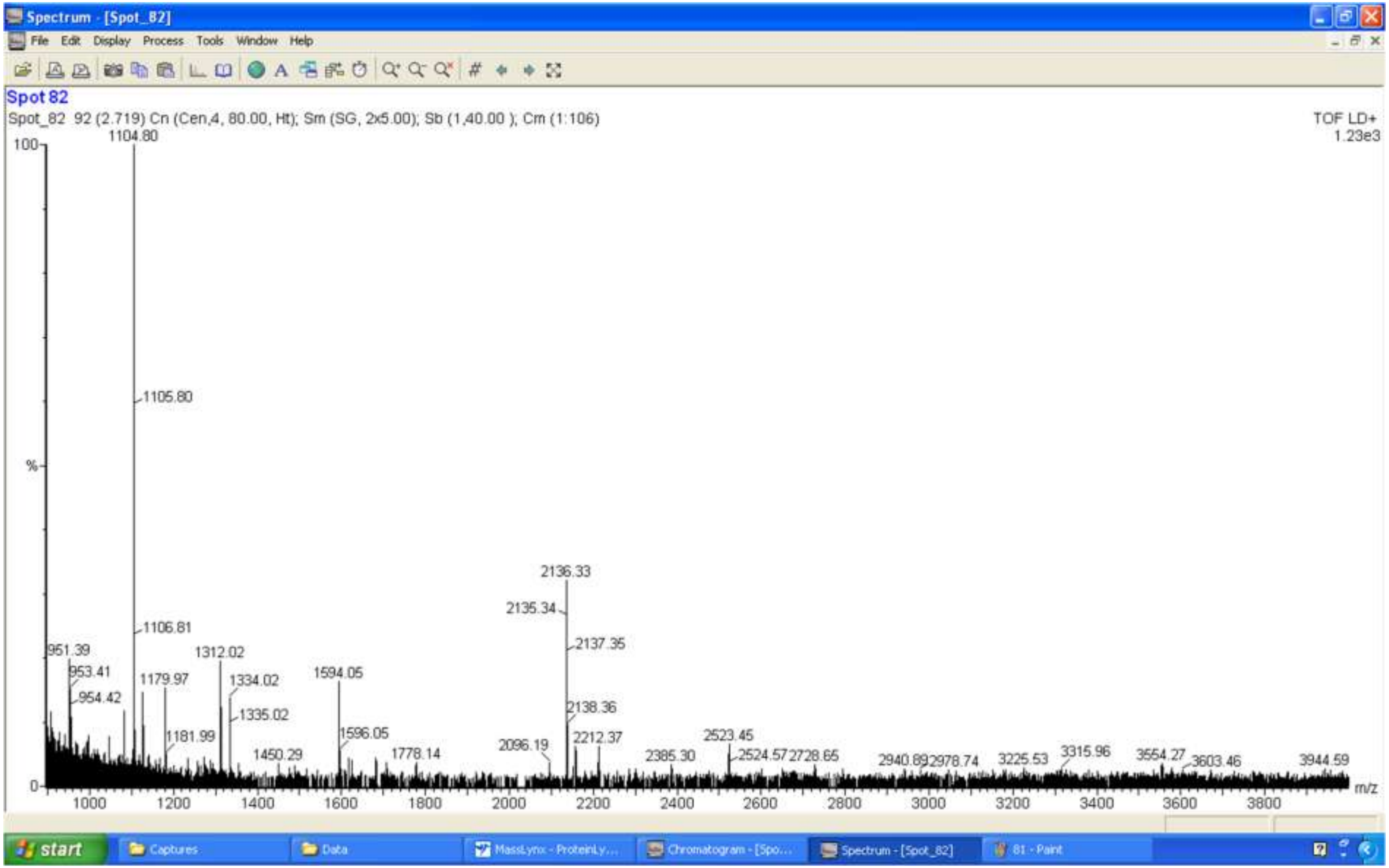

Figure S1.55

Mascot Search Results Spot 85

User : **Paul Millares**
Email : **paul.millares@gmail.com**
Search title : **Spot 85**
Database : **Haemonchus 210108 (6387 sequences; 918038 residues)**
Timestamp : **1 Aug 2011 at 11:01:05 GMT**
Top Score : **85 for HCP00208_1, putative nuclear encoded protein Method: similarity and extension**

Mascot Score Histogram

Protein score is -10*Log(P), where P is the probability that the observed match is a random event.

Protein scores greater than 51 are significant (p<0.05).

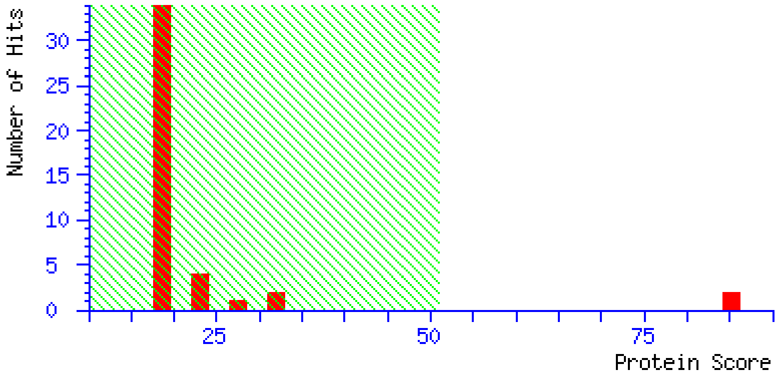

Concise Protein Summary Report

1. HCP00208_1 **Mass:** 19222 **Score:** **85** **Expect:** 1.9e-005 **Matches:** 13
putative nuclear encoded protein Method: similarity and extension

---

2. HCP00208_2 **Mass:** 19583 **Score:** **84** **Expect:** 2.3e-005 **Matches:** 13
putative nuclear encoded protein Method: similarity and extension
HCP13111_1 **Mass:** 18607 **Score:** **65** **Expect:** 0.0022 **Matches:** 11

putative nuclear encoded protein Method: similarity and extension

---

3. HCP00910_1 **Mass:** 7497 **Score:** 30 **Expect:** 6 **Matches:** 7

putative nuclear encoded protein Method: Longest ORF

Search Parameters

Type of search : Peptide Mass Fingerprint
Enzyme : Trypsin
Variable modifications : Carbamidomethyl (C),Glu->pyro-Glu (N-term E),Oxidation (M)
Mass values : Monoisotopic
Protein Mass : Unrestricted
Peptide Mass Tolerance : ± 1.2 Da
Peptide Charge State : 1+
Max Missed Cleavages : 1
Number of queries : 41

Protein View

Match to: **HCP00208_1** Score: **85** Expect: **1.9e-005**
putative nuclear encoded protein Method: similarity and extension

Nominal mass (M_r): **19222**; Calculated pI value: **6.59**
NCBI BLAST search of HCP00208_1 against nr
Unformatted sequence string for pasting into other applications

Variable modifications: Carbamidomethyl (C),Glu->pyro-Glu (N-term E),Oxidation (M)
Cleavage by Trypsin: cuts C-term side of KR unless next residue is P
Number of mass values searched: **41**
Number of mass values matched: **13**
Sequence Coverage: **68%**

Matched peptides shown in **Bold Red**

```
  1 LIATLLIAYA TATSPEDVKK NAVAALEHAP LGTTPEKDHI GRDFYKHYFT
 51 KHPEVRKYFI GAESITPDEV DKSERFKKQG TRLLTAVHVL ANTYDNDAVF
101 RGFVRDLIHR HSDKRIDPKE WKEIWSSIES FLETRGTSLT AEQKAALEAI
151 GNKFNEEAQK DLAAHGHPHV
```

Start - End Observed Mr(expt) Mr(calc) Delta Miss Sequence

21 - 37	1719.33	1718.33	1717.90	0.43	0	K.NAVAALEHAPLGTTPEK.D
21 - 42	2297.67	2296.66	2296.19	0.47	1	K.NAVAALEHAPLGTTPEKDHIGR.D
38 - 46	1149.56	1148.56	1149.56	-1.00	1	K.DHIGRDFYK.H
43 - 51	1249.13	1248.12	1247.60	0.53	1	R.DFYKHYFTK.H
57 - 72	1812.37	1811.36	1810.90	0.46	1	R.KYFIGAESITPDEVDK.S
58 - 72	1684.26	1683.25	1682.80	0.45	0	K.YFIGAESITPDEVDK.S
58 - 75	2056.44	2055.43	2054.98	0.45	1	K.YFIGAESITPDEVDKSER.F
83 - 101	2132.57	2131.57	2131.11	0.46	0	R.LLTAVHVLANTYDNDAVFR.G
123 - 135	1597.21	1596.21	1595.78	0.42	0	K.EIWSSIESFLETR.G
136 - 144	934.67	933.66	933.48	0.19	0	R.GTSLTAEQK.A
145 - 160	1733.34	1732.33	1731.88	0.45	1	K.AALEAIGNKFNEEAQK.D
154 - 170	1900.34	1899.34	1898.90	0.43	1	K.FNEEAQKDLAAHGHPHV.-
161 - 170	1053.72	1052.71	1052.52	0.19	0	K.DLAAHGHPHV.-

No match to: 900.46, 907.47, 917.48, 951.47, 997.50, 1051.90, 1066.76, 1075.71, 1092.80, 1100.76, 1166.22, 1182.06, 1199.13, 1241.00, 1302.08, 1499.19, 1539.22, 1555.22, 1571.20, 1583.20, 1850.28, 1961.43, 2036.53, 2114.58, 2146.58, 2184.54, 2211.57, 2325.65

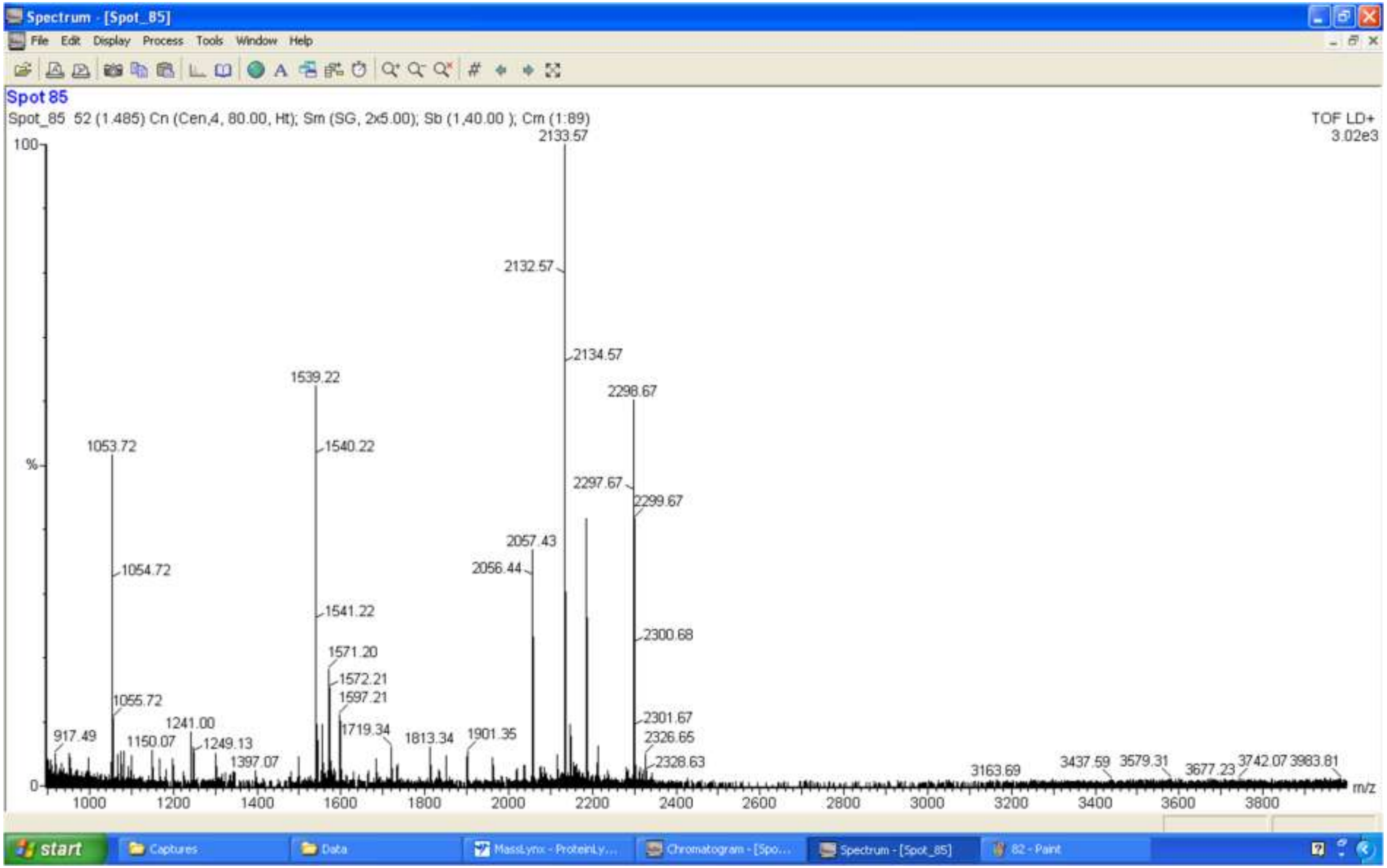
Spectrum - [Spot_85]
File Edit Display Process Tools Window Help
Spot 85
Spot_85 52 (1.485) Cn (Cen,4, 80.00, Ht); Sm (SG, 2x5.00); Sb (1,40.00); Cm (1:89)
TOF LD+
3.02e3
100
%
0
2133.57
2132.57
2134.57
2298.67
1539.22
1540.22
1053.72
2297.67
2299.67
2057.43
2056.44
1054.72
1541.22
2300.68
1571.20
1572.21
1597.21
1055.72
2301.67
1241.00
917.49
1719.34
1813.34
1901.35
2326.65
1150.07
1249.13
1397.07
2328.63
3163.69
3437.59
3579.31
3677.23
3742.07
3983.81
m/z
1000 1200 1400 1600 1800 2000 2200 2400 2600 2800 3000 3200 3400 3600 3800
start
Captures
Data
MassLynx - ProteinLy...
Chromatogram - [Spo...
Spectrum - [Spot_85]
82 - Paint

Figure S1.56

Mascot Search Results Spot 86

```
User          : Paul Millares
Email         : paul.millares@gmail.com
Search title  : Spot 86
Database      : Haemonchus 210108 (6387 sequences; 918038 residues)
Timestamp     : 1 Aug 2011 at 11:01:35 GMT
Top Score     : 87 for HCP02230_2, putative nuclear encoded protein  Method: similarity and extension
```

Mascot Score Histogram

Protein score is -10*Log(P), where P is the probability that the observed match is a random event.

Protein scores greater than 51 are significant (p<0.05).

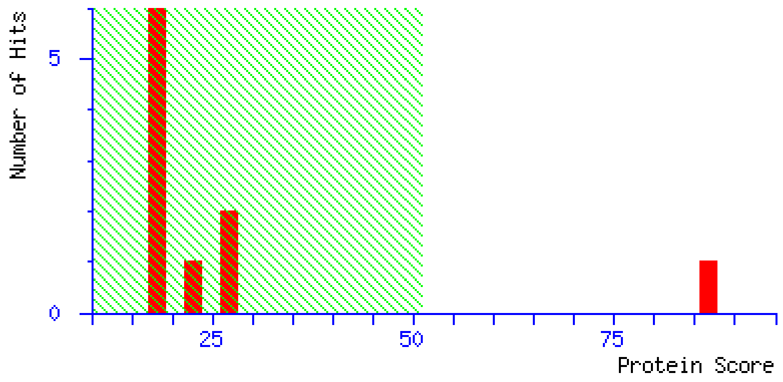

Concise Protein Summary Report

1. HCP02230_2 **Mass:** 14149 **Score:** 87 **Expect:** 1.4e-005 **Matches:** 9
   putative nuclear encoded protein Method: similarity and extension
   HCP02230_1 **Mass:** 14175 **Score:** 87 **Expect:** 1.4e-005 **Matches:** 9
   putative nuclear encoded protein Method: similarity and extension
   HCP02230_3 **Mass:** 13987 **Score:** 87 **Expect:** 1.4e-005 **Matches:** 9
   putative nuclear encoded protein Method: similarity and extension

HCP11793_1 **Mass:** 14292 **Score:** **85** **Expect:** 2.2e-005 **Matches:** 9
putative nuclear encoded protein Method: similarity and extension
HCP03065_1 **Mass:** 21802 **Score:** 25 **Expect:** 22 **Matches:** 4
putative nuclear encoded protein Method: similarity and extension
HCP06220_1 **Mass:** 11908 **Score:** 24 **Expect:** 24 **Matches:** 3
putative nuclear encoded protein Method: ESTScan
HCP02256_1 **Mass:** 4155 **Score:** 22 **Expect:** 42 **Matches:** 2
putative nuclear encoded protein Method: ESTScan
HCP09760_1 **Mass:** 20548 **Score:** 21 **Expect:** 54 **Matches:** 3
putative nuclear encoded protein Method: similarity and extension
HCP01900_1 **Mass:** 17456 **Score:** 20 **Expect:** 61 **Matches:** 3
putative nuclear encoded protein Method: similarity and extension
HCP09751_1 **Mass:** 8909 **Score:** 20 **Expect:** 65 **Matches:** 3
putative nuclear encoded protein Method: ESTScan
HCP12819_1 **Mass:** 22362 **Score:** 20 **Expect:** 67 **Matches:** 3
putative nuclear encoded protein Method: similarity and extension
HCP00319_2 **Mass:** 21269 **Score:** 20 **Expect:** 70 **Matches:** 3
putative nuclear encoded protein Method: similarity and extension
HCP04621_2 **Mass:** 23622 **Score:** 20 **Expect:** 70 **Matches:** 3
putative nuclear encoded protein Method: similarity and extension
HCP04930_2 **Mass:** 6606 **Score:** 20 **Expect:** 72 **Matches:** 2
putative nuclear encoded protein Method: ESTScan
HCP04621_1 **Mass:** 23837 **Score:** 20 **Expect:** 72 **Matches:** 3
putative nuclear encoded protein Method: similarity and extension
HCP00319_1 **Mass:** 22342 **Score:** 19 **Expect:** 77 **Matches:** 3
putative nuclear encoded protein Method: similarity and extension
HCP08671_1 **Mass:** 9034 **Score:** 19 **Expect:** 84 **Matches:** 2
putative nuclear encoded protein Method: Longest ORF
HCP00848_1 **Mass:** 6852 **Score:** 19 **Expect:** 84 **Matches:** 2
putative nuclear encoded protein Method: ESTScan

HCP04930_1 **Mass:** 7043 **Score:** 19 **Expect:** 86 **Matches:** 3
putative nuclear encoded protein Method: ESTScan
HCP00359_2 **Mass:** 24368 **Score:** 19 **Expect:** 90 **Matches:** 3
putative nuclear encoded protein Method: ESTScan
HCP05284_1 **Mass:** 7409 **Score:** 18 **Expect:** 99 **Matches:** 2
putative nuclear encoded protein Method: ESTScan
HCP01102_1 **Mass:** 30812 **Score:** 18 **Expect:** 1e+002 **Matches:** 4
putative nuclear encoded protein Method: similarity and extension
HCP00007_2 **Mass:** 53234 **Score:** 18 **Expect:** 1e+002 **Matches:** 4
putative nuclear encoded protein Method: similarity and extension
HCP00007_1 **Mass:** 53234 **Score:** 18 **Expect:** 1e+002 **Matches:** 4
putative nuclear encoded protein Method: similarity and extension
HCP07804_1 **Mass:** 8558 **Score:** 18 **Expect:** 1.1e+002 **Matches:** 2
putative nuclear encoded protein Method: ESTScan
HCP01247_1 **Mass:** 10022 **Score:** 18 **Expect:** 1.1e+002 **Matches:** 2
putative nuclear encoded protein Method: Longest ORF
HCP07146_1 **Mass:** 27383 **Score:** 18 **Expect:** 1.1e+002 **Matches:** 3
putative nuclear encoded protein Method: ESTScan
HCP02139_1 **Mass:** 8564 **Score:** 18 **Expect:** 1.1e+002 **Matches:** 2
putative nuclear encoded protein Method: similarity and extension
HCP01548_1 **Mass:** 24949 **Score:** 18 **Expect:** 1.1e+002 **Matches:** 3
putative nuclear encoded protein Method: similarity and extension
HCP11099_1 **Mass:** 10426 **Score:** 18 **Expect:** 1.1e+002 **Matches:** 2
putative nuclear encoded protein Method: similarity and extension
HCP12446_1 **Mass:** 8454 **Score:** 17 **Expect:** 1.2e+002 **Matches:** 2
putative nuclear encoded protein Method: ESTScan

---

2. HCP03152_2 **Mass:** 13553 **Score:** 27 **Expect:** 14 **Matches:** 4
putative nuclear encoded protein Method: similarity and extension
HCP03152_1 **Mass:** 13610 **Score:** 26 **Expect:** 15 **Matches:** 4

putative nuclear encoded protein Method: similarity and extension

HCP02854_1 **Mass:** 7039 **Score:** 19 **Expect:** 77 **Matches:** 2

putative nuclear encoded protein Method: Longest ORF

HCP08752_1 **Mass:** 25210 **Score:** 19 **Expect:** 77 **Matches:** 3

putative nuclear encoded protein Method: similarity and extension

HCP01860_1 **Mass:** 13671 **Score:** 19 **Expect:** 90 **Matches:** 3

putative nuclear encoded protein Method: similarity and extension

---

Search Parameters

Type of search : Peptide Mass Fingerprint
Enzyme : Trypsin
Variable modifications : Carbamidomethyl (C),Glu->pyro-Glu (N-term E),Oxidation (M)
Mass values : Monoisotopic
Protein Mass : Unrestricted
Peptide Mass Tolerance : ± 1.2 Da
Peptide Charge State : 1+
Max Missed Cleavages : 1
Number of queries : 10

Protein View

Match to: **HCP02230_2** Score: **87** Expect: **1.4e-005**
putative nuclear encoded protein Method: similarity and extension

Nominal mass (M_r): **14149**; Calculated pI value: **7.68**
NCBI BLAST search of HCP02230_2 against nr
Unformatted sequence string for pasting into other applications

Variable modifications: Carbamidomethyl (C),Glu->pyro-Glu (N-term E),Oxidation (M)
Cleavage by Trypsin: cuts C-term side of KR unless next residue is P
Number of mass values searched: **10**
Number of mass values matched: **9**
Sequence Coverage: **56%**

Matched peptides shown in **Bold Red**

1 SSVPPGDINT QPNSK**IVFNA PYDDKHTYHI K**ITNASGRRI GWAIKTTNMR
51 R**LGVDPACGV LDPKEATLMA VSCDVFDYGR** EDTNNDR**ITV EWCNTPEGAA**

101 **K**QFRR**EWFQG DGMVRR**KNLP IEYNP

Start - End	Observed	Mr(expt)	Mr(calc)	Delta	Miss	Sequence
16 - 25	1182.02	1181.02	1180.58	0.44	0	K.IVFNAPYDDK.H
16 - 31	1961.36	1960.35	1959.98	0.36	1	K.IVFNAPYDDKHTYHIK.I
52 - 64	1341.07	1340.06	1339.68	0.38	0	R.LGVDPACGVLDPK.E Carbamidomethyl (C)
65 - 80	1834.18	1833.18	1832.81	0.37	0	K.EATLMAVSCDVFDYGR.E Carbamidomethyl (C)
65 - 80	1850.18	1849.17	1848.80	0.37	0	K.EATLMAVSCDVFDYGR.E Carbamidomethyl (C); Oxidation (M)
88 - 101	1576.12	1575.11	1574.74	0.37	0	R.ITVEWCNTPEGAAK.Q Carbamidomethyl (C)
106 - 115	1224.97	1223.96	1223.54	0.42	0	R.EWFQGDGMVR.R
106 - 115	1240.96	1239.95	1239.53	0.42	0	R.EWFQGDGMVR.R Oxidation (M)
106 - 116	1397.02	1396.02	1395.64	0.38	1	R.EWFQGDGMVRR.K Oxidation (M)

No match to: 1272.94

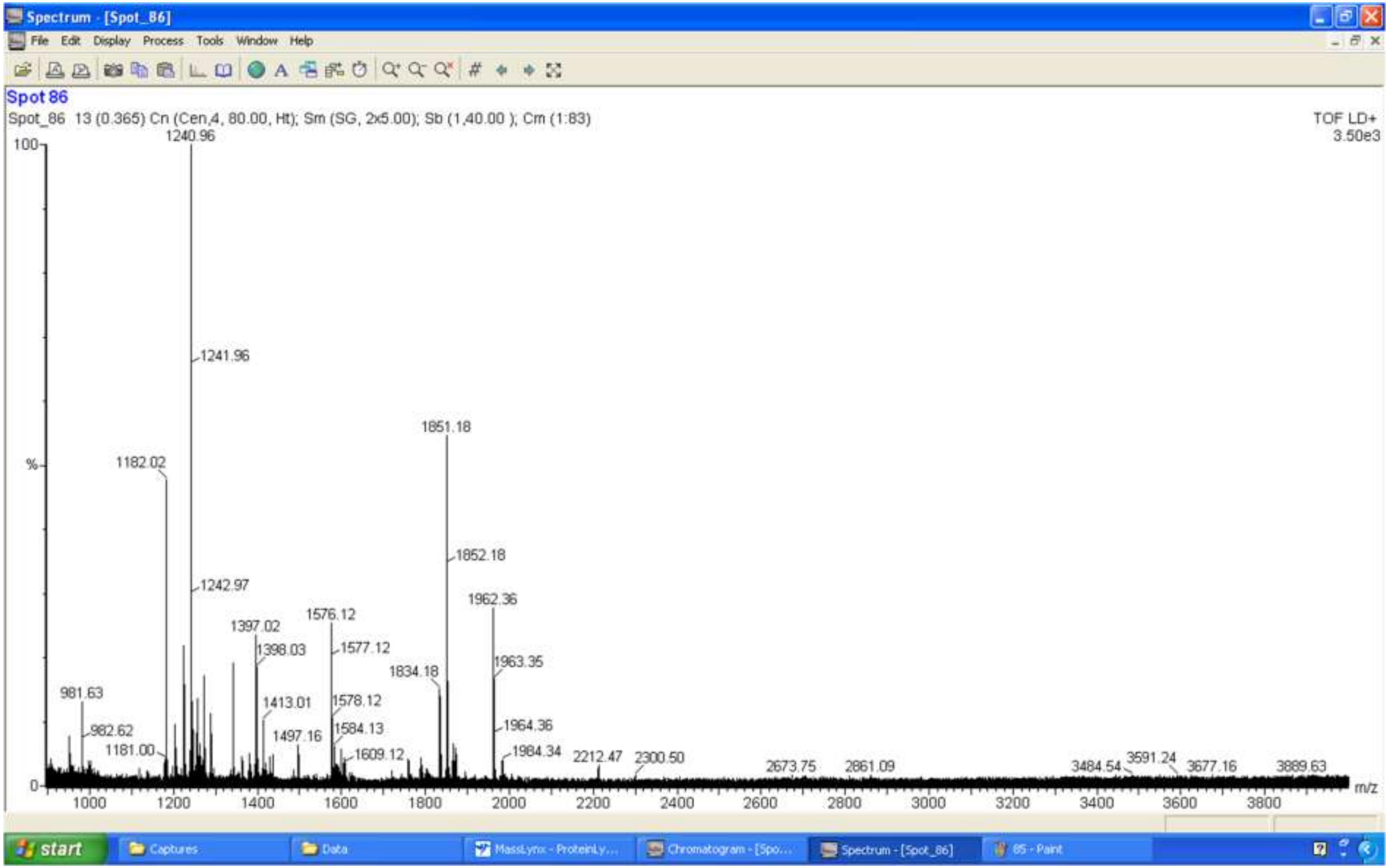

Spectrum - [Spot_86]
File Edit Display Process Tools Window Help
Spot 86
Spot_86 13 (0.365) Cn (Cen,4, 80.00, Ht); Sm (SG, 2x5.00); Sb (1,40.00); Cm (1:83)
TOF LD+
3.50e3
100
%
0
1240.96
1241.96
1182.02
1851.18
1852.18
1242.97
1962.36
1397.02
1576.12
1398.03
1577.12
1834.18
1963.35
981.63
1413.01
1578.12
1497.16
1584.13
1964.36
982.62
1181.00
1609.12
1984.34
2212.47
2300.50
2673.75
2861.09
3484.54
3591.24
3677.16
3889.63
1000 1200 1400 1600 1800 2000 2200 2400 2600 2800 3000 3200 3400 3600 3800
m/z
start
Captures
Data
MassLynx - ProteinLy...
Chromatogram - [Spo...
Spectrum - [Spot_86]
85 - Paint

Figure S1.57

Mascot Search Results Spot 88

```
User            : Paul Millares
Email           : paul.millares@gmail.com
Search title    : Spot 88
Database        : Haemonchus 210108 (6387 sequences; 918038 residues)
Timestamp       : 1 Aug 2011 at 11:01:55 GMT
Top Score       : 54 for HCP00202_5, putative nuclear encoded protein  Method: similarity and extension
```

Mascot Score Histogram

Protein score is -10*Log(P), where P is the probability that the observed match is a random event.

Protein scores greater than 51 are significant (p<0.05).

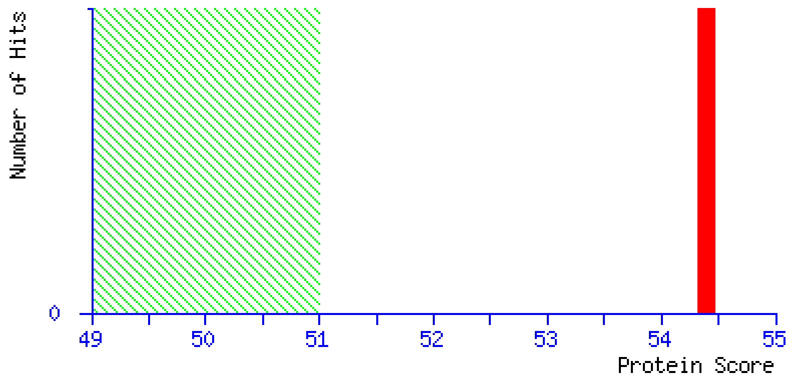

Concise Protein Summary Report

1. HCP00202_5 **Mass:** 19654 **Score:** 54 **Expect:** 0.023 **Matches:** 4

   putative nuclear encoded protein Method: similarity and extension

   HCP00202_4 **Mass:** 19330 **Score:** 36 **Expect:** 1.8 **Matches:** 3

   putative nuclear encoded protein Method: similarity and extension

   HCP00333_2 **Mass:** 19591 **Score:** 36 **Expect:** 1.8 **Matches:** 3

   putative nuclear encoded protein Method: similarity and extension

HCP00202_3 **Mass:** 19726 **Score:** 35 **Expect:** 2 **Matches:** 3
putative nuclear encoded protein Method: similarity and extension
HCP01346_1 **Mass:** 15748 **Score:** 32 **Expect:** 3.8 **Matches:** 3
putative nuclear encoded protein Method: similarity and extension
HCP06643_1 **Mass:** 4096 **Score:** 31 **Expect:** 4.6 **Matches:** 2
putative nuclear encoded protein Method: Longest ORF
HCP04874_2 **Mass:** 7436 **Score:** 28 **Expect:** 11 **Matches:** 2
putative nuclear encoded protein Method: ESTScan
HCP03043_2 **Mass:** 28046 **Score:** 26 **Expect:** 15 **Matches:** 3
putative nuclear encoded protein Method: similarity and extension
HCP03043_1 **Mass:** 28176 **Score:** 26 **Expect:** 16 **Matches:** 3
putative nuclear encoded protein Method: similarity and extension
HCP03252_2 **Mass:** 6347 **Score:** 26 **Expect:** 18 **Matches:** 2
putative nuclear encoded protein Method: Longest ORF
HCP00630_1 **Mass:** 12040 **Score:** 24 **Expect:** 29 **Matches:** 2
putative mitochondrial protein Method: similarity and extension
HCP00326_1 **Mass:** 47759 **Score:** 23 **Expect:** 35 **Matches:** 3
putative nuclear encoded protein Method: similarity and extension
HCP03265_1 **Mass:** 14639 **Score:** 22 **Expect:** 43 **Matches:** 2
putative nuclear encoded protein Method: ESTScan
HCP11248_1 **Mass:** 14897 **Score:** 22 **Expect:** 43 **Matches:** 2
putative nuclear encoded protein Method: similarity and extension
HCP01778_1 **Mass:** 13800 **Score:** 21 **Expect:** 48 **Matches:** 2
putative nuclear encoded protein Method: similarity and extension
HCP12737_1 **Mass:** 18074 **Score:** 21 **Expect:** 54 **Matches:** 2
putative nuclear encoded protein Method: ESTScan
HCP10851_1 **Mass:** 14124 **Score:** 21 **Expect:** 54 **Matches:** 2
putative nuclear encoded protein Method: similarity and extension
HCP11517_1 **Mass:** 21101 **Score:** 20 **Expect:** 64 **Matches:** 2
putative nuclear encoded protein Method: similarity and extension

HCP06532_2 **Mass:** 16814 **Score:** 20 **Expect:** 67 **Matches:** 2
putative nuclear encoded protein Method: similarity and extension
HCP06532 **Mass:** 16814 **Score:** 20 **Expect:** 67 **Matches:** 2
putative nuclear encoded protein Method: similarity and extension
HCP01043_1 **Mass:** 16440 **Score:** 20 **Expect:** 67 **Matches:** 2
putative nuclear encoded protein Method: ESTScan
HCP05076_2 **Mass:** 18986 **Score:** 20 **Expect:** 68 **Matches:** 2
putative nuclear encoded protein Method: ESTScan
HCP02568_1 **Mass:** 18387 **Score:** 20 **Expect:** 68 **Matches:** 2
putative nuclear encoded protein Method: ESTScan
HCP04244_1 **Mass:** 19212 **Score:** 20 **Expect:** 68 **Matches:** 2
putative nuclear encoded protein Method: similarity and extension
HCP09095_1 **Mass:** 19519 **Score:** 20 **Expect:** 70 **Matches:** 2
putative nuclear encoded protein Method: similarity and extension
HCP11794_1 **Mass:** 11516 **Score:** 20 **Expect:** 70 **Matches:** 2
putative nuclear encoded protein Method: ESTScan
HCP01931_1 **Mass:** 12161 **Score:** 20 **Expect:** 70 **Matches:** 2
putative nuclear encoded protein Method: similarity and extension
HCP00396_1 **Mass:** 20068 **Score:** 20 **Expect:** 72 **Matches:** 2
putative nuclear encoded protein Method: similarity and extension
HCP03268_2 **Mass:** 19831 **Score:** 20 **Expect:** 72 **Matches:** 2
putative nuclear encoded protein Method: ESTScan
HCP04949_1 **Mass:** 19648 **Score:** 20 **Expect:** 72 **Matches:** 2
putative nuclear encoded protein Method: ESTScan
HCP09394_2 **Mass:** 5508 **Score:** 19 **Expect:** 73 **Matches:** 2
putative nuclear encoded protein Method: similarity and extension
HCP02059_1 **Mass:** 12513 **Score:** 19 **Expect:** 75 **Matches:** 2
putative nuclear encoded protein Method: similarity and extension
HCP00442_1 **Mass:** 17803 **Score:** 19 **Expect:** 75 **Matches:** 2
putative nuclear encoded protein Method: similarity and extension

HCP04530_1 **Mass:** 21067 **Score:** 19 **Expect:** 77 **Matches:** 2
putative nuclear encoded protein Method: similarity and extension
HCP01138_1 **Mass:** 20615 **Score:** 19 **Expect:** 79 **Matches:** 2
putative nuclear encoded protein Method: ESTScan
HCP07636_1 **Mass:** 12464 **Score:** 19 **Expect:** 79 **Matches:** 2
putative nuclear encoded protein Method: ESTScan
HCP05076_1 **Mass:** 20702 **Score:** 19 **Expect:** 79 **Matches:** 2
putative nuclear encoded protein Method: ESTScan
HCP02189_1 **Mass:** 23381 **Score:** 19 **Expect:** 79 **Matches:** 2
putative nuclear encoded protein Method: similarity and extension
HCP03268_1 **Mass:** 20824 **Score:** 19 **Expect:** 80 **Matches:** 2
putative nuclear encoded protein Method: ESTScan
HCP06769_1 **Mass:** 25352 **Score:** 19 **Expect:** 80 **Matches:** 2
putative nuclear encoded protein Method: ESTScan
HCP06769_2 **Mass:** 25352 **Score:** 19 **Expect:** 80 **Matches:** 2
putative nuclear encoded protein Method: ESTScan
HCP00039_1 **Mass:** 19115 **Score:** 19 **Expect:** 80 **Matches:** 2
putative nuclear encoded protein Method: similarity and extension
HCP05563_1 **Mass:** 21252 **Score:** 19 **Expect:** 80 **Matches:** 2
putative nuclear encoded protein Method: ESTScan
HCP00327_1 **Mass:** 21943 **Score:** 19 **Expect:** 84 **Matches:** 2
putative nuclear encoded protein Method: similarity and extension
HCP00766_1 **Mass:** 19287 **Score:** 19 **Expect:** 84 **Matches:** 2
putative nuclear encoded protein Method: ESTScan
HCP07449_1 **Mass:** 21645 **Score:** 19 **Expect:** 86 **Matches:** 2
putative nuclear encoded protein Method: similarity and extension
HCP04006_1 **Mass:** 21878 **Score:** 19 **Expect:** 88 **Matches:** 2
putative nuclear encoded protein Method: ESTScan
HCP06239_1 **Mass:** 19794 **Score:** 19 **Expect:** 88 **Matches:** 2
putative nuclear encoded protein Method: similarity and extension

HCP13315_1 **Mass:** 22236 **Score:** 19 **Expect:** 88 **Matches:** 2
putative nuclear encoded protein Method: similarity and extension
HCP01160_2 **Mass:** 22139 **Score:** 19 **Expect:** 88 **Matches:** 2
putative nuclear encoded protein Method: similarity and extension

---

Search Parameters

Type of search : Peptide Mass Fingerprint
Enzyme : Trypsin
Variable modifications : Carbamidomethyl (C),Glu->pyro-Glu (N-term E),Oxidation (M)
Mass values : Monoisotopic
Protein Mass : Unrestricted
Peptide Mass Tolerance : ± 1.2 Da
Peptide Charge State : 1+
Max Missed Cleavages : 1
Number of queries : 4

Protein View

Match to: **HCP00202_5** Score: **54** Expect: **0.023**
putative nuclear encoded protein Method: similarity and extension

Nominal mass (M_r): **19654**; Calculated pI value: **6.20**
NCBI BLAST search of HCP00202_5 against nr
Unformatted sequence string for pasting into other applications

Variable modifications: Carbamidomethyl (C),Glu->pyro-Glu (N-term E),Oxidation (M)
Cleavage by Trypsin: cuts C-term side of KR unless next residue is P
Number of mass values searched: **4**
Number of mass values matched: **4**
Sequence Coverage: **29%**

Matched peptides shown in **Bold Red**

1 MQLLILTLAV FVAFATAMAP EDVKK**NAVAA LESVPVGTTP DK**IQNGKDFY
51 KYFFTKHPEL LFYFK**GAESF TADDVQNTDR** FVKQGGNLLT GVHVLANTFD
101 NDMVFRAYVR DLMNRHTAKE IDPKQWKDFF GCFEKFLEGR **GKPLTDDQK**A
151 ALEAIGTKFN EEAQK**HLATL GLPHA**

Start - End Observed Mr(expt) Mr(calc) Delta Miss Sequence

26 - 42	1669.13	1668.12	1667.87	0.25	0	K.NAVAALESVPVGTTPDK.I
66 - 80	1625.92	1624.91	1624.70	0.21	0	K.GAESFTADDVQNTDR.F
141 - 149	1001.65	1000.64	1000.52	0.13	0	R.GKPLTDDQK.A
166 - 175	1029.66	1028.65	1028.58	0.07	0	K.HLATLGLPHA.-

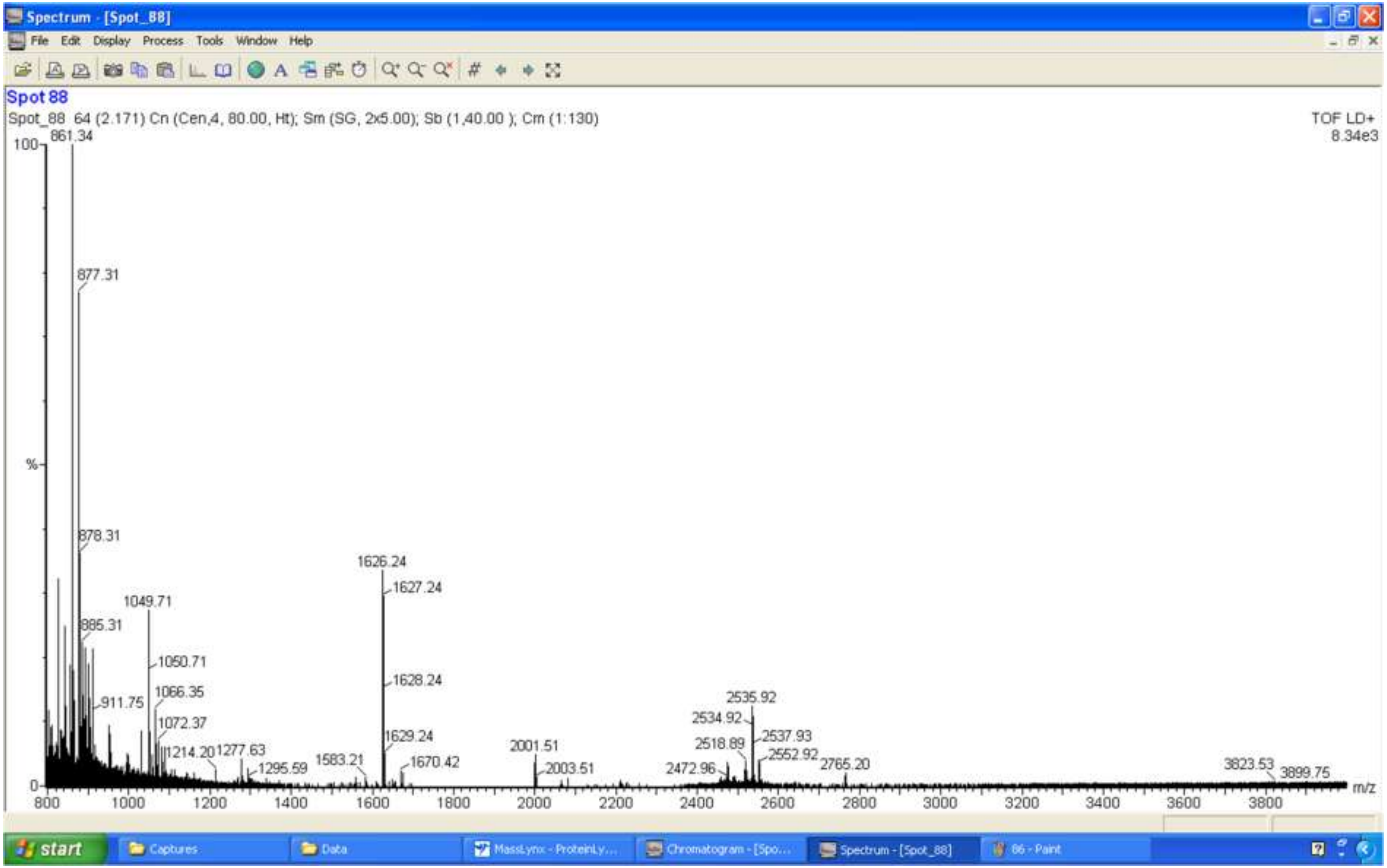

Spectrum - [Spot_88]
File Edit Display Process Tools Window Help
Spot 88
Spot_88 64 (2.171) Cn (Cen,4, 80.00, Ht); Sm (SG, 2x5.00); Sb (1,40.00); Cm (1:130)
TOF LD+
8.34e3
100
%
0
861.34
877.31
878.31
885.31
911.75
1049.71
1050.71
1066.35
1072.37
1214.20
1277.63
1295.59
1583.21
1626.24
1627.24
1628.24
1629.24
1670.42
2001.51
2003.51
2472.96
2518.89
2534.92
2535.92
2537.93
2552.92
2765.20
3823.53
3899.75
800
1000
1200
1400
1600
1800
2000
2200
2400
2600
2800
3000
3200
3400
3600
3800
m/z
start
Captures
Data
MassLynx - ProteinLy...
Chromatogram - [Spo...
Spectrum - [Spot_88]
86 - Paint

Figure S1.58

Mascot Search Results Spot 89

```
User            : Paul Millares
Email           : paul.millares@gmail.com
Search title    : Spot 89
Database        : Haemonchus 210108 (6387 sequences; 918038 residues)
Timestamp       : 1 Aug 2011 at 11:02:17 GMT
Top Score       : 106 for HCP01375_1, putative nuclear encoded protein  Method: similarity and extension
```

Mascot Score Histogram

Protein score is -10*Log(P), where P is the probability that the observed match is a random event.

Protein scores greater than 51 are significant (p<0.05).

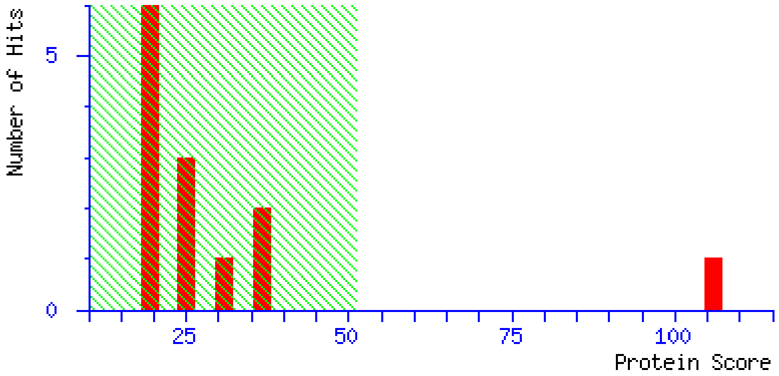

Concise Protein Summary Report

1. HCP01375_1 **Mass:** 19066 **Score:** 106 **Expect:** 1.6e-007 **Matches:** 11
   putative nuclear encoded protein Method: similarity and extension
   HCP05449_1 **Mass:** 7681 **Score:** 27 **Expect:** 12 **Matches:** 3
   putative nuclear encoded protein Method: ESTScan
   HCP13120_1 **Mass:** 9281 **Score:** 24 **Expect:** 26 **Matches:** 3
   putative nuclear encoded protein Method: ESTScan

HCP02277_1 **Mass:** 10160 **Score:** 22 **Expect:** 38 **Matches:** 4
putative nuclear encoded protein Method: ESTScan

HCP01129_2 **Mass:** 10160 **Score:** 22 **Expect:** 38 **Matches:** 4
putative nuclear encoded protein Method: ESTScan

HCP00185_1 **Mass:** 25557 **Score:** 22 **Expect:** 38 **Matches:** 4
putative nuclear encoded protein Method: similarity and extension

HCP02270_1 **Mass:** 6435 **Score:** 22 **Expect:** 39 **Matches:** 3
putative nuclear encoded protein Method: Longest ORF

HCP02640_1 **Mass:** 11355 **Score:** 22 **Expect:** 40 **Matches:** 3
putative nuclear encoded protein Method: ESTScan

HCP06506_1 **Mass:** 7964 **Score:** 22 **Expect:** 44 **Matches:** 3
putative nuclear encoded protein Method: ESTScan

HCP12420_1 **Mass:** 25303 **Score:** 22 **Expect:** 44 **Matches:** 4
putative nuclear encoded protein Method: similarity and extension

HCP01548_1 **Mass:** 24949 **Score:** 21 **Expect:** 46 **Matches:** 4
putative nuclear encoded protein Method: similarity and extension

HCP01399_1 **Mass:** 3479 **Score:** 21 **Expect:** 54 **Matches:** 2
putative nuclear encoded protein Method: ESTScan

HCP00570_2 **Mass:** 17065 **Score:** 20 **Expect:** 68 **Matches:** 4
putative nuclear encoded protein Method: similarity and extension

HCP07650_1 **Mass:** 15685 **Score:** 20 **Expect:** 72 **Matches:** 3
putative nuclear encoded protein Method: ESTScan

HCP00551_1 **Mass:** 4438 **Score:** 19 **Expect:** 73 **Matches:** 2
putative nuclear encoded protein Method: Longest ORF

HCP00290_1 **Mass:** 15656 **Score:** 19 **Expect:** 73 **Matches:** 3
putative nuclear encoded protein Method: similarity and extension

HCP00290_3 **Mass:** 15656 **Score:** 19 **Expect:** 73 **Matches:** 3
putative nuclear encoded protein Method: similarity and extension

HCP00290_2 **Mass:** 15684 **Score:** 19 **Expect:** 73 **Matches:** 3
putative nuclear encoded protein Method: similarity and extension

HCP01264_1 **Mass:** 14734 **Score:** 19 **Expect:** 79 **Matches:** 3
putative nuclear encoded protein Method: similarity and extension
HCP13058_1 **Mass:** 16736 **Score:** 19 **Expect:** 80 **Matches:** 3
putative nuclear encoded protein Method: ESTScan
HCP03349_1 **Mass:** 16141 **Score:** 19 **Expect:** 82 **Matches:** 3
putative nuclear encoded protein Method: similarity and extension
HCP03349_2 **Mass:** 16141 **Score:** 19 **Expect:** 82 **Matches:** 3
putative nuclear encoded protein Method: similarity and extension

---

2. HCP02978_1 **Mass:** 21635 **Score:** 36 **Expect:** 1.6 **Matches:** 6
putative nuclear encoded protein Method: similarity and extension
HCP10318_1 **Mass:** 21866 **Score:** 26 **Expect:** 18 **Matches:** 5
putative nuclear encoded protein Method: similarity and extension
HCP02978_3 **Mass:** 21993 **Score:** 26 **Expect:** 18 **Matches:** 5
putative nuclear encoded protein Method: similarity and extension
HCP10318_3 **Mass:** 23802 **Score:** 25 **Expect:** 22 **Matches:** 5
putative nuclear encoded protein Method: similarity and extension
HCP04900_1 **Mass:** 24991 **Score:** 21 **Expect:** 56 **Matches:** 4
putative nuclear encoded protein Method: similarity and extension
HCP00710_3 **Mass:** 17805 **Score:** 20 **Expect:** 65 **Matches:** 4
putative nuclear encoded protein Method: similarity and extension
HCP00710_2 **Mass:** 17805 **Score:** 20 **Expect:** 65 **Matches:** 4
putative nuclear encoded protein Method: similarity and extension
HCP10832_1 **Mass:** 14856 **Score:** 20 **Expect:** 67 **Matches:** 3
putative nuclear encoded protein Method: similarity and extension
HCP00018_3 **Mass:** 37748 **Score:** 19 **Expect:** 79 **Matches:** 5
putative nuclear encoded protein Method: similarity and extension

---

Search Parameters

Type of search : Peptide Mass Fingerprint

Enzyme : Trypsin
Variable modifications : Carbamidomethyl (C),Glu->pyro-Glu (N-term E),Oxidation (M)
Mass values : Monoisotopic
Protein Mass : Unrestricted
Peptide Mass Tolerance : ± 1.2 Da
Peptide Charge State : 1+
Max Missed Cleavages : 1
Number of queries : 13

Protein View

Match to: **HCP01375_1** Score: **106** Expect: **1.6e-007**
putative nuclear encoded protein Method: similarity and extension

Nominal mass (M_r): **19066**; Calculated pI value: **6.45**
NCBI BLAST search of HCP01375_1 against nr
Unformatted sequence string for pasting into other applications

Variable modifications: Carbamidomethyl (C),Glu->pyro-Glu (N-term E),Oxidation (M)
Cleavage by Trypsin: cuts C-term side of KR unless next residue is P
Number of mass values searched: **13**
Number of mass values matched: **11**
Sequence Coverage: **45%**

Matched peptides shown in **Bold Red**

```
  1 MTGLLFLLFV LGAPFAVNAK ELPEKFYGKF DLDHSENFDE YLEAKGYGWF
 51 TRKLVTFATF KKEFKKSDKR GKFDYANLTS KKNVFYDNVE LGKEFVGEGL
101 DSTKHKIVFD LVGDVMFEKH HPIEEGEAKD ETYEYSFITK DGKEYLLVKM
151 EANGVIGKRF YKRV
```

Start - End	Observed	Mr(expt)	Mr(calc)	Delta	Miss	Sequence	
54 - 61	926.65	925.65	925.53	0.12	0	K.LVTFATFK.K	
54 - 62	1054.74	1053.73	1053.62	0.11	1	K.LVTFATFKK.E	
71 - 81	1244.00	1243.00	1242.62	0.37	1	R.GKFDYANLTSK.K	
82 - 93	1426.07	1425.06	1424.73	0.33	1	K.KNVFYDNVELGK.E	
83 - 93	1297.99	1296.99	1296.64	0.35	0	K.NVFYDNVELGK.E	
94 - 104	1181.97	1180.96	1180.56	0.40	0	K.EFVGEGLDSTK.H	
120 - 129	1146.93	1145.92	1145.55	0.37	0	K.HHPIEEGEAK.D	
130 - 140	1395.96	1394.95	1394.62	0.33	0	K.DETYEYSFITK.D	
150 - 158	918.55	917.55	917.46	0.08	0	K.MEANGVIGK.R	
150 - 158	934.61	933.60	933.46	0.14	0	K.MEANGVIGK.R	Oxidation (M)

**150 - 159**	**1090.74**	**1089.74**	**1089.56**	**0.18**	**1**	**K.MEANGVIGKR.F**	Oxidation (M)

No match to: 902.55, 1345.03

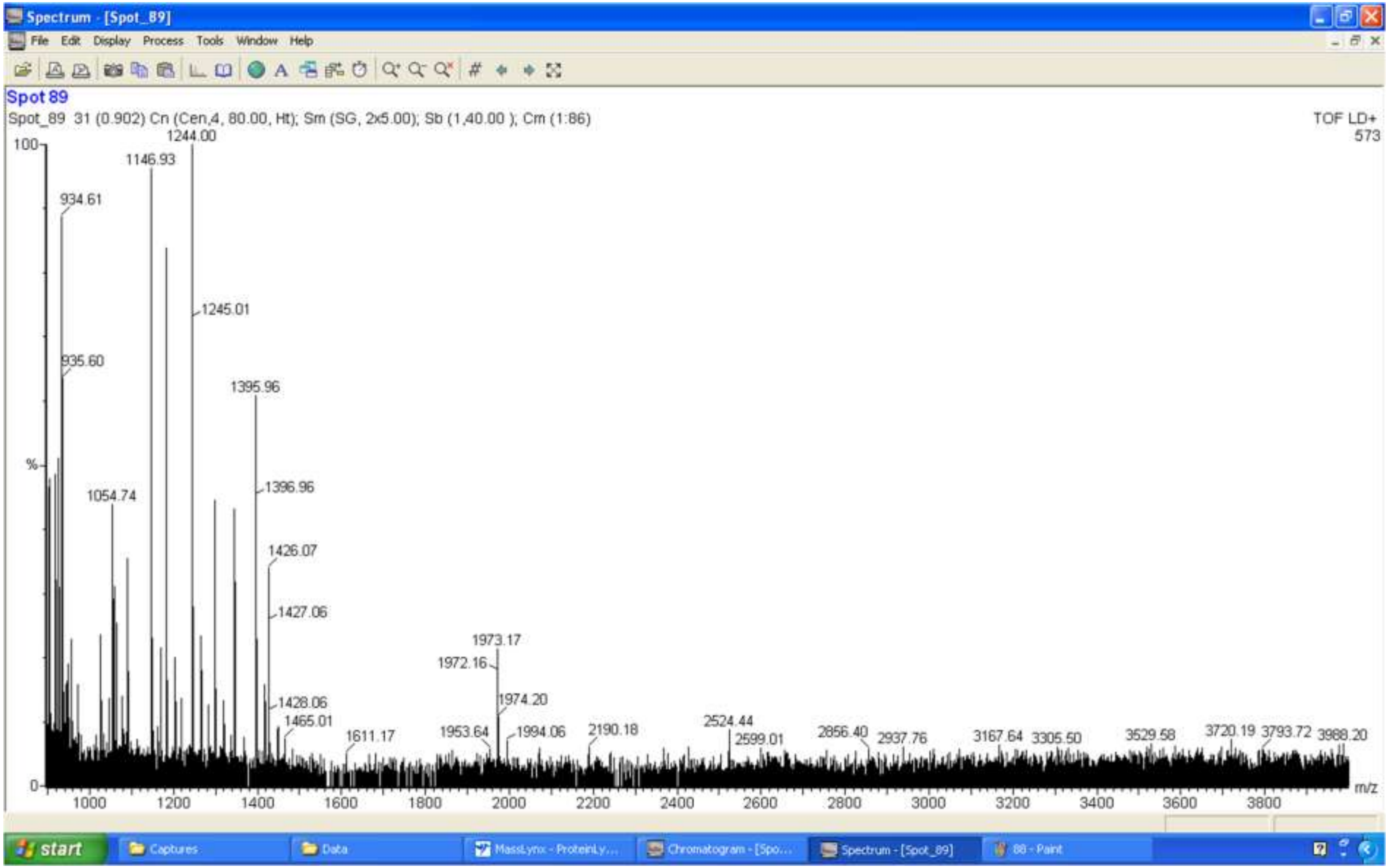

Spectrum - [Spot_89]
File Edit Display Process Tools Window Help
Spot 89
Spot_89 31 (0.902) Cn (Cen,4, 80.00, Ht); Sm (SG, 2x5.00); Sb (1,40.00); Cm (1:86)
TOF LD+
573
100
%
0
1244.00
1146.93
934.61
1245.01
935.60
1395.96
1054.74
1396.96
1426.07
1427.06
1973.17
1972.16
1974.20
1428.06
1465.01
1611.17
1953.64
1994.06
2190.18
2524.44
2599.01
2856.40
2937.76
3167.64
3305.50
3529.58
3720.19
3793.72
3988.20
m/z
1000
1200
1400
1600
1800
2000
2200
2400
2600
2800
3000
3200
3400
3600
3800
start
Captures
Data
MassLynx - ProteinLy...
Chromatogram - [Spo...
Spectrum - [Spot_89]
88 - Paint

Figure S1.59

Mascot Search Results Spot 90

```
User            : Paul Millares
Email           : paul.millares@gmail.com
Search title    : Spot 90
Database        : Haemonchus 210108 (6387 sequences; 918038 residues)
Timestamp       : 1 Aug 2011 at 11:03:18 GMT
Top Score       : 46 for HCP00907_1, putative nuclear encoded protein  Method: ESTScan
```

Mascot Score Histogram

Protein score is -10*Log(P), where P is the probability that the observed match is a random event.

Protein scores greater than 51 are significant (p<0.05).

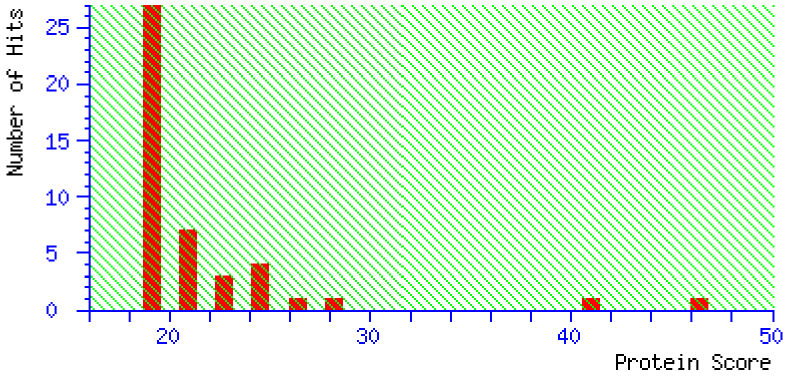

Concise Protein Summary Report

1. HCP00907_1 **Mass:** 15063 **Score:** 46 **Expect:** 0.15 **Matches:** 17
   putative nuclear encoded protein Method: ESTScan
   HCP04027_2 **Mass:** 15063 **Score:** 46 **Expect:** 0.15 **Matches:** 17
   putative nuclear encoded protein Method: ESTScan
   HCP04027_3 **Mass:** 15644 **Score:** 34 **Expect:** 2.5 **Matches:** 16
   putative nuclear encoded protein Method: ESTScan

Search Parameters

Type of search : Peptide Mass Fingerprint
Enzyme : Trypsin
Variable modifications : Carbamidomethyl (C),Glu->pyro-Glu (N-term E),Oxidation (M)
Mass values : Monoisotopic
Protein Mass : Unrestricted
Peptide Mass Tolerance : ± 1.2 Da
Peptide Charge State : 1+
Max Missed Cleavages : 1
Number of queries : 71

Protein View

Match to: **HCP00907_1** Score: **46** Expect: **0.15**
putative nuclear encoded protein Method: ESTScan

Nominal mass (M_r): **15063**; Calculated pI value: **8.49**
NCBI BLAST search of HCP00907_1 against nr
Unformatted sequence string for pasting into other applications

Variable modifications: Carbamidomethyl (C),Glu->pyro-Glu (N-term E),Oxidation (M)
Cleavage by Trypsin: cuts C-term side of KR unless next residue is P
Number of mass values searched: **71**
Number of mass values matched: **17**
Sequence Coverage: **73%**

Matched peptides shown in **Bold Red**

```
  1 GTRITTMKPD EARAALKPHY EALLKNMNEG KFEENFKHFH PHCAVVHRGK
 51 GAYYGKEQIG AMLKKLFEEQ HPKNIKRTNE VYCGCECCIC VSFDITFDSP
101 KGAKKVSEQH IWKKHENDWK LYHAEYDMN
```

Start - End	Observed	Mr(expt)	Mr(calc)	Delta	Miss	Sequence
1 - 13	1476.03	1475.02	1474.76	0.26	1	-.GTRITTMKPDEAR.A
14 - 25	1354.09	1353.08	1352.78	0.30	0	R.AALKPHYEALLK.N
14 - 31	2027.28	2026.27	2026.07	0.21	1	R.AALKPHYEALLKNMNEGK.F
26 - 37	1502.94	1501.93	1501.65	0.28	1	K.NMNEGKFEENFK.H Oxidation (M)
38 - 48	1339.96	1338.95	1338.65	0.30	0	K.HFHPHCAVVHR.G
38 - 48	1396.98	1395.97	1395.67	0.30	0	K.HFHPHCAVVHR.G Carbamidomethyl (C)

51 - 64	1545.06	1544.05	1543.77	0.28	1	K.GAYYGKEQIGAMLK.K Oxidation (M)
57 - 64	905.57	904.56	904.47	0.09	0	K.EQIGAMLK.K Oxidation (M)
57 - 65	1018.63	1017.62	1016.57	1.05	1	K.EQIGAMLKK.L
57 - 65	1034.66	1033.65	1032.56	1.09	1	K.EQIGAMLKK.L Oxidation (M)
65 - 73	1155.97	1154.96	1154.61	0.36	1	K.KLFEEQHPK.N
66 - 73	1027.62	1026.62	1026.51	0.10	0	K.LFEEQHPK.N
106 - 113	1026.63	1025.62	1025.53	0.09	0	K.VSEQHIWK.K
106 - 114	1154.98	1153.97	1153.62	0.35	1	K.VSEQHIWKK.H
114 - 120	957.56	956.55	955.45	1.10	1	K.KHENDWK.L
115 - 129	1981.15	1980.14	1979.81	0.33	1	K.HENDWKLYHAEYDMN.- Oxidation (M)
121 - 129	1171.84	1170.83	1170.47	0.37	0	K.LYHAEYDMN.- Oxidation (M)

No match to: 900.37, 911.59, 927.56, 949.52, 951.39, 970.60, 988.56, 992.58, 1010.55, 1041.62, 1046.65, 1049.60, 1053.66, 1059.63, 1071.58, 1075.64, 1112.82, 1165.93, 1179.96, 1186.99, 1193.84, 1208.96, 1210.96, 1255.94, 1273.00, 1305.99, 1376.07, 1383.98, 1387.01, 1394.96, 1408.98, 1410.99, 1418.96, 1458.05, 1477.04, 1489.02, 1495.04, 1517.03, 1556.16, 1578.16, 1684.25, 1692.15, 1706.22, 1714.13, 1736.11, 1758.10, 1992.34, 2019.30, 2036.31, 2051.28, 2058.29, 2080.27, 2156.35, 2211.33

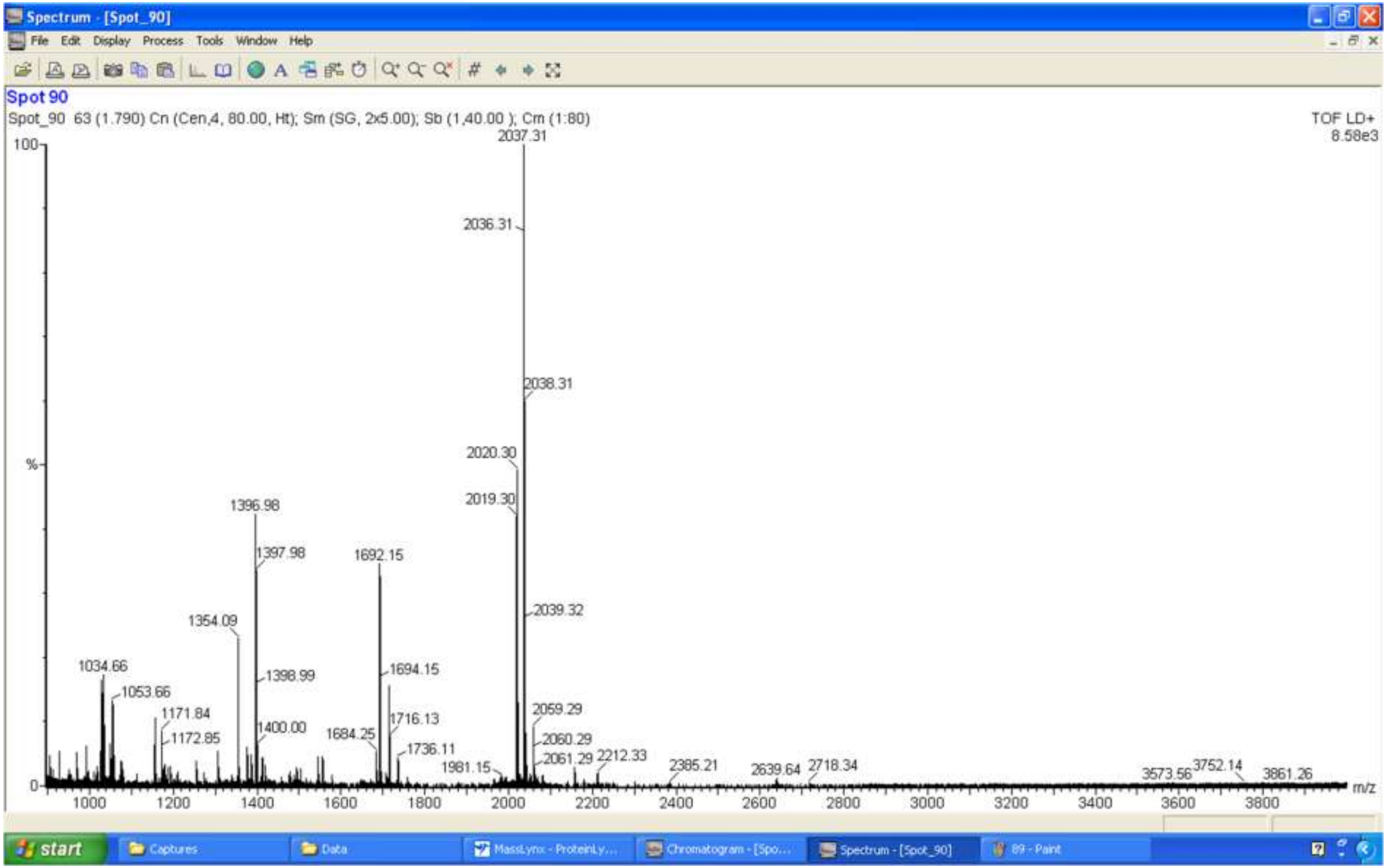
Spectrum - [Spot_90]
File Edit Display Process Tools Window Help
Spot 90
Spot_90 63 (1.790) Cn (Cen,4, 80.00, Ht); Sm (SG, 2x5.00); Sb (1,40.00); Cm (1:80)
TOF LD+
8.58e3
100
%
0
2037.31
2036.31
2038.31
2020.30
2019.30
2039.32
1396.98
1397.98
1692.15
1354.09
1034.66
1053.66
1171.84
1172.85
1398.99
1400.00
1694.15
1716.13
1684.25
1736.11
1981.15
2059.29
2060.29
2061.29
2212.33
2385.21
2639.64
2718.34
3573.56
3752.14
3861.26
1000 1200 1400 1600 1800 2000 2200 2400 2600 2800 3000 3200 3400 3600 3800
m/z
start
Captures
Data
MassLynx - ProteinLy...
Chromatogram - [Spo...
Spectrum - [Spot_90]
89 - Paint

Figure S1.60

MATRIX SCIENCE Mascot Search Results Spot 91

User : **Paul Millares**
Email : **paul.millares@gmail.com**
Search title : **Spot 91**
Database : **Haemonchus 210108 (6387 sequences; 918038 residues)**
Timestamp : **1 Aug 2011 at 11:03:37 GMT**
Top Score : **68 for HCP03264_1, putative nuclear encoded protein Method: similarity and extension**

Mascot Score Histogram

Protein score is -10*Log(P), where P is the probability that the observed match is a random event.

Protein scores greater than 51 are significant (p<0.05).

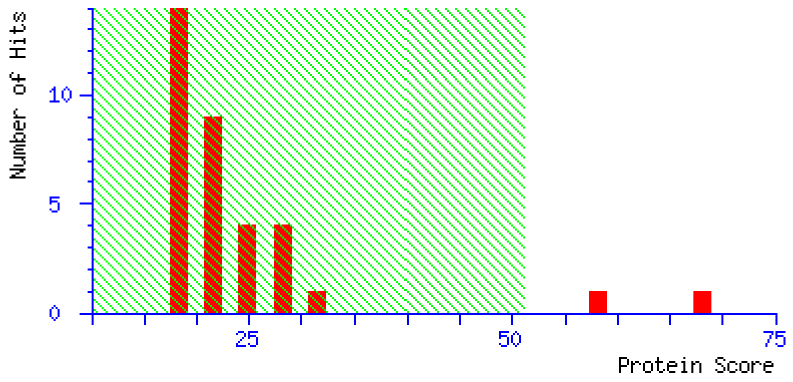

Concise Protein Summary Report

1. HCP03264_1 **Mass:** 9282 **Score:** **68** **Expect:** 0.00099 **Matches:** 6
   putative nuclear encoded protein Method: similarity and extension
   HCP03264_2 **Mass:** 11118 **Score:** **64** **Expect:** 0.0025 **Matches:** 6
   putative nuclear encoded protein Method: similarity and extension
   HCP00372_3 **Mass:** 15990 **Score:** **56** **Expect:** 0.018 **Matches:** 6
   putative nuclear encoded protein Method: similarity and extension

HCP04660_2 **Mass:** 13124 **Score:** 32 **Expect:** 3.7 **Matches:** 4
putative nuclear encoded protein Method: ESTScan
HCP07979_1 **Mass:** 12516 **Score:** 22 **Expect:** 43 **Matches:** 3
putative nuclear encoded protein Method: ESTScan
HCP04660_1 **Mass:** 12910 **Score:** 21 **Expect:** 50 **Matches:** 3
putative nuclear encoded protein Method: ESTScan
HCP08170_1 **Mass:** 16110 **Score:** 19 **Expect:** 75 **Matches:** 3
putative nuclear encoded protein Method: similarity and extension
HCP08170_2 **Mass:** 16110 **Score:** 19 **Expect:** 75 **Matches:** 3
putative nuclear encoded protein Method: similarity and extension

---

2. HCP00372_1 **Mass:** 24748 **Score:** 58 **Expect:** 0.011 **Matches:** 7
putative nuclear encoded protein Method: similarity and extension
HCP00372_2 **Mass:** 25560 **Score:** 57 **Expect:** 0.014 **Matches:** 7
putative nuclear encoded protein Method: similarity and extension

---

3. HCP03101_2 **Mass:** 24755 **Score:** 32 **Expect:** 3.8 **Matches:** 5
putative nuclear encoded protein Method: similarity and extension
HCP03101_1 **Mass:** 22107 **Score:** 24 **Expect:** 24 **Matches:** 4
putative nuclear encoded protein Method: similarity and extension
HCP02233_1 **Mass:** 23081 **Score:** 21 **Expect:** 56 **Matches:** 4
putative nuclear encoded protein Method: similarity and extension

---

Search Parameters

Type of search : Peptide Mass Fingerprint
Enzyme : Trypsin
Variable modifications : Carbamidomethyl (C),Glu->pyro-Glu (N-term E),Oxidation (M)
Mass values : Monoisotopic
Protein Mass : Unrestricted
Peptide Mass Tolerance : ± 1.2 Da
Peptide Charge State : 1+
Max Missed Cleavages : 1

Number of queries : 13

Protein View

Match to: **HCP03264_1** Score: **68** Expect: **0.00099**

putative nuclear encoded protein Method: similarity and extension

Nominal mass (M_r): **9282**; Calculated pI value: **9.99**

NCBI BLAST search of HCP03264_1 against nr

Unformatted sequence string for pasting into other applications

Variable modifications: Carbamidomethyl (C),Glu->pyro-Glu (N-term E),Oxidation (M)

Cleavage by Trypsin: cuts C-term side of KR unless next residue is P

Number of mass values searched: **13**

Number of mass values matched: **6**

Sequence Coverage: **75%**

Matched peptides shown in **Bold Red**

1 RKRSLERMAQ VTR**QIIHSAN APGAVGPYSQ AVRVDNTIYI SGSLGLDPK**S

51 GDLK**QGIKEQ THQSLKNIGE ILKAAGVGYG NVVK**TTV

Start - End	Observed	Mr(expt)	Mr(calc)	Delta	Miss	Sequence
14 - 33	2036.32	2035.31	2035.06	0.25	0	R.QIIHSANAPGAVGPYSQAVR.V
34 - 49	1692.16	1691.15	1690.88	0.27	0	R.VDNTIYISGSLGLDPK.S
55 - 66	1396.98	1395.97	1395.75	0.23	1	K.QGIKEQTHQSLK.N
59 - 66	970.61	969.60	969.49	0.11	0	K.EQTHQSLK.N
59 - 73	1737.12	1736.11	1736.94	-0.83	1	K.EQTHQSLKNIGEILK.A
74 - 84	1034.66	1033.66	1033.56	0.10	0	K.AAGVGYGNVVK.T

No match to: 992.59, 1031.69, 1053.68, 1354.08, 1714.14, 2019.30, 2058.30

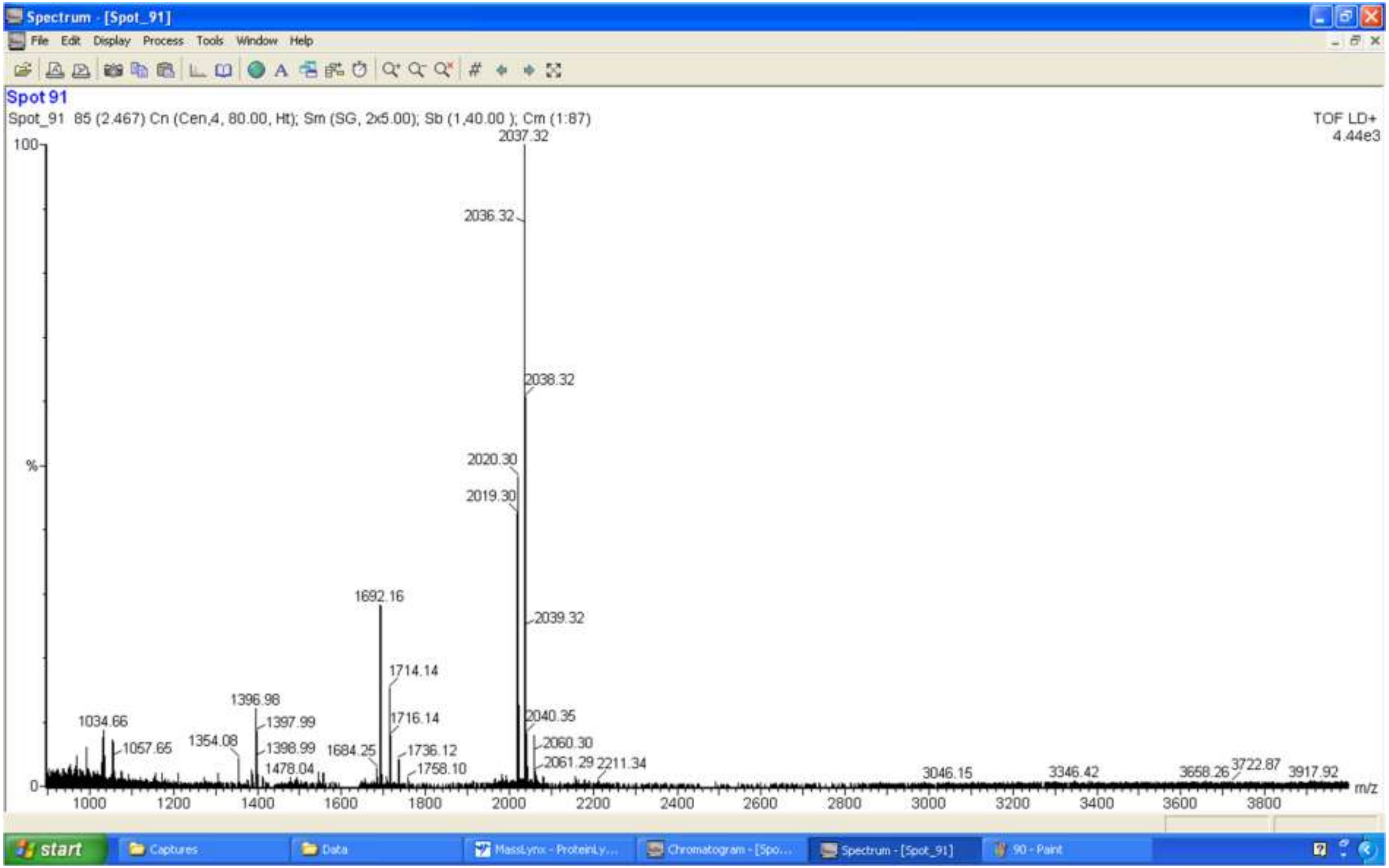

Spectrum - [Spot_91]
File Edit Display Process Tools Window Help
Spot 91
Spot_91 85 (2.467) Cn (Cen,4, 80.00, Ht); Sm (SG, 2x5.00); Sb (1,40.00); Cm (1:87)
TOF LD+
4.44e3
2037.32
2036.32
2038.32
2020.30
2019.30
1692.16
2039.32
1714.14
1396.98
1034.66
1397.99
1716.14
2040.35
1354.08
1057.65
1398.99
1684.25
1736.12
2060.30
1478.04
1758.10
2061.29
2211.34
3046.15
3346.42
3658.26
3722.87
3917.92
100
%
0
1000 1200 1400 1600 1800 2000 2200 2400 2600 2800 3000 3200 3400 3600 3800
m/z
start
Captures
Data
MassLynx - ProteinLy...
Chromatogram - [Spo...
Spectrum - [Spot_91]
90 - Paint

Figure S1.61

Mascot Search Results Spot 92

```
User            : Paul Millares
Email           : paul.millares@gmail.com
Search title    : Spot 92
Database        : Haemonchus 210108 (6387 sequences; 918038 residues)
Timestamp       : 1 Aug 2011 at 11:04:02 GMT
Top Score       : 53 for HCP00372_1, putative nuclear encoded protein  Method: similarity and extension
```

Mascot Score Histogram

Protein score is -10*Log(P), where P is the probability that the observed match is a random event.

Protein scores greater than 51 are significant (p<0.05).

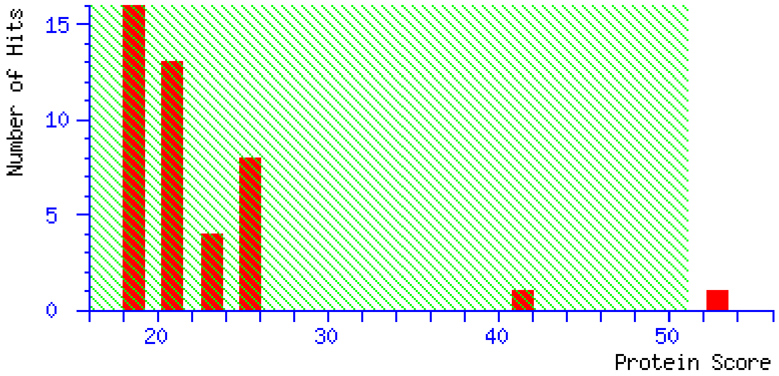

Concise Protein Summary Report

1. HCP00372_1 **Mass:** 24748 **Score:** 53 **Expect:** 0.034 **Matches:** 12
putative nuclear encoded protein Method: similarity and extension
HCP03264_2 **Mass:** 11118 **Score:** 43 **Expect:** 0.33 **Matches:** 8
putative nuclear encoded protein Method: similarity and extension
HCP03264_1 **Mass:** 9282 **Score:** 36 **Expect:** 1.5 **Matches:** 7
putative nuclear encoded protein Method: similarity and extension

HCP00372_2 **Mass:** 25560 **Score:** 36 **Expect:** 1.6 **Matches:** 10
putative nuclear encoded protein Method: similarity and extension
HCP00372_3 **Mass:** 15990 **Score:** 26 **Expect:** 17 **Matches:** 7
putative nuclear encoded protein Method: similarity and extension

---

2. HCP02352_1 **Mass:** 17007 **Score:** 41 **Expect:** 0.54 **Matches:** 10
putative nuclear encoded protein Method: ESTScan

Search Parameters

Type of search : Peptide Mass Fingerprint
Enzyme : Trypsin
Variable modifications : Carbamidomethyl (C),Glu->pyro-Glu (N-term E),Oxidation (M)
Mass values : Monoisotopic
Protein Mass : Unrestricted
Peptide Mass Tolerance : ± 1.2 Da
Peptide Charge State : 1+
Max Missed Cleavages : 1
Number of queries : 41

Protein View

Match to: **HCP00372_1** Score: **53** Expect: **0.034**
putative nuclear encoded protein Method: similarity and extension

Nominal mass (M_r): **24748**; Calculated pI value: **7.82**
NCBI BLAST search of HCP00372_1 against nr
Unformatted sequence string for pasting into other applications

Variable modifications: Carbamidomethyl (C),Glu->pyro-Glu (N-term E),Oxidation (M)
Cleavage by Trypsin: cuts C-term side of KR unless next residue is P
Number of mass values searched: **41**
Number of mass values matched: **12**
Sequence Coverage: **52%**

Matched peptides shown in **Bold Red**

1 RKSDEALDDS APDGKKRSLE RMAQVTR**QII HSANAPGAVG PYSQAVRVDN**
51 **TIYISGSLGL DPKSGDLKQG IKEQTHQSLK** NIGEILK**AAG VGYGNVVKTT**

101 **VLLADINDFT TVNDIYKEYF TAKFPARAAY QVAALPKKAL VEIEAIAVTG**
151 **EIK**DIXTAST FVVRDYTSLL LERLCGISLH AYDSAITYYA NWYRCILLGV
201 LLFAIDVLFL FCLSERLISK DLREK

Start - End	Observed	Mr(expt)	Mr(calc)	Delta	Miss	Sequence
28 - 47	2036.42	2035.41	2035.06	0.35	0	R.QIIHSANAPGAVGPYSQAVR.V
48 - 63	1692.25	1691.24	1690.88	0.36	0	R.VDNTIYISGSLGLDPK.S
64 - 72	945.43	944.42	944.53	-0.11	1	K.SGDLKQGIK.E
69 - 80	1397.05	1396.04	1395.75	0.30	1	K.QGIKEQTHQSLK.N
73 - 80	951.42	950.41	951.48	-1.06	0	K.EQTHQSLK.N Glu->pyro-Glu (N-term E)
73 - 80	970.64	969.63	969.49	0.14	0	K.EQTHQSLK.N
88 - 98	1033.72	1032.71	1033.56	-0.84	0	K.AAGVGYGNVVK.T
99 - 117	2156.45	2155.44	2155.10	0.34	0	K.TTVLLADINDFTTVNDIYK.E
118 - 127	1211.01	1210.00	1210.61	-0.61	1	K.EYFTAKFPAR.A Glu->pyro-Glu (N-term E)
128 - 137	1031.74	1030.73	1030.58	0.15	0	R.AAYQVAALPK.K
138 - 153	1684.34	1683.33	1682.98	0.35	1	K.KALVEIEAIAVTGEIK.D
139 - 153	1556.26	1555.25	1554.89	0.37	0	K.ALVEIEAIAVTGEIK.D

No match to: 907.42, 925.43, 997.45, 1019.19, 1027.68, 1053.72, 1055.69, 1077.35, 1155.05, 1171.91, 1273.07, 1306.06, 1354.16, 1376.14, 1387.09, 1387.09, 1411.06, 1458.13, 1477.10, 1491.08, 1495.12, 1545.14, 1578.20, 1714.22, 1912.40, 1989.41, 2019.39, 2058.40, 2211.47

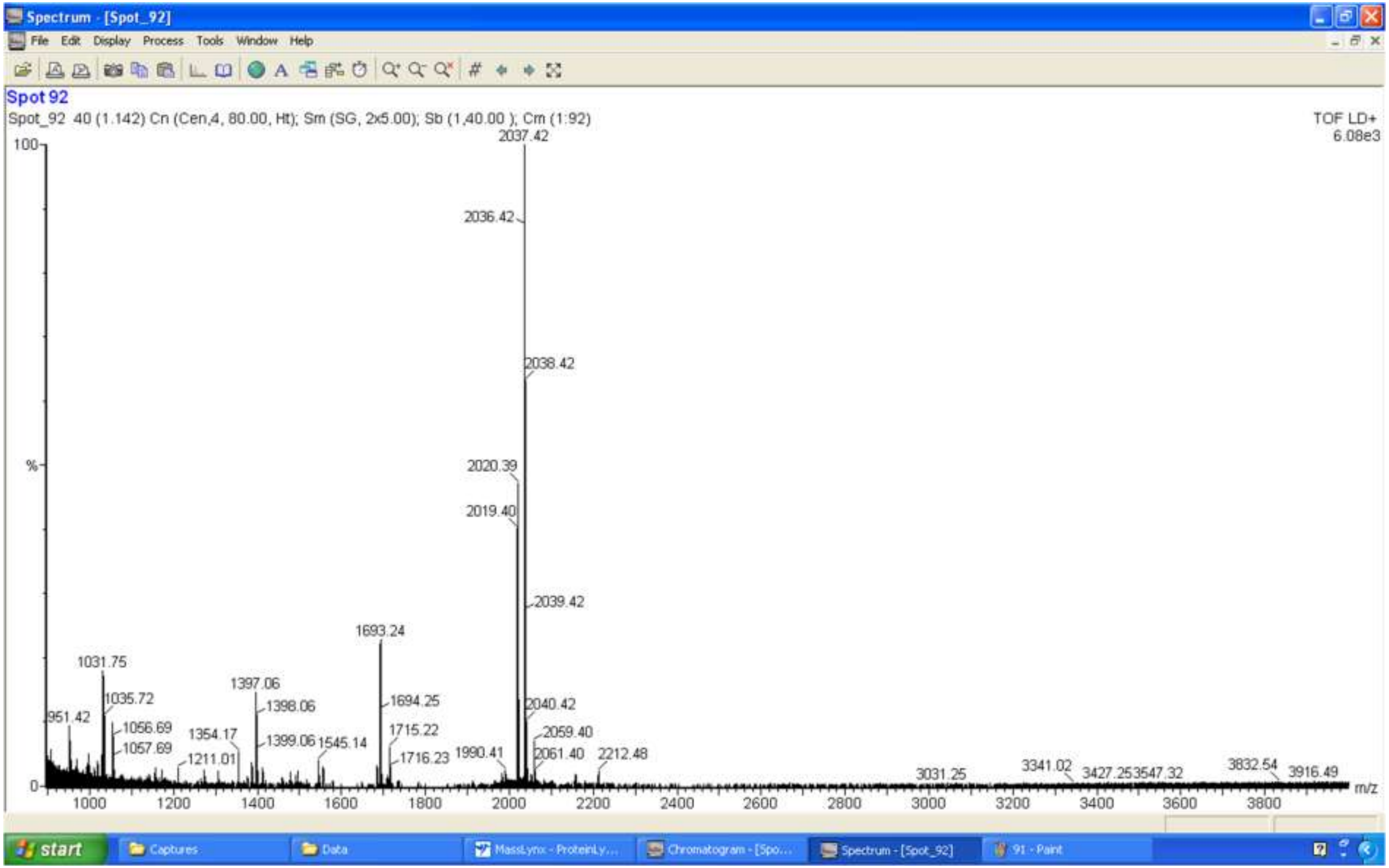

Spectrum - [Spot_92]
File Edit Display Process Tools Window Help
Spot 92
Spot_92 40 (1.142) Cn (Cen,4, 80.00, Ht); Sm (SG, 2x5.00); Sb (1,40.00); Cm (1:92)
TOF LD+
6.08e3
2037.42
2036.42
2038.42
2020.39
2019.40
2039.42
1693.24
1031.75
1397.06
1035.72
1398.06
1694.25
2040.42
951.42
1056.69
1354.17
1057.69
1399.06
1545.14
1715.22
2059.40
1211.01
1716.23
1990.41
2061.40
2212.48
3031.25
3341.02
3427.25
3547.32
3832.54
3916.49
100
%
0
1000 1200 1400 1600 1800 2000 2200 2400 2600 2800 3000 3200 3400 3600 3800
m/z
start
Captures
Data
MassLynx - ProteinLy...
Chromatogram - [Spo...
Spectrum - [Spot_92]
91 - Paint

Figure S1.62

Mascot Search Results Spot 93

```
User          : Paul Millares
Email         : paul.millares@gmail.com
Search title  : Spot 93
Database      : Haemonchus 210108 (6387 sequences; 918038 residues)
Timestamp     : 1 Aug 2011 at 11:04:29 GMT
Top Score     : 36 for HCP03264_2, putative nuclear encoded protein  Method: similarity and extension
```

Mascot Score Histogram

Protein score is -10*Log(P), where P is the probability that the observed match is a random event.

Protein scores greater than 51 are significant (p<0.05).

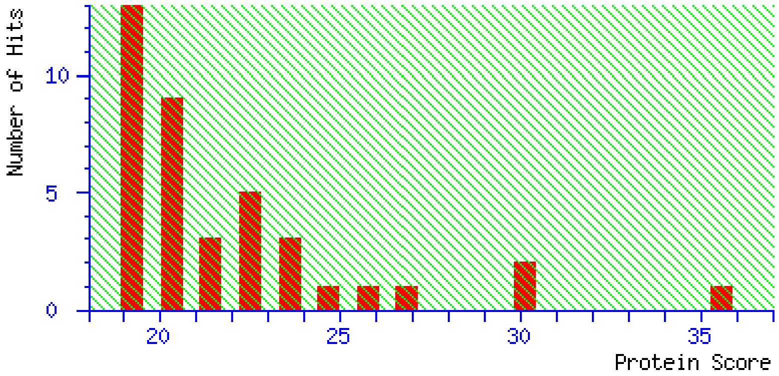

Concise Protein Summary Report

1. HCP03264_2 **Mass:** 11118 **Score:** 36 **Expect:** 1.8 **Matches:** 7
   putative nuclear encoded protein Method: similarity and extension
   HCP03264_1 **Mass:** 9282 **Score:** 29 **Expect:** 7.9 **Matches:** 6
   putative nuclear encoded protein Method: similarity and extension
   HCP00372_3 **Mass:** 15990 **Score:** 21 **Expect:** 54 **Matches:** 6
   putative nuclear encoded protein Method: similarity and extension

Search Parameters

Type of search : Peptide Mass Fingerprint
Enzyme : Trypsin
Variable modifications : Carbamidomethyl (C),Glu->pyro-Glu (N-term E),Oxidation (M)
Mass values : Monoisotopic
Protein Mass : Unrestricted
Peptide Mass Tolerance : ± 1.2 Da
Peptide Charge State : 1+
Max Missed Cleavages : 1
Number of queries : 40

Protein View

Match to: **HCP03264_2** Score: **36** Expect: **1.8**
putative nuclear encoded protein Method: similarity and extension

Nominal mass (M_r): **11118**; Calculated pI value: **9.52**
NCBI BLAST search of HCP03264_2 against nr
Unformatted sequence string for pasting into other applications

Variable modifications: Carbamidomethyl (C),Glu->pyro-Glu (N-term E),Oxidation (M)
Cleavage by Trypsin: cuts C-term side of KR unless next residue is P
Number of mass values searched: **40**
Number of mass values matched: **7**
Sequence Coverage: **75%**

Matched peptides shown in **Bold Red**

1 RKRSLERMAQ VTR**QIIHSAN APGAVGPYSQ AVRVDNTIYI SGSLGLDPK**S
51 GDLK**QGIKEQ THQSLK**NIGE ILK**AAGVGYG NVVKTTVLLA DINDFTTVND**
101 **IYK**

Start - End	Observed	Mr(expt)	Mr(calc)	Delta	Miss	Sequence
14 - 33	2036.41	2035.40	2035.06	0.34	0	R.QIIHSANAPGAVGPYSQAVR.V
34 - 49	1692.25	1691.24	1690.88	0.36	0	R.VDNTIYISGSLGLDPK.S
55 - 66	1397.05	1396.05	1395.75	0.30	1	K.QGIKEQTHQSLK.N
59 - 66	951.43	950.42	951.48	-1.06	0	K.EQTHQSLK.N Glu->pyro-Glu (N-term E)
59 - 66	969.45	968.44	969.49	-1.05	0	K.EQTHQSLK.N
74 - 84	1034.71	1033.70	1033.56	0.14	0	K.AAGVGYGNVVK.T

85 - 103 2156.46 2155.45 2155.10 0.34 0 K.TTVLLADINDFTTVNDIYK.-

No match to: 897.44, 900.42, 907.44, 914.43, 917.44, 925.43, 927.44, 935.43, 942.45, 943.43, 966.46, 985.45, 993.45, 995.45, 997.45, 1010.46, 1026.69, 1027.68, 1049.66, 1058.68, 1155.05, 1156.04, 1171.90, 1187.04, 1256.03, 1306.06, 1354.16, 1376.14, 1411.07, 1419.03, 1503.03, 2019.39, 2211.44

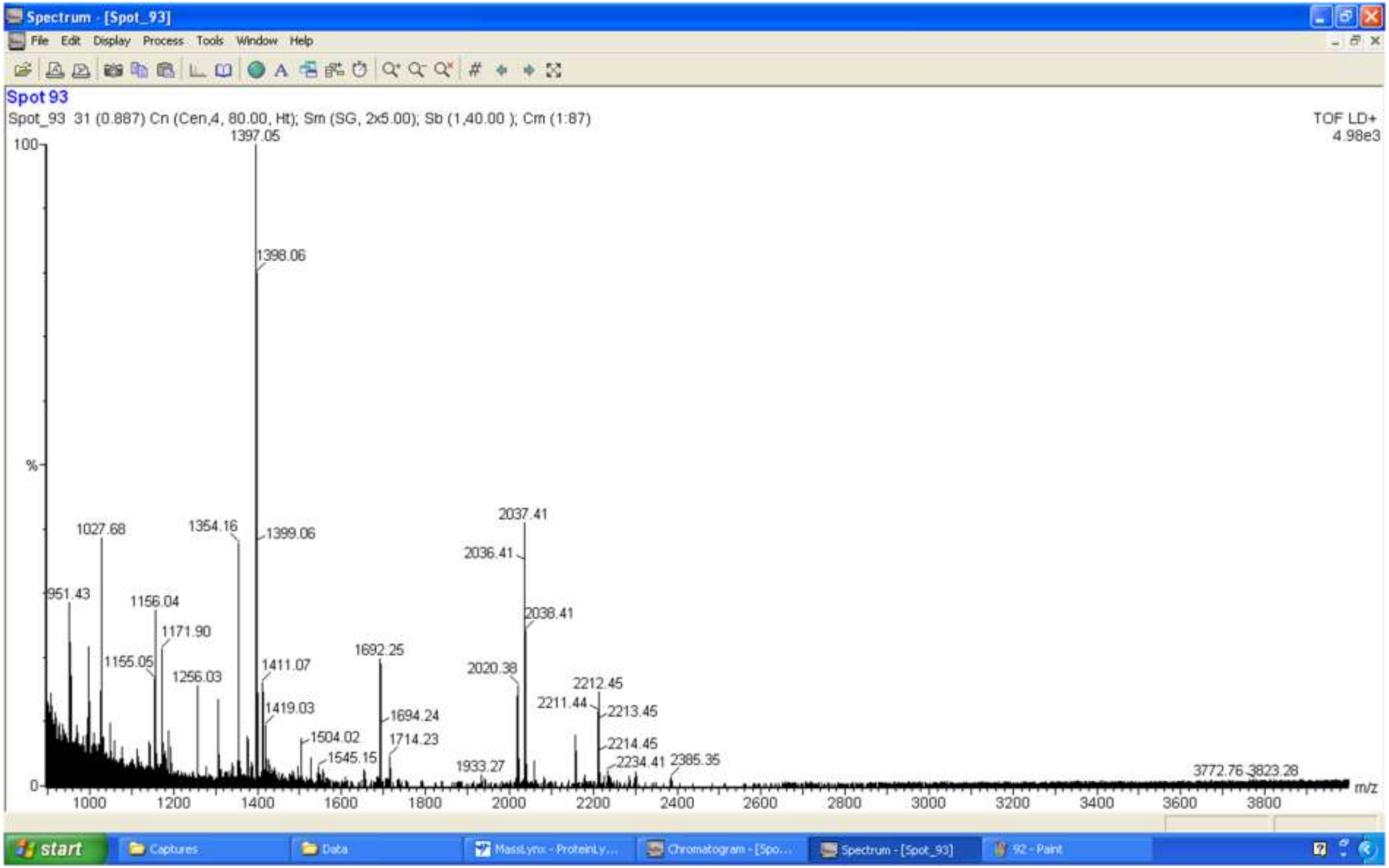
Spectrum - [Spot_93]
File Edit Display Process Tools Window Help
Spot 93
Spot_93 31 (0.887) Cn (Cen,4, 80.00, Ht); Sm (SG, 2x5.00); Sb (1,40.00); Cm (1:87)
TOF LD+
4.98e3
100
%
0
1397.05
1398.06
1399.06
1027.68
1354.16
951.43
1156.04
1171.90
1155.05
1256.03
1411.07
1419.03
1504.02
1545.15
1692.25
1694.24
1714.23
1933.27
2037.41
2036.41
2038.41
2020.38
2212.45
2211.44
2213.45
2214.45
2234.41
2385.35
3772.76
3823.28
m/z
1000
1200
1400
1600
1800
2000
2200
2400
2600
2800
3000
3200
3400
3600
3800
start
Captures
Data
MassLynx - ProteinLy...
Chromatogram - [Spo...
Spectrum - [Spot_93]
92 - Paint

Figure S1.63

Mascot Search Results Spot 94

User : Paul Millares
Email : paul.millares@gmail.com
Search title : Spot 94
Database : Haemonchus 210108 (6387 sequences; 918038 residues)
Timestamp : 1 Aug 2011 at 11:04:59 GMT
Top Score : 46 for HCP04833_1, putative nuclear encoded protein Method: similarity and extension

Mascot Score Histogram

Protein score is -10*Log(P), where P is the probability that the observed match is a random event.

Protein scores greater than 51 are significant (p<0.05).

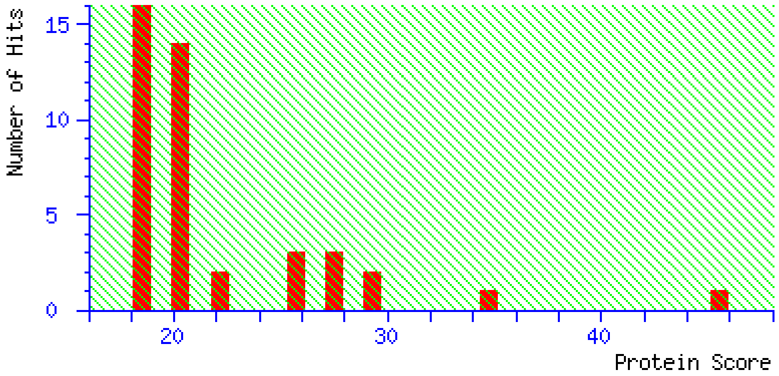

Concise Protein Summary Report

1. HCP04833_1 **Mass:** 17763 **Score:** 46 **Expect:** 0.18 **Matches:** 9
putative nuclear encoded protein Method: similarity and extension
HCP01640_1 **Mass:** 9672 **Score:** 19 **Expect:** 88 **Matches:** 3
putative nuclear encoded protein Method: similarity and extension

Search Parameters

Type of search : Peptide Mass Fingerprint
Enzyme : Trypsin
Variable modifications : Carbamidomethyl (C),Glu->pyro-Glu (N-term E),Oxidation (M)
Mass values : Monoisotopic
Protein Mass : Unrestricted
Peptide Mass Tolerance : ± 1.2 Da
Peptide Charge State : 1+
Max Missed Cleavages : 1
Number of queries : 22

Protein View

Match to: **HCP04833_1** Score: **46** Expect: **0.18**
putative nuclear encoded protein Method: similarity and extension

Nominal mass (M_r): **17763**; Calculated pI value: **9.55**
NCBI BLAST search of HCP04833_1 against nr
Unformatted sequence string for pasting into other applications

Variable modifications: Carbamidomethyl (C),Glu->pyro-Glu (N-term E),Oxidation (M)
Cleavage by Trypsin: cuts C-term side of KR unless next residue is P
Number of mass values searched: **22**
Number of mass values matched: **9**
Sequence Coverage: **50%**

Matched peptides shown in **Bold Red**

```
  1 MLITRTLTNA SRHCLDNYIL CQNSFFNGMR RRLHYGTIKK MPMVRVATNL
 51 PDKDVPANFE ERLTDLLAES MNKPRARIAV EMMAGQRIMH GGVRNPVVLI
101 KVESIGALDP DSTIRHTQRV TQLCTEVLHV PKDKVIISYF DLAPTNVGFP
151 GTTVAAATV
```

Start - End	Observed	Mr(expt)	Mr(calc)	Delta	Miss	Sequence
33 - 40	958.58	957.58	958.56	-0.98	1	R.LHYGTIKK.M
41 - 53	1487.08	1486.07	1486.76	-0.69	1	K.MPMVRVATNLPDK.D Oxidation (M)
46 - 62	1915.22	1914.22	1913.95	0.27	1	R.VATNLPDKDVPANFEER.L
54 - 62	1076.60	1075.59	1075.49	0.10	0	K.DVPANFEER.L
63 - 75	1504.08	1503.08	1502.78	0.30	0	R.LTDLLAESMNKPR.A Oxidation (M)
78 - 87	1121.82	1120.81	1120.54	0.27	0	R.IAVEMMAGQR.I Oxidation (M)
78 - 87	1137.86	1136.86	1136.53	0.33	0	R.IAVEMMAGQR.I 2 Oxidation (M)
102 - 115	1473.06	1472.06	1471.75	0.31	0	K.VESIGALDPDSTIR.H

120 - 132 1524.12 1523.11 1522.82 0.29 0 R.VTQLCTEVLHVPK.D Carbamidomethyl (C)

No match to: 900.38, 907.39, 951.38, 996.39, 1159.90, 1179.97, 1268.04, 1278.04, 1384.09, 1546.10, 1929.23, 1937.20, 2211.34

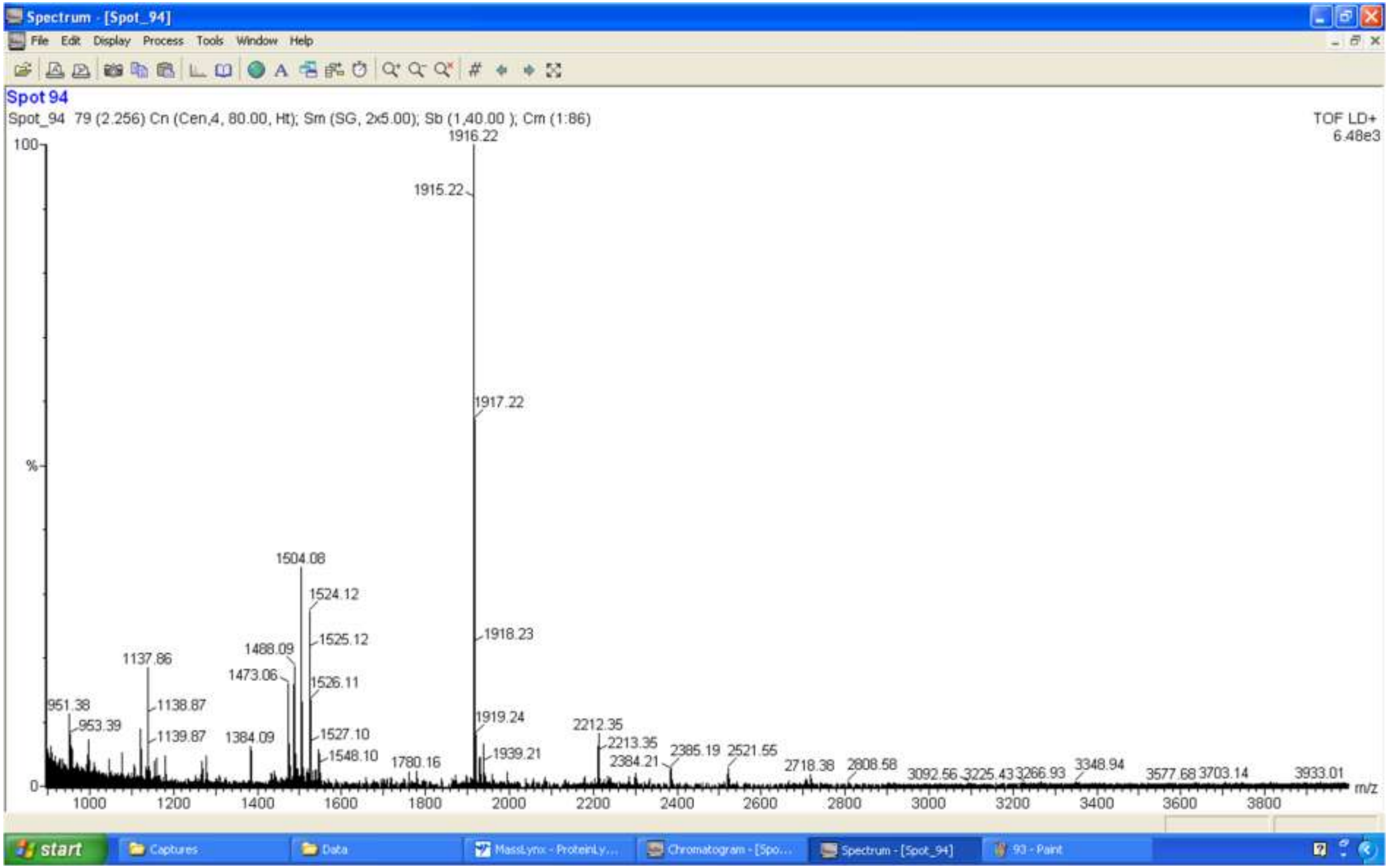

Figure S1.64

Mascot Search Results Spot 96

User : Paul Millares
Email : paul.millares@gmail.com
Search title : Spot 96
Database : Haemonchus 210108 (6387 sequences; 918038 residues)
Timestamp : 1 Aug 2011 at 11:05:27 GMT
Top Score : 41 for HCP00229_1, putative nuclear encoded protein Method: similarity and extension

Mascot Score Histogram

Protein score is -10*Log(P), where P is the probability that the observed match is a random event.

Protein scores greater than 51 are significant (p<0.05).

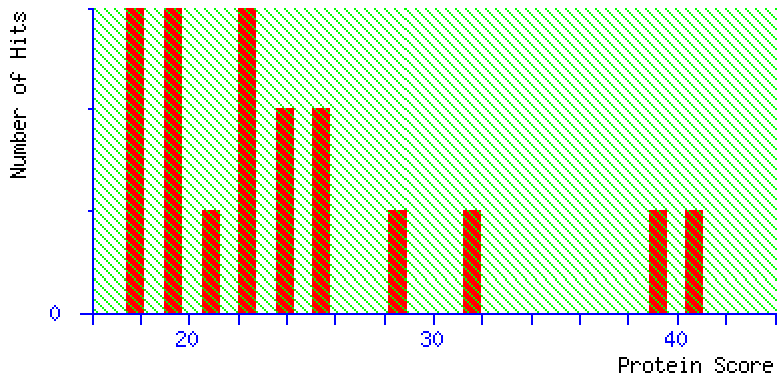

Concise Protein Summary Report

1. HCP00229_1 **Mass:** 10399 **Score:** 41 **Expect:** 0.54 **Matches:** 4
putative nuclear encoded protein Method: similarity and extension
HCP00229_2 **Mass:** 12055 **Score:** 38 **Expect:** 1 **Matches:** 4
putative nuclear encoded protein Method: similarity and extension
HCP11913_1 **Mass:** 4094 **Score:** 25 **Expect:** 23 **Matches:** 2
putative nuclear encoded protein Method: Longest ORF

HCP01161_1 **Mass:** 12876 **Score:** 20 **Expect:** 60 **Matches:** 3
putative nuclear encoded protein Method: similarity and extension
HCP01156_2 **Mass:** 16853 **Score:** 18 **Expect:** 94 **Matches:** 3
putative nuclear encoded protein Method: similarity and extension
HCP01107_1 **Mass:** 10286 **Score:** 18 **Expect:** 1.1e+002 **Matches:** 2
putative nuclear encoded protein Method: ESTScan
HCP06341_1 **Mass:** 23795 **Score:** 18 **Expect:** 1.1e+002 **Matches:** 3
putative nuclear encoded protein Method: similarity and extension

---

Search Parameters

Type of search : Peptide Mass Fingerprint
Enzyme : Trypsin
Variable modifications : Carbamidomethyl (C),Glu->pyro-Glu (N-term E),Oxidation (M)
Mass values : Monoisotopic
Protein Mass : Unrestricted
Peptide Mass Tolerance : ± 1.2 Da
Peptide Charge State : 1+
Max Missed Cleavages : 1
Number of queries : 8

Protein View

Match to: **HCP00229_1** Score: **41** Expect: **0.54**
putative nuclear encoded protein Method: similarity and extension

Nominal mass (M_r): **10399**; Calculated pI value: **7.03**
NCBI BLAST search of HCP00229_1 against nr
Unformatted sequence string for pasting into other applications

Variable modifications: Carbamidomethyl (C),Glu->pyro-Glu (N-term E),Oxidation (M)
Cleavage by Trypsin: cuts C-term side of KR unless next residue is P
Number of mass values searched: **8**
Number of mass values matched: **4**
Sequence Coverage: **47%**

Matched peptides shown in **Bold Red**

```
 1 KSFKPLHDRV LVERCAAETK TKGGIMLPEK SQGKVLEATV IAVGPGARNE
51 KGDLIPMCVK SGDHVLLPEY GGTKVVVDEK EYSIFREQDL LGVFH
```

Start - End	Observed	Mr(expt)	Mr(calc)	Delta	Miss	Sequence
2 - 9	999.60	998.60	998.53	0.07	0	K.SFKPLHDR.V
35 - 48	1353.05	1352.04	1351.78	0.26	0	K.VLEATVIAVGPGAR.N
61 - 74	1472.98	1471.97	1471.73	0.24	0	K.SGDHVLLPEYGGTK.V
87 - 95	1057.59	1056.58	1056.52	0.06	0	R.EQDLLGVFH.-

No match to: 948.53, 951.34, 968.56, 1404.98

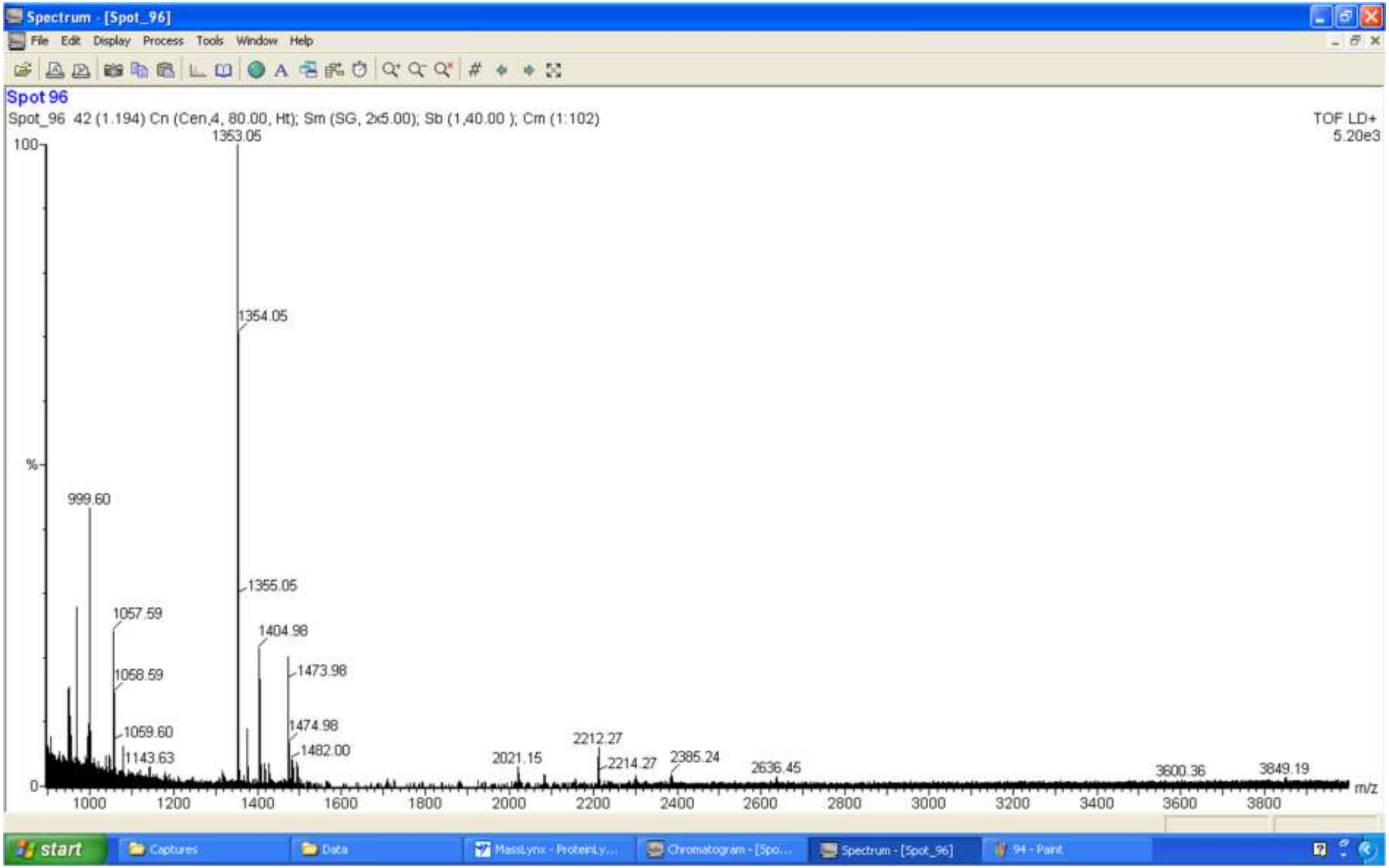
Spectrum - [Spot_96]
File Edit Display Process Tools Window Help
Spot 96
Spot_96 42 (1.194) Cn (Cen,4, 80.00, Ht); Sm (SG, 2x5.00); Sb (1,40.00); Cm (1:102)
TOF LD+
5.20e3
100
%
0
1353.05
1354.05
999.60
1355.05
1057.59
1404.98
1473.98
1058.59
1474.98
1059.60
1482.00
1143.63
2021.15
2212.27
2214.27
2385.24
2636.45
3600.36
3849.19
1000
1200
1400
1600
1800
2000
2200
2400
2600
2800
3000
3200
3400
3600
3800
m/z
start
Captures
Data
MassLynx - ProteinLy...
Chromatogram - [Spo...
Spectrum - [Spot_96]
94 - Paint
