# Supplementary material for: Proteomic Profiling and Protein Identification by MALDI-TOF Mass Spectrometry in Unsequenced Parasitic Nematodes
Source: PLoS One. 2012 Mar 29;7(3):e33590. doi: 10.1371/journal.pone.0033590 (PMC3315570; doi:10.1371/journal.pone.0033590)

Figure S2 (S2.01 – S2.47). ***De novo*** **limited peptide sequencing from MS/MS spectra for each 2D gel spot, together with the output of FASTS searches of the sequences against the NCBI nr protein database.**

The results of these searches for each spot number is summarised in Supplementary Multi-Media Tables S5, S6 and S7.

Figure S2.01.

Spot 7 MS/MS sequence 1
Ion 650.37

```
>>gi|111378147|emb|CAL30086.1| calreticulin precursor [Heligmosomoides polyg  (403 aa)
 initn: 137 init1:  77 opt: 137  Z-score: 31.1  bits: 44.0 E(13605827): 0.0058
Smith-Waterman score: 137; 77.8% identity (83.3% similar) in 18 aa overlap (1-18:24-336)
Entrez Lookup  Re-search database  General re-search

QUERY                         FLDDSWEK----------------------------------------------
                              : ::::::
gi|111 MRSLAVLLPLLGLVAAEVYFKEEFSDDSWEKRWVQSKHKDDYGAFKLSAGKYFDDEKRDQGLKTSQDAKFYSRAAKFPKP
               10        20        30        40        50        60        70        80

QUERY  --------------------------------------------------------------------------------

gi|111 FSNKGKTVVIQYTVKHEQGIDCGGGYVKVMSSDVNLEDFHGETPYNVMFGPDICGPTKKVHVIFSYKGKNHLIKKDIRCK
               90       100       110       120       130       140       150       160

QUERY  --------------------------------------------------------------------------------

gi|111 DDELTHLYTLILRPDNTYEVQIDGEKVESGEIEADWDMLPPKKIKDPDAKKPEDWDEREYIDDAEDKKPEDWDKPEHIPD
              170       180       190       200       210       220       230       240

QUERY  --------------------------------------------------------------------------------

gi|111 PDAKKPDDWDDEMDGEWEPPMIDNPDYKGEWKAKQIKNPDYKGKWIHPEIDNPEYTPDDELYLYKDWGAIGFDLWQVKSG
              250       260       270       280       290       300       310       320
```

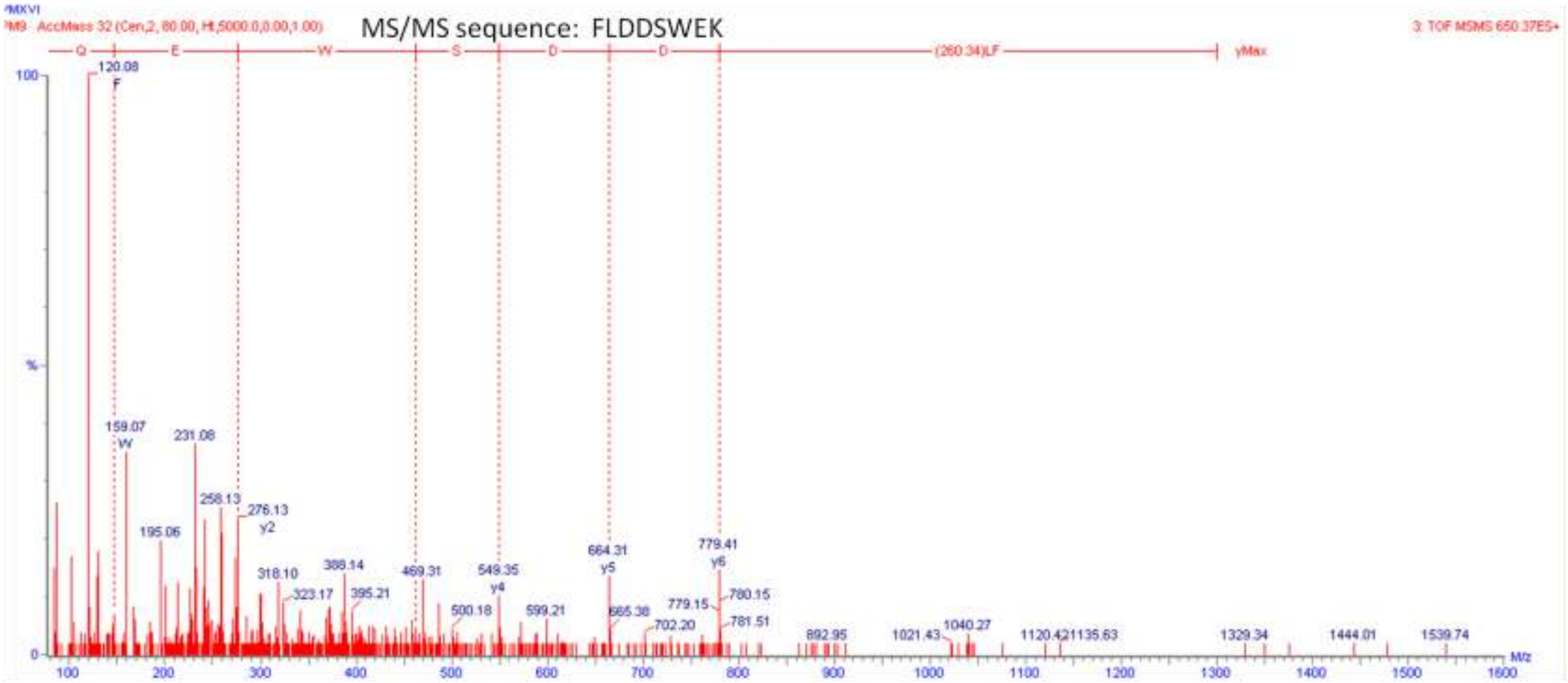

Figure S2.02.

```
>>gi|111378147|emb|CAL30086.1| calreticulin precursor [Heligmosomoides polyg  (403 aa)
 initn: 137 init1:  77 opt: 137  Z-score: 31.1  bits: 44.0 E(13605827): 0.0058
Smith-Waterman score: 137; 77.8% identity (83.3% similar) in 18 aa overlap (1-18:24-336)
Entrez Lookup  Re-search database  General re-search

QUERY                        FLDDSWEK------------------------------------------------
                             : ::::::
gi|111 MRSLAVLLPLLGLVAAEVYFKEEFSDDSWEKRWVQSKHKDDYGAFKLSAGKYFDDEKRDQGLKTSQDAKFYSRAAKFPKP
               10        20        30        40        50        60        70        80

QUERY  --------------------------------------------------------------------------------

gi|111 FSNKGKTVVIQYTVKHEQGIDCGGGYVKVMSSDVNLEDFHGETPYNVMFGPDICGPTKKVHVIFSYKGKNHLIKKDIRCK
               90       100       110       120       130       140       150       160

QUERY  --------------------------------------------------------------------------------

gi|111 DDELTHLYTLILRPDNTYEVQIDGEKVESGEIEADWDMLPPKKIKDPDAKKPEDWDEREYIDDAEDKKPEDWDKPEHIPD
              170       180       190       200       210       220       230       240

QUERY  --------------------------------------------------------------------------------

gi|111 PDAKKPDDWDDEMDGEWEPPMIDNPDYKGEWKAKQIKNPDYKGKWIHPEIDNPEYTPDDELYLYKDWGAIGFDLWQVKSG
              250       260       270       280       290       300       310       320
```

Spot 7 MS/MS sequence 2
Ion 957.91

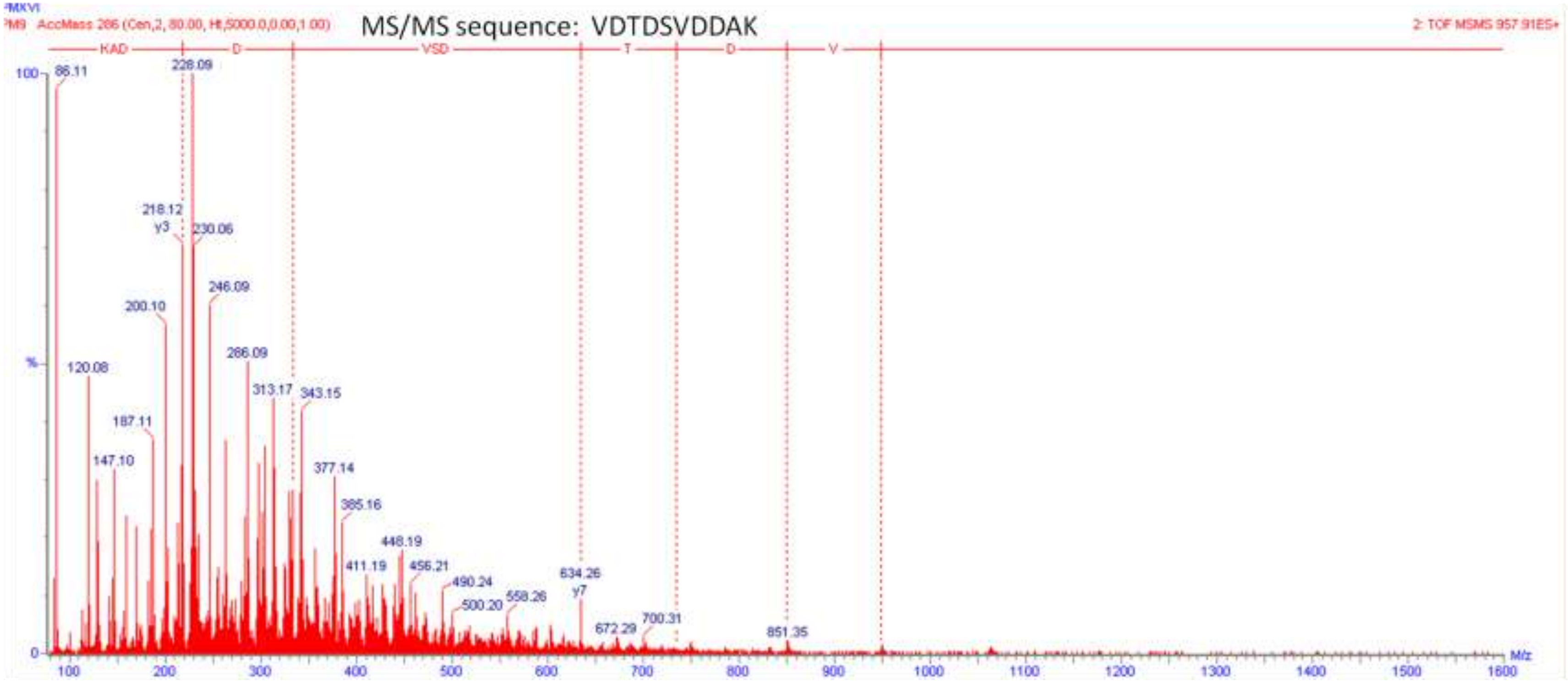

Figure S2.03.

```
>>>QUERY, 39 aa vs N library

>>gi|253721983|gb|ACT34055.1| glutamate dehydrogenase [Haemonchus contortus]  (532 aa)
 initn: 368 init1: 145 opt: 368  Z-score: 106.1  bits: 120.4 E(13331964): 1.6e-25
Smith-Waterman score: 368; 94.6% identity (94.6% similar) in 37 aa overlap (1-37:199-475)
Entrez Lookup  Re-search database  General re-search
                                                          10
QUERY                                                DVPAPDMGTGER---------------------------
                                                     ::::::::::::
gi|253 GGAKGGVKIDPKQYTDYEIEKITRRIAIEFAKKGFLGPGVDVPAPDMGTGEREMGWIADTYAQTIGHLDRDASACITGKP
           160       170       180       190       200       210       220       230

QUERY  --------------------------------------------------------------------------------

gi|253 IVAGGIHGRVSATGRGVWKGLEVFTKEPEYMNKVGLSLGLEGKTIITQGFGNVGLHTMRYLHRAGAKVIGVQEWDCAVFN
           240       250       260       270       280       290       300       310

QUERY  --------------------------------------------------------------------------------

gi|253 PDGIHPKELEDWRDENGTIKNFPKAKNFEPFAELMYEPCDIFVPAACEKAIHKENANRIQAKIIAEAANGPTTPAADKIL
           320       330       340       350       360       370       380       390

                                                                 20            30
QUERY  ------------------------------------------------FLLKSVQDSLEK----EAPVHPNDEFTAR
                                                         :: ::::::::    :::::::::::::
gi|253 LERGNCLIIPDMFINSGGVTVSYFEWLKNLNHVSYGRLSFKYEEDSNRMLLQSVQDSLEKALNKEAPVHPNDEFTARIAG
           400       410       420       430       440       450       460       470

gi|253 ASEKDIVHSGLEYTMTRSGEAIIRTARKYNLGLDIRTAAYANSIEKYIIPTELP
           480       490       500       510       520       530
```

Spot 12 MS/MS sequence 1
Ion 703.80

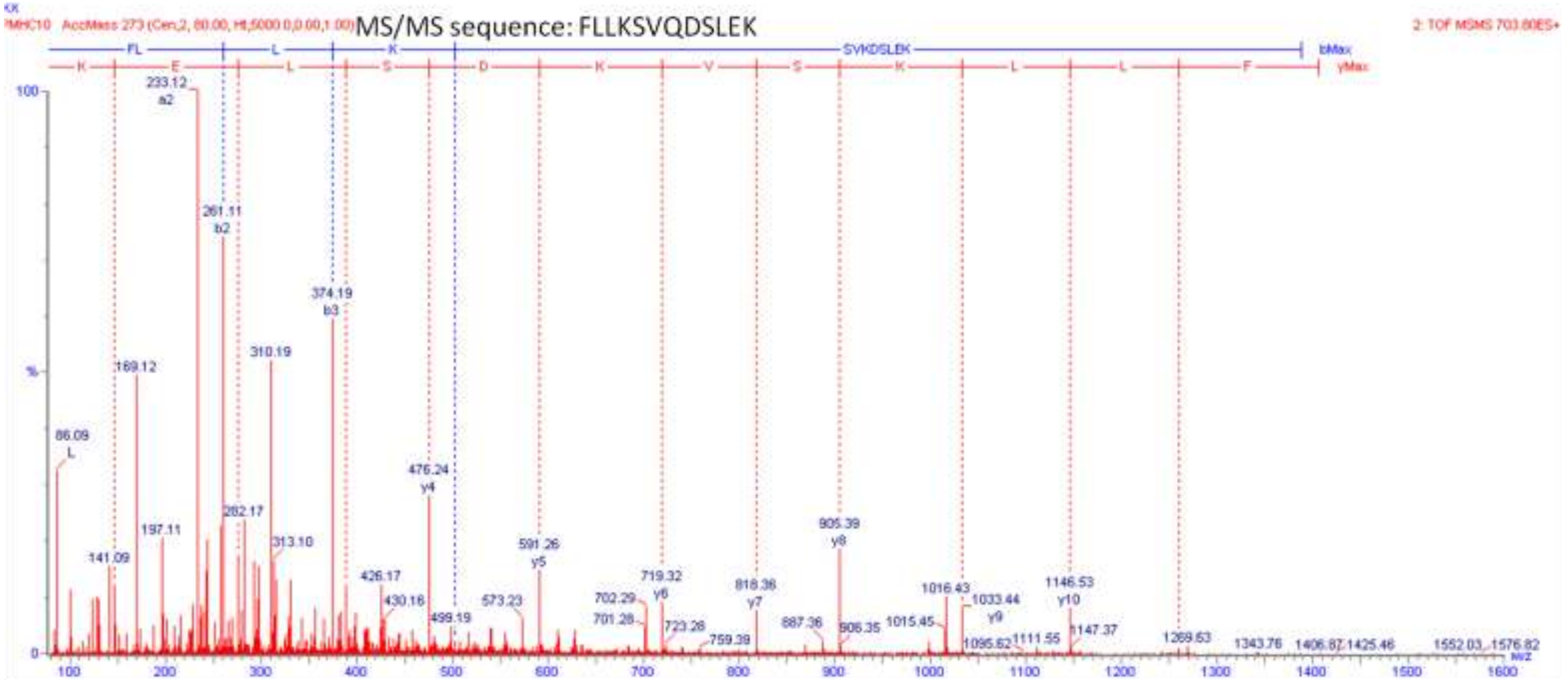

Figure S2.04.

Spot 12 MS/MS sequence 2
Ion 741.79

```
>>>QUERY, 39 aa vs N library

>>gi|253721983|gb|ACT34055.1| glutamate dehydrogenase [Haemonchus contortus]  (532 aa)
 initn: 368 initl: 145 opt: 368  Z-score: 106.1  bits: 120.4 E(13331964): 1.6e-25
Smith-Waterman score: 368; 94.6% identity (94.6% similar) in 37 aa overlap (1-37:199-475)
Entrez Lookup  Re-search database  General re-search
                                                        10
QUERY                                               DVPAPDMGTGER----------------------------
                                                    ::::::::::::
gi|253 GGAKGGVKIDPKQYTDYEIEKITRRIAIEFAKKGFLGPGVDVPAPDMGTGEREMGWIADTYAQTIGHLDRDASACITGKP
           160       170       180       190       200       210       220       230

QUERY  --------------------------------------------------------------------------------

gi|253 IVAGGIHGRVSATGRGVWKGLEVFTKEPEYMNKVGLSLGLEGKTIITQGFGNVGLHTMRYLHRAGAKVIGVQEWDCAVFN
           240       250       260       270       280       290       300       310

QUERY  --------------------------------------------------------------------------------

gi|253 PDGIHPKELEDWRDENGTIKNFPKAKNFEPFAELMYEPCDIFVPAACEKAIHKENANRIQAKIIAEAANGPTTPAADKIL
           320       330       340       350       360       370       380       390

                                                                   20            30
QUERY  ------------------------------------------------FLLKSVQDSLEK----EAPVHPNDEFTAR
                                                         :: ::::::::    :::::::::::::
gi|253 LERGNCLIIPDMFINSGGVTVSYFEWLKNLNHVSYGRLSFKYEEDSNRMLLQSVQDSLEKALNKEAPVHPNDEFTARIAG
           400       410       420       430       440       450       460       470

gi|253 ASEKDIVHSGLEYTMTRSGEAIIRTARKYNLGLDIRTAAYANSIEKYIIPTELP
           480       490       500       510       520       530
```

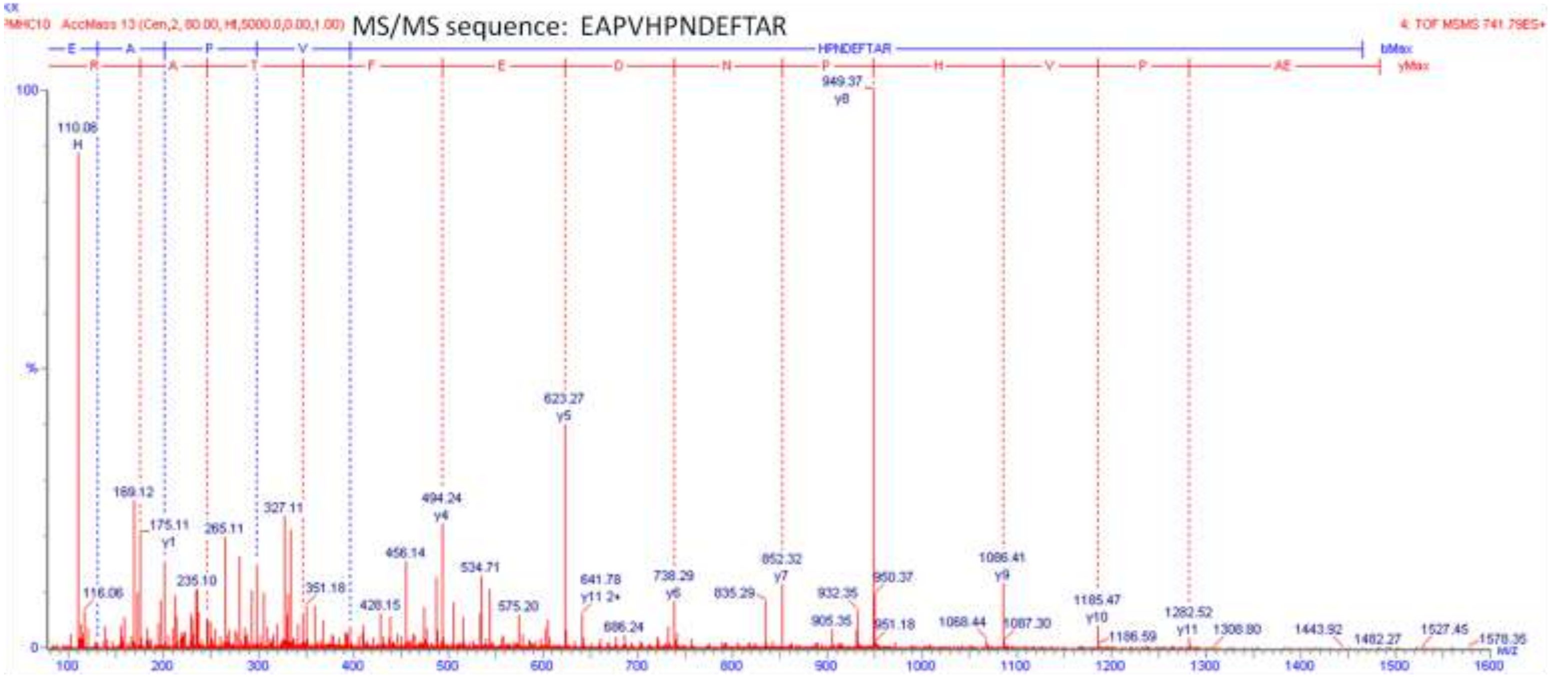

Figure S2.05.

Spot 12 MS/MS sequence 3
Ion 936.38

```
>>>QUERY, 39 aa vs N library

>>gi|253721983|gb|ACT34055.1| glutamate dehydrogenase [Haemonchus contortus]  (532 aa)
 initn: 368 init1: 145 opt: 368  Z-score: 106.1  bits: 120.4 E(13331964): 1.6e-25
Smith-Waterman score: 368; 94.6% identity (94.6% similar) in 37 aa overlap (1-37:199-475)
Entrez Lookup  Re-search database  General re-search
                                                       10
QUERY                                          DVPAPDMGTGER----------------------------
                                               ::::::::::::
gi|253 GGAKGGVKIDPKQYTDYEIEKITRRIAIEFAKKGFLGPGVDVPAPDMGTGEREMGWIADTYAQTIGHLDRDASACITGKP
          160       170       180       190       200       210       220       230

QUERY  --------------------------------------------------------------------------------

gi|253 IVAGGIHGRVSATGRGVWKGLEVFTKEPEYMNKVGLSLGLEGKTIITQGFGNVGLHTMRYLHRAGAKVIGVQEWDCAVFN
          240       250       260       270       280       290       300       310

QUERY  --------------------------------------------------------------------------------

gi|253 PDGIHPKELEDWRDENGTIKNFPKAKNFEPFAELMYEPCDIFVPAACEKAIHKENANRIQAKIIAEAANGPTTPAADKIL
          320       330       340       350       360       370       380       390

                                                             20            30
QUERY  ------------------------------------------------FLLKSVQDSLEK----EAPVHPNDEFTAR
                                                        :: ::::::::    :::::::::::::
gi|253 LERGNCLIIPDMFINSGGVTVSYFEWLKNLNHVSYGRLSFKYEEDSNRMLLQSVQDSLEKALNKEAPVHPNDEFTARIAG
          400       410       420       430       440       450       460       470

gi|253 ASEKDIVHSGLEYTMTRSGEAIIRTARKYNLGLDIRTAAYANSIEKYIIPTELP
          480       490       500       510       520       530
```

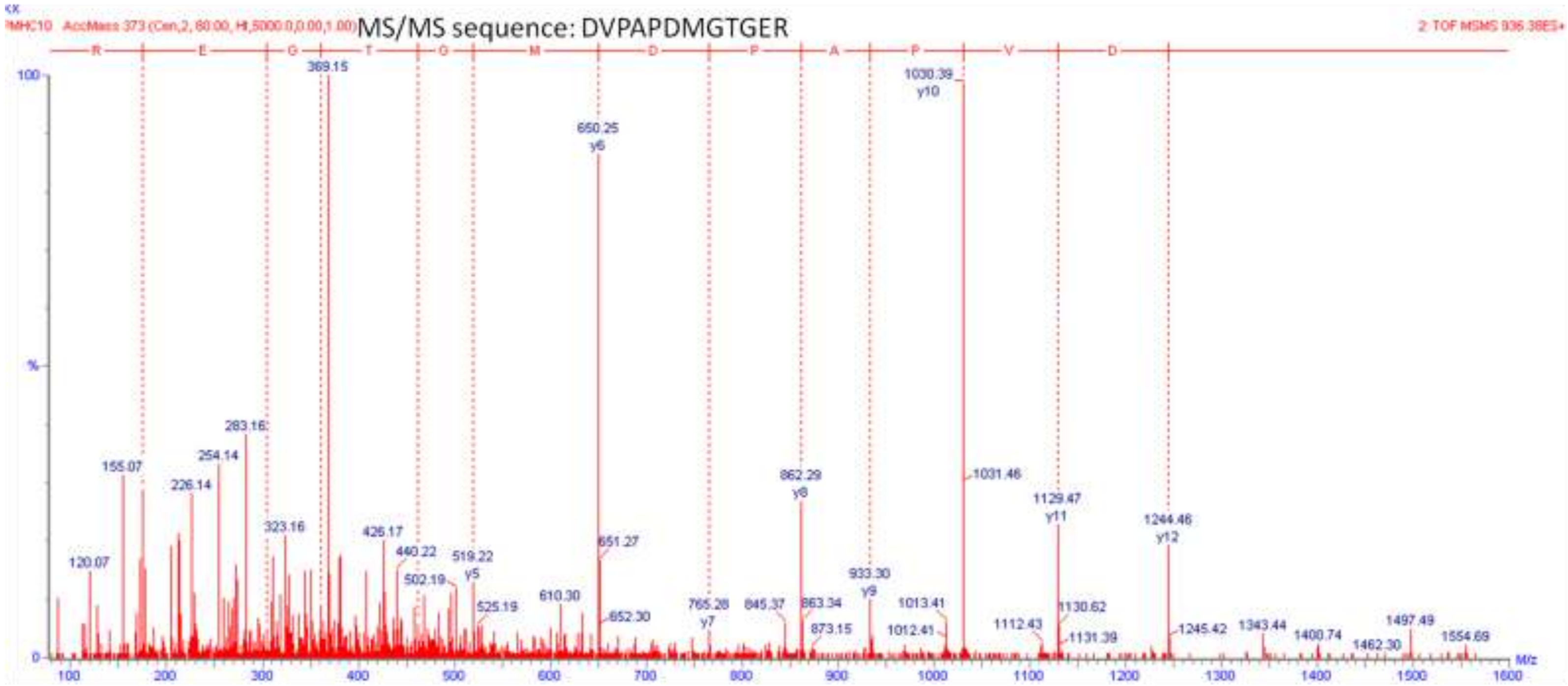

Figure S2.06.

Spot 13 MS/MS sequence 1
Ion 732.74

```
>>>QUERY, 37 aa vs N library

>>gi|253721983|gb|ACT34055.1| glutamate dehydrogenase [Haemonchus contortus]  (532 aa)
 initn: 333 init1: 124 opt: 333  Z-score: 89.4  bits: 103.6 E(13331964): 1.7e-20
Smith-Waterman score: 333; 85.7% identity (94.3% similar) in 35 aa overlap (1-35:104-396)
Entrez Lookup  Re-search database  General re-search
                                                    10
QUERY                                         NDGEFEVLEAWR---------------------------
                                              ..:::::: ::::
gi|253 VEEMKSNVMSTKDKKNLVSGILKAIKPVNKVLYITFPIRRDNGEFEVIEAWRAQHSEMRTPTRGGIRYSMDVCEDEVKAL
             70        80        90       100       110       120       130       140

QUERY  --------------------------------------------------------------------------------

gi|253 SALMTYKCAAVDVPFGGAKGGVKIDPKQYTDYEIEKITRRIAIEFAKKGFLGPGVDVPAPDMGTGEREMGWIADTYAQTI
            150       160       170       180       190       200       210       220

QUERY  --------------------------------------------------------------------------------

gi|253 GHLDRDASACITGKPIVAGGIHGRVSATGRGVWKGLEVFTKEPEYMNKVGLSLGLEGKTIITQGFGNVGLHTMRYLHRAG
            230       240       250       260       270       280       290       300

                                    20
QUERY  -------------------------DWRDENGTLK---------------------------------------------
                                :::::::: :
gi|253 AKVIGVQEWDCAVFNPDGIHPKELEDWRDENGTIKNFPKAKNFEPFAELMYEPCDIFVPAACEKAIHKENANRIQAKIIA
            310       320       330       340       350       360       370       380

          30
QUERY  EAADGPTTPAADK
       :::.:::::::::
gi|253 EAANGPTTPAADKILLERGNCLIIPDMFINSGGVTVSYFEWLKNLNHVSYGRLSFKYEEDSNRMLLQSVQDSLEKALNKE
            390       400       410       420       430       440       450       460
```

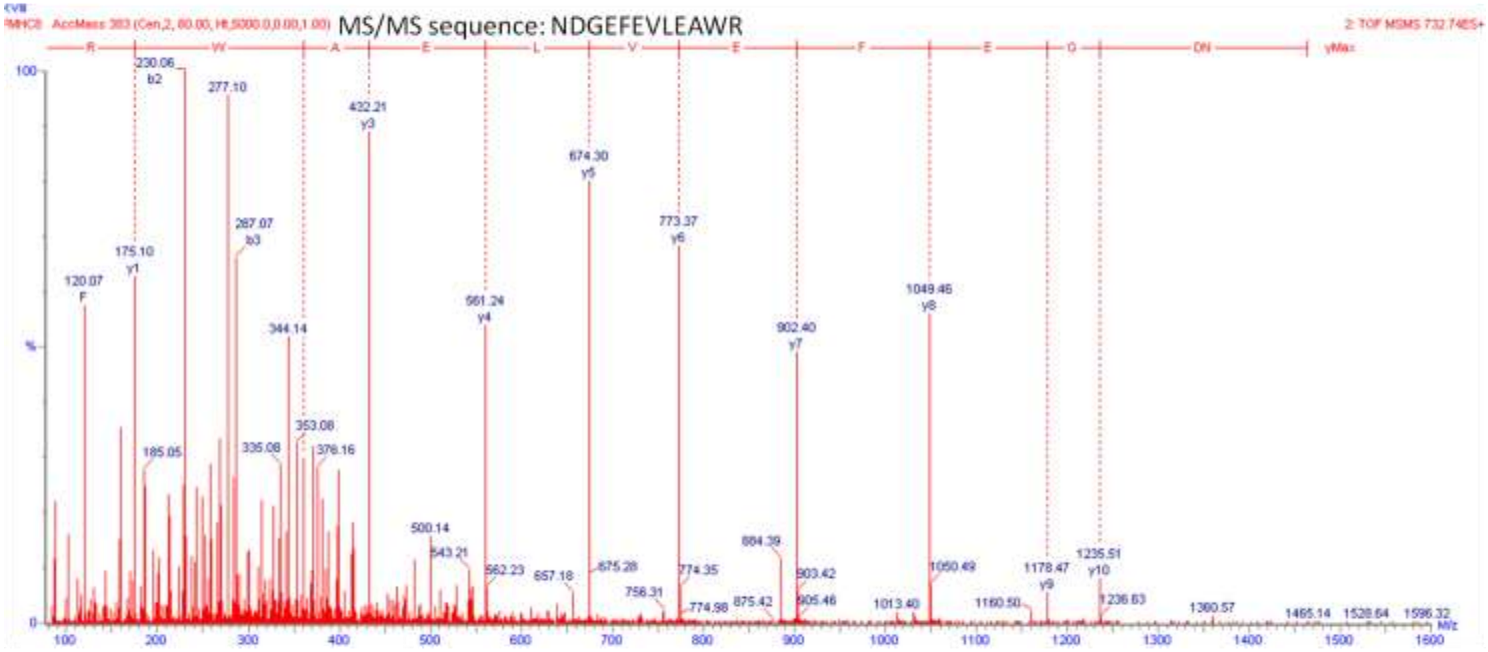

Figure S2.07.

```
>>>QUERY, 37 aa vs N library

>>gi|253721983|gb|ACT34055.1| glutamate dehydrogenase [Haemonchus contortus]  (532 aa)
 initn: 333 init1: 124 opt: 333  Z-score: 89.4  bits: 103.6 E(13331964): 1.7e-20
Smith-Waterman score: 333; 85.7% identity (94.3% similar) in 35 aa overlap (1-35:104-396)
Entrez Lookup  Re-search database  General re-search
                                                                10
QUERY                                                     NDGEFEVLEAWR---------------------------
                                                          ..:::::: ::::
gi|253 VEEMKSNVMSTKDKKNLVSGILKAIKPVNKVLYITFPIRRDNGEFEVIEAWRAQHSEHRTPTRGGIRYSMDVCEDEVKAL
           70        80        90       100       110       120       130       140

QUERY  ------------------------------------------------------------------------------------

gi|253 SALMTYKCAAVDVPFGGAKGGVKIDPKQYTDYEIEKITRRIAIEFAKKGFLGPGVDVPAPDMGTGEREMGWIADTYAQTI
          150       160       170       180       190       200       210       220

QUERY  ------------------------------------------------------------------------------------

gi|253 GHLDRDASACITGKPIVAGGIHGRVSATGRGVWKGLEVFTKEPEYMNKVGLSLGLEGKTIITQGFGNVGLHTMRYLHRAG
          230       240       250       260       270       280       290       300

                                    20
QUERY  -------------------------DWRDENGTLK-------------------------------------------
                                :::::::: :
gi|253 AKVIGVQEWDCAVFNPDGIHPKELEDWRDENGTIKNFPKAKNFEPFAELMYEPCDIFVPAACEKAIHKENANRIQAKIIA
          310       320       330       340       350       360       370       380

           30
QUERY  EAADGPTTPAADK
       :::.:::::::::
gi|253 EAANGPTTPAADKILLERGNCLIIPDMFINSGGVTVSYFEWLKNLNHVSYGRLSFKYEEDSNRMLLQSVQDSLEKALNKE
          390       400       410       420       430       440       450       460
```

Spot 13 MS/MS sequence 2
Ion 770.84

MS/MS sequence: EAADGPTTPAADK

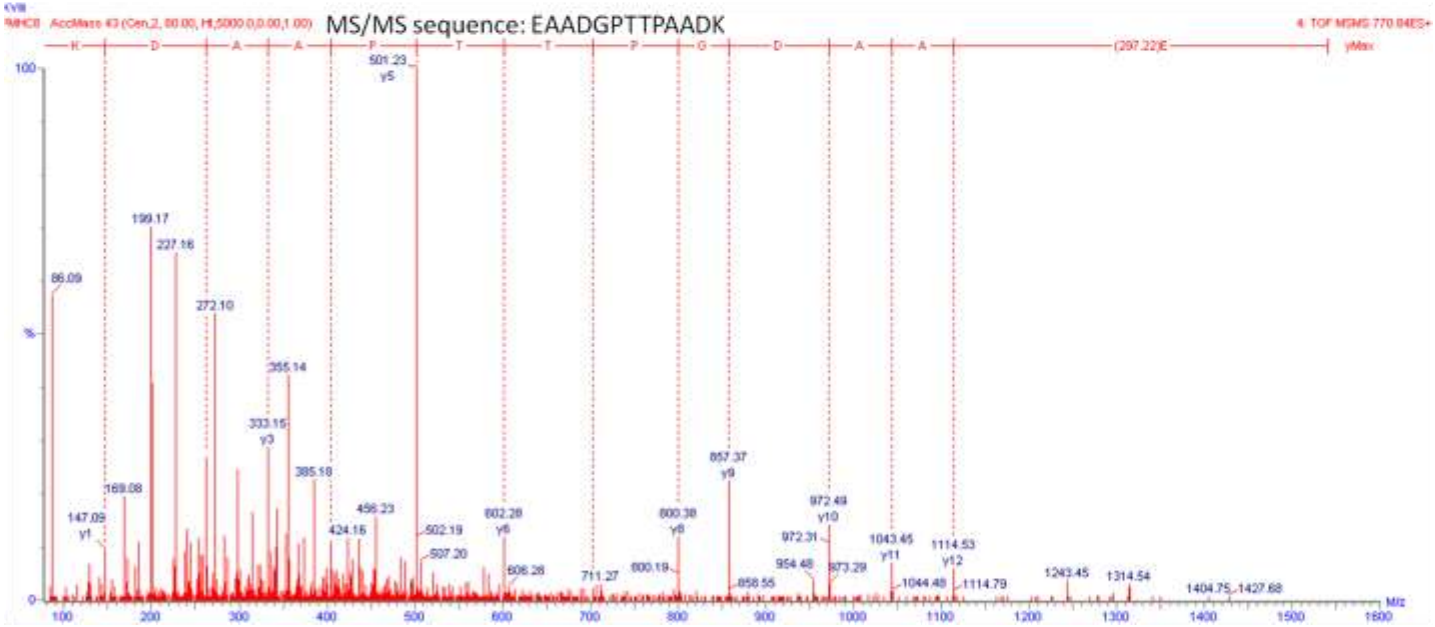

Figure S2.08.

Spot 13 MS/MS sequence 3
Ion 794.35

```
>>>QUERY, 37 aa vs N library

>>gi|253721983|gb|ACT34055.1| glutamate dehydrogenase [Haemonchus contortus]  (532 aa)
 initn: 333 init1: 124 opt: 333  Z-score: 89.4  bits: 103.6 E(13331964): 1.7e-20
Smith-Waterman score: 333; 85.7% identity (94.3% similar) in 35 aa overlap (1-35:104-396)
Entrez Lookup  Re-search database  General re-search
                                                         10
QUERY                                              NDGEFEVLEAWR---------------------------
                                                   ..::::: ::::
gi|253 VEEMKSNVNSTKDKKNLVSGILKAIKPVNKVLYITFPIRRDNGEFEVIEAWRAQHSEHRIPTRGGIRYSMDVCEDEVKAL
              70        80        90       100       110       120       130       140

QUERY  --------------------------------------------------------------------------------

gi|253 SALMTYKCAAVDVPFGGAKGGVKIDPKQYTDYKIEKITRRIAIEFAKKGFLGPGVDVPAPDMGTGEREMGWIADTYAQTI
             150       160       170       180       190       200       210       220

QUERY  --------------------------------------------------------------------------------

gi|253 GHLDRDASACITGKPIVAGGIHGRVSATGRGVWKGLEVFTKEPEYMSKVGLSLGLEGKTIIITQGFGNVGLHTMRYLHRAG
             230       240       250       260       270       280       290       300

                                    20
QUERY  -------------------------DWRDENGTLK---------------------------------------------
                                 ::::::::: :
gi|253 AKVIGVQEWDCAVFNPDGIHPKELEDWRDENGTIKNFPKAKNFEPFAKLMYEPCDIFVPAACEKAIHKENANRIQAKIIA
             310       320       330       340       350       360       370       380

             30
QUERY  EAADGPTTPAADK
       :::.:::::::::
gi|253 EAANGPTTPAADKILLERGNCLIIPDMFINSGGVTVSYFEWLKNLNHVSYGRLSFKYEEDSNRMLLQSVQDSLEKALNKE
             390       400       410       420       430       440       450       460
```

MS/MS sequence: DWRDENGTLK

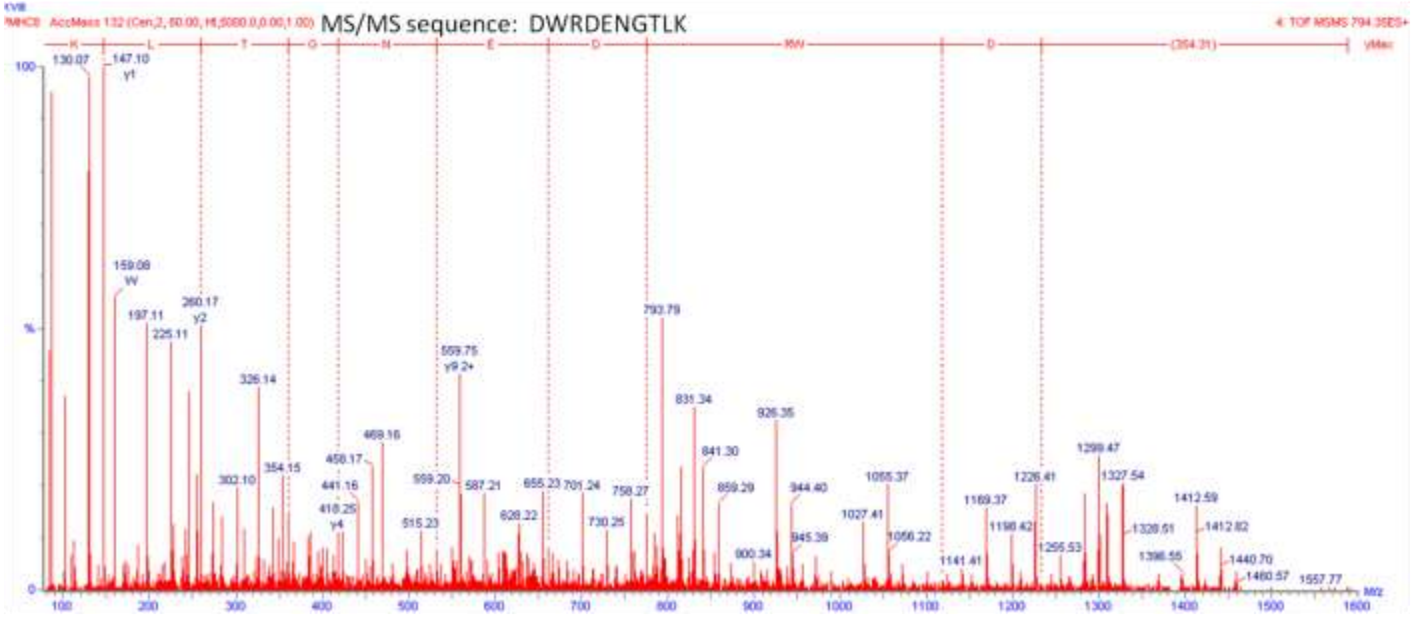

Figure S2.09.

Spot 16 MS/MS sequence 1
Ion 664.74

>>>QUERY, 42 aa vs N library

```
>>gi|71994129|ref|NP_001024806.1| CALUmenin (calcium-binding protein) homolo  (314 aa)
 initn: 329 init1: 147 opt: 329  Z-score: 88.1  bits: 101.8 E(13331964): 4e-20
Smith-Waterman score: 329; 82.5% identity (87.5% similar) in 40 aa overlap (1-40:48-197)
Entrez Lookup  Re-search database  General re-search
                                                       10                  20
QUERY                                          DTAAEFDELTPEK-----------DMSDGDGFVEEK----
                                               :::::::::::::            .: :::.::
gi|719 GLLAATALASHHSSDPSKDGEHFKGKEHDKKYDHEQFLGKDTAAEFDELTPEKSKEKLAKLVPKMDADSDGFIEENELKD
          10        20        30        40        50        60        70        80

QUERY  --------------------------------------------------------------------------------

gi|719 HINFMQKRYVNNDVDRTWKNYKAEKIVDGKIKNEDYREMVYGSADGAGQELSPEYAKMIARDEKRWAVADYDSNGALDRT
          90       100       110       120       130       140       150       160

                             30        40
QUERY  ---------------DVVVAETVDDLDKNK
                      :::::::::: ::::
gi|719 EYGCFMHPEDCDMMRDVVVAETVDDIDKNKDGSVDLDEYIGDMYRPDDYPELNGKEPDWVQSEREMFKEHRDKDGDGKLN
         170       180       190       200       210       220       230       240
```

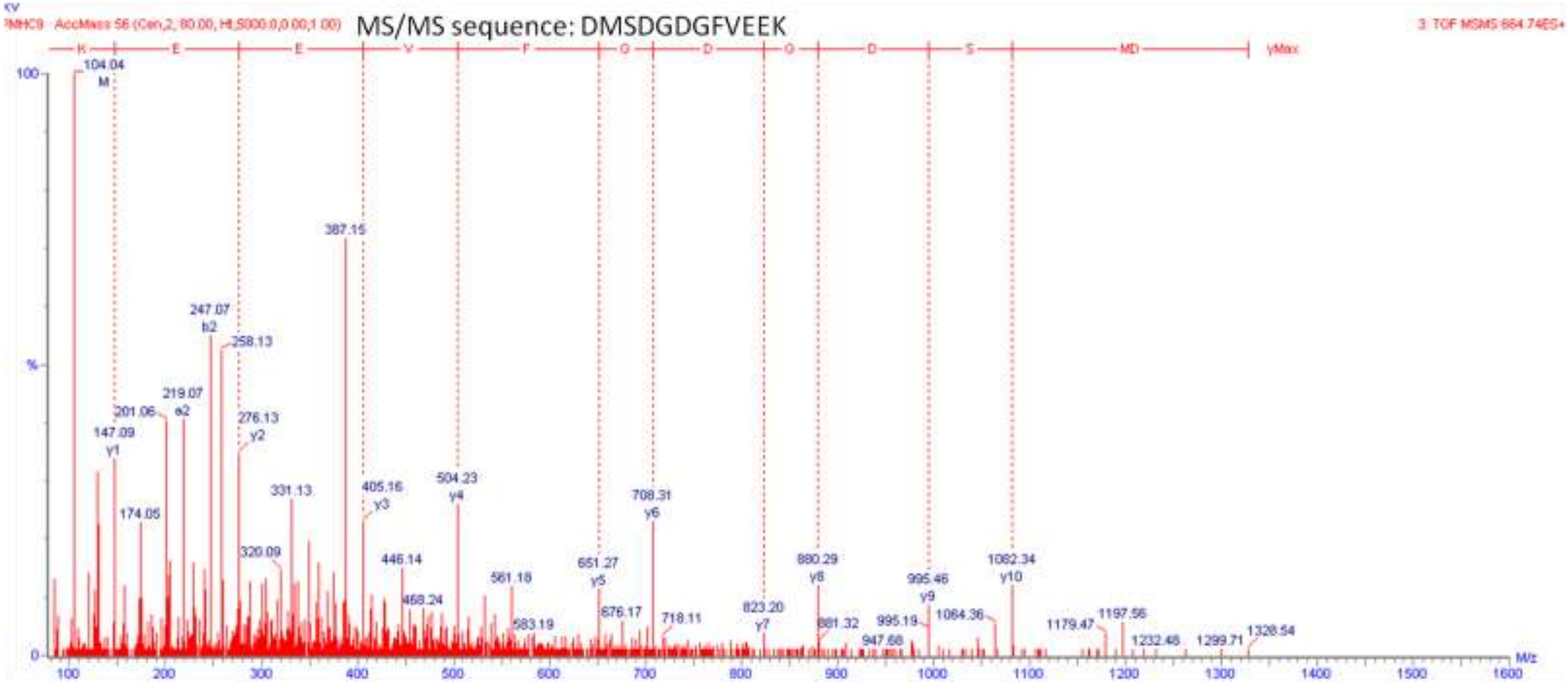

Figure S2.10.

Spot 16 MS/MS sequence 2
Ion 733.31

```
>>>QUERY, 42 aa vs N library

>>gi|71994129|ref|NP_001024806.1| CALUmenin (calcium-binding protein) homolo  (314 aa)
 initn: 329 init1: 147 opt: 329  Z-score: 88.1  bits: 101.8 E(13331964): 4e-20
Smith-Waterman score: 329; 82.5% identity (87.5% similar) in 40 aa overlap (1-40:48-197)
Entrez Lookup  Re-search database  General re-search
                                                                 10                  20
QUERY                                            DTAAEFDELTPEK-----------DMSDGDGFVEEK----
                                                 :::::::::::::           .: :::.::
gi|719 GLLAATALASHHSSDPSKDGEHFKGKEHDKKYDHEQFLGKDTAAEFDELTPEKSKEKLAKLVPKMDADSDGFIEENELKD
          10        20        30        40        50        60        70        80

QUERY  --------------------------------------------------------------------------------

gi|719 HINFMQKRYVNNDVDRTWKNYKAEKIVDGKIKWEDYREMVYGSADGAGQELSPEYAKMIARDEKRWAVADYDSNGALDRT
          90       100       110       120       130       140       150       160

                          30        40
QUERY  ---------------DVVVAETVDDLDKNK
                      :::::::::: ::::
gi|719 EYGCFMHPEDCDHMRDVVVAETVDDIDKNKDGSVDLDEYIGDMYRPDDYPELNGKEPDWVQSEREMFKEHRDKDGDGKLN
         170       180       190       200       210       220       230       240
```

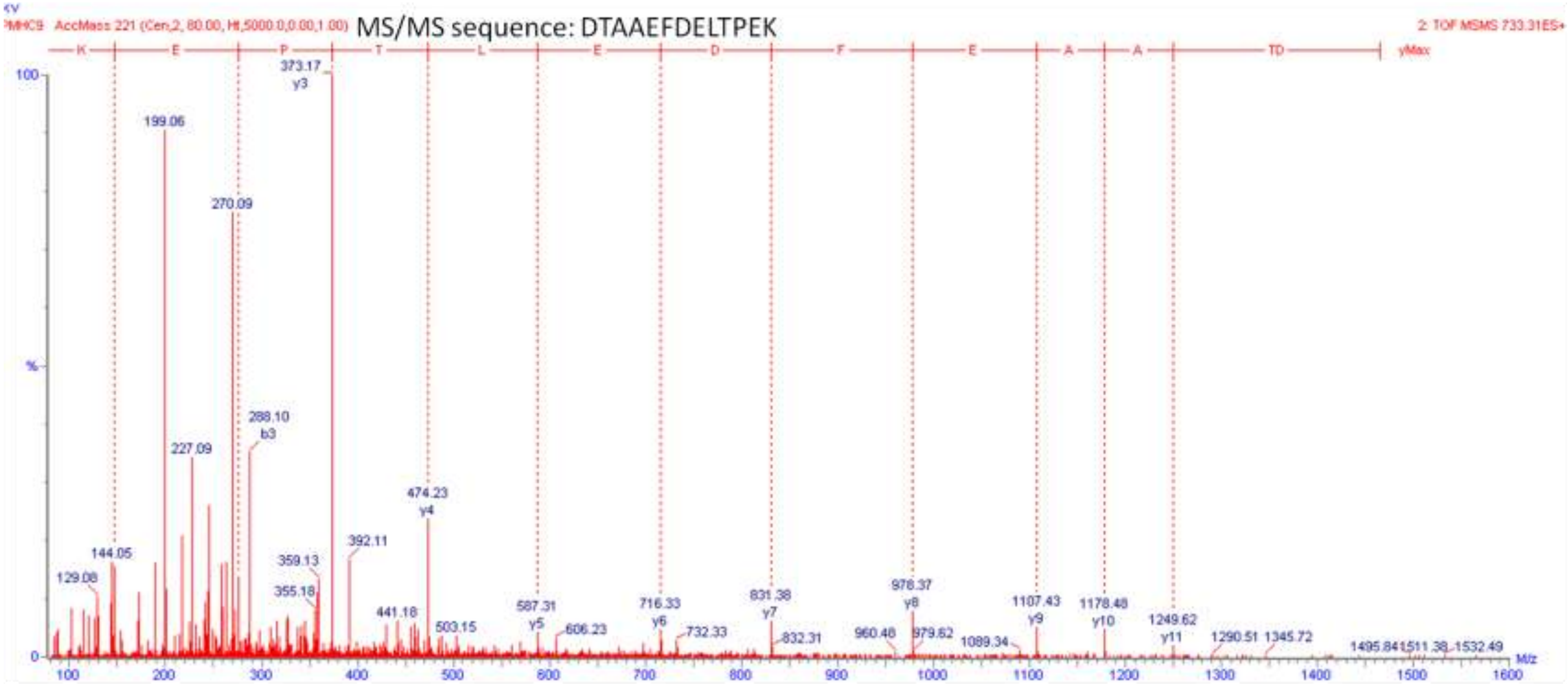

Figure S2.11.

```
>>>QUERY, 42 aa vs N library

>>gi|71994129|ref|NP_001024806.1| CALUmenin (calcium-binding protein) homolo  (314 aa)
 initn: 329 init1: 147 opt: 329  Z-score: 88.1  bits: 101.8 E(13331964): 4e-20
Smith-Waterman score: 329; 82.5% identity (87.5% similar) in 40 aa overlap (1-40:48-197)
Entrez Lookup  Re-search database  General re-search
                                                          10                  20
QUERY                                          DTAAEFDELTPEK-----------DMSDGDGFVEEK----
                                               :::::::::::::             .: :::.::
gi|719 GLLAATALASHHSSDPSKDGEHFKGKEHDKKYDHEQFLGKDTAAEFDELTPEKSKEKLAKLVPKMDADSDGFIEENELKD
         10        20        30        40        50        60        70        80

QUERY  --------------------------------------------------------------------------------

gi|719 HINFMQKRYVNNDVDRTWKNYKAEKIVDGKIKWEDYREMVYGSADGAGQELSPEYAKMIARDEKRWAVADYDSNGALDRT
         90        100       110       120       130       140       150       160

                              30        40
QUERY  ---------------DVVVAETVDDLDKNK
                      :::::::::: ::::
gi|719 EYGCFMHPEDCDHMRDVVVAETVDDIDKNKDGSVDLDEYIGDMYRPDDYPELNGKEPDWVQSEREMFKEHRDKDGDGKLN
        170       180       190       200       210       220       230       240
```

Spot 16 MS/MS sequence 3
Ion 830.37

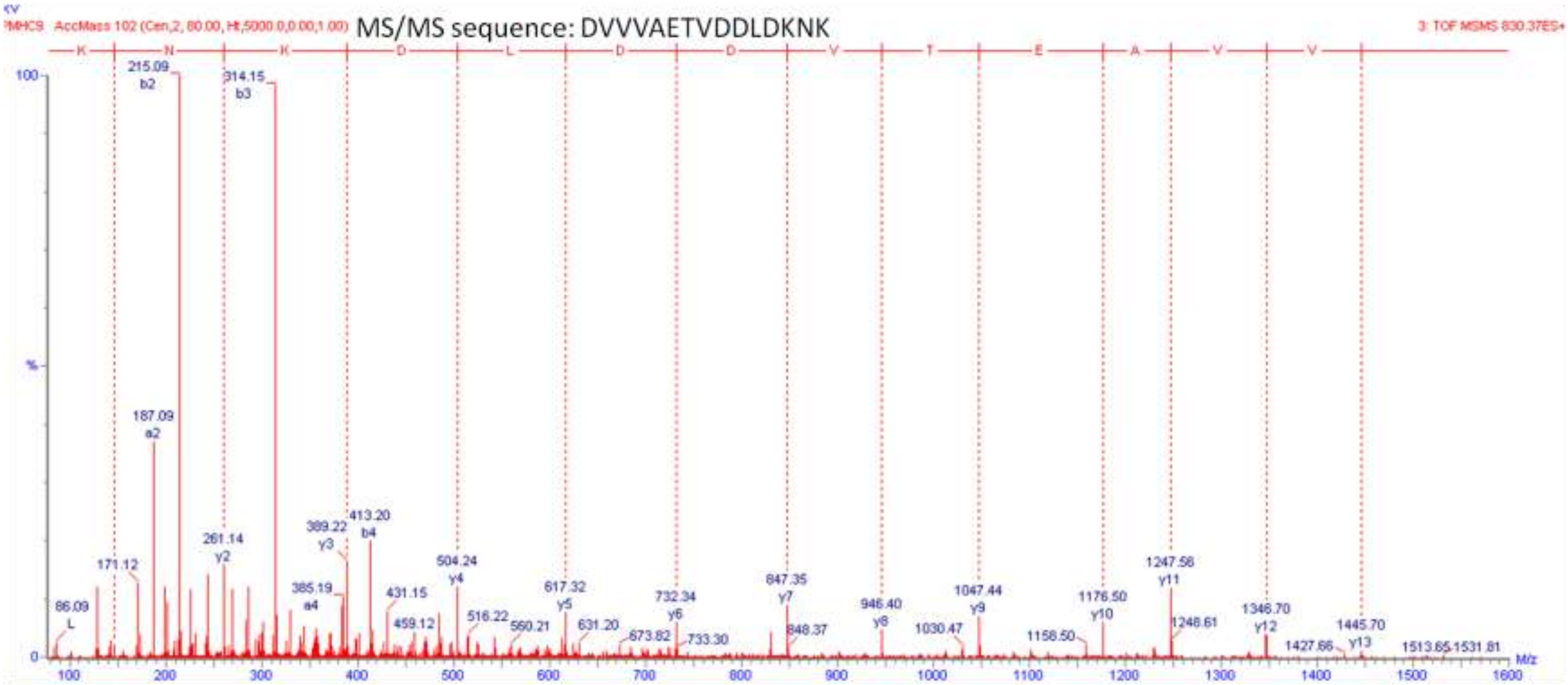

Figure S2.12.

Spot 26 MS/MS sequence 1
Ion 590.29

```
>>>QUERY, 25 aa vs N library

>>gi|301015486|gb|ADK47524.1| enolase [Haemonchus contortus]              (434 aa)
 initn: 182 init1: 132 opt: 182  Z-score: 47.9  bits: 61.3 E(13605827): 5.3e-08
Smith-Waterman score: 182; 83.3% identity (83.3% similar) in 24 aa overlap (1-24:38-182)
Entrez Lookup  Re-search database  General re-search
                                                   10
QUERY                                      GASTGVHEALELR------------------------------
                                           :::::::::::::
gi|301 MPITKIHARQIYDSRGNPTVEVDLYTEKGVFRAAVPSGASTGVHEALELRDQDKKVHHGKGVLKAVANINDKIAPALIAK
               10        20        30        40        50        60        70        80

QUERY  --------------------------------------------------------------------------------

gi|301 NFCVTQQRDIDQFMLALDGTENKSNLGANAILGVSLAVAKAGAVHKGMPLYKYIAELAGVSKVILPVPAFNVINGGSHAG
               90       100       110       120       130       140       150       160

                            20
QUERY  -----------LPVKTSFHEAM
                        :::::::
gi|301 NKLAMQEFMILPVGATSFHEAMRMGSEVYHHLKAEIKKRYGLDATAVGDEGGFAPNIQDNKEGLDLLKTAIDLAGYTGKI
              170       180       190       200       210       220       230       240
```

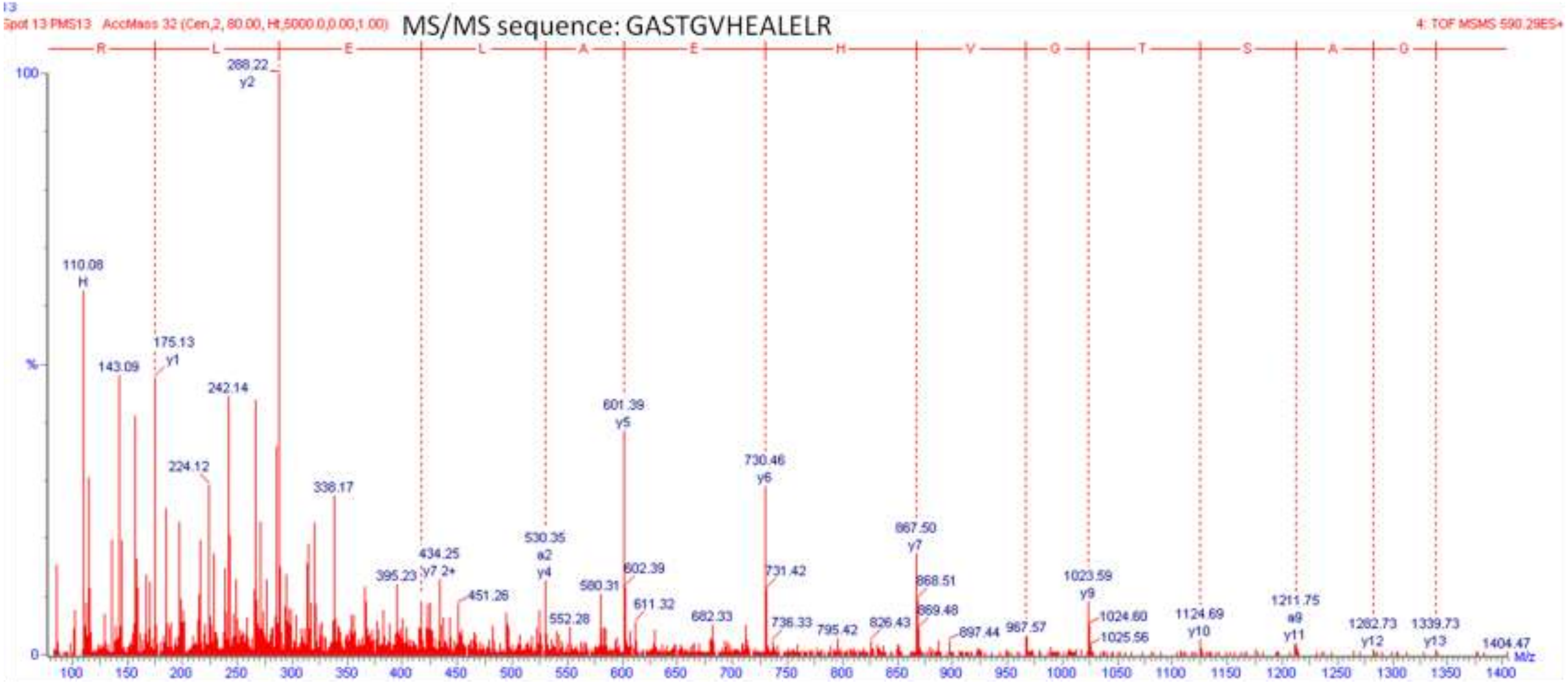

Figure S2.13.

Spot 26 MS/MS sequence 2
Ion 815.39

```
>>>QUERY, 25 aa vs N library

>>gi|301015486|gb|ADK47524.1| enolase [Haemonchus contortus]                    (434 aa)
 initn: 182 init1: 132 opt: 182  Z-score: 47.9  bits: 61.3 E(13605827): 5.3e-08
Smith-Waterman score: 182; 83.3% identity (83.3% similar) in 24 aa overlap (1-24:38-182)
Entrez Lookup  Re-search database  General re-search
                                                                  10
QUERY                                                     GASTGVHEALELR------------------------------
                                                          :::::::::::::
gi|301 MPITKIHARQIYDSRGNPTVEVDLYTEKGVFRAAVPSGASTGVHEALELRDQDKKVHHGKGVLKAVANINDKIAPALIAK
                10        20        30        40        50        60        70        80

QUERY  --------------------------------------------------------------------------------

gi|301 NFCVTQQRDIDQFMLALDGTENKSNLGANAILGVSLAVAKAGAVHKGMPLYKYIAELAGVSKVILPVPAFNVINGGSHAG
                90       100       110       120       130       140       150       160

                           20
QUERY  -----------LPVKTSFHEAM
                      :::::::
gi|301 NKLAMQEFMILPVGATSFHEAMRMGSEVYHHLKAEIKKRYGLDATAVGDEGGFAPNIQDNKEGLDLLKTAIDLAGYTGKI
               170       180       190       200       210       220       230       240
```

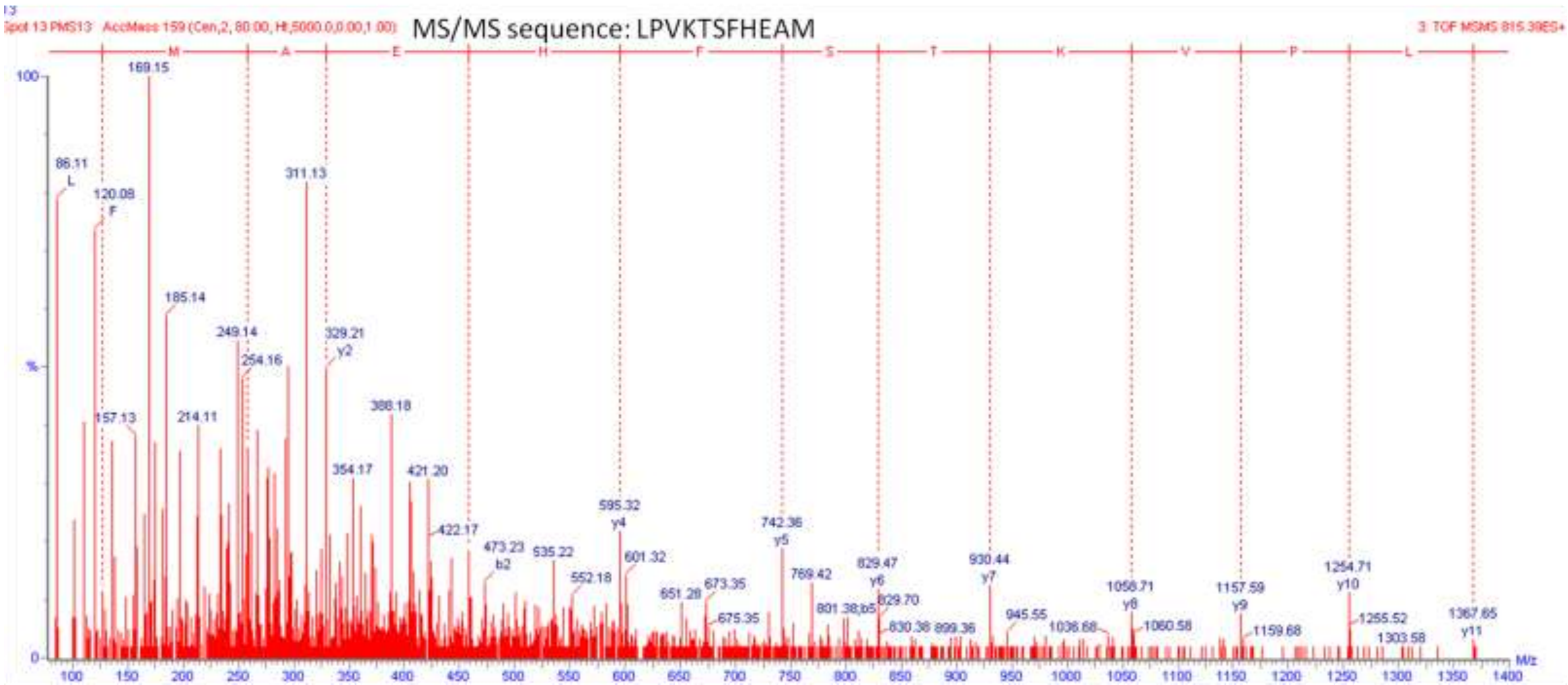

Figure S2.14.

```
>>>QUERY, 32 aa vs N library

>>gi|308452859|ref|XP_003089209.1| hypothetical protein CRE_23811 [Caenorhab  (231 aa)
 initn: 215 initl:  93 opt: 215  Z-score: 49.7  bits: 62.5 E(13331964): 1.5e-08
Smith-Waterman score: 215; 73.3% identity (80.0% similar) in 30 aa overlap (1-30:127-212)
Entrez Lookup  Re-search database  General re-search
                                                        10        20
QUERY                                            VLYLTIFLPR-NDGEFEVLEANR------------------
                                                 ::: ::  : ..::::: :: :
gi|308 KGAEVIAPKLVEELKSNSLSQKDKKNLVTGILGAIKPVNKVLYITFPIRRDNGEFEVVEAWRSQHSEHRTPTKGGIRYSL
           90       100       110       120       130       140       150       160

                                                     30
QUERY  -------------------------------------QYTDYELEK
                                             :::::: ::
gi|308 DVCEDEVKALSALMTYKCAVVDVPFGGAKGGVKIDPKQYTDYEIEKITRRIAIEFAKKGFLGPGS
          170       180       190       200       210       220       230
```

```
>>gi|253721983|gb|ACT34055.1| glutamate dehydrogenase [Haemonchus contortus]  (532 aa)
 initn: 216 initl:  93 opt: 216  Z-score: 46.3  bits: 60.4 E(13331964): 1.5e-07
Smith-Waterman score: 216; 73.3% identity (80.0% similar) in 30 aa overlap (1-30:94-179)
Entrez Lookup  Re-search database  General re-search
                                                        10        20
QUERY                                            VLYLTIFLPR-NDGEFEVLEANR------------------
                                                 ::: ::  : ..::::: :: :
gi|253 KGASVIEPKLVEEMKSNVNSTKDKKNLVSGILKAIKPVNKVLYITFPIRRDNGEFEVIEAWRAQHSEHRTPTRGGIRYSM
             60        70        80        90       100       110       120       130

                                                     30
QUERY  -------------------------------------QYTDYELEK
                                             :::::: ::
gi|253 DVCEDEVKALSALMTYKCAAVDVPFGGAKGGVKIDPKQYTDYEIEKITRRIAIEFAKKGFLGPGVDVPAPDMGTGEREMG
            140       150       160       170       180       190       200       210

gi|253 WIADTYAQTIGHLDRDASACITGKPIVAGGIHGRVSATGRGVWKGLEVFIKEPEYMNKVGLSLGLEGKIIITQGFGNVGL
            220       230       240       250       260       270       280       290
```

Spot 28 MS/MS sequence 1
Ion 561.31

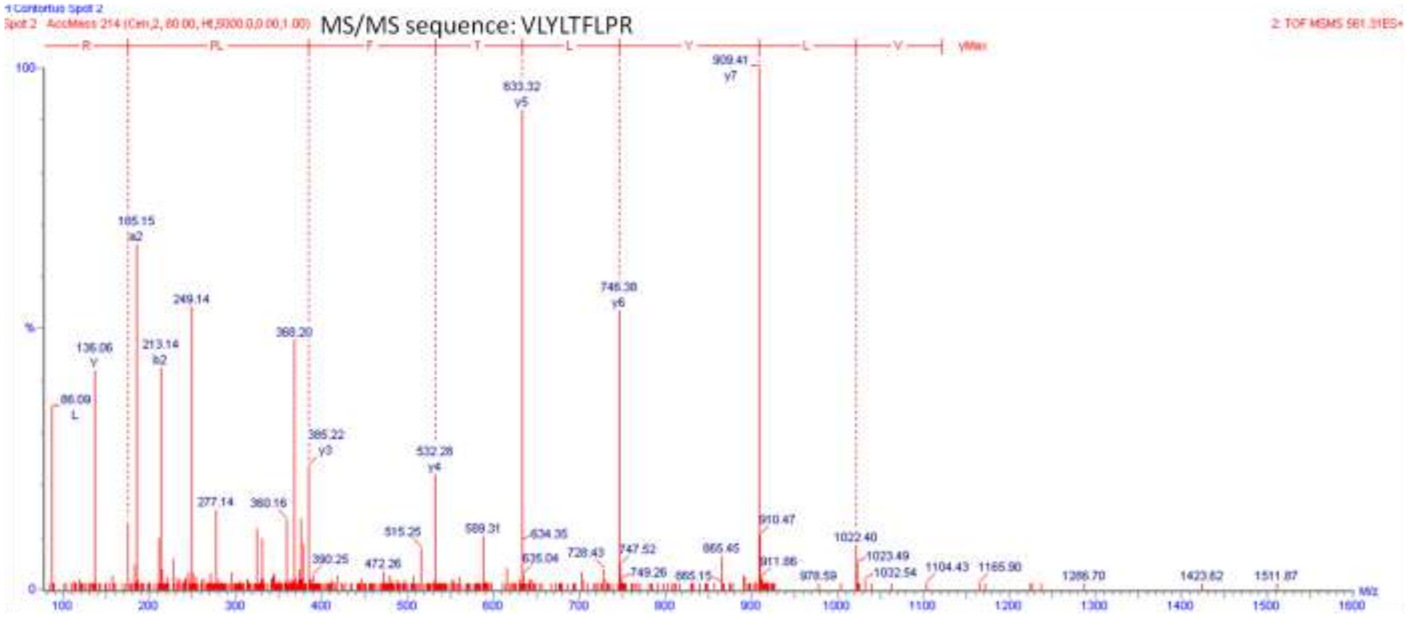

Figure S2.15.

Spot 28 MS/MS sequence 2
Ion 594.75

```
>>>QUERY, 32 aa vs N library

>>gi|308452859|ref|XP_003089209.1| hypothetical protein CRE_23811 (Caenorhab  (231 aa)
 initn: 215 init1:  93 opt: 215  Z-score: 49.7  bits: 62.5 E(13331964): 1.5e-08
Smith-Waterman score: 215; 73.3% identity (80.0% similar) in 30 aa overlap (1-30:127-212)
Entrez Lookup  Re-search database  General re-search
                                                                10        20
QUERY                                                   VLYLTFLPR-NDGEFEVLEANR------------------
                                                        ::: ::  : ..::::: :: :
gi|308 KGAEVIAPKLVEELKSNSLSQKDKKNLVTGILGAIKPVNKVLYITFPIRRDNGEFEVVEAWRSQHSEHRTPTKGGIRYSL
           90        100       110       120       130       140       150       160

                                                       30
QUERY  -------------------------------------QYTDYELEK
                                          :::::: ::
gi|308 DVCEDEVKALSALMTYKCAVVDVPFGGAKGGVKIDPKQYTDYEIEKITRRIAIEFAKKGFLGPGS
          170       180       190       200       210       220       230

>>gi|253721983|gb|ACT34055.1| glutamate dehydrogenase [Haemonchus contortus]  (532 aa)
 initn: 216 init1:  93 opt: 216  Z-score: 46.3  bits: 60.4 E(13331964): 1.5e-07
Smith-Waterman score: 216; 73.3% identity (80.0% similar) in 30 aa overlap (1-30:94-179)
Entrez Lookup  Re-search database  General re-search
                                                                10        20
QUERY                                                   VLYLTFLPR-NDGEFEVLEANR------------------
                                                        ::: ::  : ..::::: :: :
gi|253 KGASVIEPKLVEEMKSNVMSTKDKKNLVSGILKAIKPVNKVLYITFPIRRDNGEFEVIEAWRAQHSEHRTPTRGGIRYSM
             60        70        80        90       100       110       120       130

                                                       30
QUERY  -------------------------------------QYTDYELEK
                                          :::::: ::
gi|253 DVCEDEVKALSALMTYKCAAVDVPFGGAKGGVKIDPKQYTDYEIEKITRRIAIEFAKKGFLGPGVDVPAPDMGTGEREMG
            140       150       160       170       180       190       200       210

gi|253 WIADTYAQTIGHLDRDASACITGKPIVAGGIHGRVSATGRGVWKGLEVFTKEPEYMNKVGLSLGLEGRTIITQGFGNVGL
            220       230       240       250       260       270       280       290
```

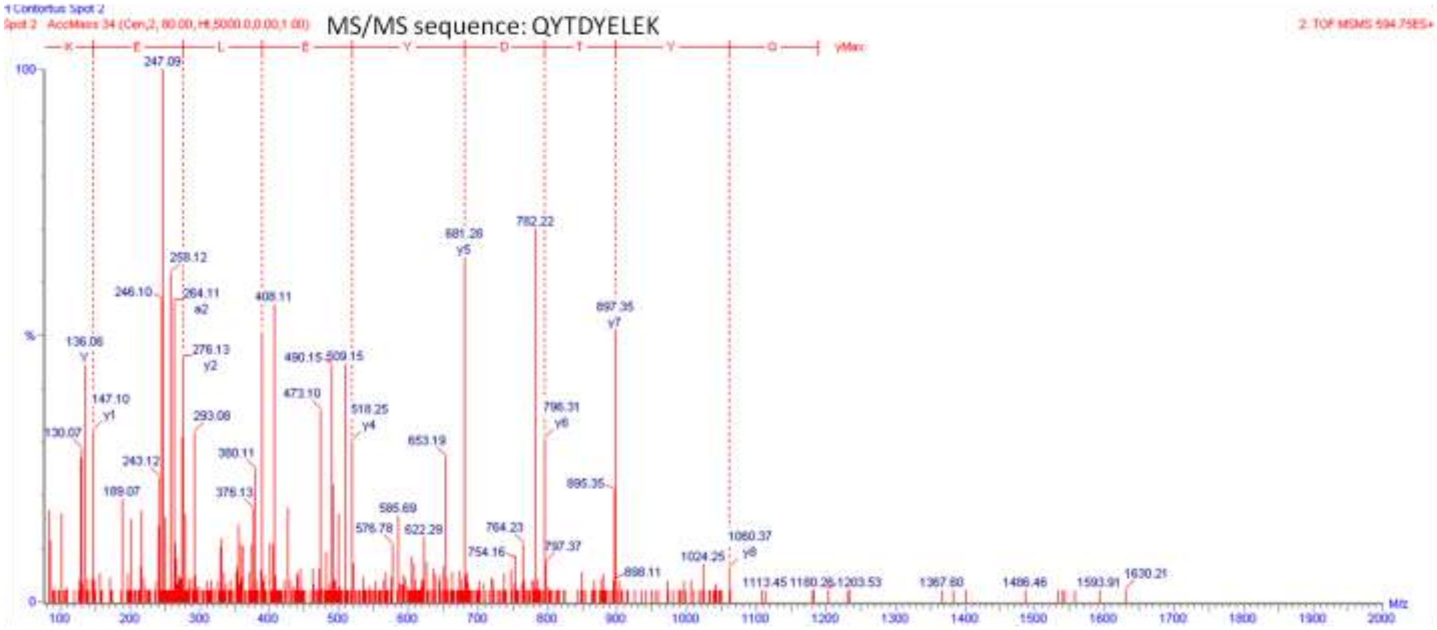

Figure S2.16.

Spot 28 MS/MS sequence 3
Ion 732.81

```
>>>QUERY, 32 aa vs N library

>>gi|308452859|ref|XP_003089209.1| hypothetical protein CRE_23811 [Caenorhab  (231 aa)
 initn: 215 init1:  93 opt: 215  Z-score: 49.7  bits: 62.5 E(13331964): 1.5e-06
Smith-Waterman score: 215; 73.3% identity (80.0% similar) in 30 aa overlap (1-30:127-212)
Entrez Lookup  Re-search database  General re-search
                                                          10        20
QUERY                                            VLYLTFLPR-NDGEFEVLEANR------------------
                                                 ::: ::  :  ..::::: :: :
gi|308 KGAEVIAPKLVEEKLKSNSLSQKDKKNLVTGILGAIKPVNKVLYITFPIRRDNGEFEVVEAWRSQHSEHRTPTKGGIRYSL
               90       100       110       120       130       140       150       160

                                                  30
QUERY  -------------------------------------QYTDYELEK
                                             :::::: ::
gi|308 DVCEDEVKALSALMTYKCAVVDVPFGGAKGGVKIDPKQYTDYEIEKITRRIAIEFAKKGFLGPGS
            170       180       190       200       210       220       230

>>gi|253721983|gb|ACT34055.1| glutamate dehydrogenase [Haemonchus contortus]  (532 aa)
 initn: 216 init1:  93 opt: 216  Z-score: 46.3  bits: 60.4 E(13331964): 1.5e-07
Smith-Waterman score: 216; 73.3% identity (80.0% similar) in 30 aa overlap (1-30:94-179)
Entrez Lookup  Re-search database  General re-search
                                                          10        20
QUERY                                            VLYLTFLPR-NDGEFEVLEANR------------------
                                                 ::: ::  :  ..::::: :: :
gi|253 KGASVIEPKLVEEMKSNVMSTKDKKNLVSGILKAIKPVNKVLYIIFPIRRDNGEFEVIEAWRAQHSEHRTPTRGGIRYSM
                 60        70        80        90       100       110       120       130

                                                  30
QUERY  -------------------------------------QYTDYELEK
                                             :::::: ::
gi|253 DVCEDEVKALSALMTYKCAAVDVPFGGAKGGVKIDPKQYTDYEIEKITRRIAIEFAKKGFLGPGVDVPAPDMGTGEREMG
              140       150       160       170       180       190       200       210

gi|253 WIADTYAQTIGHLDRDASACITGKPIVAGGIHGRVSATGRGVWKGLEVFTKEPEYMNKVGLSLGLEGKTIITQGFGNVGL
              220       230       240       250       260       270       280       290
```

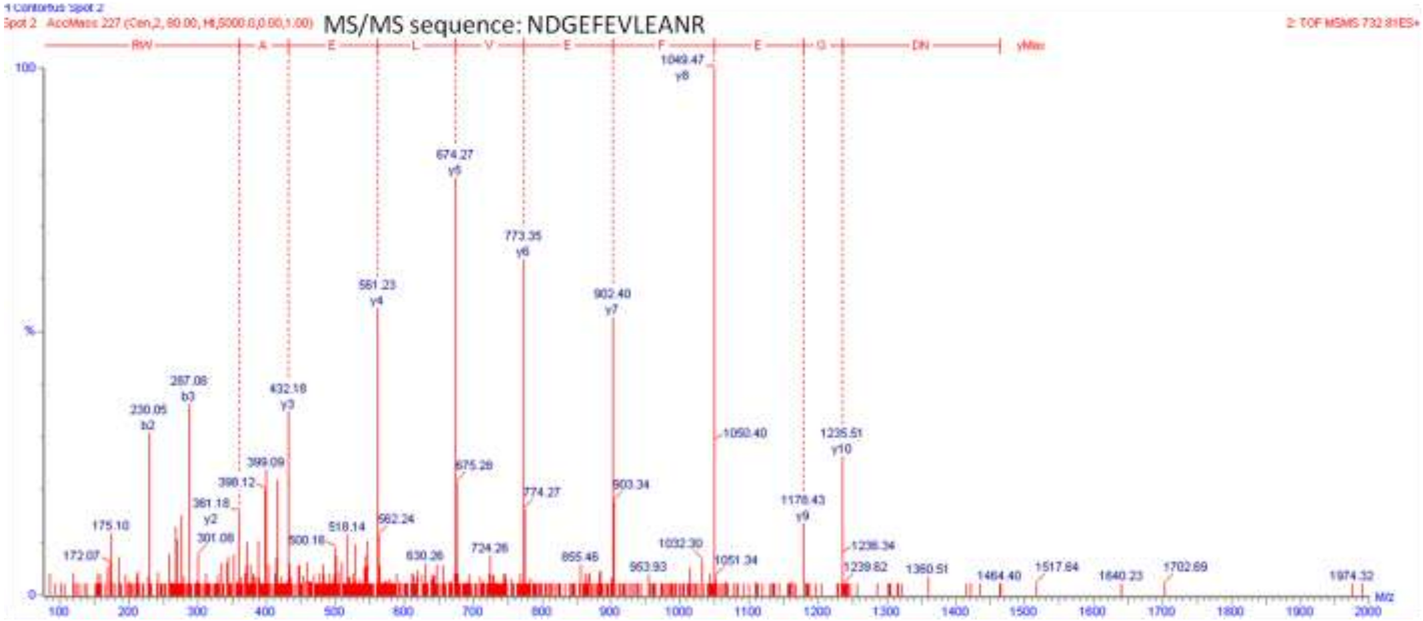

Figure S2.17.

Spot 33 MS/MS sequence 1
Ion 581.38

```
>>>QUERY, 38 aa vs N library

>>gi|31580769|gb|AAP51177.1| fumarase [Ascaris suum]                (467 aa)
 initn: 248 init1: 148 opt: 248  Z-score: 58.6  bits: 72.7 E(13331964): 3e-11
Smith-Waterman score: 248; 80.6% identity (83.3% similar) in 36 aa overlap (1-36:12-263)
Entrez Lookup  Re-search database  General re-search
                          10
QUERY             DTFGELEVWPR---------------------------------------------------------
                  :::::::::
gi|315 MATTQKIRKERDTFGELEVPADCYYGAQTARSKMNFKIGGPEERMPLPVIHAFGILKKAAAQVNQEFGLDAKLAKAICQA
                10        20        30        40        50        60        70        80


QUERY  --------------------------------------------------------------------------------

gi|315 ADEVIEGKLDEHFPLVVWQTGSGTQSNMNVNEVISNRAIEILGGELGSKKPVHPNDHVNMSQSSNDTYPTAMHIAVAREV
                90       100       110       120       130       140       150       160

                                                                              20
QUERY  --------------------------------------------------------------------LAAGGTAVGTGL
                                                                    ::::::::::::
gi|315 NSRLLPSLKQLRDSLAKKSKEFDKIIKIGRTHTQDAVPLTLGQEFSGYVQQMDNGIARVEATLPHLYKLAAGGTAVGTGL
               170       180       190       200       210       220       230       240

                         30
QUERY  NTR-----------VTLWTGLPFK
       :::           :.  :::::
gi|315 NTRIGFAEKVAAKVSELTGLPFETAPNKFEALAGHDAMVEVHGALNTVAVSLMKIANDIRFLGSGPRCGLGELSLPENEP
               250       260       270       280       290       300       310       320
```

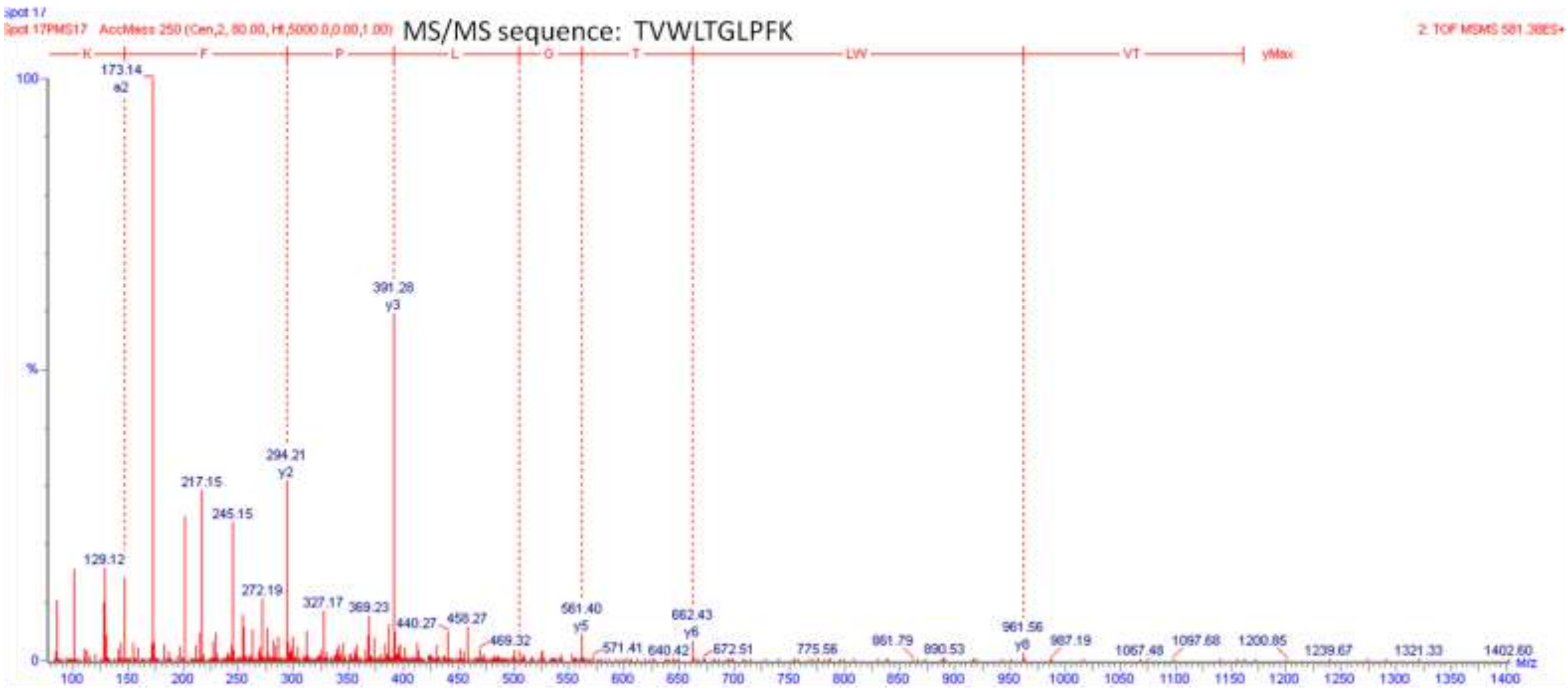

Figure S2.18.

Spot 33 MS/MS sequence 2
Ion 674.88

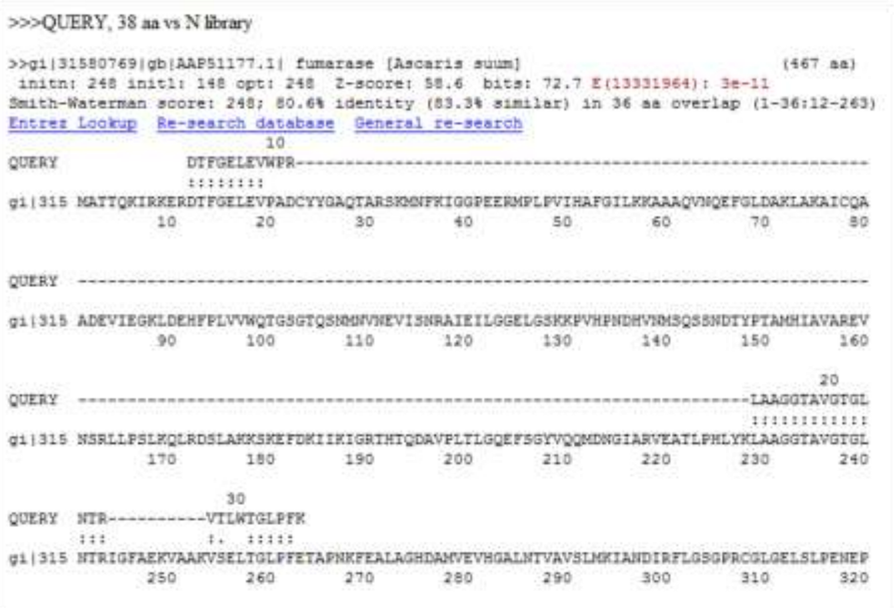

```
>>>QUERY, 38 aa vs N library

>>gi|31580769|gb|AAP51177.1| fumarase [Ascaris suum]                    (467 aa)
 initn: 248 initl: 148 opt: 248  Z-score: 58.6  bits: 72.7 E(13331964): 3e-11
Smith-Waterman score: 248; 80.6% identity (83.3% similar) in 36 aa overlap (1-36:12-263)
Entrez Lookup  Re-search database  General re-search
                         10
QUERY             DTFGELEVWPR-----------------------------------------------------------
                  ::::::::
gi|315 MATTQKIRKERDTFGELEVPADCYYGAQTARSKMNFKIGGPEERMPLPVIHAFGILKKAAAQVNQEFGLDAKLAKAICQA
               10        20        30        40        50        60        70        80


QUERY  --------------------------------------------------------------------------------

gi|315 ADEVIEGKLDEHFPLVVWQTGSGTQSNMNVNEVISNRAIEILGGELGSKKPVHPNDHVNMSQSSNDTYPTAMHIAVAREV
               90       100       110       120       130       140       150       160

                                                                                 20
QUERY  --------------------------------------------------------------------LAAGGTAVGTGL
                                                                       ::::::::::::
gi|315 NSRLLPSLKQLRDSLAKKSKEFDKIIKIGRTHTQDAVPLTLGQEFSGYVQQMDNGIARVEATLPHLYKLAAGGTAVGTGL
              170       180       190       200       210       220       230       240

                    30
QUERY  NTR----------VTLWTGLPFK
       :::          :.  :::::
gi|315 NTRIGFAEKVAAKVSELTGLPFETAPNKFEALAGHDAMVEVHGALNTVAVSLMKIANDIRFLGSGPRCGLGELSLPENEP
              250       260       270       280       290       300       310       320
```

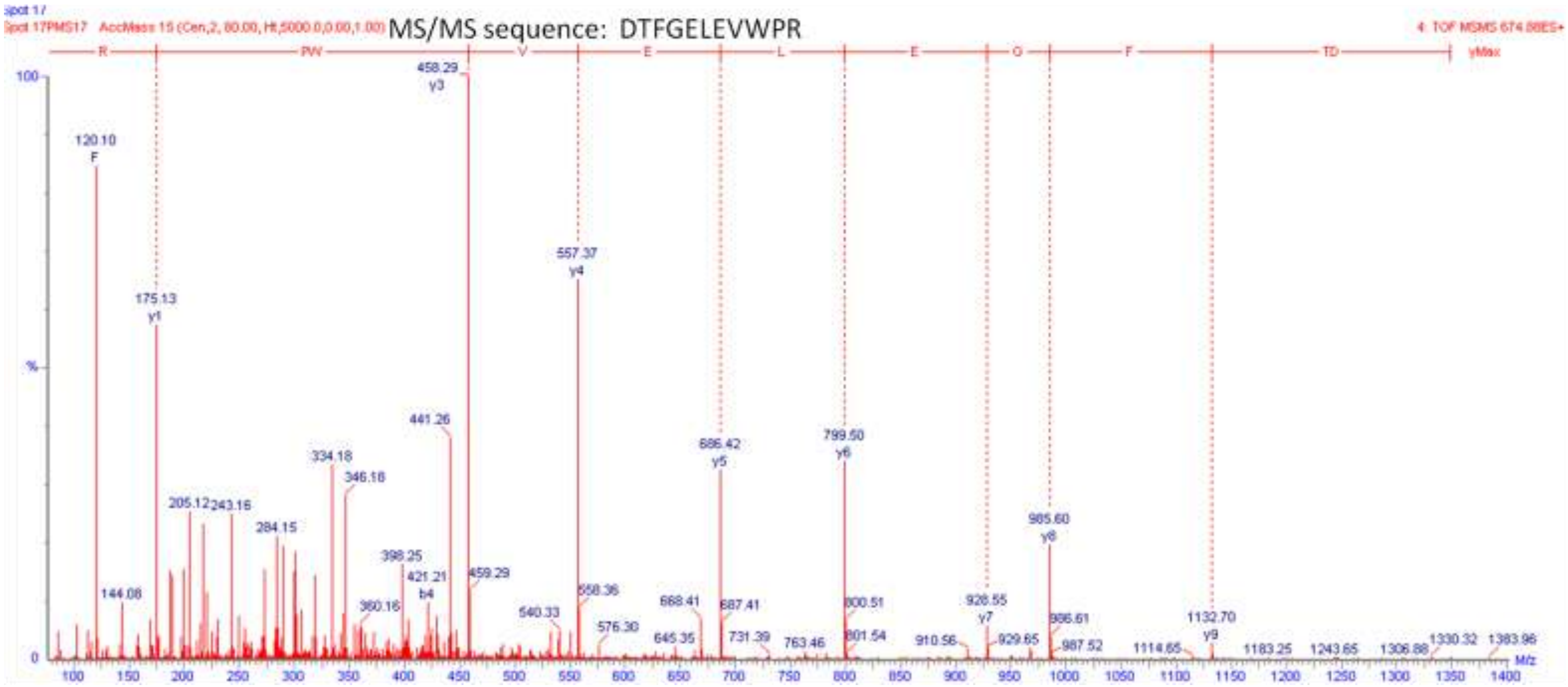

Figure S2.19.

Spot 33 MS/MS sequence 3
Ion 882.49

```
>>>QUERY, 38 aa vs N library

>>gi|31580769|gb|AAP51177.1| fumarase [Ascaris suum]                    (467 aa)
 initn: 248 init1: 148 opt: 248  Z-score: 58.6  bits: 72.7 E(13331964): 3e-11
Smith-Waterman score: 248; 80.6% identity (83.3% similar) in 36 aa overlap (1-36:12-263)
Entrez Lookup  Re-search database  General re-search
                          10
QUERY           DTFGELEVWPR---------------------------------------------------------
                ::::::::
gi|315 MATTQKIRKERDTFGELEVPADCYYGAQTARSKMNFKIGGPEERMPLPVIHAFGILKKAAAQVNQEFGLDAKLAKAICQA
                 10        20        30        40        50        60        70        80

QUERY  --------------------------------------------------------------------------------

gi|315 ADEVIEGKLDEHFPLVVWQTGSGTQSNMNVNEVISNRAIEILGGELGSKKPVHPNDHVNMSQSSNDTYPTAMHIAVAREV
                 90       100       110       120       130       140       150       160

                                                                                  20
QUERY  --------------------------------------------------------------------LAAGGTAVGTGL
                                                                        ::::::::::::
gi|315 NSRLLPSLKQLRDSLAKKSKEFDKIIKIGRTHTQDAVPLTLGQEFSGYVQQMDNGIARVEATLPHLYKLAAGGTAVGTGL
                170       180       190       200       210       220       230       240

                   30
QUERY  NTR----------VTLWTGLPFK
       :::          :.  :::::
gi|315 NTRIGFAEKVAAKVSELTGLPFETAPNKFEALAGHDAMVEVHGALNTVAVSLMKIANDIRFLGSGPRCGLGELSLPENEP
                250       260       270       280       290       300       310       320
```

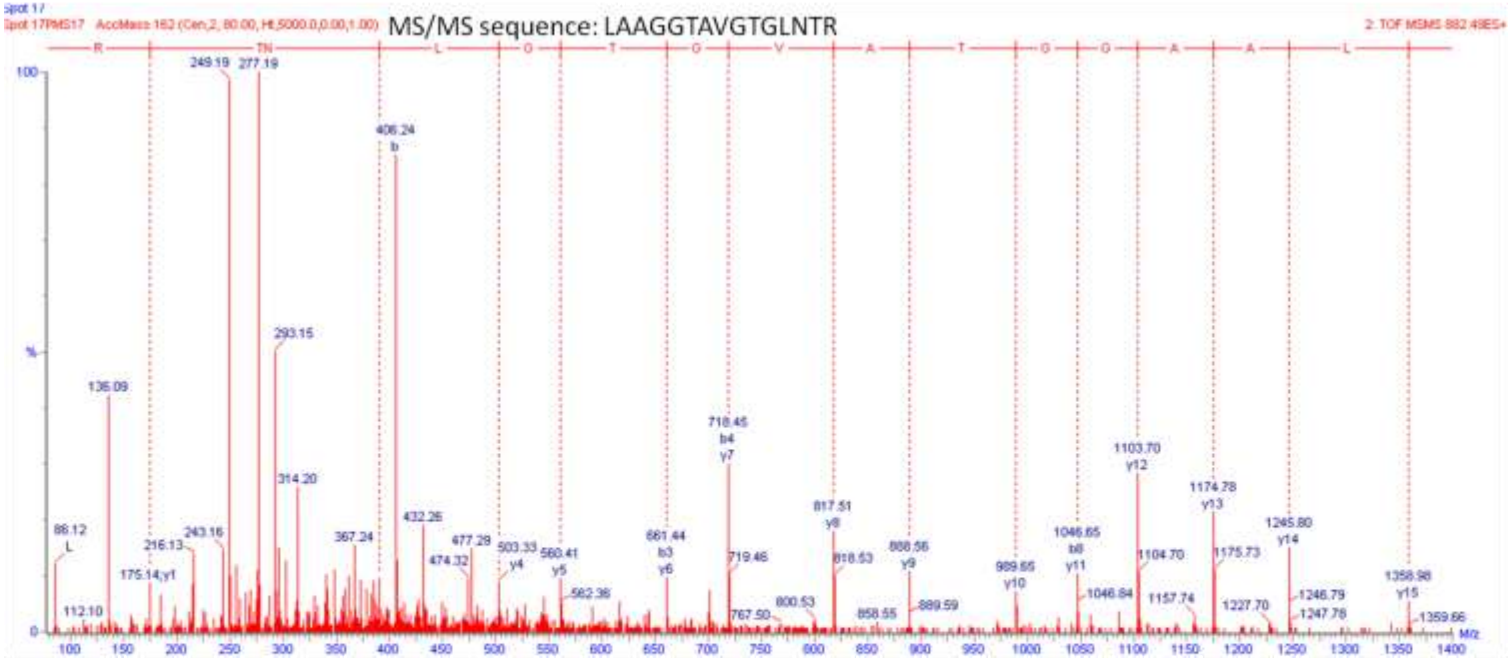

Figure S2.20.

Spot 40 MS/MS sequence 1
Ion 610.44

```
>>gi|268575912|ref|XP_002642936.1| C. briggsae CBR-MDH-1 protein [Caenorhabd  (341 aa)
 initn: 302 init1: 114 opt: 302  Z-score: 84.7  bits: 98.2 E(13331964): 4.4e-19
Smith-Waterman score: 302; 87.9% identity (87.9% similar) in 33 aa overlap (1-33:96-180)
Entrez Lookup  Re-search database  General re-search
                                                         10                  20
QUERY                                               NADVLVLPAGVPR---------FNTNAGLVR---------
                                                    :::: : ::::::         :::::: ::
gi|268 HLALYDVVNTPGVAADLSHIDSNAKVTAHTGPKELYAAVENADVIVIPAGVPRKPGMTRDDLFNTNAGIVRDLAAVIAKA
          60        70        80        90       100       110       120       130

                                                    30
QUERY  ----------------------------------LFGVTTLDVVR
                                          ::::::::::
gi|268 SPKALIAIITNPVNSTVPIASEVLKKAGVYDPKRVFGVTTLDVVRSQAFVSELKGHDATKTVVPVVGGHAGITIIPLLSQ
         140       150       160       170       180       190       200       210

gi|268 VTPSTKFSEEEIAKLTPRIQDAGTEVVNAKAGAGSATLSMALAGARFANALVRGIKGEKNVQCAYVASDAVKGVEYFSTP
         220       230       240       250       260       270       280       290
```

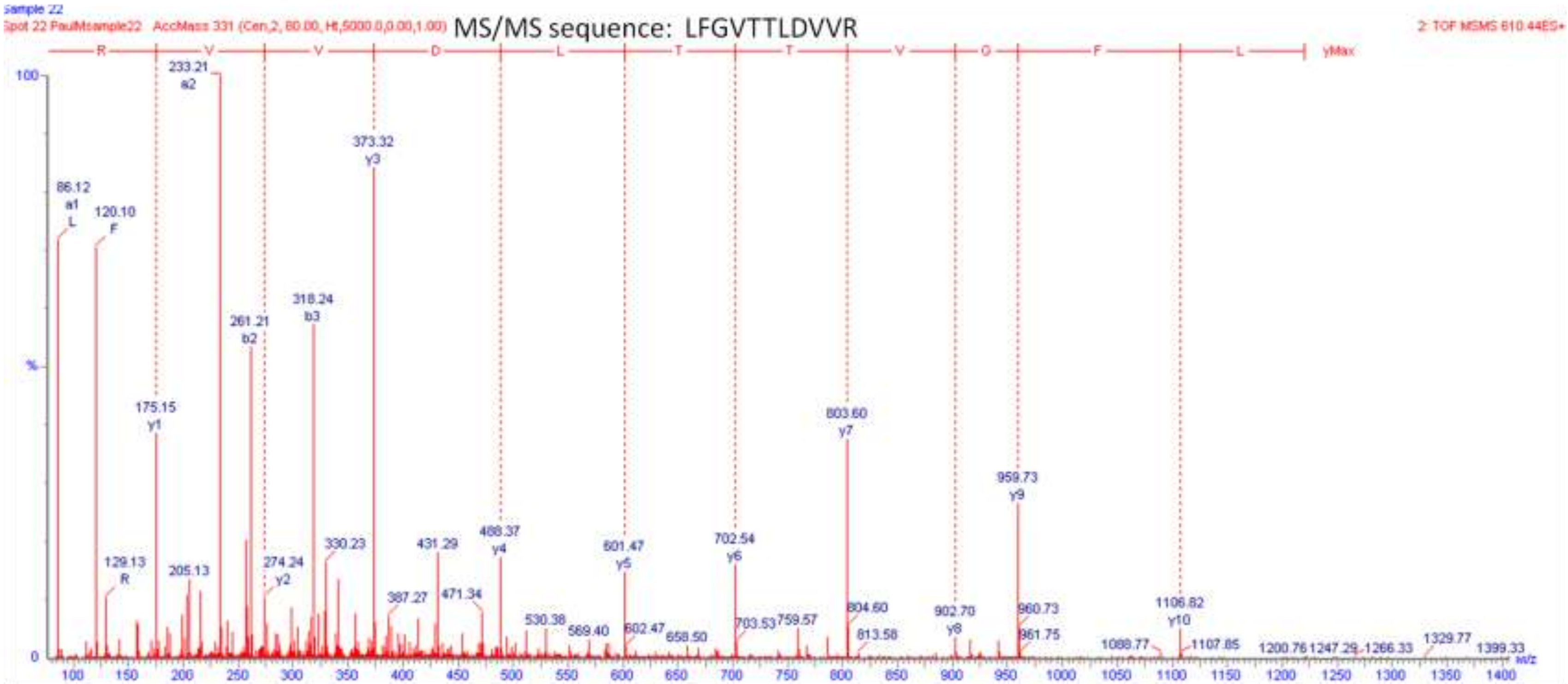

Figure S2.21.

Spot 40 MS/MS sequence 2
Ion 667.90

```
>>gi|268575912|ref|XP_002642936.1| C. briggsae CBR-MDH-1 protein [Caenorhabd  (341 aa)
 initn: 302 initl: 114 opt: 302  Z-score: 84.7  bits: 98.2 E(13331964): 4.4e-19
Smith-Waterman score: 302; 87.9% identity (87.9% similar) in 33 aa overlap (1-33:96-180)
Entrez Lookup  Re-search database  General re-search
                                                         10                  20
QUERY                                                NADVLVLPAGVPR---------FNTNAGLVR---------
                                                     :::: : ::::::         :::::: ::
gi|268 HLALYDVVNTPGVAADLSHIDSNAKVTAHTGPKELYAAVENADVIVIPAGVPRKPGMTRDDLFNTNAGIVRDLAAVIAKA
             60        70        80        90       100       110       120       130

                                              30
QUERY  ----------------------------------LFGVTILDVVR
                                          ::::::::::
gi|268 SPKALIAIITNPVNSTVPIASEVLKKAGVYDPKRVFGVTILDVVRSQAFVSELKGHDATKTVVPVVGGHAGITIIPLLSQ
            140       150       160       170       180       190       200       210

gi|268 VTPSTKFSEEEIAKLTPRIQDAGTEVVNAKAGAGSATLSMALAGARFANALVRGIKGEKNVQCAYVASDAVKGVEYFSTP
            220       230       240       250       260       270       280       290
```

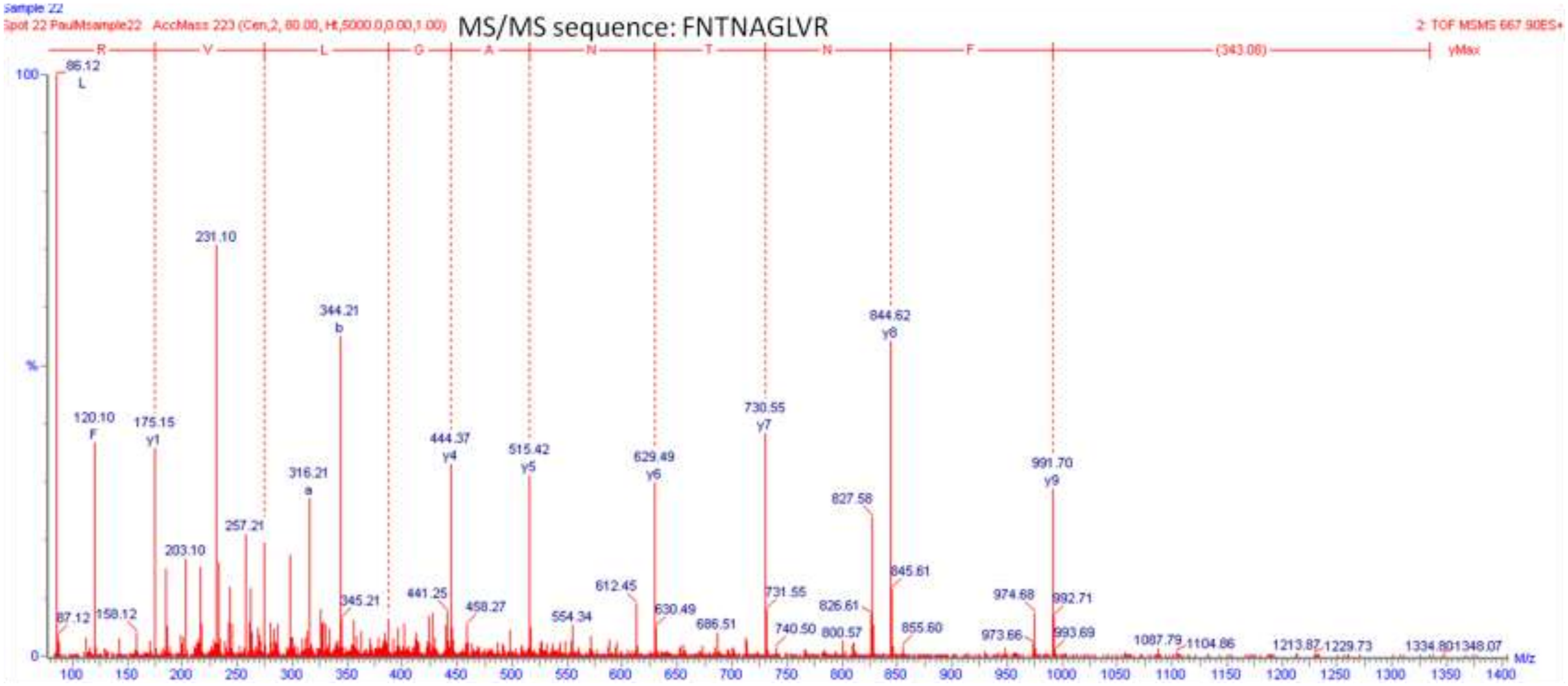

Figure S2.22.

Spot 40 MS/MS sequence 3
Ion 974.69

```
>>gi|268575912|ref|XP_002642936.1| C. briggsae CBR-MDH-1 protein [Caenorhabd  (341 aa)
 initn: 302 init1: 114 opt: 302  Z-score: 84.7  bits: 98.2 E(13331964): 4.4e-19
Smith-Waterman score: 302; 87.9% identity (87.9% similar) in 33 aa overlap (1-33:96-180)
Entrez Lookup  Re-search database  General re-search
                                                           10                  20
QUERY                                                  NADVLVLPAGVPR---------FNTNAGLVR---------
                                                       :::: : ::::::         :::::: ::
gi|268 HLALYDVVNTPGVAADLSHIDSNAKVTAHTGPKELYAAVENADVIVIPAGVPRKPGMTRDDLFNTNAGIVRDLAAVIAKA
              60        70        80        90       100       110       120       130

                                                   30
QUERY  ----------------------------------LFGVTTLDVVR
                                          ::::::::::
gi|268 SPKALIAIITNPVNSTVPIASEVLKKAGVYDPKRVFGVTTLDVVRSQAFVSELKGHDATKTVVPVVGGHAGITIIPLLSQ
             140       150       160       170       180       190       200       210

gi|268 VTPSTKFSEEEIAKLTPRIQDAGTEVVNAKAGAGSATLSMALAGARFANALVRGIKGEKNVQCAYVASDAVKGVEYFSTP
             220       230       240       250       260       270       280       290
```

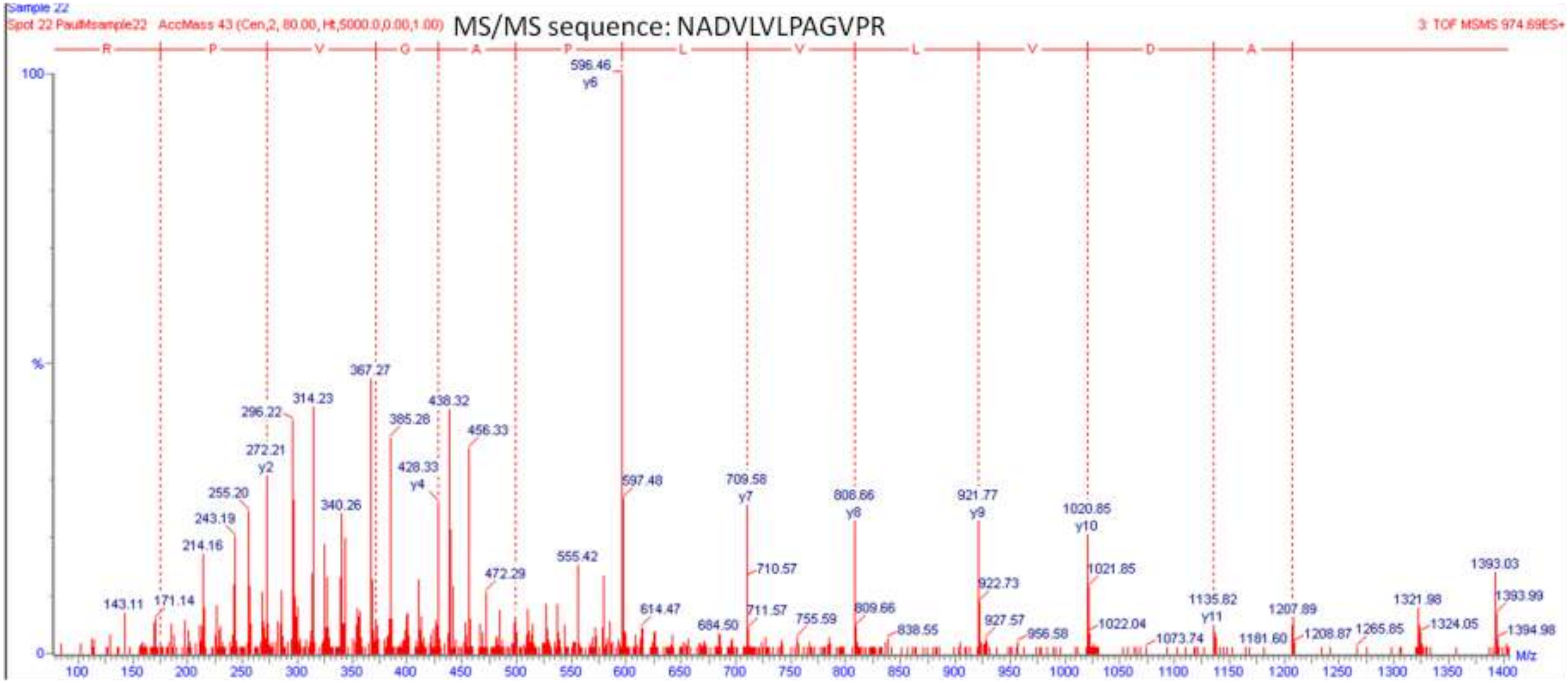

Figure S2.23.

```
>>gi|155675706|gb|ABU25173.1| malate dehydrogenase [Leishmania guyanensis] g  (317 aa)
 initn: 164 init1: 117 opt: 164  Z-score: 40.8  bits: 54.3 E(13605827): 7e-06
Smith-Waterman score: 164; 64.7% identity (67.6% similar) in 34 aa overlap (1-34:94-184)
Entrez Lookup  Re-search database  General re-search
                                                                         10
QUERY                                                            DDLFNTNAGLVR---------------------------
                                                                 ::::::::: ::
gi|155 HICSSAKVTGYSQEELNKAVQNTDLVLIPAGVPRKPGMTRDDLFNTNAGIVRDLVTAVARAAPKAIIGVISNPVNSTVPV
               60        70        80        90       100       110       120       130

                                   20                            30
QUERY  ----------------LFGTTLDVVR-------------LDVDVLAVEAPK
                           :::::::              :.:: :
gi|155 AAETLKKLGAYDPGRLFGVTTLDVVRARTFVAEALGRSPYDIDVPVVGGHSGETIVPLLSGFPSLSKEQVEQLTYRIQFG
              140       150       160       170       180       190       200       210

gi|155 GDEVVKAKSGAGSATLSMAHAGNEWATAVLRALSGEKGVTVCTYVESSVEPSCTFFSSSVELGKNGVEKIHCLPKLNAYE
              220       230       240       250       260       270       280       290
```

Spot 42 MS/MS sequence 1
Ion 610.30

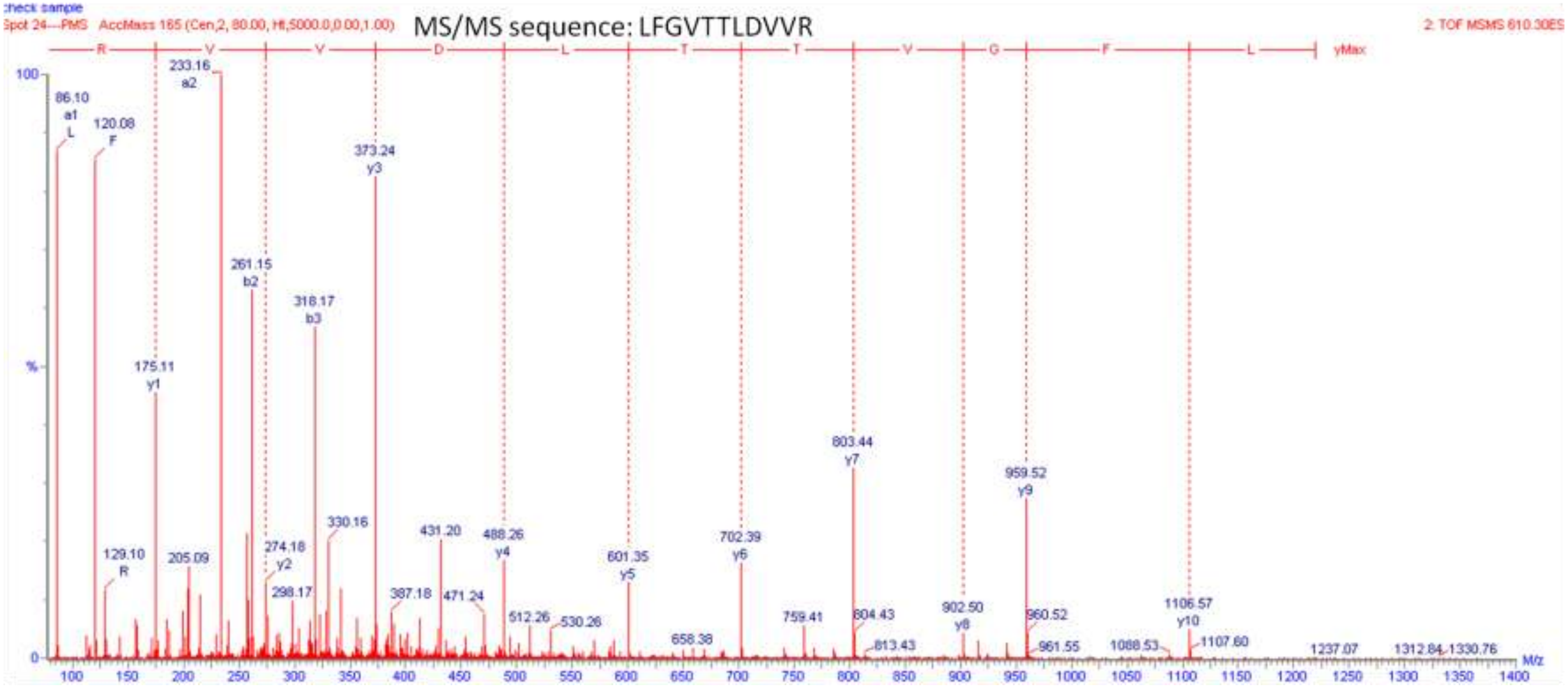

Figure S2.24

Spot 42 MS/MS sequence 2
Ion 634.85

```
>>gi|155675706|gb|ABU25173.1| malate dehydrogenase [Leishmania guyanensis] g  (317 aa)
 initn: 164 init1: 117 opt: 164  Z-score: 40.8  bits: 54.3 E(13605827): 7e-06
Smith-Waterman score: 164; 64.7% identity (67.6% similar) in 34 aa overlap (1-34:94-184)
Entrez Lookup  Re-search database  General re-search
                                                        10
QUERY                                           DDLFNTNAGLVR----------------------------
                                                ::::::::: ::
gi|155 HICSSAKVTGYSQEELNKAVQNTDLVLIPAGVPRKPGMTRDDLFNTNAGIVRDLVTAVARAAPKAIIGVISNPVNSTVPV
             60        70        80        90       100       110       120       130

                                20                            30
QUERY  ----------------LFGTTLDVVR-------------LDVDVLAVEAPK
                           :::::::             :.::  :
gi|155 AAETLKKLGAYDPGRLFGVTTLDVVRARTFVAEALGRSPYDIDVPVVGGHSGETIVPLLSGFPSLSKEQVEQLTYRIQFG
             140       150       160       170       180       190       200       210

gi|155 GDEVVKAKSGAGSATLSMAHAGNEWATAVLRALSGEKGVTVCTYVESSVEPSCTFFSSSVELGKNGVEKIHCLPKLNAYE
             220       230       240       250       260       270       280       290
```

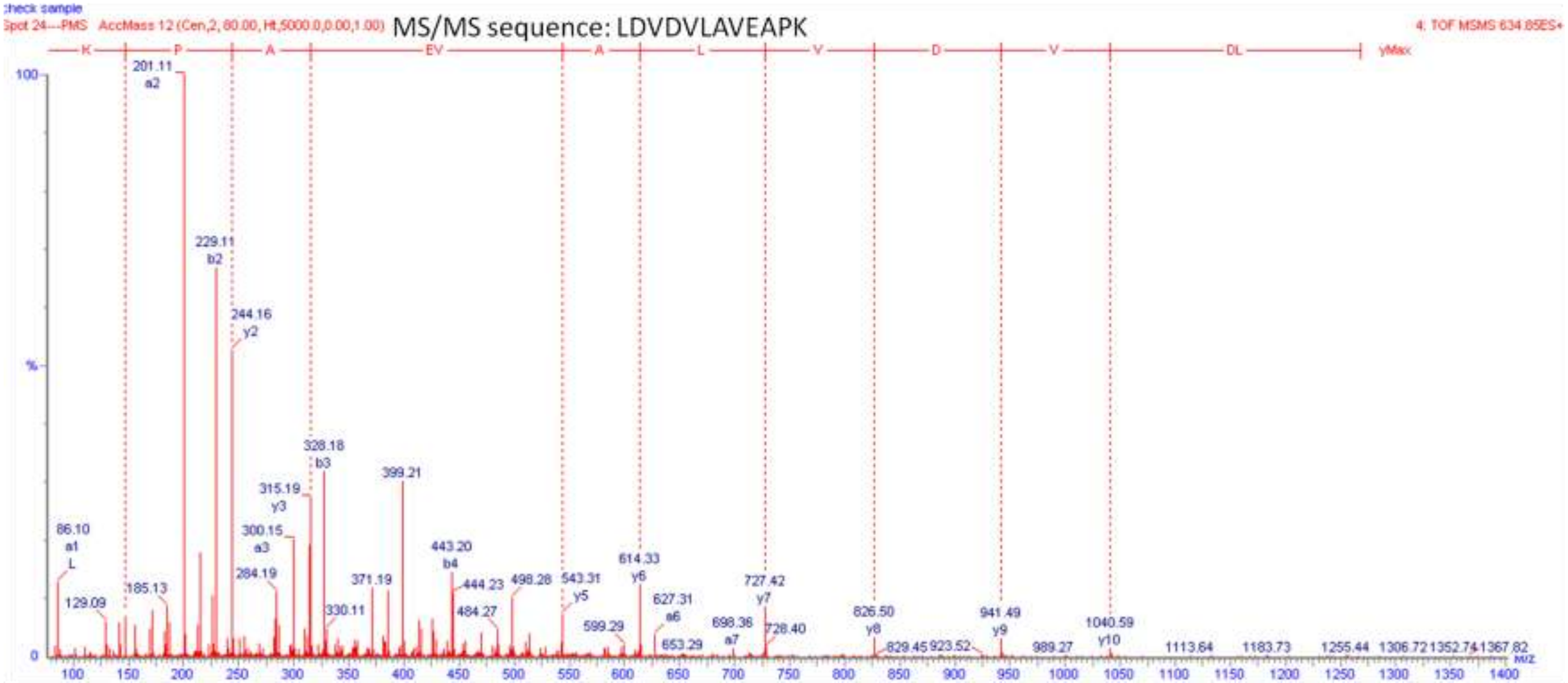

Figure S2.25.

Spot 42 MS/MS sequence 3
Ion 667.77

```
>>gi|155675706|gb|ABU25173.1| malate dehydrogenase [Leishmania guyanensis] g  (317 aa)
 initn: 164 init1: 117 opt: 164  Z-score: 40.8  bits: 54.3 E(13605827): 7e-06
Smith-Waterman score: 164; 64.7% identity (67.6% similar) in 34 aa overlap (1-34:94-184)
Entrez Lookup  Re-search database  General re-search
                                                           10
QUERY                                              DDLFNTNAGLVR----------------------------
                                                   ::::::::: ::
gi|155 HICSSAKVTGYSQEELNKAVQNTDLVLIPAGVPRKPGMTRDDLFNTNAGIVRDLVTAVARAAPKAIIGVISNPVNSTVPV
             60        70        80        90       100       110       120       130

                                20                       30
QUERY  ----------------LFGTTLDVVR-------------LDVDVLAVEAPK
                           :::::::             :.::  :
gi|155 AAETLKKLGAYDPGRLFGVTTLDVVRARTFVAEALGRSPYDIDVPVVGGHSGETIVPLLSGFPSLSKEQVEQLTYRIQFG
            140       150       160       170       180       190       200       210

gi|155 GDEVVKAKSGAGSATLSMAHAGNEWATAVLRALSGEKGVTVCTYVESSVEPSCTFFSSSVELGKNGVEKIHCLPKLNAYE
            220       230       240       250       260       270       280       290
```

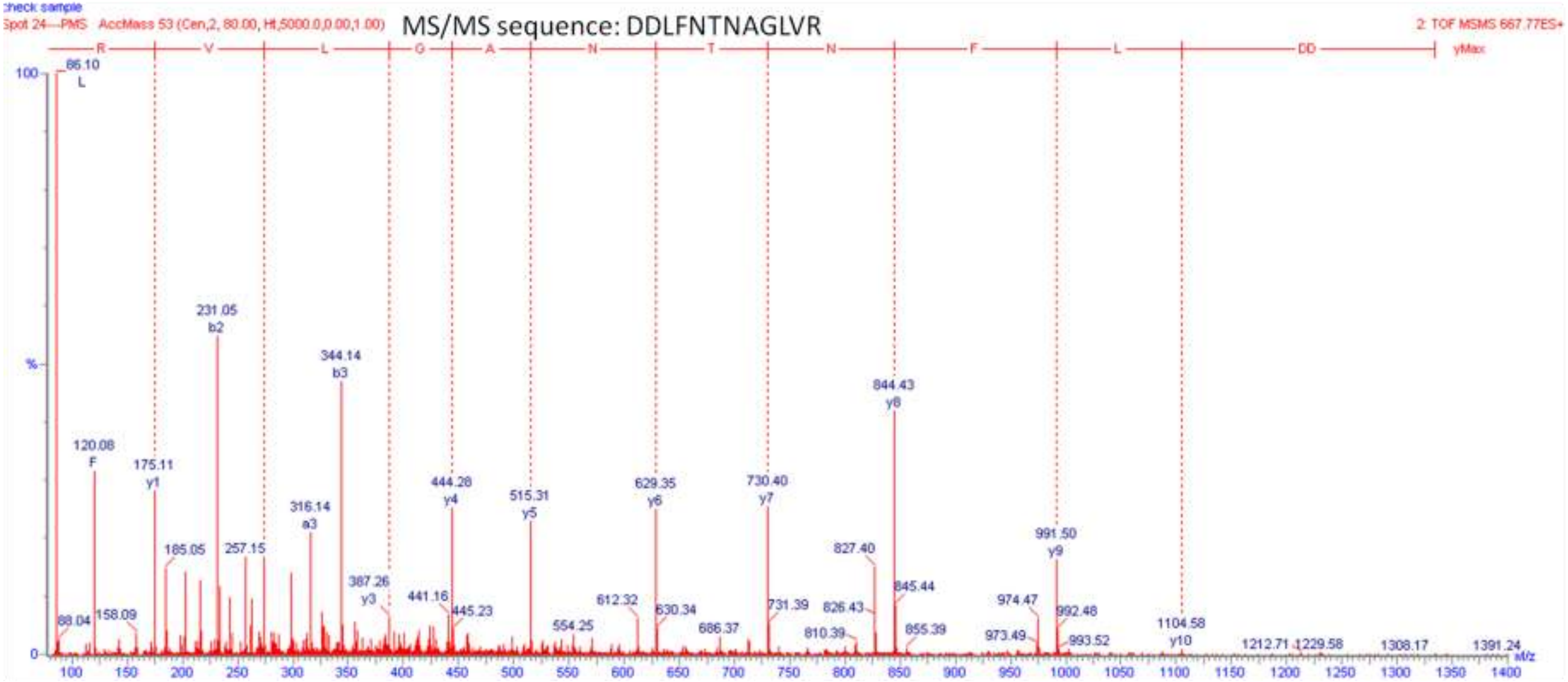

Figure S2.26.

Spot 51 MS/MS sequence 1
Ion 597.93

```
>>>QUERY, 30 aa vs N library

>>gi|68146615|emb|CAJ09947.1| NIM-1 protein [Haemonchus contortus]          (178 aa)
 initn: 306 init1: 177 opt: 306  Z-score: 104.5  bits: 116.9 E(13605827): 4.7e-25
Smith-Waterman score: 306; 100.0% identity (100.0% similar) in 29 aa overlap (1-29:53-87)
Entrez Lookup  Re-search database  General re-search
                                                           10                  20
QUERY                                               FDVSKDNTATQK------SPLVTAAMPLAGVDNEK
                                                    ::::::::::::      :::::::::::::::::
gi|681  FAAATTCESDSVDGTWFFLMDTAVVSHAHGGEELNPKYNCFDVSKDNTATQKFRVDLKSPLVTAAMPLAGVDNEKYCVDK
                20        30        40        50        60        70        80        90

gi|681  QLRTEIAEAVKDAEDSCEGHVKALASKLNRPGWAMNCVSRSPKGFNFGFFGTDQNFCHFDAVKGGKVYSLTVVKLDKSEP
               100       110       120       130       140       150       160       170
```

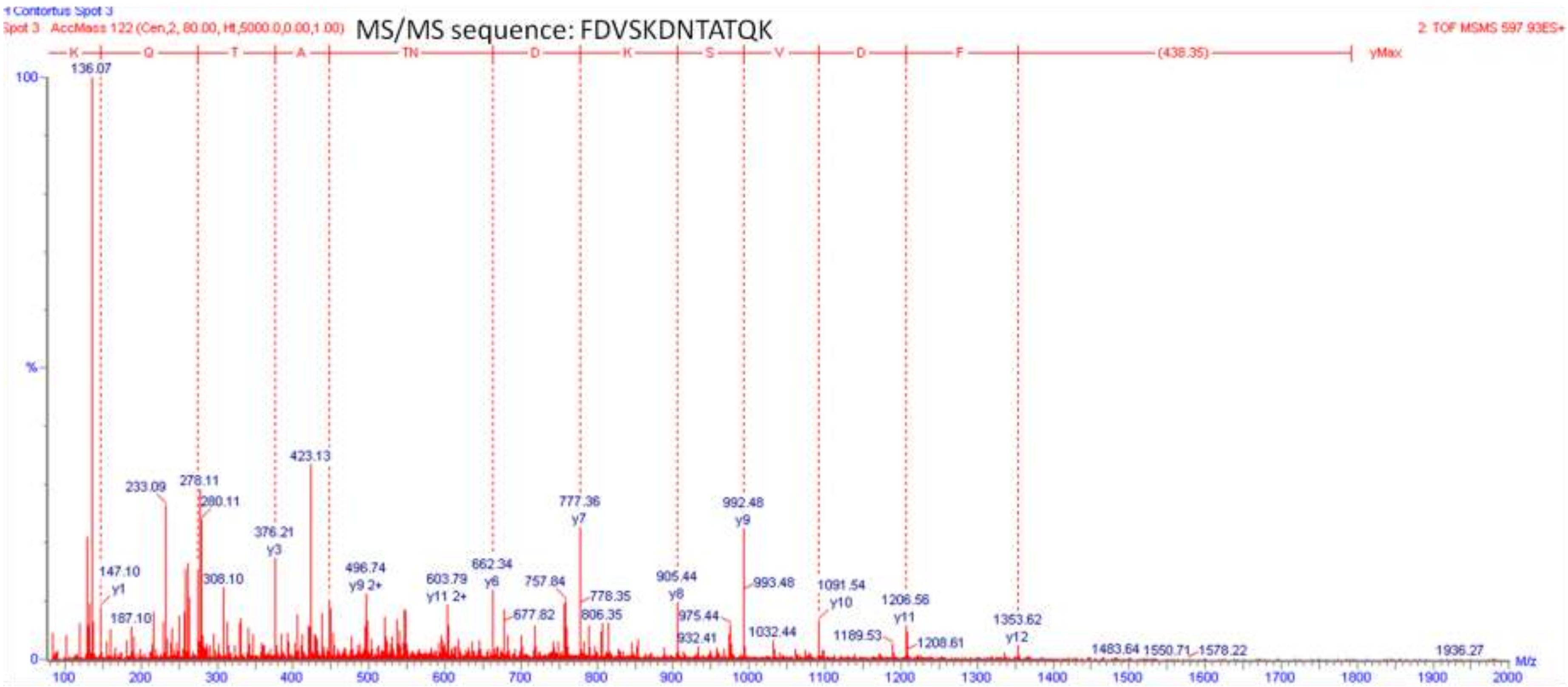

Figure S2.27.

Spot 51 MS/MS sequence 2

Ion 856.94

```
>>>QUERY, 30 aa vs N library

>>gi|68146615|emb|CAJ09947.1| NIM-1 protein [Haemonchus contortus]          (178 aa)
 initn: 306 init1: 177 opt: 306  Z-score: 104.5  bits: 116.9 E(13605827): 4.7e-25
Smith-Waterman score: 306; 100.0% identity (100.0% similar) in 29 aa overlap (1-29:53-87)
Entrez Lookup   Re-search database   General re-search
                                                          10                20
QUERY                                                 FDVSKDNTATQK------SPLVTAAMPLAGVDNEK
                                                      ::::::::::::      :::::::::::::::::
gi|681 FAAATTCESDSVDGTWFFLMDTAVVSHAHGGEELNPKYNCFDVSKDNTATQKFRVDLKSPLVTAAMPLAGVDNEKYCVDK
              20        30        40        50        60        70        80        90

gi|681 QLRTEIAEAVKDAEDSCEGHVKALASKLNRPGWAMNCVSRSPKGFNFGFFGTDQNFCHFDAVKGGKVYSLTVVKLDKSEP
             100       110       120       130       140       150       160       170
```

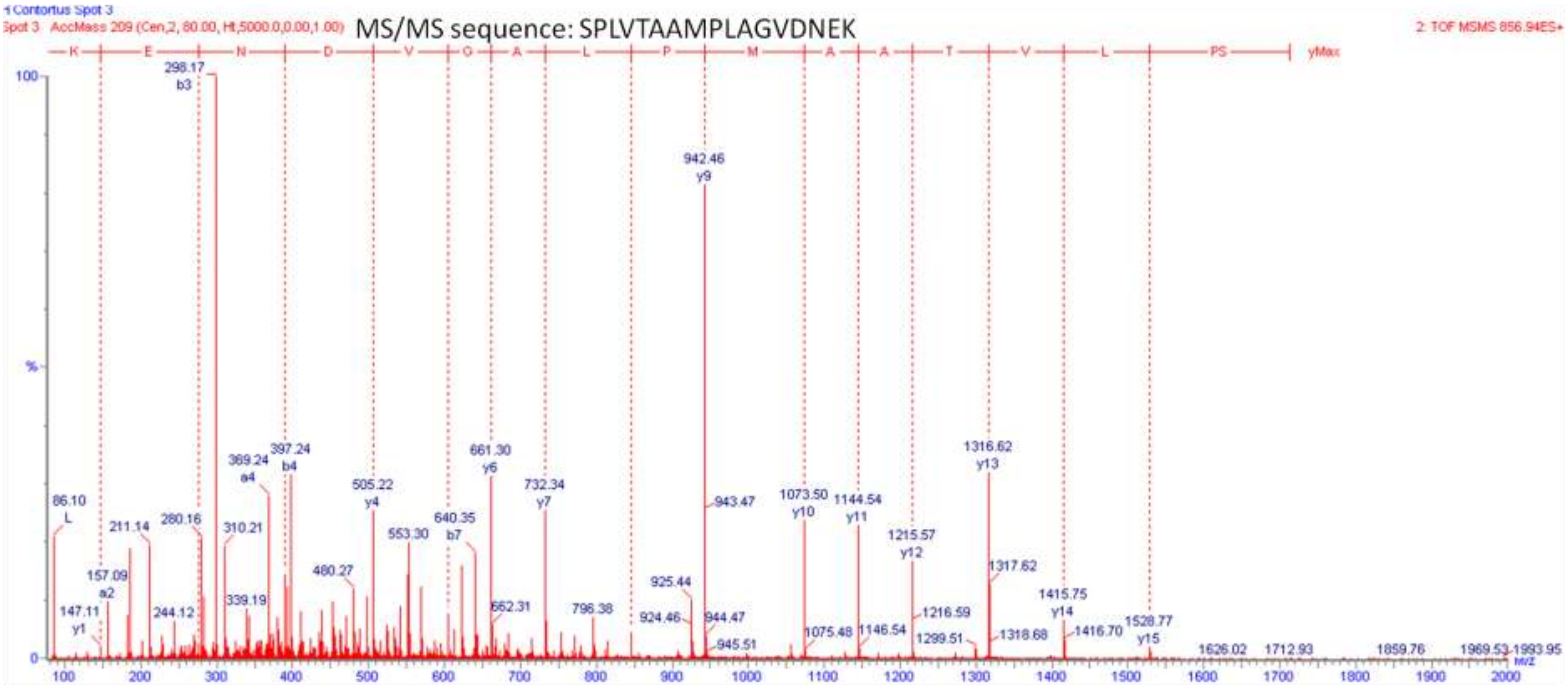

Figure S2.28.

Spot 56 MS/MS sequence 1

Ion 494.94

```
>>>QUERY, 39 aa vs N library

>>gi|253721983|gb|ACT34055.1| glutamate dehydrogenase [Haemonchus contortus]  (532 aa)
 initn: 253 init1: 142 opt: 253  Z-score: 59.1  bits: 73.5 E(13331964): 2.1e-11
Smith-Waterman score: 253; 78.4% identity (81.1% similar) in 37 aa overlap (1-37:381-475)
Entrez Lookup  Re-search database  General re-search
                                                      10
QUERY                                          LLAEAANGPTTPAADK------------------------
                                                 ::::::::::::::
gi|253 PKAKNFEPFAELMYEPCDIFVPAACEKAIHKENANRIQAKIIAEAANGPTTPAADKILLERGNCLIIPDMFINSGGVTVS
                 350       360       370       380       390       400       410       420

                                      20                  30
QUERY  --------------------------FLLQADKDSLEK--------HPNDFETAR
                                   :::. :::::        ::::  :::
gi|253 YFEWLKNLNHVSYGRLSFKYEEDSNRMLLQSVQDSLEKALNKEAPVHPNDEFTARIAGASEKDIVHSGLEYTMTRSGEAI
                 430       440       450       460       470       480       490       500

gi|253 IRTARKYNLGLDIRTAAYANSIEKYIIPTELP
                 510       520       530
```

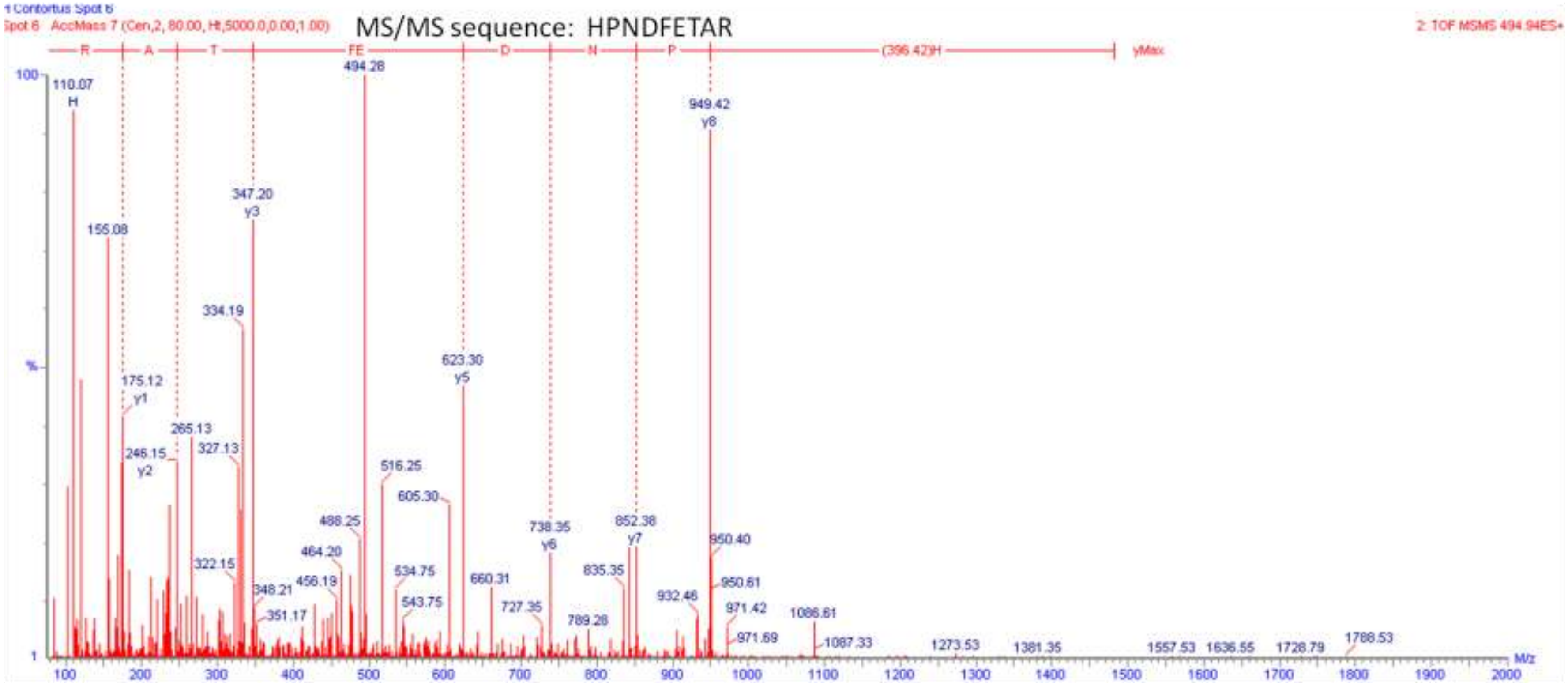

Figure S2.29.

Spot 56 MS/MS sequence 2
Ion 703.90

```
>>>QUERY, 39 aa vs N library

>>gi|253721983|gb|ACT34055.1| glutamate dehydrogenase [Haemonchus contortus]  (532 aa)
 initn: 253 init1: 142 opt: 253  Z-score: 59.1  bits: 73.5 E(13331964): 2.1e-11
Smith-Waterman score: 253; 78.4% identity (81.1% similar) in 37 aa overlap (1-37:381-475)
Entrez Lookup  Re-search database  General re-search
                                                        10
QUERY                                          LLAEAANGPTTPAADK------------------------
                                                 ::::::::::::::
gi|253 PKAKNFEPFAELMYEPCDIFVPAACEKAIHKENANRIQAKIIAEAANGPTTPAADKILLERGNCLIIPDMFINSGGVTVS
              350       360       370       380       390       400       410       420

                                   20                30
QUERY  --------------------------FLLQADKDSLEK--------HPNDFETAR
                                  :::.  :::::        ::::  :::
gi|253 YFEWLKNLNHVSYGRLSFKYEEDSNRMLLQSVQDSLEKALNKEAPVHPNDEFTARIAGASEKDIVHSGLEYTMTRSGEAI
              430       440       450       460       470       480       490       500

gi|253 IRTARKYNLGLDIRTAAYANSIEKYIIPTELP
              510       520       530
```

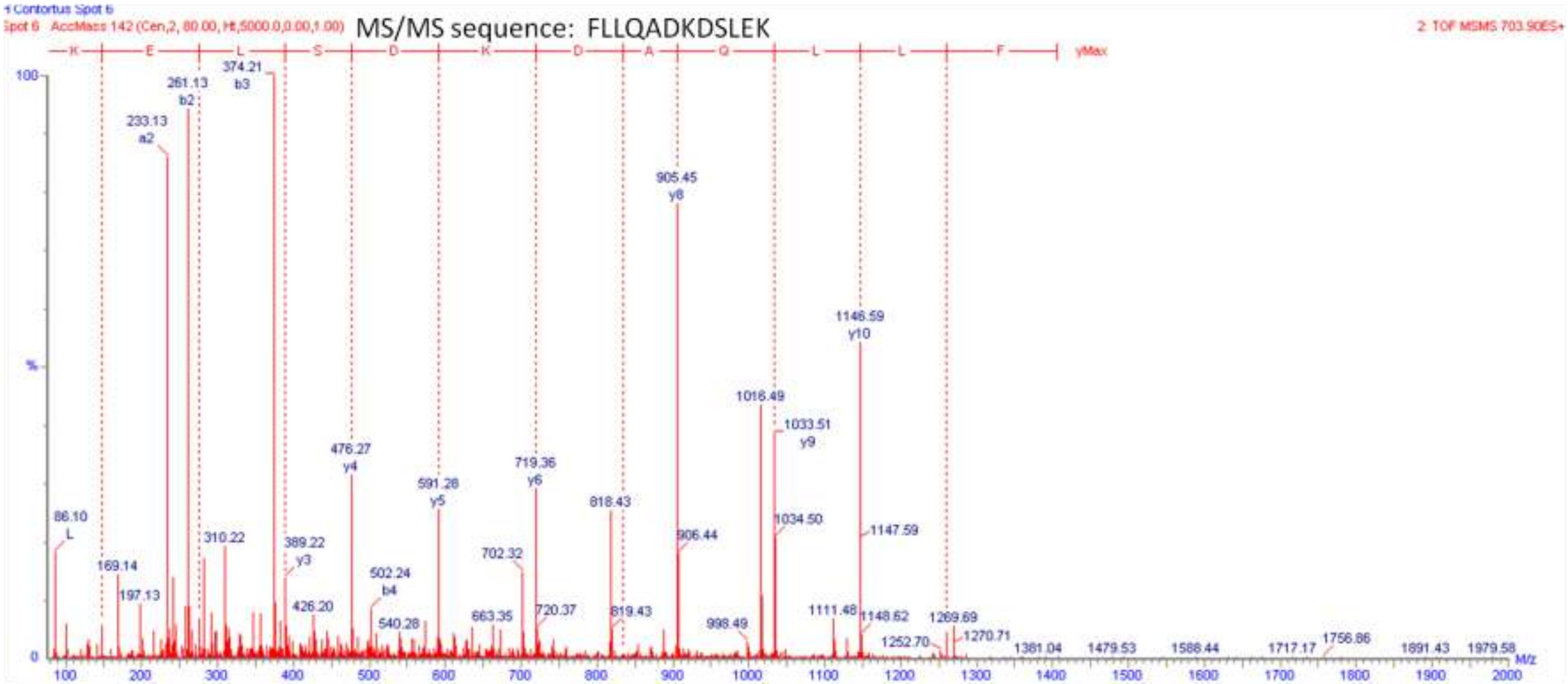

Figure S2.30.

Spot 56 MS/MS sequence 3
Ion 770.41

```
>>>QUERY, 39 aa vs N library

>>gi|253721983|gb|ACT34055.1| glutamate dehydrogenase [Haemonchus contortus]  (532 aa)
 initn: 253 init1: 142 opt: 253  Z-score: 59.1  bits: 73.5 E(13331964): 2.1e-11
Smith-Waterman score: 253; 78.4% identity (81.1% similar) in 37 aa overlap (1-37:381-475)
Entrez Lookup  Re-search database  General re-search
                                                               10
QUERY                                                   LLAEAANGPTTPAADK----------------------
                                                          ::::::::::::::
gi|253 PKAKNFEPFAELMYEPCDIFVPAACEKAIHKENANRIQAKIIAEAANGPTTPAADKILLERGNCLIIPDMFINSGGVTVS
               350       360       370       380       390       400       410       420

                                           20                  30
QUERY  --------------------------FLLQADKDSLEK--------HPNDFETAR
                                   :::.  :::::          ::::  :::
gi|253 YFEWLKNLNHVSYGRLSFKYEEDSNRMLLQSVQDSLEKALNKEAPVHPNDEFTARIAGASEKDIVHSGLEYTMTRSGEAI
               430       440       450       460       470       480       490       500

gi|253 IRTARKYNLGLDIRTAAYANSIEKYIIPTELP
               510       520       530
```

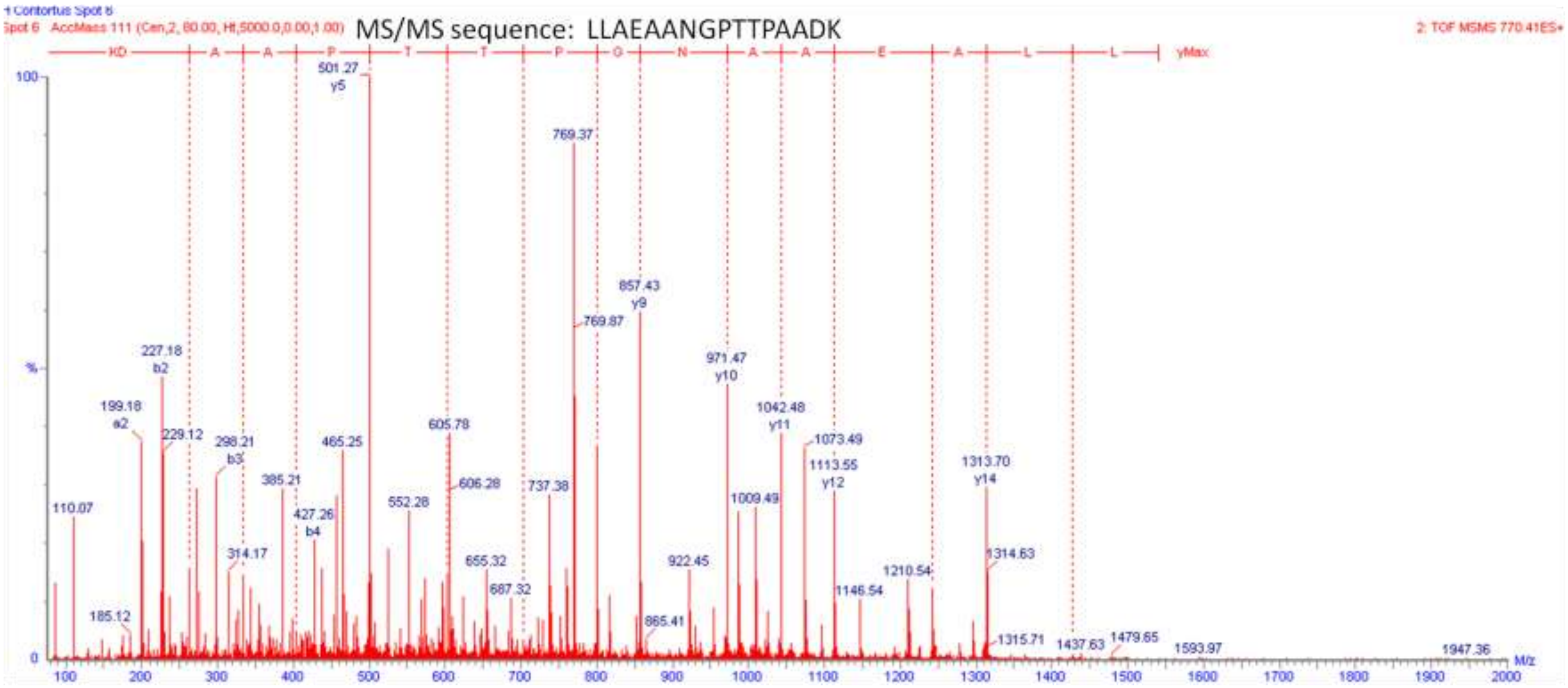

Figure S2.31.

Spot 73 MS/MS sequence 1
Ion 729.37

```
>>>QUERY, 32 aa vs N library

>>gi|308504633|ref|XP_003114500.1| hypothetical protein CRE_27196 [Caenorhab  (136 aa)
 initn: 171 init1:  74 opt: 171  Z-score: 37.8  bits: 49.9 E(13331964): 5.6e-05
Smith-Waterman score: 171; 66.7% identity (70.0% similar) in 30 aa overlap (1-30:13-61)
Entrez Lookup  Re-search database  General re-search
                            10                  20          30
QUERY              FVSSENFEAVFK--------------NLKPTLEFVK------MTSVSTFK
                   :::::::.   :                ::::::      : : ::::
gi|308 MPIQTDIVGKWNFVSSENFDEYLKEVGVGYLVRTVATKTKPTLEFAVKGDEWTMNSNSTFKNYTIITWKLGNAKDEKTADG
               10        20        30        40        50        60        70        80

gi|308 RDVSSVFNIEGDKLVQVETGKGGGKDSKIERYIENGKLVIVCTSNGVKCTRVYEKA
               90       100       110       120       130
```

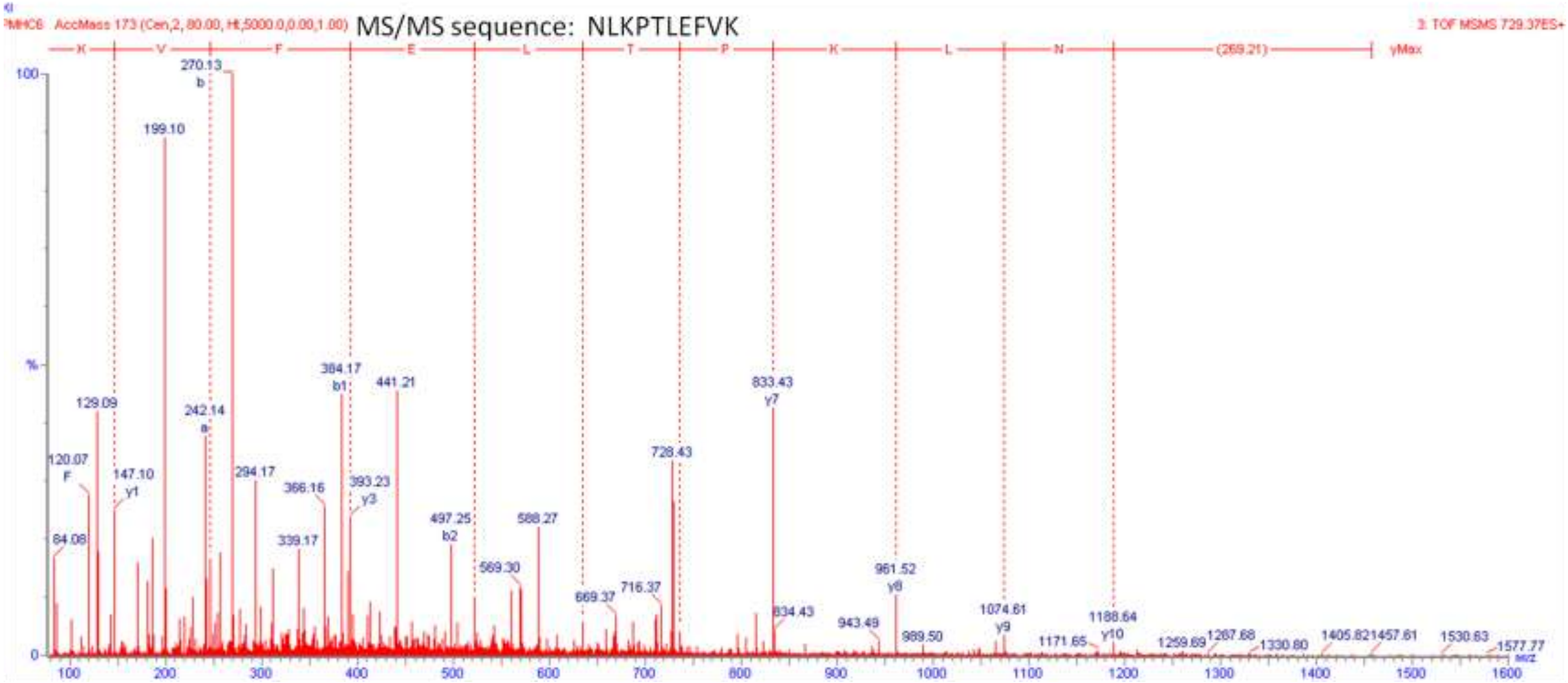

Figure S2.32.

Spot 73 MS/MS sequence 2
Ion 798.34

```
>>>QUERY, 32 aa vs N library

>>gi|308504633|ref|XP_003114500.1| hypothetical protein CRE_27196 [Caenorhab  (136 aa)
 initn: 171 init1:  74 opt: 171  Z-score: 37.8  bits: 49.9 E(13331964): 5.6e-05
Smith-Waterman score: 171; 66.7% identity (70.0% similar) in 30 aa overlap (1-30:13-61)
Entrez Lookup  Re-search database  General re-search
                           10                    20              30
QUERY              FVSSENFEAVFK-------------NLKPTLEFVK------MTSVSTFK
                   :::::::.   :               ::::::        : : ::::
gi|308 MPIQTDIVGKWNFVSSENFDEYLKEVGVGYLVRTVATKTKPTLEFAVKGDEWTMNSNSTFKNYTITWKLGNAKDEKTADG
               10        20        30        40        50        60        70        80

gi|308 RDVSSVFNIEGDKLVQVETGKGGGKDSKIERYIENGKLVIVCTSNGVKCTRVYEKA
               90       100       110       120       130
```

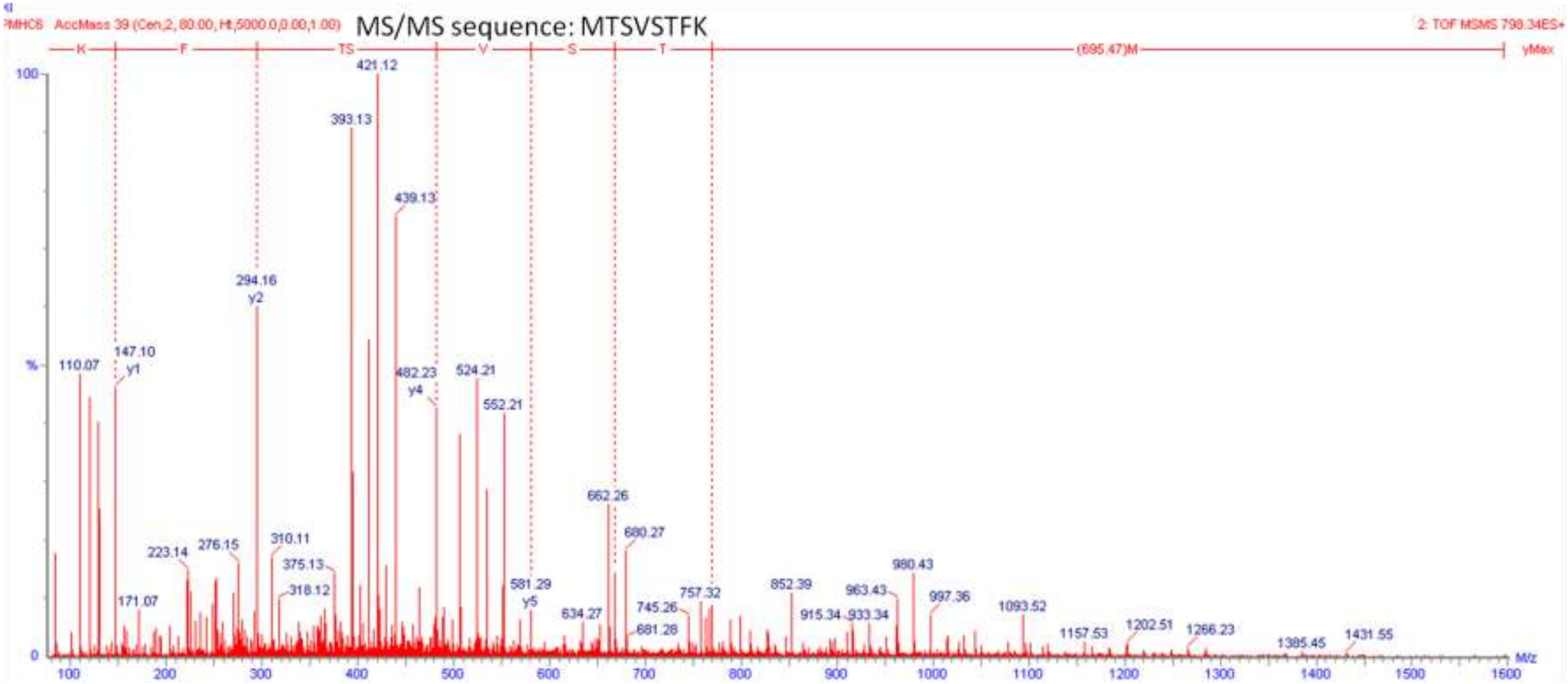

Figure S2.33.

Spot 73 MS/MS sequence 3
Ion 878.36

```
>>>QUERY, 32 aa vs N library

>>gi|308504633|ref|XP_003114500.1| hypothetical protein CRE_27196 [Caenorhab  (136 aa)
 initn: 171 init1:  74 opt: 171  Z-score: 37.8  bits: 49.9 E(13331964): 5.6e-05
Smith-Waterman score: 171; 66.7% identity (70.0% similar) in 30 aa overlap (1-30:13-61)
Entrez Lookup  Re-search database  General re-search
                           10                    20              30
QUERY             FVSSENFEAVFK-------------NLKPTLEFVK------MTSVSTFK
                  :::::::.   :             ::::::      : : ::::
gi|308 MPIQTDIVGKWNFVSSENFDEYLKEVGVGYLVRTVATKTKPTLEFAVKGDEWTMNSNSTFKNYTITWKLGNAKDEKTADG
               10        20        30        40        50        60        70        80

gi|308 RDVSSVFNIEGDKLVQVETGKGGGKDSKIERYIENGKLVIVCTSNGVKCTRVYEKA
               90       100       110       120       130
```

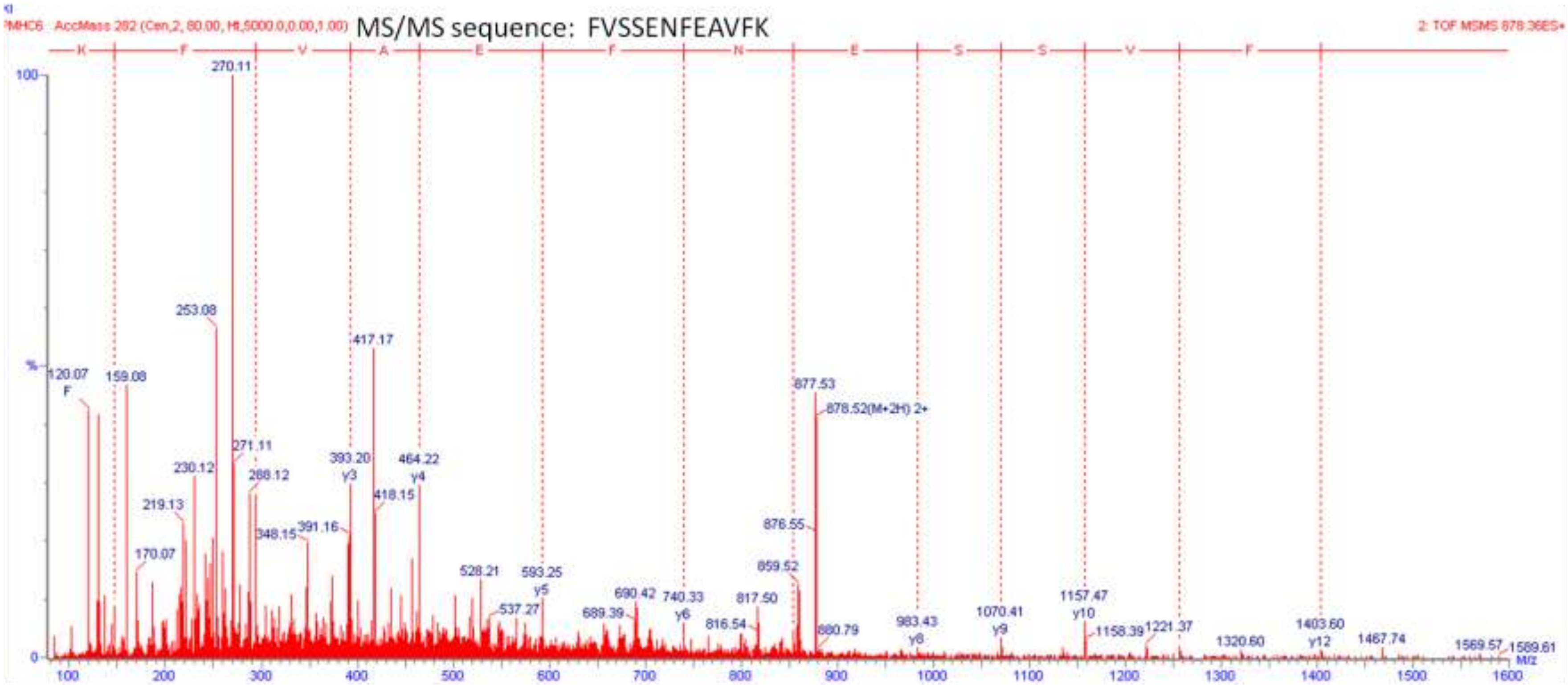

Figure S2.34.

Spot 76 MS/MS sequence 1
Ion 489.21

```
>>>QUERY, 19 aa vs N library

>>gi|324537447|gb|ADY49503.1| Peptidyl-prolyl cis-trans isomerase 3 [Ascaris  (157 aa)
 initn: 172 init1:  87 opt: 172  Z-score: 48.3  bits: 59.9 E(13331964): 3.8e-08
Smith-Waterman score: 172; 88.9% identity (94.4% similar) in 18 aa overlap (1-18:66-132)
Entrez Lookup  Re-search database  General re-search

QUERY                                                  GGESLYGEK-------------------------------
                                                       :::: ::::
gi|324 TGEKGIGKSGVPLHYKGSKFHRVIPNFMCQGGDFTRGNGTGGESIYGEKFADENFQEKHTGPGVLSMANAGPNTNGSQFF
               30        40        50        60        70        80        90       100

                         10
QUERY  ------------------VTEGMDVVK
                         ::::::.:::
gi|324 LCTVKTDWLDGKHVVFGRVTEGMNVVKEIESVGSQSGKTARDVVVADCGQLS
              110       120       130       140       150
```

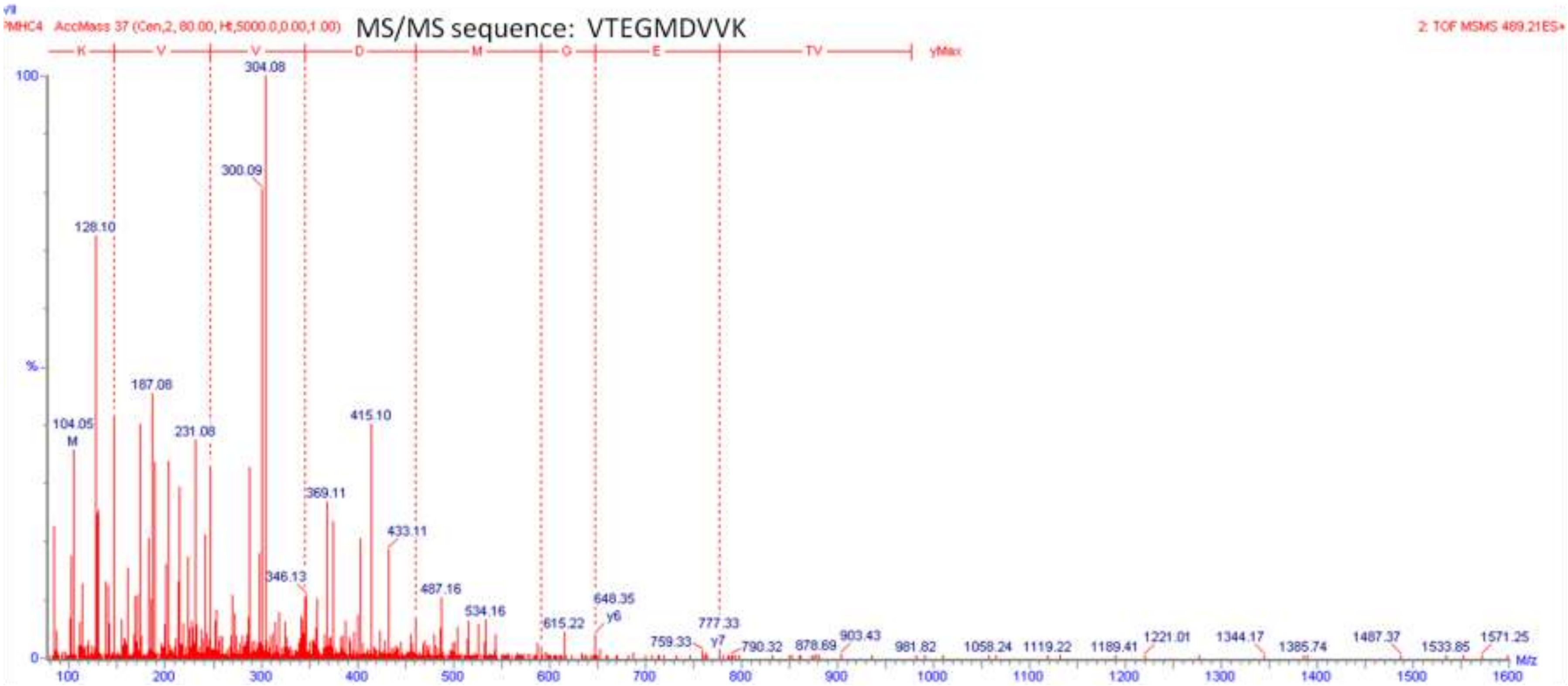

Figure S2.35.

Spot 76 MS/MS sequence 1
Ion 489.21

```
>>>QUERY, 19 aa vs N library

>>gi|324537447|gb|ADY49503.1| Peptidyl-prolyl cis-trans isomerase 3 [Ascaris  (157 aa)
 initn: 172 init1:  87 opt: 172  Z-score: 48.3  bits: 59.9 E(13331964): 3.8e-08
Smith-Waterman score: 172; 88.9% identity (94.4% similar) in 18 aa overlap (1-18:66-132)
Entrez Lookup  Re-search database  General re-search

QUERY                                               GGESLYGEK-------------------------------
                                                    :::: ::::
gi|324 TGEKGIGKSGVPLHYKGSKFHRVIPNFMCQGGDFTRGNGTGGESIYGEKFADENFQEKHTGPGVLSMANAGPNTNGSQFF
           30        40        50        60        70        80        90       100

                         10
QUERY  ------------------VTEGMDVVK
                          :::::.:::
gi|324 LCTVKTDWLDGKHVVFGRVTEGMNVVKEIESVGSQSGKTARDVVVADCGQLS
          110       120       130       140       150
```

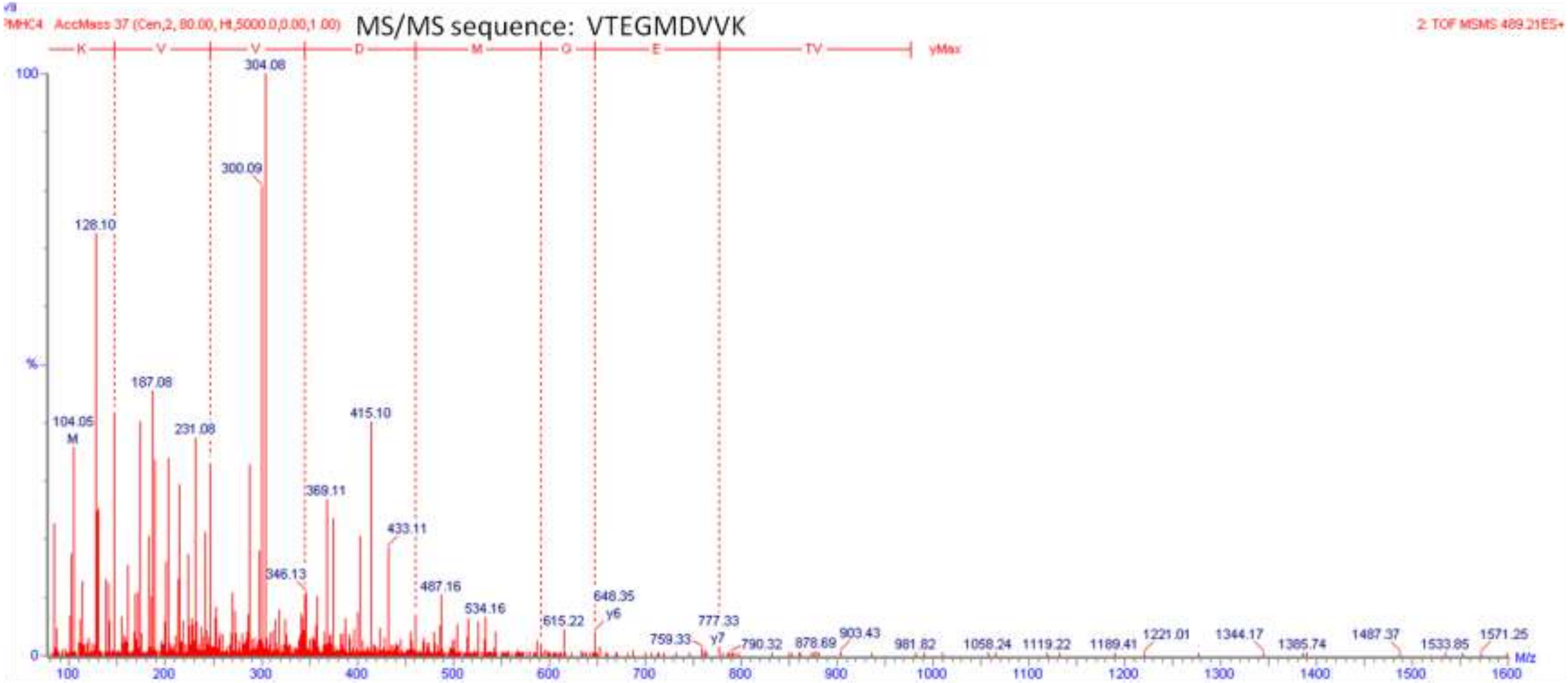

Figure S2.36.

Spot 85 MS/MS sequence 1
Ion 842.43

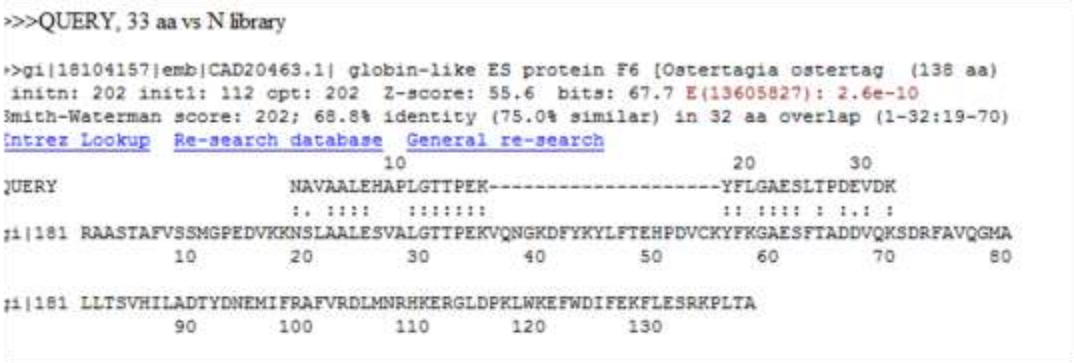

```
>>>QUERY, 33 aa vs N library

>>gi|18104157|emb|CAD20463.1| globin-like ES protein F6 [Ostertagia ostertag  (138 aa)
 initn: 202 init1: 112 opt: 202  Z-score: 55.6  bits: 67.7 E(13605827): 2.6e-10
Smith-Waterman score: 202; 68.8% identity (75.0% similar) in 32 aa overlap (1-32:19-70)
Entrez Lookup  Re-search database  General re-search
                                  10                             20        30
QUERY                   NAVAALEHAPLGTTPEK--------------------YFLGAESLTPDEVDK
                        :. ::::   :::::::                    :: :::: : :.: :
gi|181  RAASTAFVSSMGPEDVKKNSLAALESVALGTTPEKVQNGKDFYKYLFTEHPDVCKYFKGAESFTADDVQKSDRFAVQGMA
                10        20        30        40        50        60        70        80

gi|181  LLTSVHILADTYDNEMIFRAFVRDLMNRHKERGLDPKLWKEFWDIFEKFLESRKPLTA
                90       100       110       120       130
```

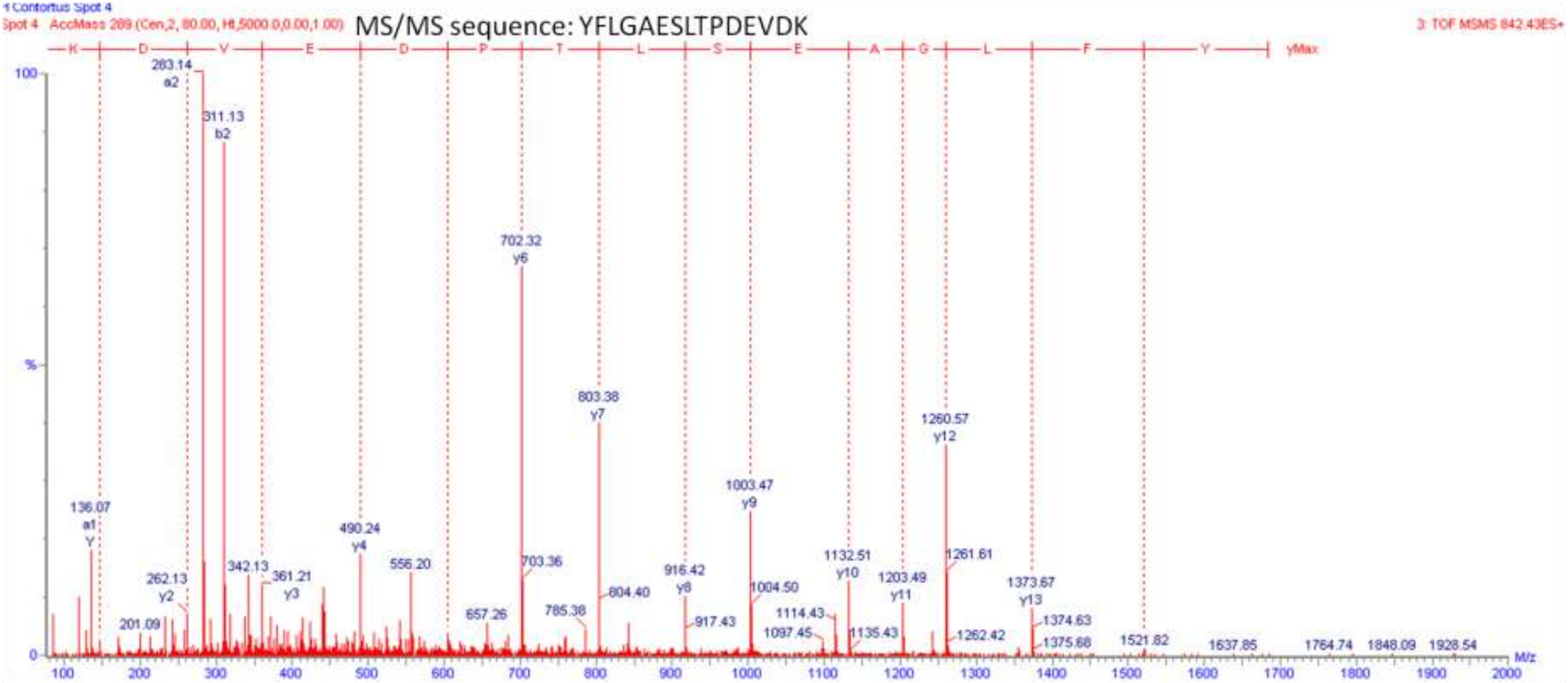

Figure S2.37.

```
>>>QUERY, 33 aa vs N library

>>gi|18104157|emb|CAD20463.1| globin-like ES protein F6 [Ostertagia ostertag  (138 aa)
 initn: 202 init1: 112 opt: 202  Z-score: 55.6  bits: 67.7 E(13605827): 2.6e-10
Smith-Waterman score: 202; 68.8% identity (75.0% similar) in 32 aa overlap (1-32:19-70)
Entrez Lookup  Re-search database  General re-search
                              10                                     20        30
QUERY                   NAVAALEHAPLGTTPEK-------------------YFLGAESLTPDEVDK
                        :. ::::   :::::::                   :: :::: : :.: :
gi|181 RAASTAFVSSMGPEDVKKNSLAALESVALGTTPEKVQNGKDFYKYLFTEHPDVCKYFKGAESFTADDVQKSDRFAVQGMA
               10        20        30        40        50        60        70        80

gi|181 LLTSVHILADTYDNEMIFRAFVRDLMNRHKERGLDPKLWKEFWDIFEKFLESRKPLTA
               90       100       110       120       130
```

Spot 85 MS/MS sequence 2
Ion 859.96

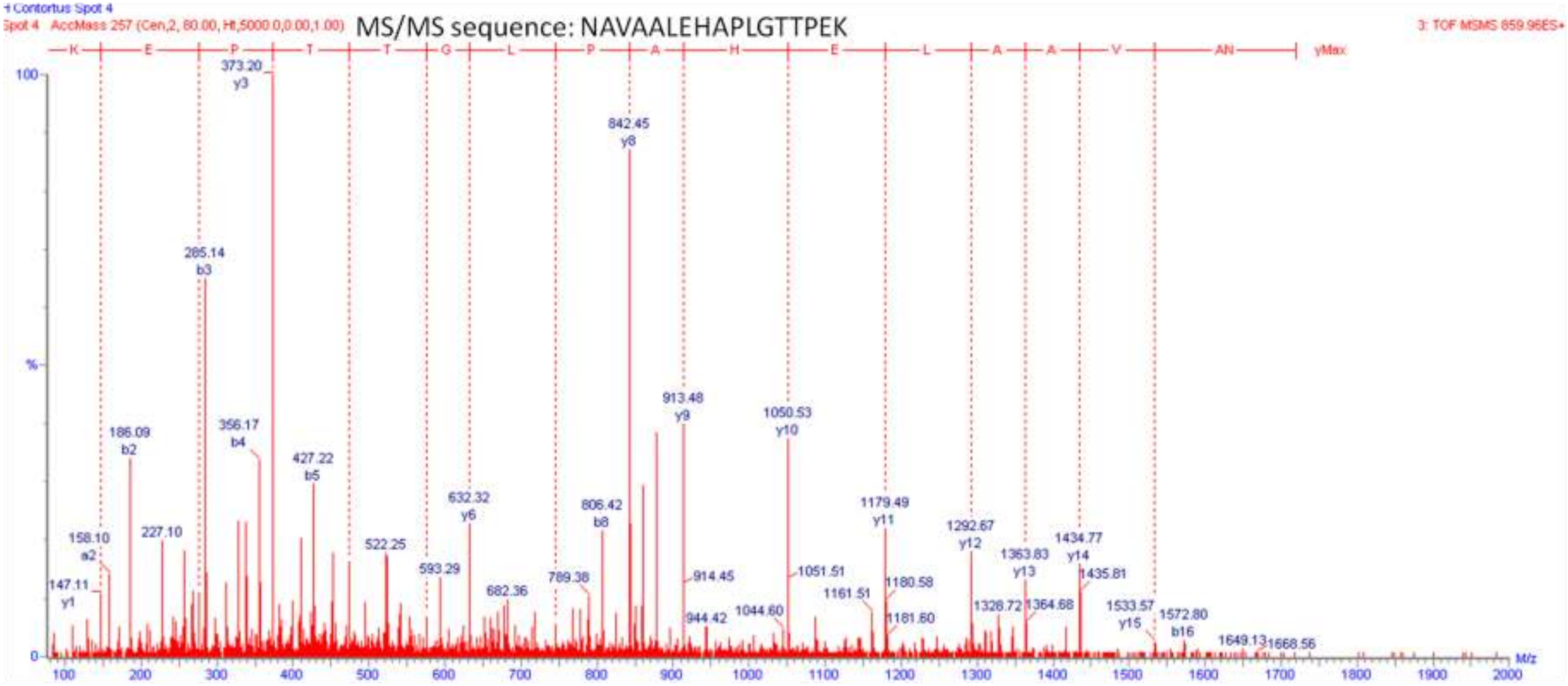

Figure S2.38.

Spot 86 MS/MS sequence 1
Ion 788.32

```
>>>QUERY, 29 aa vs N library

>>gi|290784590|dbj|BAI81973.1| major sperm protein 1 [Nippostrongylus brasil  (117 aa)
 initn: 178 init1: 106 opt: 178  Z-score: 49.1  bits: 60.8 E(13331964): 2.2e-08
Smith-Waterman score: 178; 64.3% identity (75.0% similar) in 28 aa overlap (1-28:63-97)
Entrez Lookup  Re-search database  General re-search
                                                        10                  20
QUERY                                           LMAVSGCDVFDYGR-------LTVEWHHTPEGAAK
                                                 .    ::::::::        :::: .::.::::
gi|290 TYMIKIINASGRRIGWAIKTTNMKRLGVDPACGVLDPKEATLMAVSCDVFDYGREDTNNDRITVEWCNTPDGAAKQFRRE
                 30        40        50        60        70        80        90       100

gi|290 WFQGDGMVRRKNLPI
                 110
```

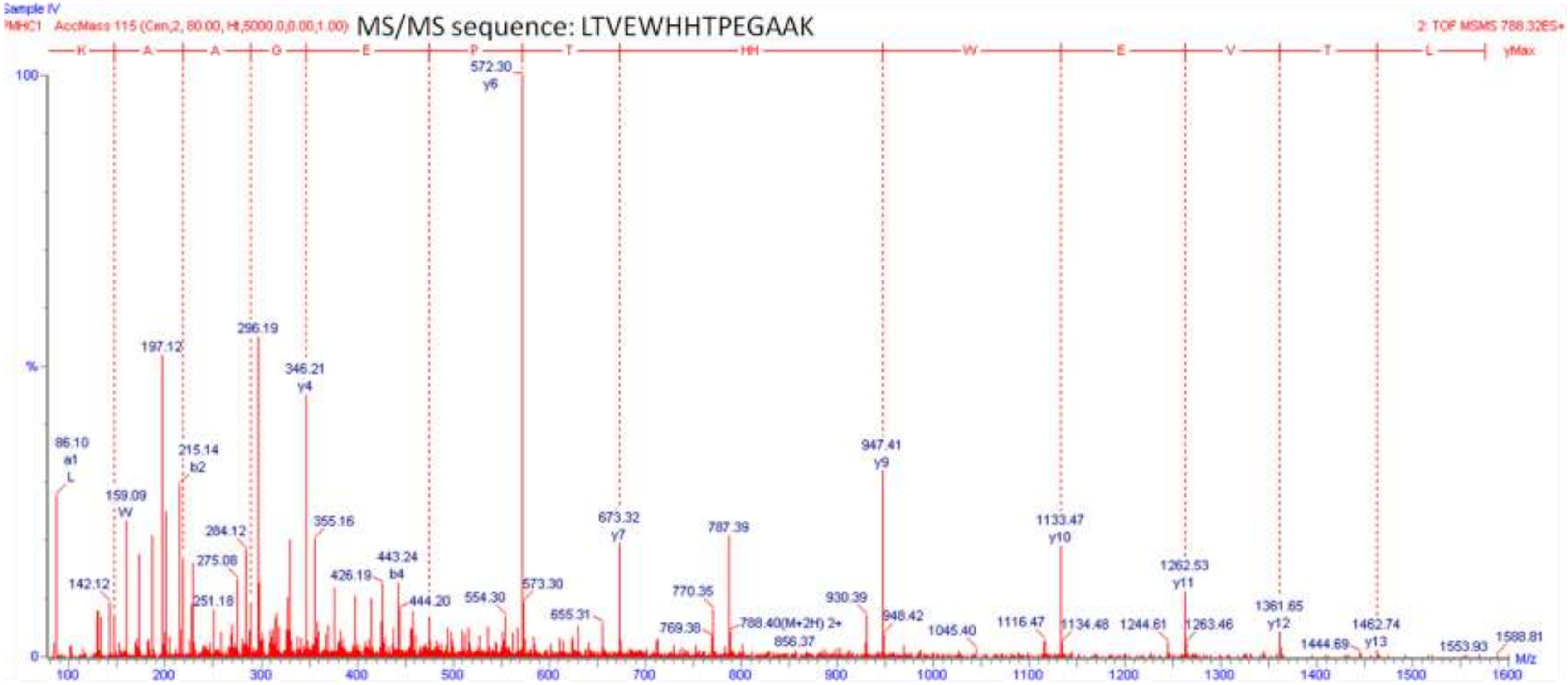

Figure S2.39.

Spot 86 MS/MS sequence 2
Ion 918.96

```
>>>QUERY, 29 aa vs N library

>>gi|290784590|dbj|BAI81973.1| major sperm protein 1 [Nippostrongylus brasil  (117 aa)
 initn: 178 init1: 106 opt: 178  Z-score: 49.1  bits: 60.8 E(13331964): 2.2e-08
Smith-Waterman score: 178; 64.3% identity (75.0% similar) in 28 aa overlap (1-28:63-97)
Entrez Lookup  Re-search database  General re-search
                                                         10                20
QUERY                                                  LMAVSGCDVFDYGR-------LTVEWHHTPEGAAK
                                                       .    ::::::::       :::: .::.::::
gi|290 TYHIKIINASGRRIGWAIKTTNMKRLGVDPACGVLDPKEATLMAVSCDVFDYGREDTNNDRITVEWCNTPDGAAKQFRRE
                30        40        50        60        70        80        90       100

gi|290 WFQGDGMVRRKNLPI
              110
```

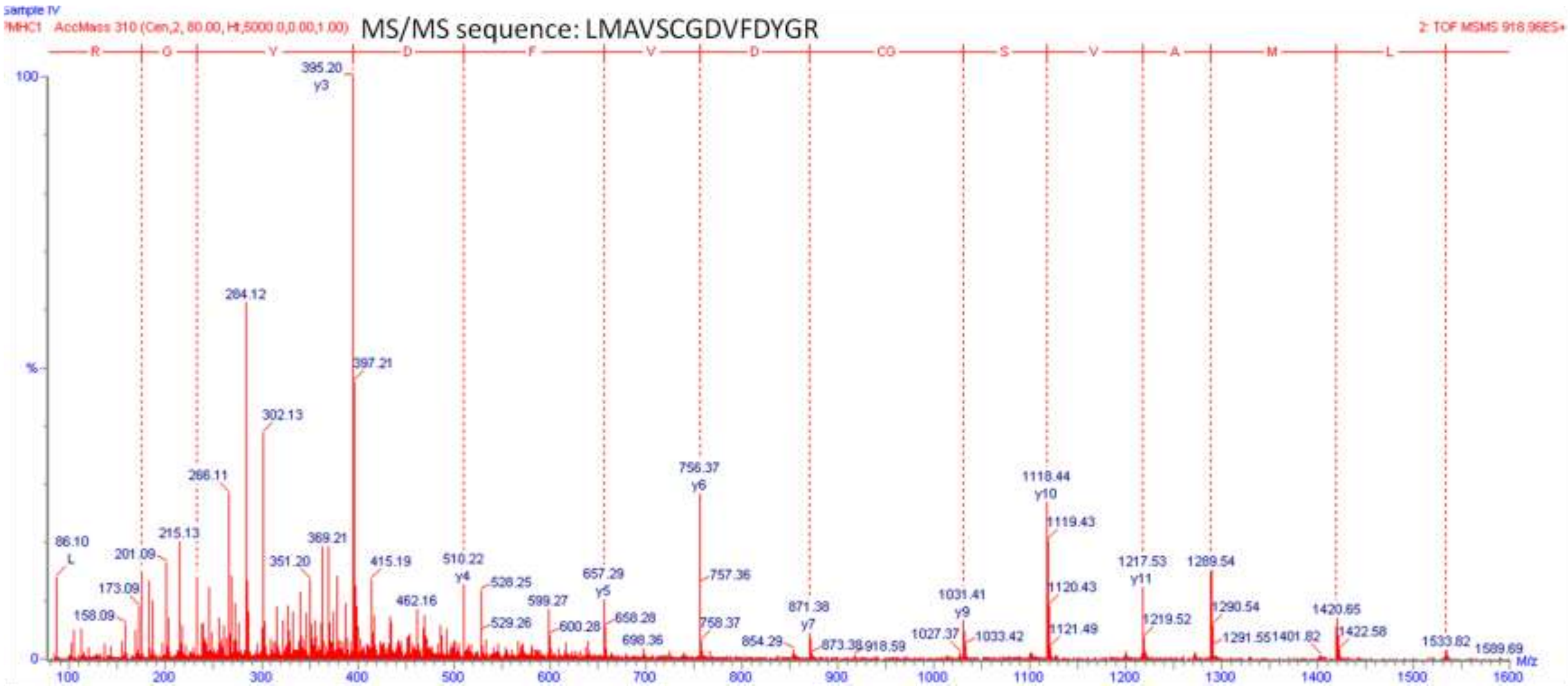

Figure S2.40.

Spot 88 MS/MS sequence 1
Ion 834.95

```
>>>QUERY, 27 aa vs N library

>>gi|18104157|emb|CAD20463.1| globin-like ES protein F6 [Ostertagia ostertag  (138 aa)
 initn: 188 init1: 109 opt: 188  Z-score: 51.2  bits: 63.0 E(13331964): 5.3e-09
Smith-Waterman score: 188; 76.9% identity (80.8% similar) in 26 aa overlap (1-26:22-73)
Entrez Lookup  Re-search database  General re-search
                                    10                                      20
QUERY                         AALESVPVGTTPDK--------------------------SFTADDVQNCLR
                              ::::::  ::::.:                          ::::::::   :
gi|181 RAASTAFVSSMGPEDVKKNSLAALESVALGTTPEKVQNGKDFYKYLFTEHPDVCKYFKGAESFTADDVQKSDRFAVQGMA
                 10        20        30        40        50        60        70        80

gi|181 LLTSVHILADTYDNEMIFRAFVRDLMNRHKERGLDPKLWKEFWDIFEKFLESRKPLTA
                 90       100       110       120       130
```

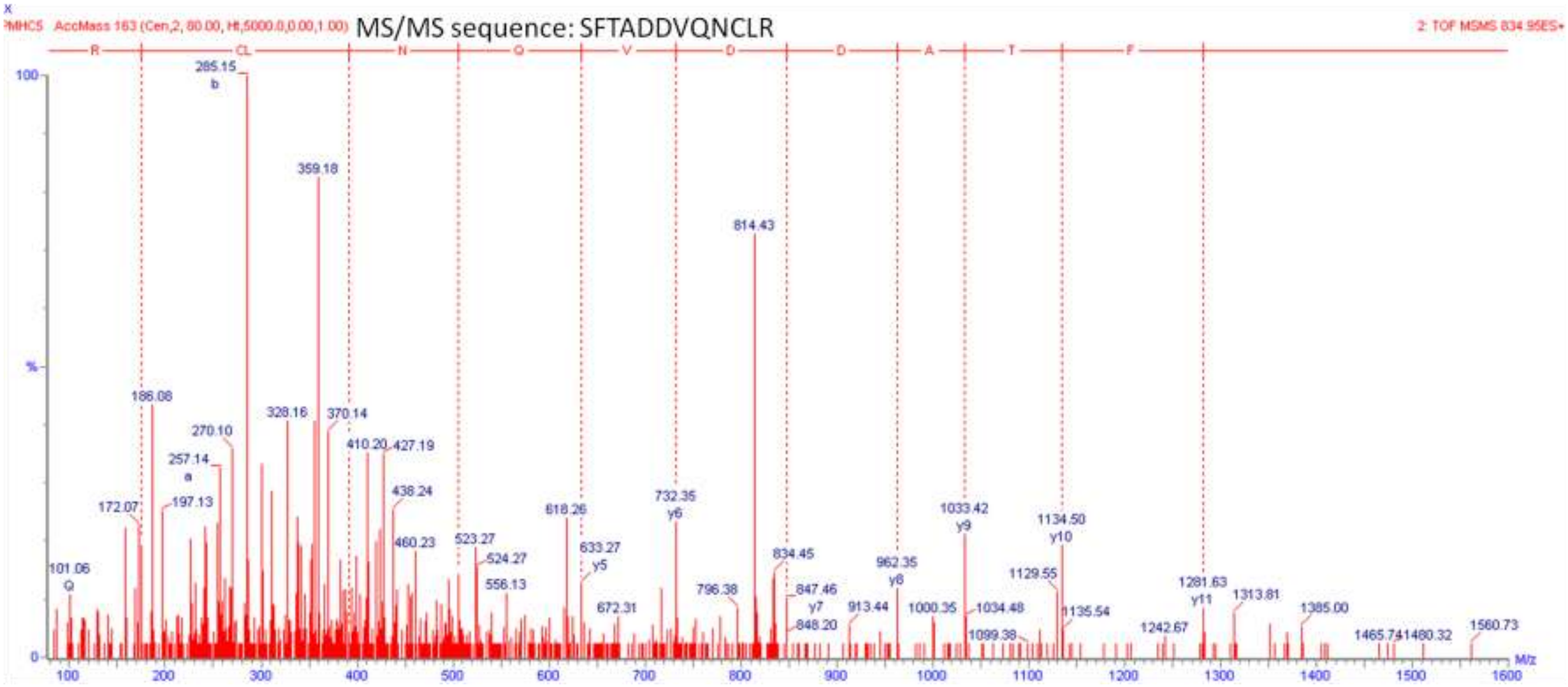

Figure S2.41.

Spot 88 MS/MS sequence 2
Ion 845.90

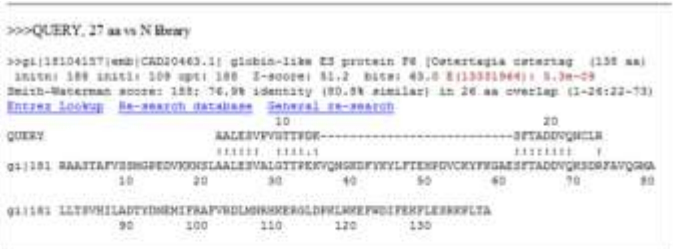

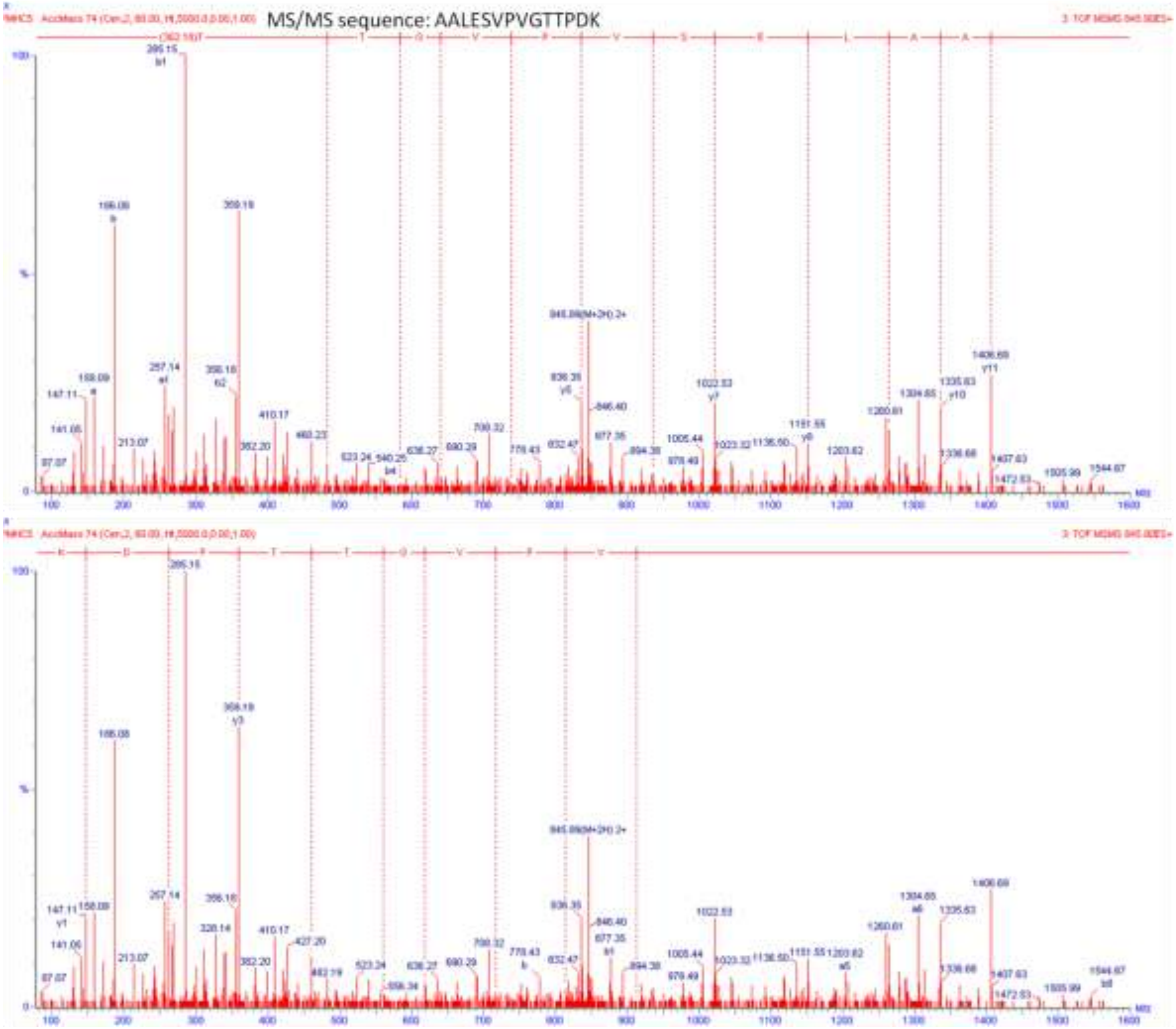

Figure S2.42.

Spot 91 MS/MS sequence 1
Ion 846.40

```
>>>QUERY, 25 aa vs N library

>>gi|308498616|ref|XP_003111494.1| hypothetical protein CRE_02928 [Caenorhab  (171 aa)
 initn: 175 init1: 112 opt: 175  Z-score: 46.6  bits: 58.6 E(13331964): 1.3e-07
Smith-Waterman score: 175; 70.8% identity (83.3% similar) in 24 aa overlap (1-24:66-131)
Entrez Lookup  Re-search database  General re-search
                                                          10
QUERY                                             LYLSGSLGLDPK----------------------------
                                                   :::::::::::
gi|308 AAKRTKVIDMAQKVTRQIISSVNAPGAIGPYSQAVRAGNTIYLSGSLGLDPKTGDLKEGIVEQTHQSLKNIGEVLKAAGA
             30        40        50        60        70        80        90       100

                             20
QUERY    --------------LNDFTTVNDLYK
                        .::.:::. :
gi|308 DYGHVVKTTVLLQNISDFATVNEVYGQYFKAPYPARAAYQVAALPKGGLVEIEAVAIAGEIEEVQN
            110       120       130       140       150       160       170
```

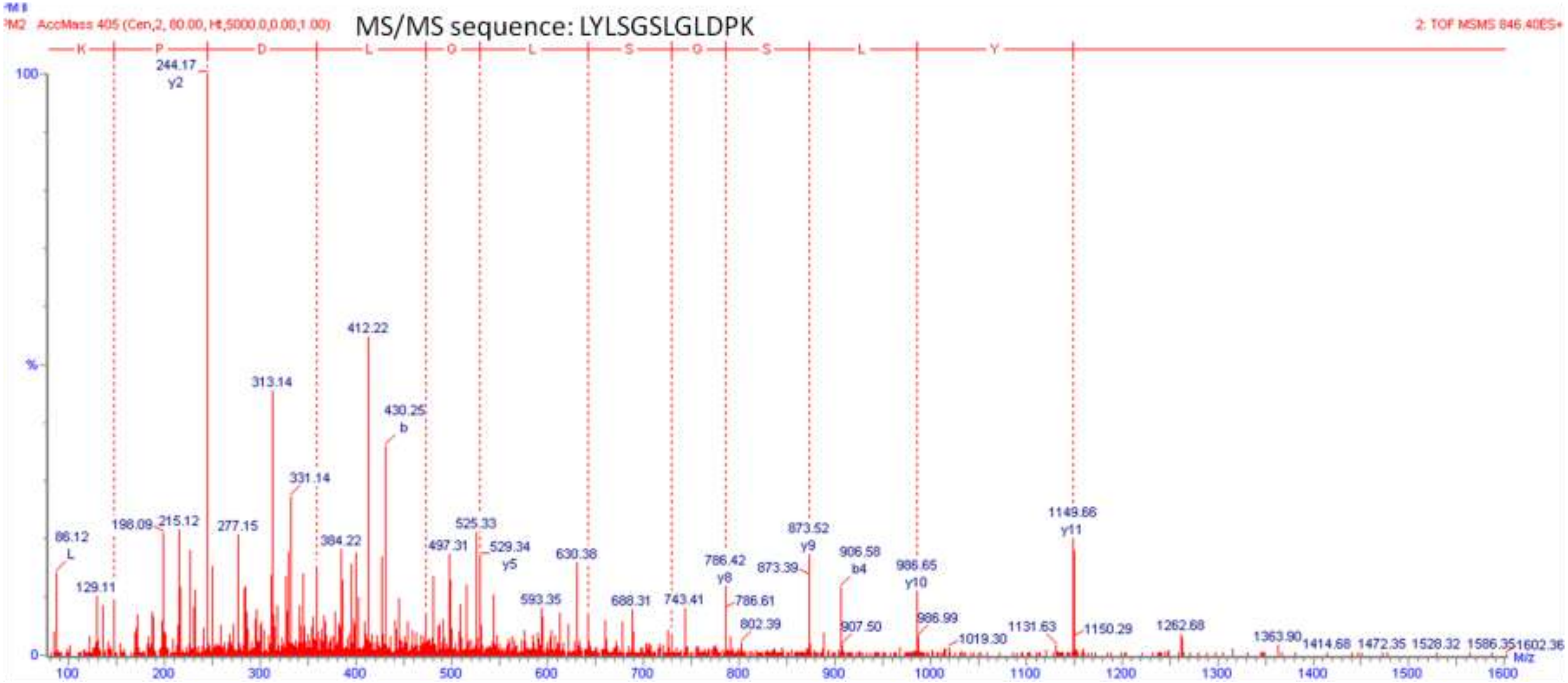

Figure S2.43.

Spot 91 MS/MS sequence 2
Ion 1078.98

```
>>>QUERY, 25 aa vs N library

>>gi|308498616|ref|XP_003111494.1| hypothetical protein CRE_02928 [Caenorhab  (171 aa)
 initn: 175 init1: 112 opt: 175  Z-score: 46.6  bits: 58.6 E(13331964): 1.3e-07
Smith-Waterman score: 175; 70.8% identity (83.3% similar) in 24 aa overlap (1-24:66-131)
Entrez Lookup  Re-search database  General re-search
                                                                    10
QUERY                                                       LYLSGSLGLDPK---------------------------
                                                             :::::::::::
gi|308 AAKRTKVIDMAQKVTRQIISSVNAPGAIGPYSQAVRAGNTIYLSGSLGLDPKTGDLKEGIVEQTHQSLKNIGEVLKAAGA
             30        40        50        60        70        80        90       100

                             20
QUERY  --------------LNDFTTVNDLYK
                      .::.:::. :
gi|308 DYGHVVKTTVLLQNISDFATVNEVYGQYFKAPYPARAAYQVAALPKGGLVEIEAVAIAGEIEEVQN
           110       120       130       140       150       160       170
```

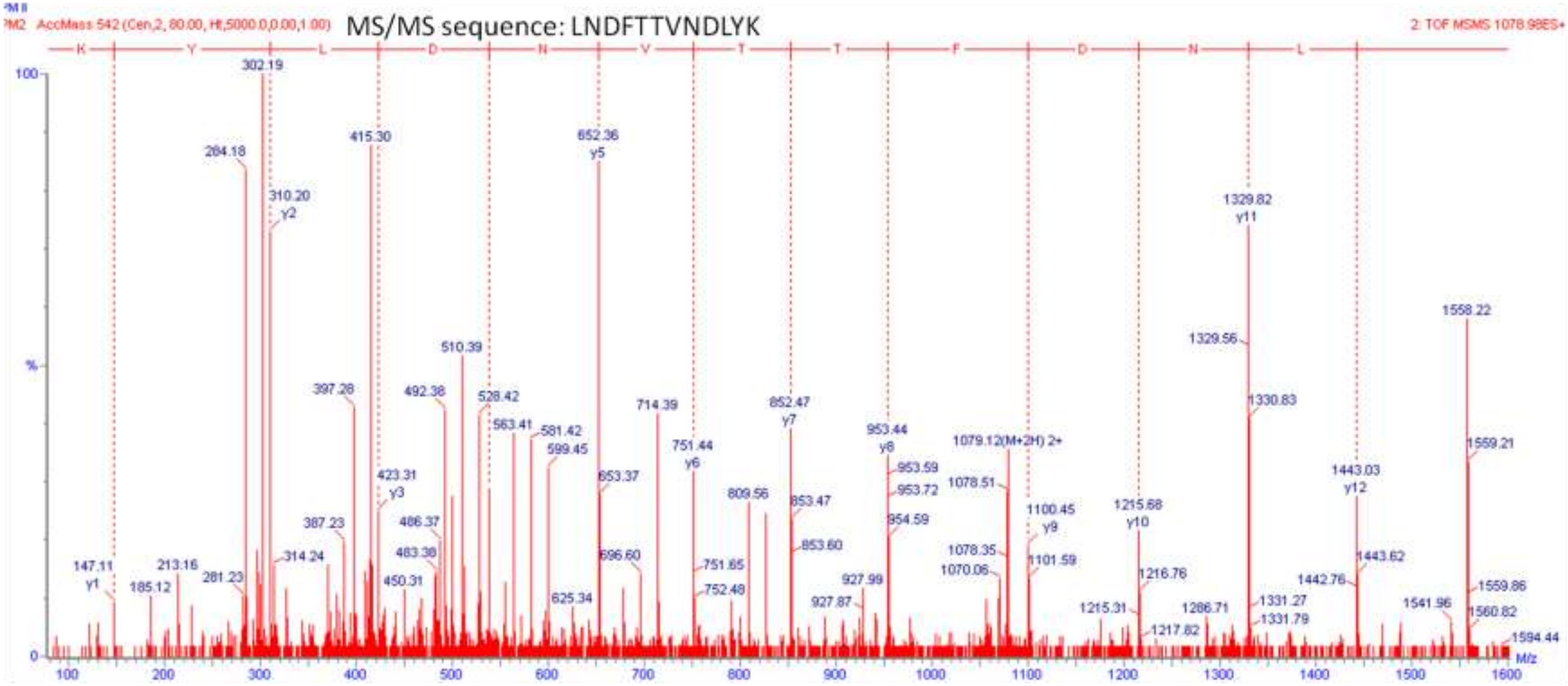

Figure S2.44.

Spot 96 MS/MS sequence 1
Ion 676.88

```
>>>QUERY, 22 aa vs N library

>>gi|226371944|gb|ACO51597.1| 10 kDa heat shock protein, mitochondrial [Rana  (102 aa)
 initn: 192 init1: 106 opt: 192  Z-score: 56.6  bits: 67.7 E(13331964): 1.2e-10
Smith-Waterman score: 192; 90.5% identity (95.2% similar) in 21 aa overlap (1-21:41-80)
Entrez Lookup  Re-search database  General re-search
                                                             10                      20
QUERY                                               VLEATVLAVGE------------------VLLPEYGGTK
                                                    ::.::: ::::                  ::::::::::
gi|226 MAGKAFKTFLPLFDRVLVERLCQETVTKGGIMLPEKAQGKVLQATVVAVGEGSRAKSGEVHPVSVTVGEKVLLPEYGGTK
               10        20        30        40        50        60        70        80

gi|226 VVLDDKDYYLFRDGDILGKYLN
               90       100
```

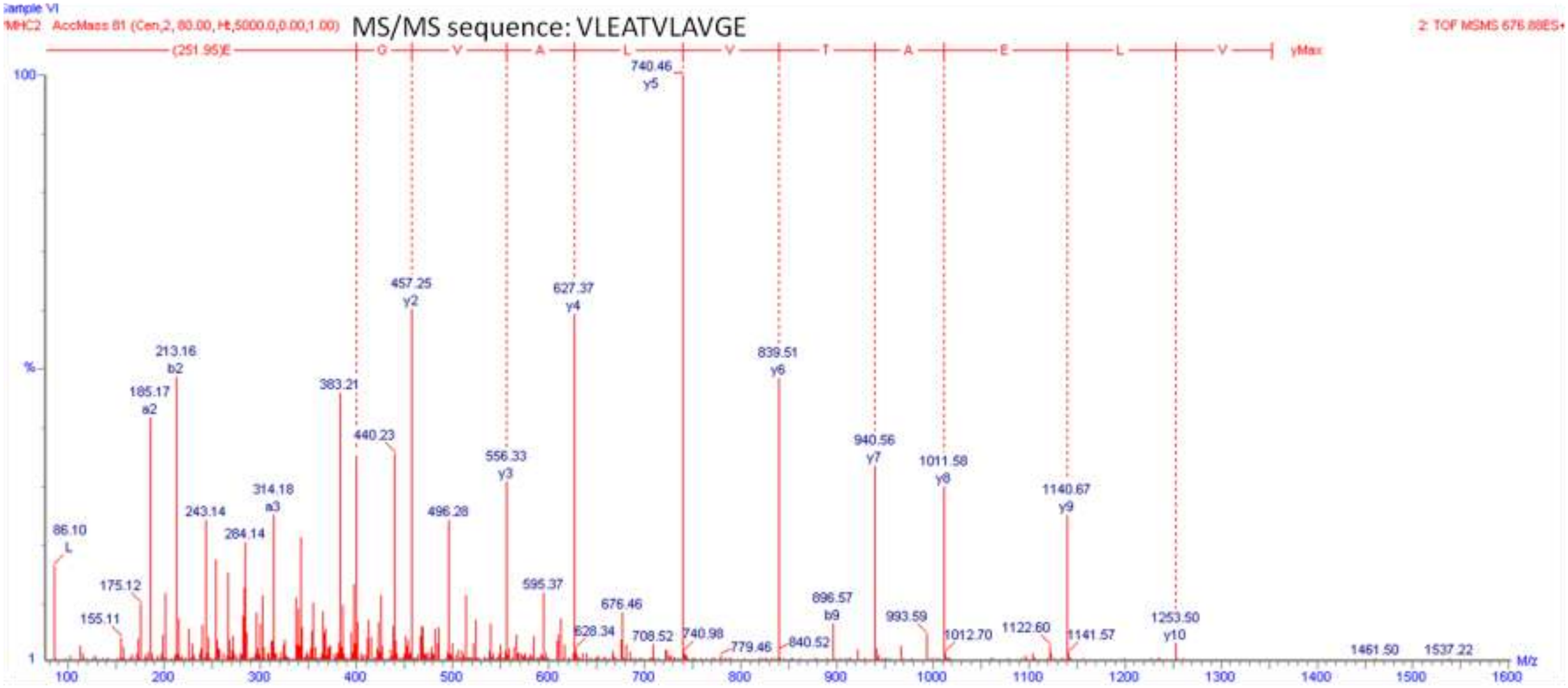

Figure S2.45.

Spot 96 MS/MS sequence 2
Ion 736.86

```
>>>QUERY, 22 aa vs N library

>>gi|226371944|gb|ACO51597.1| 10 kDa heat shock protein, mitochondrial [Rana  (102 aa)
 initn: 192 init1: 106 opt: 192  Z-score: 56.6  bits: 67.7 E(13331964): 1.2e-10
Smith-Waterman score: 192; 90.5% identity (95.2% similar) in 21 aa overlap (1-21:41-80)
Entrez Lookup  Re-search database  General re-search
                                                            10                  20
QUERY                                                       VLEATVLAVGE-------------------VLLPEYGGTK
                                                            ::.::: ::::                   ::::::::::
gi|226 MAGKAFKTFLPLFDRVLVERLCQETVTKGGIMLPEKAQGKVLQATVVAVGEGSRAKSGEVHPVSVTVGEKVLLPEYGGTK
               10        20        30        40        50        60        70        80

gi|226 VVLDDKDYYLFRDGDILGKYLN
               90       100
```

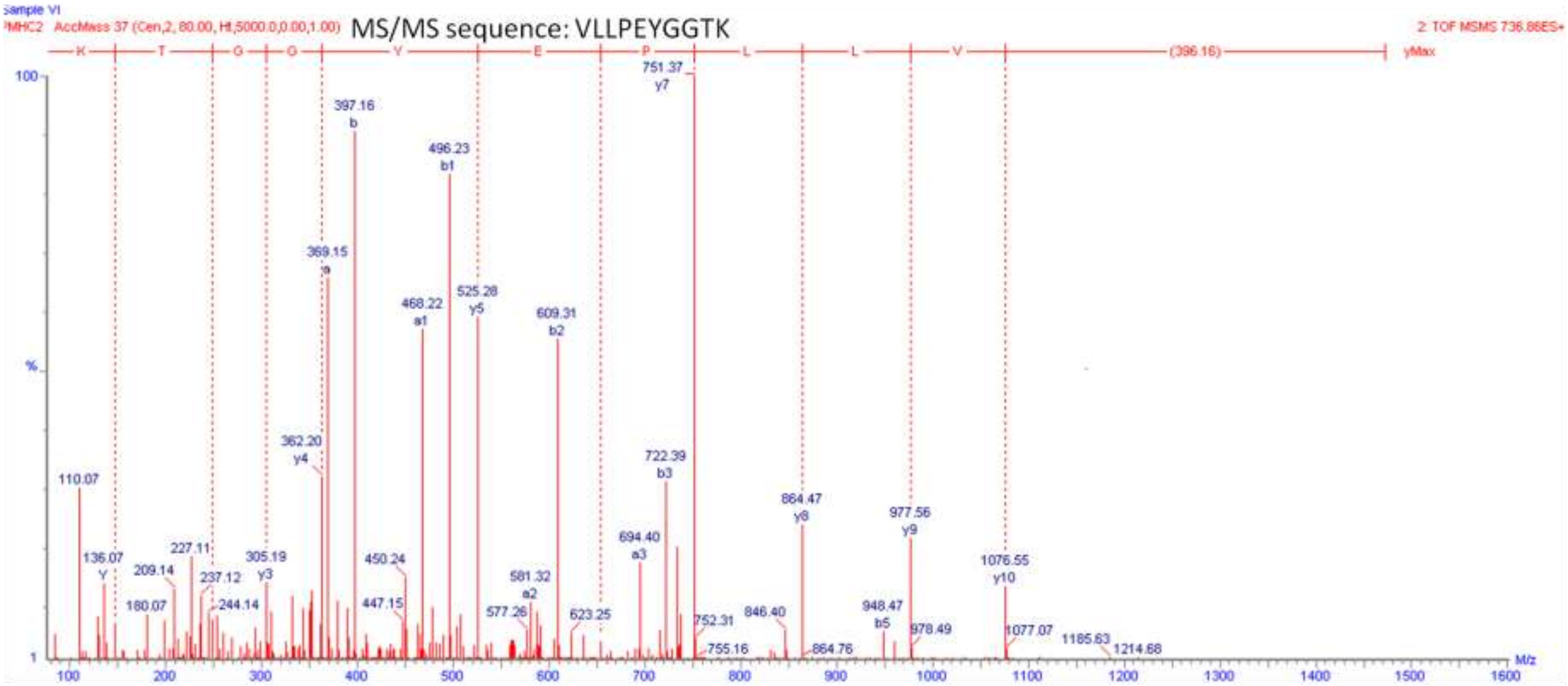

Figure S2.46.

Spot 98 MS/MS sequence 1
Ion 526.84

```
>>>QUERY, 16 aa vs N library

>>gi|259231884|gb|ACW20129.1| ubiquitin [Strongylocentrotus nudus]          (25 aa)
 initn: 149 init1:  81 opt: 149  Z-score: 48.8  bits: 57.4 E(13331964): 2.8e-08
Smith-Waterman score: 149; 93.3% identity (93.3% similar) in 15 aa overlap (1-15:5-20)
Entrez Lookup   Re-search database   General re-search
                         10
QUERY       SDYNLQK-STLHLVLR
            :::: :: ::::::::
gi|259 GRTLSDYNIQKESTLHLVLRLRGGQ
                10        20
```

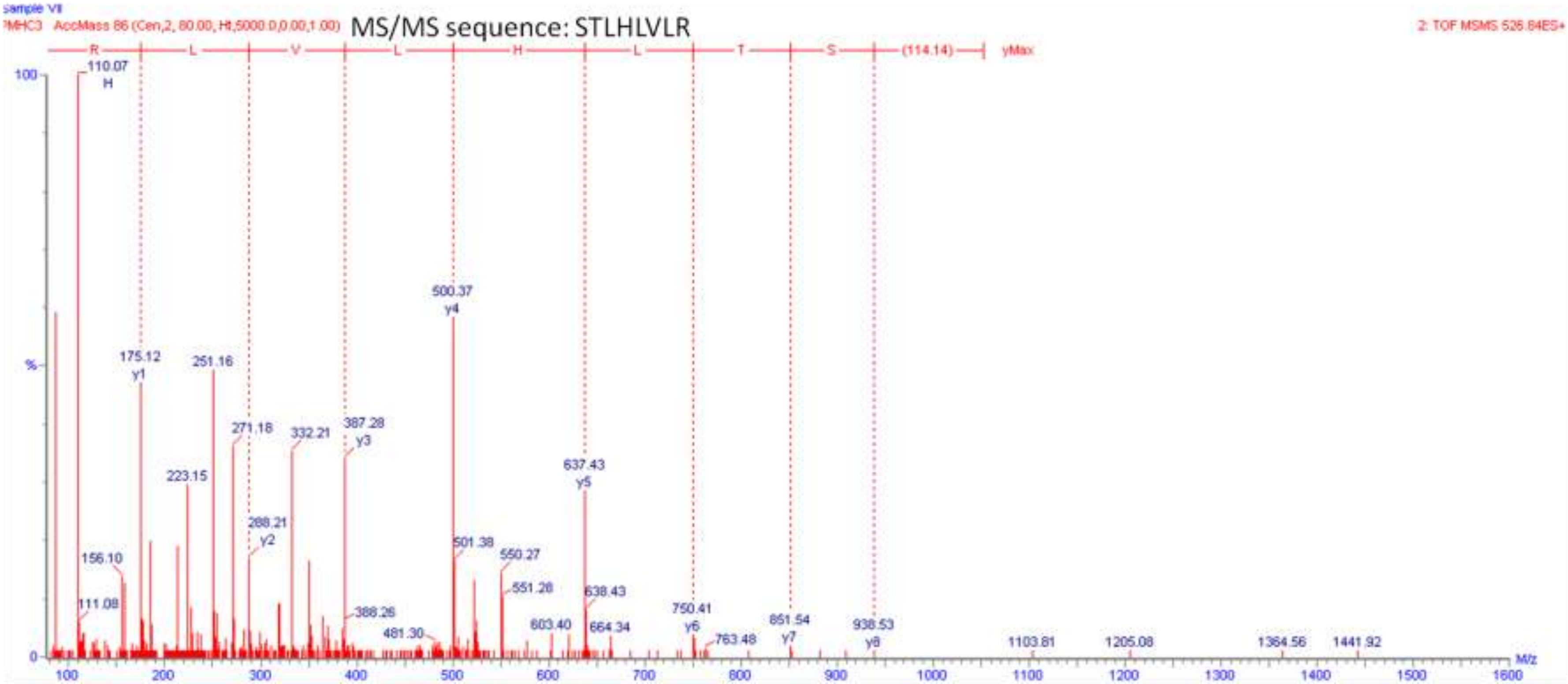

Figure S2.47.

Spot 98 MS/MS sequence 2
Ion 542.83

```
>>>QUERY, 16 aa vs N library

>>gi|259231884|gb|ACW20129.1| ubiquitin [Strongylocentrotus nudus]          (25 aa)
 initn: 149 init1:  81 opt: 149  Z-score: 48.8  bits: 57.4 E(13331964): 2.8e-08
Smith-Waterman score: 149; 93.3% identity (93.3% similar) in 15 aa overlap (1-15:5-20)
Entrez Lookup  Re-search database  General re-search
                         10
QUERY       SDYNLQK-STLHLVLR
            :::: :: ::::::::
gi|259 GRTLSDYNIQKESTLHLVLRLRGGQ
               10        20
```

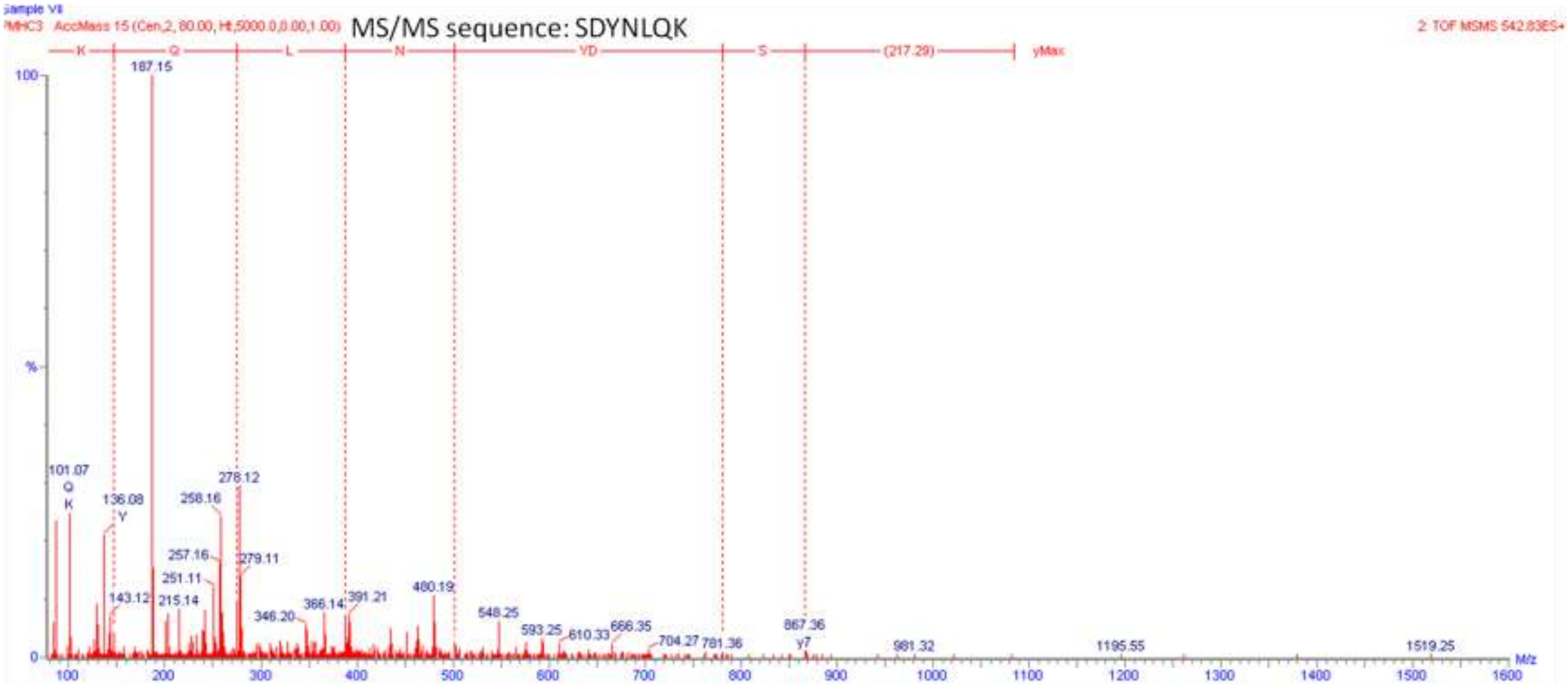

Supplement: Figure S2 — (S2.01–S2.47) De novo limited peptide sequencing from MS/MS spectra for each 2D gel spot, together with the output of FASTS searches of the sequences against the NCBI nr protein database. The results of these searches for each spot number is summarised in Tables S5, S6 and S7. (PDF) [file pone.0033590.s002.pdf]
