# Supplementary material for: Proteomic Profiling and Protein Identification by MALDI-TOF Mass Spectrometry in Unsequenced Parasitic Nematodes
Source: PLoS One. 2012 Mar 29;7(3):e33590. doi: 10.1371/journal.pone.0033590 (PMC3315570; doi:10.1371/journal.pone.0033590)
Supplement: Table S1 — Statistically significant MALDI-TOF MS protein spot identifications by PMF searching of the H. contortus putative EST protein database. Each protein spot was excised from the 250 µg protein-loaded gel and analysed by MALDI-TOF MS. A local MASCOT PMF search of the H. contortus putative EST protein database was performed and the highest scoring EST sequence match, along with its MOWSE-based score (significance threshold score >51, p-value<0.05), sequence coverage and the number of matched peptides is reported. For each search, the highest scoring hit EST sequence accession number and its theoretical Mw/pI are also detailed. (DOC) [file pone.0033590.s003.doc]

**Table S1.** Statistically significant MALDI-TOF MS protein spot identifications by PMF searching of the *H. contortus* putative EST protein database.

| Protein spot | Observed Mw/pI (kDa) | Mascot MOWSE Score | Sequence Coverage % | Matched Peptides (Total) | EST Accession Number | EST sequence Theoretical Mw/pI (kDa) |
| --- | --- | --- | --- | --- | --- | --- |
| 4 | 59.4/3.84 | 65 | 30 | 20 | 00006 1 | 59.6/6.67 |
| 8 | 59.5/5.00 | 58 | 24 | 14 | 00006 1 | 59.6/6.67 |
| 9 | 59.1/5.19 | 68 | 29 | 18 | 00006 1 | 59.6/6.67 |
| 10 | 60.6/6.17 | 64 | 29 | 14 | 00006 1 | 59.6/6.67 |
| 11 | 60.3/6.38 | 78 | 29 | 15 | 00006 1 | 59.6/6.67 |
| 12 | 59.8/6.60 | 108 | 38 | 21 | 00006 1 | 59.6/6.67 |
| 13 | 59.5/6.76 | 71 | 60 | 36 | 00006 1 | 59.6/6.67 |
| 14 | 59.9/6.91 | 56 | 33 | 17 | 00006 1 | 59.6/6.67 |
| 15 | 61.0/7.16 | 74 | 21 | 13 | 00006 1 | 59.6/6.67 |
| 17 | 48.4/3.88 | 51 | 54 | 44 | 00006 1 | 59.6/6.67 |
| 18 | 41.9/3.89 | 54 | 25 | 9 | 06327 1 | 18.7/5.96 |
| 21 | 42.2/4.47 | 79 | 42 | 8 | 00195 1 | 24.5/5.87 |
| 22 | 41.9/4.54 | 77 | 49 | 9 | 00195 1 | 24.5/5.87 |
| 25 | 40.1/5.95 | 53 | 50 | 13 | 01204 1 | 25.9/6.10 |
| 27 | 41.0/6.55 | 62 | 28 | 13 | 00006 1 | 59.6/6.67 |
| 28 | 41.2/6.99 | 59 | 45 | 26 | 00006 1 | 59.6/6.67 |
| 29 | 48.9/7.07 | 59 | 40 | 25 | 00006 1 | 59.6/6.67 |
| 40 | 39.2/9.10 | 51 | 62 | 10 | 11007 1 | 23.8/8.12 |
| 41 | 39.9/9.32 | 64 | 62 | 9 | 11007 1 | 23.8/8.12 |
| 43 | 32.4/6.30 | 68 | 55 | 35 | 00006 1 | 59.6/6.67 |
| 44 | 28.4/8.16 | 83 | 36 | 9 | 06393 1 | 27.4/8.18 |
| 45 | 28.6/8.45 | 96 | 48 | 13 | 06393 1 | 27.4/8.18 |
| 46 | 26.1/6.76 | 78 | 60 | 8 | 02208 1 | 19.5/5.63 |
| 47 | 24.6/7.45 | 58 | 25 | 5 | 00515 1 | 22.9/6.69 |
| 53 | 22.9/5.97 | 52 | 23 | 6 | 01409 1 | 21.9/8.76 |
| 54 | 23.0/6.27 | 57 | 42 | 9 | 07180 1 | 19.6/7.88 |
| 59 | 19.5/6.31 | 53 | 43 | 9 | 06327 1 | 18.7/5.96 |
| 61 | 19.5/6.92 | 59 | 42 | 9 | 02260 2 | 18.4/6.30 |
| 70 | 15.2/4.73 | 66 | 72 | 9 | 02240 2 | 15.1/6.15 |
| 72 | 15.2/5.24 | 79 | 49 | 8 | 02740 1 | 15.1/6.15 |
| 73 | 14.6/5.23 | 85 | 77 | 11 | 02740 1 | 15.1/6.15 |
| 77 | 13.9/5.46 | 71 | 58 | 6 | 07574 1 | 14.2/6.13 |
| 81 | 16.5/6.21 | 61 | 83 | 19 | 00942 2 | 17.4/6.31 |
| 85 | 17.0/7.82 | 85 | 68 | 13 | 00208 1 | 19.2/6.59 |
| 86 | 16.7/8.08 | 87 | 56 | 9 | 02230 2 | 14.1/7.68 |
| 88 | 16.1/7.94 | 54 | 29 | 4 | 00202 5 | 19.6/6.20 |
| 89 | 15.8/7.72 | 106 | 45 | 11 | 01375 1 | 19.0/6.45 |
| 91 | 14.1/8.23 | 68 | 75 | 6 | 03264 1 | 9.2/9.99 |
| 92 | 14.5/8.57 | 53 | 52 | 12 | 00372 1 | 24.7/7.82 |

Each protein spot was excised from the 250 μg protein-loaded gel and analysed by MALDI-TOF MS. A local MASCOT PMF search of the *H. contortus* putative EST protein database was performed and the highest scoring EST sequence match, along with its MOWSE-based score (significance threshold score > 51, p-value < 0.05), sequence coverage and the number of matched peptides is reported. For each search, the highest scoring hit EST sequence accession number and its theoretical Mw/p*I* are also detailed.
