# Supplementary material for: Proteomic Profiling and Protein Identification by MALDI-TOF Mass Spectrometry in Unsequenced Parasitic Nematodes
Source: PLoS One. 2012 Mar 29;7(3):e33590. doi: 10.1371/journal.pone.0033590 (PMC3315570; doi:10.1371/journal.pone.0033590)
Supplement: Table S2 — Annotation of the statistically significant EST sequence hits using BLASTp searches against the entire NCBI nr protein database. Each EST sequence hit was submitted to a BLASTp search against the entire NCBI nr protein database. For each search, the highest scoring hit score (significance threshold >44, p-value<0.01), its accession number and protein name are reported. For information, the species corresponding to the highest scoring hit, its molecular function according to the NCBI nr protein database and Wormbase when available1 and the theoretical Mw/pI of the full length sequence are also described. (DOC) [file pone.0033590.s004.doc]

**Table S2.** Annotation of the statistically significant EST sequence hits using BLASTp searches against the entire NCBI nr protein database.

| Protein spot | EST Accession Number | BLAST score | Accession Number | Protein Identified | Species | Molecular Function | Theoretical Mw/pI of full length sequence(kDa) |
| --- | --- | --- | --- | --- | --- | --- | --- |
| 4 | 00006 1 | 1077 | ACT34056 | Putative Glutamate deHydrogenase | *H. contortus* | oxidoreductase | 59.1/6.67 |
| 8 | 00006 1 | 1077 | ACT34056 | Putative Glutamate deHydrogenase | *H. contortus* | oxidoreductase | 59.1/6.67 |
| 9 | 00006 1 | 1077 | ACT34056 | Putative Glutamate deHydrogenase | *H. contortus* | oxidoreductase | 59.1/6.67 |
| 10 | 00006 1 | 1077 | ACT34056 | Putative Glutamate deHydrogenase | *H. contortus* | oxidoreductase | 59.1/6.67 |
| 11 | 00006 1 | 1077 | ACT34056 | Putative Glutamate deHydrogenase | *H. contortus* | oxidoreductase | 59.1/6.67 |
| 12 | 00006 1 | 1077 | ACT34056 | Putative Glutamate deHydrogenase | *H. contortus* | oxidoreductase | 59.1/6.67 |
| 13 | 00006 1 | 1077 | ACT34056 | Putative Glutamate deHydrogenase | *H. contortus* | oxidoreductase | 59.1/6.67 |
| 14 | 00006 1 | 1077 | ACT34056 | Putative Glutamate deHydrogenase | *H. contortus* | oxidoreductase | 59.1/6.67 |
| 15 | 00006 1 | 1077 | ACT34056 | Putative Glutamate deHydrogenase | *H. contortus* | oxidoreductase | 59.1/6.67 |
| 17 | 00006 1 | 1077 | ACT34056 | Putative Glutamate deHydrogenase | *H. contortus* | oxidoreductase | 59.1/6.67 |
| 18 | 06327 1 | 298 | XP_001897798 | Ubiquitin conjugating enzyme E2 H | *B. malayi* | small conjugating protein ligase | 22.4/5.05 |
| 21 | 00195 1 | 398 | XP_001899521 | Disorganized muscle protein 1 | *B. malayi* | unknown | 35.5/4.99 |
| 22 | 00195 1 | 398 | XP_001899521 | Disorganized muscle protein 1 | *B. malayi* | unknown | 35.5/4.99 |
| 25 | 01204 1 | 351 | NP_001023074 | Inorganic Pyrophosphatase | *C. elegans* | inorganic diphosphatase | 37.3/5.13 |
| 27 | 00006 1 | 1077 | ACT34056 | Putative Glutamate deHydrogenase | *H. contortus* | oxidoreductase | 59.1/6.67 |
| 28 | 00006 1 | 1077 | ACT34056 | Putative Glutamate deHydrogenase | *H. contortus* | oxidoreductase | 59.1/6.67 |
| 29 | 00006 1 | 1077 | ACT34056 | Putative Glutamate deHydrogenase | *H. contortus* | oxidoreductase | 59.1/6.67 |
| 40 | 11007 1 | 336 | XP_001666501 | CBG15213 | *C. briggsae AF16* | malate dehydrogenase/oxidoreductase | 35.0/9.33 |
| 41 | 11007 1 | 336 | XP_001666501 | CBG15213 | *C. briggsae AF16* | malate dehydrogenase/oxidoreductase | 35.0/9.33 |
| 43 | 00006 1 | 1077 | ACT34056 | Putative Glutamate deHydrogenase | *H. contortus* | oxidoreductase | 59.1/6.67 |
| 44 | 06393 1 | 400 | XP_001680248 | CBG21017 | *C. briggsae AF16* | triose-phosphate isomerase | 26.5/5.99 |
| 45 | 06393 1 | 400 | XP_001680248 | CBG21017 | *C. briggsae AF16* | triose-phosphate isomerase | 26.5/5.99 |
| 46 | 02208 1 | 252 | 1TW9A | Glutathione Transferase-2, Apo Form | *H. polygyrus* | glutathione transferase | 23.3/6.84 |
| 47 | 00515 1 | 171 | XP_001671373 | CBG17729 | *C. briggsae AF16* | unknown | 26.2/8.45 |
| 53 | 01409 1 | 249 | NP_499900 | K02D7.1 | *C. elegans* | purine-nucleoside phosphorylase | 32.4/5.62 |
| 54 | 07180 1 | 226 | XP_001669439 | CBG19736 | *C. briggsae AF16* | peptidyl-prolyl cis-trans isomerase | 22.1/8.85 |
| 59 | 06327 1 | 298 | XP_001897798 | Ubiquitin conjugating enzyme E2 H | *B. malayi* | small conjugating protein ligase | 22.4/5.05 |
| 61 | 02260 2 | 267 | CAG25499 | heat shck protein 20 | *O. ostertagi* | chaperonin | 18.2/6.30 |
| 70 | 02240 2 | 167 | NP_001033512 | Lipid Binding Protein | *C. elegans* | lipid binding/transporter | 16.9/7.93 |
| 72 | 02740 1 | 167 | NP_001033512 | Lipid Binding Protein | *C. elegans* | lipid binding/transporter | 16.9/7.93 |
| 73 | 02740 1 | 167 | NP_001033512 | Lipid Binding Protein | *C. elegans* | lipid binding/transporter | 16.9/7.93 |
| 77 | 07574 1 | 69,3 | NP_495503 | E04F6.9 | *C. elegans* | unknown | 13.7/7.77 |
| 81 | 00942 2 | 165 | AAN05752 | heat shock protein 20 | *H. contortus* | chaperonin | 18.3/6.23 |
| 85 | 00208 1 | 197 | P27613 | Globin-like host protective antigen | *T. colubriformis* | heme/iron ion/oxygen binding | 19.9/7.03 |
| 86 | 02230 2 | 260 | ABJ97284 | major sperm protein | *D. viviparus* | structurale molecule | 14.2/7.71 |
| 88 | 00202 5 | 216 | P27613 | Globin-like host protective antigen | *T. colubriformis* | heme/iron ion/oxygen binding | 19.9/7.03 |
| 89 | 01375 1 | 205 | NP_508557 | Lipid Binding Protein | *C. elegans* | lipid binding/transporter | 18.2/8.50 |
| 91 | 03264 1 | 137 | NP_741178 | C23G10.2 | *C. elegans* | unknown | 15.1/6.58 |
| 92 | 00372 1 | 210 | CAP20913 | CBG24261 | *C. briggsae* | unknown | 18.1/5.73 |

^1^ Wormbase was used to infer molecular function when a link from the NCBI nr protein database webpage of the protein in question was available. If not available, the closest *C. elegans* protein BLASTp hit (with the highest similarity score) Wormbase link was used to infer function in the same manner.

Each EST sequence hit was submitted to a BLASTp search against the entire NCBI nr protein database. For each search, the highest scoring hit score (significance threshold > 44, p-value < 0.01), its accession number and protein name are reported. For information, the species corresponding to the highest scoring hit, its molecular function according to the NCBI nr protein database and Wormbase when available^1^ and the theoretical Mw/p*I* of the full length sequence are also described.
