# Supplementary material for: Proteomic Profiling and Protein Identification by MALDI-TOF Mass Spectrometry in Unsequenced Parasitic Nematodes
Source: PLoS One. 2012 Mar 29;7(3):e33590. doi: 10.1371/journal.pone.0033590 (PMC3315570; doi:10.1371/journal.pone.0033590)
Supplement: Table S3 — Non-statistically significant MALDI-TOF MS protein spot identifications using the H. contortus putative EST protein database. Each protein spot was excised from the 250 µg protein-loaded gel and analysed by MALDI-TOF MS. A local MASCOT PMF search of the H. contortus putative EST protein database was performed and the highest scoring EST sequence match along with its MOWSE-based score (significance threshold score >51, p-value<0.05), sequence coverage and the number of matched peptides is reported. For each search, the highest scoring hit EST sequence accession number and its theoretical Mw/pI are also detailed. The observed Mw of the spots, calculated on the gel image, were correlated with the theoretical Mw of the intact protein in the best BLASTp match, given in Table S4. (DOC) [file pone.0033590.s005.doc]

**Table S3.** Non-statistically significant MALDI-TOF MS protein spot

identifications using the *H. contortus* putative EST protein database.

| Protein spot | Observed Mw/pI (kDa) | Mascot MOWSE Score | Sequence Coverage % | Matched Peptides (Total) | EST Accession Number | EST sequence Theoretical Mw/pI (kDa) |
| --- | --- | --- | --- | --- | --- | --- |
| 2 | 62.8/4.68 | 34 | 19 | 7 | 03377 1 | 45.3/5.93 |
| 6 | 59.4/4.40 | 47 | 49 | 11 | 00273 1 | 26.4/6.04 |
| 7 | 57.0/4.20 | 41 | 18 | 8 | 00592 1 | 46.9/4.77 |
| 19 | 49.5/4.64 | 50 | 33 | 9 | 00199 6 | 37.6/5.20 |
| 20 | 49.9/5.22 | 31 | 25 | 5 | 00199 8 | 28.9/5.30 |
| 24 | 40.6/5.66 | 40 | 53 | 14 | 01204 1 | 25.9/6.10 |
| 30 | 52.4/7.41 | 42 | 72 | 10 | 00280 1 | 20.5/9.14 |
| 32 | 52.8/8.03 | 43 | 44 | 8 | 00537 1 | 18.4/5.81 |
| 34 | 48.2/7.85 | 50 | 56 | 12 | 00183 1 | 29.6/7.11 |
| 35 | 47.9/8.08 | 48 | 68 | 15 | 00183 1 | 29.6/7.11 |
| 37 | 45.2/7.92 | 37 | 33 | 9 | 01607 2 | 39.1/7.83 |
| 42 | 38.4/9.36 | 34 | 18 | 4 | 11007 1 | 23.8/8.12 |
| 57 | 20.1/5.74 | 35 | 34 | 7 | 06327 1 | 18.7/5.96 |
| 60 | 19.3/6.62 | 43 | 32 | 6 | 00814 1 | 18.3/6.23 |
| 62 | 20.9/7.28 | 48 | 40 | 6 | 03240 3 | 23.0/8.69 |
| 63 | 21.6/7.73 | 39 | 78 | 15 | 03240 1 | 23.4/8.99 |
| 78 | 14.1/5.56 | 49 | 30 | 4 | 00413 1 | 16.1/5.17 |
| 80 | 16.9/5.67 | 45 | 65 | 8 | 11248 1 | 14.8/5.67 |
| 82 | 17.0/6.91 | 41 | 43 | 5 | 00047 3 | 16.5/6.39 |
| 90 | 14.2/7.97 | 46 | 73 | 17 | 00907 1 | 15.0/8.49 |
| 93 | 13.9/8.56 | 36 | 75 | 7 | 03264 2 | 11.1/9.52 |
| 94 | 12.5/8.00 | 46 | 50 | 9 | 04833 1 | 17.7/9.55 |
| 96 | 11.7/7.46 | 41 | 47 | 4 | 00229 1 | 10.3/7.03 |

Each protein spot was excised from the 250 μg protein-loaded gel and analysed by MALDI-TOF MS. A local MASCOT PMF search of the *H. contortus* putative EST protein database was performed and the highest scoring EST sequence match along with its MOWSE-based score (significance threshold score > 51, p-value < 0.05), sequence coverage and the number of matched peptides is reported. For each search, the highest scoring hit EST sequence accession number and its theoretical Mw/pI are also detailed. The observed Mw of the spots, calculated on the gel image, were correlated with the theoretical Mw of the intact protein in the best BLASTp match, given in Supplementary Table S4.
