# Supplementary material for: Proteomic Profiling and Protein Identification by MALDI-TOF Mass Spectrometry in Unsequenced Parasitic Nematodes
Source: PLoS One. 2012 Mar 29;7(3):e33590. doi: 10.1371/journal.pone.0033590 (PMC3315570; doi:10.1371/journal.pone.0033590)
Supplement: Table S4 — Annotation of the non-statistically significant hit EST sequences using BLASTp against the entire NCBI nr protein database. Each EST sequence hit was submitted to a BLASTp search against the entire NCBI nr protein database. For each search, the highest scoring hit score (significance threshold >44, p-value<0.01), its accession number and protein name are reported. For information, the species corresponding to the highest scoring hit, its molecular function according to the NCBI nr database and Wormbase when available1 and the theoretical Mw/pI of its full length sequence are also described. (DOC) [file pone.0033590.s006.doc]

**Table S4.** Annotation of the non-statistically significant hit EST sequences using BLASTp against the entire NCBI nr protein database.

| Protein spot | EST Accession Number | BLAST score | Accession Number | Protein Identified | Species | Molecular Function | Theoretical Mw/pI of full length sequence (kDa) |
| --- | --- | --- | --- | --- | --- | --- | --- |
| 2 | 03377 1 | 697 | XP_001664603 | CBG11701 | *C. briggsae AF16* | chaperonin | 59.9/5.37 |
| 6 | 00273 1 | 420 | ABC86956 | Protein Disulfide Isomerase | *T. circumcincta* | Isomerase | 54.9/4.93 |
| 7 | 00592 1 | 540 | CAL30086 | Calreticulin precursor | *H. polygyrus* | calcium binding chaperonin | 47.0/4.74 |
| 19 | 00199 6 | 683 | ABX82966 | Actin variant 1 | *D. vivparus* | ATP binding Protein binding | 41.8/5.16 |
| 20 | 00199 8 | 515 | ABX82966 | Actin variant 1 | *D. vivparus* | ATP binding Protein binding | 41.8/5.16 |
| 24 | 01204 1 | 351 | NP_001023074 | Inorganic Pyrophosphatase | *C. elegans* | inorganic diphosphatase | 37.3/5.13 |
| 30 | 00280 1 | 344 | XP_001679131 | CBG03214 | *C. briggsae AF16* | phosphopyruvate hydratase | 46.6/5.41 |
| 32 | 00537 1 | 317 | NP_491955 | K02F2.2 | *C. elegans* | adenosylhomocysteinase | 47.5/6.19 |
| 34 | 00183 1 | 446 | XP_001666207 | CBG09180 | *C. briggsae AF16* | fumarate hydratase/lyase | 52.4/8.22 |
| 35 | 00183 1 | 446 | XP_001666207 | CBG09180 | *C. briggsae AF16* | fumarate hydratase/lyase | 52.4/8.22 |
| 37 | 01607 2 | 605 | NP_001021240 | F01F1.12 | *C. elegans* | fructose-biphosphate aldolase | 38.8/8.02 |
| 42 | 11007 1 | 336 | XP_001666501 | CBG15213 | *C. briggsae AF16* | malate dehydrogenase/oxidoreductase | 35.0/9.33 |
| 57 | 06327 1 | 298 | XP_001897798 | Ubiquitin conjugating enzyme E2 H | *B. malayi* | small conjugating protein ligase | 22.4/5.05 |
| 60 | 00814 1 | 328 | AAN05752 | heat shock protein 20 | *H. contortus* | chaperonin | 18.3/6.23 |
| 62 | 03240 3 | 297 | NP_001023903 | F40A3.3 | *C. elegans* | unknown | 20.3/6.81 |
| 63 | 03240 1 | 297 | NP_001023903 | F40A3.3 | *C. elegans* | unknown | 20.3/6.81 |
| 78 | 00413 1 | 170 | NP_001123180 | T08A9.11 | *C. elegans* | unknown | 15.7/4.90 |
| 80 | 11248 1 | 154 | AAN05752 | heat shock protein 20 | *H. contortus* | chaperonin | 18.3/6.23 |
| 82 | 00047 3 | 333 | Q27666 | Superoxide dismutase | *H. contortus* | metal ion binding | 16.6/6.58 |
| 90 | 00907 1 | 64 | NP_001024064 | R02C2.7 | *C. elegans* | unknown | 13.8/5.32 |
| 93 | 03264 2 | 157 | CAP20913 | CBG24261 | *C. briggsae* | unknown | 18.1/5.73 |
| 94 | 04833 1 | 188 | 2OS5_A | Macrophage Migration Inhibitory Factor | *A. ceylanicum* | unknown | 13.1/7.92 |
| 96 | 00229 1 | 159 | XP_001664602 | CBG11702 | *C. briggsae AF16* | chaperonin | 11.8/9.26 |

^1^ Wormbase was used to infer molecular function when a link from the NCBI nr protein database webpage of the protein in question was available. If not available, the closest *C. elegans* protein BLASTp hit (with the highest similarity score) Wormbase link was used to infer function in the same manner.

Each EST sequence hit was submitted to a BLASTp search against the entire NCBI nr protein database. For each search, the highest scoring hit score (significance threshold > 44, p-value < 0.01), its accession number and protein name are reported. For information, the species corresponding to the highest scoring hit, its molecular function according to the NCBI nr database and Wormbase when available^1^ and the theoretical Mw/p*I* of its full length sequence are also described.
