# Supplementary material for: Proteomic Profiling and Protein Identification by MALDI-TOF Mass Spectrometry in Unsequenced Parasitic Nematodes
Source: PLoS One. 2012 Mar 29;7(3):e33590. doi: 10.1371/journal.pone.0033590 (PMC3315570; doi:10.1371/journal.pone.0033590)
Supplement: Table S5 — Q-TOF MS/MS protein spot identifications using the H. contortus putative EST protein database. Each protein spot was excised from the 500 µg protein-loaded gel and analysed by Q-TOF MS/MS. A local MASCOT Ion search of the H. contortus putative EST protein database was performed and the highest scoring EST sequence match along with its MOWSE-based score (significance threshold score >25, p-value<0.05), sequence coverage and the number of matched peptides is reported. For each search, the highest scoring hit EST sequence accession number and its theoretical Mw/pI are also detailed. (DOC) [file pone.0033590.s007.doc]

**Table S5. Q-TOF MS/MS protein spot identifications using the *H. contortus* putative EST protein database.**

| Protein spot | Observed Mw/pI (kDa) | Mascot MOWSE Score | Sequence Coverage % | Matched Peptides (Total) | EST Accession Number | EST sequence Theoretical Mw/pI (kDa) |
| --- | --- | --- | --- | --- | --- | --- |
| 7 | 57.0/4.20 | 77 | 22 | 5 | 00592 1 | 46.9/4.77 |
| 12 | 59.8/6.60 | 659 | 42 | 19 | 00006 1 | 59.6/6.67 |
| 13 | 59.5/6.76 | 707 | 45 | 21 | 00006 1 | 59.6/6.67 |
| 16 | 51.9/3.98 | 342 | 33 | 6 | 00296 2 | 25.2/4.66 |
| 26 | 50.7/6.04 | 52 | 34 | 3 | 00280 1 | 20.5/9.14 |
| 28 | 41.2/6.99 | 404 | 28 | 13 | 00006 1 | 59.6/6.67 |
| 33 | 52.3/8.38 | 238 | 34 | 6 | 00183 1 | 29.6/7.11 |
| 34 | 48.2/7.85 | 56 | 24 | 3 | 00183 1 | 29.6/7.11 |
| 40 | 39.2/9.10 | 232 | 60 | 9 | 11007 1 | 23.8/8.12 |
| 42 | 38.4/9.36 | 333 | 63 | 8 | 11007 1 | 23.8/8.12 |
| 51 | 20.7/4.46 | 2727 | 46 | 10 | 00182 4 | 20.9/5.88 |
| 56 | 22.7/6.56 | 1839 | 13 | 6 | 00006 1 | 59.6/6.67 |
| 73 | 14.6/5.23 | 145 | 47 | 7 | 02740 1 | 15.1/6.15 |
| 76 | 12.9/5.19 | 158 | 33 | 4 | 00822 1 | 18.5/8.59 |
| 85 | 17.0/7.82 | 1595 | 25 | 5 | 00208 1 | 19.2/6.59 |
| 86 | 16.7/8.08 | 384 | 57 | 8 | 02230 1 | 14.1/7.68 |
| 88 | 16.1/7.94 | 67 | 31 | 3 | 00202 3 | 19.7/6.97 |
| 91 | 14.1/8.23 | 196 | 37 | 6 | 00372 1 | 24.7/7.82 |
| 96 | 11.7/7.46 | 114 | 54 | 4 | 00229 1 | 10.3/7.03 |
| 98 | 9.5/7.75 | 122 | 38 | 7 | 01027 1 | 21.2/9.72 |

Each protein spot was excised from the 500 μg protein-loaded gel and analysed by Q-TOF MS/MS. A local MASCOT Ion search of the *H. contortus* putative EST protein database was performed and the highest scoring EST sequence match along with its MOWSE-based score (significance threshold score > 25, p-value < 0.05), sequence coverage and the number of matched peptides is reported. For each search, the highest scoring hit EST sequence accession number and its theoretical Mw/p*I* are also detailed.
