# Supplementary material for: Proteomic Profiling and Protein Identification by MALDI-TOF Mass Spectrometry in Unsequenced Parasitic Nematodes
Source: PLoS One. 2012 Mar 29;7(3):e33590. doi: 10.1371/journal.pone.0033590 (PMC3315570; doi:10.1371/journal.pone.0033590)
Supplement: Table S6 — Annotation of the hit EST sequences from Table S5 using BLASTp searches against the entire NCBI nr protein database. Each EST sequence hit was submitted to a BLASTp search against the entire NCBI nr protein database. For each search, the highest scoring hit score (significance threshold >44, p-value<0.01), its accession number and protein name are reported. For information, the species for the highest scoring hit, its molecular function according to Wormbase1 and the theoretical Mw/pI of its sequence are also described. (DOC) [file pone.0033590.s008.doc]

**Table S6.** Annotation of the hit EST sequences from Table S5 using BLASTp searches against the entire NCBI nr protein database.

| Protein spot | EST Accession Number | BLAST score | Accession Number | Protein Identified | Species | Molecular Function | Theoretical Mw/pI of full length sequence (kDa) |
| --- | --- | --- | --- | --- | --- | --- | --- |
| 7 | 00592 1 | 540 | CAL30086 | Calreticulin precursor | *H. polygyrus* | calcium binding chaperonin | 47.0/4.74 |
| 12 | 00006 1 | 1077 | ACT34056 | Putative Glutamate deHydrogenase | *H. contortus* | oxidoreductase | 59.1/6.67 |
| 13 | 00006 1 | 1077 | ACT34056 | Putative Glutamate deHydrogenase | *H. contortus* | oxidoreductase | 59.1/6.67 |
| 16 | 00296 2 | 388 | NP_001024806 | Calumenin | *C. elegans* | calcium ion binding | 36.0/4.64 |
| 26 | 00280 1 | 344 | XP_001679131 | CBG03214 | *C. briggsae AF16* | phosphopyruvate hydratase | 46.6/5.41 |
| 28 | 00006 1 | 1077 | ACT34056 | Putative Glutamate deHydrogenase | *H. contortus* | oxidoreductase | 59.1/6.67 |
| 33 | 00183 1 | 446 | XP_001666207 | CBG09180 | *C. briggsae AF16* | fumarate hydratase/lyase | 52.4/8.22 |
| 34 | 00183 1 | 446 | XP_001666207 | CBG09180 | *C. briggsae AF16* | fumarate hydratase/lyase | 52.4/8.22 |
| 40 | 11007 1 | 336 | XP_001666501 | CBG15213 | *C. briggsae AF16* | malate dehydrogenase/oxidoreductase | 35.0/9.33 |
| 42 | 11007 1 | 336 | XP_001666501 | CBG15213 | *C. briggsae AF16* | malate dehydrogenase/oxidoreductase | 35.0/9.33 |
| 51 | 00182 4 | 362 | CAJ09947 | NIM-1 protein | *H. contortus* | unknown | 19.4/5.13 |
| 56 | 00006 1 | 1077 | ACT34056 | Putative Glutamate deHydrogenase | *H. contortus* | oxidoreductase | 59.1/6.67 |
| 73 | 02740 1 | 167 | NP_001033512 | Lipid Binding Protein | *C. elegans* | lipid binding/transporter | 16.9/7.93 |
| 76 | 00822 1 | 303 | XP 001675459 | CBG18577 | *C. briggsae AF16* | peptidyl-prolyl cis-trans isomerase | 18.4/8.68 |
| 85 | 00208 1 | 197 | P27613 | Globin-like host protective antigen | *T. colubriformis* | heme/iron ion/oxygen binding | 19.9/7.03 |
| 86 | 02230 1 | 261 | ABJ97284 | major sperm protein | *D. viviparus* | structurale molecule | 14.2/7.71 |
| 88 | 00202 3 | 210 | P27613 | Globin-like host protective antigen | *T. colubriformis* | heme/iron ion/oxygen binding | 19.9/7.03 |
| 91 | 00372 1 | 210 | CAP20913 | CBG24261 | *C. briggsae* | unknown | 18.1/5.73 |
| 96 | 00229 1 | 159 | XP_001664602 | CBG11702 | *C. briggsae AF16* | chaperonin | 11.8/9.26 |
| 98 | 01027 1 | 290 | XP 0024231426 | ubiquitin, putative | *P. humanus corporis* | unknown | 94.7/7.69 |

^1^ Wormbase was used to infer molecular function when a link from the NCBI nr protein database webpage of the protein in question was available. If not available, the closest *C. elegans* protein BLASTp hit (with the highest similarity score) Wormbase link was used to infer function in the same manner.

Each EST sequence hit was submitted to a BLASTp search against the entire NCBI nr protein database. For each search, the highest scoring hit score (significance threshold > 44, p-value < 0.01), its accession number and protein name are reported. For information, the species for the highest scoring hit, its molecular function according to Wormbase^1^ and the theoretical Mw/pI of its sequence are also described.
