# Supplementary material for: Proteomic Profiling and Protein Identification by MALDI-TOF Mass Spectrometry in Unsequenced Parasitic Nematodes
Source: PLoS One. 2012 Mar 29;7(3):e33590. doi: 10.1371/journal.pone.0033590 (PMC3315570; doi:10.1371/journal.pone.0033590)
Supplement: Table S7 — Q-TOF MS/MS protein spot identifications using FASTS against the NCBI nr protein database. Each protein spot was excised from the 500 µg protein-loaded gel and analysed by Q-TOF MS/MS. Selected peptides were then subjected to manual de novo sequencing and reported in this table. These peptide sequences were finally submitted to a FASTS search against the entire NCBI nr protein database. For each search, the highest scoring hit score (significance threshold >44, p-value<0.01) and corresponding e-value, accession number, protein name and species are also reported. Identical amino-acids between query sequence and FASTS search highest scoring hit are shown as underlined text. (DOC) [file pone.0033590.s009.doc]

**Table S7.** Q-TOF MS/MS protein spot identifications using FASTS against the NCBI nr protein database.

| Protein spot | MSMS sequence | Ion | Protein | Accession | Species | Amino-acid identity (%) | Amino-acid similarity (%) | Protein Length (aa) | Score (bits) | E-value | Already identified by other techniques (Corresponding Table in brackets) |
| --- | --- | --- | --- | --- | --- | --- | --- | --- | --- | --- | --- |
| 7 | FLDDSWEK | 650.37 | Calreticulin precursor | CAL30086.1 | *H. polygyrus* | 77.8 | 83.3 | 403 | 44.4 | 0.0058 | MALDI-TOF PMF similarity (S4) |
|  | VDTDSVDDAK | 957.91 |  |  |  |  |  |  |  |  | MS/MS/BLAST (S6) |
| 12 | FLLKSVQDSLEK | 703.80 | Glutamate dehydrogenase | ACT34055.1 | *H. contortus* | 94.6 | 94.6 | 532 | 120.4 | 1.6e-25 | MALDI-TOF/PMF (S2) |
|  | EAPVHPNDEFTAR | 741.79 |  |  |  |  |  |  |  |  | MS/MS/BLAST (S6) |
|  | DVPAPDMGTGER | 936.38 |  |  |  |  |  |  |  |  |  |
| 13 | NDGEFEVLEAWR | 732.74 | Glutamate dehydrogenase | ACT34055.1 | *H. contortus* | 85.7 | 94.3 | 532 | 103.6 | 1.7e-20 | MALDI-TOF/PMF (S2) |
|  | EAADGPTTPAADK | 770.84 |  |  |  |  |  |  |  |  | MS/MS/BLAST (S6) |
|  | DWRDENGTLK | 794.35 |  |  |  |  |  |  |  |  |  |
| 16 | DMSDGDGFVEEK | 664.74 | CALUmenin | NP_001024806.1 | *C. elegans* | 82.5 | 87.5 | 314 | 101.8 | 4.0e-20 | MS/MS/BLAST (S6) |
|  | DTAAEFDELTPEK | 733.31 |  |  |  |  |  |  |  |  |  |
|  | DVVVAETVDDLDKNK | 830.37 |  |  |  |  |  |  |  |  |  |
| 26 | GASTGVHEALELR | 590.29 | Enolase | ADK47524.1 | *H. contortus* | 83.3 | 83.3 | 434 | 61.3 | 5.3e-08 | MS/MS/BLAST (S6) |
|  | LPVKTSFHEAM | 815.39 |  |  |  |  |  |  |  |  |  |
| 28 | VLYLTFLPR | 561.31 | CRE_23811 | XP_003089209.1 | *C. elegans* | 73.3 | 80.0 | 231 | 62.5 | 5.0e-08 | MALDI-TOF/PMF (S2) |
|  | QYTDYELEK | 594.75 |  |  |  |  |  |  |  |  | MS/MS/BLAST (S6) |
|  | NDGEFEVLEANR | 732.81 |  |  |  |  |  |  |  |  |  |
| 33 | TVWLTGLPFK | 581.38 | Fumarase | AAP51177.1 | *A. suum* | 80.6 | 83.3 | 467 | 72.7 | 3.0e-11 | MS/MS/BLAST (S6) |
|  | DTFGELEVWPR | 674.88 |  |  |  |  |  |  |  |  |  |
|  | LAAGGTAVGTGLNTR | 882.49 |  |  |  |  |  |  |  |  |  |
| 40 | LFGVTTLDVVR | 610.44 | CBR-MDH-1 protein | XP_002642936.1 | *C. briggsae* | 87.9 | 87.9 | 341 | 98.2 | 4.4e-19 | MALDI-TOF/PMF (S2) |
|  | FNTNAGLVR | 667.90 |  |  |  |  |  |  |  |  | MS/MS/BLAST (S6) |
|  | NADVLVLPAGVPR | 974.69 |  |  |  |  |  |  |  |  |  |
| 42 | LFGVTTLDVVR | 610.30 | Malate deHydrogenase | ABU25173.1 | *L. guyanensis* | 64.7 | 67.6 | 317 | 54.3 | 7.0e-06 | MALDI-TOF PMF similarity (S4) |
|  | LDVDVLAVEAPK | 634.85 |  |  |  |  |  |  |  |  | MS/MS/BLAST (S6) |
|  | DDLFNTNAGLVR | 667.77 |  |  |  |  |  |  |  |  |  |
| 51 | FDVSKDNTATQK | 597.93 | NIM-1 protein | CAJ09947.1 | *H. contortus* | 100 | 100 | 178 | 116.9 | 4.7e-25 | MS/MS/BLAST (S6) |
|  | SPLVTAAMPLAGVDNEK | 856.94 |  |  |  |  |  |  |  |  |  |
| 56 | HPNDFETAR | 494.94 | Glutamate dehydrogenase | ACT34055.1 | *H. contortus* | 78.4 | 81.1 | 532 | 73.5 | 2.1e-11 | MS/MS/BLAST (S6) |
|  | FLLQADKDSLEK | 703.90 |  |  |  |  |  |  |  |  |  |
|  | LLAEAANGPTTPAADK | 770.41 |  |  |  |  |  |  |  |  |  |
| 73 | NLKPTLEFVK | 729.37 | CRE_27196 | XP_003114500.1 | *C. elegans* | 66.7 | 70.0 | 136 | 49.9 | 5.6e-05 | MALDI-TOF/PMF (S2) |
|  | MTSVSTFK | 798.34 |  |  |  |  |  |  |  |  | MS/MS/BLAST (S6) |
|  | FVSSENFEAVFK | 878.36 |  |  |  |  |  |  |  |  |  |
| 76 | VTEGMDVVK | 489.21 | Peptidyl-prolyl cis-trans isomerase 3 | ADY49503.1 | *A. suum* | 88.9 | 94.4 | 157 | 59.9 | 3.8e-08 | MS/MS/BLAST (S6) |
|  | GGESLYGEK | 627.28 |  |  |  |  |  |  |  |  |  |
| 85 | YFLGAESLTPDEVDK | 842.43 | Globin-like ES protein F6 | CAD20463.1 | *O. ostertagi* | 68.8 | 75.0 | 138 | 67.7 | 2.6e-10 | MALDI-TOF PMF similarity (S4) |
|  | NAVAALEHAPLGTTPEK | 859.96 |  |  |  |  |  |  |  |  | MS/MS/BLAST (S6) |
| 86 | LTVEWHHTPEGAAK | 788.32 | Major sperm protein 1 | BAI81973.1 | *N. brasiliensis* | 64.3 | 75.0 | 117 | 60.8 | 2.2e-08 | MALDI-TOF PMF similarity (S4) |
|  | LMAVSGCDVFDYGR | 918.96 |  |  |  |  |  |  |  |  | MS/MS/BLAST (S6) |
| 88 | SFTADDVQNCLR | 834.95 | Globin-like ES protein F6 | CAD20463.1 | *O. ostertagi* | 76.9 | 80.8 | 138 | 63.0 | 5.3e-09 | MALDI-TOF/PMF (S2) |
|  | AALESVPVGTTPDK | 845.90 |  |  |  |  |  |  |  |  | MS/MS/BLAST (S6) |
| 91 | LYLSGSLGLDPK | 846.40 | CRE_02928 | XP_003111494.1 | *C. elegans* | 70.8 | 83.3 | 171 | 58.6 | 1.3e-07 | MALDI-TOF/PMF (S2) |
|  | LNDFTTVNDLYK | 1078.98 |  |  |  |  |  |  |  |  | MS/MS/BLAST (S6) |
| 96 | VLEATVLAVGE | 676.88 | 10 kDa heat shock protein | ACO51597.1 | *R. catesbeiana* | 90.5 | 95.5 | 102 | 67.7 | 1.2e-10 | MALDI-TOF PMF similarity (S4) |
|  | VLLPEYGGTK | 736.86 |  |  |  |  |  |  |  |  | MS/MS/BLAST (S6) |
| 98 | STLHLVLR | 526.84 | Ubiquitin | ACW20129.1 | *S. nudus* | 93.3 | 93.3 | 25 | 57.4 | 2.8e-08 | MS/MS/BLAST (S6) |
|  | SDYNLQK | 542.83 |  |  |  |  |  |  |  |  |  |

Each protein spot was excised from the 500 μg protein-loaded gel and analysed by Q-TOF MS/MS. Selected peptides were then subjected to manual *de novo* sequencing and reported in this table. These peptide sequences were finally submitted to a FASTS search against the entire NCBI nr protein database. For each search, the highest scoring hit score (significance threshold > 44, p-value < 0.01) and corresponding e-value, accession number, protein name and species are also reported. Identical amino-acids between query sequence and FASTS search highest scoring hit are shown as underlined text.
